# Supplementary material for: Untargeted Lipidomics after D2O Administration Reveals the Turnover Rate of Individual Lipids in Various Organs of Living Organisms
Source: Int J Mol Sci. 2023 Jul 21;24(14):11725. doi: 10.3390/ijms241411725 (PMC10380497; doi:10.3390/ijms241411725)
Supplement: Supplementary file 1 [file ijms-24-11725-s001.zip › Supporting Info 2. Deuterium distribution for all organs by FisherPy.pdf]

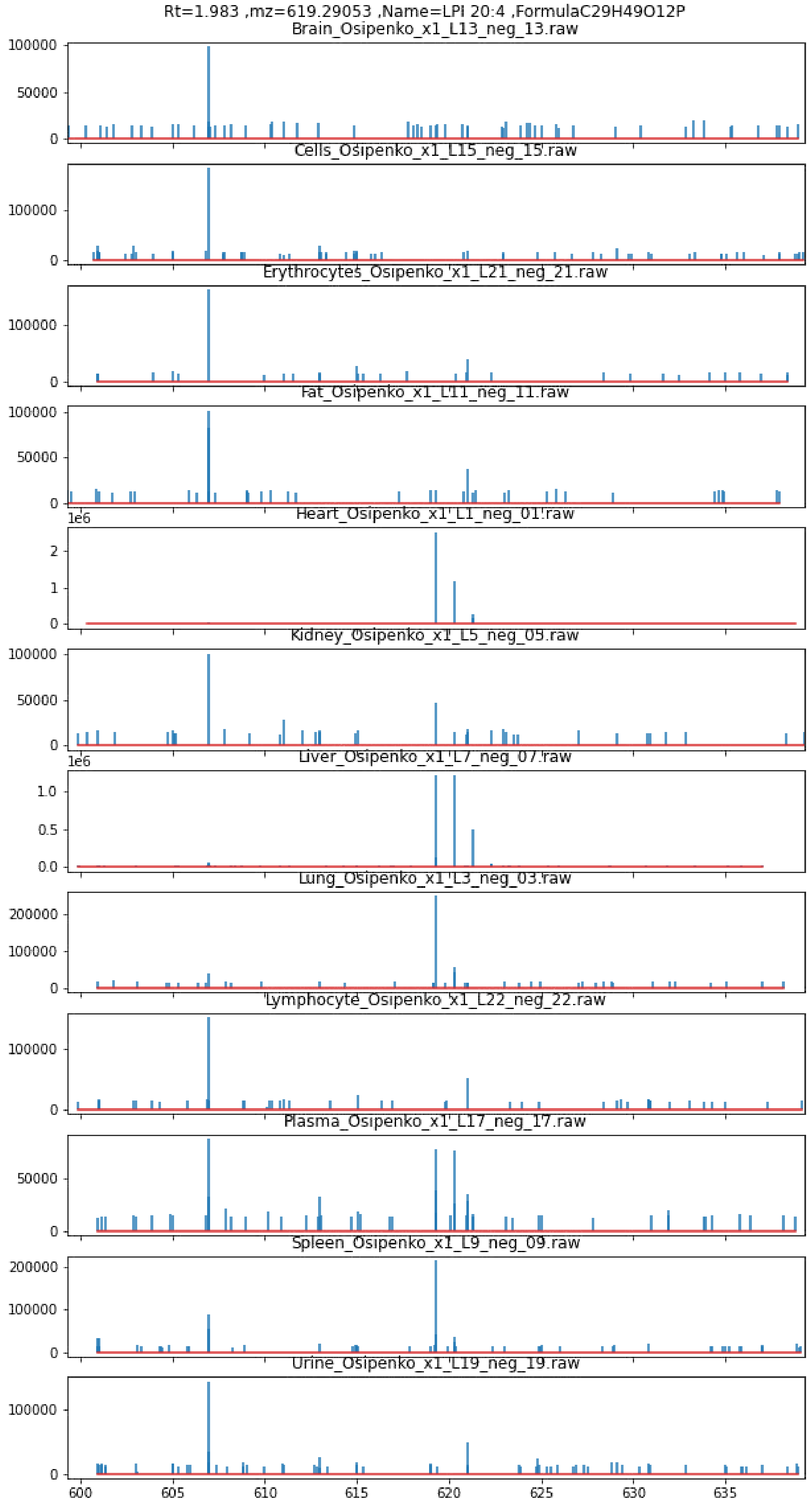
Rt=1.983 ,mz=619.29053 ,Name=LPI 20:4 ,FormulaC29H49O12P
Brain_Osipenko_x1_L13_neg_13.raw
Cells_Osipenko_x1_L15_neg_15.raw
Erythrocytes_Osipenko_x1_L21_neg_21.raw
Fat_Osipenko_x1_L11_neg_11.raw
Heart_Osipenko_x1_L1_neg_01.raw
Kidney_Osipenko_x1_L5_neg_05.raw
Liver_Osipenko_x1_L7_neg_07.raw
Lung_Osipenko_x1_L3_neg_03.raw
Lymphocyte_Osipenko_x1_L22_neg_22.raw
Plasma_Osipenko_x1_L17_neg_17.raw
Spleen_Osipenko_x1_L9_neg_09.raw
Urine_Osipenko_x1_L19_neg_19.raw
1e6
1e6
600
605
610
615
620
625
630
635

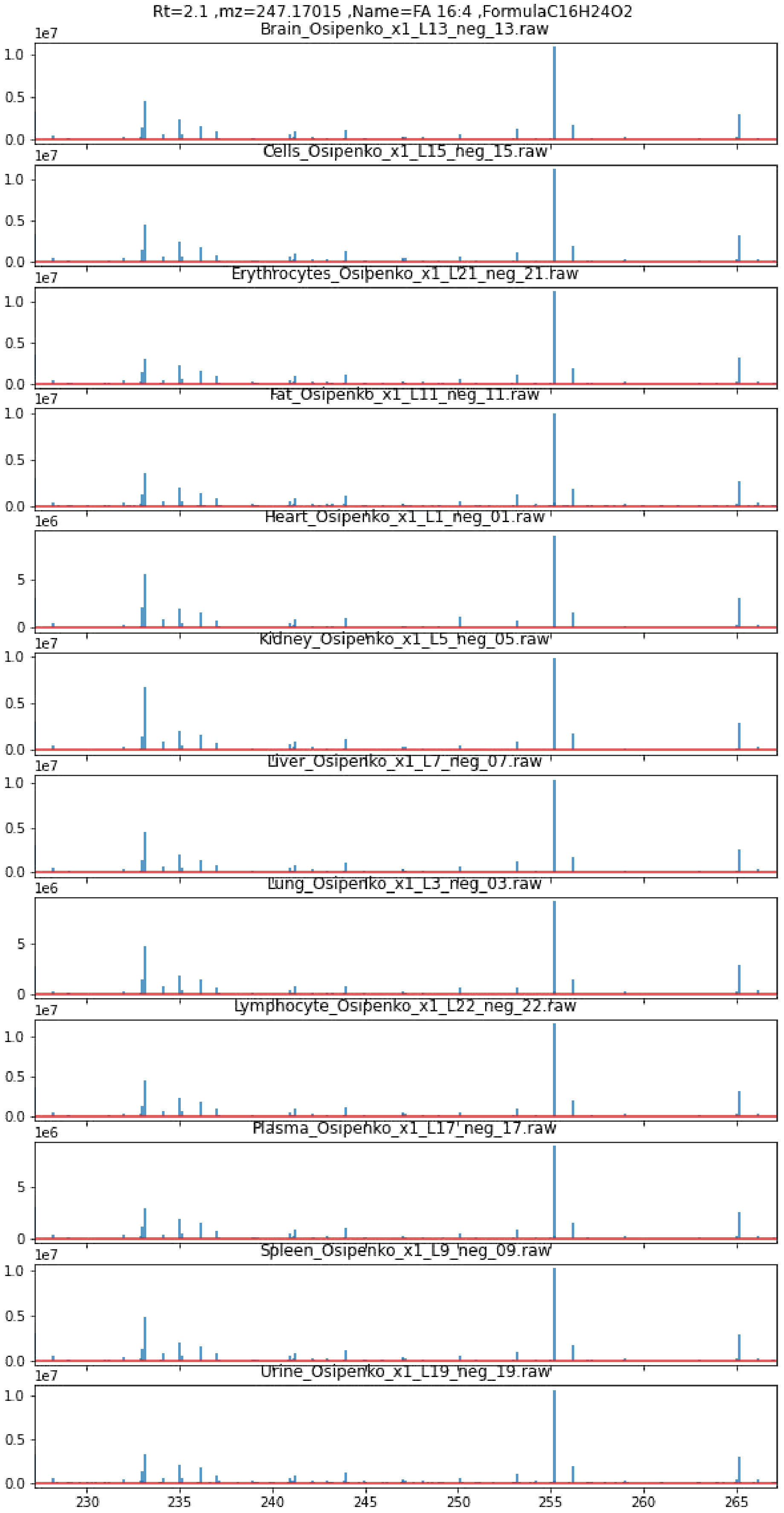

Rt=2.1 ,mz=247.17015 ,Name=FA 16:4 ,FormulaC16H24O2
Brain_Osipenko_x1_L13_neg_13.raw
Cells_Osipenko_x1_L15_neg_15.raw
Erythrocytes_Osipenko_x1_L21_neg_21.raw
Fat_Osipenko_x1_L11_neg_11.raw
Heart_Osipenko_x1_L1_neg_01.raw
Kidney_Osipenko_x1_L5_neg_05.raw
Liver_Osipenko_x1_L7_neg_07.raw
Lung_Osipenko_x1_L3_neg_03.raw
Lymphocyte_Osipenko_x1_L22_neg_22.raw
Plasma_Osipenko_x1_L17_neg_17.raw
Spleen_Osipenko_x1_L9_neg_09.raw
Urine_Osipenko_x1_L19_neg_19.raw
1e7
1e6
230
235
240
245
250
255
260
265

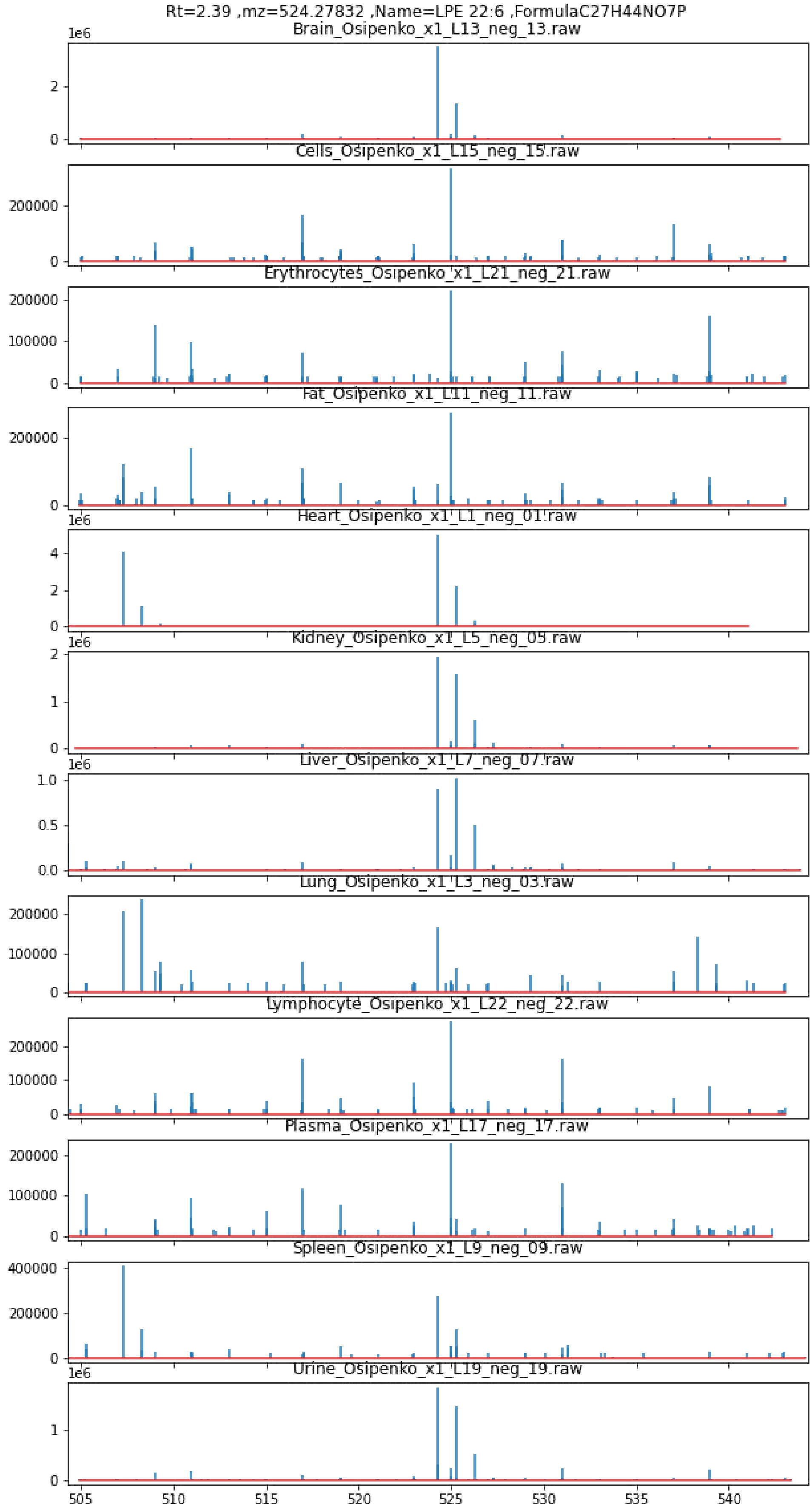

Rt=2.39 ,mz=524.27832 ,Name=LPE 22:6 ,FormulaC27H44NO7P
Brain_Osipenko_x1_L13_neg_13.raw
Cells_Osipenko_x1_L15_neg_15.raw
Erythrocytes_Osipenko_x1_L21_neg_21.raw
Fat_Osipenko_x1_L11_neg_11.raw
Heart_Osipenko_x1_L1_neg_01.raw
Kidney_Osipenko_x1_L5_neg_05.raw
Liver_Osipenko_x1_L7_neg_07.raw
Lung_Osipenko_x1_L3_neg_03.raw
Lymphocyte_Osipenko_x1_L22_neg_22.raw
Plasma_Osipenko_x1_L17_neg_17.raw
Spleen_Osipenko_x1_L9_neg_09.raw
Urine_Osipenko_x1_L19_neg_19.raw
505
510
515
520
525
530
535
540

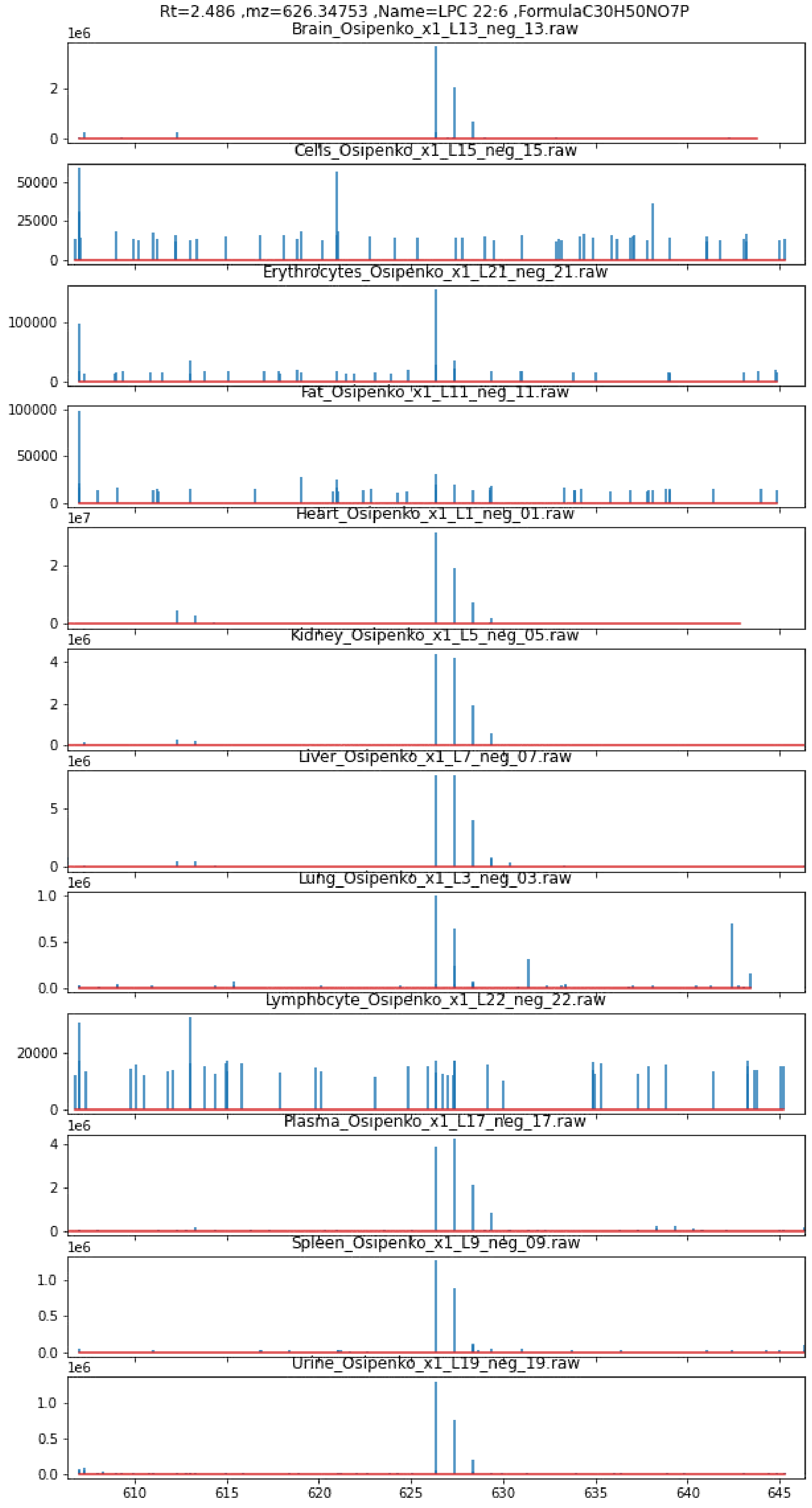
Rt=2.486 ,mz=626.34753 ,Name=LPC 22:6 ,FormulaC30H50NO7P
Brain_Osipenko_x1_L13_neg_13.raw
Cells_Osipenko_x1_L15_neg_15.raw
Erythrocytes_Osipenko_x1_L21_neg_21.raw
Fat_Osipenko_x1_L11_neg_11.raw
Heart_Osipenko_x1_L1_neg_01.raw
Kidney_Osipenko_x1_L5_neg_05.raw
Liver_Osipenko_x1_L7_neg_07.raw
Lung_Osipenko_x1_L3_neg_03.raw
Lymphocyte_Osipenko_x1_L22_neg_22.raw
Plasma_Osipenko_x1_L17_neg_17.raw
Spleen_Osipenko_x1_L9_neg_09.raw
Urine_Osipenko_x1_L19_neg_19.raw
610
615
620
625
630
635
640
645

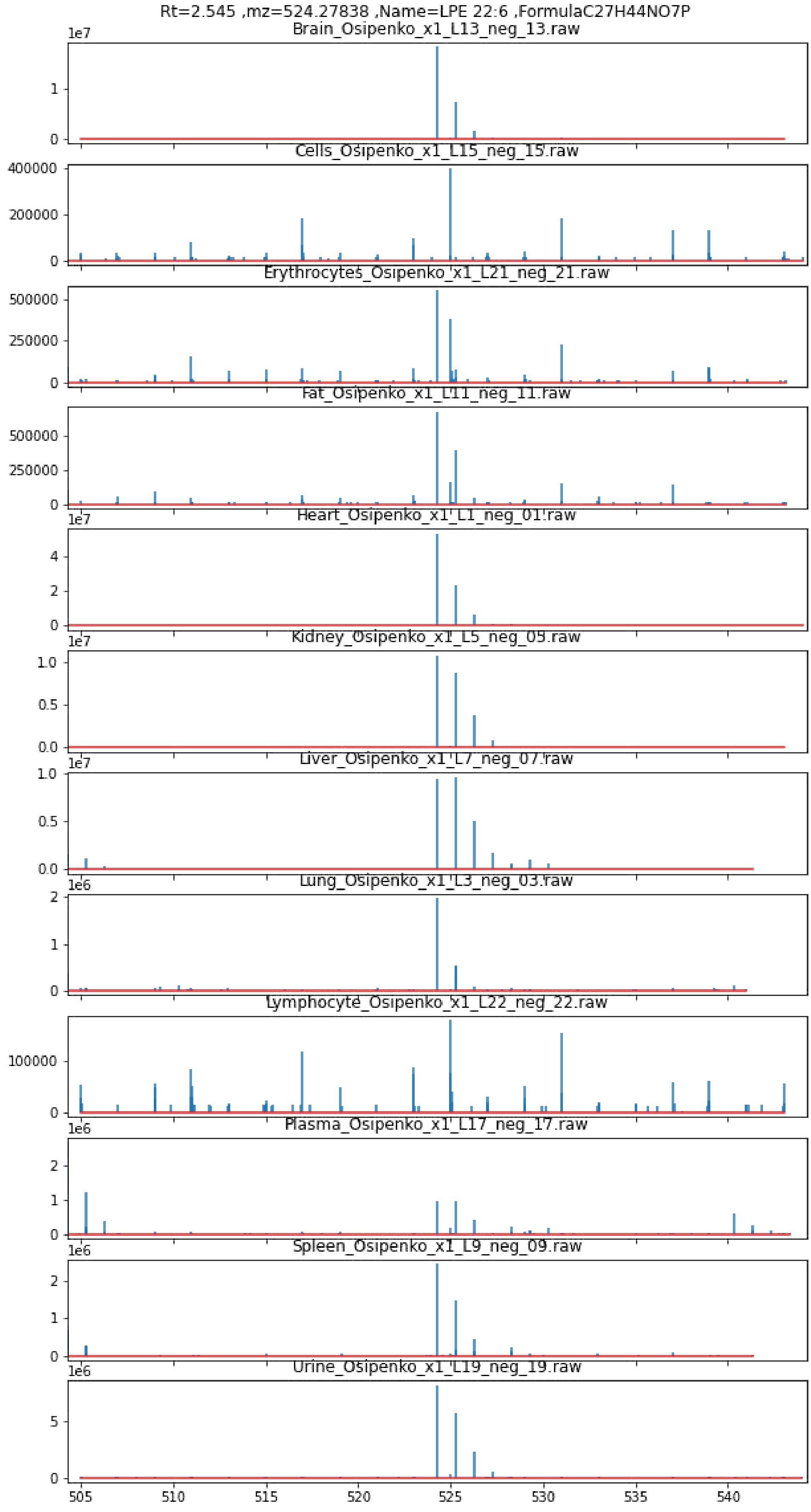

Rt=2.545 ,mz=524.27838 ,Name=LPE 22:6 ,FormulaC27H44NO7P
Brain_Osipenko_x1_L13_neg_13.raw
Cells_Osipenko_x1_L15_neg_15.raw
Erythrocytes_Osipenko_x1_L21_neg_21.raw
Fat_Osipenko_x1_L11_neg_11.raw
Heart_Osipenko_x1_L1_neg_01.raw
Kidney_Osipenko_x1_L5_neg_05.raw
Liver_Osipenko_x1_L7_neg_07.raw
Lung_Osipenko_x1_L3_neg_03.raw
Lymphocyte_Osipenko_x1_L22_neg_22.raw
Plasma_Osipenko_x1_L17_neg_17.raw
Spleen_Osipenko_x1_L9_neg_09.raw
Urine_Osipenko_x1_L19_neg_19.raw
505
510
515
520
525
530
535
540

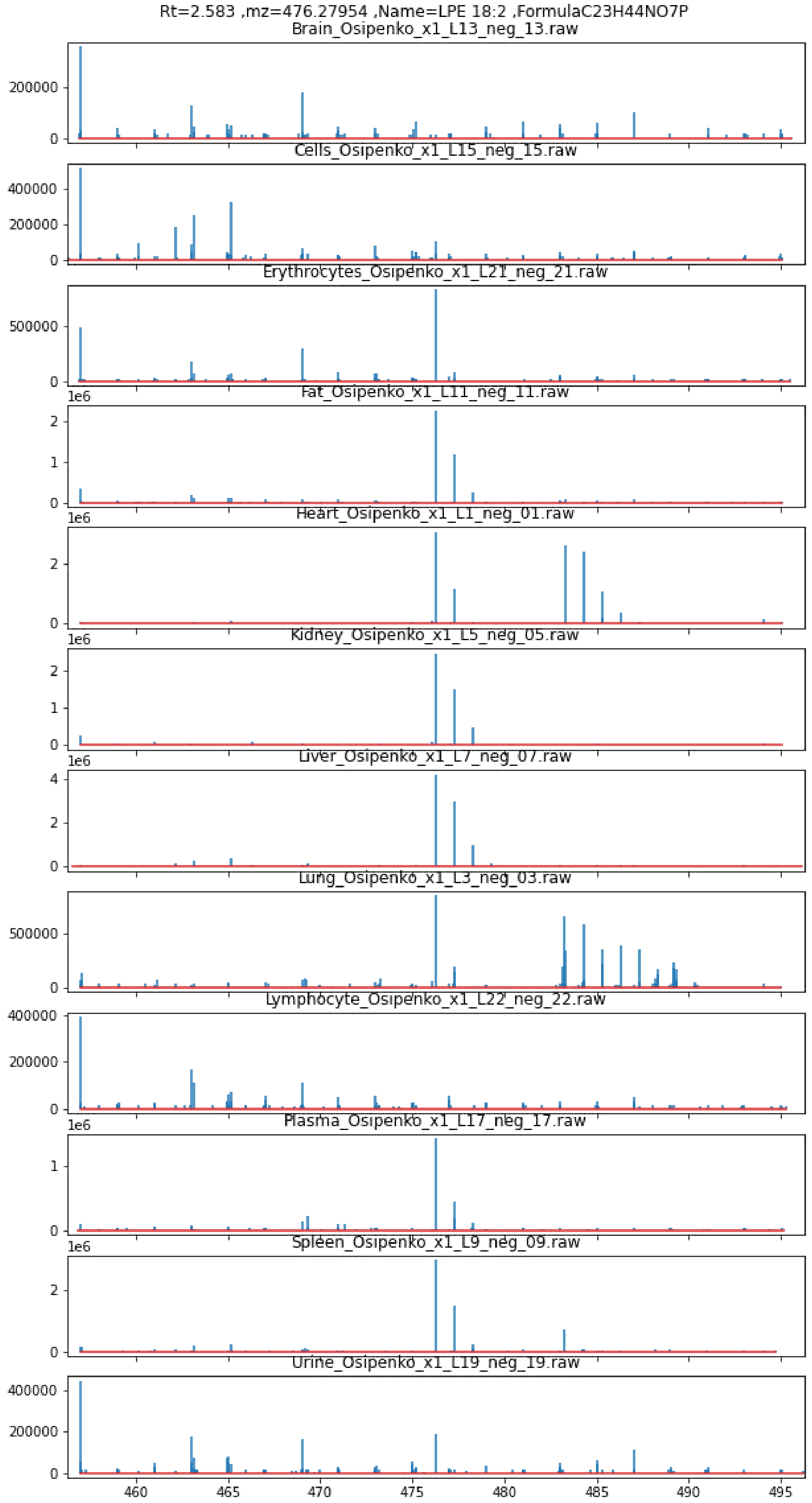

Rt=2.583 ,mz=476.27954 ,Name=LPE 18:2 ,FormulaC23H44NO7P
Brain_Osipenko_x1_L13_neg_13.raw
Cells_Osipenko_x1_L15_neg_15.raw
Erythrocytes_Osipenko_x1_L21_neg_21.raw
Fat_Osipenko_x1_L11_neg_11.raw
Heart_Osipenko_x1_L1_neg_01.raw
Kidney_Osipenko_x1_L5_neg_05.raw
Liver_Osipenko_x1_L7_neg_07.raw
Lung_Osipenko_x1_L3_neg_03.raw
Lymphocyte_Osipenko_x1_L22_neg_22.raw
Plasma_Osipenko_x1_L17_neg_17.raw
Spleen_Osipenko_x1_L9_neg_09.raw
Urine_Osipenko_x1_L19_neg_19.raw
1e6
460
465
470
475
480
485
490
495

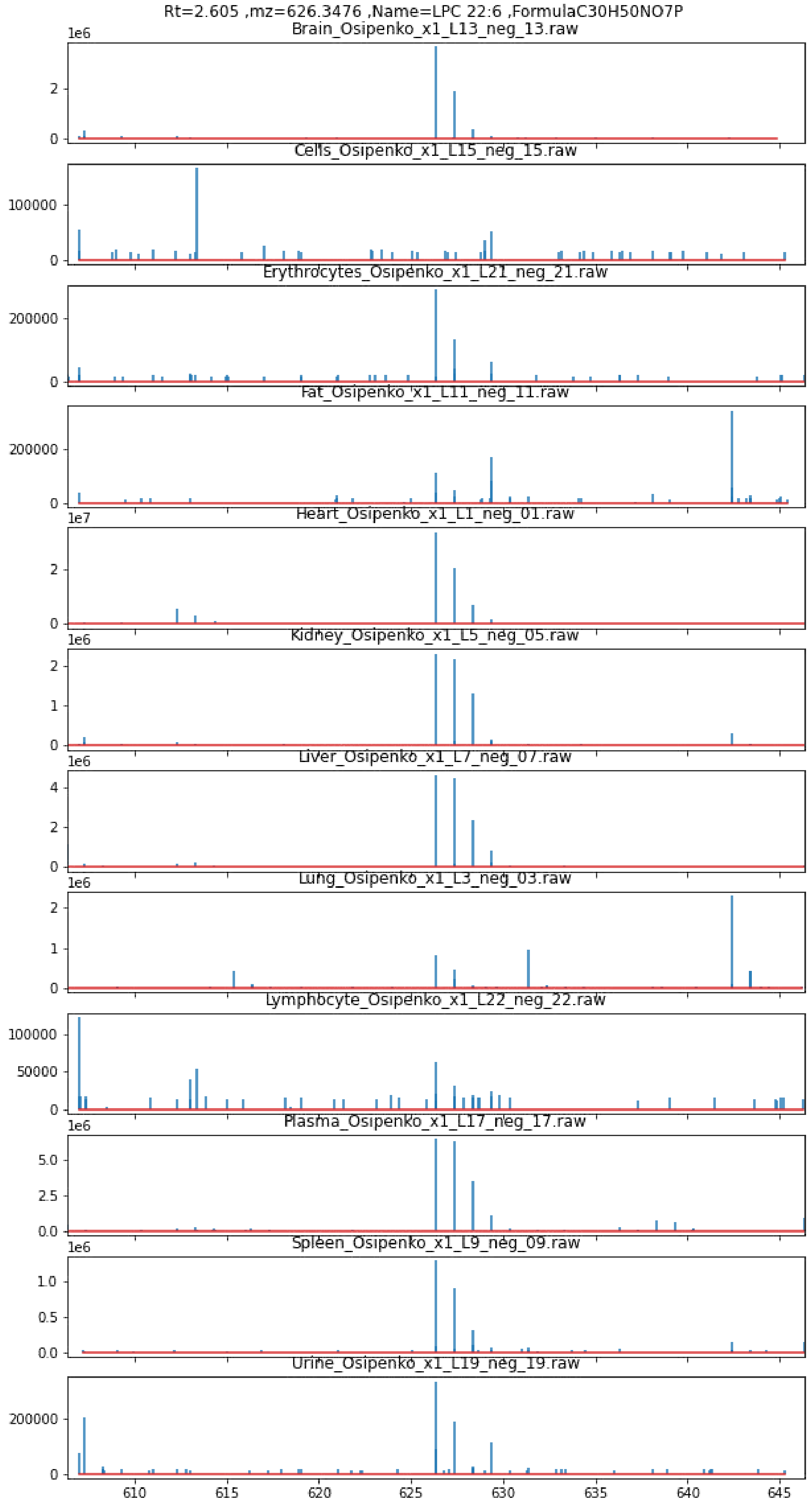
Rt=2.605 ,mz=626.3476 ,Name=LPC 22:6 ,FormulaC30H50NO7P
Brain_Osipenko_x1_L13_neg_13.raw
Cells_Osipenko_x1_L15_neg_15.raw
Erythrocytes_Osipenko_x1_L21_neg_21.raw
Fat_Osipenko_x1_L11_neg_11.raw
Heart_Osipenko_x1_L1_neg_01.raw
Kidney_Osipenko_x1_L5_neg_05.raw
Liver_Osipenko_x1_L7_neg_07.raw
Lung_Osipenko_x1_L3_neg_03.raw
Lymphocyte_Osipenko_x1_L22_neg_22.raw
Plasma_Osipenko_x1_L17_neg_17.raw
Spleen_Osipenko_x1_L9_neg_09.raw
Urine_Osipenko_x1_L19_neg_19.raw
1e6
1e7
610
615
620
625
630
635
640
645

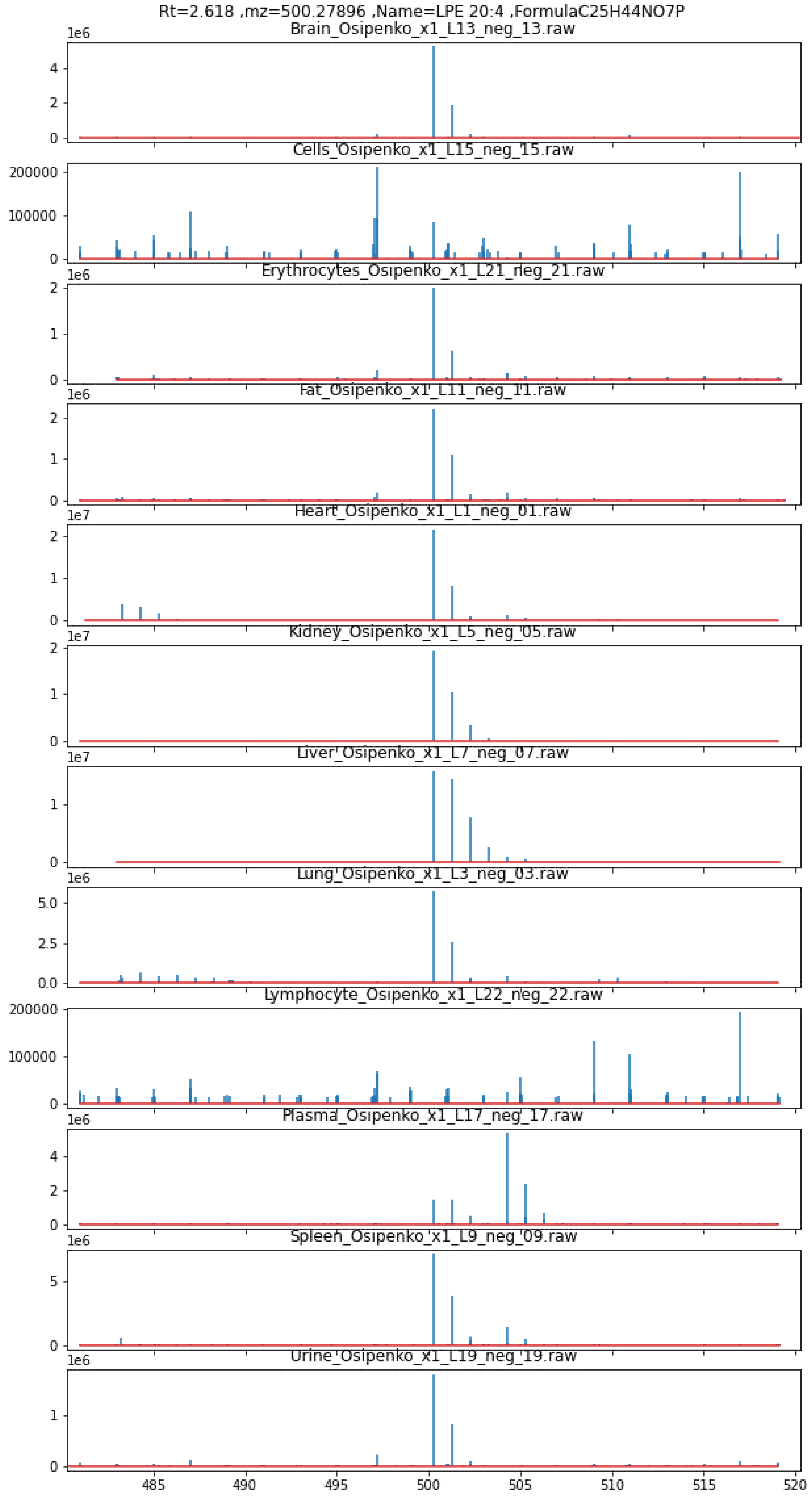
Rt=2.618 ,mz=500.27896 ,Name=LPE 20:4 ,FormulaC25H44NO7P
Brain_Osipenko_x1_L13_neg_13.raw
Cells_Osipenko_x1_L15_neg_15.raw
Erythrocytes_Osipenko_x1_L21_neg_21.raw
Fat_Osipenko_x1_L11_neg_11.raw
Heart_Osipenko_x1_L1_neg_01.raw
Kidney_Osipenko_x1_L5_neg_05.raw
Liver_Osipenko_x1_L7_neg_07.raw
Lung_Osipenko_x1_L3_neg_03.raw
Lymphocyte_Osipenko_x1_L22_neg_22.raw
Plasma_Osipenko_x1_L17_neg_17.raw
Spleen_Osipenko_x1_L9_neg_09.raw
Urine_Osipenko_x1_L19_neg_19.raw
1e6
1e7
485
490
495
500
505
510
515
520

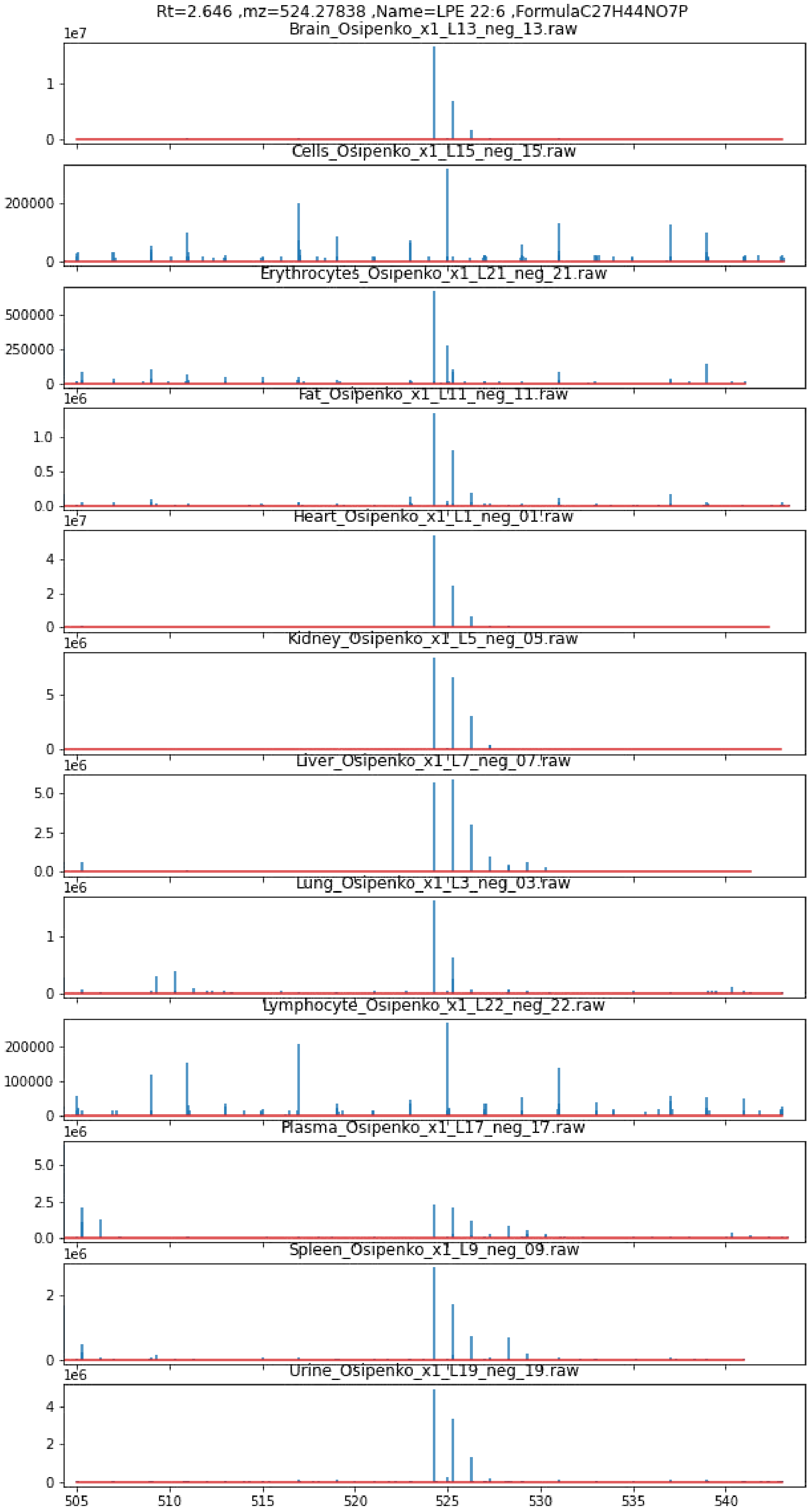

Rt=2.646 ,mz=524.27838 ,Name=LPE 22:6 ,FormulaC27H44NO7P
Brain_Osipenko_x1_L13_neg_13.raw
Cells_Osipenko_x1_L15_neg_15.raw
Erythrocytes_Osipenko_x1_L21_neg_21.raw
Fat_Osipenko_x1_L11_neg_11.raw
Heart_Osipenko_x1_L1_neg_01.raw
Kidney_Osipenko_x1_L5_neg_05.raw
Liver_Osipenko_x1_L7_neg_07.raw
Lung_Osipenko_x1_L3_neg_03.raw
Lymphocyte_Osipenko_x1_L22_neg_22.raw
Plasma_Osipenko_x1_L17_neg_17.raw
Spleen_Osipenko_x1_L9_neg_09.raw
Urine_Osipenko_x1_L19_neg_19.raw

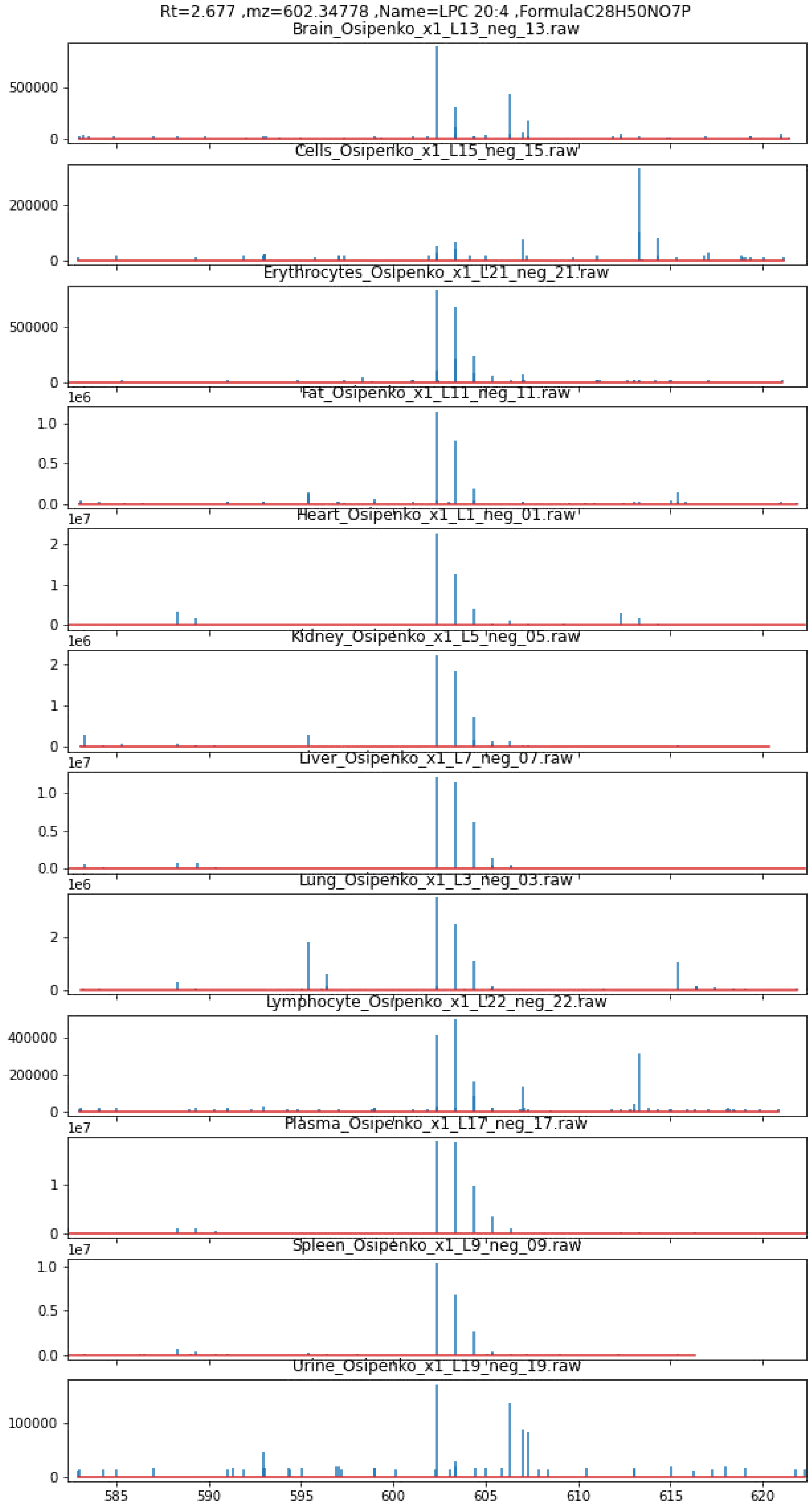
Rt=2.677 ,mz=602.34778 ,Name=LPC 20:4 ,FormulaC28H50NO7P
Brain_Osipenko_x1_L13_neg_13.raw
500000
0
Cells_Osipenko_x1_L15_neg_15.raw
200000
0
Erythrocytes_Osipenko_x1_L21_neg_21.raw
500000
0
1e6
Fat_Osipenko_x1_L11_neg_11.raw
1.0
0.5
0.0
1e7
Heart_Osipenko_x1_L1_neg_01.raw
2
1
0
1e6
Kidney_Osipenko_x1_L5_neg_05.raw
2
1
0
1e7
Liver_Osipenko_x1_L7_neg_07.raw
1.0
0.5
0.0
1e6
Lung_Osipenko_x1_L3_neg_03.raw
2
0
Lymphocyte_Osipenko_x1_L22_neg_22.raw
400000
200000
0
1e7
Plasma_Osipenko_x1_L17_neg_17.raw
1
0
1e7
Spleen_Osipenko_x1_L9_neg_09.raw
1.0
0.5
0.0
Urine_Osipenko_x1_L19_neg_19.raw
100000
0
585
590
595
600
605
610
615
620

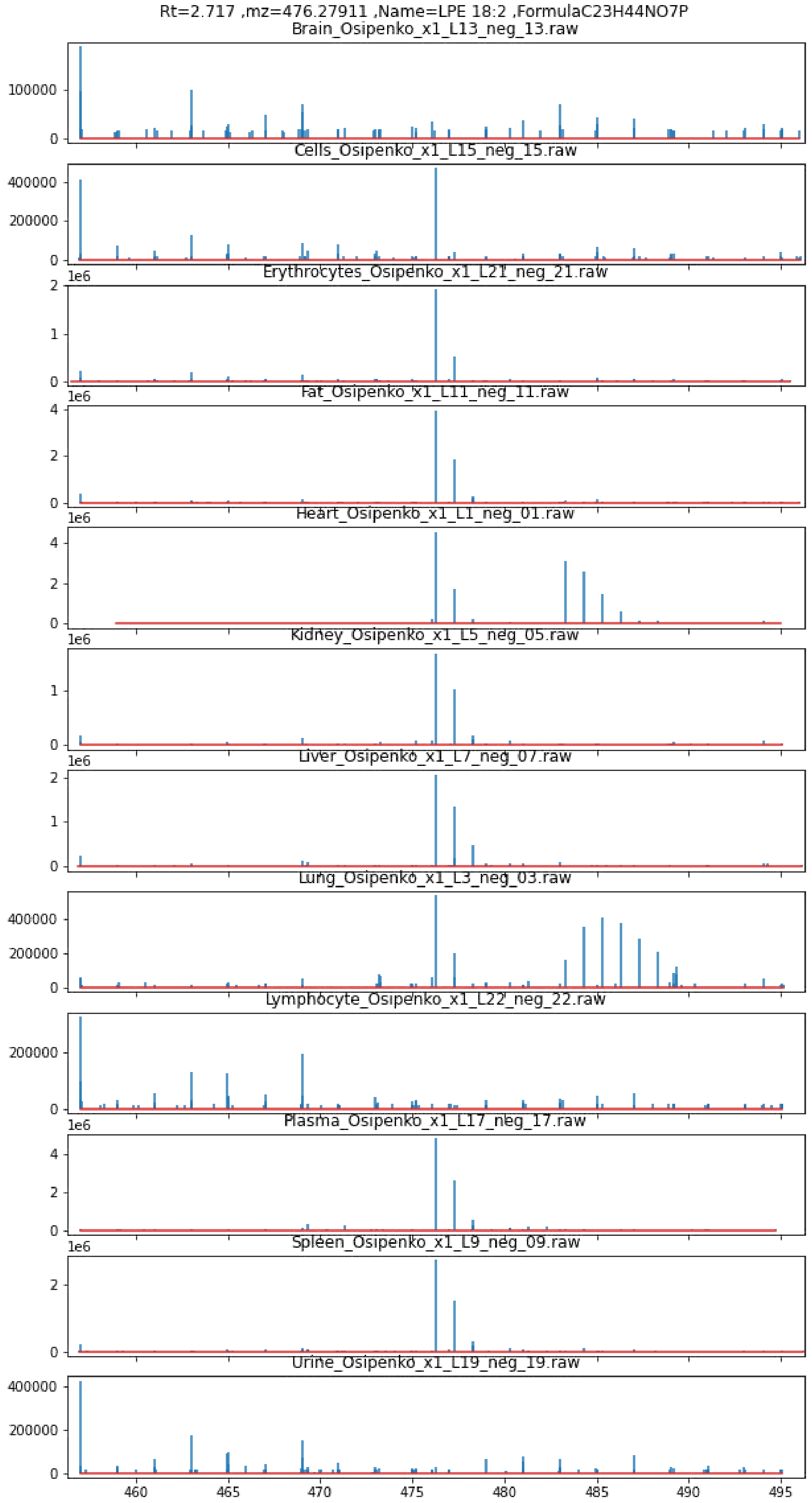

Rt=2.717 ,mz=476.27911 ,Name=LPE 18:2 ,FormulaC23H44NO7P
Brain_Osipenko_x1_L13_neg_13.raw
Cells_Osipenko_x1_L15_neg_15.raw
Erythrocytes_Osipenko_x1_L21_neg_21.raw
Fat_Osipenko_x1_L11_neg_11.raw
Heart_Osipenko_x1_L1_neg_01.raw
Kidney_Osipenko_x1_L5_neg_05.raw
Liver_Osipenko_x1_L7_neg_07.raw
Lung_Osipenko_x1_L3_neg_03.raw
Lymphocyte_Osipenko_x1_L22_neg_22.raw
Plasma_Osipenko_x1_L17_neg_17.raw
Spleen_Osipenko_x1_L9_neg_09.raw
Urine_Osipenko_x1_L19_neg_19.raw
1e6
460
465
470
475
480
485
490
495

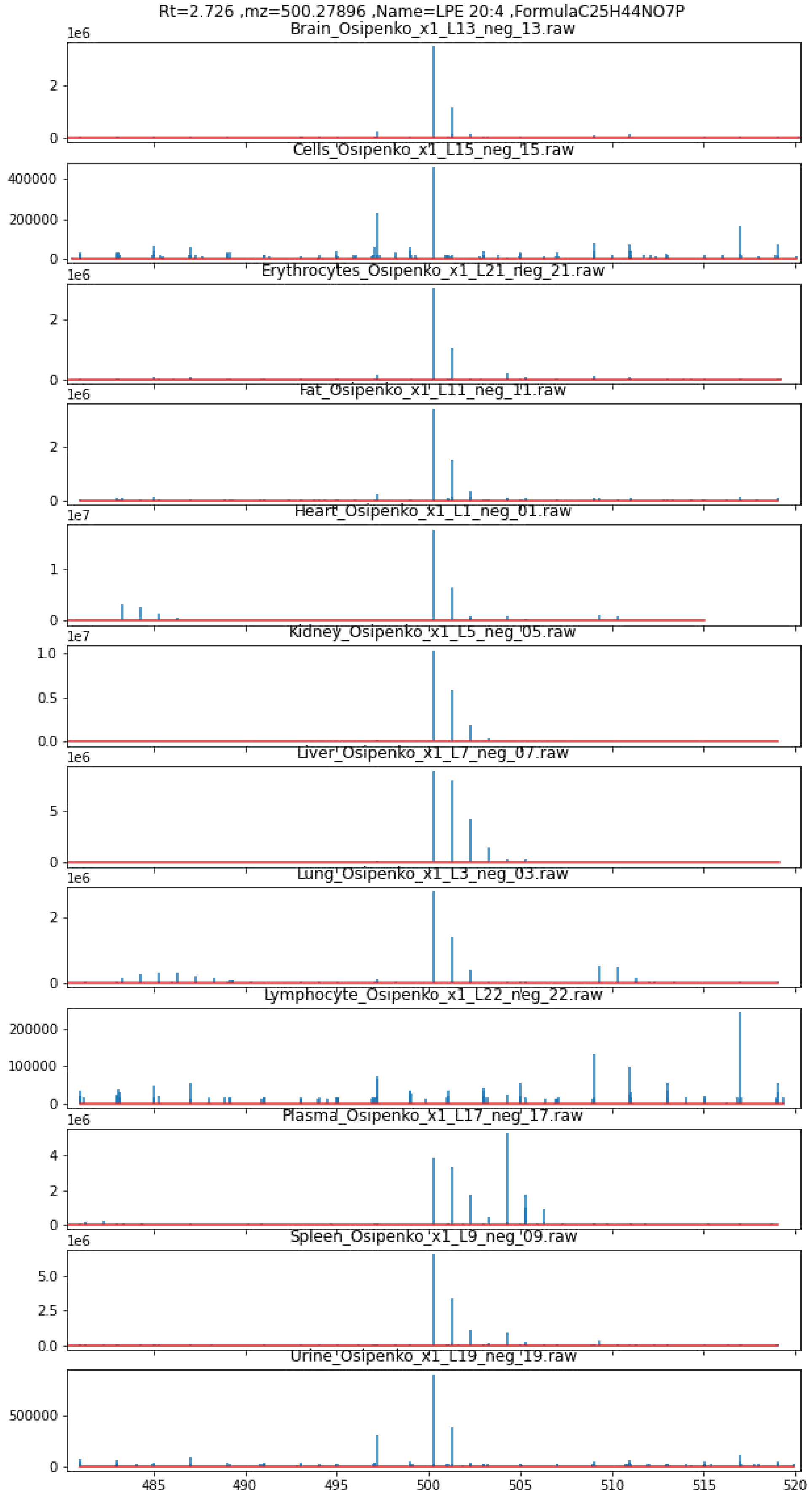

Rt=2.726 ,mz=500.27896 ,Name=LPE 20:4 ,FormulaC25H44NO7P
Brain_Osipenko_x1_L13_neg_13.raw
Cells_Osipenko_x1_L15_neg_15.raw
Erythrocytes_Osipenko_x1_L21_neg_21.raw
Fat_Osipenko_x1_L11_neg_11.raw
Heart_Osipenko_x1_L1_neg_01.raw
Kidney_Osipenko_x1_L5_neg_05.raw
Liver_Osipenko_x1_L7_neg_07.raw
Lung_Osipenko_x1_L3_neg_03.raw
Lymphocyte_Osipenko_x1_L22_neg_22.raw
Plasma_Osipenko_x1_L17_neg_17.raw
Spleen_Osipenko_x1_L9_neg_09.raw
Urine_Osipenko_x1_L19_neg_19.raw
485
490
495
500
505
510
515
520

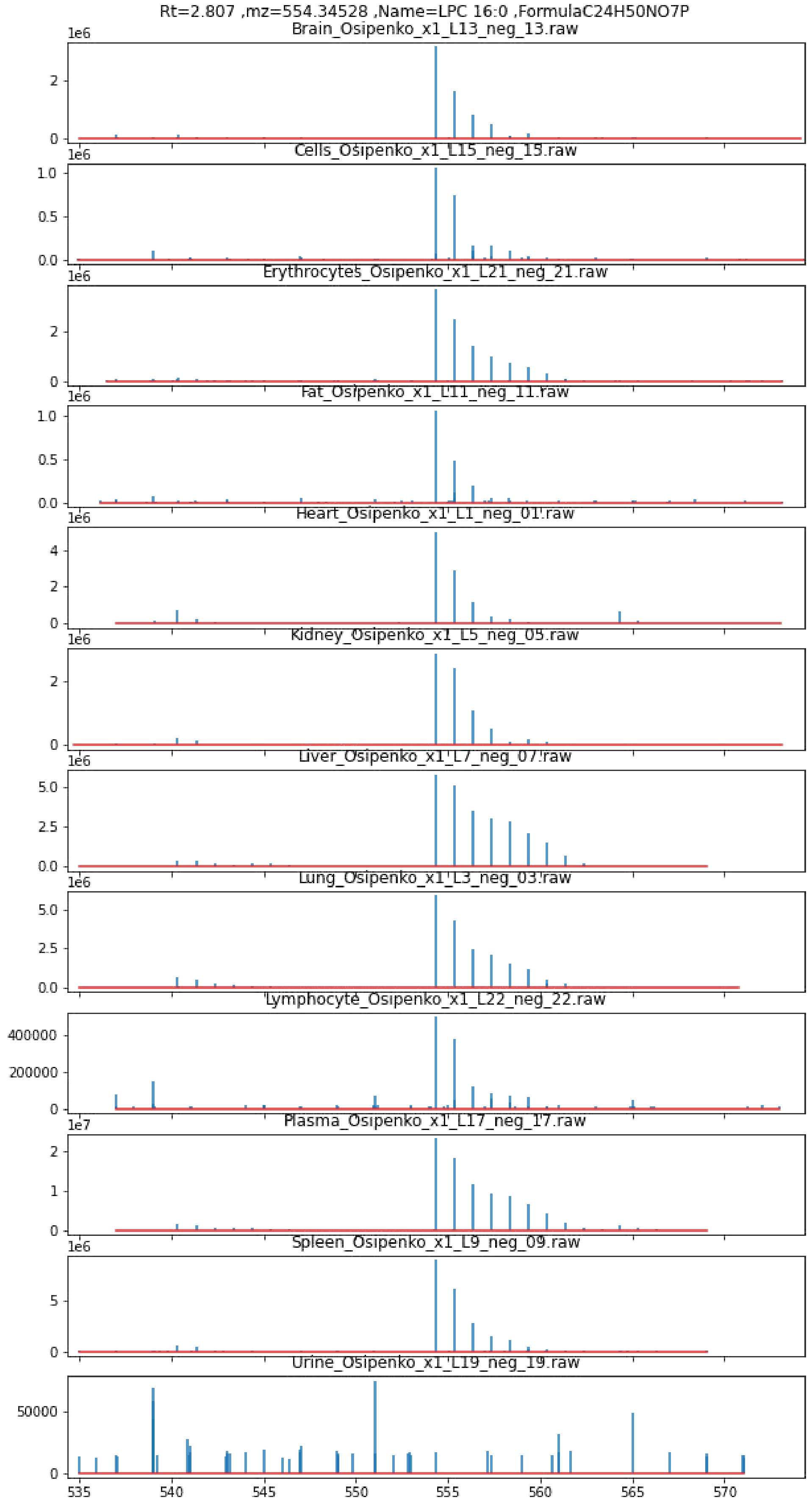

Rt=2.807 ,mz=554.34528 ,Name=LPC 16:0 ,FormulaC24H50NO7P
Brain_Osipenko_x1_L13_neg_13.raw
Cells_Osipenko_x1_L15_neg_15.raw
Erythrocytes_Osipenko_x1_L21_neg_21.raw
Fat_Osipenko_x1_L11_neg_11.raw
Heart_Osipenko_x1_L1_neg_01.raw
Kidney_Osipenko_x1_L5_neg_05.raw
Liver_Osipenko_x1_L7_neg_07.raw
Lung_Osipenko_x1_L3_neg_03.raw
Lymphocyte_Osipenko_x1_L22_neg_22.raw
Plasma_Osipenko_x1_L17_neg_17.raw
Spleen_Osipenko_x1_L9_neg_09.raw
Urine_Osipenko_x1_L19_neg_19.raw

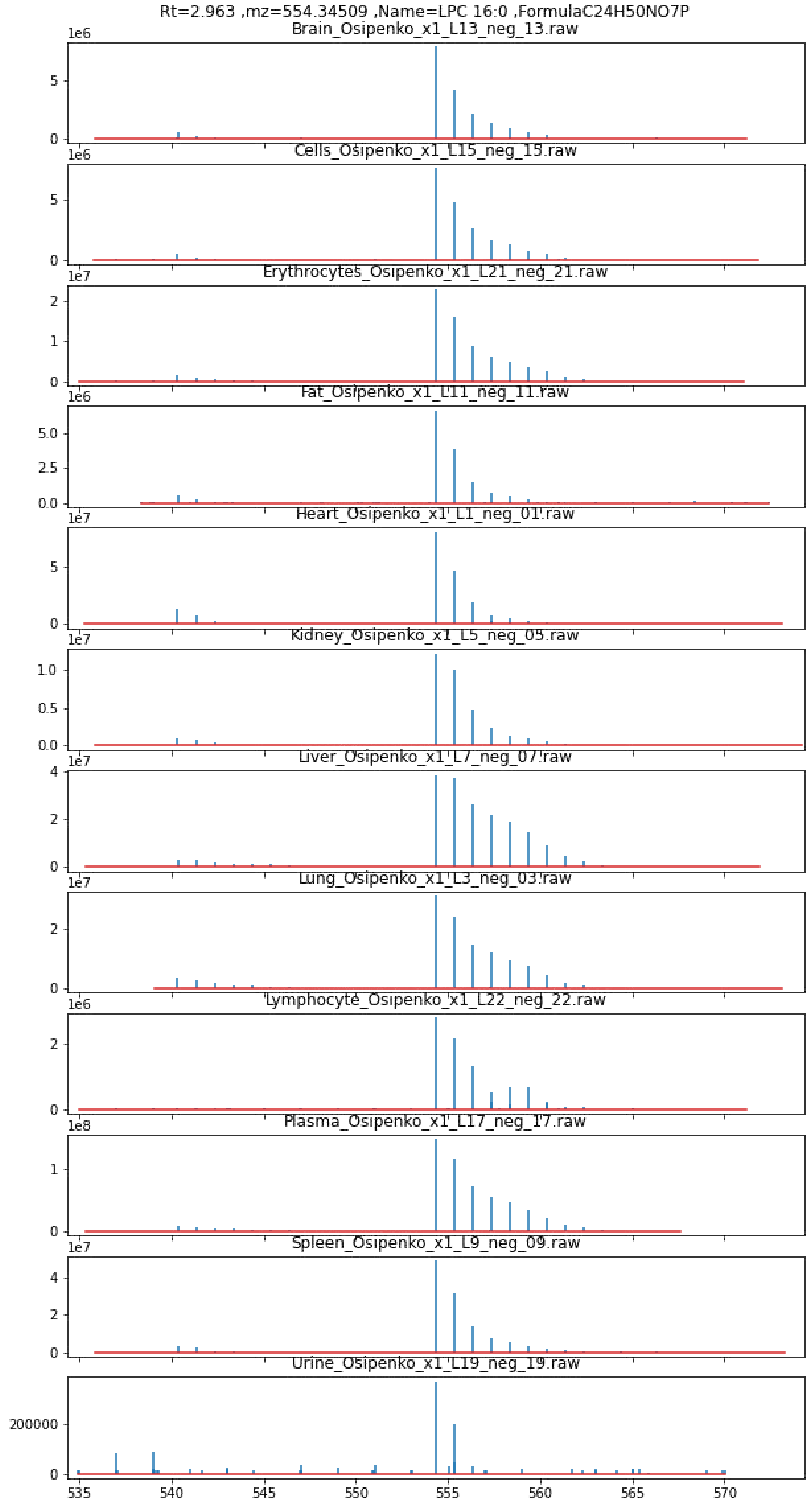

Rt=2.963 ,mz=554.34509 ,Name=LPC 16:0 ,FormulaC24H50NO7P
Brain_Osipenko_x1_L13_neg_13.raw
Cells_Osipenko_x1_L15_neg_15.raw
Erythrocytes_Osipenko_x1_L21_neg_21.raw
Fat_Osipenko_x1_L11_neg_11.raw
Heart_Osipenko_x1_L1_neg_01.raw
Kidney_Osipenko_x1_L5_neg_05.raw
Liver_Osipenko_x1_L7_neg_07.raw
Lung_Osipenko_x1_L3_neg_03.raw
Lymphocyte_Osipenko_x1_L22_neg_22.raw
Plasma_Osipenko_x1_L17_neg_17.raw
Spleen_Osipenko_x1_L9_neg_09.raw
Urine_Osipenko_x1_L19_neg_19.raw
1e6
1e7
1e8
200000
535
540
545
550
555
560
565
570

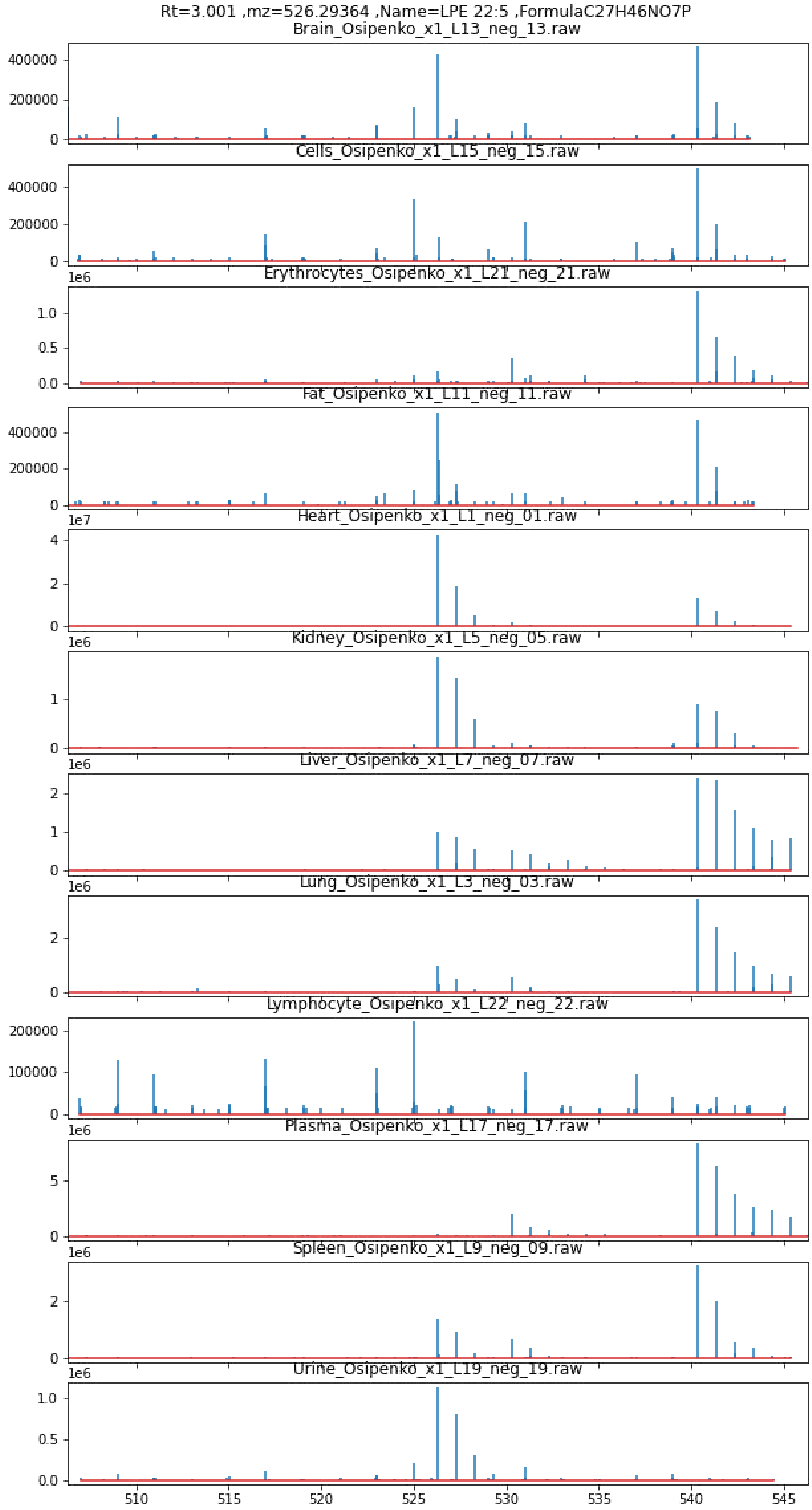

Rt=3.001 ,mz=526.29364 ,Name=LPE 22:5 ,FormulaC27H46NO7P
Brain_Osipenko_x1_L13_neg_13.raw
Cells_Osipenko_x1_L15_neg_15.raw
Erythrocytes_Osipenko_x1_L21_neg_21.raw
Fat_Osipenko_x1_L11_neg_11.raw
Heart_Osipenko_x1_L1_neg_01.raw
Kidney_Osipenko_x1_L5_neg_05.raw
Liver_Osipenko_x1_L7_neg_07.raw
Lung_Osipenko_x1_L3_neg_03.raw
Lymphocyte_Osipenko_x1_L22_neg_22.raw
Plasma_Osipenko_x1_L17_neg_17.raw
Spleen_Osipenko_x1_L9_neg_09.raw
Urine_Osipenko_x1_L19_neg_19.raw
1e6
1e7
510
515
520
525
530
535
540
545

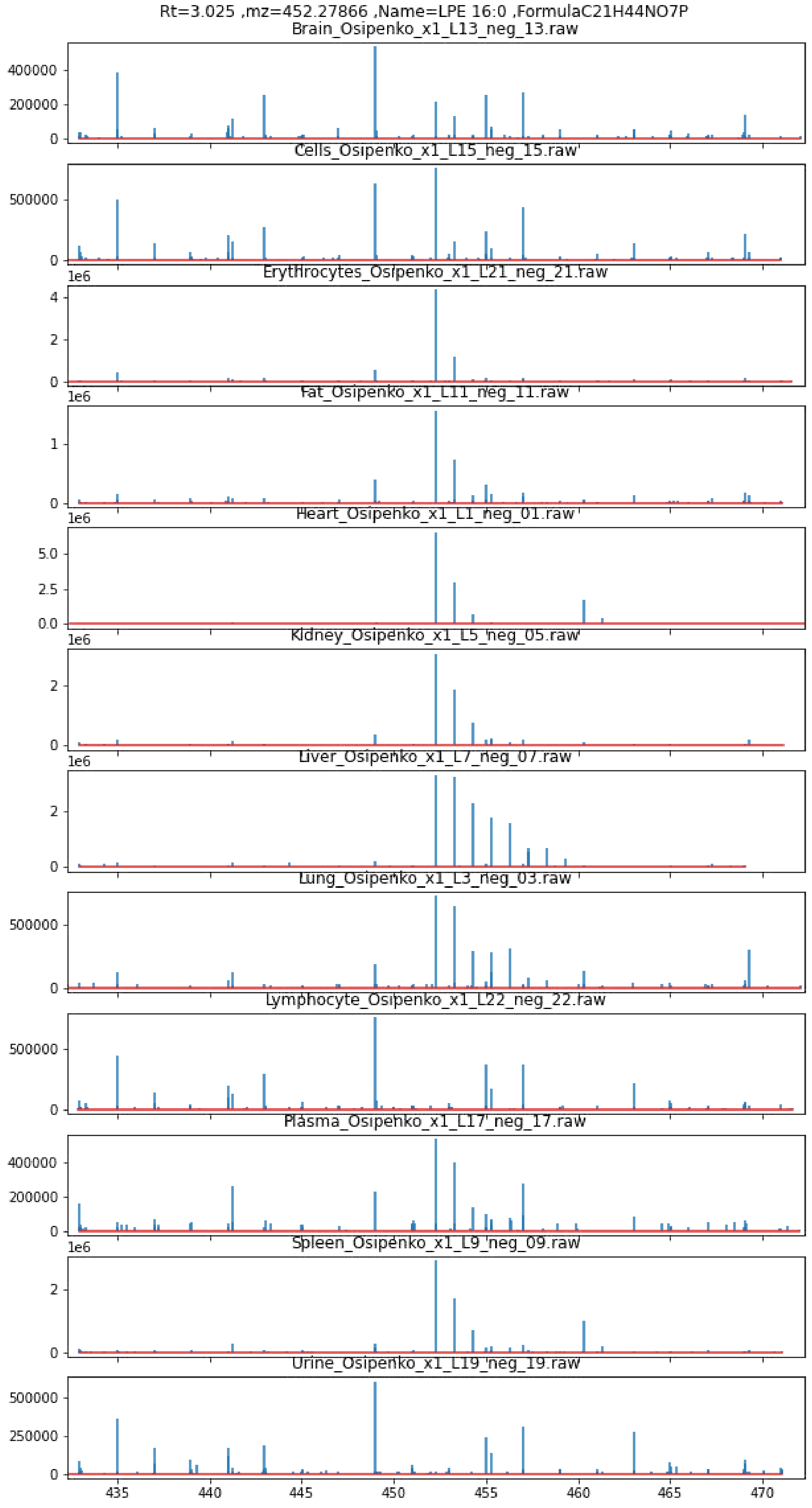

Rt=3.025 ,mz=452.27866 ,Name=LPE 16:0 ,FormulaC21H44NO7P
Brain_Osipenko_x1_L13_neg_13.raw
Cells_Osipenko_x1_L15_neg_15.raw
Erythrocytes_Osipenko_x1_L21_neg_21.raw
Fat_Osipenko_x1_L11_neg_11.raw
Heart_Osipenko_x1_L1_neg_01.raw
Kidney_Osipenko_x1_L5_neg_05.raw
Liver_Osipenko_x1_L7_neg_07.raw
Lung_Osipenko_x1_L3_neg_03.raw
Lymphocyte_Osipenko_x1_L22_neg_22.raw
Plasma_Osipenko_x1_L17_neg_17.raw
Spleen_Osipenko_x1_L9_neg_09.raw
Urine_Osipenko_x1_L19_neg_19.raw
1e6
435
440
445
450
455
460
465
470

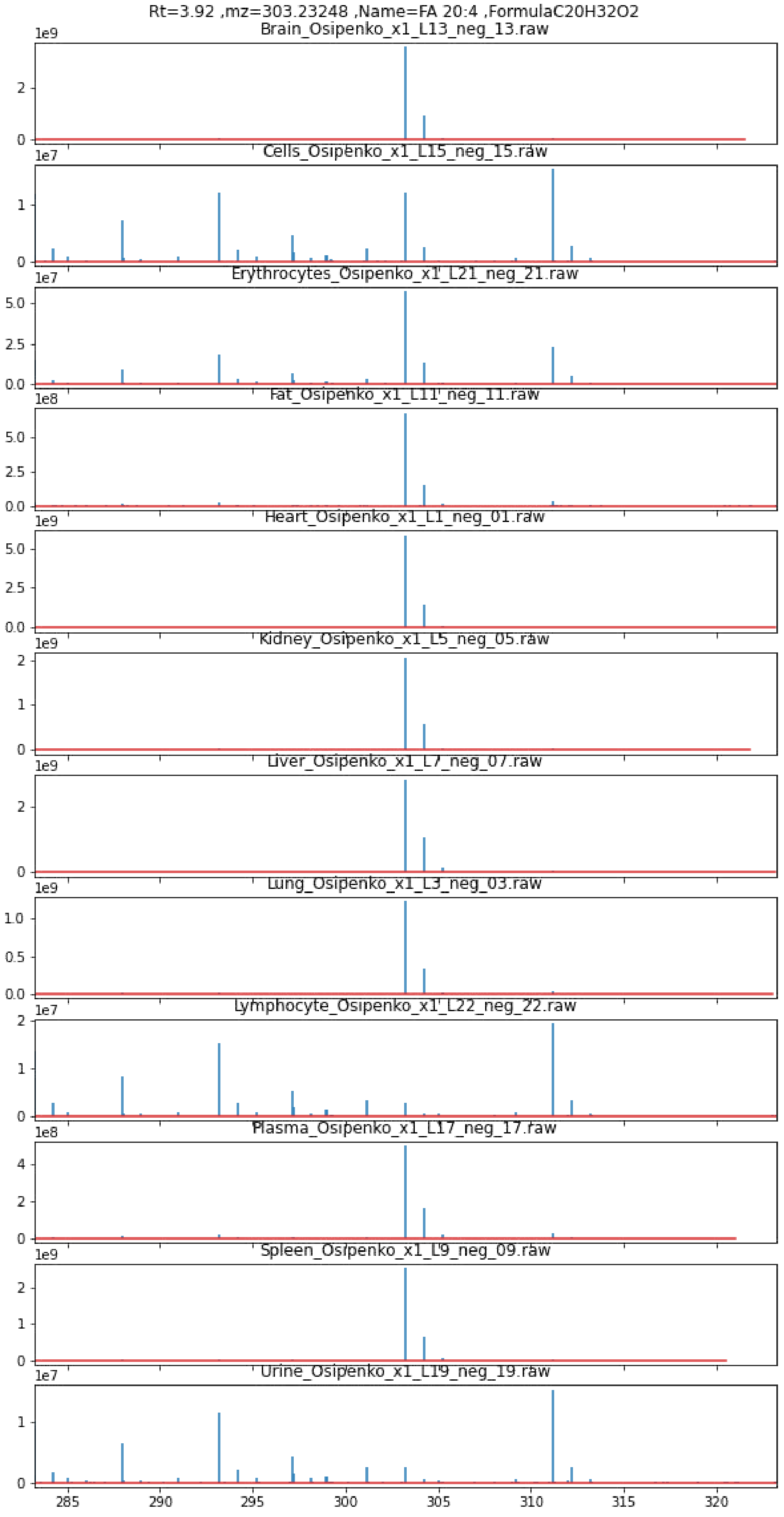

Rt=3.92 ,mz=303.23248 ,Name=FA 20:4 ,FormulaC20H32O2
Brain_Osipenko_x1_L13_neg_13.raw
Cells_Osipenko_x1_L15_neg_15.raw
Erythrocytes_Osipenko_x1_L21_neg_21.raw
Fat_Osipenko_x1_L11_neg_11.raw
Heart_Osipenko_x1_L1_neg_01.raw
Kidney_Osipenko_x1_L5_neg_05.raw
Liver_Osipenko_x1_L7_neg_07.raw
Lung_Osipenko_x1_L3_neg_03.raw
Lymphocyte_Osipenko_x1_L22_neg_22.raw
Plasma_Osipenko_x1_L17_neg_17.raw
Spleen_Osipenko_x1_L9_neg_09.raw
Urine_Osipenko_x1_L19_neg_19.raw
285
290
295
300
305
310
315
320

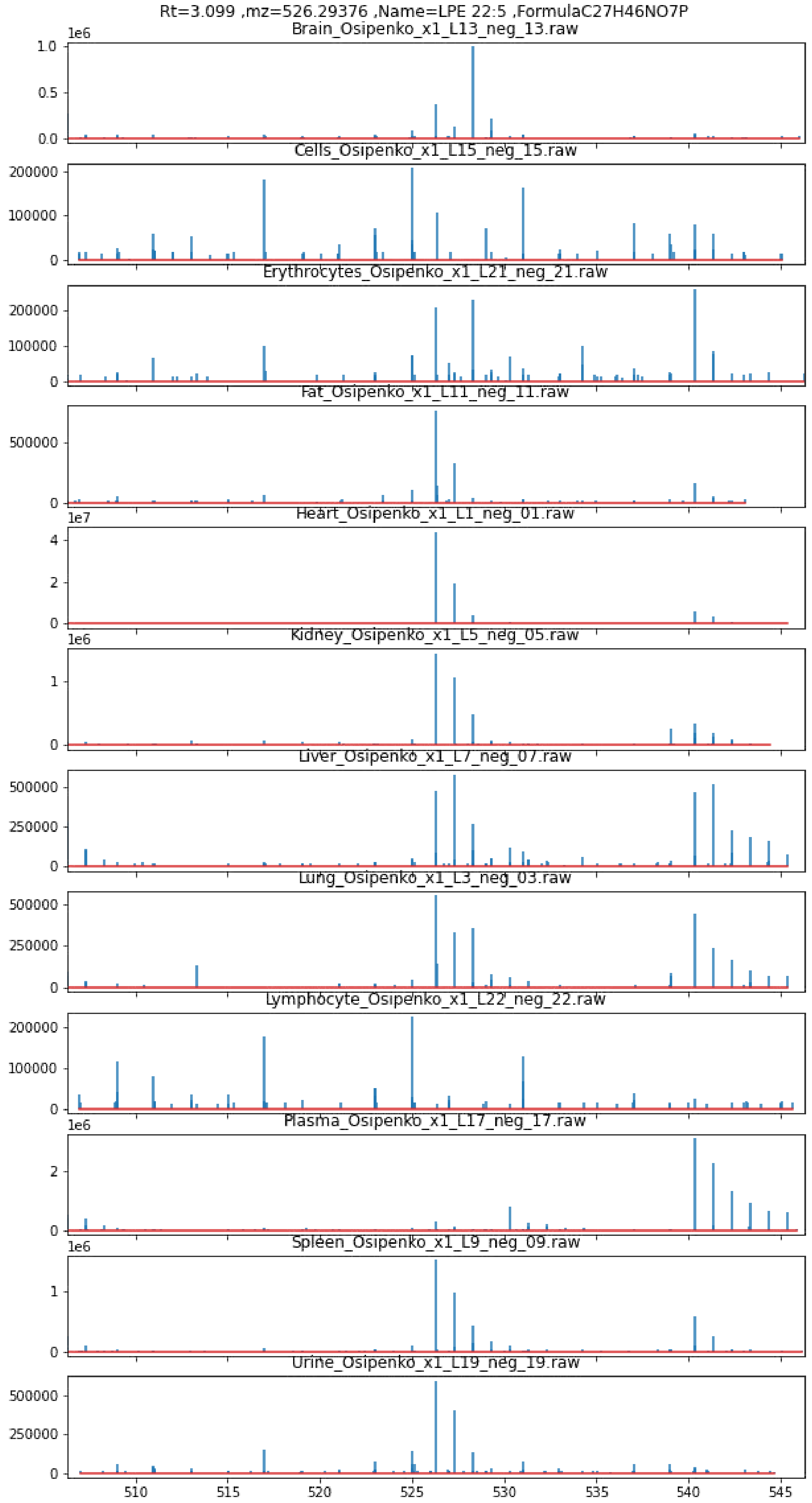

Rt=3.099 ,mz=526.29376 ,Name=LPE 22:5 ,FormulaC27H46NO7P
Brain_Osipenko_x1_L13_neg_13.raw
Cells_Osipenko_x1_L15_neg_15.raw
Erythrocytes_Osipenko_x1_L21_neg_21.raw
Fat_Osipenko_x1_L11_neg_11.raw
Heart_Osipenko_x1_L1_neg_01.raw
Kidney_Osipenko_x1_L5_neg_05.raw
Liver_Osipenko_x1_L7_neg_07.raw
Lung_Osipenko_x1_L3_neg_03.raw
Lymphocyte_Osipenko_x1_L22_neg_22.raw
Plasma_Osipenko_x1_L17_neg_17.raw
Spleen_Osipenko_x1_L9_neg_09.raw
Urine_Osipenko_x1_L19_neg_19.raw
1e6
1e7
510
515
520
525
530
535
540
545

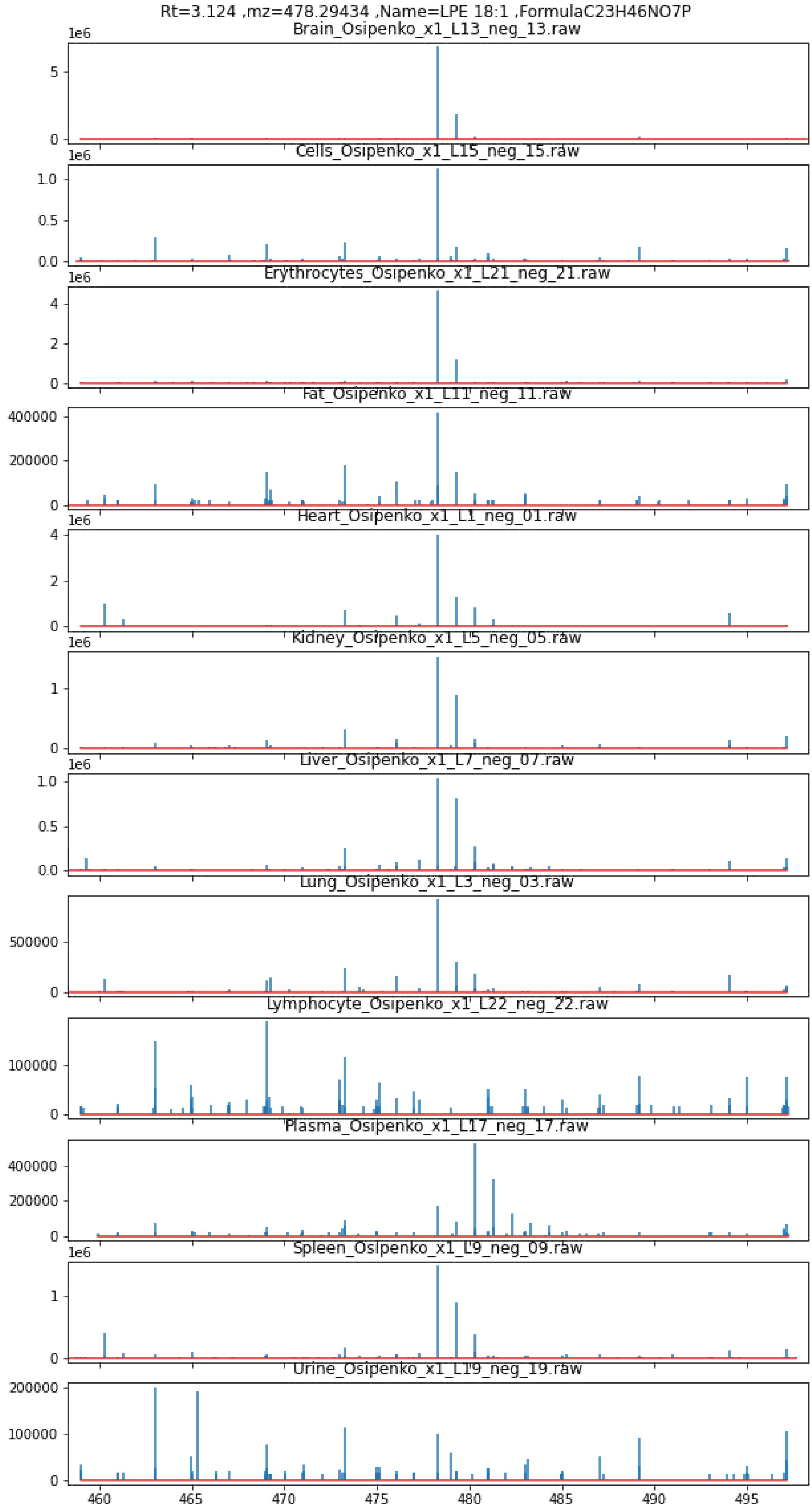
Rt=3.124 ,mz=478.29434 ,Name=LPE 18:1 ,FormulaC23H46NO7P
Brain_Osipenko_x1_L13_neg_13.raw
Cells_Osipenko_x1_L15_neg_15.raw
Erythrocytes_Osipenko_x1_L21_neg_21.raw
Fat_Osipenko_x1_L11_neg_11.raw
Heart_Osipenko_x1_L1_neg_01.raw
Kidney_Osipenko_x1_L5_neg_05.raw
Liver_Osipenko_x1_L7_neg_07.raw
Lung_Osipenko_x1_L3_neg_03.raw
Lymphocyte_Osipenko_x1_L22_neg_22.raw
Plasma_Osipenko_x1_L17_neg_17.raw
Spleen_Osipenko_x1_L9_neg_09.raw
Urine_Osipenko_x1_L19_neg_19.raw
1e6
460
465
470
475
480
485
490
495

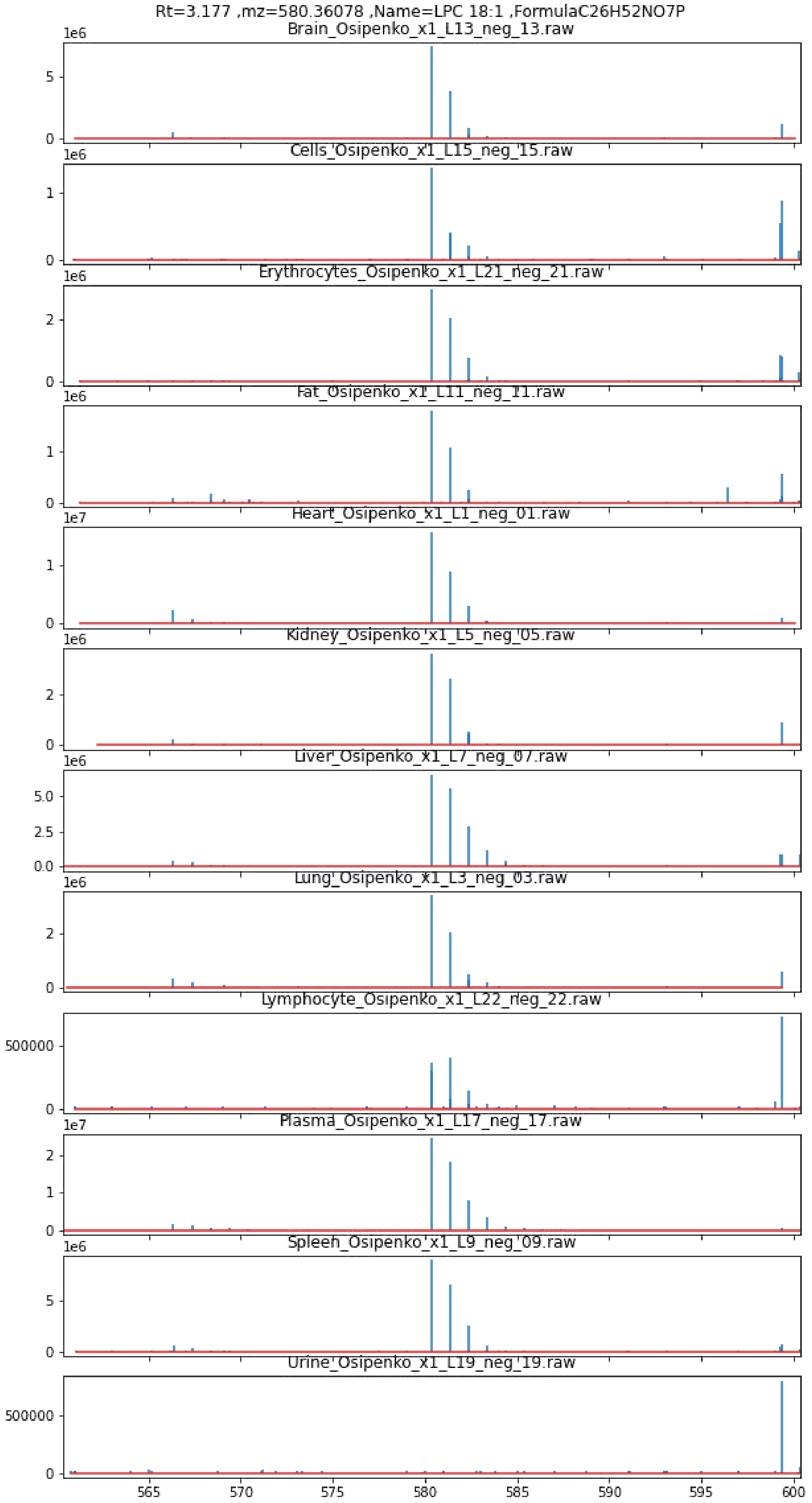
Rt=3.177 ,mz=580.36078 ,Name=LPC 18:1 ,FormulaC26H52NO7P
Brain_Osipenko_x1_L13_neg_13.raw
Cells_Osipenko_x1_L15_neg_15.raw
Erythrocytes_Osipenko_x1_L21_neg_21.raw
Fat_Osipenko_x1_L11_neg_11.raw
Heart_Osipenko_x1_L1_neg_01.raw
Kidney_Osipenko_x1_L5_neg_05.raw
Liver_Osipenko_x1_L7_neg_07.raw
Lung_Osipenko_x1_L3_neg_03.raw
Lymphocyte_Osipenko_x1_L22_neg_22.raw
Plasma_Osipenko_x1_L17_neg_17.raw
Spleen_Osipenko_x1_L9_neg_09.raw
Urine_Osipenko_x1_L19_neg_19.raw
1e6
1e7
565
570
575
580
585
590
595
600

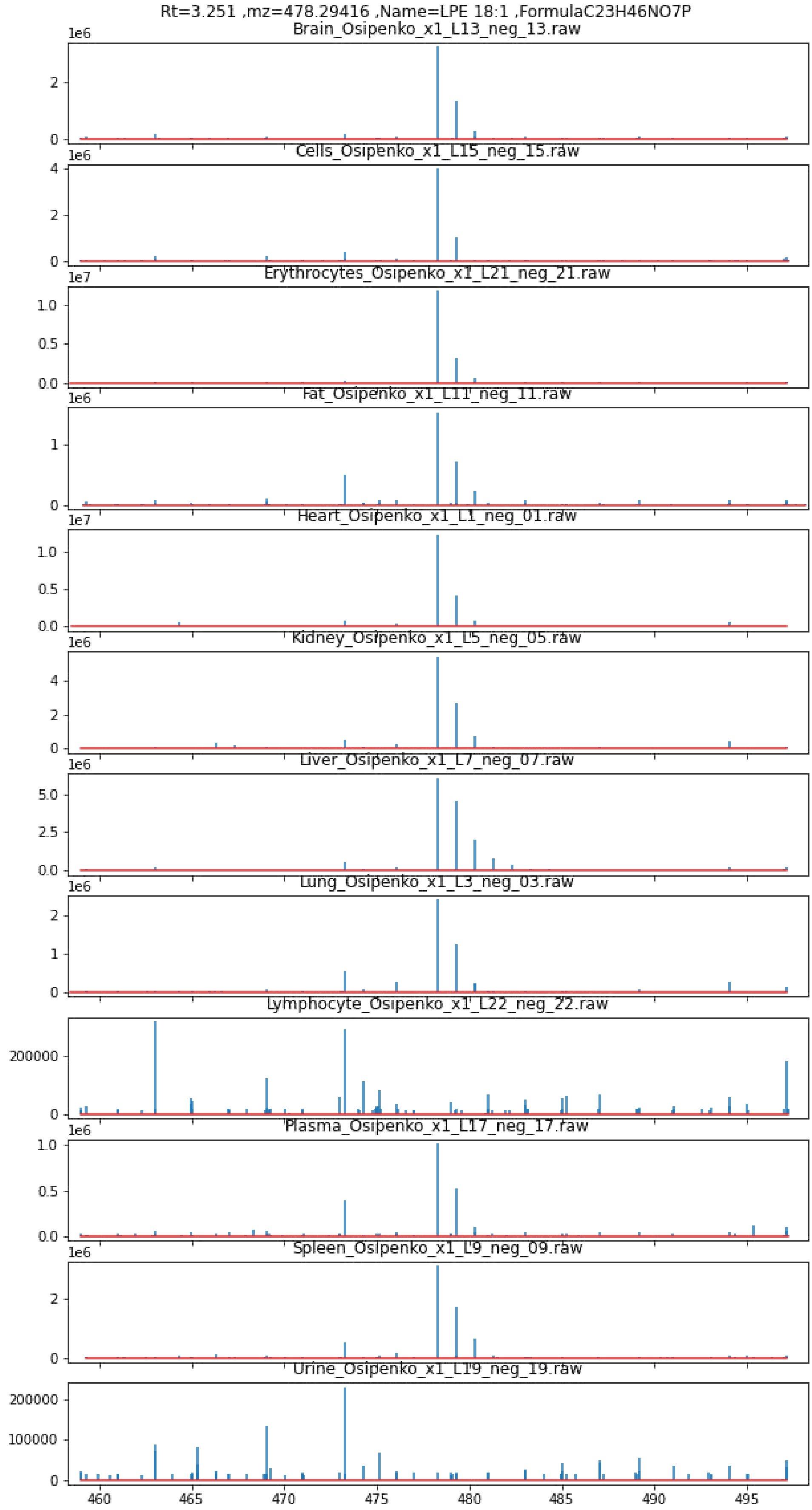

Rt=3.251 ,mz=478.29416 ,Name=LPE 18:1 ,FormulaC23H46NO7P
Brain_Osipenko_x1_L13_neg_13.raw
Cells_Osipenko_x1_L15_neg_15.raw
Erythrocytes_Osipenko_x1_L21_neg_21.raw
Fat_Osipenko_x1_L11_neg_11.raw
Heart_Osipenko_x1_L1_neg_01.raw
Kidney_Osipenko_x1_L5_neg_05.raw
Liver_Osipenko_x1_L7_neg_07.raw
Lung_Osipenko_x1_L3_neg_03.raw
Lymphocyte_Osipenko_x1_L22_neg_22.raw
Plasma_Osipenko_x1_L17_neg_17.raw
Spleen_Osipenko_x1_L9_neg_09.raw
Urine_Osipenko_x1_L19_neg_19.raw
460
465
470
475
480
485
490
495

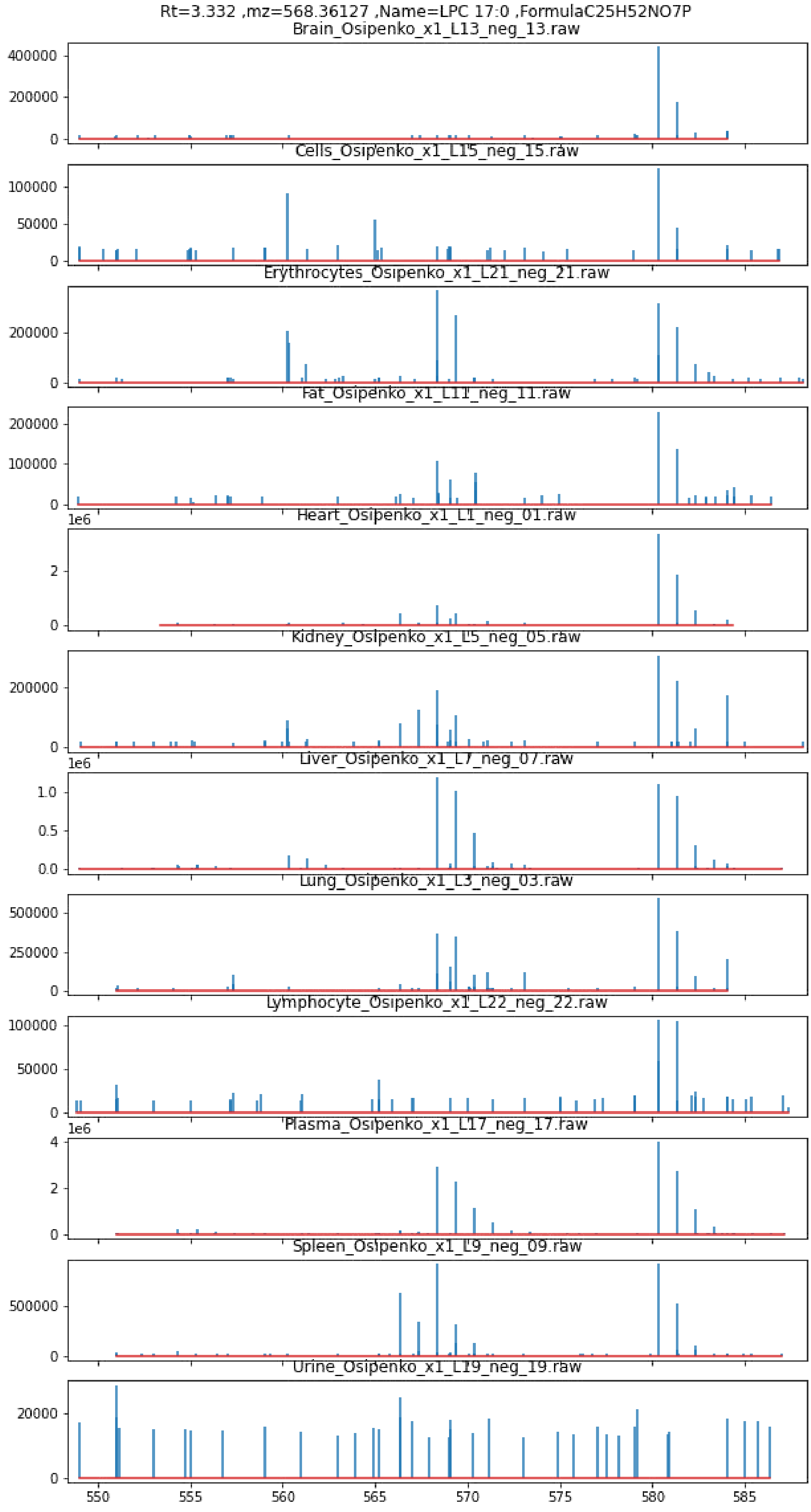
Rt=3.332 ,mz=568.36127 ,Name=LPC 17:0 ,FormulaC25H52NO7P
Brain_Osipenko_x1_L13_neg_13.raw
Cells_Osipenko_x1_L15_neg_15.raw
Erythrocytes_Osipenko_x1_L21_neg_21.raw
Fat_Osipenko_x1_L11_neg_11.raw
Heart_Osipenko_x1_L1_neg_01.raw
Kidney_Osipenko_x1_L5_neg_05.raw
Liver_Osipenko_x1_L7_neg_07.raw
Lung_Osipenko_x1_L3_neg_03.raw
Lymphocyte_Osipenko_x1_L22_neg_22.raw
Plasma_Osipenko_x1_L17_neg_17.raw
Spleen_Osipenko_x1_L9_neg_09.raw
Urine_Osipenko_x1_L19_neg_19.raw
550
555
560
565
570
575
580
585

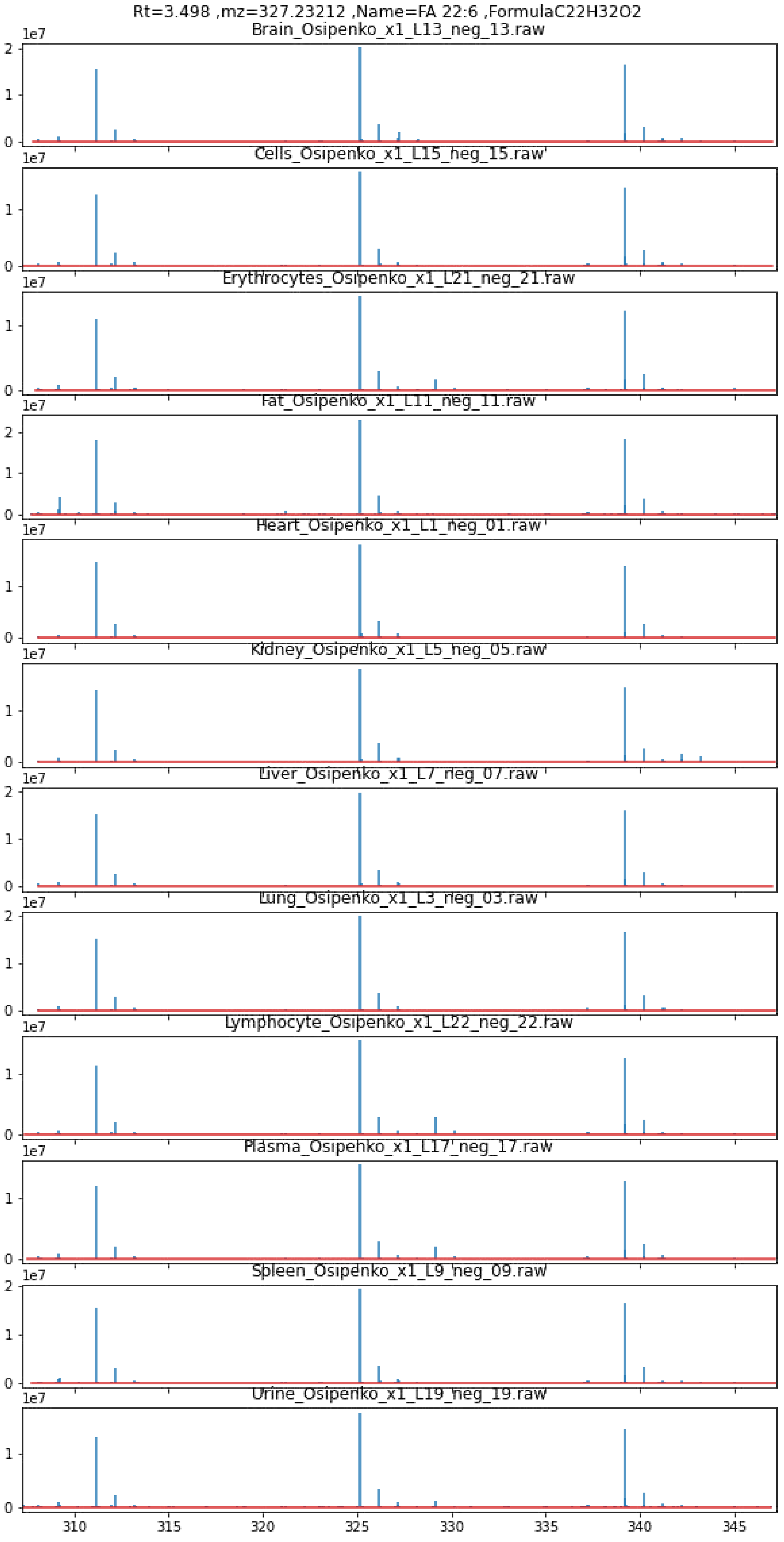
Rt=3.498 ,mz=327.23212 ,Name=FA 22:6 ,FormulaC22H32O2
Brain_Osipenko_x1_L13_neg_13.raw
Cells_Osipenko_x1_L15_neg_15.raw
Erythrocytes_Osipenko_x1_L21_neg_21.raw
Fat_Osipenko_x1_L11_neg_11.raw
Heart_Osipenko_x1_L1_neg_01.raw
Kidney_Osipenko_x1_L5_neg_05.raw
Liver_Osipenko_x1_L7_neg_07.raw
Lung_Osipenko_x1_L3_neg_03.raw
Lymphocyte_Osipenko_x1_L22_neg_22.raw
Plasma_Osipenko_x1_L17_neg_17.raw
Spleen_Osipenko_x1_L9_neg_09.raw
Urine_Osipenko_x1_L19_neg_19.raw
1e7
310
315
320
325
330
335
340
345

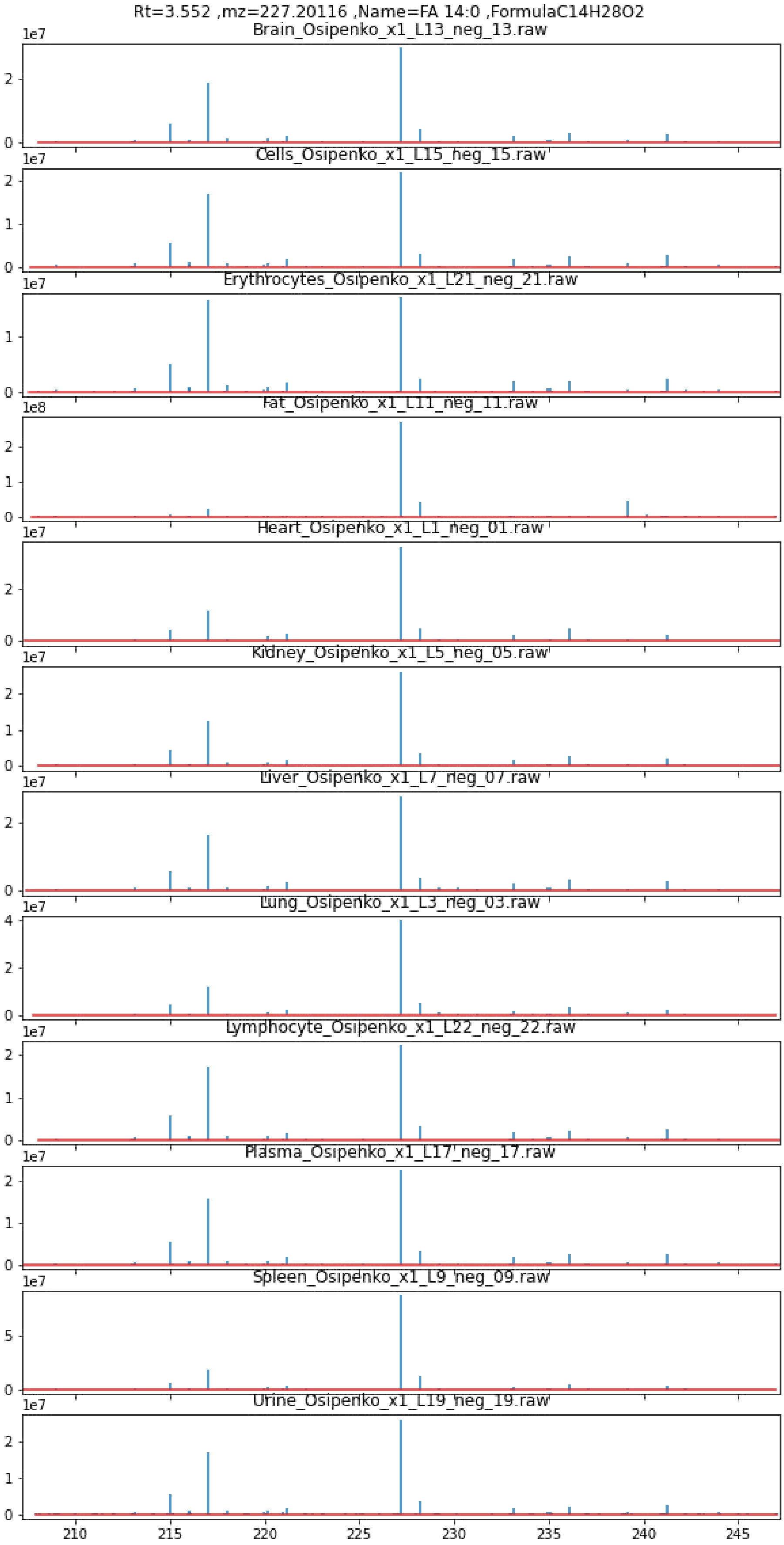

Rt=3.552 ,mz=227.20116 ,Name=FA 14:0 ,FormulaC14H28O2
Brain_Osipenko_x1_L13_neg_13.raw
Cells_Osipenko_x1_L15_neg_15.raw
Erythrocytes_Osipenko_x1_L21_neg_21.raw
Fat_Osipenko_x1_L11_neg_11.raw
Heart_Osipenko_x1_L1_neg_01.raw
Kidney_Osipenko_x1_L5_neg_05.raw
Liver_Osipenko_x1_L7_neg_07.raw
Lung_Osipenko_x1_L3_neg_03.raw
Lymphocyte_Osipenko_x1_L22_neg_22.raw
Plasma_Osipenko_x1_L17_neg_17.raw
Spleen_Osipenko_x1_L9_neg_09.raw
Urine_Osipenko_x1_L19_neg_19.raw
1e7
1e8
210
215
220
225
230
235
240
245

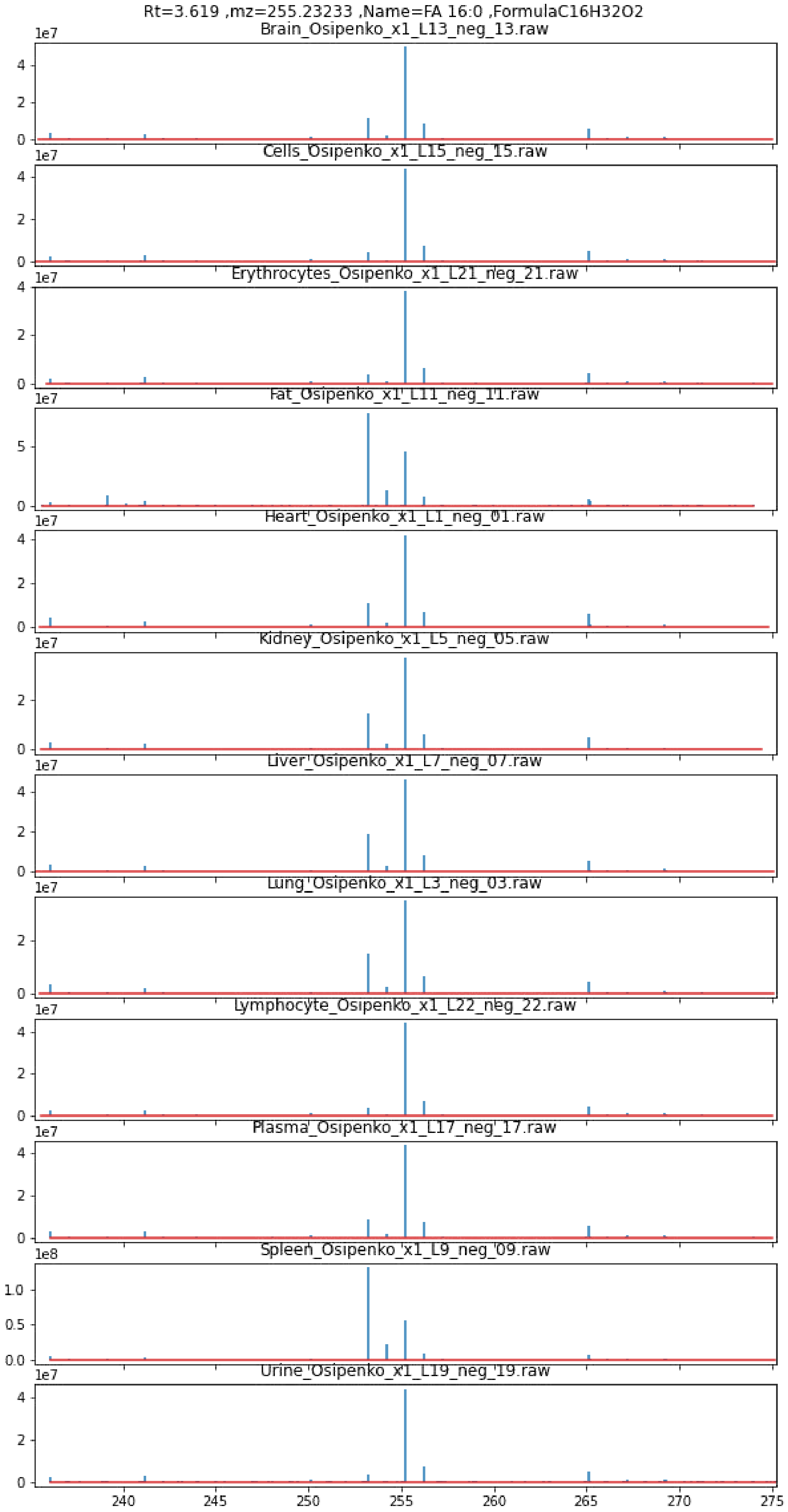

Rt=3.619 ,mz=255.23233 ,Name=FA 16:0 ,FormulaC16H32O2
Brain_Osipenko_x1_L13_neg_13.raw
Cells_Osipenko_x1_L15_neg_15.raw
Erythrocytes_Osipenko_x1_L21_neg_21.raw
Fat_Osipenko_x1_L11_neg_11.raw
Heart_Osipenko_x1_L1_neg_01.raw
Kidney_Osipenko_x1_L5_neg_05.raw
Liver_Osipenko_x1_L7_neg_07.raw
Lung_Osipenko_x1_L3_neg_03.raw
Lymphocyte_Osipenko_x1_L22_neg_22.raw
Plasma_Osipenko_x1_L17_neg_17.raw
Spleen_Osipenko_x1_L9_neg_09.raw
Urine_Osipenko_x1_L19_neg_19.raw
1e7
1e8
240
245
250
255
260
265
270
275

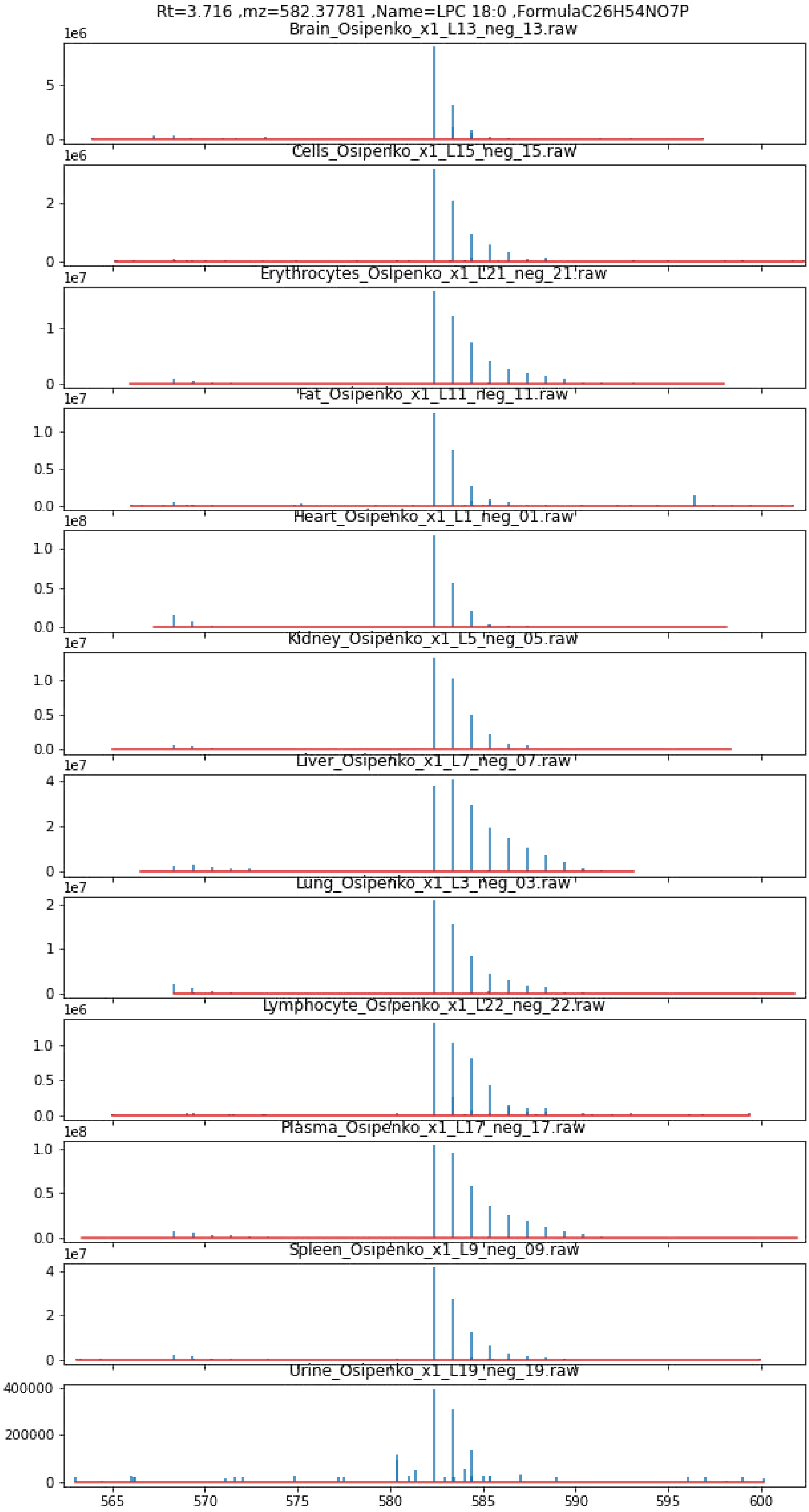

Rt=3.716 ,mz=582.37781 ,Name=LPC 18:0 ,FormulaC26H54NO7P
Brain_Osipenko_x1_L13_neg_13.raw
Cells_Osipenko_x1_L15_neg_15.raw
Erythrocytes_Osipenko_x1_L21_neg_21.raw
Fat_Osipenko_x1_L11_neg_11.raw
Heart_Osipenko_x1_L1_neg_01.raw
Kidney_Osipenko_x1_L5_neg_05.raw
Liver_Osipenko_x1_L7_neg_07.raw
Lung_Osipenko_x1_L3_neg_03.raw
Lymphocyte_Osipenko_x1_L22_neg_22.raw
Plasma_Osipenko_x1_L17_neg_17.raw
Spleen_Osipenko_x1_L9_neg_09.raw
Urine_Osipenko_x1_L19_neg_19.raw
565
570
575
580
585
590
595
600

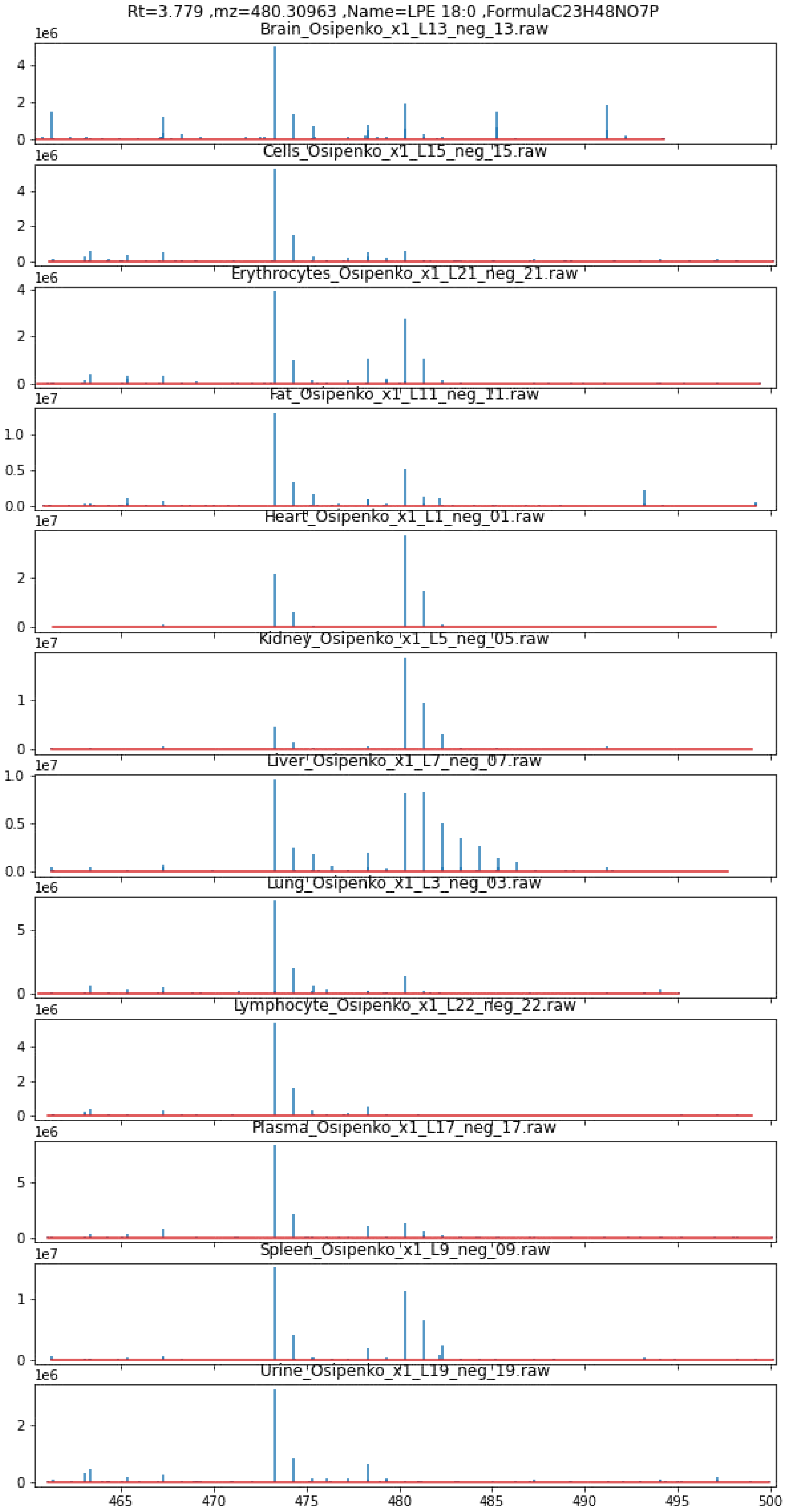
Rt=3.779 ,mz=480.30963 ,Name=LPE 18:0 ,FormulaC23H48NO7P
Brain_Osipenko_x1_L13_neg_13.raw
Cells_Osipenko_x1_L15_neg_15.raw
Erythrocytes_Osipenko_x1_L21_neg_21.raw
Fat_Osipenko_x1_L11_neg_11.raw
Heart_Osipenko_x1_L1_neg_01.raw
Kidney_Osipenko_x1_L5_neg_05.raw
Liver_Osipenko_x1_L7_neg_07.raw
Lung_Osipenko_x1_L3_neg_03.raw
Lymphocyte_Osipenko_x1_L22_neg_22.raw
Plasma_Osipenko_x1_L17_neg_17.raw
Spleen_Osipenko_x1_L9_neg_09.raw
Urine_Osipenko_x1_L19_neg_19.raw
465
470
475
480
485
490
495
500

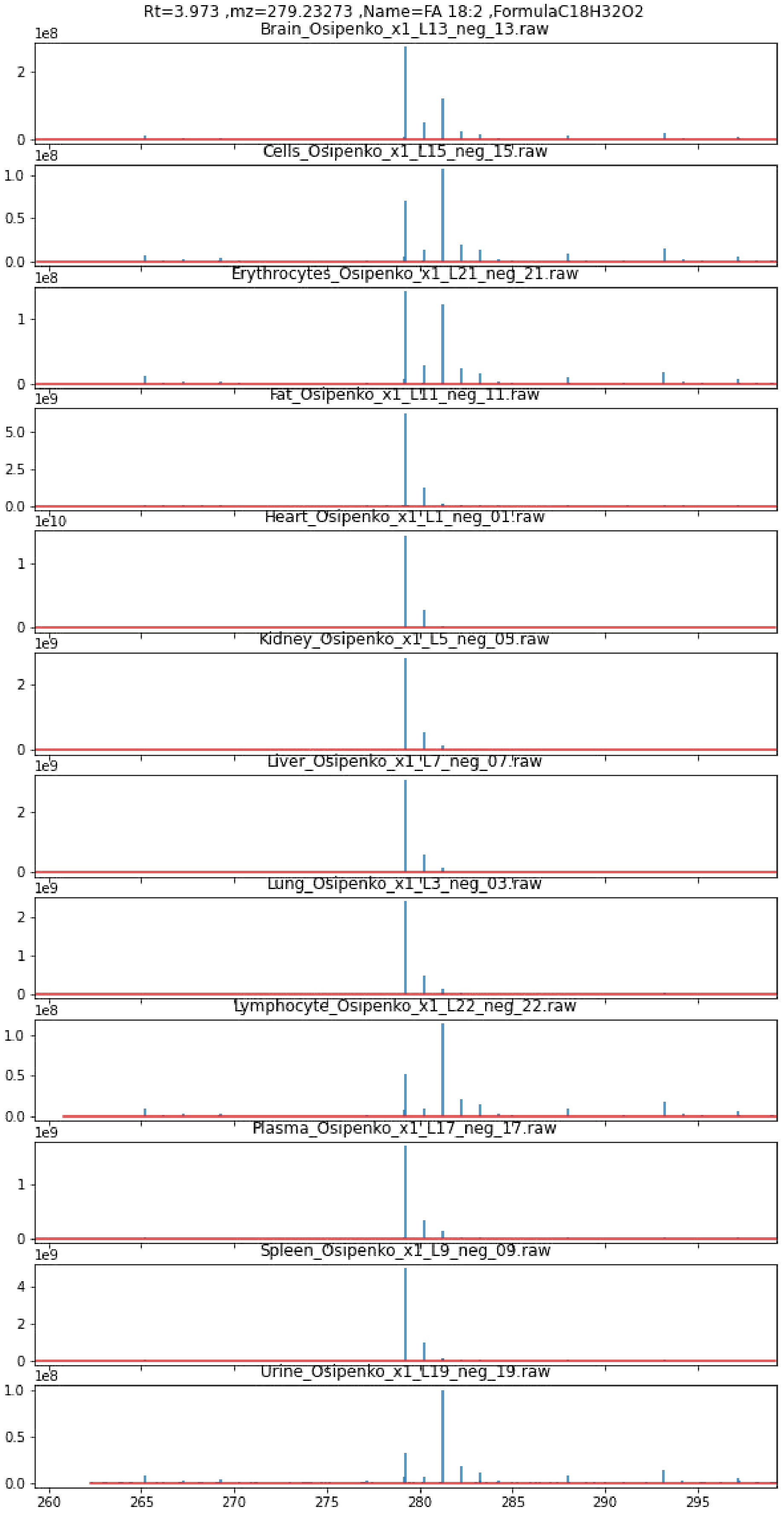

Rt=3.973 ,mz=279.23273 ,Name=FA 18:2 ,FormulaC18H32O2
Brain_Osipenko_x1_L13_neg_13.raw
Cells_Osipenko_x1_L15_neg_15.raw
Erythrocytes_Osipenko_x1_L21_neg_21.raw
Fat_Osipenko_x1_L11_neg_11.raw
Heart_Osipenko_x1_L1_neg_01.raw
Kidney_Osipenko_x1_L5_neg_05.raw
Liver_Osipenko_x1_L7_neg_07.raw
Lung_Osipenko_x1_L3_neg_03.raw
Lymphocyte_Osipenko_x1_L22_neg_22.raw
Plasma_Osipenko_x1_L17_neg_17.raw
Spleen_Osipenko_x1_L9_neg_09.raw
Urine_Osipenko_x1_L19_neg_19.raw
1e8
1e9
1e10
260
265
270
275
280
285
290
295

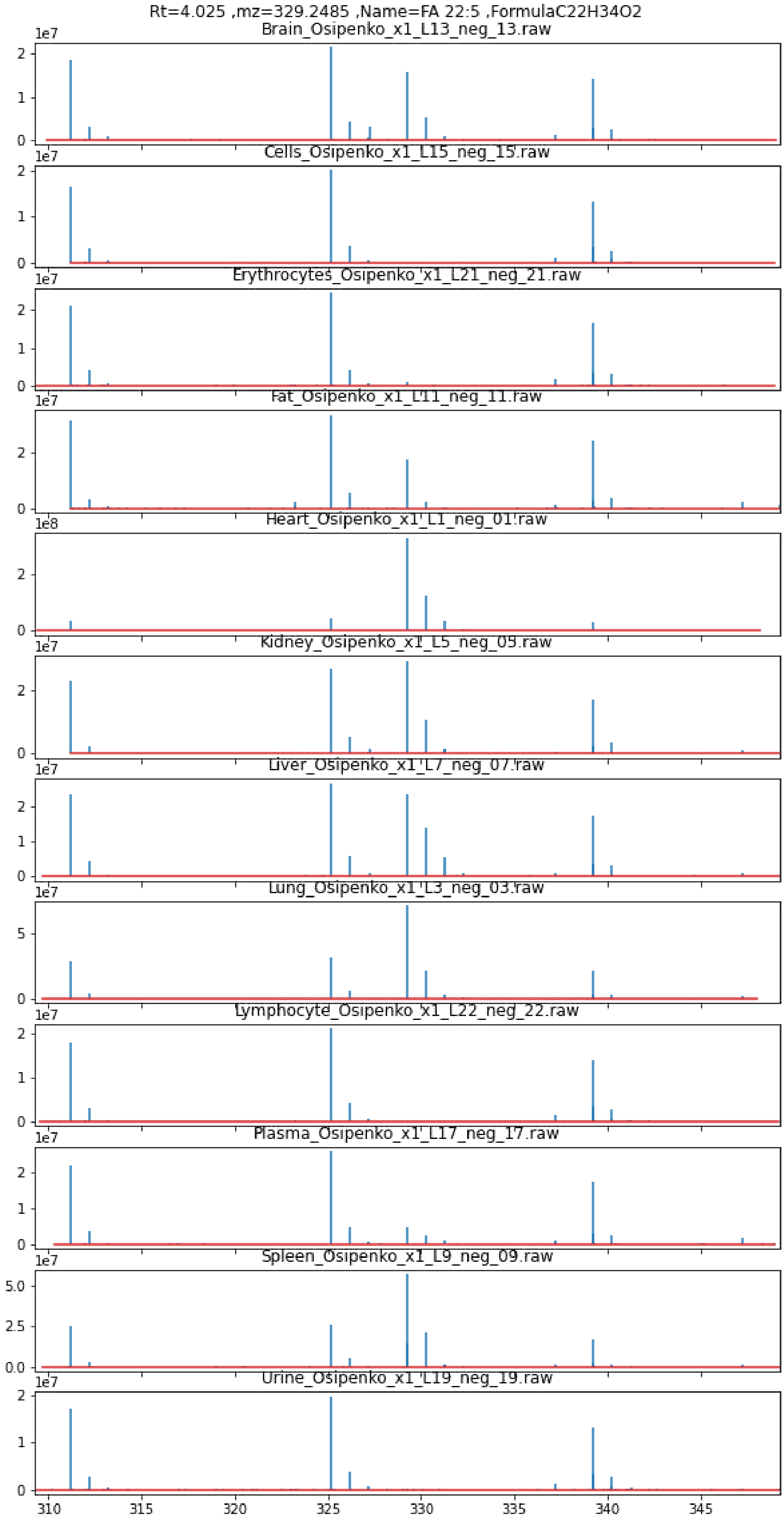
Rt=4.025 ,mz=329.2485 ,Name=FA 22:5 ,FormulaC22H34O2
Brain_Osipenko_x1_L13_neg_13.raw
Cells_Osipenko_x1_L15_neg_15.raw
Erythrocytes_Osipenko_x1_L21_neg_21.raw
Fat_Osipenko_x1_L11_neg_11.raw
Heart_Osipenko_x1_L1_neg_01.raw
Kidney_Osipenko_x1_L5_neg_05.raw
Liver_Osipenko_x1_L7_neg_07.raw
Lung_Osipenko_x1_L3_neg_03.raw
Lymphocyte_Osipenko_x1_L22_neg_22.raw
Plasma_Osipenko_x1_L17_neg_17.raw
Spleen_Osipenko_x1_L9_neg_09.raw
Urine_Osipenko_x1_L19_neg_19.raw
1e7
1e8
310
315
320
325
330
335
340
345

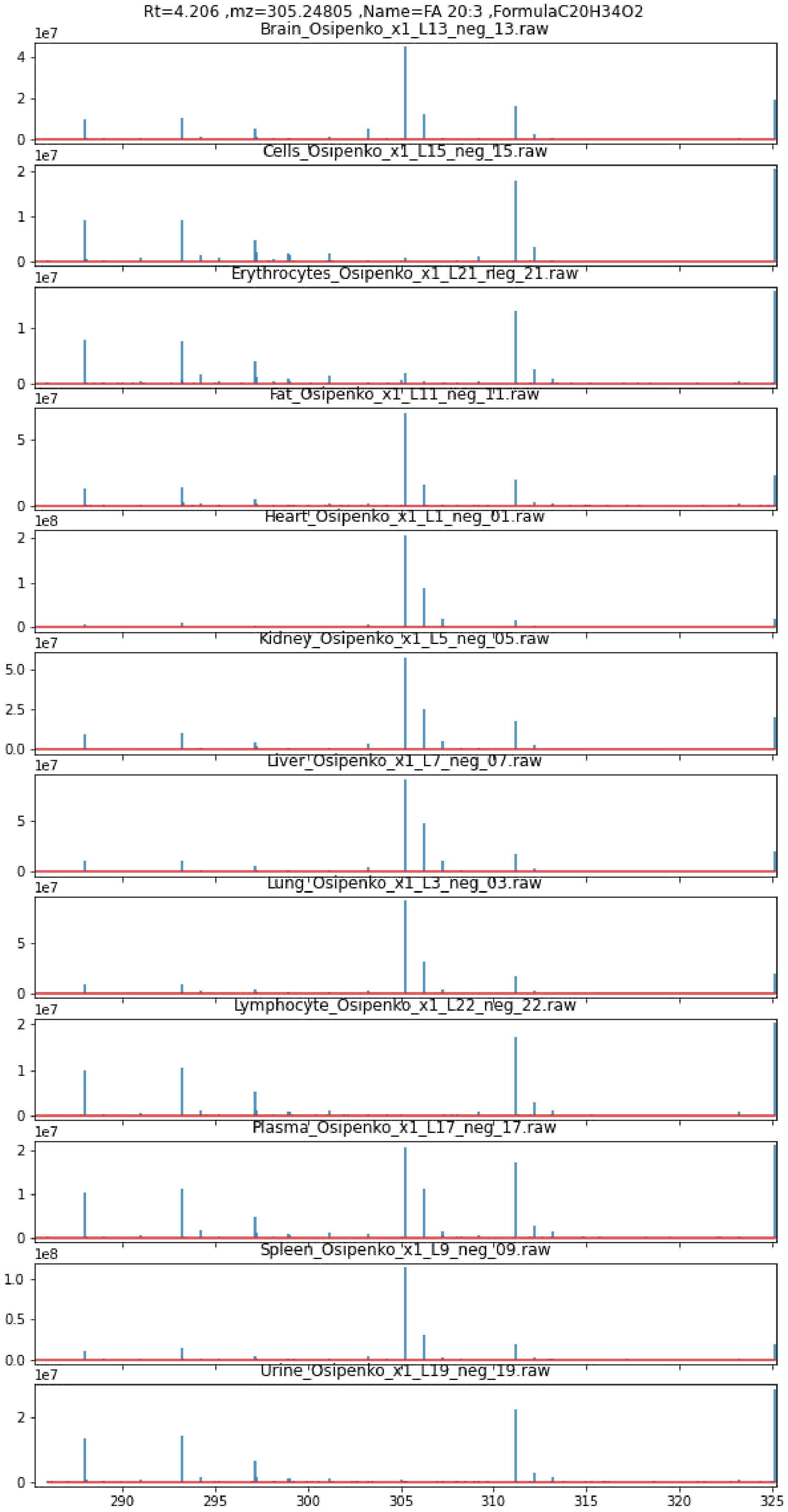

Rt=4.206 ,mz=305.24805 ,Name=FA 20:3 ,FormulaC20H34O2
Brain_Osipenko_x1_L13_neg_13.raw
Cells_Osipenko_x1_L15_neg_15.raw
Erythrocytes_Osipenko_x1_L21_neg_21.raw
Fat_Osipenko_x1_L11_neg_11.raw
Heart_Osipenko_x1_L1_neg_01.raw
Kidney_Osipenko_x1_L5_neg_05.raw
Liver_Osipenko_x1_L7_neg_07.raw
Lung_Osipenko_x1_L3_neg_03.raw
Lymphocyte_Osipenko_x1_L22_neg_22.raw
Plasma_Osipenko_x1_L17_neg_17.raw
Spleen_Osipenko_x1_L9_neg_09.raw
Urine_Osipenko_x1_L19_neg_19.raw
1e7
1e8
290
295
300
305
310
315
320
325

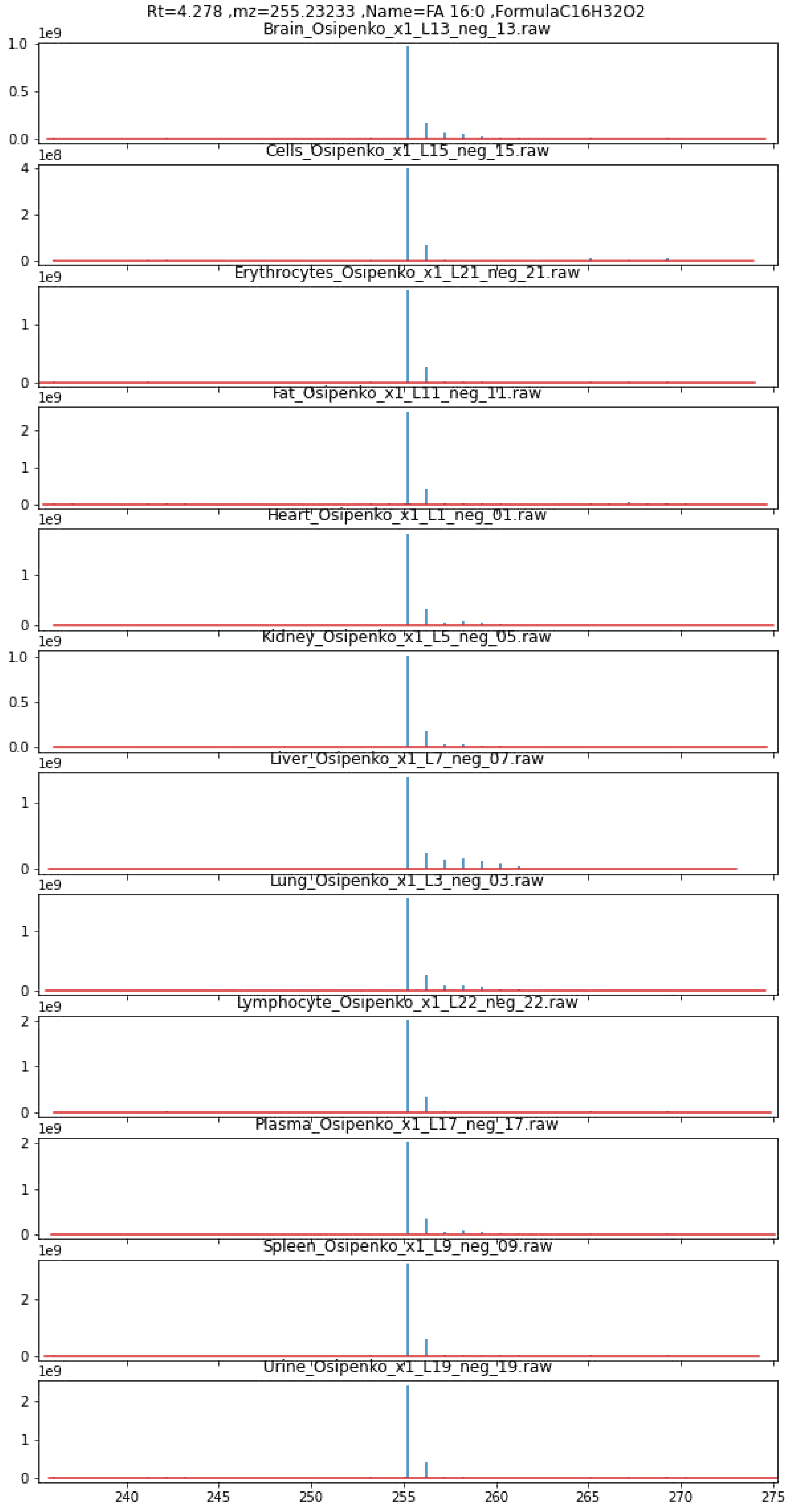
Rt=4.278 ,mz=255.23233 ,Name=FA 16:0 ,FormulaC16H32O2
Brain_Osipenko_x1_L13_neg_13.raw
Cells_Osipenko_x1_L15_neg_15.raw
Erythrocytes_Osipenko_x1_L21_neg_21.raw
Fat_Osipenko_x1_L11_neg_11.raw
Heart_Osipenko_x1_L1_neg_01.raw
Kidney_Osipenko_x1_L5_neg_05.raw
Liver_Osipenko_x1_L7_neg_07.raw
Lung_Osipenko_x1_L3_neg_03.raw
Lymphocyte_Osipenko_x1_L22_neg_22.raw
Plasma_Osipenko_x1_L17_neg_17.raw
Spleen_Osipenko_x1_L9_neg_09.raw
Urine_Osipenko_x1_L19_neg_19.raw
240
245
250
255
260
265
270
275

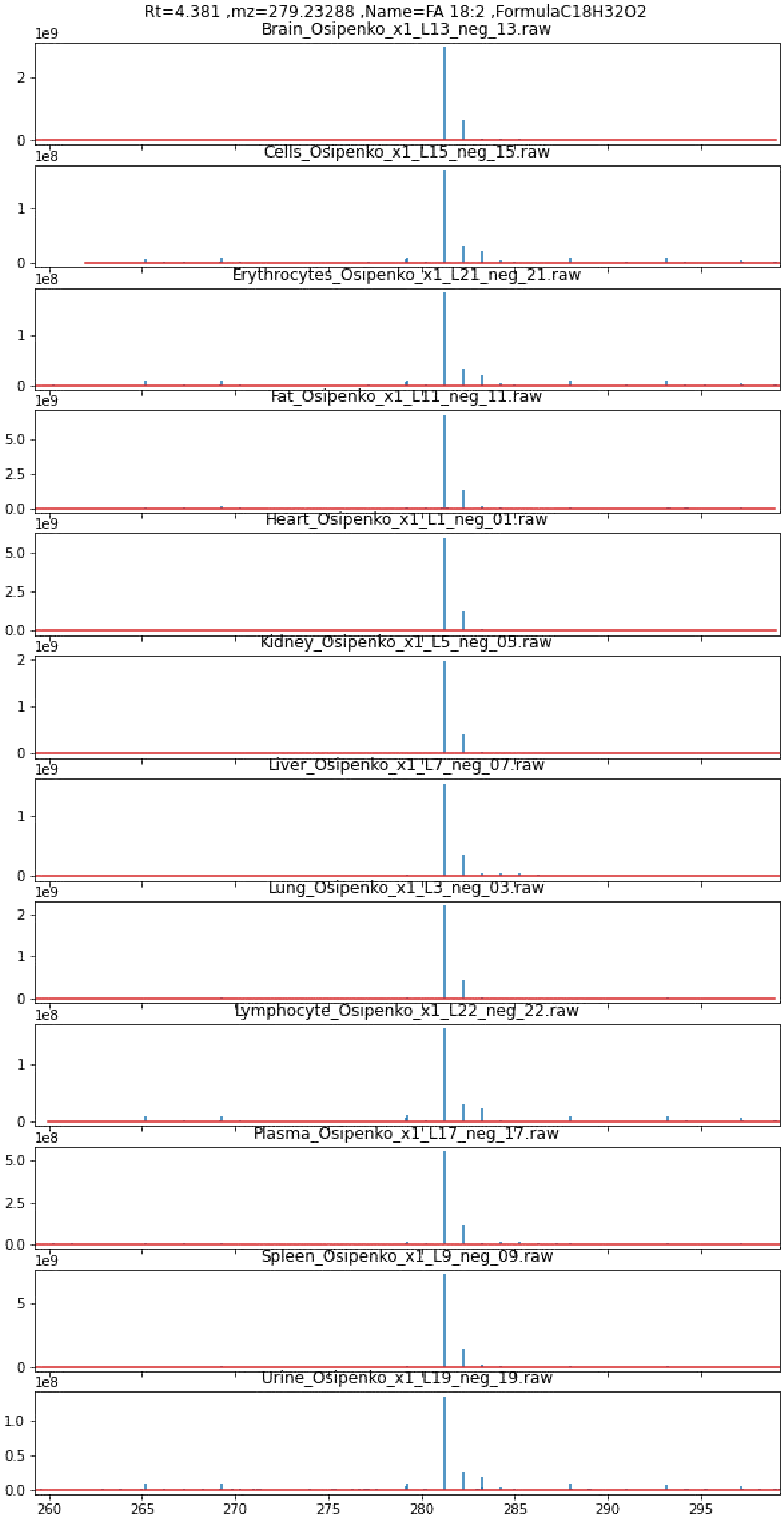
Rt=4.381 ,mz=279.23288 ,Name=FA 18:2 ,FormulaC18H32O2
Brain_Osipenko_x1_L13_neg_13.raw
Cells_Osipenko_x1_L15_neg_15.raw
Erythrocytes_Osipenko_x1_L21_neg_21.raw
Fat_Osipenko_x1_L11_neg_11.raw
Heart_Osipenko_x1_L1_neg_01.raw
Kidney_Osipenko_x1_L5_neg_05.raw
Liver_Osipenko_x1_L7_neg_07.raw
Lung_Osipenko_x1_L3_neg_03.raw
Lymphocyte_Osipenko_x1_L22_neg_22.raw
Plasma_Osipenko_x1_L17_neg_17.raw
Spleen_Osipenko_x1_L9_neg_09.raw
Urine_Osipenko_x1_L19_neg_19.raw

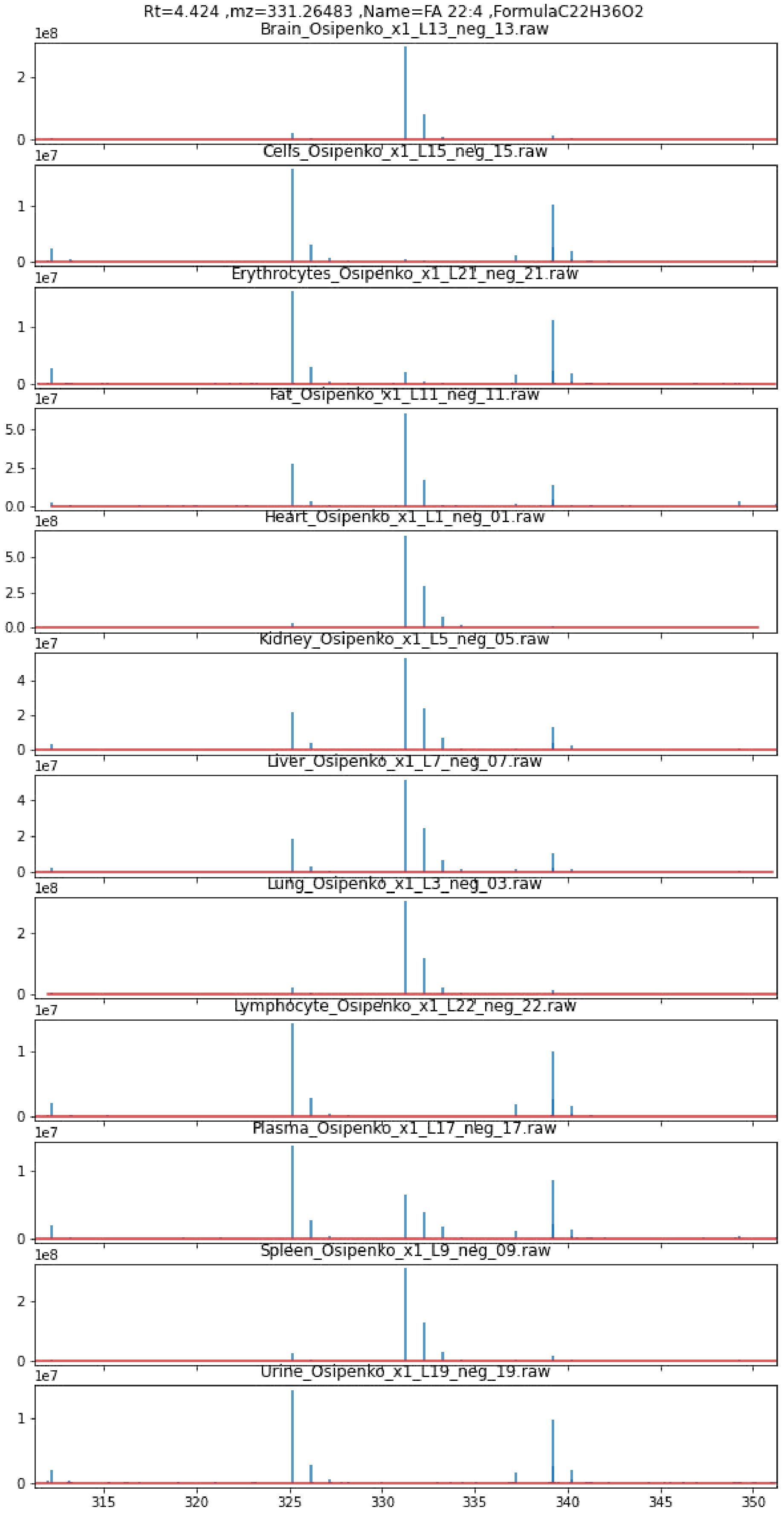
Rt=4.424 ,mz=331.26483 ,Name=FA 22:4 ,FormulaC22H36O2
Brain_Osipenko_x1_L13_neg_13.raw
Cells_Osipenko_x1_L15_neg_15.raw
Erythrocytes_Osipenko_x1_L21_neg_21.raw
Fat_Osipenko_x1_L11_neg_11.raw
Heart_Osipenko_x1_L1_neg_01.raw
Kidney_Osipenko_x1_L5_neg_05.raw
Liver_Osipenko_x1_L7_neg_07.raw
Lung_Osipenko_x1_L3_neg_03.raw
Lymphocyte_Osipenko_x1_L22_neg_22.raw
Plasma_Osipenko_x1_L17_neg_17.raw
Spleen_Osipenko_x1_L9_neg_09.raw
Urine_Osipenko_x1_L19_neg_19.raw
1e8
1e7
315
320
325
330
335
340
345
350

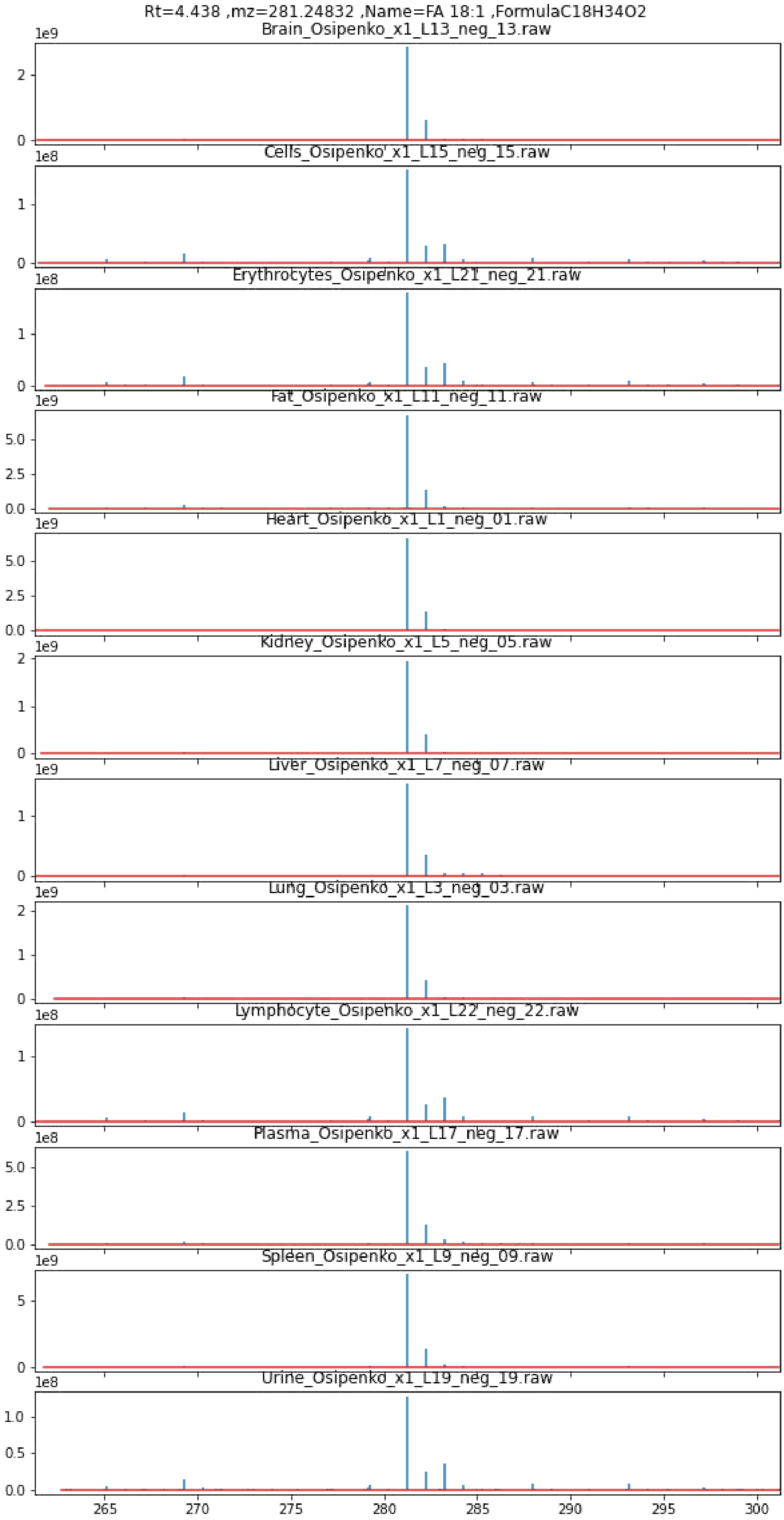
Rt=4.438 ,mz=281.24832 ,Name=FA 18:1 ,FormulaC18H34O2
Brain_Osipenko_x1_L13_neg_13.raw
Cells_Osipenko_x1_L15_neg_15.raw
Erythrocytes_Osipenko_x1_L21_neg_21.raw
Fat_Osipenko_x1_L11_neg_11.raw
Heart_Osipenko_x1_L1_neg_01.raw
Kidney_Osipenko_x1_L5_neg_05.raw
Liver_Osipenko_x1_L7_neg_07.raw
Lung_Osipenko_x1_L3_neg_03.raw
Lymphocyte_Osipenko_x1_L22_neg_22.raw
Plasma_Osipenko_x1_L17_neg_17.raw
Spleen_Osipenko_x1_L9_neg_09.raw
Urine_Osipenko_x1_L19_neg_19.raw
265
270
275
280
285
290
295
300

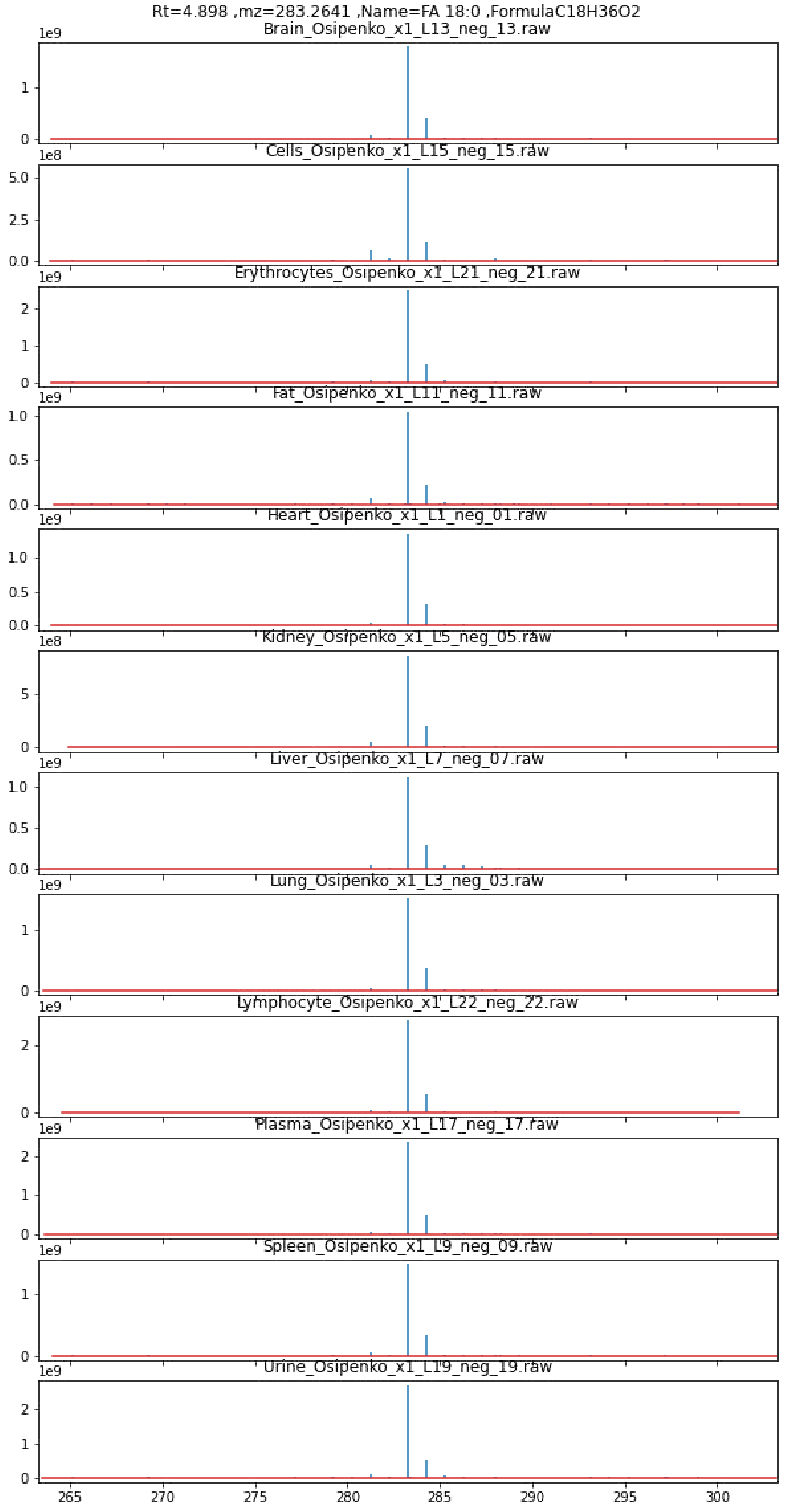

Rt=4.898 ,mz=283.2641 ,Name=FA 18:0 ,FormulaC18H36O2
Brain_Osipenko_x1_L13_neg_13.raw
Cells_Osipenko_x1_L15_neg_15.raw
Erythrocytes_Osipenko_x1_L21_neg_21.raw
Fat_Osipenko_x1_L11_neg_11.raw
Heart_Osipenko_x1_L1_neg_01.raw
Kidney_Osipenko_x1_L5_neg_05.raw
Liver_Osipenko_x1_L7_neg_07.raw
Lung_Osipenko_x1_L3_neg_03.raw
Lymphocyte_Osipenko_x1_L22_neg_22.raw
Plasma_Osipenko_x1_L17_neg_17.raw
Spleen_Osipenko_x1_L9_neg_09.raw
Urine_Osipenko_x1_L19_neg_19.raw
265 270 275 280 285 290 295 300

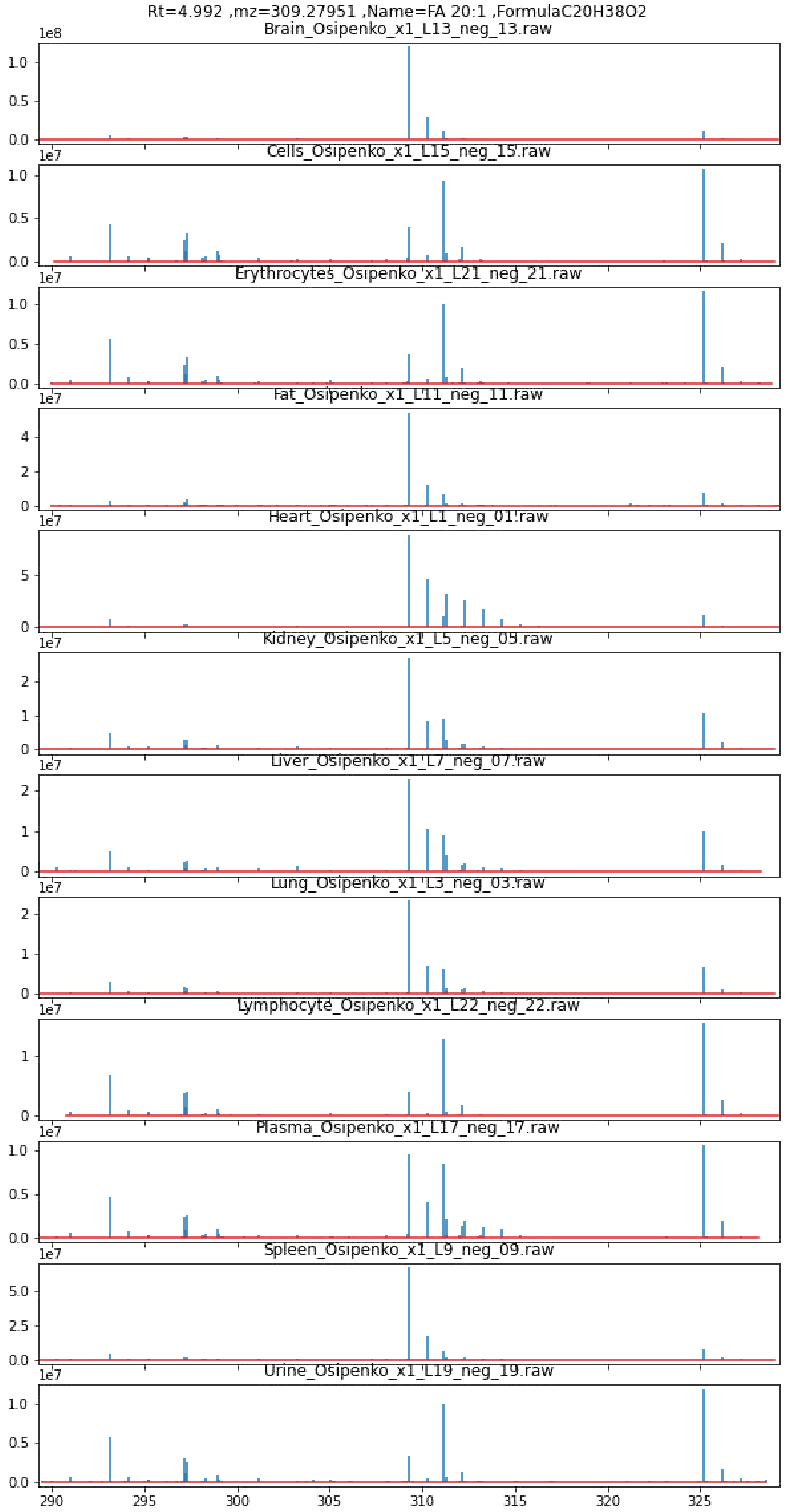
Rt=4.992 ,mz=309.27951 ,Name=FA 20:1 ,FormulaC20H38O2
Brain_Osipenko_x1_L13_neg_13.raw
Cells_Osipenko_x1_L15_neg_15.raw
Erythrocytes_Osipenko_x1_L21_neg_21.raw
Fat_Osipenko_x1_L11_neg_11.raw
Heart_Osipenko_x1_L1_neg_01.raw
Kidney_Osipenko_x1_L5_neg_05.raw
Liver_Osipenko_x1_L7_neg_07.raw
Lung_Osipenko_x1_L3_neg_03.raw
Lymphocyte_Osipenko_x1_L22_neg_22.raw
Plasma_Osipenko_x1_L17_neg_17.raw
Spleen_Osipenko_x1_L9_neg_09.raw
Urine_Osipenko_x1_L19_neg_19.raw
1e8
1e7
290
295
300
305
310
315
320
325

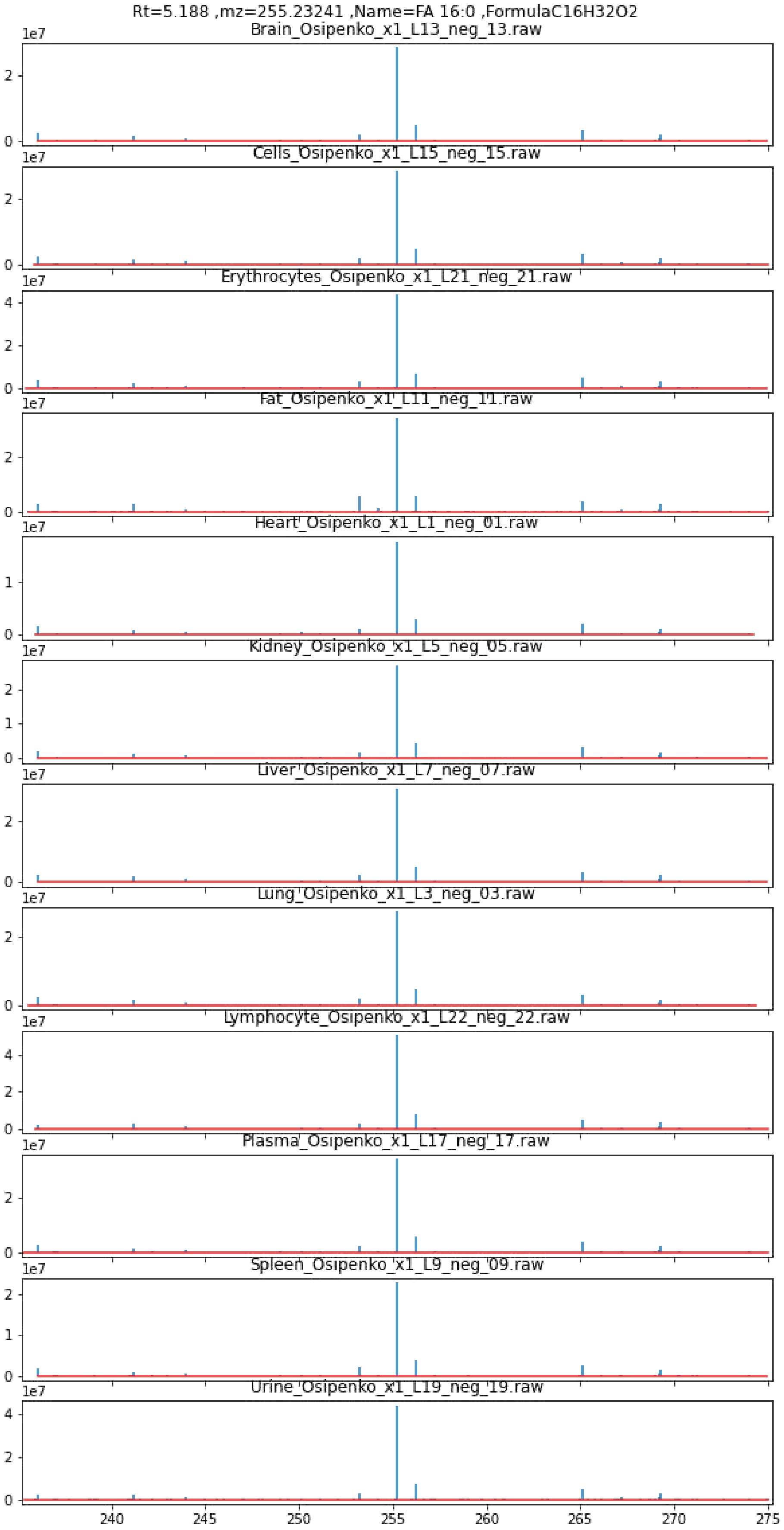
Rt=5.188 ,mz=255.23241 ,Name=FA 16:0 ,FormulaC16H32O2
Brain_Osipenko_x1_L13_neg_13.raw
Cells_Osipenko_x1_L15_neg_15.raw
Erythrocytes_Osipenko_x1_L21_neg_21.raw
Fat_Osipenko_x1_L11_neg_11.raw
Heart_Osipenko_x1_L1_neg_01.raw
Kidney_Osipenko_x1_L5_neg_05.raw
Liver_Osipenko_x1_L7_neg_07.raw
Lung_Osipenko_x1_L3_neg_03.raw
Lymphocyte_Osipenko_x1_L22_neg_22.raw
Plasma_Osipenko_x1_L17_neg_17.raw
Spleen_Osipenko_x1_L9_neg_09.raw
Urine_Osipenko_x1_L19_neg_19.raw
1e7
240
245
250
255
260
265
270
275

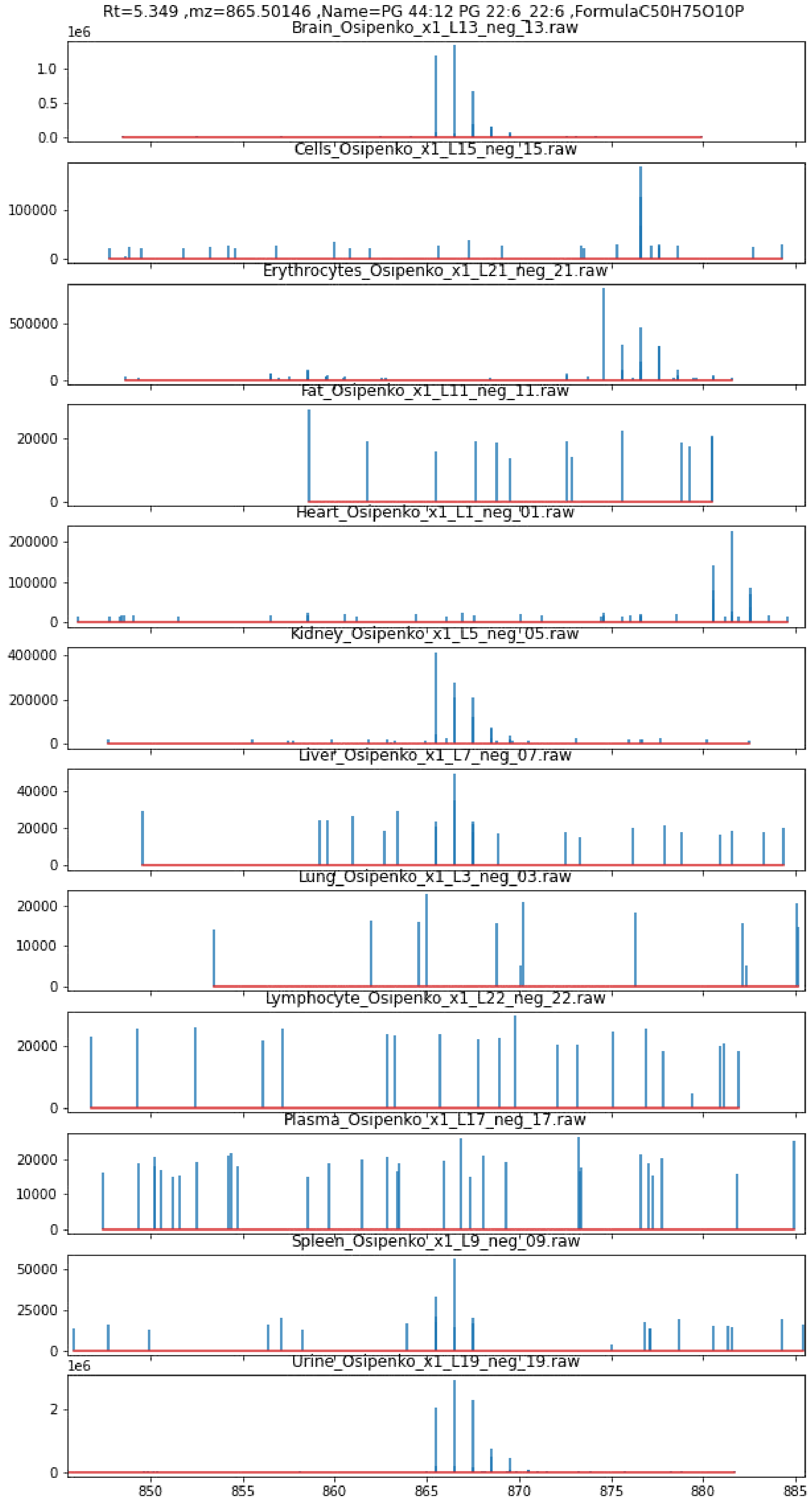

Rt=5.349 ,mz=865.50146 ,Name=PG 44:12 PG 22:6_22:6 ,FormulaC50H75O10P
Brain_Osipenko_x1_L13_neg_13.raw
Cells_Osipenko_x1_L15_neg_15.raw
Erythrocytes_Osipenko_x1_L21_neg_21.raw
Fat_Osipenko_x1_L11_neg_11.raw
Heart_Osipenko_x1_L1_neg_01.raw
Kidney_Osipenko_x1_L5_neg_05.raw
Liver_Osipenko_x1_L7_neg_07.raw
Lung_Osipenko_x1_L3_neg_03.raw
Lymphocyte_Osipenko_x1_L22_neg_22.raw
Plasma_Osipenko_x1_L17_neg_17.raw
Spleen_Osipenko_x1_L9_neg_09.raw
Urine_Osipenko_x1_L19_neg_19.raw

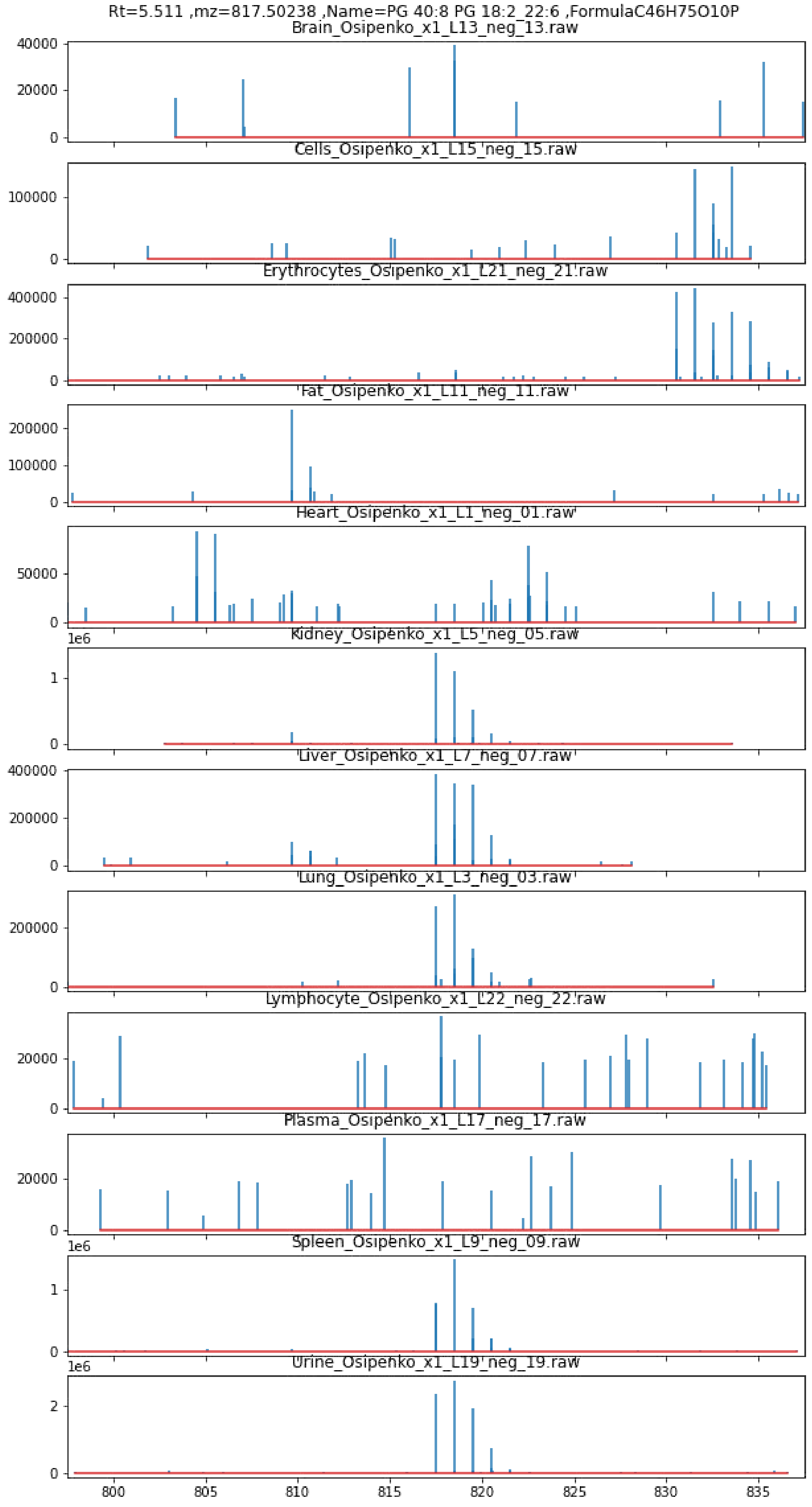

Rt=5.511 ,mz=817.50238 ,Name=PG 40:8 PG 18:2_22:6 ,FormulaC46H75O10P
Brain_Osipenko_x1_L13_neg_13.raw
Cells_Osipenko_x1_L15_neg_15.raw
Erythrocytes_Osipenko_x1_L21_neg_21.raw
Fat_Osipenko_x1_L11_neg_11.raw
Heart_Osipenko_x1_L1_neg_01.raw
Kidney_Osipenko_x1_L5_neg_05.raw
Liver_Osipenko_x1_L7_neg_07.raw
Lung_Osipenko_x1_L3_neg_03.raw
Lymphocyte_Osipenko_x1_L22_neg_22.raw
Plasma_Osipenko_x1_L17_neg_17.raw
Spleen_Osipenko_x1_L9_neg_09.raw
Urine_Osipenko_x1_L19_neg_19.raw
1e6
800
805
810
815
820
825
830
835

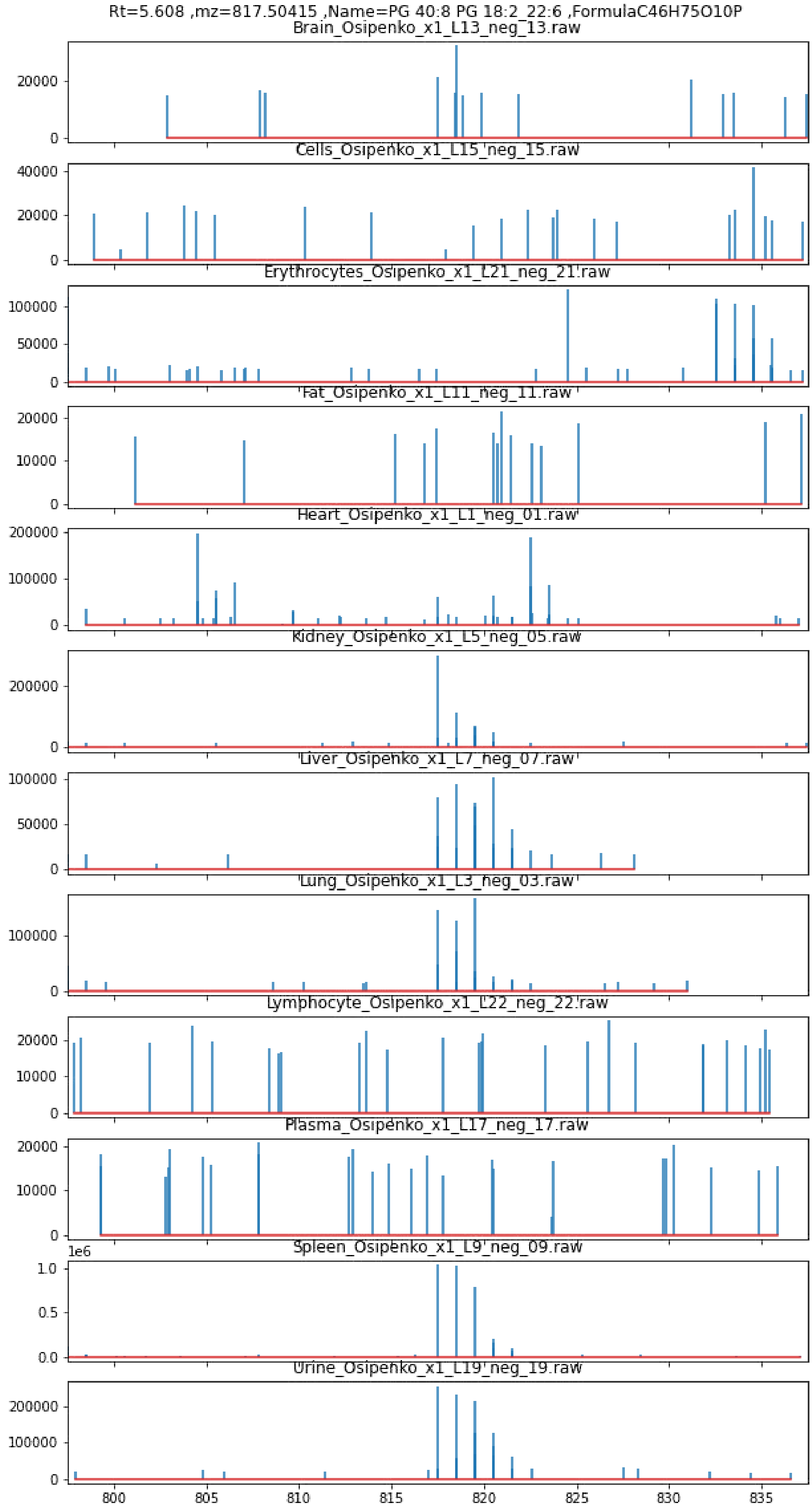
Rt=5.608 ,mz=817.50415 ,Name=PG 40:8 PG 18:2_22:6 ,FormulaC46H75O10P
Brain_Osipenko_x1_L13_neg_13.raw
Cells_Osipenko_x1_L15_neg_15.raw
Erythrocytes_Osipenko_x1_L21_neg_21.raw
Fat_Osipenko_x1_L11_neg_11.raw
Heart_Osipenko_x1_L1_neg_01.raw
Kidney_Osipenko_x1_L5_neg_05.raw
Liver_Osipenko_x1_L7_neg_07.raw
Lung_Osipenko_x1_L3_neg_03.raw
Lymphocyte_Osipenko_x1_L22_neg_22.raw
Plasma_Osipenko_x1_L17_neg_17.raw
Spleen_Osipenko_x1_L9_neg_09.raw
Urine_Osipenko_x1_L19_neg_19.raw
1e6
800
805
810
815
820
825
830
835

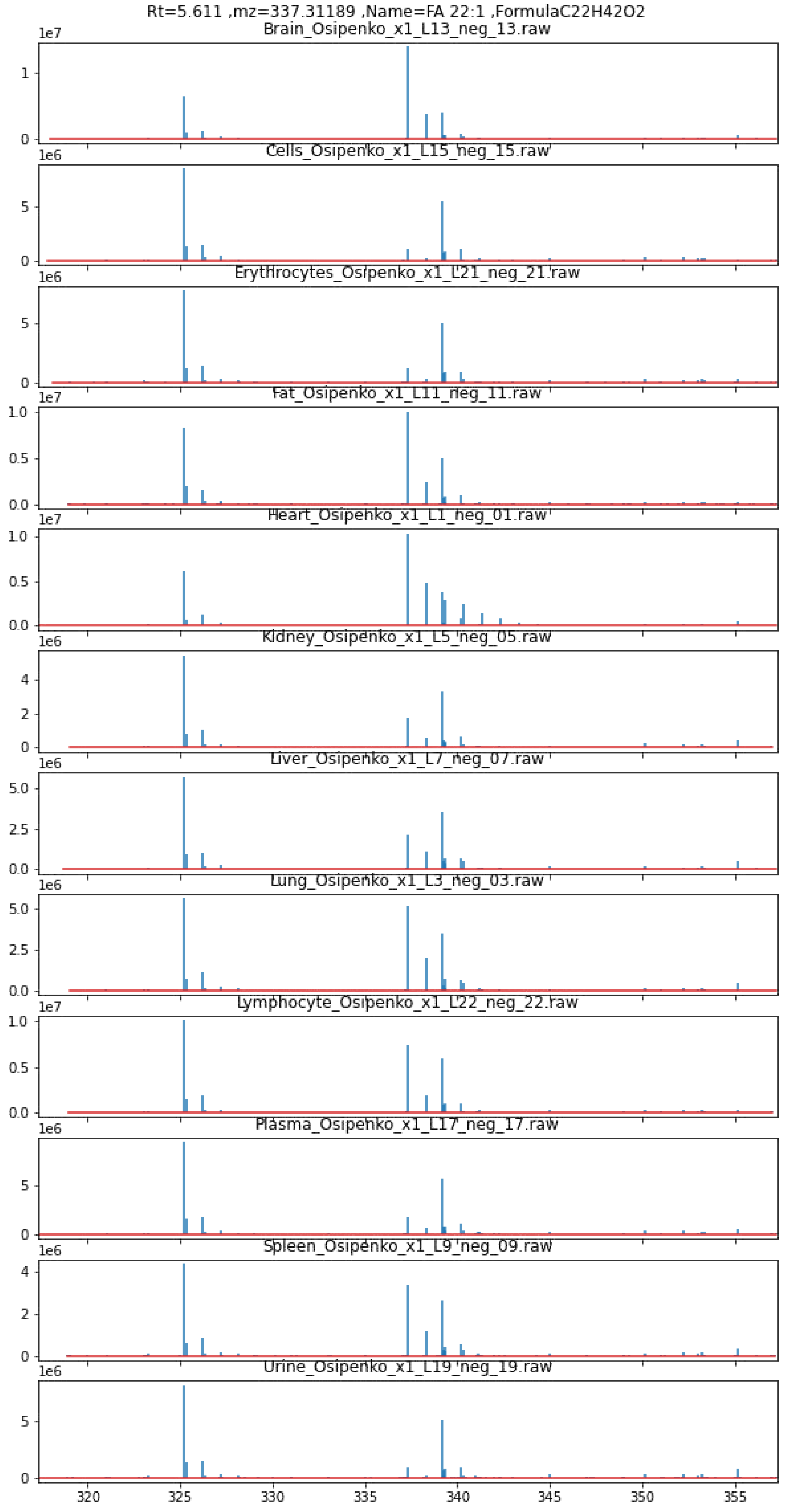

Rt=5.611 ,mz=337.31189 ,Name=FA 22:1 ,FormulaC22H42O2
Brain_Osipenko_x1_L13_neg_13.raw
Cells_Osipenko_x1_L15_neg_15.raw
Erythrocytes_Osipenko_x1_L21_neg_21.raw
Fat_Osipenko_x1_L11_neg_11.raw
Heart_Osipenko_x1_L1_neg_01.raw
Kidney_Osipenko_x1_L5_neg_05.raw
Liver_Osipenko_x1_L7_neg_07.raw
Lung_Osipenko_x1_L3_neg_03.raw
Lymphocyte_Osipenko_x1_L22_neg_22.raw
Plasma_Osipenko_x1_L17_neg_17.raw
Spleen_Osipenko_x1_L9_neg_09.raw
Urine_Osipenko_x1_L19_neg_19.raw

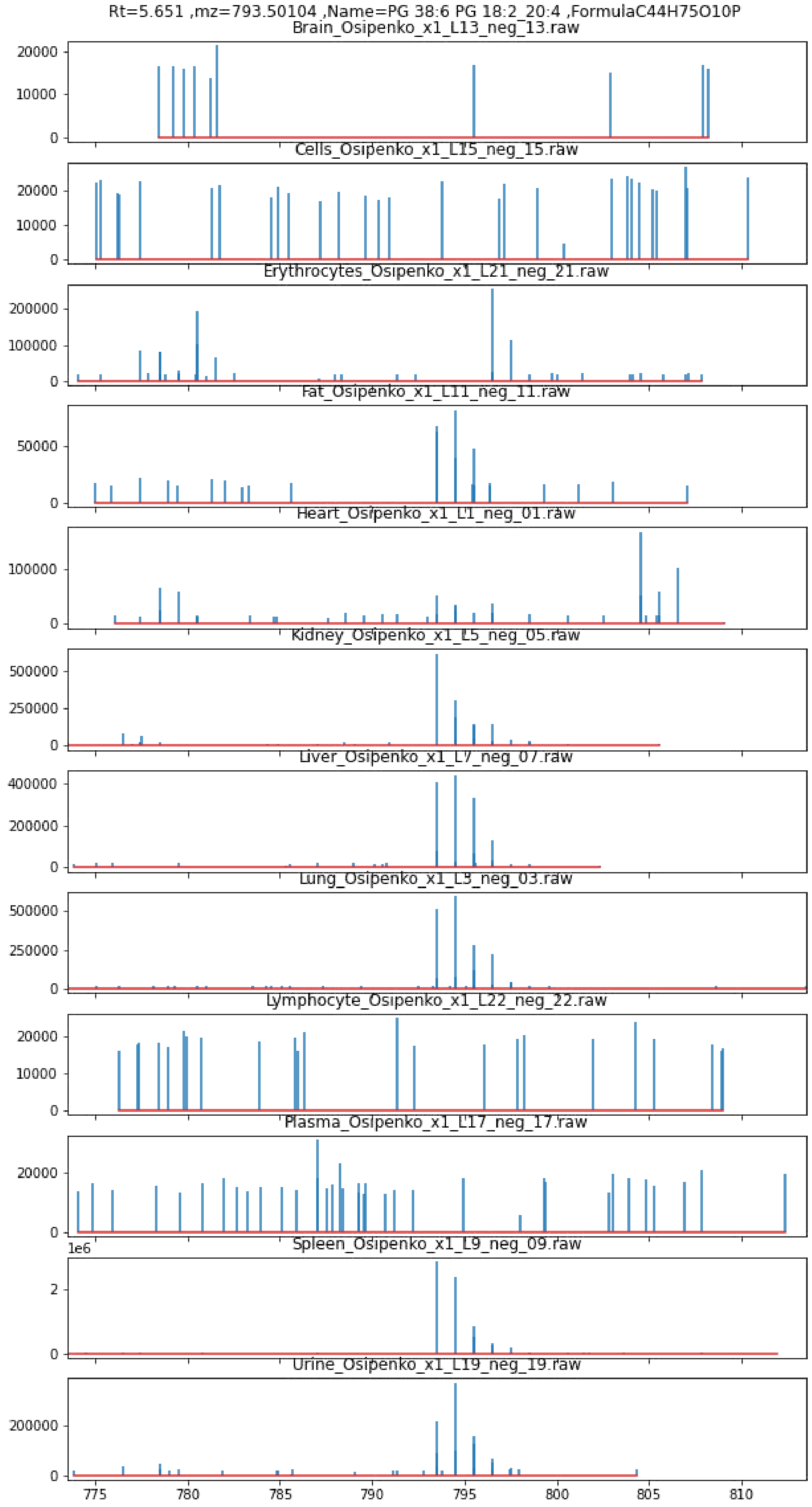
Rt=5.651 ,mz=793.50104 ,Name=PG 38:6 PG 18:2_20:4 ,FormulaC44H75O10P
Brain_Osipenko_x1_L13_neg_13.raw
Cells_Osipenko_x1_L15_neg_15.raw
Erythrocytes_Osipenko_x1_L21_neg_21.raw
Fat_Osipenko_x1_L11_neg_11.raw
Heart_Osipenko_x1_L1_neg_01.raw
Kidney_Osipenko_x1_L5_neg_05.raw
Liver_Osipenko_x1_L7_neg_07.raw
Lung_Osipenko_x1_L3_neg_03.raw
Lymphocyte_Osipenko_x1_L22_neg_22.raw
Plasma_Osipenko_x1_L17_neg_17.raw
1e6
Spleen_Osipenko_x1_L9_neg_09.raw
Urine_Osipenko_x1_L19_neg_19.raw
775
780
785
790
795
800
805
810

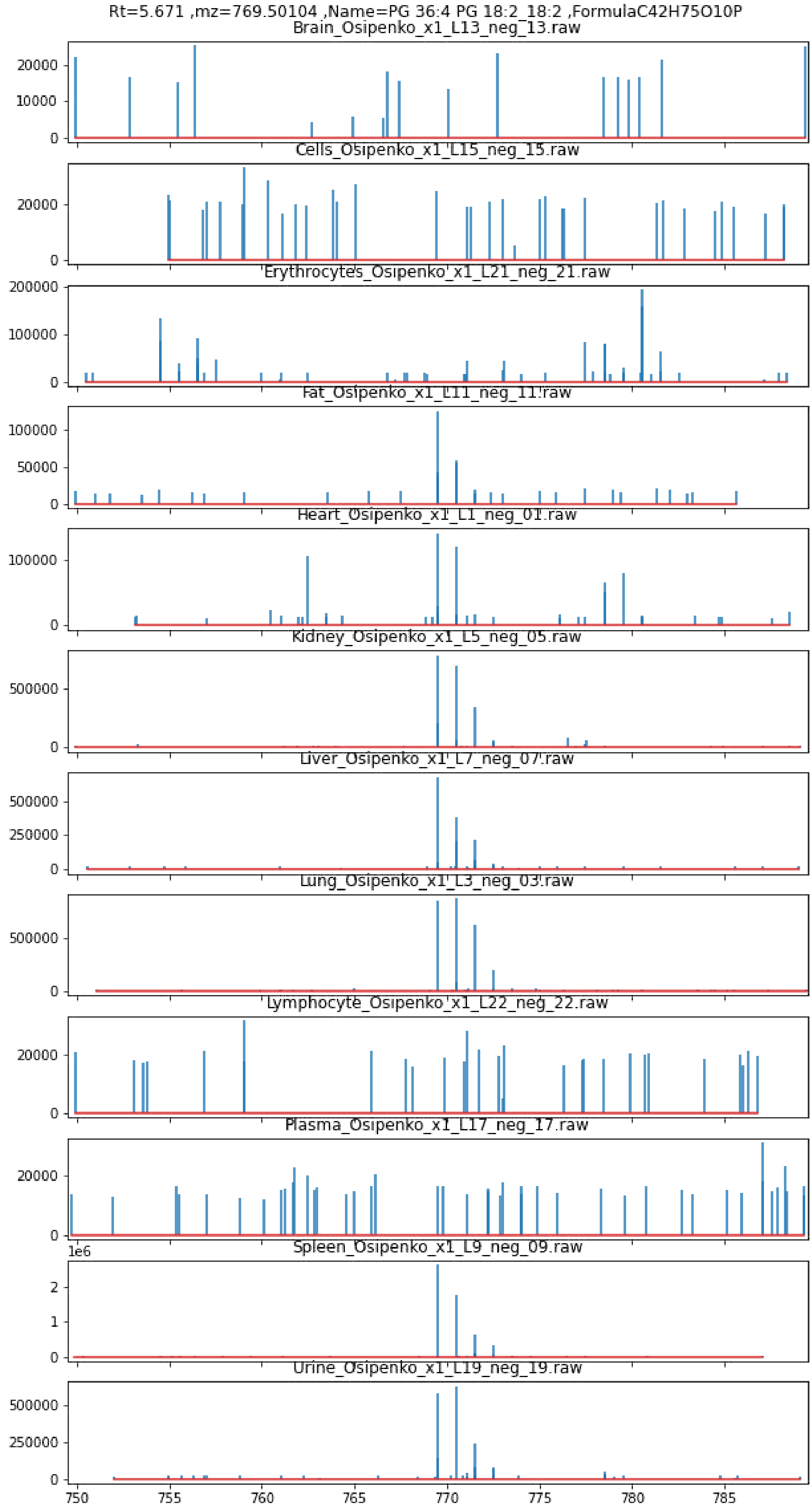

Rt=5.671 ,mz=769.50104 ,Name=PG 36:4 PG 18:2_18:2 ,FormulaC42H75O10P
Brain_Osipenko_x1_L13_neg_13.raw
Cells_Osipenko_x1_L15_neg_15.raw
Erythrocytes_Osipenko_x1_L21_neg_21.raw
Fat_Osipenko_x1_L11_neg_11.raw
Heart_Osipenko_x1_L1_neg_01.raw
Kidney_Osipenko_x1_L5_neg_05.raw
Liver_Osipenko_x1_L7_neg_07.raw
Lung_Osipenko_x1_L3_neg_03.raw
Lymphocyte_Osipenko_x1_L22_neg_22.raw
Plasma_Osipenko_x1_L17_neg_17.raw
1e6
Spleen_Osipenko_x1_L9_neg_09.raw
Urine_Osipenko_x1_L19_neg_19.raw
750
755
760
765
770
775
780
785

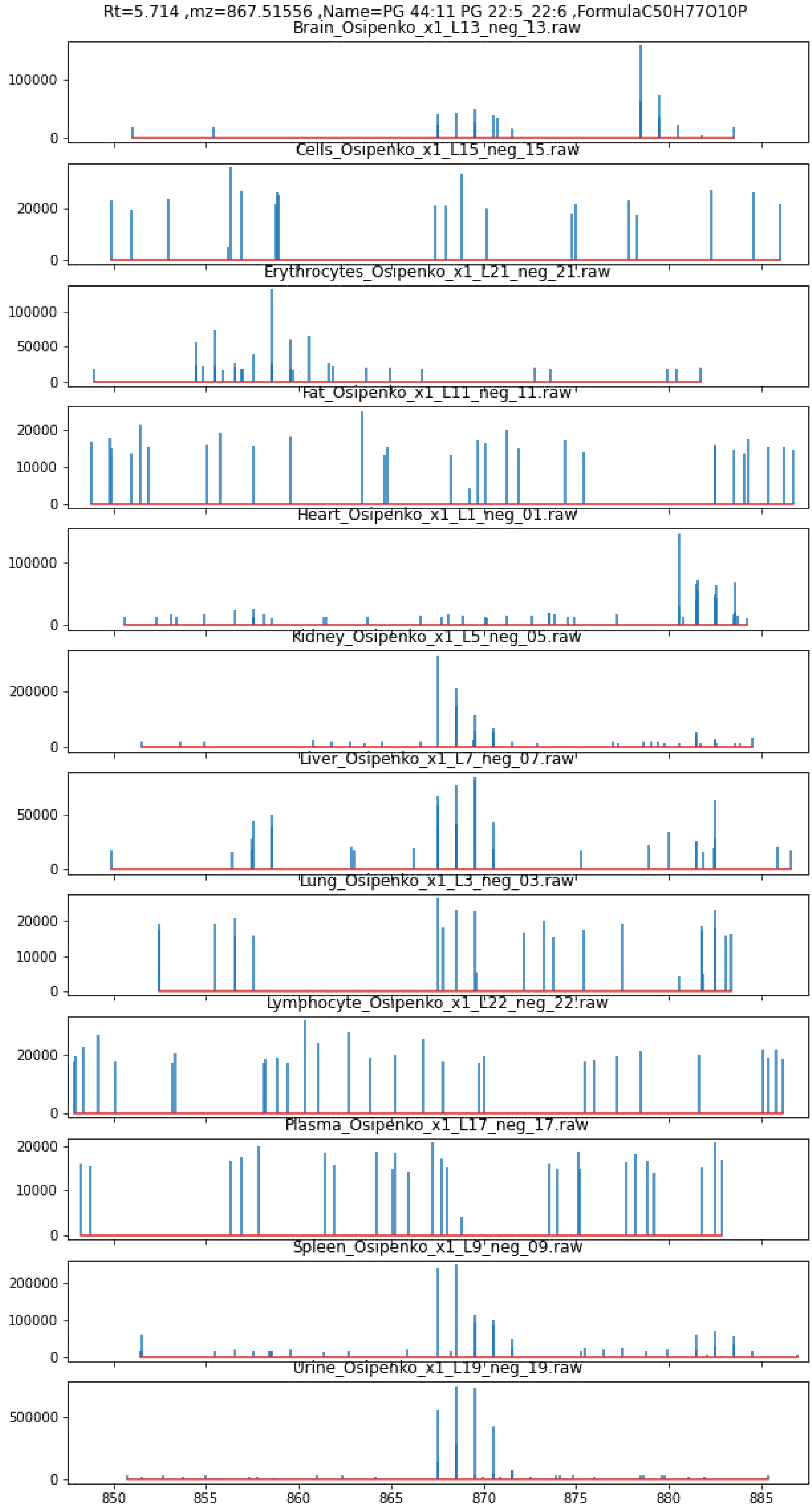

Rt=5.714 ,mz=867.51556 ,Name=PG 44:11 PG 22:5_22:6 ,FormulaC50H77O10P
Brain_Osipenko_x1_L13_neg_13.raw
Cells_Osipenko_x1_L15_neg_15.raw
Erythrocytes_Osipenko_x1_L21_neg_21.raw
Fat_Osipenko_x1_L11_neg_11.raw
Heart_Osipenko_x1_L1_neg_01.raw
Kidney_Osipenko_x1_L5_neg_05.raw
Liver_Osipenko_x1_L7_neg_07.raw
Lung_Osipenko_x1_L3_neg_03.raw
Lymphocyte_Osipenko_x1_L22_neg_22.raw
Plasma_Osipenko_x1_L17_neg_17.raw
Spleen_Osipenko_x1_L9_neg_09.raw
Urine_Osipenko_x1_L19_neg_19.raw
850
855
860
865
870
875
880
885

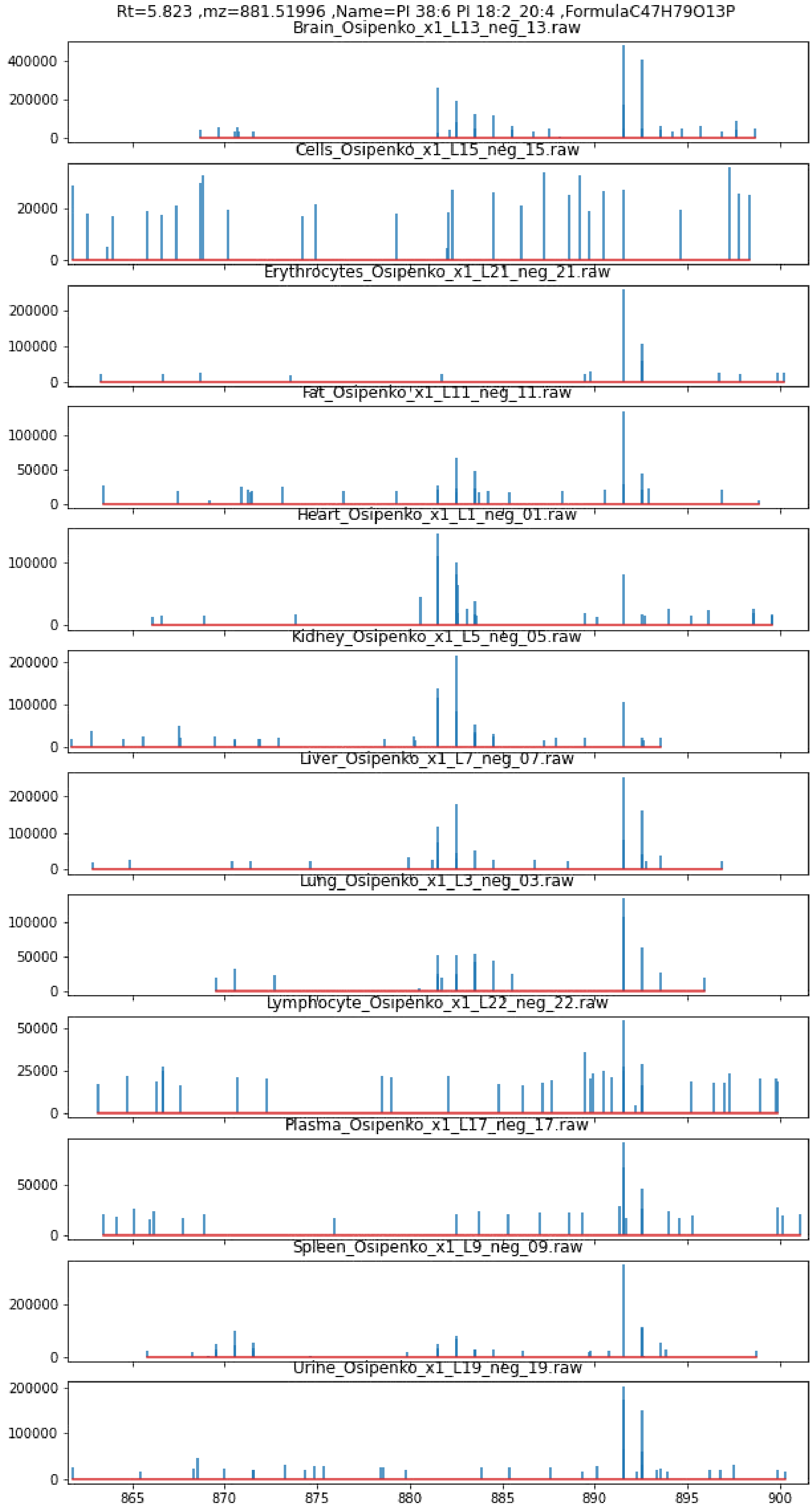

Rt=5.823 ,mz=881.51996 ,Name=PI 38:6 PI 18:2_20:4 ,FormulaC47H79O13P
Brain_Osipenko_x1_L13_neg_13.raw
Cells_Osipenko_x1_L15_neg_15.raw
Erythrocytes_Osipenko_x1_L21_neg_21.raw
Fat_Osipenko_x1_L11_neg_11.raw
Heart_Osipenko_x1_L1_neg_01.raw
Kidney_Osipenko_x1_L5_neg_05.raw
Liver_Osipenko_x1_L7_neg_07.raw
Lung_Osipenko_x1_L3_neg_03.raw
Lymphocyte_Osipenko_x1_L22_neg_22.raw
Plasma_Osipenko_x1_L17_neg_17.raw
Spleen_Osipenko_x1_L9_neg_09.raw
Urine_Osipenko_x1_L19_neg_19.raw
865
870
875
880
885
890
895
900

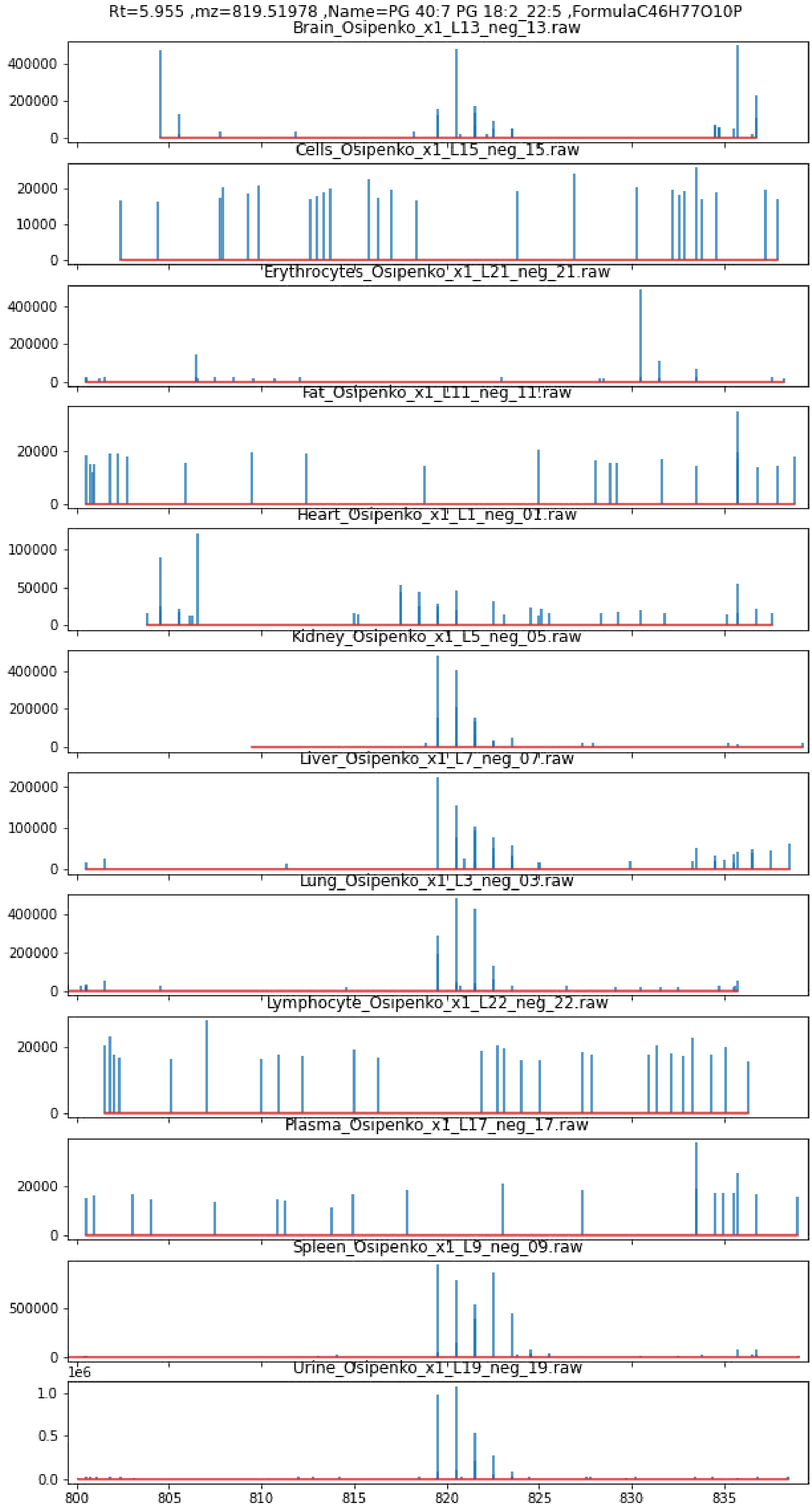
Rt=5.955 ,mz=819.51978 ,Name=PG 40:7 PG 18:2_22:5 ,FormulaC46H77O10P
Brain_Osipenko_x1_L13_neg_13.raw
Cells_Osipenko_x1_L15_neg_15.raw
Erythrocytes_Osipenko_x1_L21_neg_21.raw
Fat_Osipenko_x1_L11_neg_11.raw
Heart_Osipenko_x1_L1_neg_01.raw
Kidney_Osipenko_x1_L5_neg_05.raw
Liver_Osipenko_x1_L7_neg_07.raw
Lung_Osipenko_x1_L3_neg_03.raw
Lymphocyte_Osipenko_x1_L22_neg_22.raw
Plasma_Osipenko_x1_L17_neg_17.raw
Spleen_Osipenko_x1_L9_neg_09.raw
Urine_Osipenko_x1_L19_neg_19.raw
1e6
800
805
810
815
820
825
830
835

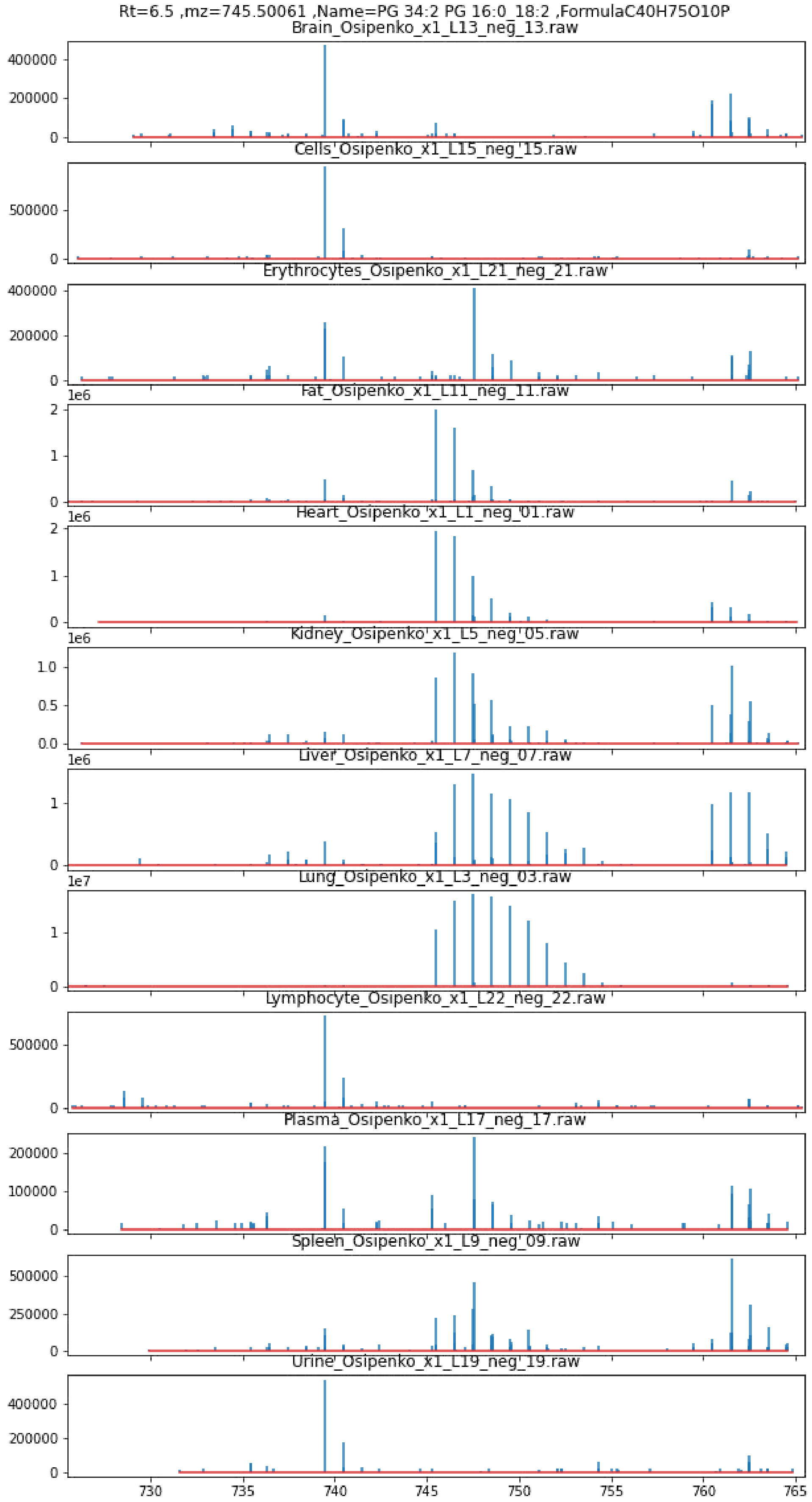
Rt=6.5 ,mz=745.50061 ,Name=PG 34:2 PG 16:0_18:2 ,FormulaC40H75O10P
Brain_Osipenko_x1_L13_neg_13.raw
Cells_Osipenko_x1_L15_neg_15.raw
Erythrocytes_Osipenko_x1_L21_neg_21.raw
Fat_Osipenko_x1_L11_neg_11.raw
Heart_Osipenko_x1_L1_neg_01.raw
Kidney_Osipenko_x1_L5_neg_05.raw
Liver_Osipenko_x1_L7_neg_07.raw
Lung_Osipenko_x1_L3_neg_03.raw
Lymphocyte_Osipenko_x1_L22_neg_22.raw
Plasma_Osipenko_x1_L17_neg_17.raw
Spleen_Osipenko_x1_L9_neg_09.raw
Urine_Osipenko_x1_L19_neg_19.raw
1e6
1e7
730
735
740
745
750
755
760
765

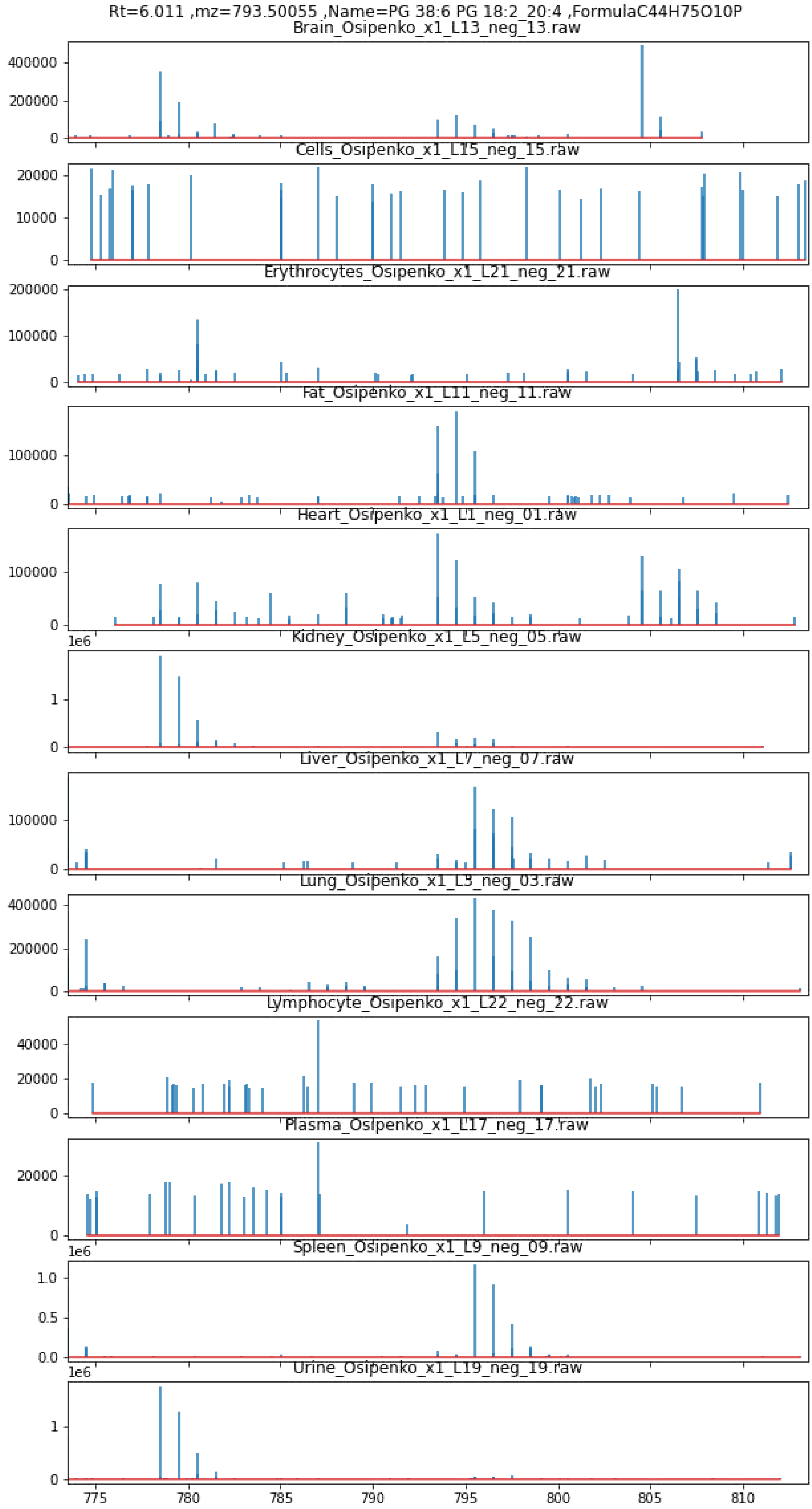

Rt=6.011 ,mz=793.50055 ,Name=PG 38:6 PG 18:2_20:4 ,FormulaC44H75O10P
Brain_Osipenko_x1_L13_neg_13.raw
Cells_Osipenko_x1_L15_neg_15.raw
Erythrocytes_Osipenko_x1_L21_neg_21.raw
Fat_Osipenko_x1_L11_neg_11.raw
Heart_Osipenko_x1_L1_neg_01.raw
Kidney_Osipenko_x1_L5_neg_05.raw
Liver_Osipenko_x1_L7_neg_07.raw
Lung_Osipenko_x1_L3_neg_03.raw
Lymphocyte_Osipenko_x1_L22_neg_22.raw
Plasma_Osipenko_x1_L17_neg_17.raw
Spleen_Osipenko_x1_L9_neg_09.raw
Urine_Osipenko_x1_L19_neg_19.raw
1e6
775
780
785
790
795
800
805
810

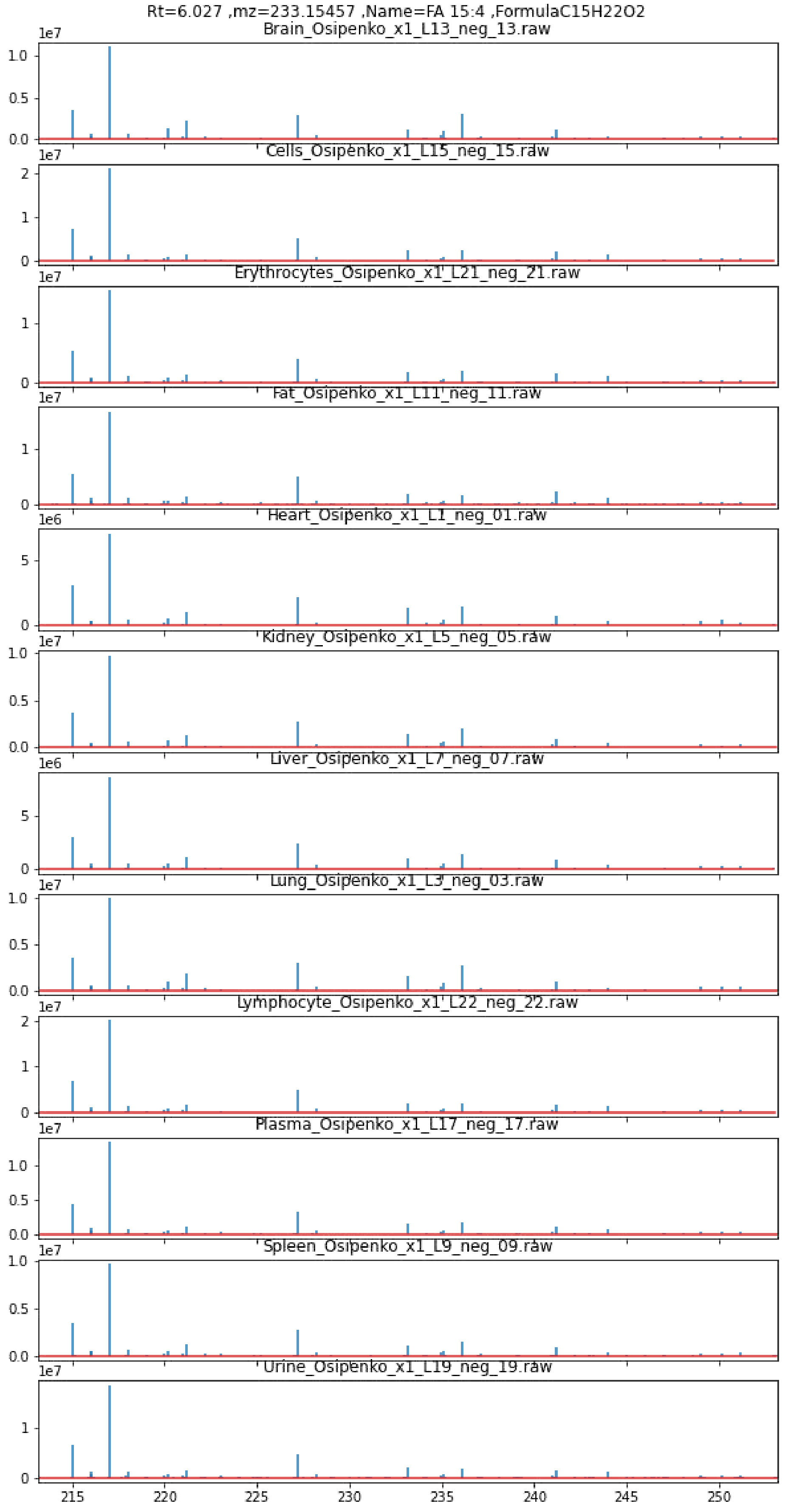

Rt=6.027 ,mz=233.15457 ,Name=FA 15:4 ,FormulaC15H22O2
Brain_Osipenko_x1_L13_neg_13.raw
Cells_Osipenko_x1_L15_neg_15.raw
Erythrocytes_Osipenko_x1_L21_neg_21.raw
Fat_Osipenko_x1_L11_neg_11.raw
Heart_Osipenko_x1_L1_neg_01.raw
Kidney_Osipenko_x1_L5_neg_05.raw
Liver_Osipenko_x1_L7_neg_07.raw
Lung_Osipenko_x1_L3_neg_03.raw
Lymphocyte_Osipenko_x1_L22_neg_22.raw
Plasma_Osipenko_x1_L17_neg_17.raw
Spleen_Osipenko_x1_L9_neg_09.raw
Urine_Osipenko_x1_L19_neg_19.raw
215
220
225
230
235
240
245
250

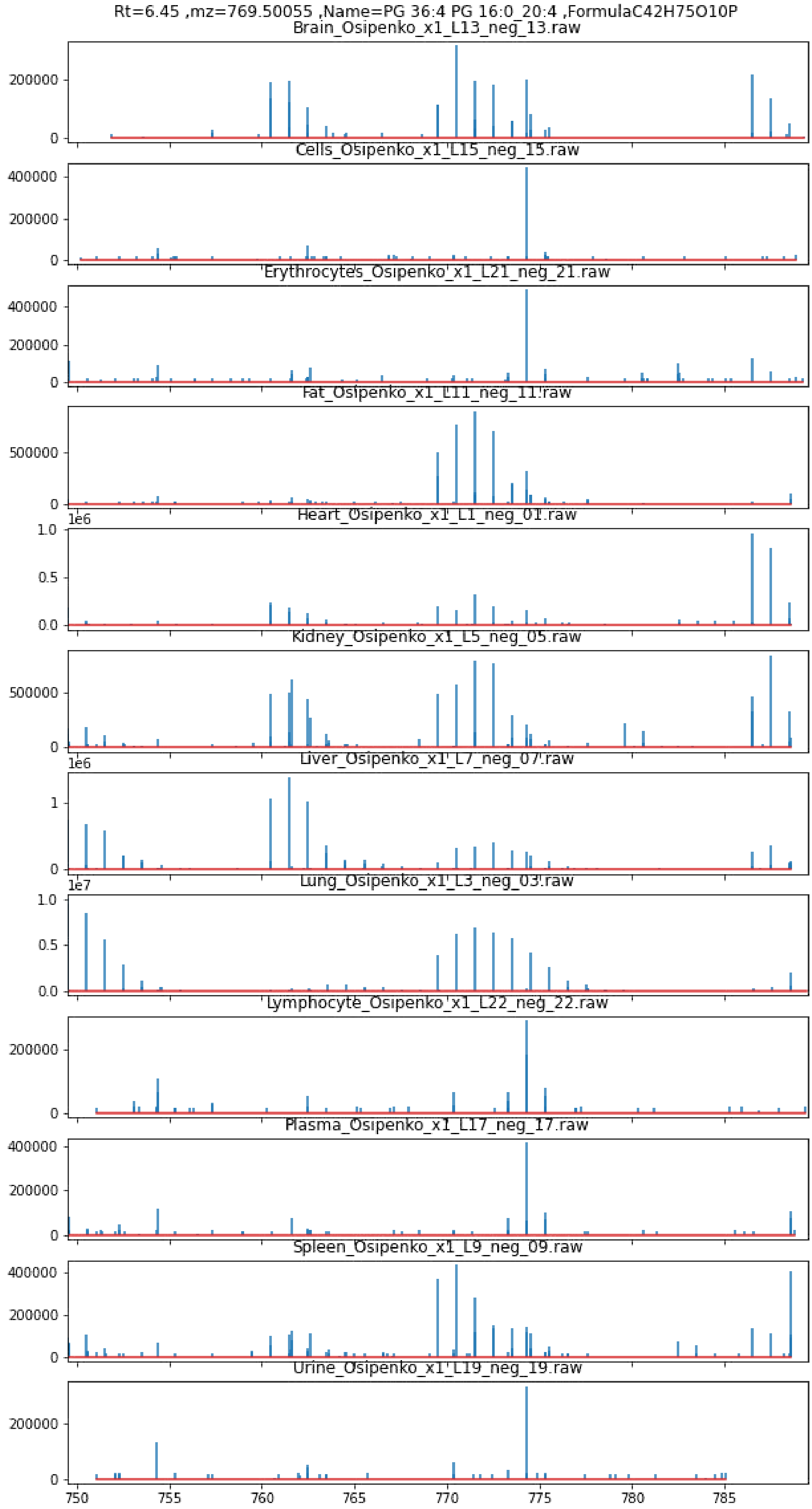

Rt=6.45 ,mz=769.50055 ,Name=PG 36:4 PG 16:0_20:4 ,FormulaC42H75O10P
Brain_Osipenko_x1_L13_neg_13.raw
Cells_Osipenko_x1_L15_neg_15.raw
Erythrocytes_Osipenko_x1_L21_neg_21.raw
Fat_Osipenko_x1_L11_neg_11.raw
Heart_Osipenko_x1_L1_neg_01.raw
Kidney_Osipenko_x1_L5_neg_05.raw
Liver_Osipenko_x1_L7_neg_07.raw
Lung_Osipenko_x1_L3_neg_03.raw
Lymphocyte_Osipenko_x1_L22_neg_22.raw
Plasma_Osipenko_x1_L17_neg_17.raw
Spleen_Osipenko_x1_L9_neg_09.raw
Urine_Osipenko_x1_L19_neg_19.raw
1e6
1e6
1e7
750
755
760
765
770
775
780
785

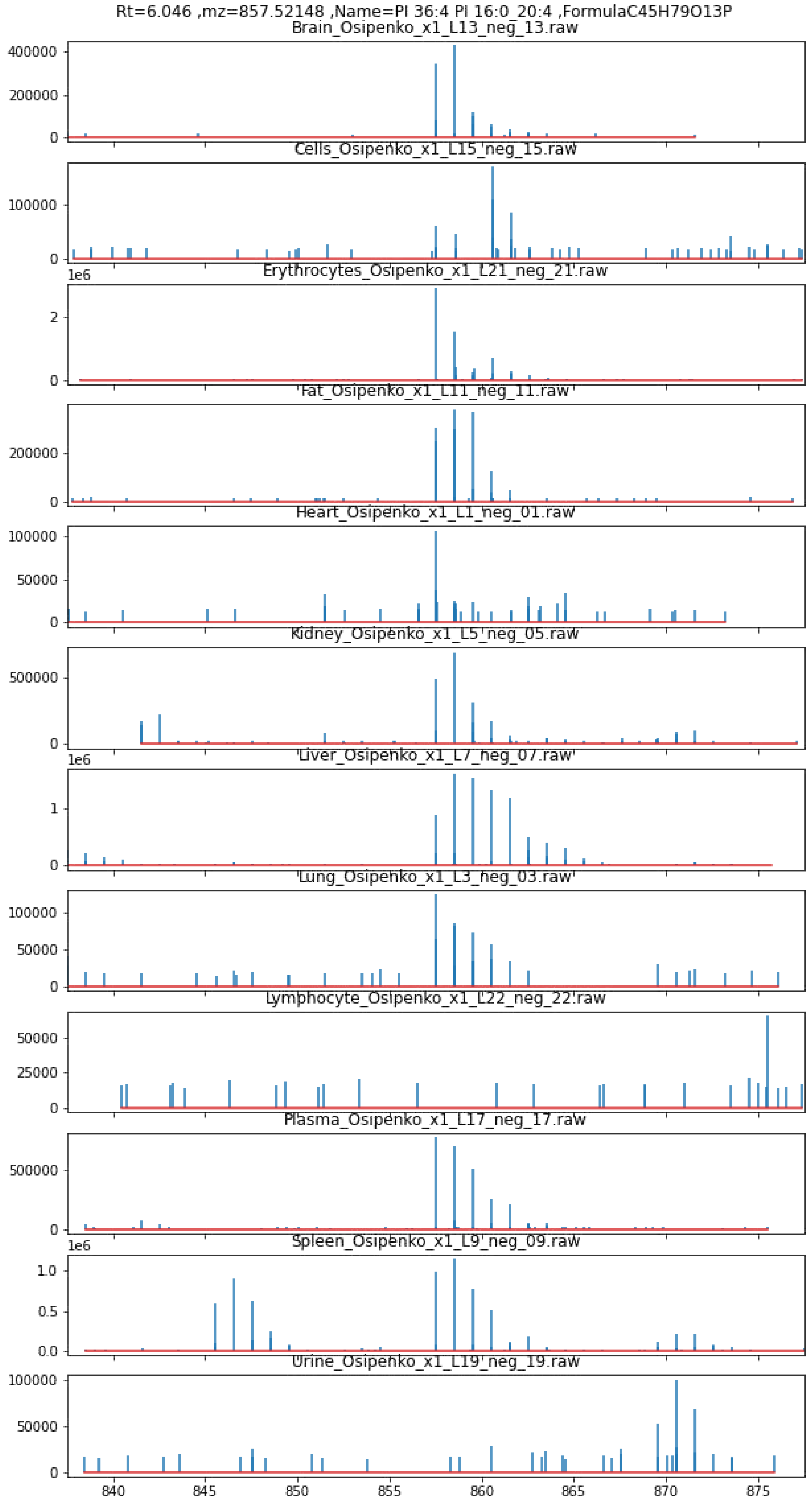
Rt=6.046 ,mz=857.52148 ,Name=PI 36:4 PI 16:0_20:4 ,FormulaC45H79O13P
Brain_Osipenko_x1_L13_neg_13.raw
Cells_Osipenko_x1_L15_neg_15.raw
Erythrocytes_Osipenko_x1_L21_neg_21.raw
'Fat_Osipenko_x1_L11_neg_11.raw'
Heart_Osipenko_x1_L1_neg_01.raw
Kidney_Osipenko_x1_L5_neg_05.raw
'Liver_Osipenko_x1_L7_neg_07.raw'
'Lung_Osipenko_x1_L3_neg_03.raw'
Lymphocyte_Osipenko_x1_L22_neg_22.raw
Plasma_Osipenko_x1_L17_neg_17.raw
Spleen_Osipenko_x1_L9_neg_09.raw
Urine_Osipenko_x1_L19_neg_19.raw
1e6
840
845
850
855
860
865
870
875

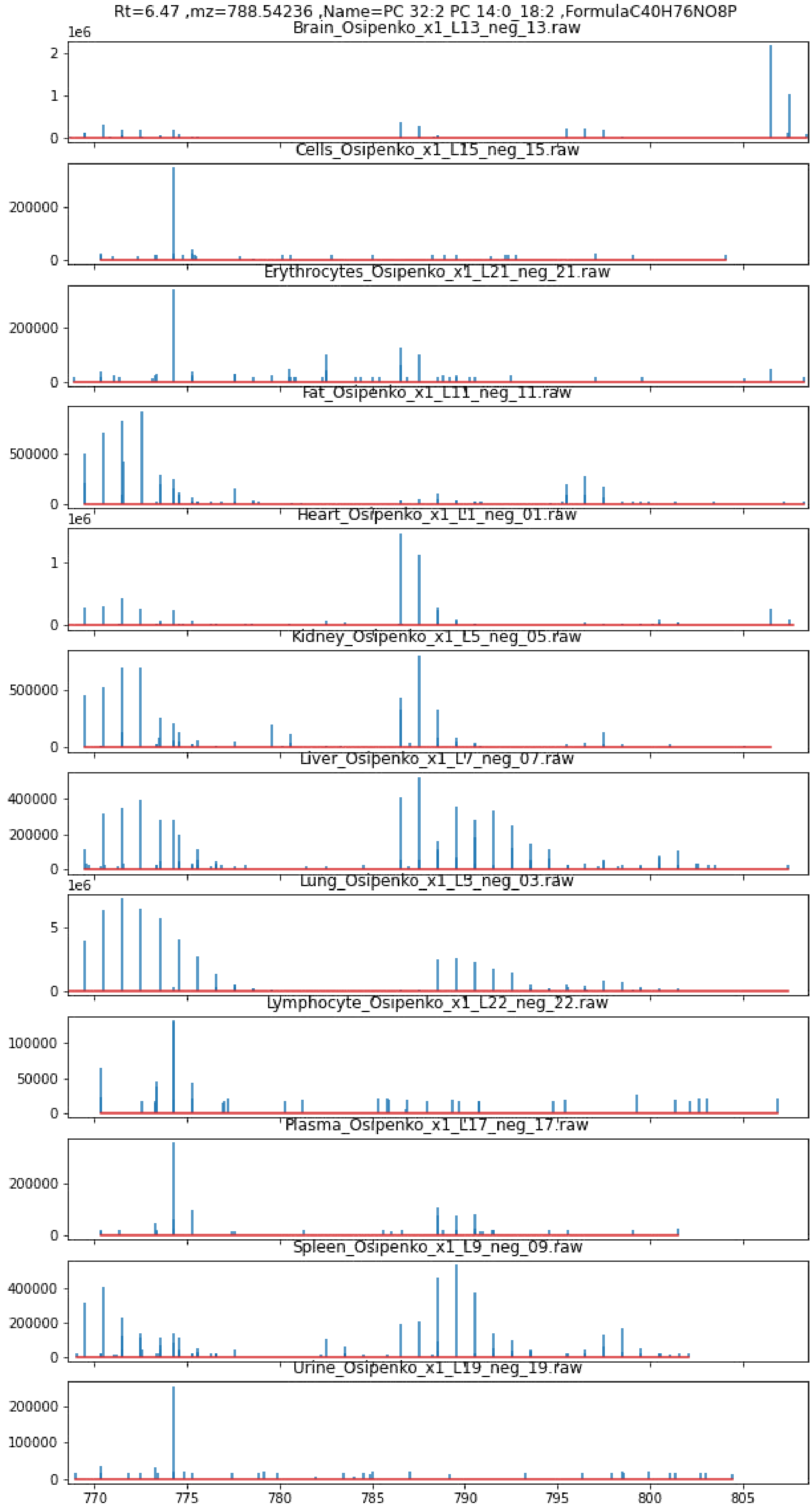

Rt=6.47 ,mz=788.54236 ,Name=PC 32:2 PC 14:0_18:2 ,FormulaC40H76NO8P
Brain_Osipenko_x1_L13_neg_13.raw
Cells_Osipenko_x1_L15_neg_15.raw
Erythrocytes_Osipenko_x1_L21_neg_21.raw
Fat_Osipenko_x1_L11_neg_11.raw
Heart_Osipenko_x1_L1_neg_01.raw
Kidney_Osipenko_x1_L5_neg_05.raw
Liver_Osipenko_x1_L7_neg_07.raw
Lung_Osipenko_x1_L3_neg_03.raw
Lymphocyte_Osipenko_x1_L22_neg_22.raw
Plasma_Osipenko_x1_L17_neg_17.raw
Spleen_Osipenko_x1_L9_neg_09.raw
Urine_Osipenko_x1_L19_neg_19.raw
770
775
780
785
790
795
800
805

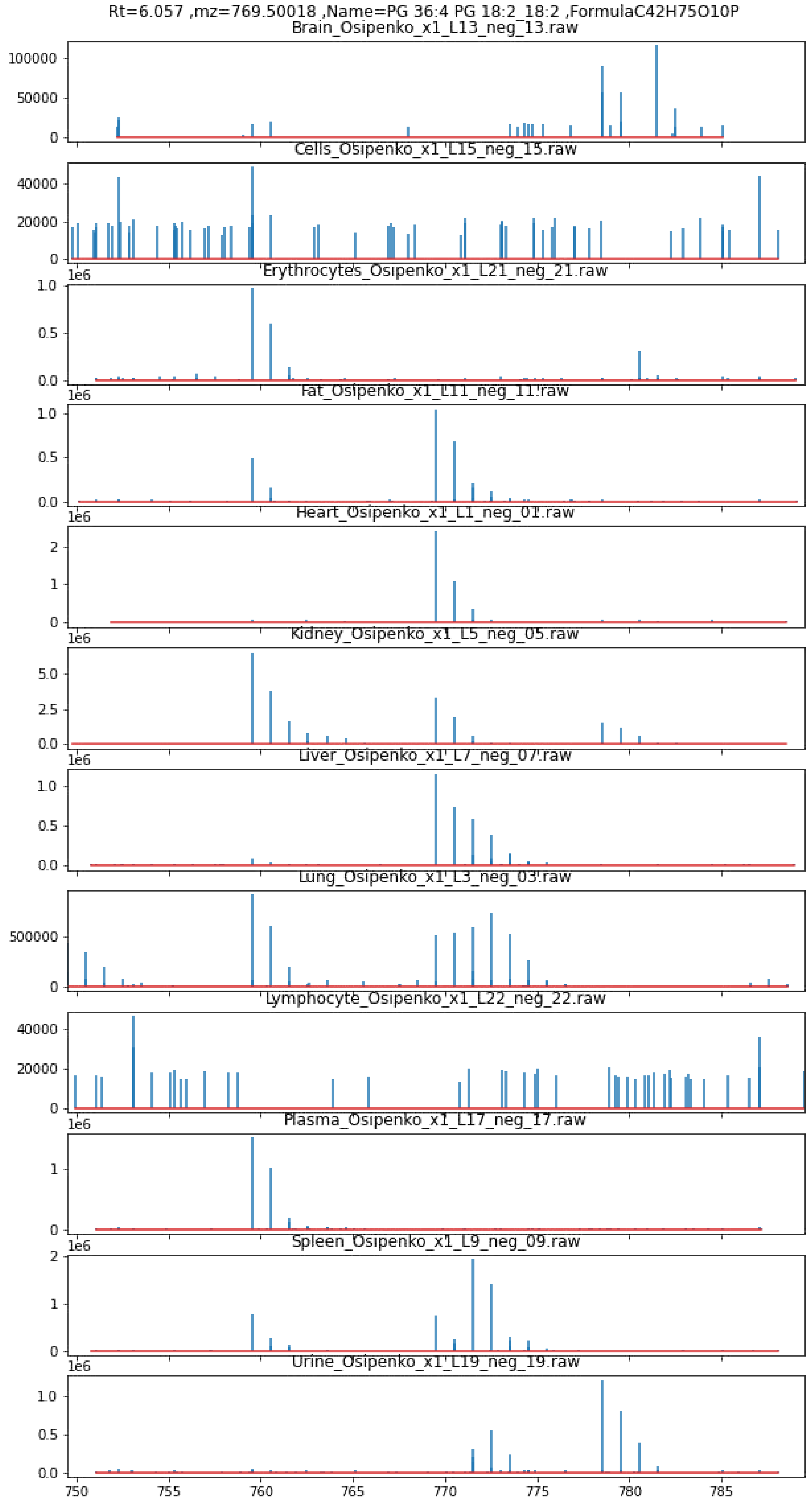
Rt=6.057 ,mz=769.50018 ,Name=PG 36:4 PG 18:2_18:2 ,FormulaC42H75O10P
Brain_Osipenko_x1_L13_neg_13.raw
Cells_Osipenko_x1_L15_neg_15.raw
Erythrocytes_Osipenko_x1_L21_neg_21.raw
Fat_Osipenko_x1_L11_neg_11.raw
Heart_Osipenko_x1_L1_neg_01.raw
Kidney_Osipenko_x1_L5_neg_05.raw
Liver_Osipenko_x1_L7_neg_07.raw
Lung_Osipenko_x1_L3_neg_03.raw
Lymphocyte_Osipenko_x1_L22_neg_22.raw
Plasma_Osipenko_x1_L17_neg_17.raw
Spleen_Osipenko_x1_L9_neg_09.raw
Urine_Osipenko_x1_L19_neg_19.raw

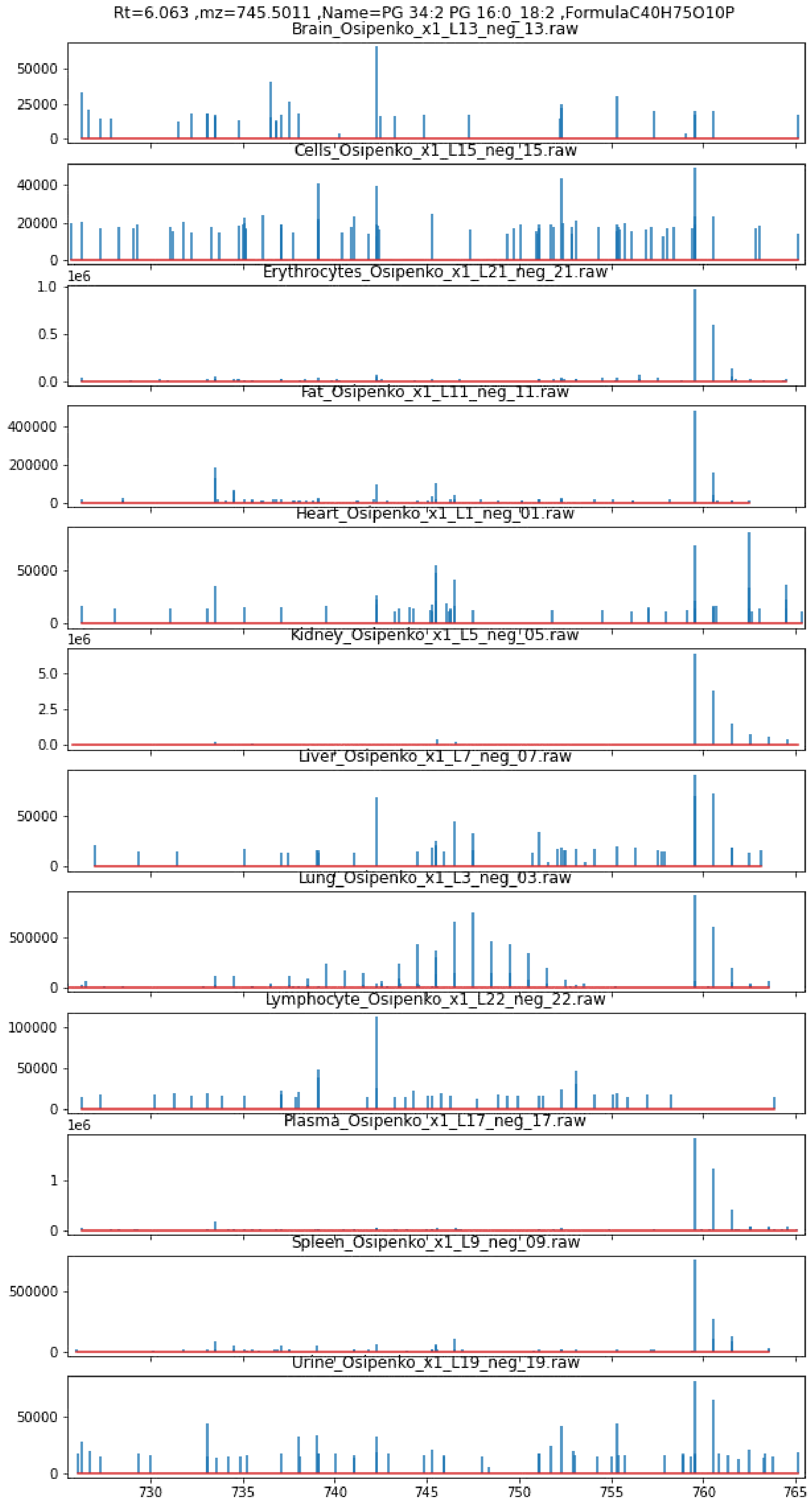

Rt=6.063 ,mz=745.5011 ,Name=PG 34:2 PG 16:0_18:2 ,FormulaC40H75O10P
Brain_Osipenko_x1_L13_neg_13.raw
Cells_Osipenko_x1_L15_neg_15.raw
Erythrocytes_Osipenko_x1_L21_neg_21.raw
Fat_Osipenko_x1_L11_neg_11.raw
Heart_Osipenko_x1_L1_neg_01.raw
Kidney_Osipenko_x1_L5_neg_05.raw
Liver_Osipenko_x1_L7_neg_07.raw
Lung_Osipenko_x1_L3_neg_03.raw
Lymphocyte_Osipenko_x1_L22_neg_22.raw
Plasma_Osipenko_x1_L17_neg_17.raw
Spleen_Osipenko_x1_L9_neg_09.raw
Urine_Osipenko_x1_L19_neg_19.raw
1e6
730
735
740
745
750
755
760
765

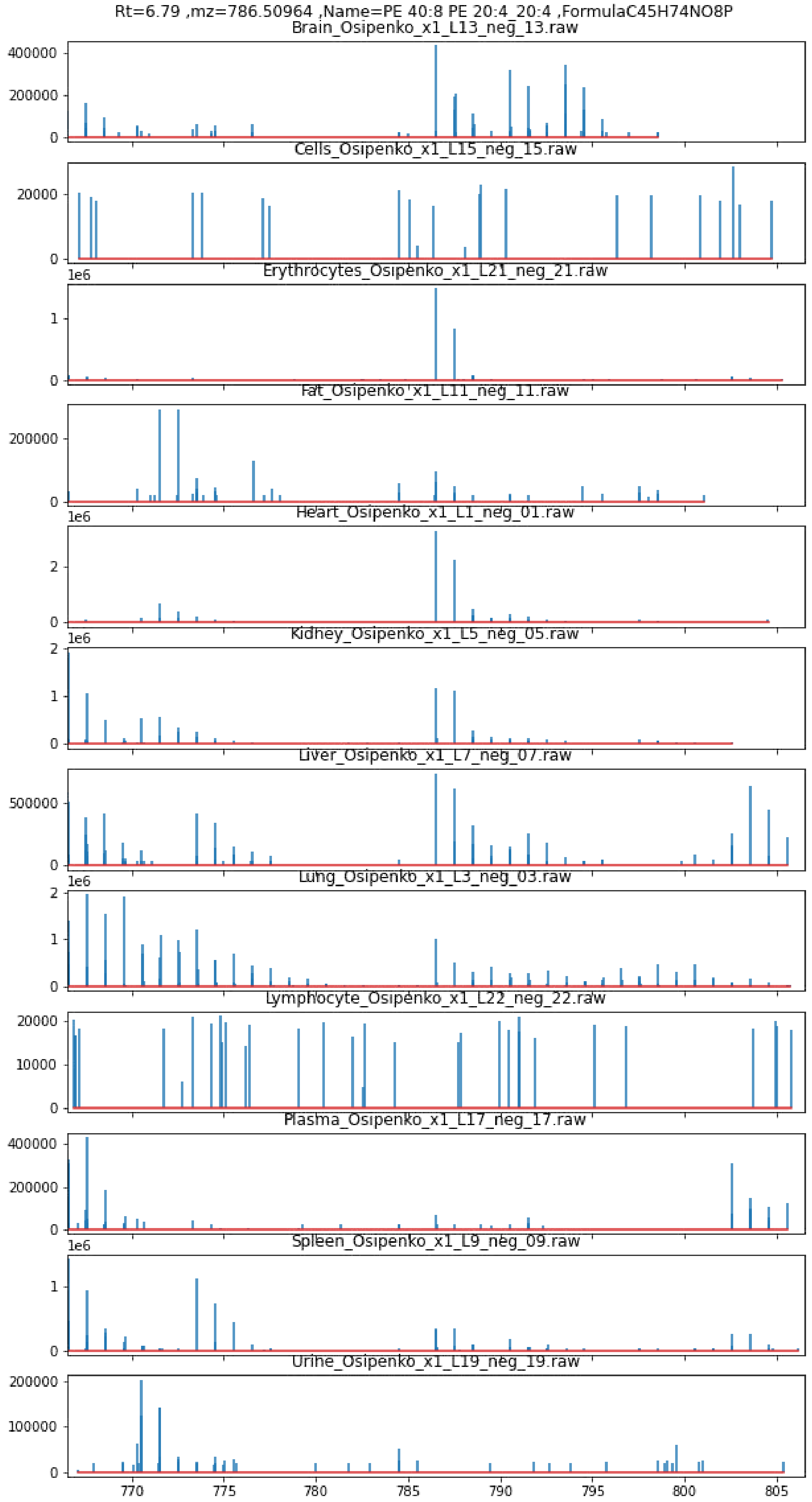
Rt=6.79 ,mz=786.50964 ,Name=PE 40:8 PE 20:4_20:4 ,FormulaC45H74NO8P
Brain_Osipenko_x1_L13_neg_13.raw
Cells_Osipenko_x1_L15_neg_15.raw
Erythrocytes_Osipenko_x1_L21_neg_21.raw
Fat_Osipenko_x1_L11_neg_11.raw
Heart_Osipenko_x1_L1_neg_01.raw
Kidney_Osipenko_x1_L5_neg_05.raw
Liver_Osipenko_x1_L7_neg_07.raw
Lung_Osipenko_x1_L3_neg_03.raw
Lymphocyte_Osipenko_x1_L22_neg_22.raw
Plasma_Osipenko_x1_L17_neg_17.raw
Spleen_Osipenko_x1_L9_neg_09.raw
Urine_Osipenko_x1_L19_neg_19.raw
770
775
780
785
790
795
800
805

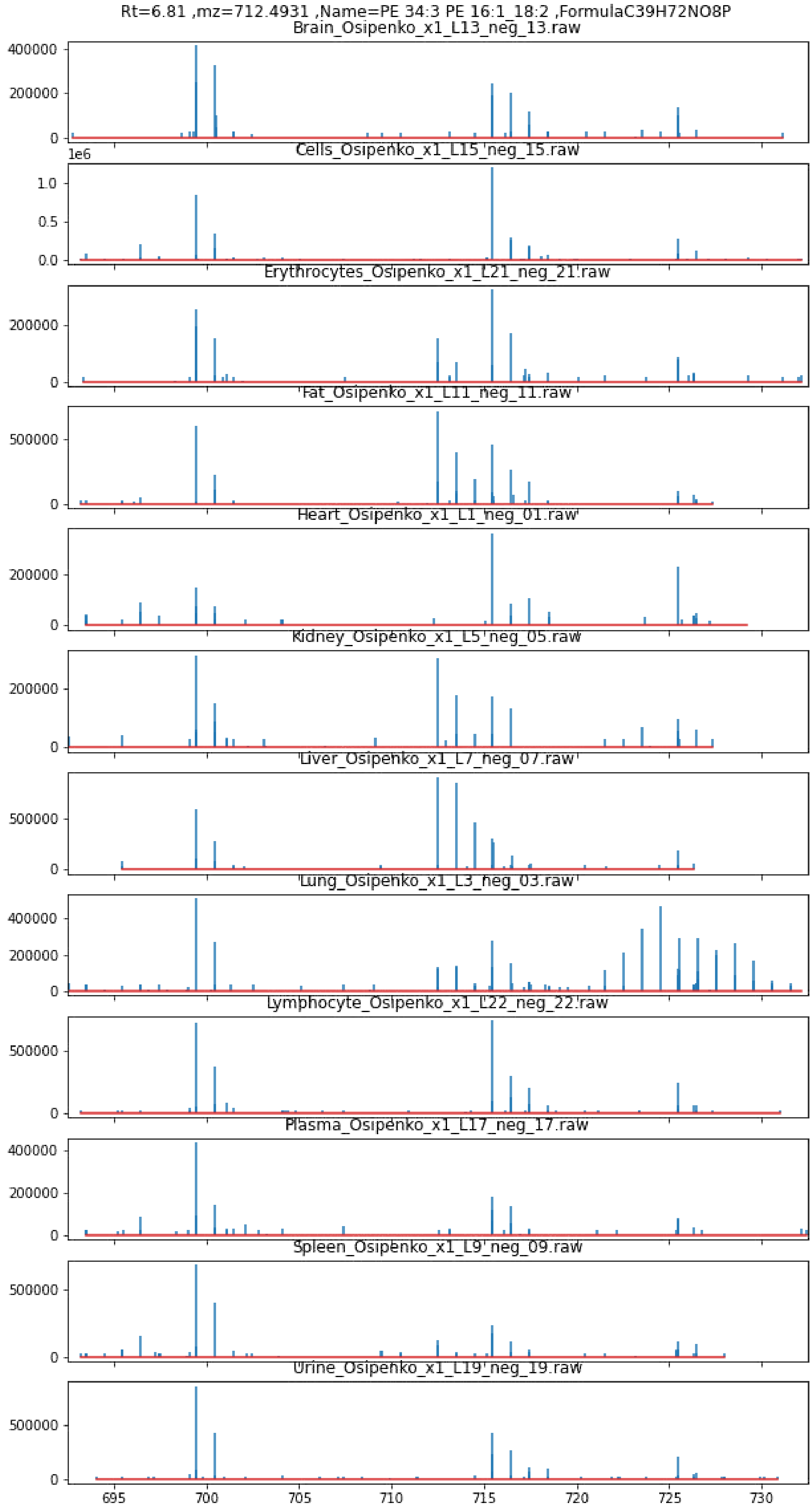
Rt=6.81 ,mz=712.4931 ,Name=PE 34:3 PE 16:1_18:2 ,FormulaC39H72NO8P
Brain_Osipenko_x1_L13_neg_13.raw
Cells_Osipenko_x1_L15_neg_15.raw
Erythrocytes_Osipenko_x1_L21_neg_21.raw
Fat_Osipenko_x1_L11_neg_11.raw
Heart_Osipenko_x1_L1_neg_01.raw
Kidney_Osipenko_x1_L5_neg_05.raw
Liver_Osipenko_x1_L7_neg_07.raw
Lung_Osipenko_x1_L3_neg_03.raw
Lymphocyte_Osipenko_x1_L22_neg_22.raw
Plasma_Osipenko_x1_L17_neg_17.raw
Spleen_Osipenko_x1_L9_neg_09.raw
Urine_Osipenko_x1_L19_neg_19.raw
1e6
695
700
705
710
715
720
725
730

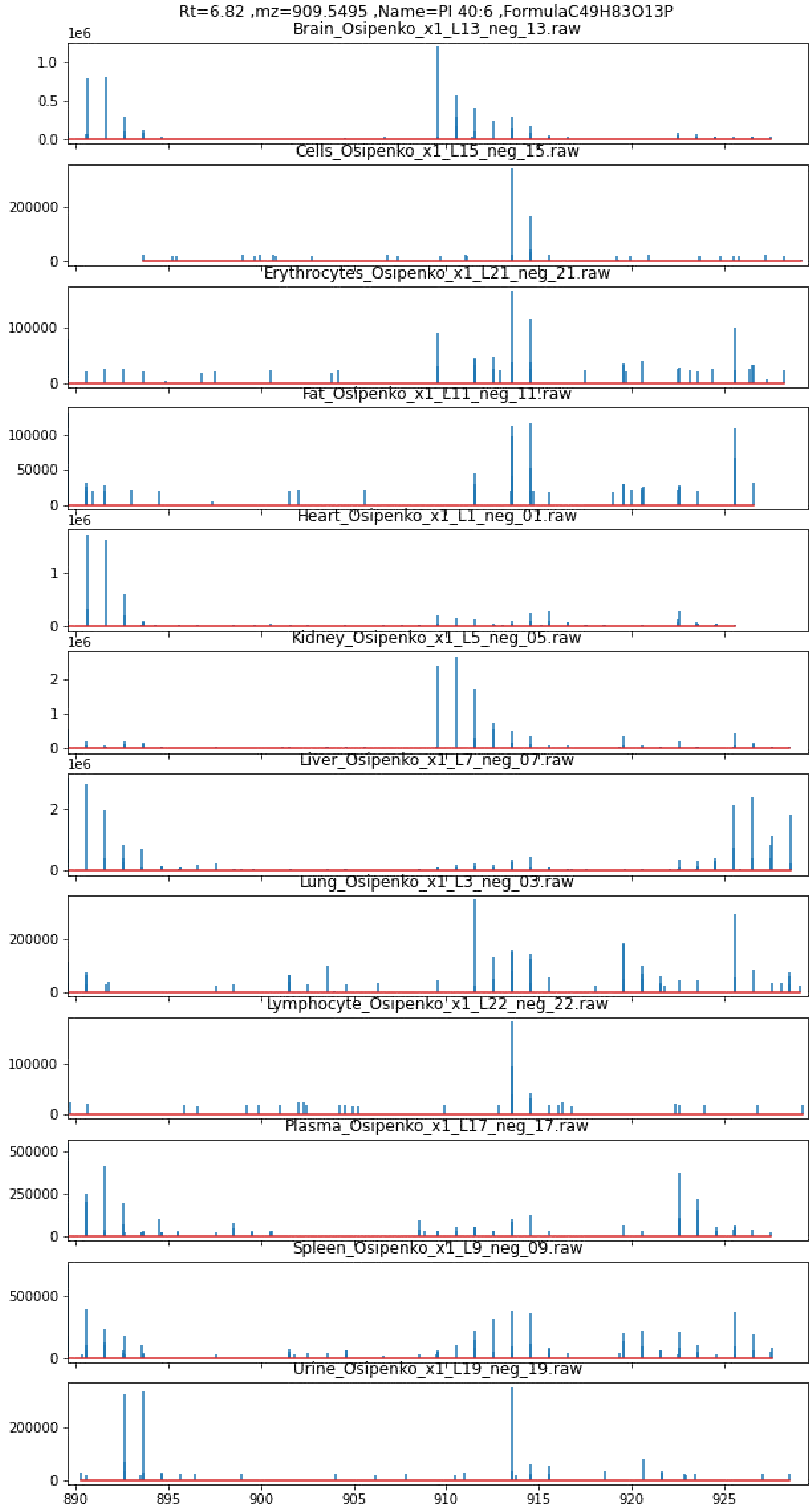

Rt=6.82 ,mz=909.5495 ,Name=PI 40:6 ,FormulaC49H83O13P
Brain_Osipenko_x1_L13_neg_13.raw
Cells_Osipenko_x1_L15_neg_15.raw
Erythrocytes_Osipenko_x1_L21_neg_21.raw
Fat_Osipenko_x1_L11_neg_11.raw
Heart_Osipenko_x1_L1_neg_01.raw
Kidney_Osipenko_x1_L5_neg_05.raw
Liver_Osipenko_x1_L7_neg_07.raw
Lung_Osipenko_x1_L3_neg_03.raw
Lymphocyte_Osipenko_x1_L22_neg_22.raw
Plasma_Osipenko_x1_L17_neg_17.raw
Spleen_Osipenko_x1_L9_neg_09.raw
Urine_Osipenko_x1_L19_neg_19.raw
890
895
900
905
910
915
920
925

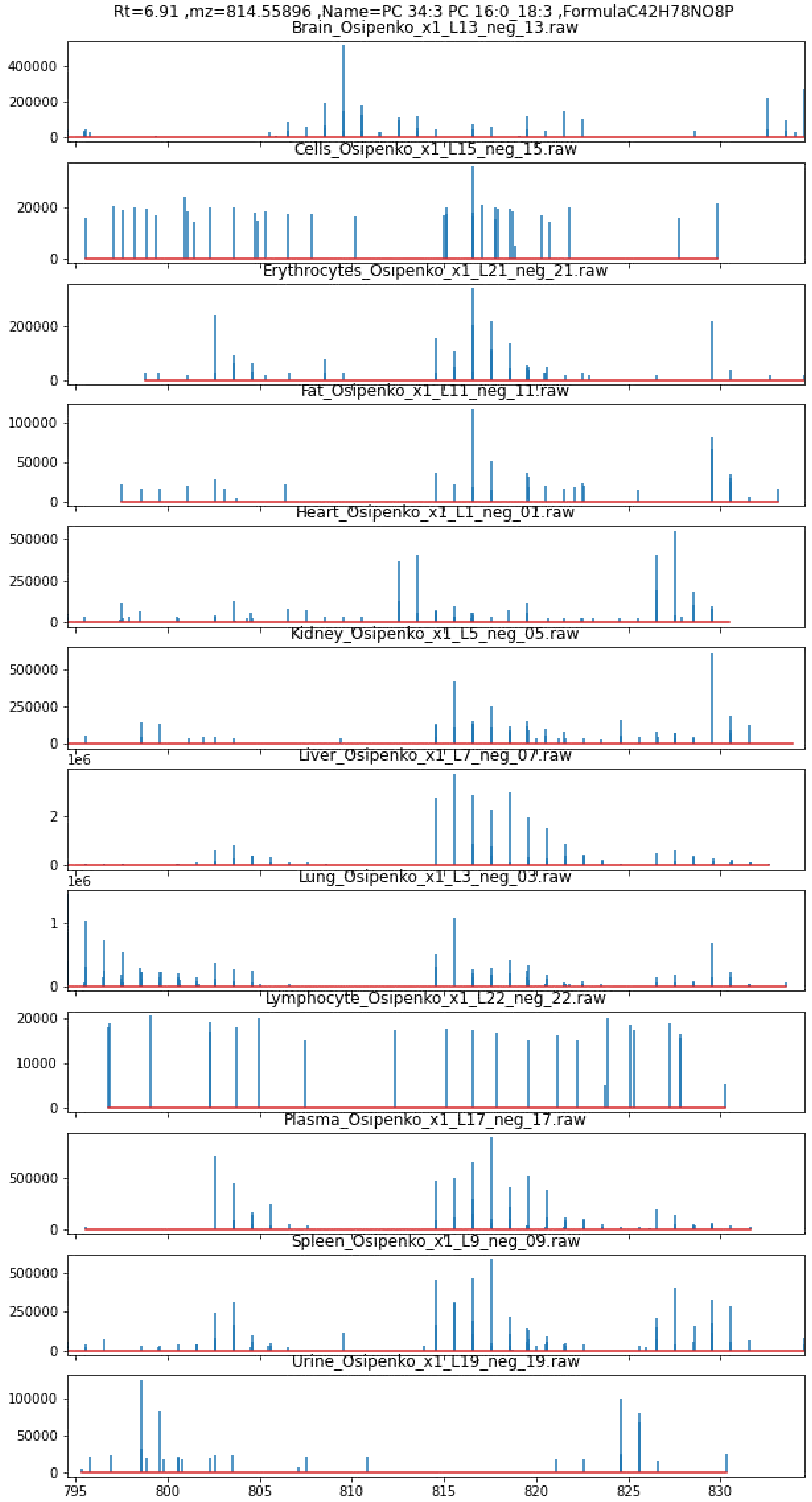

Rt=6.91 ,mz=814.55896 ,Name=PC 34:3 PC 16:0_18:3 ,FormulaC42H78NO8P
Brain_Osipenko_x1_L13_neg_13.raw
Cells_Osipenko_x1_L15_neg_15.raw
Erythrocytes_Osipenko_x1_L21_neg_21.raw
Fat_Osipenko_x1_L11_neg_11.raw
Heart_Osipenko_x1_L1_neg_01.raw
Kidney_Osipenko_x1_L5_neg_05.raw
Liver_Osipenko_x1_L7_neg_07.raw
Lung_Osipenko_x1_L3_neg_03.raw
Lymphocyte_Osipenko_x1_L22_neg_22.raw
Plasma_Osipenko_x1_L17_neg_17.raw
Spleen_Osipenko_x1_L9_neg_09.raw
Urine_Osipenko_x1_L19_neg_19.raw
1e6
795
800
805
810
815
820
825
830

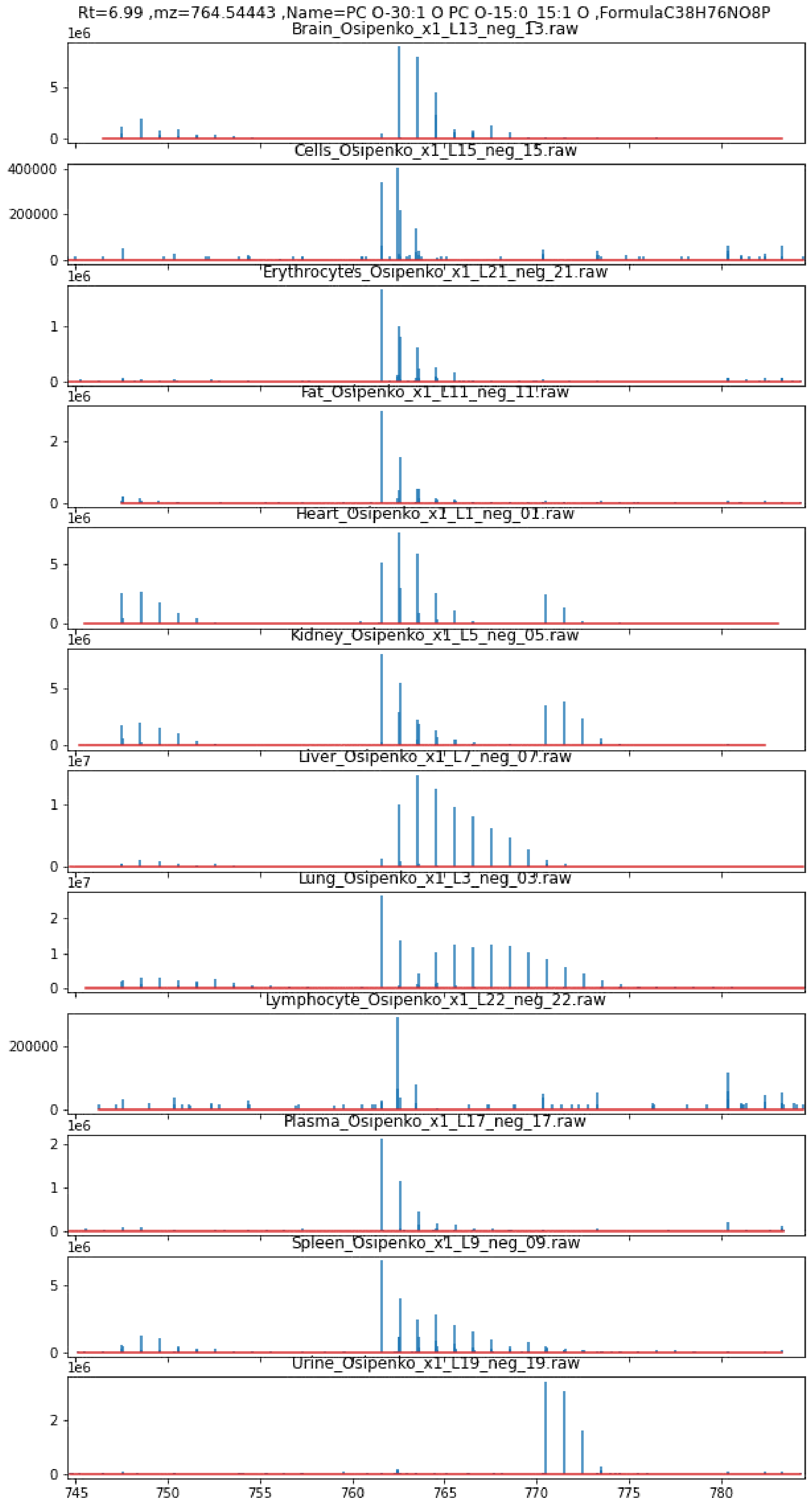

Rt=6.99 ,mz=764.54443 ,Name=PC O-30:1 O PC O-15:0_15:1 O ,FormulaC38H76NO8P
Brain_Osipenko_x1_L13_neg_13.raw
Cells_Osipenko_x1_L15_neg_15.raw
Erythrocytes_Osipenko_x1_L21_neg_21.raw
Fat_Osipenko_x1_L11_neg_11.raw
Heart_Osipenko_x1_L1_neg_01.raw
Kidney_Osipenko_x1_L5_neg_05.raw
Liver_Osipenko_x1_L7_neg_07.raw
Lung_Osipenko_x1_L3_neg_03.raw
Lymphocyte_Osipenko_x1_L22_neg_22.raw
Plasma_Osipenko_x1_L17_neg_17.raw
Spleen_Osipenko_x1_L9_neg_09.raw
Urine_Osipenko_x1_L19_neg_19.raw
745
750
755
760
765
770
775
780

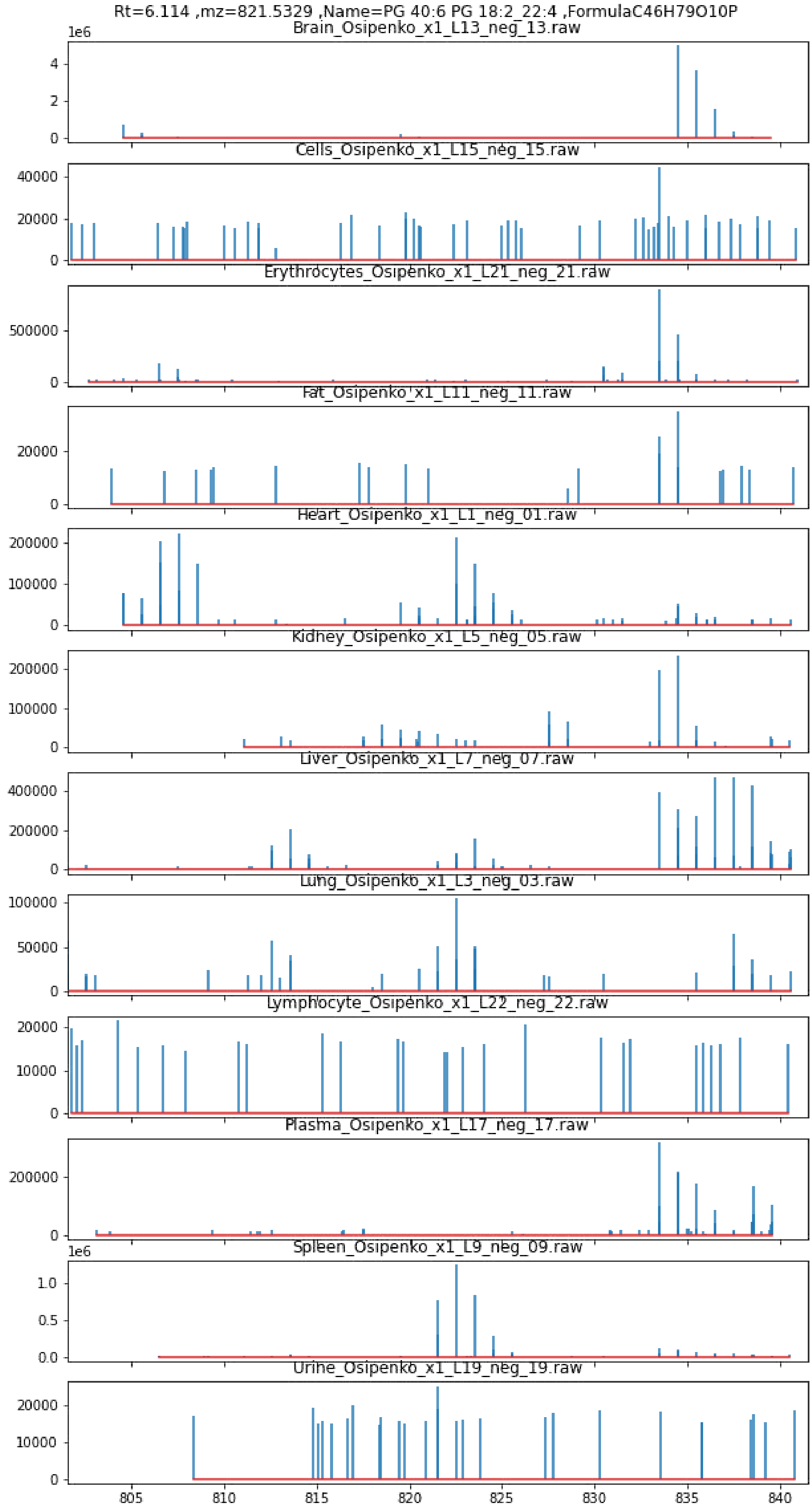
Rt=6.114 ,mz=821.5329 ,Name=PG 40:6 PG 18:2_22:4 ,FormulaC46H79O10P
Brain_Osipenko_x1_L13_neg_13.raw
1e6
Cells_Osipenko_x1_L15_neg_15.raw
Erythrocytes_Osipenko_x1_L21_neg_21.raw
Fat_Osipenko_x1_L11_neg_11.raw
Heart_Osipenko_x1_L1_neg_01.raw
Kidney_Osipenko_x1_L5_neg_05.raw
Liver_Osipenko_x1_L7_neg_07.raw
Lung_Osipenko_x1_L3_neg_03.raw
Lymphocyte_Osipenko_x1_L22_neg_22.raw
Plasma_Osipenko_x1_L17_neg_17.raw
Spleen_Osipenko_x1_L9_neg_09.raw
1e6
Urine_Osipenko_x1_L19_neg_19.raw
805
810
815
820
825
830
835
840

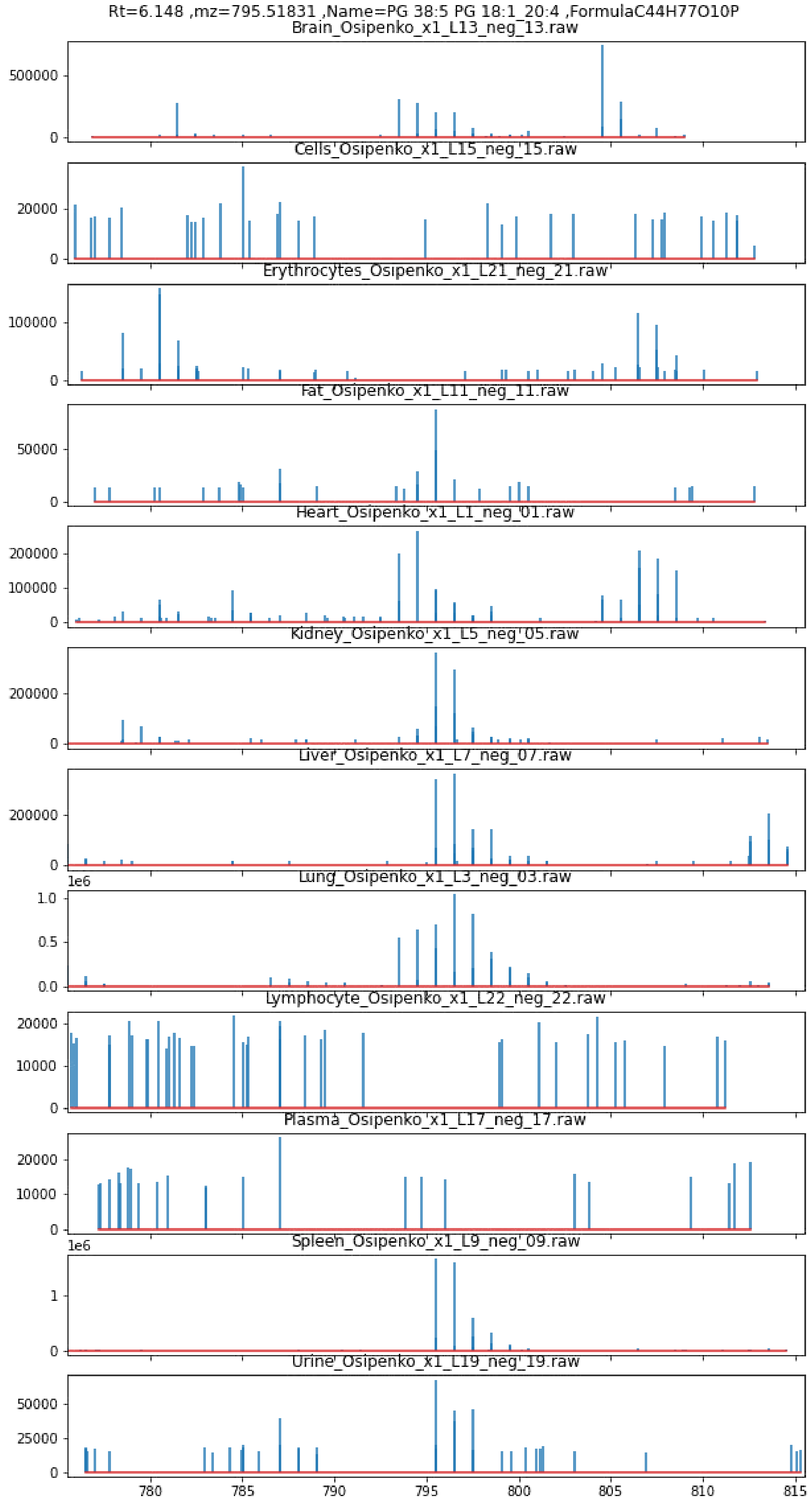

Rt=6.148 ,mz=795.51831 ,Name=PG 38:5 PG 18:1_20:4 ,FormulaC44H77O10P
Brain_Osipenko_x1_L13_neg_13.raw
Cells_Osipenko_x1_L15_neg_15.raw
Erythrocytes_Osipenko_x1_L21_neg_21.raw
Fat_Osipenko_x1_L11_neg_11.raw
Heart_Osipenko_x1_L1_neg_01.raw
Kidney_Osipenko_x1_L5_neg_05.raw
Liver_Osipenko_x1_L7_neg_07.raw
Lung_Osipenko_x1_L3_neg_03.raw
Lymphocyte_Osipenko_x1_L22_neg_22.raw
Plasma_Osipenko_x1_L17_neg_17.raw
Spleen_Osipenko_x1_L9_neg_09.raw
Urine_Osipenko_x1_L19_neg_19.raw
1e6
780
785
790
795
800
805
810
815

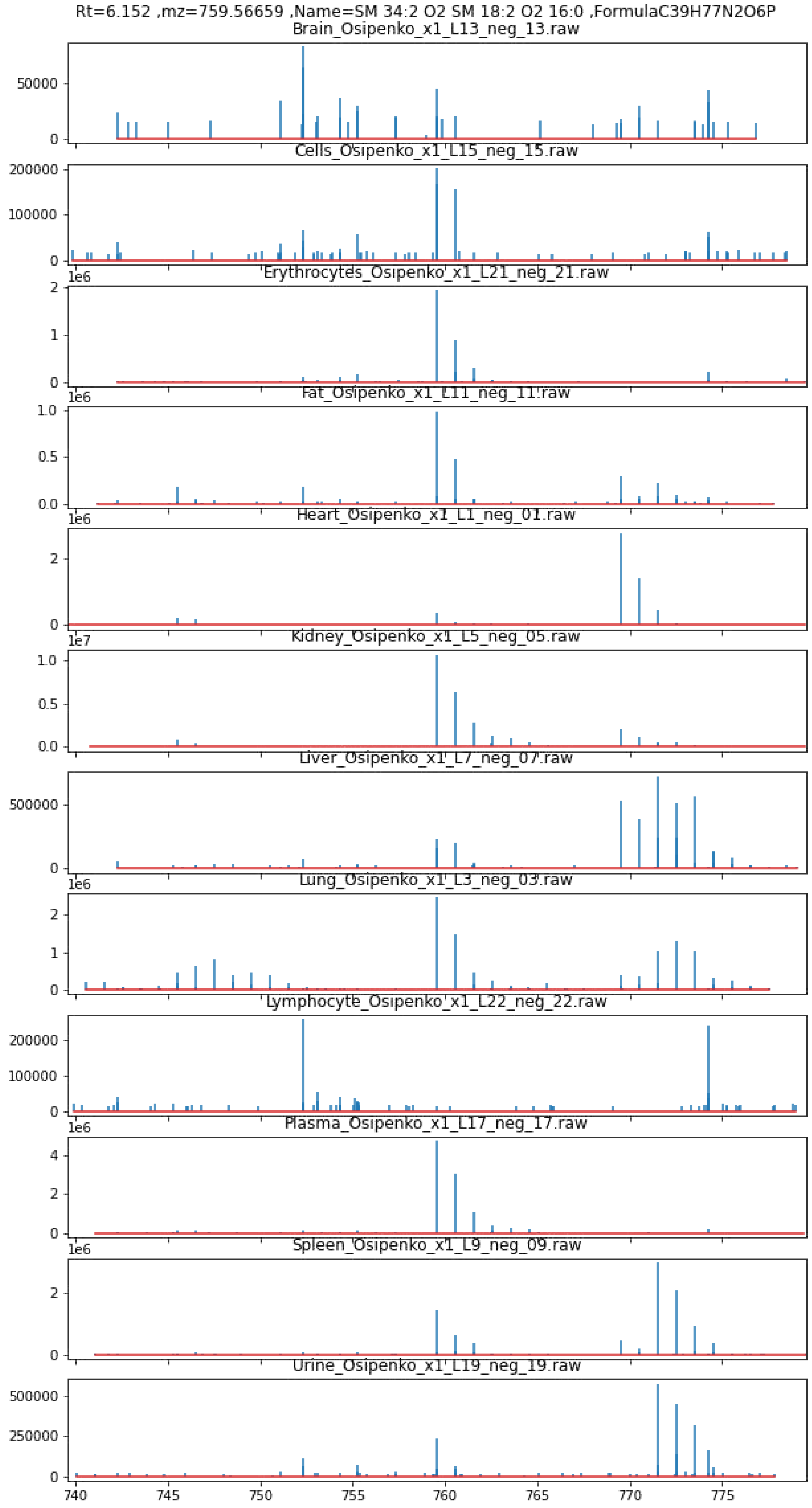
Rt=6.152 ,mz=759.56659 ,Name=SM 34:2 O2 SM 18:2 O2 16:0 ,FormulaC39H77N2O6P
Brain_Osipenko_x1_L13_neg_13.raw
Cells_Osipenko_x1_L15_neg_15.raw
Erythrocytes_Osipenko_x1_L21_neg_21.raw
Fat_Osipenko_x1_L11_neg_11.raw
Heart_Osipenko_x1_L1_neg_01.raw
Kidney_Osipenko_x1_L5_neg_05.raw
Liver_Osipenko_x1_L7_neg_07.raw
Lung_Osipenko_x1_L3_neg_03.raw
Lymphocyte_Osipenko_x1_L22_neg_22.raw
Plasma_Osipenko_x1_L17_neg_17.raw
Spleen_Osipenko_x1_L9_neg_09.raw
Urine_Osipenko_x1_L19_neg_19.raw
1e6
1e7
740
745
750
755
760
765
770
775

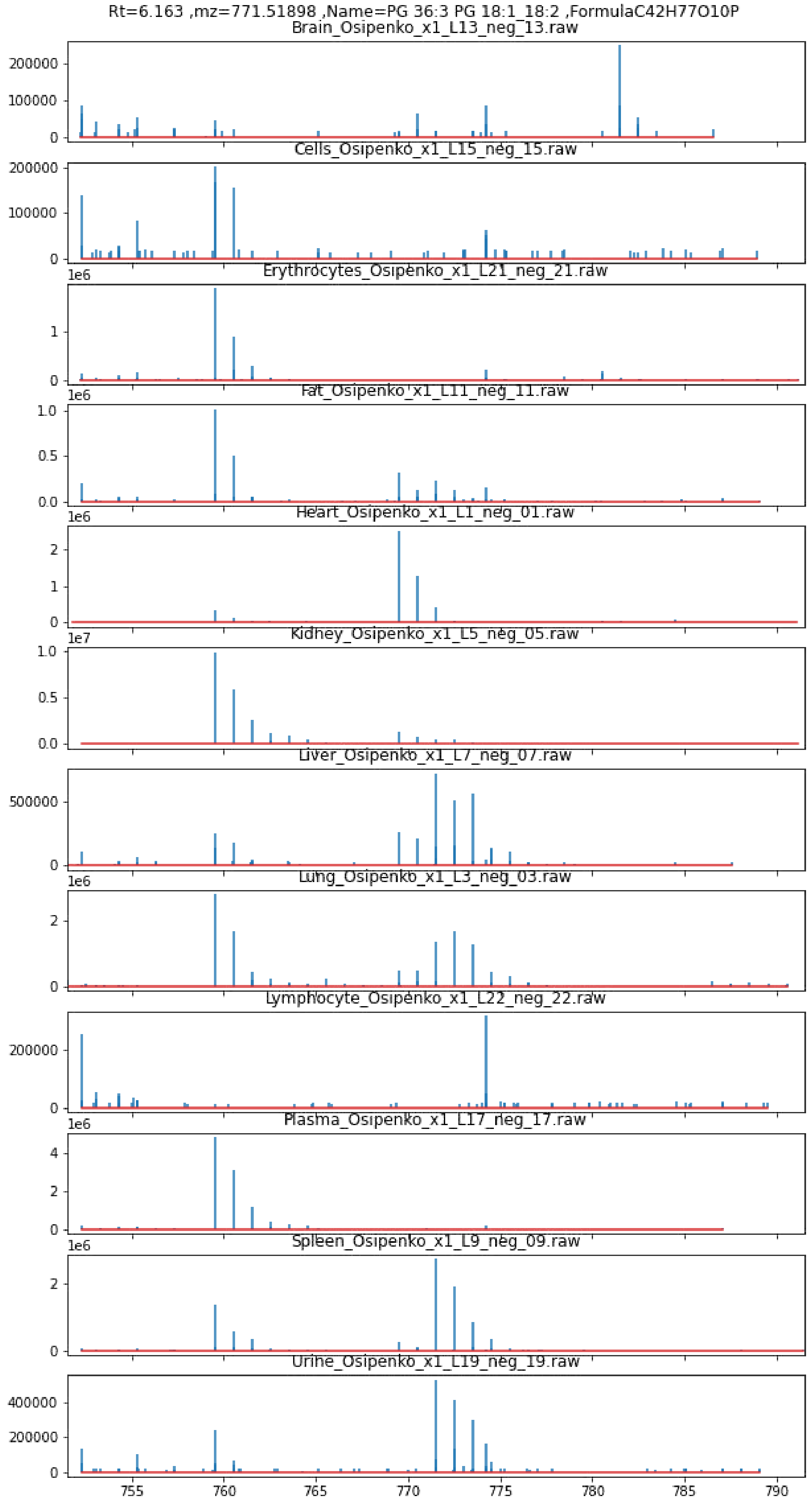

Rt=6.163 ,mz=771.51898 ,Name=PG 36:3 PG 18:1_18:2 ,FormulaC42H77O10P
Brain_Osipenko_x1_L13_neg_13.raw
Cells_Osipenko_x1_L15_neg_15.raw
Erythrocytes_Osipenko_x1_L21_neg_21.raw
Fat_Osipenko_x1_L11_neg_11.raw
Heart_Osipenko_x1_L1_neg_01.raw
Kidney_Osipenko_x1_L5_neg_05.raw
Liver_Osipenko_x1_L7_neg_07.raw
Lung_Osipenko_x1_L3_neg_03.raw
Lymphocyte_Osipenko_x1_L22_neg_22.raw
Plasma_Osipenko_x1_L17_neg_17.raw
Spleen_Osipenko_x1_L9_neg_09.raw
Urine_Osipenko_x1_L19_neg_19.raw
1e6
1e7
755
760
765
770
775
780
785
790

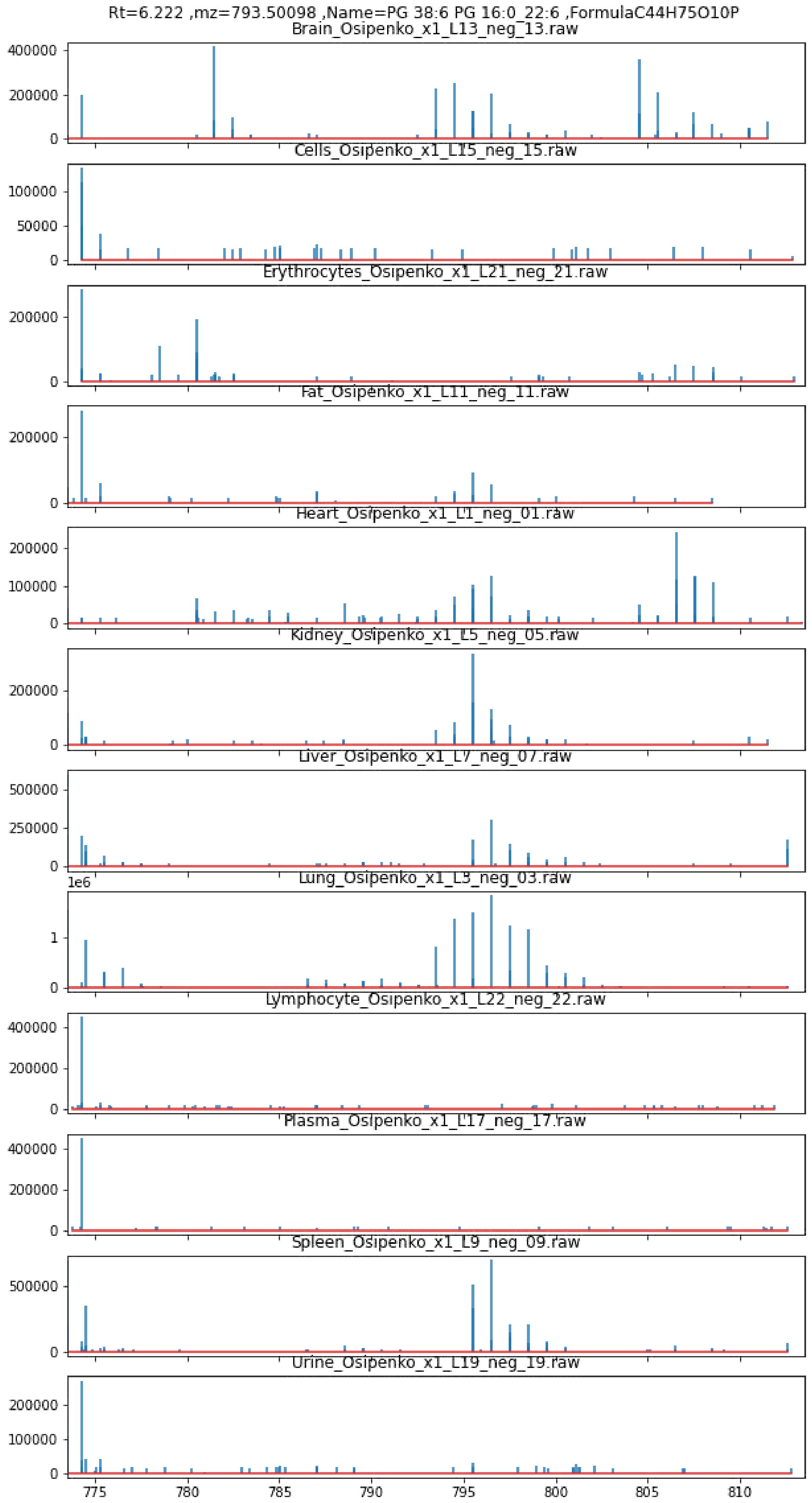

Rt=6.222 ,mz=793.50098 ,Name=PG 38:6 PG 16:0_22:6 ,FormulaC44H75O10P
Brain_Osipenko_x1_L13_neg_13.raw
Cells_Osipenko_x1_L15_neg_15.raw
Erythrocytes_Osipenko_x1_L21_neg_21.raw
Fat_Osipenko_x1_L11_neg_11.raw
Heart_Osipenko_x1_L1_neg_01.raw
Kidney_Osipenko_x1_L5_neg_05.raw
Liver_Osipenko_x1_L7_neg_07.raw
Lung_Osipenko_x1_L3_neg_03.raw
Lymphocyte_Osipenko_x1_L22_neg_22.raw
Plasma_Osipenko_x1_L17_neg_17.raw
Spleen_Osipenko_x1_L9_neg_09.raw
Urine_Osipenko_x1_L19_neg_19.raw
1e6
775
780
785
790
795
800
805
810

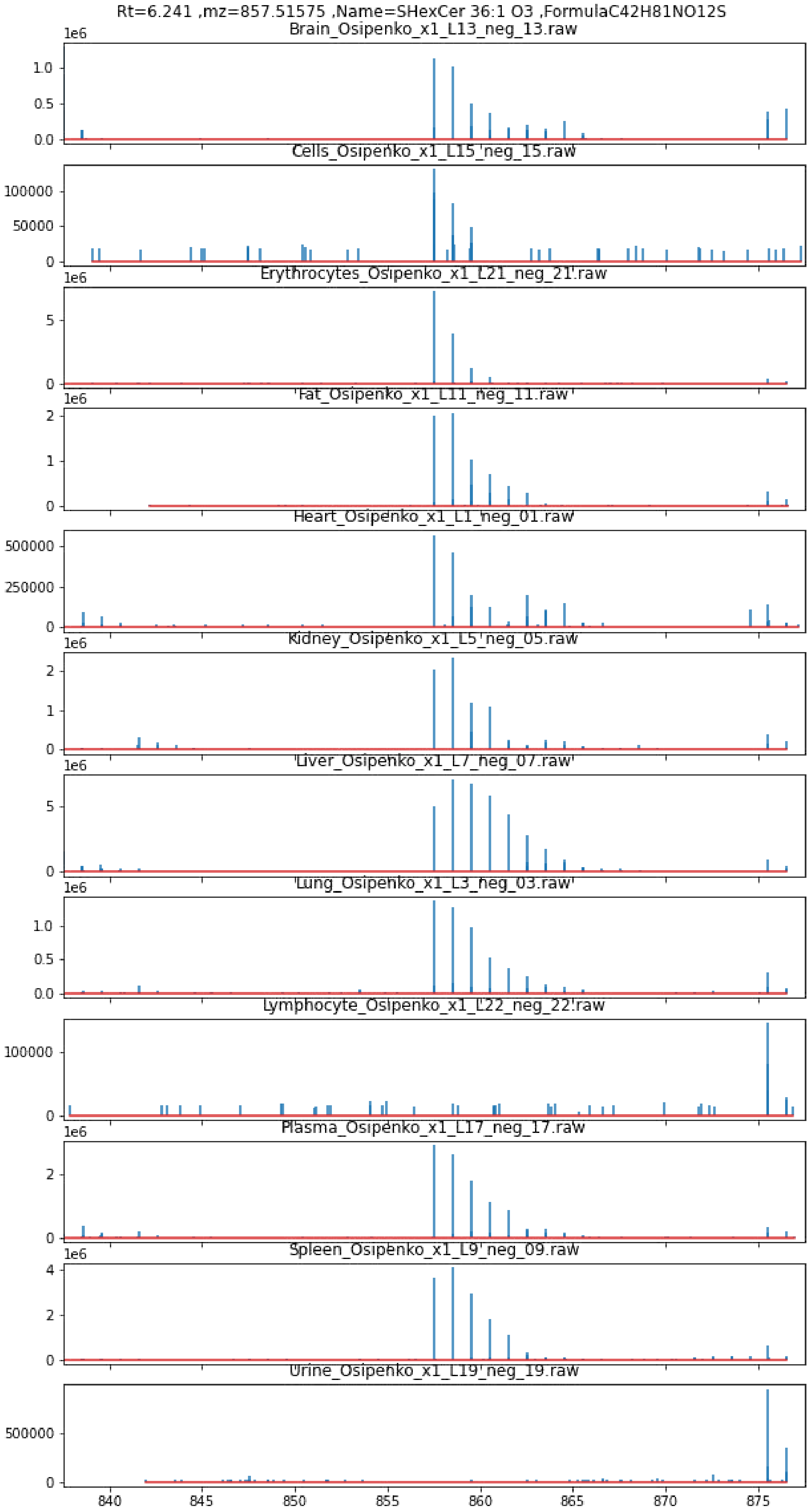
Rt=6.241 ,mz=857.51575 ,Name=SHexCer 36:1 O3 ,FormulaC42H81NO12S
Brain_Osipenko_x1_L13_neg_13.raw
Cells_Osipenko_x1_L15_neg_15.raw
Erythrocytes_Osipenko_x1_L21_neg_21.raw
Fat_Osipenko_x1_L11_neg_11.raw
Heart_Osipenko_x1_L1_neg_01.raw
Kidney_Osipenko_x1_L5_neg_05.raw
Liver_Osipenko_x1_L7_neg_07.raw
Lung_Osipenko_x1_L3_neg_03.raw
Lymphocyte_Osipenko_x1_L22_neg_22.raw
Plasma_Osipenko_x1_L17_neg_17.raw
Spleen_Osipenko_x1_L9_neg_09.raw
Urine_Osipenko_x1_L19_neg_19.raw
840
845
850
855
860
865
870
875

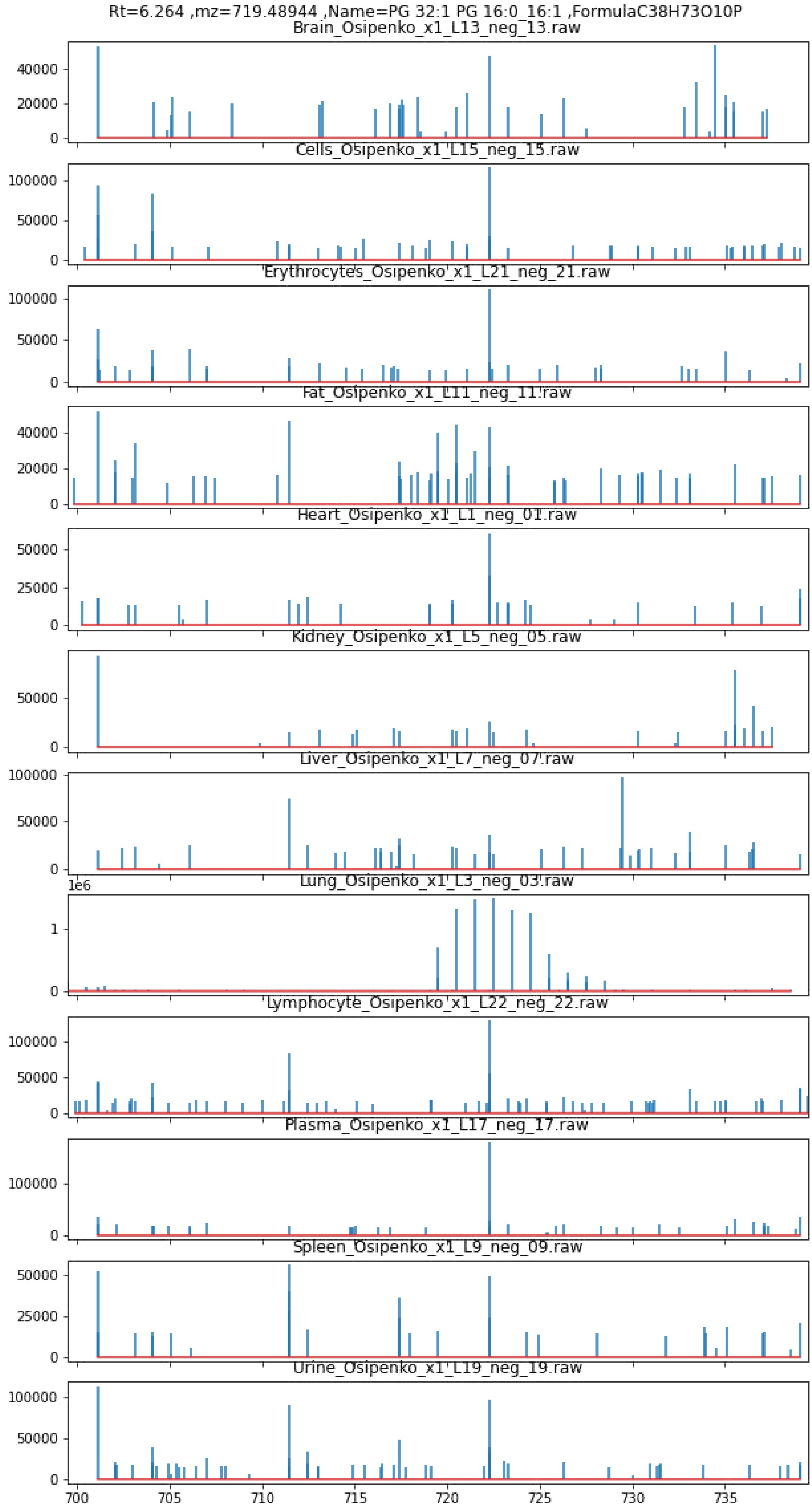

Rt=6.264 ,mz=719.48944 ,Name=PG 32:1 PG 16:0_16:1 ,FormulaC38H73O10P
Brain_Osipenko_x1_L13_neg_13.raw
Cells_Osipenko_x1_L15_neg_15.raw
Erythrocytes_Osipenko_x1_L21_neg_21.raw
Fat_Osipenko_x1_L11_neg_11.raw
Heart_Osipenko_x1_L1_neg_01.raw
Kidney_Osipenko_x1_L5_neg_05.raw
Liver_Osipenko_x1_L7_neg_07.raw
Lung_Osipenko_x1_L3_neg_03.raw
1e6
Lymphocyte_Osipenko_x1_L22_neg_22.raw
Plasma_Osipenko_x1_L17_neg_17.raw
Spleen_Osipenko_x1_L9_neg_09.raw
Urine_Osipenko_x1_L19_neg_19.raw
700
705
710
715
720
725
730
735

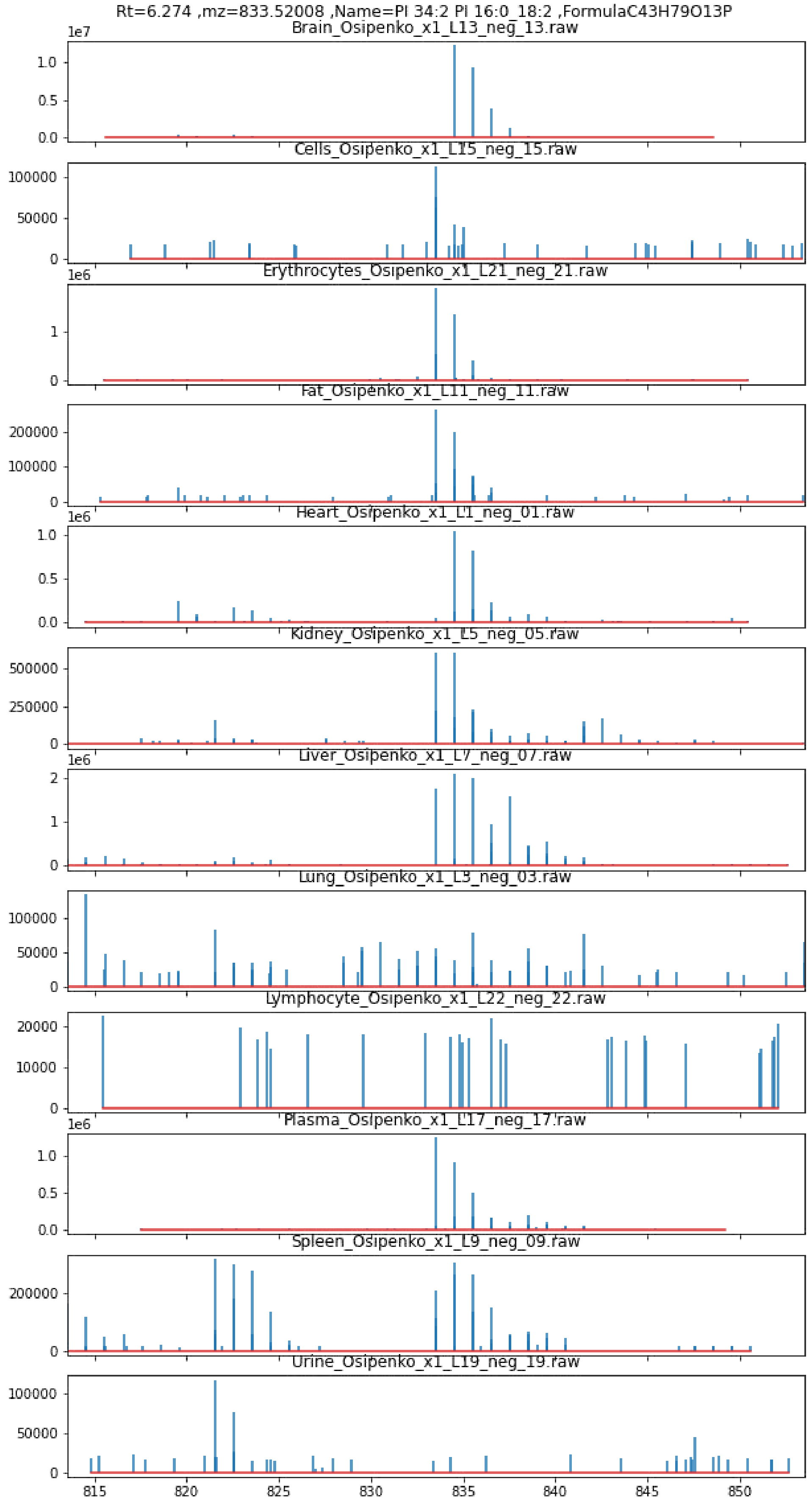

Rt=6.274 ,mz=833.52008 ,Name=PI 34:2 PI 16:0_18:2 ,FormulaC43H79O13P
Brain_Osipenko_x1_L13_neg_13.raw
Cells_Osipenko_x1_L15_neg_15.raw
Erythrocytes_Osipenko_x1_L21_neg_21.raw
Fat_Osipenko_x1_L11_neg_11.raw
Heart_Osipenko_x1_L1_neg_01.raw
Kidney_Osipenko_x1_L5_neg_05.raw
Liver_Osipenko_x1_L7_neg_07.raw
Lung_Osipenko_x1_L3_neg_03.raw
Lymphocyte_Osipenko_x1_L22_neg_22.raw
Plasma_Osipenko_x1_L17_neg_17.raw
Spleen_Osipenko_x1_L9_neg_09.raw
Urine_Osipenko_x1_L19_neg_19.raw
815
820
825
830
835
840
845
850

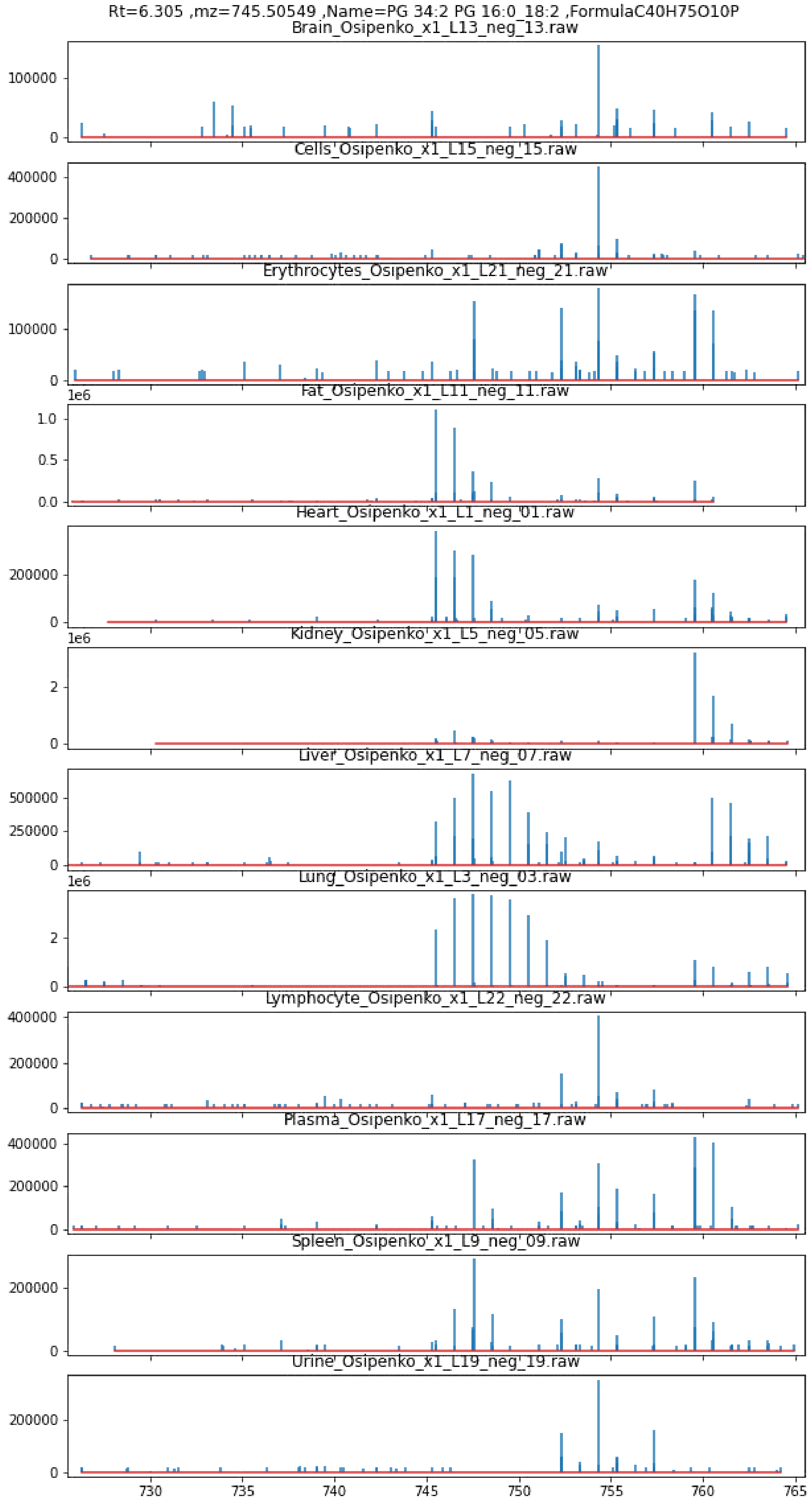
Rt=6.305 ,mz=745.50549 ,Name=PG 34:2 PG 16:0_18:2 ,FormulaC40H75O10P
Brain_Osipenko_x1_L13_neg_13.raw
Cells_Osipenko_x1_L15_neg_15.raw
Erythrocytes_Osipenko_x1_L21_neg_21.raw
Fat_Osipenko_x1_L11_neg_11.raw
Heart_Osipenko_x1_L1_neg_01.raw
Kidney_Osipenko_x1_L5_neg_05.raw
Liver_Osipenko_x1_L7_neg_07.raw
Lung_Osipenko_x1_L3_neg_03.raw
Lymphocyte_Osipenko_x1_L22_neg_22.raw
Plasma_Osipenko_x1_L17_neg_17.raw
Spleen_Osipenko_x1_L9_neg_09.raw
Urine_Osipenko_x1_L19_neg_19.raw
1e6
730
735
740
745
750
755
760
765

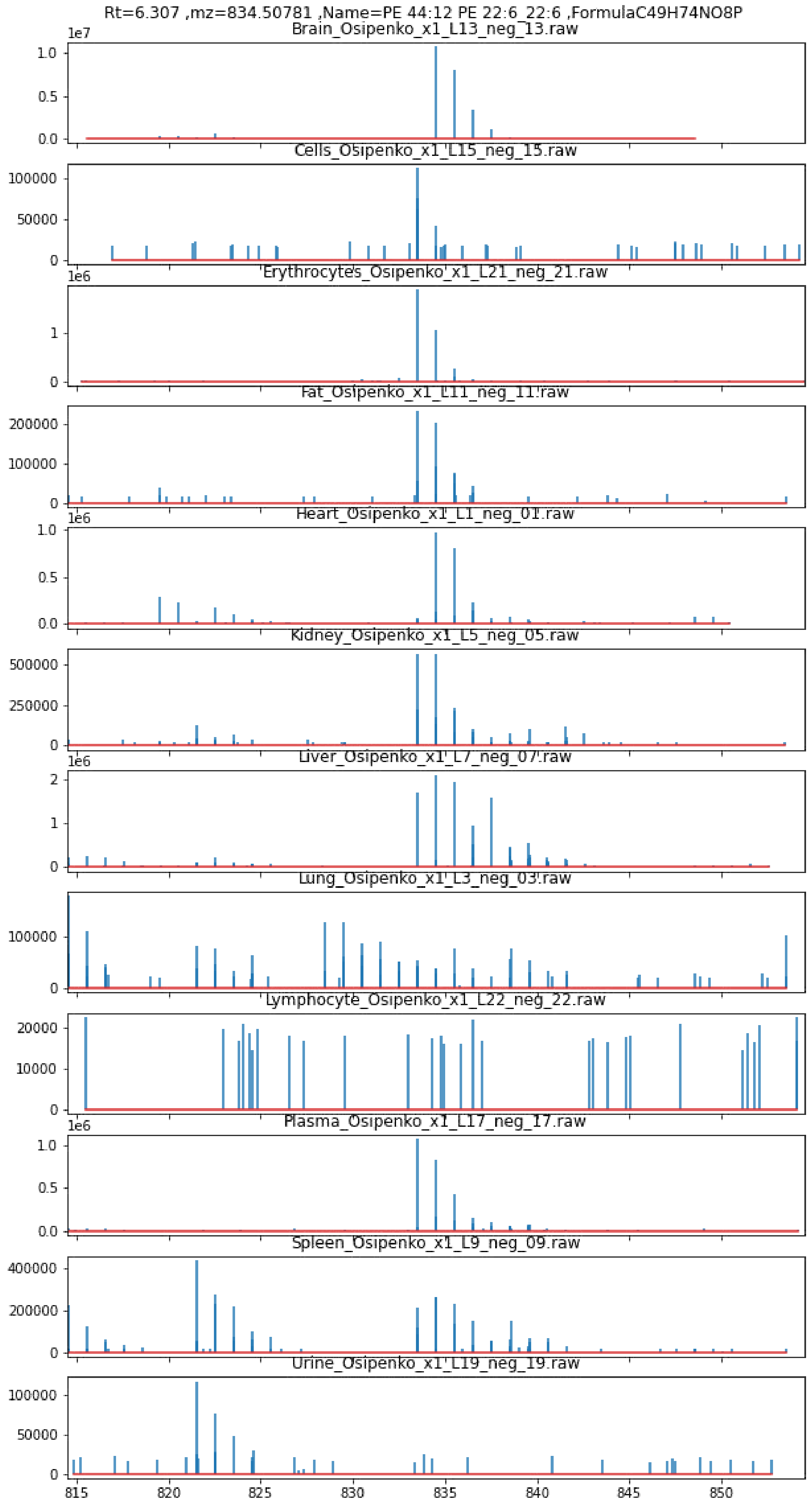

Rt=6.307 ,mz=834.50781 ,Name=PE 44:12 PE 22:6_22:6 ,FormulaC49H74NO8P
Brain_Osipenko_x1_L13_neg_13.raw
Cells_Osipenko_x1_L15_neg_15.raw
Erythrocytes_Osipenko_x1_L21_neg_21.raw
Fat_Osipenko_x1_L11_neg_11.raw
Heart_Osipenko_x1_L1_neg_01.raw
Kidney_Osipenko_x1_L5_neg_05.raw
Liver_Osipenko_x1_L7_neg_07.raw
Lung_Osipenko_x1_L3_neg_03.raw
Lymphocyte_Osipenko_x1_L22_neg_22.raw
Plasma_Osipenko_x1_L17_neg_17.raw
Spleen_Osipenko_x1_L9_neg_09.raw
Urine_Osipenko_x1_L19_neg_19.raw
815
820
825
830
835
840
845
850

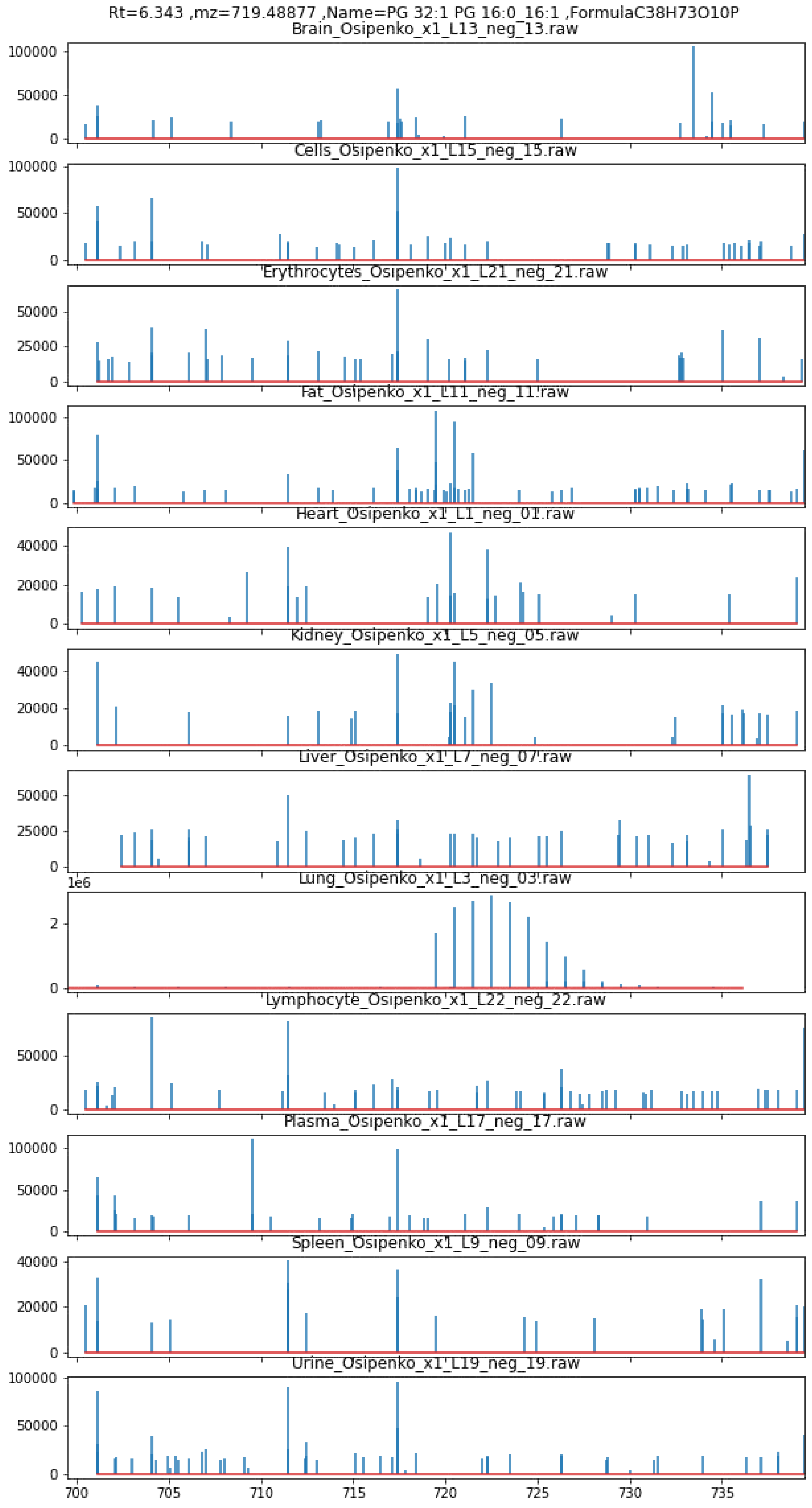

Rt=6.343 ,mz=719.48877 ,Name=PG 32:1 PG 16:0_16:1 ,FormulaC38H73O10P
Brain_Osipenko_x1_L13_neg_13.raw
Cells_Osipenko_x1_L15_neg_15.raw
Erythrocytes_Osipenko_x1_L21_neg_21.raw
Fat_Osipenko_x1_L11_neg_11.raw
Heart_Osipenko_x1_L1_neg_01.raw
Kidney_Osipenko_x1_L5_neg_05.raw
Liver_Osipenko_x1_L7_neg_07.raw
Lung_Osipenko_x1_L3_neg_03.raw
1e6
Lymphocyte_Osipenko_x1_L22_neg_22.raw
Plasma_Osipenko_x1_L17_neg_17.raw
Spleen_Osipenko_x1_L9_neg_09.raw
Urine_Osipenko_x1_L19_neg_19.raw
700
705
710
715
720
725
730
735

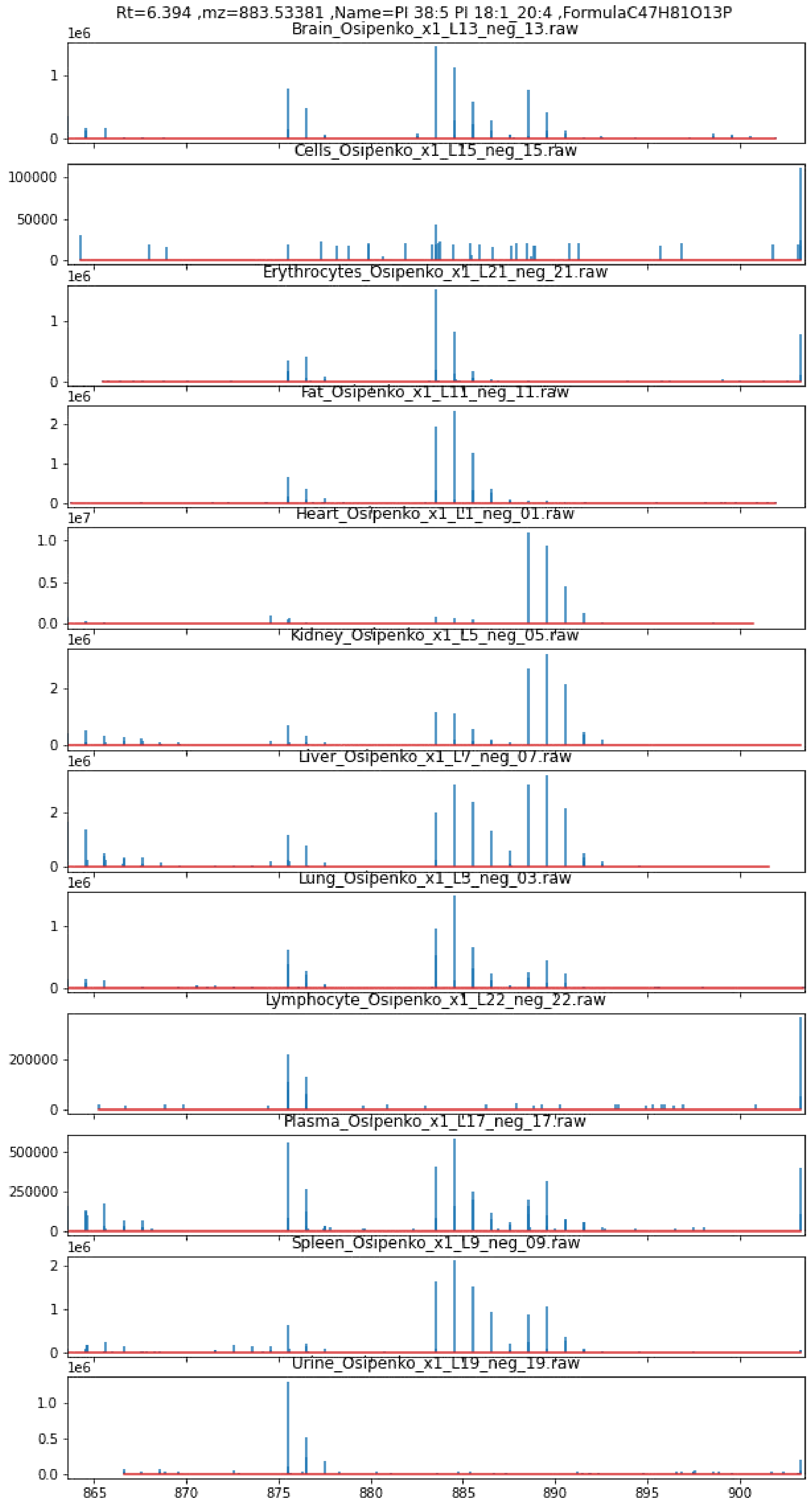
Rt=6.394 ,mz=883.53381 ,Name=PI 38:5 PI 18:1_20:4 ,FormulaC47H81O13P
Brain_Osipenko_x1_L13_neg_13.raw
Cells_Osipenko_x1_L15_neg_15.raw
Erythrocytes_Osipenko_x1_L21_neg_21.raw
Fat_Osipenko_x1_L11_neg_11.raw
Heart_Osipenko_x1_L1_neg_01.raw
Kidney_Osipenko_x1_L5_neg_05.raw
Liver_Osipenko_x1_L7_neg_07.raw
Lung_Osipenko_x1_L3_neg_03.raw
Lymphocyte_Osipenko_x1_L22_neg_22.raw
Plasma_Osipenko_x1_L17_neg_17.raw
Spleen_Osipenko_x1_L9_neg_09.raw
Urine_Osipenko_x1_L19_neg_19.raw
865
870
875
880
885
890
895
900

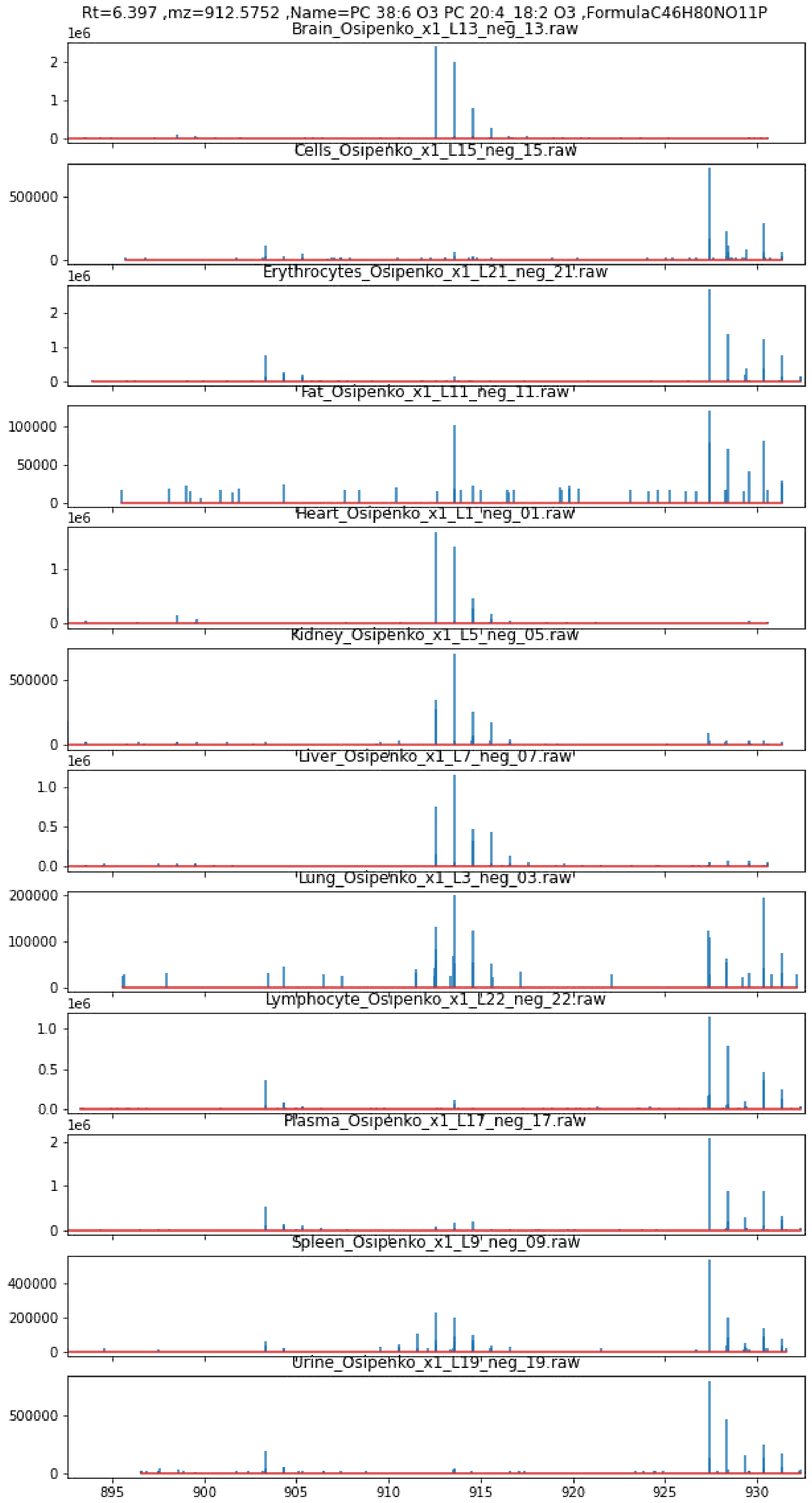

Rt=6.397 ,mz=912.5752 ,Name=PC 38:6 O3 PC 20:4_18:2 O3 ,FormulaC46H80NO11P
Brain_Osipenko_x1_L13_neg_13.raw
Cells_Osipenko_x1_L15_neg_15.raw
Erythrocytes_Osipenko_x1_L21_neg_21.raw
Fat_Osipenko_x1_L11_neg_11.raw
Heart_Osipenko_x1_L1_neg_01.raw
Kidney_Osipenko_x1_L5_neg_05.raw
Liver_Osipenko_x1_L7_neg_07.raw
Lung_Osipenko_x1_L3_neg_03.raw
Lymphocyte_Osipenko_x1_L22_neg_22.raw
Plasma_Osipenko_x1_L17_neg_17.raw
Spleen_Osipenko_x1_L9_neg_09.raw
Urine_Osipenko_x1_L19_neg_19.raw
1e6
895
900
905
910
915
920
925
930

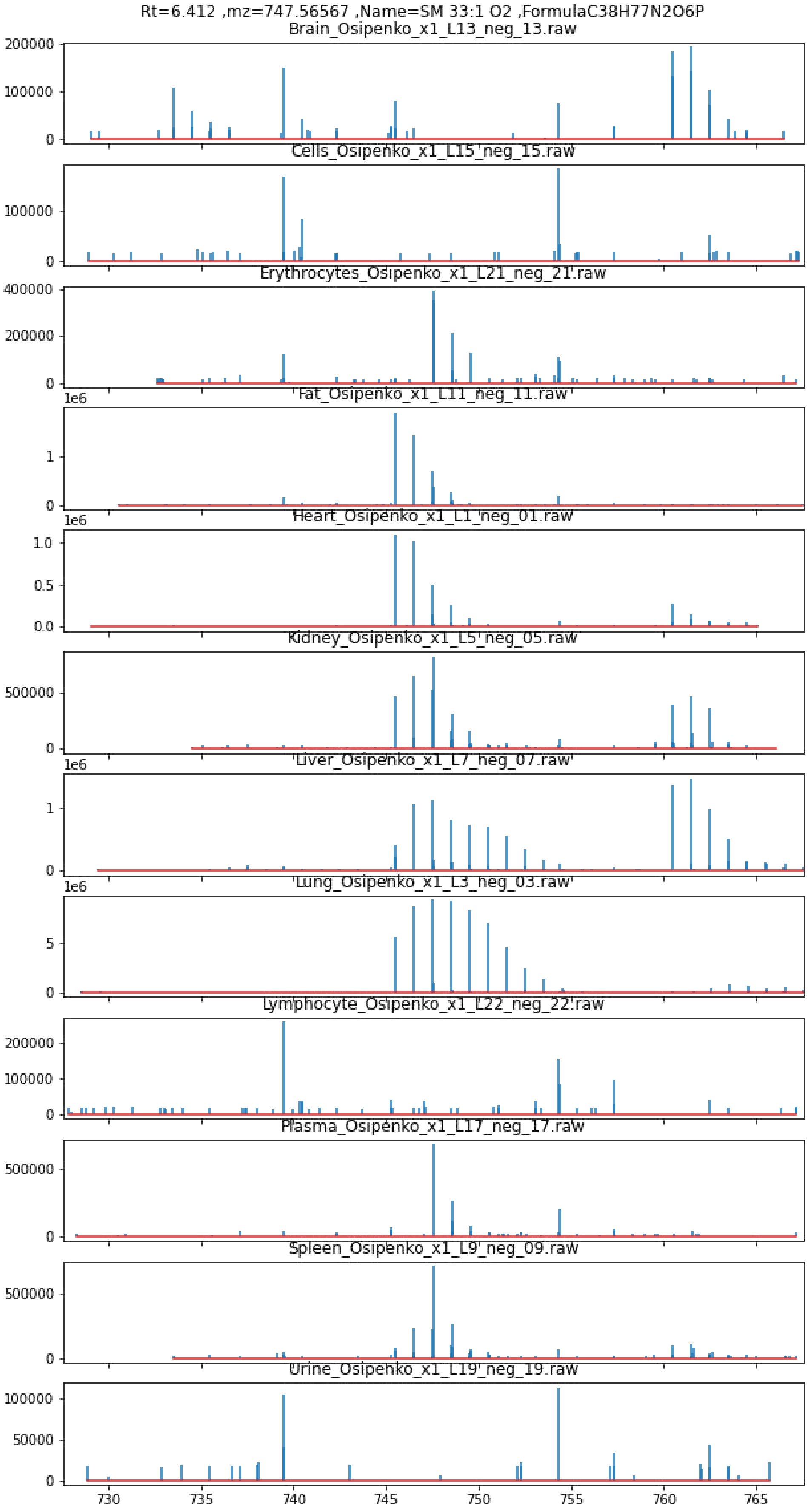
Rt=6.412 ,mz=747.56567 ,Name=SM 33:1 O2 ,FormulaC38H77N2O6P
Brain_Osipenko_x1_L13_neg_13.raw
200000
100000
0
Cells_Osipenko_x1_L15_neg_15.raw
100000
0
Erythrocytes_Osipenko_x1_L21_neg_21.raw
400000
200000
0
1e6
Fat_Osipenko_x1_L11_neg_11.raw
1
0
1e6
Heart_Osipenko_x1_L1_neg_01.raw
1.0
0.5
0.0
Kidney_Osipenko_x1_L5_neg_05.raw
500000
0
1e6
Liver_Osipenko_x1_L7_neg_07.raw
1
0
1e6
Lung_Osipenko_x1_L3_neg_03.raw
5
0
Lymphocyte_Osipenko_x1_L22_neg_22.raw
200000
100000
0
Plasma_Osipenko_x1_L17_neg_17.raw
500000
0
Spleen_Osipenko_x1_L9_neg_09.raw
500000
0
Urine_Osipenko_x1_L19_neg_19.raw
100000
50000
0
730
735
740
745
750
755
760
765

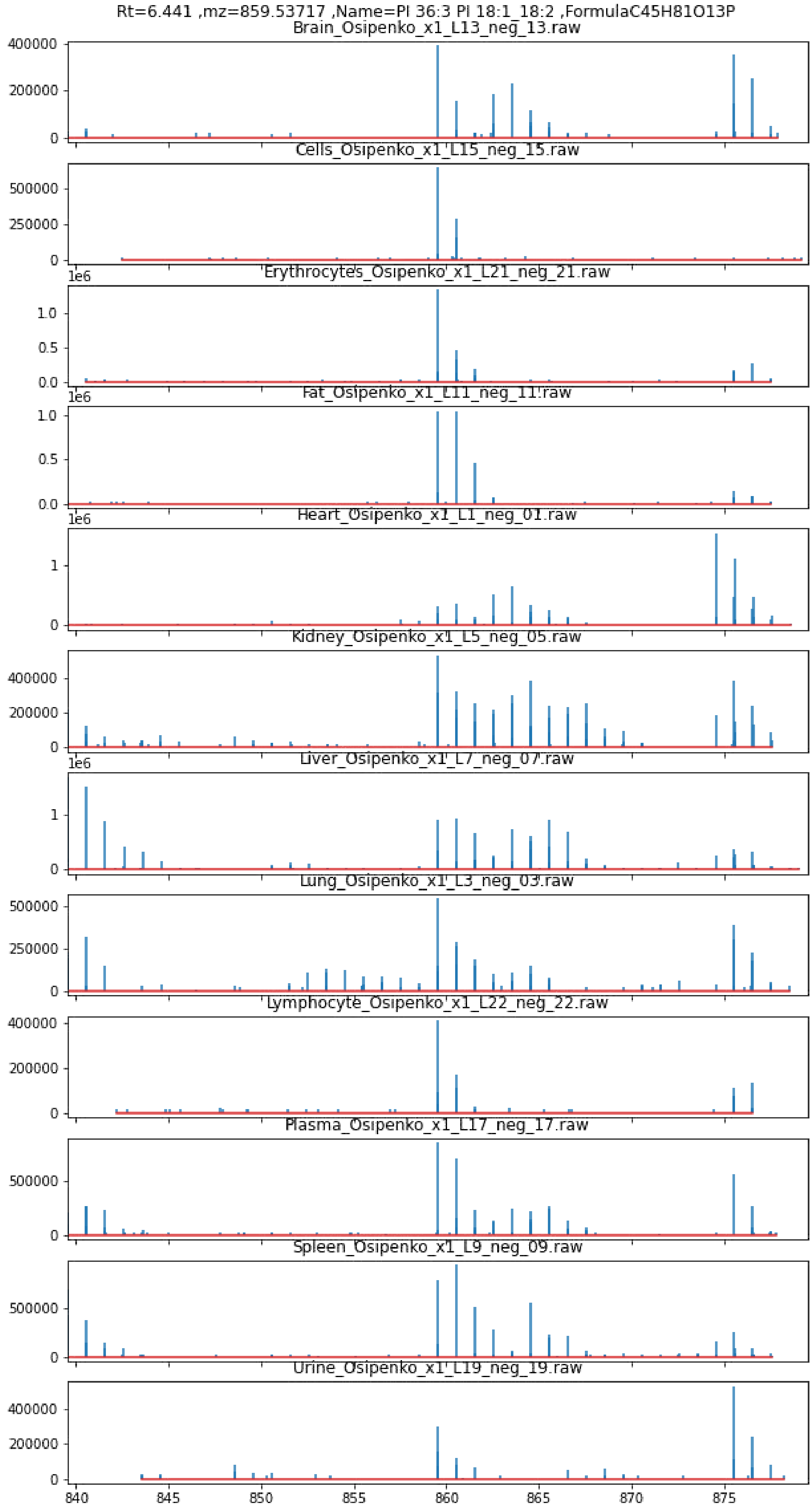
Rt=6.441 ,mz=859.53717 ,Name=PI 36:3 PI 18:1_18:2 ,FormulaC45H81O13P
Brain_Osipenko_x1_L13_neg_13.raw
Cells_Osipenko_x1_L15_neg_15.raw
Erythrocytes_Osipenko_x1_L21_neg_21.raw
Fat_Osipenko_x1_L11_neg_11.raw
Heart_Osipenko_x1_L1_neg_01.raw
Kidney_Osipenko_x1_L5_neg_05.raw
Liver_Osipenko_x1_L7_neg_07.raw
Lung_Osipenko_x1_L3_neg_03.raw
Lymphocyte_Osipenko_x1_L22_neg_22.raw
Plasma_Osipenko_x1_L17_neg_17.raw
Spleen_Osipenko_x1_L9_neg_09.raw
Urine_Osipenko_x1_L19_neg_19.raw
1e6
840
845
850
855
860
865
870
875

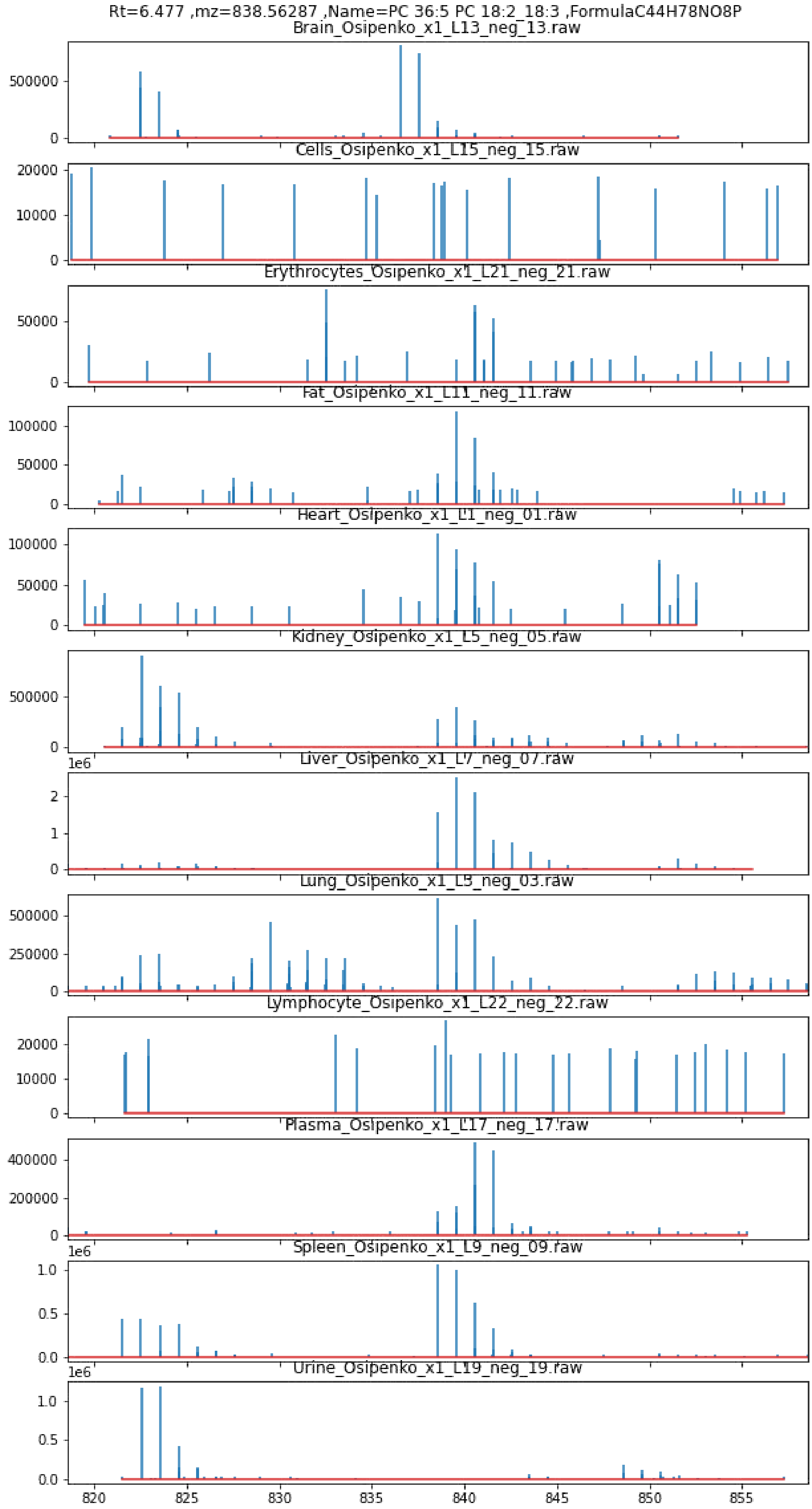

Rt=6.477 ,mz=838.56287 ,Name=PC 36:5 PC 18:2_18:3 ,FormulaC44H78NO8P
Brain_Osipenko_x1_L13_neg_13.raw
Cells_Osipenko_x1_L15_neg_15.raw
Erythrocytes_Osipenko_x1_L21_neg_21.raw
Fat_Osipenko_x1_L11_neg_11.raw
Heart_Osipenko_x1_L1_neg_01.raw
Kidney_Osipenko_x1_L5_neg_05.raw
Liver_Osipenko_x1_L7_neg_07.raw
Lung_Osipenko_x1_L3_neg_03.raw
Lymphocyte_Osipenko_x1_L22_neg_22.raw
Plasma_Osipenko_x1_L17_neg_17.raw
Spleen_Osipenko_x1_L9_neg_09.raw
Urine_Osipenko_x1_L19_neg_19.raw
1e6
820
825
830
835
840
845
850
855

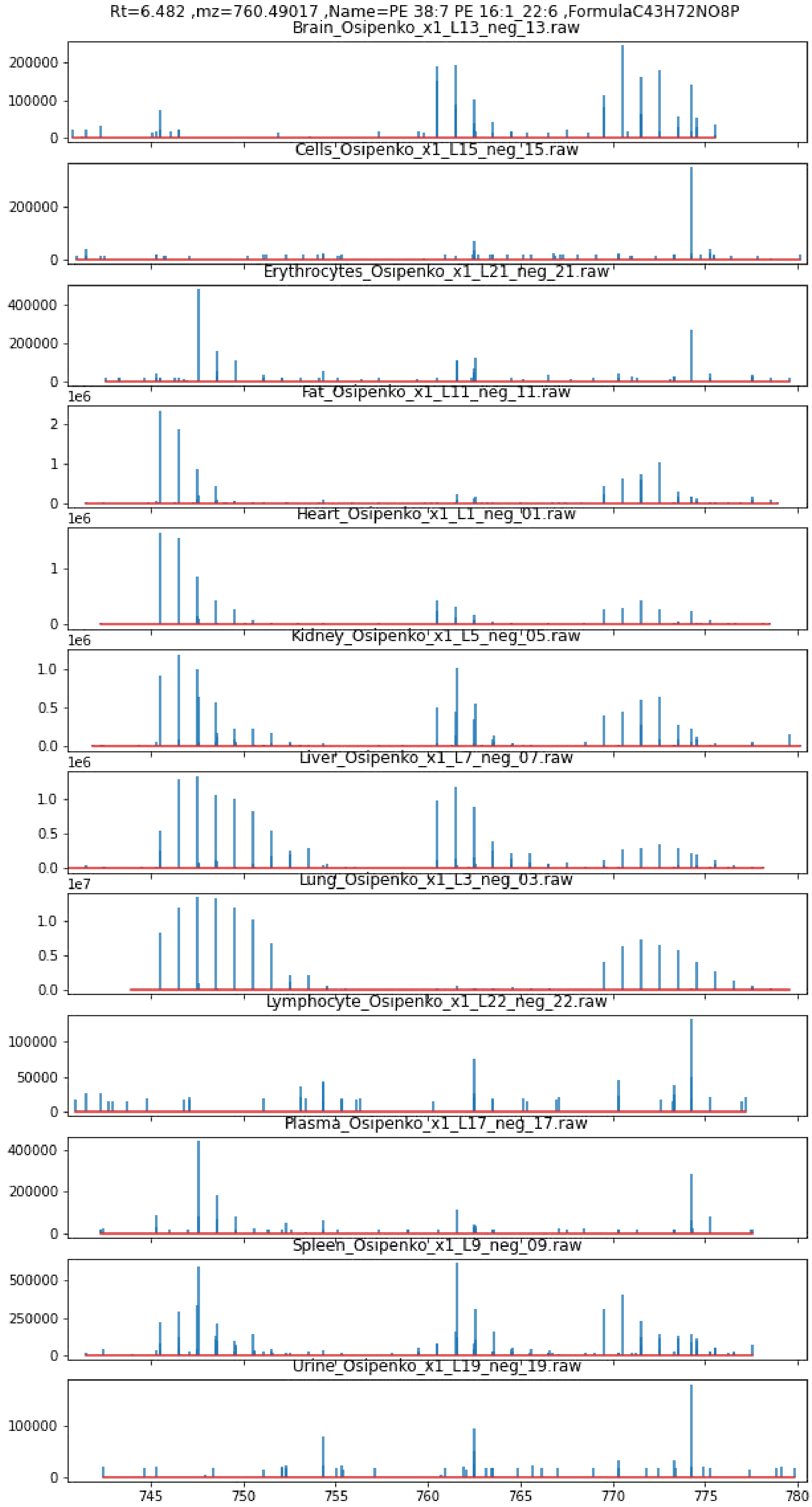

Rt=6.482 ,mz=760.49017 ,Name=PE 38:7 PE 16:1_22:6 ,FormulaC43H72NO8P
Brain_Osipenko_x1_L13_neg_13.raw
Cells_Osipenko_x1_L15_neg_15.raw
Erythrocytes_Osipenko_x1_L21_neg_21.raw
Fat_Osipenko_x1_L11_neg_11.raw
Heart_Osipenko_x1_L1_neg_01.raw
Kidney_Osipenko_x1_L5_neg_05.raw
Liver_Osipenko_x1_L7_neg_07.raw
Lung_Osipenko_x1_L3_neg_03.raw
Lymphocyte_Osipenko_x1_L22_neg_22.raw
Plasma_Osipenko_x1_L17_neg_17.raw
Spleen_Osipenko_x1_L9_neg_09.raw
Urine_Osipenko_x1_L19_neg_19.raw
745
750
755
760
765
770
775
780

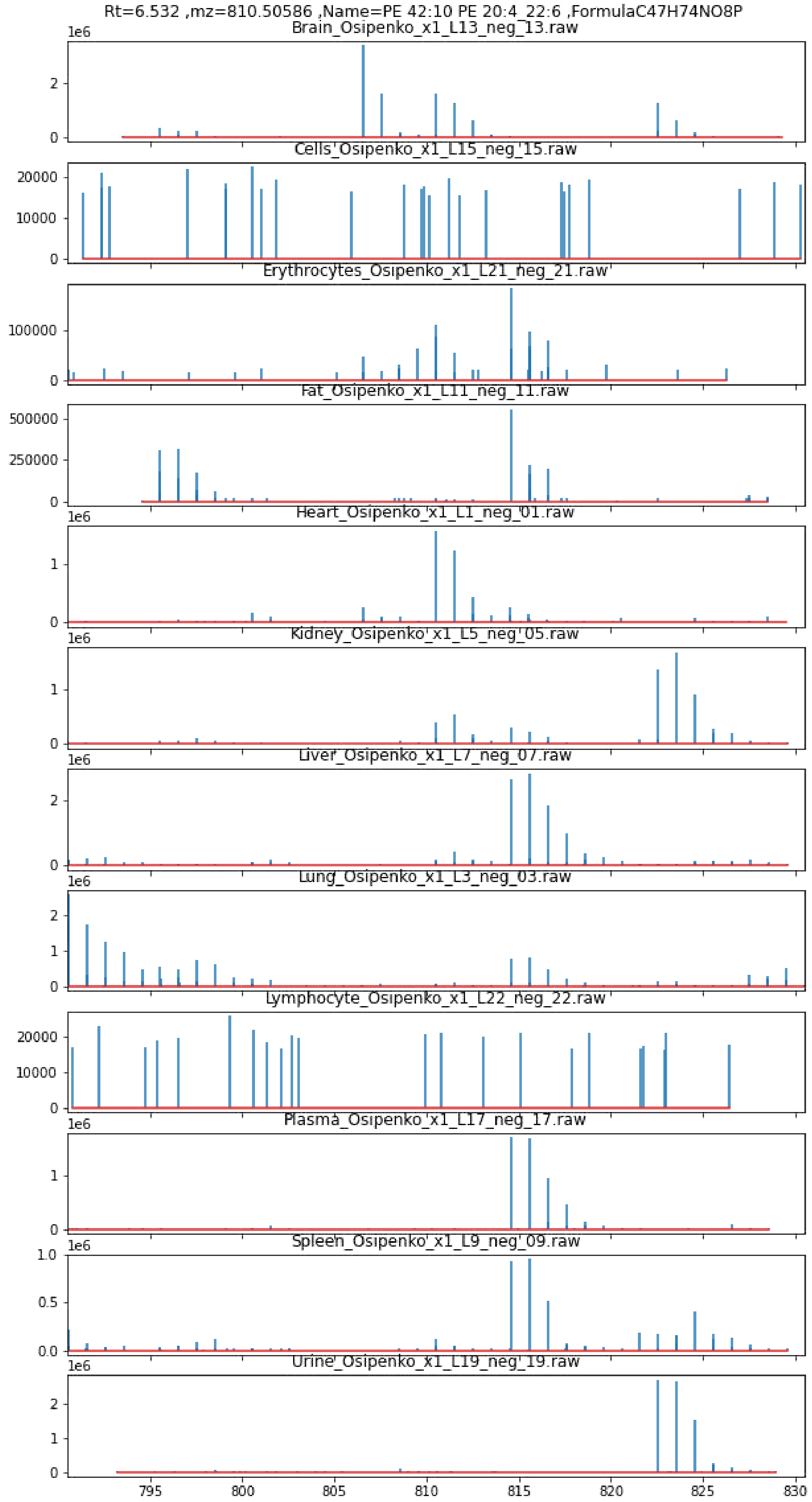

Rt=6.532 ,mz=810.50586 ,Name=PE 42:10 PE 20:4_22:6 ,FormulaC47H74NO8P
Brain_Osipenko_x1_L13_neg_13.raw
Cells_Osipenko_x1_L15_neg_15.raw
Erythrocytes_Osipenko_x1_L21_neg_21.raw
Fat_Osipenko_x1_L11_neg_11.raw
Heart_Osipenko_x1_L1_neg_01.raw
Kidney_Osipenko_x1_L5_neg_05.raw
Liver_Osipenko_x1_L7_neg_07.raw
Lung_Osipenko_x1_L3_neg_03.raw
Lymphocyte_Osipenko_x1_L22_neg_22.raw
Plasma_Osipenko_x1_L17_neg_17.raw
Spleen_Osipenko_x1_L9_neg_09.raw
Urine_Osipenko_x1_L19_neg_19.raw
795
800
805
810
815
820
825
830

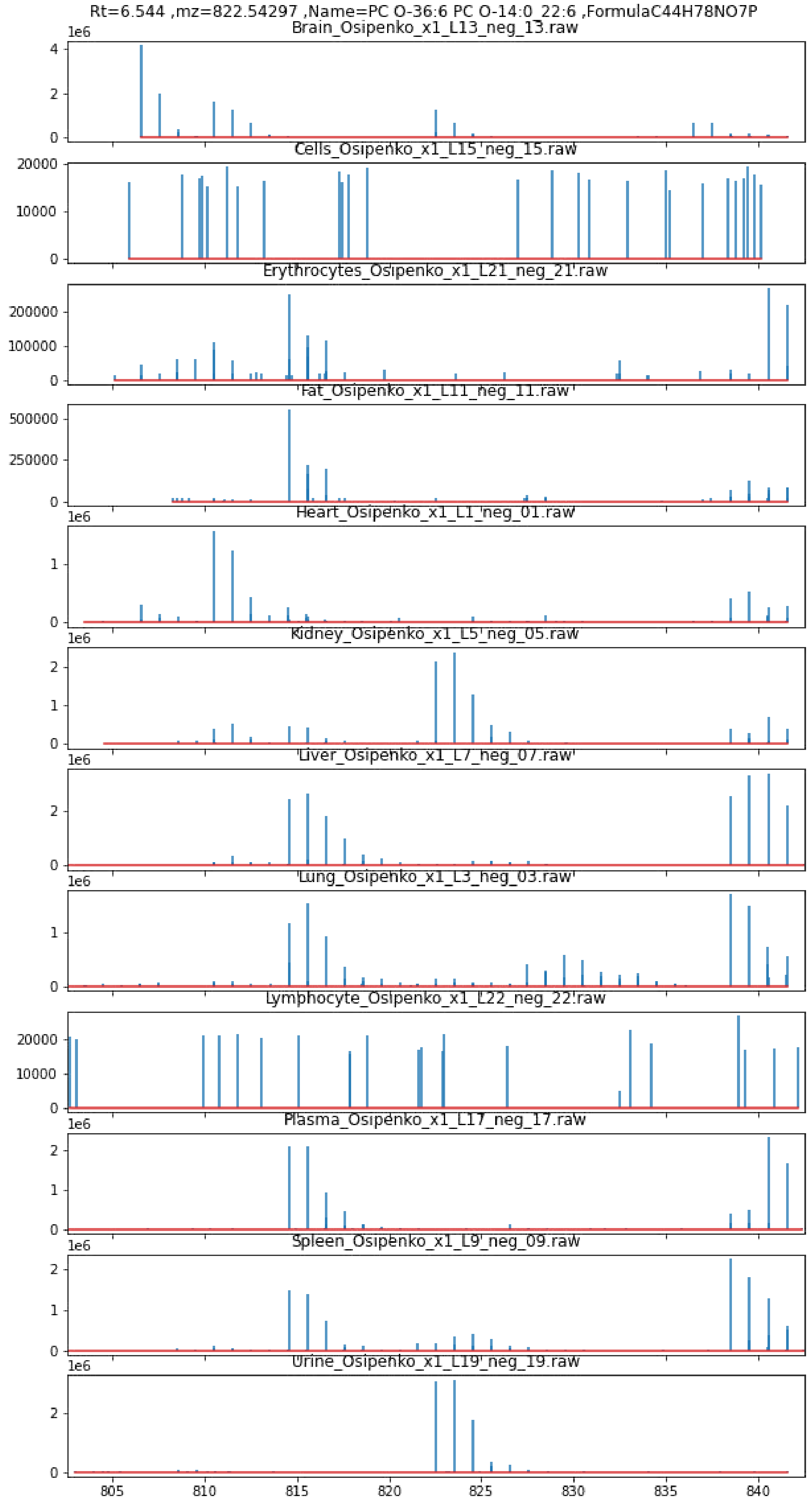

Rt=6.544 ,mz=822.54297 ,Name=PC O-36:6 PC O-14:0_22:6 ,FormulaC44H78NO7P
Brain_Osipenko_x1_L13_neg_13.raw
Cells_Osipenko_x1_L15_neg_15.raw
Erythrocytes_Osipenko_x1_L21_neg_21.raw
Fat_Osipenko_x1_L11_neg_11.raw
Heart_Osipenko_x1_L1_neg_01.raw
Kidney_Osipenko_x1_L5_neg_05.raw
Liver_Osipenko_x1_L7_neg_07.raw
Lung_Osipenko_x1_L3_neg_03.raw
Lymphocyte_Osipenko_x1_L22_neg_22.raw
Plasma_Osipenko_x1_L17_neg_17.raw
Spleen_Osipenko_x1_L9_neg_09.raw
Urine_Osipenko_x1_L19_neg_19.raw
1e6
805
810
815
820
825
830
835
840

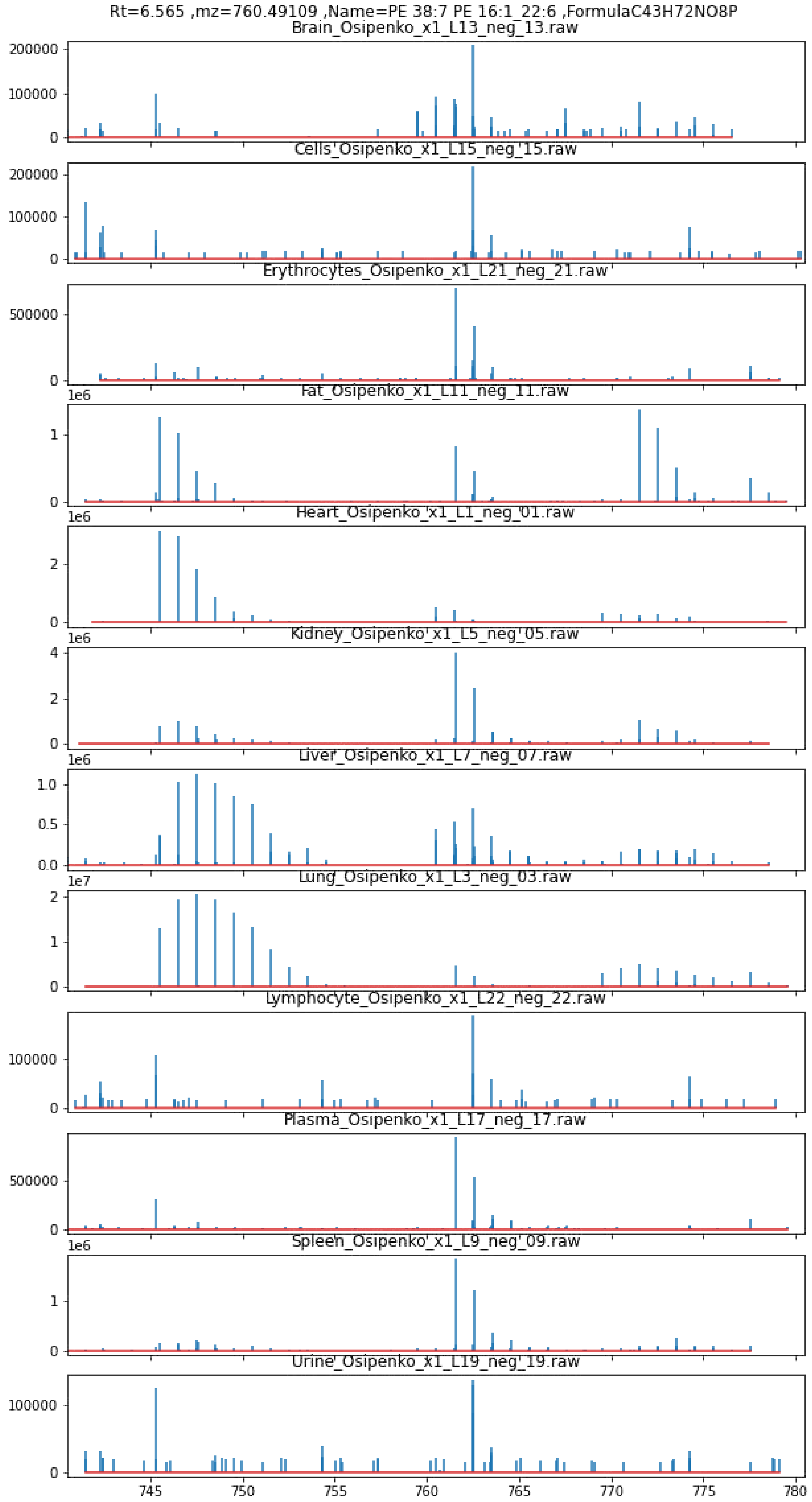
Rt=6.565 ,mz=760.49109 ,Name=PE 38:7 PE 16:1_22:6 ,FormulaC43H72NO8P
Brain_Osipenko_x1_L13_neg_13.raw
Cells_Osipenko_x1_L15_neg_15.raw
Erythrocytes_Osipenko_x1_L21_neg_21.raw
Fat_Osipenko_x1_L11_neg_11.raw
Heart_Osipenko_x1_L1_neg_01.raw
Kidney_Osipenko_x1_L5_neg_05.raw
Liver_Osipenko_x1_L7_neg_07.raw
Lung_Osipenko_x1_L3_neg_03.raw
Lymphocyte_Osipenko_x1_L22_neg_22.raw
Plasma_Osipenko_x1_L17_neg_17.raw
Spleen_Osipenko_x1_L9_neg_09.raw
Urine_Osipenko_x1_L19_neg_19.raw
745
750
755
760
765
770
775
780

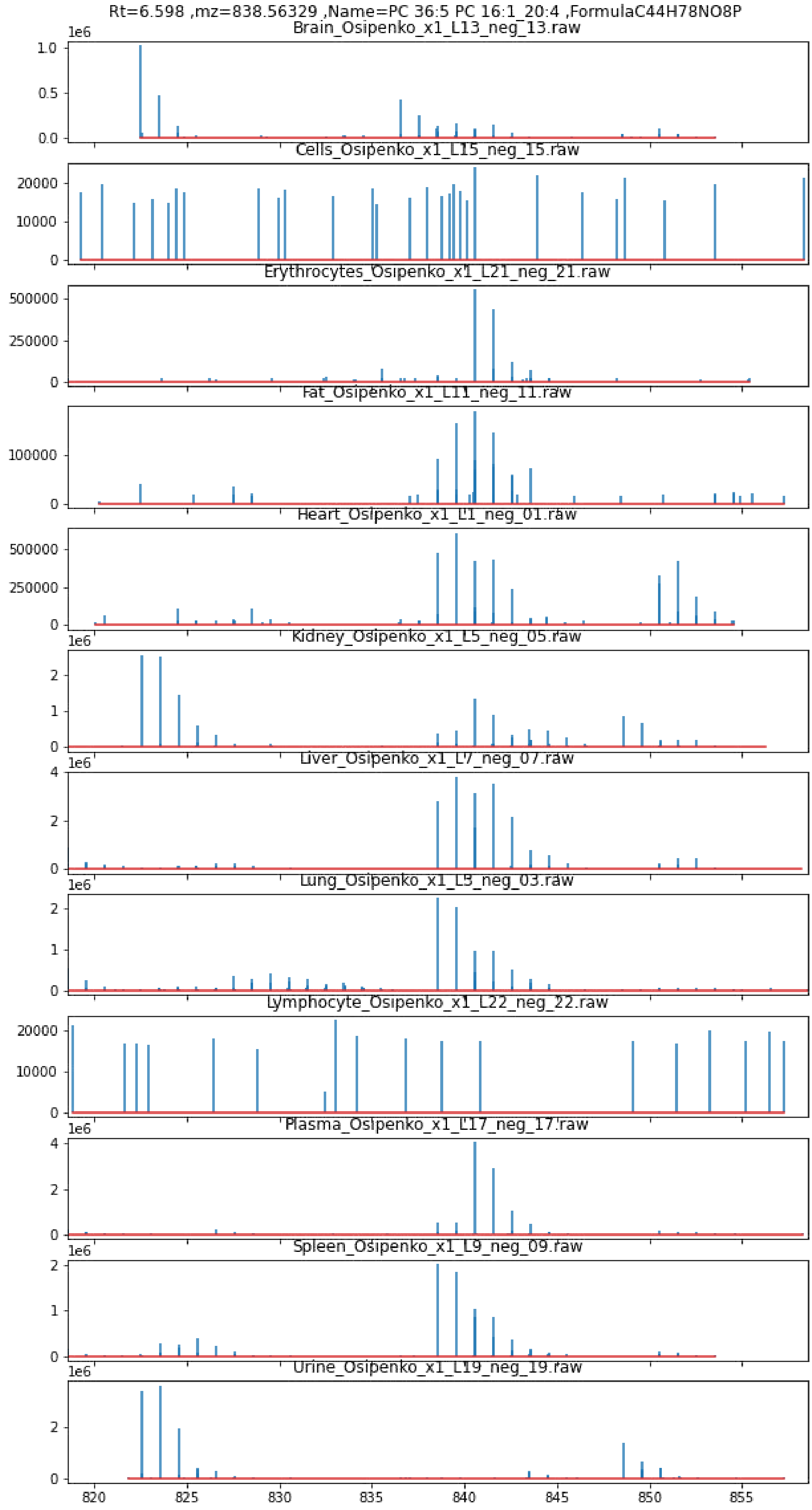

Rt=6.598 ,mz=838.56329 ,Name=PC 36:5 PC 16:1_20:4 ,FormulaC44H78NO8P
Brain_Osipenko_x1_L13_neg_13.raw
Cells_Osipenko_x1_L15_neg_15.raw
Erythrocytes_Osipenko_x1_L21_neg_21.raw
Fat_Osipenko_x1_L11_neg_11.raw
Heart_Osipenko_x1_L1_neg_01.raw
Kidney_Osipenko_x1_L5_neg_05.raw
Liver_Osipenko_x1_L7_neg_07.raw
Lung_Osipenko_x1_L3_neg_03.raw
Lymphocyte_Osipenko_x1_L22_neg_22.raw
Plasma_Osipenko_x1_L17_neg_17.raw
Spleen_Osipenko_x1_L9_neg_09.raw
Urine_Osipenko_x1_L19_neg_19.raw
1e6
820
825
830
835
840
845
850
855

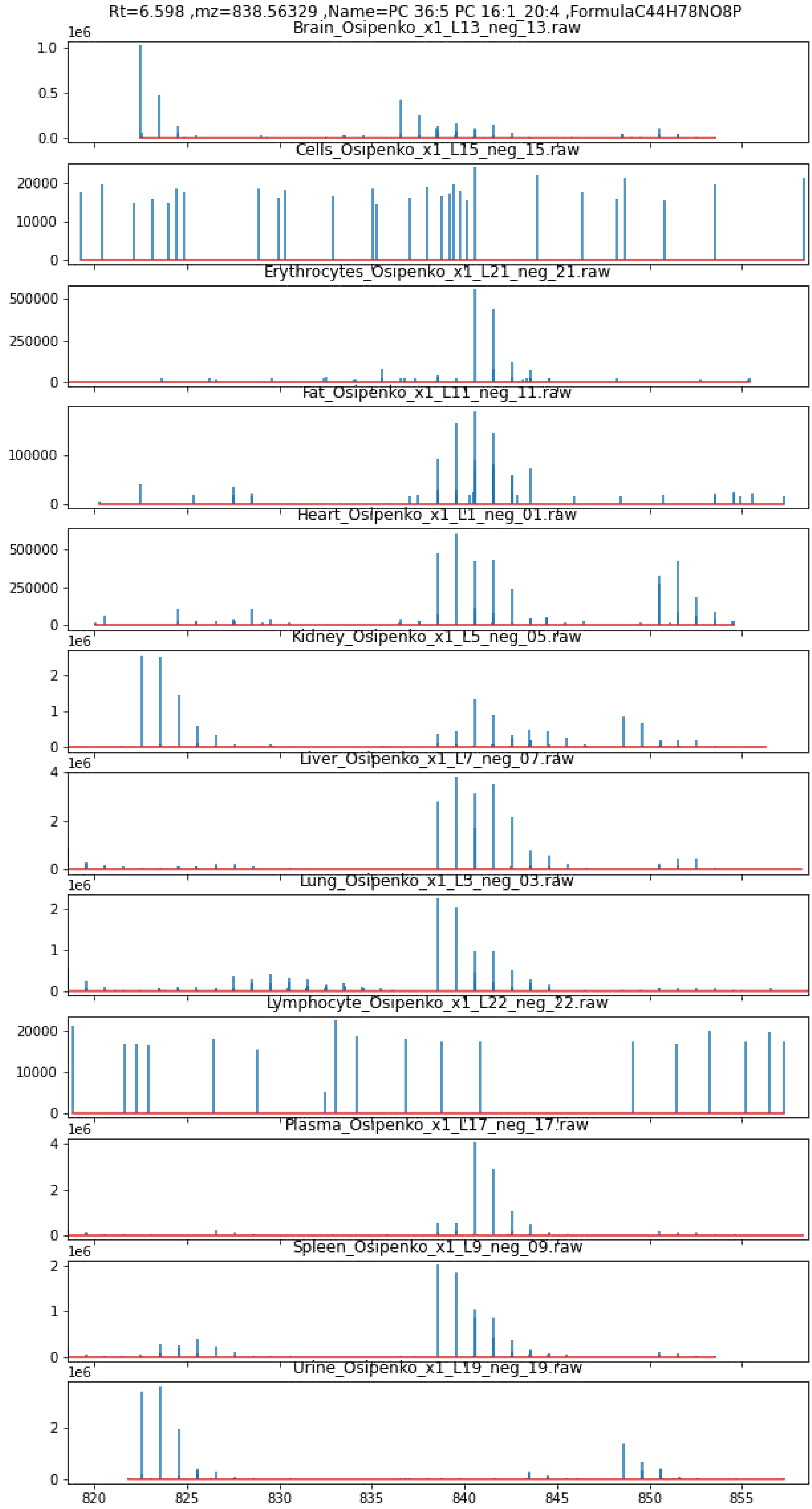
Rt=6.598 ,mz=838.56329 ,Name=PC 36:5 PC 16:1_20:4 ,FormulaC44H78NO8P
Brain_Osipenko_x1_L13_neg_13.raw
Cells_Osipenko_x1_L15_neg_15.raw
Erythrocytes_Osipenko_x1_L21_neg_21.raw
Fat_Osipenko_x1_L11_neg_11.raw
Heart_Osipenko_x1_L1_neg_01.raw
Kidney_Osipenko_x1_L5_neg_05.raw
Liver_Osipenko_x1_L7_neg_07.raw
Lung_Osipenko_x1_L3_neg_03.raw
Lymphocyte_Osipenko_x1_L22_neg_22.raw
Plasma_Osipenko_x1_L17_neg_17.raw
Spleen_Osipenko_x1_L9_neg_09.raw
Urine_Osipenko_x1_L19_neg_19.raw
820
825
830
835
840
845
850
855

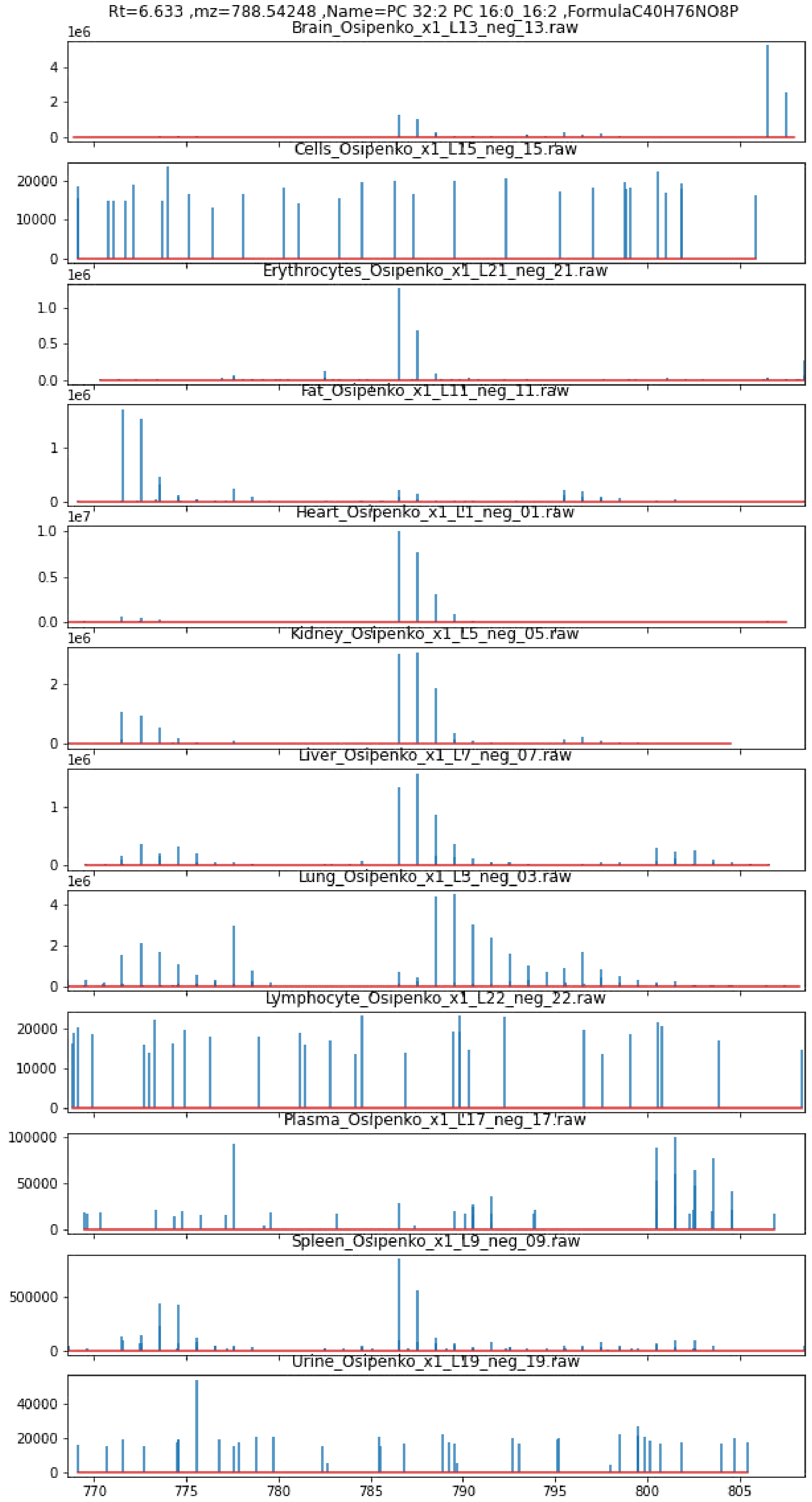
Rt=6.633 ,mz=788.54248 ,Name=PC 32:2 PC 16:0_16:2 ,FormulaC40H76NO8P
Brain_Osipenko_x1_L13_neg_13.raw
Cells_Osipenko_x1_L15_neg_15.raw
Erythrocytes_Osipenko_x1_L21_neg_21.raw
Fat_Osipenko_x1_L11_neg_11.raw
Heart_Osipenko_x1_L1_neg_01.raw
Kidney_Osipenko_x1_L5_neg_05.raw
Liver_Osipenko_x1_L7_neg_07.raw
Lung_Osipenko_x1_L3_neg_03.raw
Lymphocyte_Osipenko_x1_L22_neg_22.raw
Plasma_Osipenko_x1_L17_neg_17.raw
Spleen_Osipenko_x1_L9_neg_09.raw
Urine_Osipenko_x1_L19_neg_19.raw
770
775
780
785
790
795
800
805

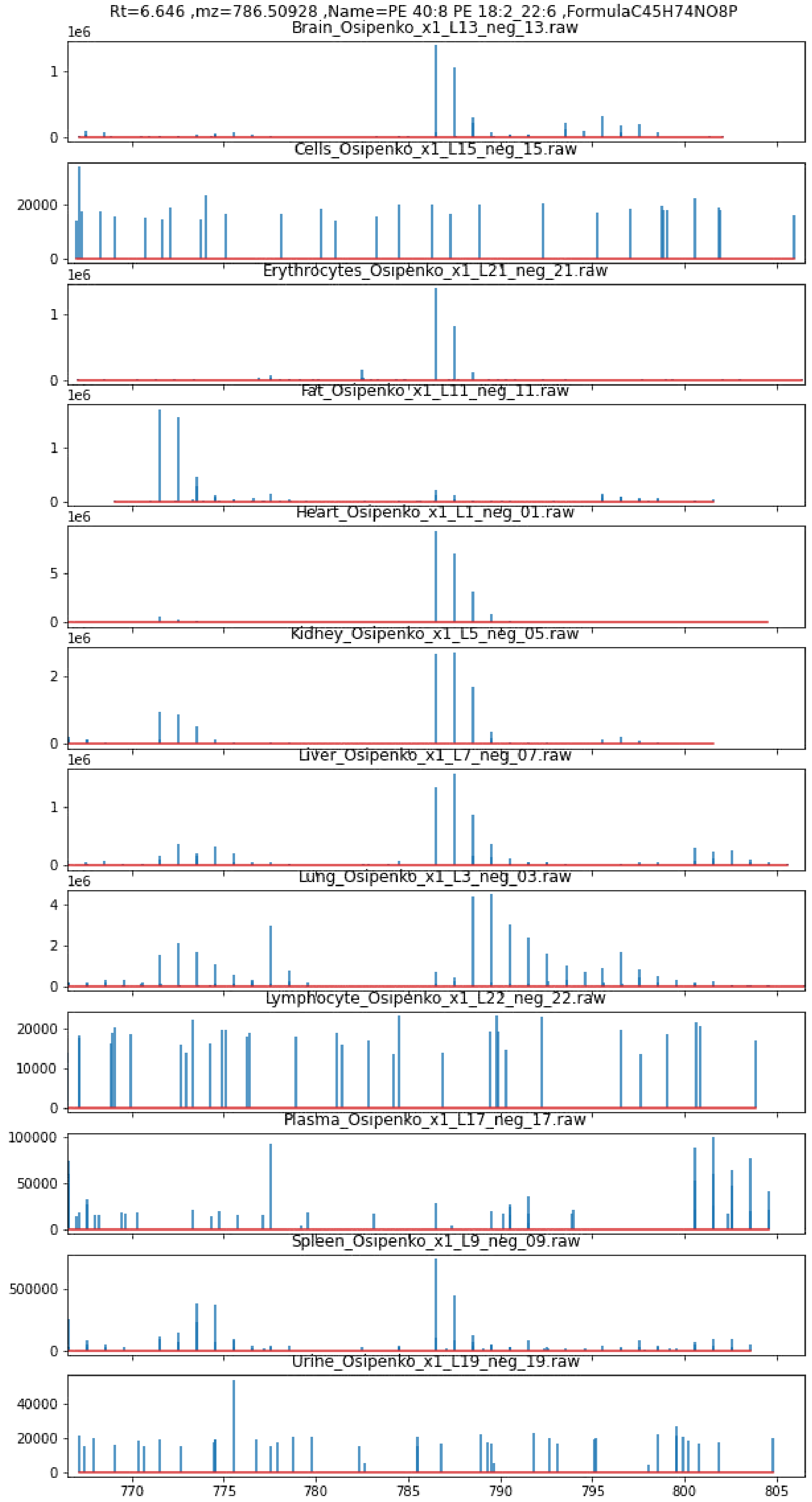

Rt=6.646 ,mz=786.50928 ,Name=PE 40:8 PE 18:2_22:6 ,FormulaC45H74NO8P
Brain_Osipenko_x1_L13_neg_13.raw
Cells_Osipenko_x1_L15_neg_15.raw
Erythrocytes_Osipenko_x1_L21_neg_21.raw
Fat_Osipenko_x1_L11_neg_11.raw
Heart_Osipenko_x1_L1_neg_01.raw
Kidney_Osipenko_x1_L5_neg_05.raw
Liver_Osipenko_x1_L7_neg_07.raw
Lung_Osipenko_x1_L3_neg_03.raw
Lymphocyte_Osipenko_x1_L22_neg_22.raw
Plasma_Osipenko_x1_L17_neg_17.raw
Spleen_Osipenko_x1_L9_neg_09.raw
Urine_Osipenko_x1_L19_neg_19.raw
770
775
780
785
790
795
800
805

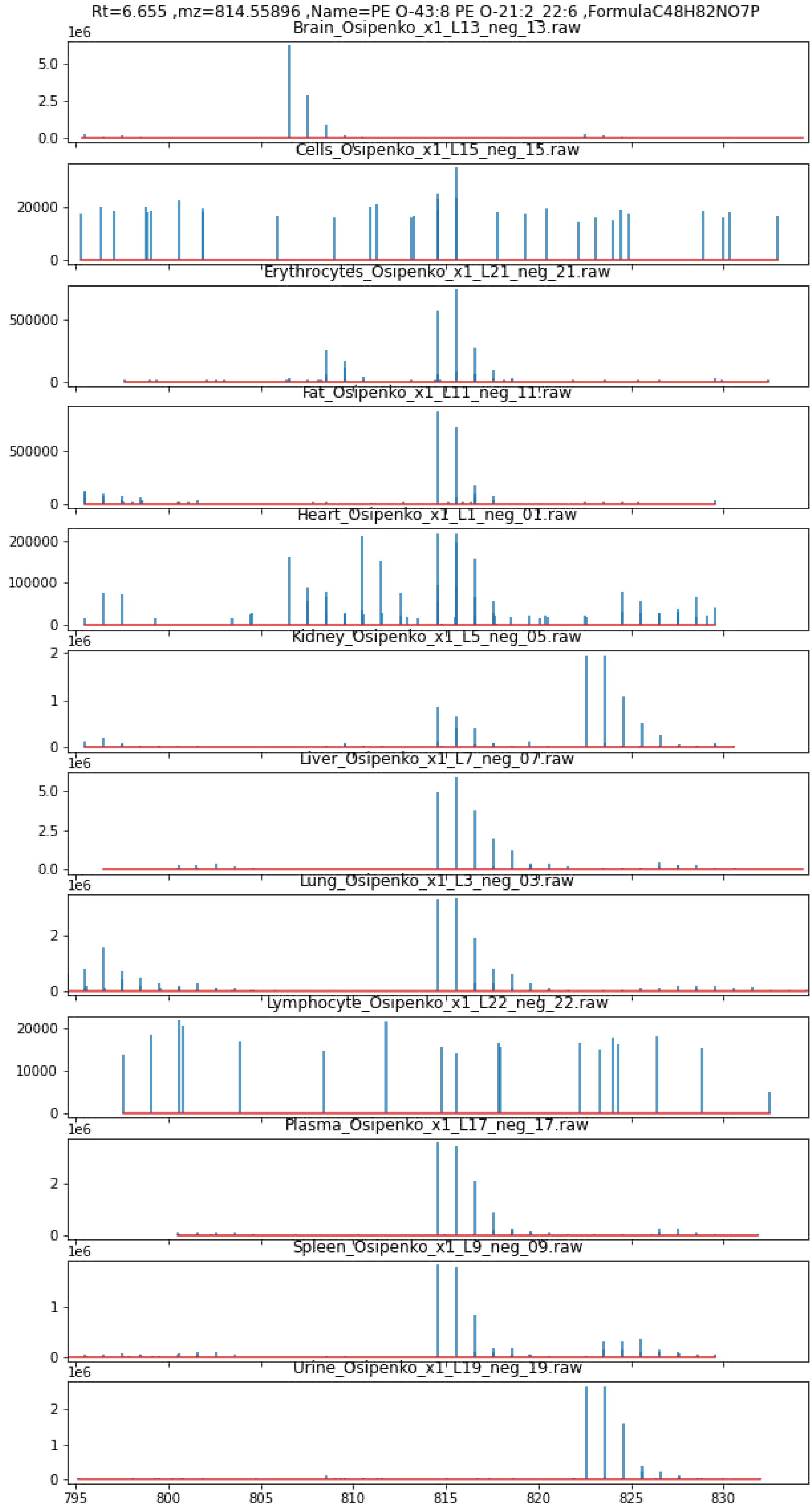

Rt=6.655 ,mz=814.55896 ,Name=PE O-43:8 PE O-21:2_22:6 ,FormulaC48H82NO7P
Brain_Osipenko_x1_L13_neg_13.raw
Cells_Osipenko_x1_L15_neg_15.raw
Erythrocytes_Osipenko_x1_L21_neg_21.raw
Fat_Osipenko_x1_L11_neg_11.raw
Heart_Osipenko_x1_L1_neg_01.raw
Kidney_Osipenko_x1_L5_neg_05.raw
Liver_Osipenko_x1_L7_neg_07.raw
Lung_Osipenko_x1_L3_neg_03.raw
Lymphocyte_Osipenko_x1_L22_neg_22.raw
Plasma_Osipenko_x1_L17_neg_17.raw
Spleen_Osipenko_x1_L9_neg_09.raw
Urine_Osipenko_x1_L19_neg_19.raw
795
800
805
810
815
820
825
830

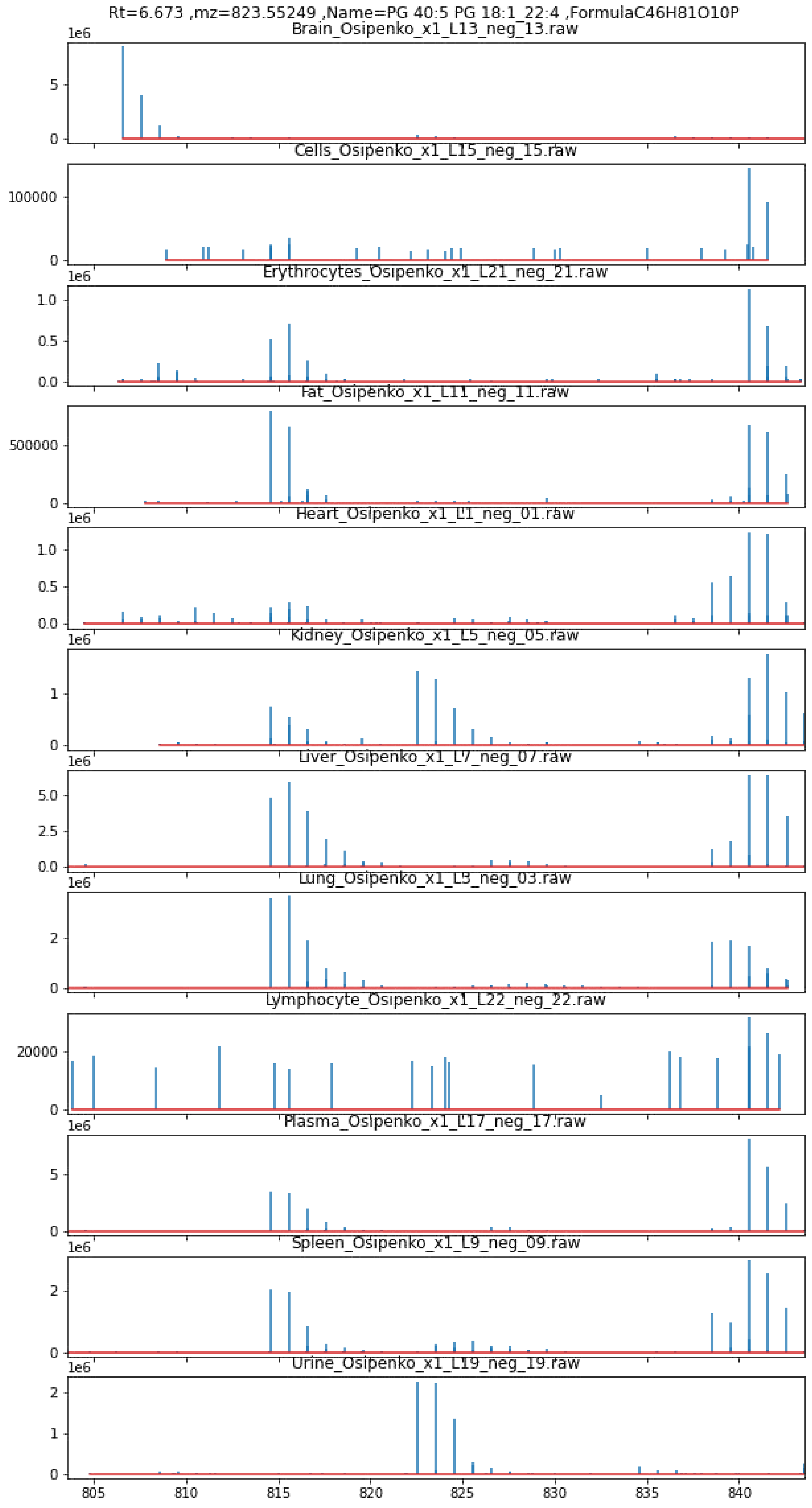

Rt=6.673 ,mz=823.55249 ,Name=PG 40:5 PG 18:1_22:4 ,FormulaC46H81O10P
Brain_Osipenko_x1_L13_neg_13.raw
Cells_Osipenko_x1_L15_neg_15.raw
Erythrocytes_Osipenko_x1_L21_neg_21.raw
Fat_Osipenko_x1_L11_neg_11.raw
Heart_Osipenko_x1_L1_neg_01.raw
Kidney_Osipenko_x1_L5_neg_05.raw
Liver_Osipenko_x1_L7_neg_07.raw
Lung_Osipenko_x1_L3_neg_03.raw
Lymphocyte_Osipenko_x1_L22_neg_22.raw
Plasma_Osipenko_x1_L17_neg_17.raw
Spleen_Osipenko_x1_L9_neg_09.raw
Urine_Osipenko_x1_L19_neg_19.raw

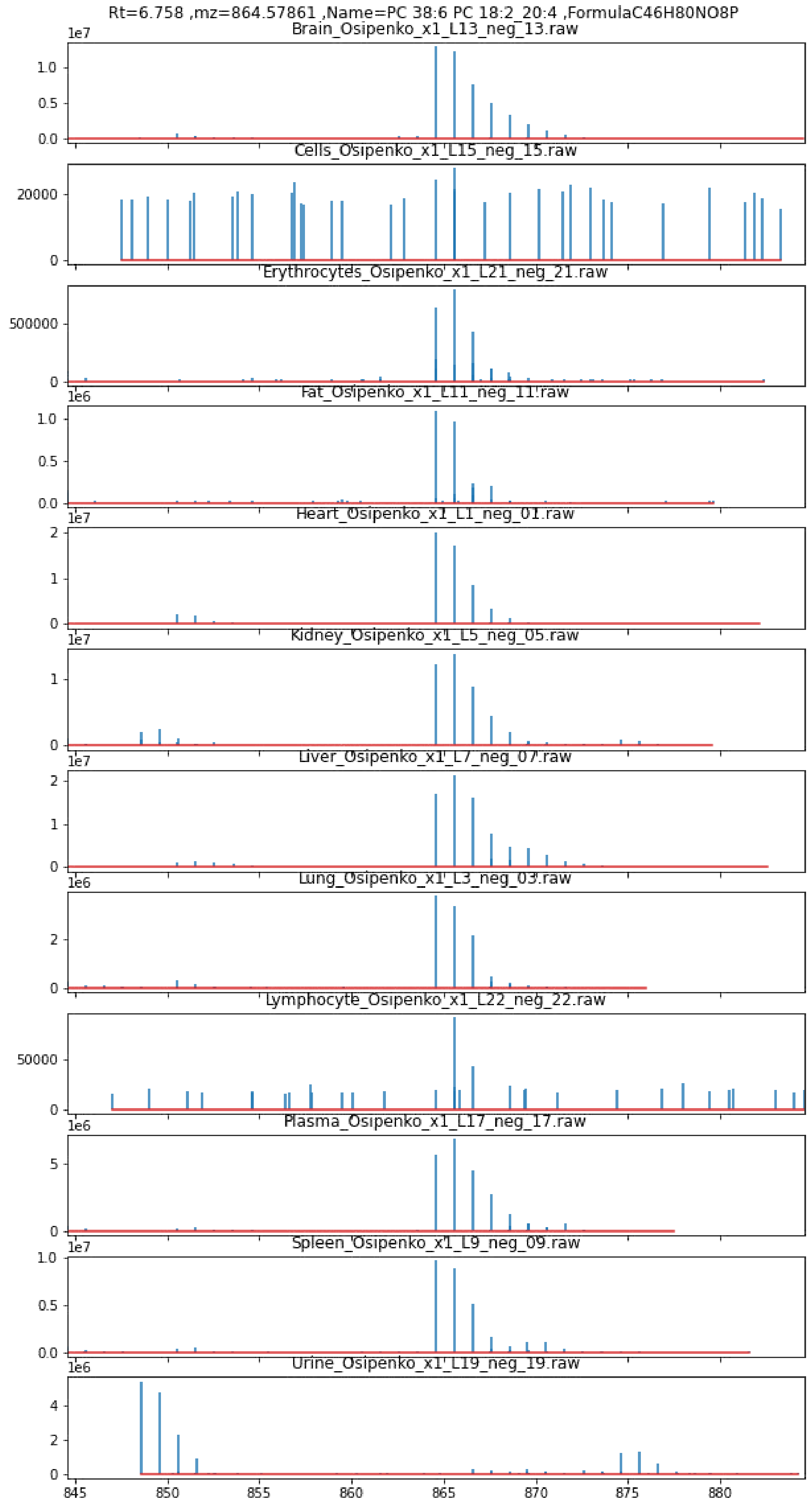

Rt=6.758 ,mz=864.57861 ,Name=PC 38:6 PC 18:2_20:4 ,FormulaC46H80NO8P
Brain_Osipenko_x1_L13_neg_13.raw
Cells_Osipenko_x1_L15_neg_15.raw
Erythrocytes_Osipenko_x1_L21_neg_21.raw
Fat_Osipenko_x1_L11_neg_11.raw
Heart_Osipenko_x1_L1_neg_01.raw
Kidney_Osipenko_x1_L5_neg_05.raw
Liver_Osipenko_x1_L7_neg_07.raw
Lung_Osipenko_x1_L3_neg_03.raw
Lymphocyte_Osipenko_x1_L22_neg_22.raw
Plasma_Osipenko_x1_L17_neg_17.raw
Spleen_Osipenko_x1_L9_neg_09.raw
Urine_Osipenko_x1_L19_neg_19.raw
845 850 855 860 865 870 875 880

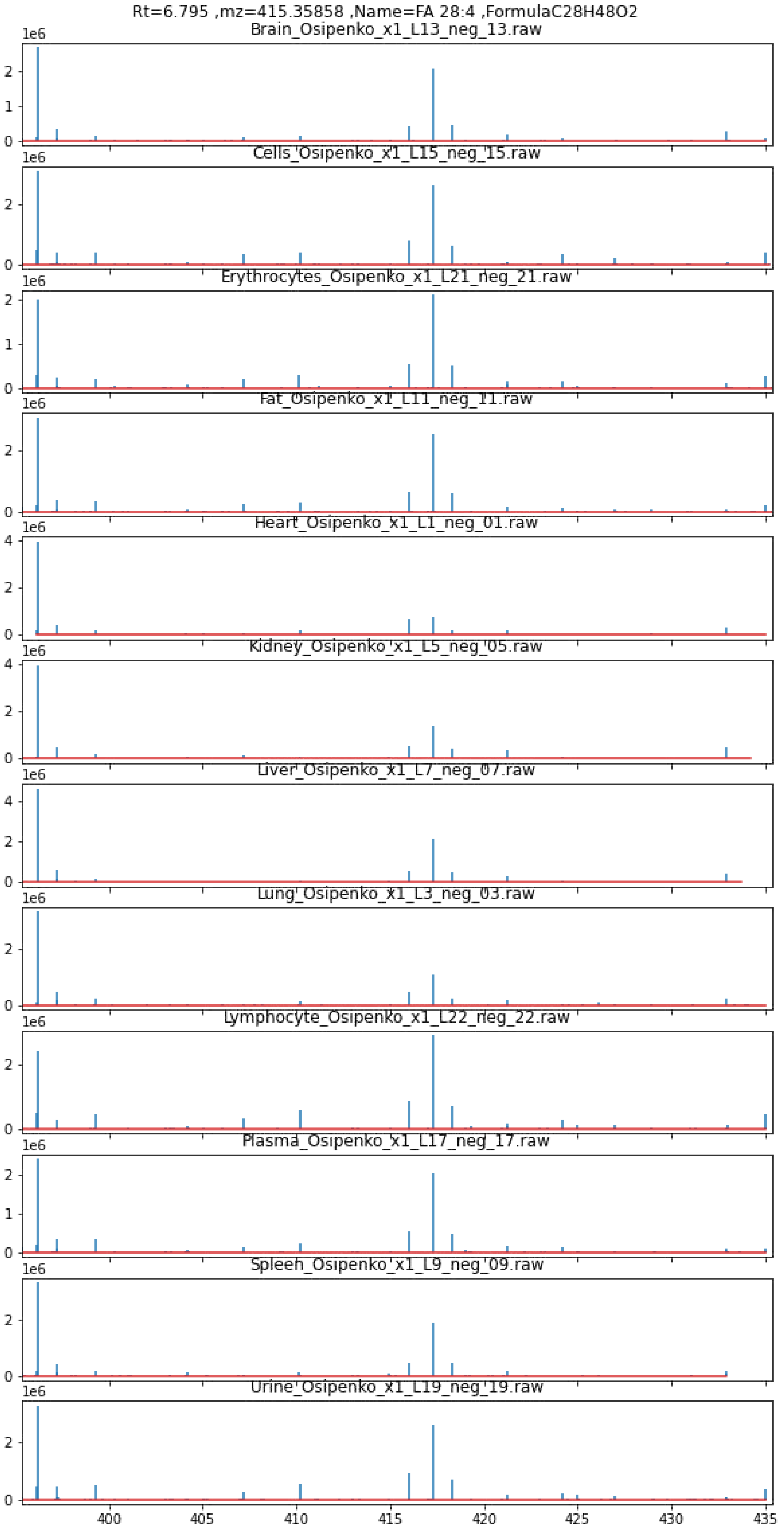

Rt=6.795 ,mz=415.35858 ,Name=FA 28:4 ,FormulaC28H48O2
Brain_Osipenko_x1_L13_neg_13.raw
Cells_Osipenko_x1_L15_neg_15.raw
Erythrocytes_Osipenko_x1_L21_neg_21.raw
Fat_Osipenko_x1_L11_neg_11.raw
Heart_Osipenko_x1_L1_neg_01.raw
Kidney_Osipenko_x1_L5_neg_05.raw
Liver_Osipenko_x1_L7_neg_07.raw
Lung_Osipenko_x1_L3_neg_03.raw
Lymphocyte_Osipenko_x1_L22_neg_22.raw
Plasma_Osipenko_x1_L17_neg_17.raw
Spleen_Osipenko_x1_L9_neg_09.raw
Urine_Osipenko_x1_L19_neg_19.raw
1e6
400
405
410
415
420
425
430
435

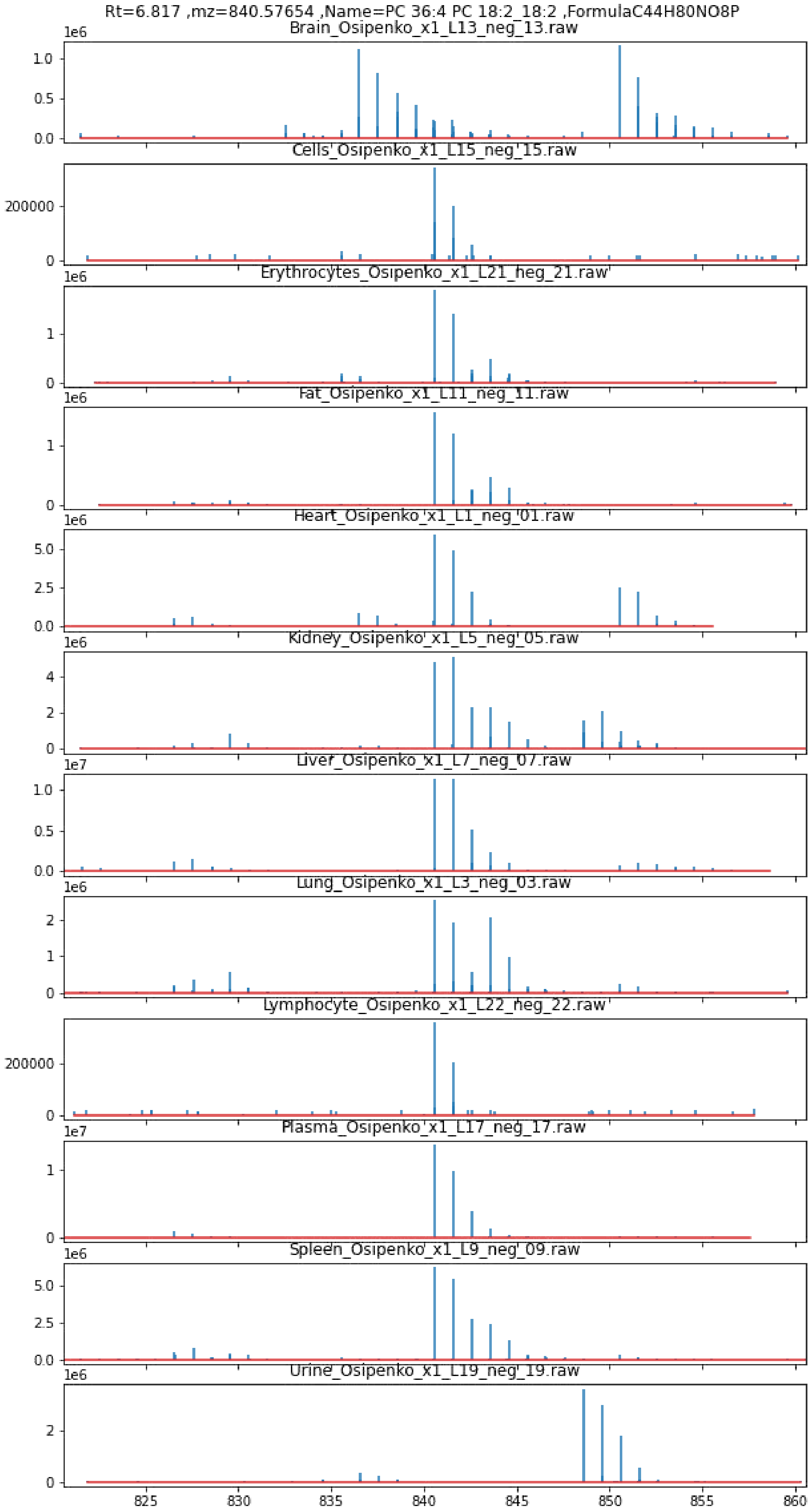

Rt=6.817 ,mz=840.57654 ,Name=PC 36:4 PC 18:2_18:2 ,FormulaC44H80NO8P
Brain_Osipenko_x1_L13_neg_13.raw
Cells_Osipenko_x1_L15_neg_15.raw
Erythrocytes_Osipenko_x1_L21_neg_21.raw
Fat_Osipenko_x1_L11_neg_11.raw
Heart_Osipenko_x1_L1_neg_01.raw
Kidney_Osipenko_x1_L5_neg_05.raw
Liver_Osipenko_x1_L7_neg_07.raw
Lung_Osipenko_x1_L3_neg_03.raw
Lymphocyte_Osipenko_x1_L22_neg_22.raw
Plasma_Osipenko_x1_L17_neg_17.raw
Spleen_Osipenko_x1_L9_neg_09.raw
Urine_Osipenko_x1_L19_neg_19.raw
825
830
835
840
845
850
855
860

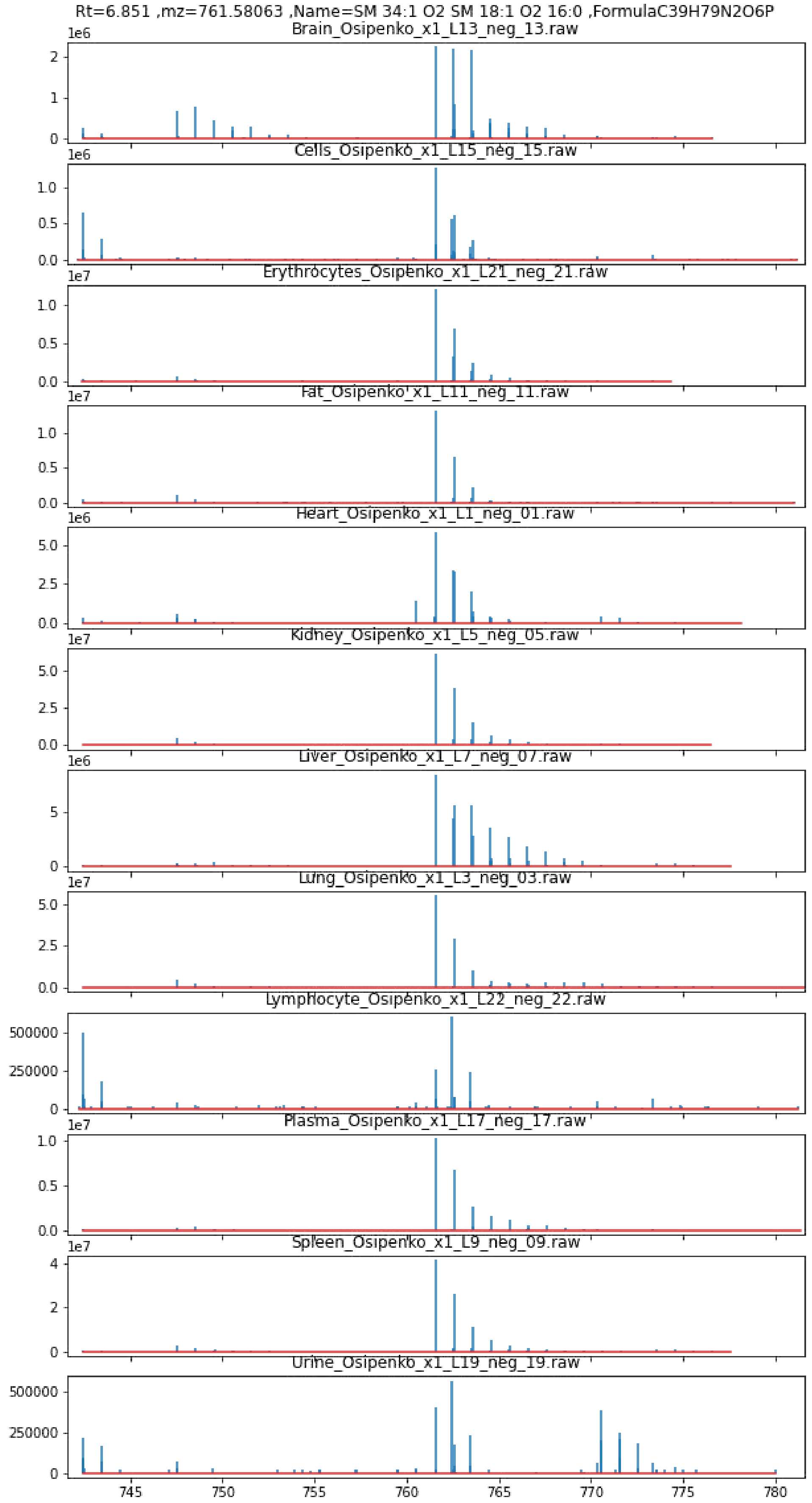
Rt=6.851 ,mz=761.58063 ,Name=SM 34:1 O2 SM 18:1 O2 16:0 ,FormulaC39H79N2O6P
Brain_Osipenko_x1_L13_neg_13.raw
Cells_Osipenko_x1_L15_neg_15.raw
Erythrocytes_Osipenko_x1_L21_neg_21.raw
Fat_Osipenko_x1_L11_neg_11.raw
Heart_Osipenko_x1_L1_neg_01.raw
Kidney_Osipenko_x1_L5_neg_05.raw
Liver_Osipenko_x1_L7_neg_07.raw
Lung_Osipenko_x1_L3_neg_03.raw
Lymphocyte_Osipenko_x1_L22_neg_22.raw
Plasma_Osipenko_x1_L17_neg_17.raw
Spleen_Osipenko_x1_L9_neg_09.raw
Urine_Osipenko_x1_L19_neg_19.raw
745
750
755
760
765
770
775
780

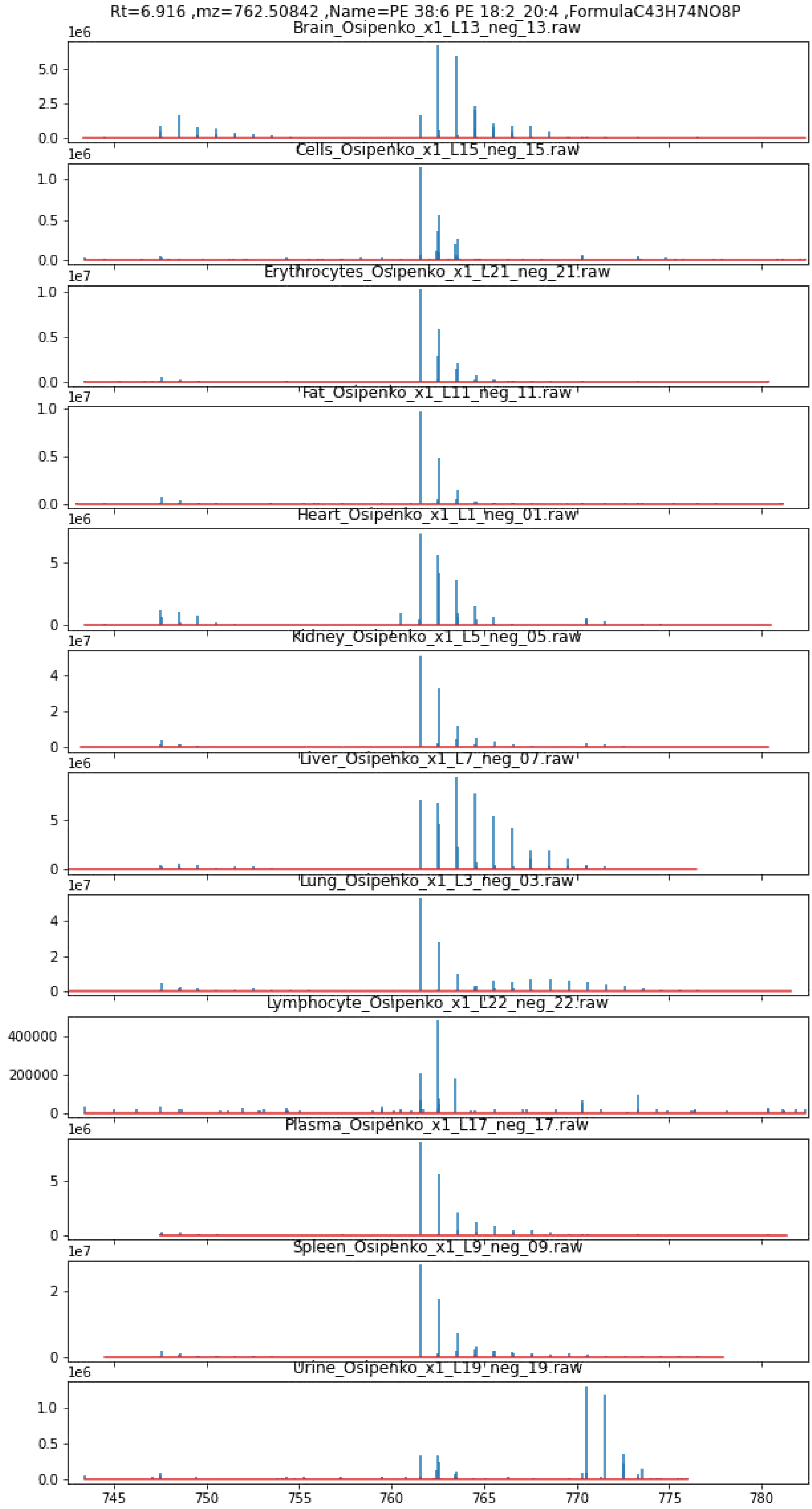

Rt=6.916 ,mz=762.50842 ,Name=PE 38:6 PE 18:2_20:4 ,FormulaC43H74NO8P
Brain_Osipenko_x1_L13_neg_13.raw
Cells_Osipenko_x1_L15_neg_15.raw
Erythrocytes_Osipenko_x1_L21_neg_21.raw
Fat_Osipenko_x1_L11_neg_11.raw
Heart_Osipenko_x1_L1_neg_01.raw
Kidney_Osipenko_x1_L5_neg_05.raw
Liver_Osipenko_x1_L7_neg_07.raw
Lung_Osipenko_x1_L3_neg_03.raw
Lymphocyte_Osipenko_x1_L22_neg_22.raw
Plasma_Osipenko_x1_L17_neg_17.raw
Spleen_Osipenko_x1_L9_neg_09.raw
Urine_Osipenko_x1_L19_neg_19.raw
745
750
755
760
765
770
775
780

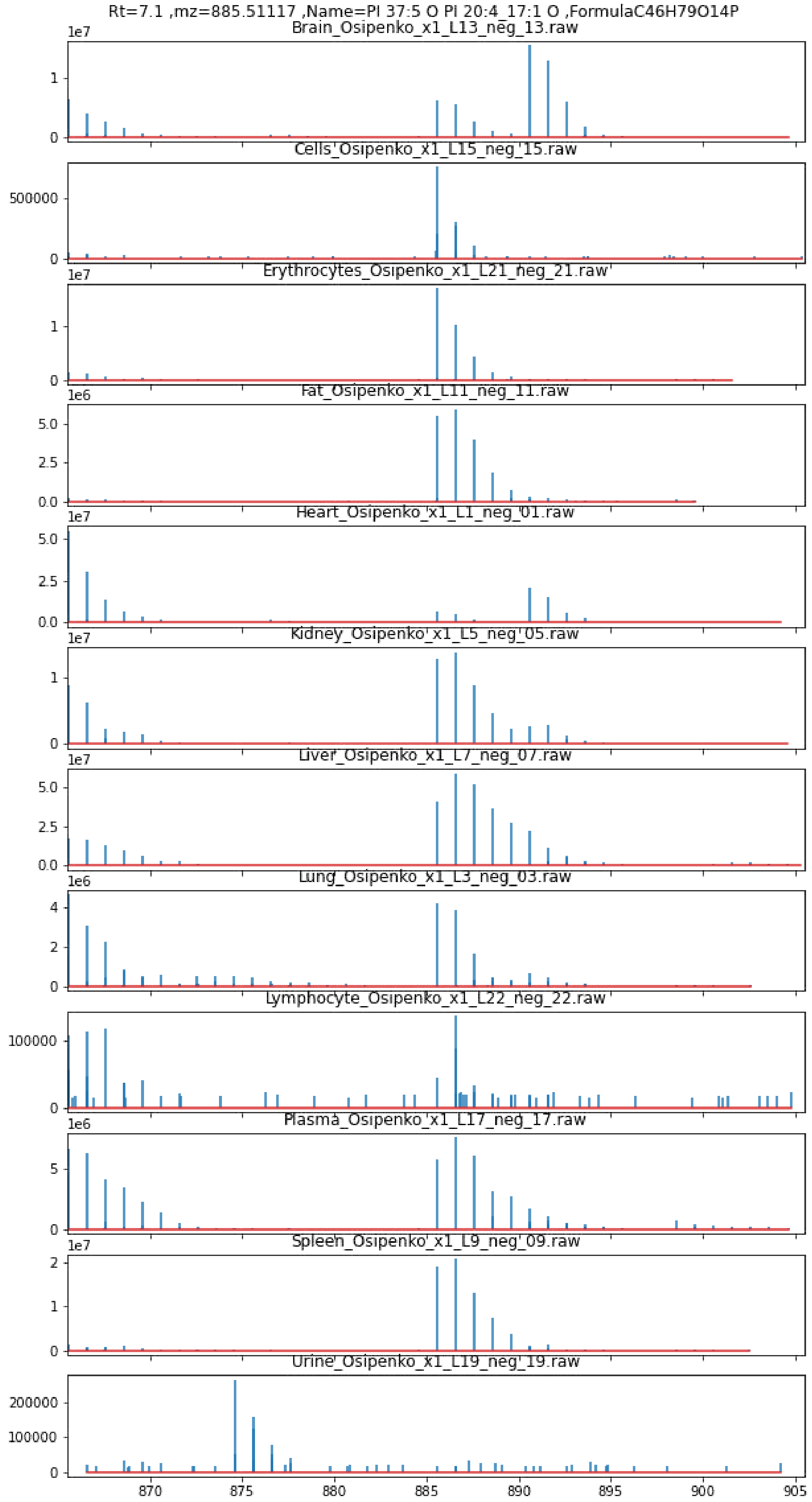
Rt=7.1 ,mz=885.51117 ,Name=PI 37:5 O PI 20:4_17:1 O ,FormulaC46H79O14P
Brain_Osipenko_x1_L13_neg_13.raw
Cells_Osipenko_x1_L15_neg_15.raw
Erythrocytes_Osipenko_x1_L21_neg_21.raw
Fat_Osipenko_x1_L11_neg_11.raw
Heart_Osipenko_x1_L1_neg_01.raw
Kidney_Osipenko_x1_L5_neg_05.raw
Liver_Osipenko_x1_L7_neg_07.raw
Lung_Osipenko_x1_L3_neg_03.raw
Lymphocyte_Osipenko_x1_L22_neg_22.raw
Plasma_Osipenko_x1_L17_neg_17.raw
Spleen_Osipenko_x1_L9_neg_09.raw
Urine_Osipenko_x1_L19_neg_19.raw
870
875
880
885
890
895
900
905

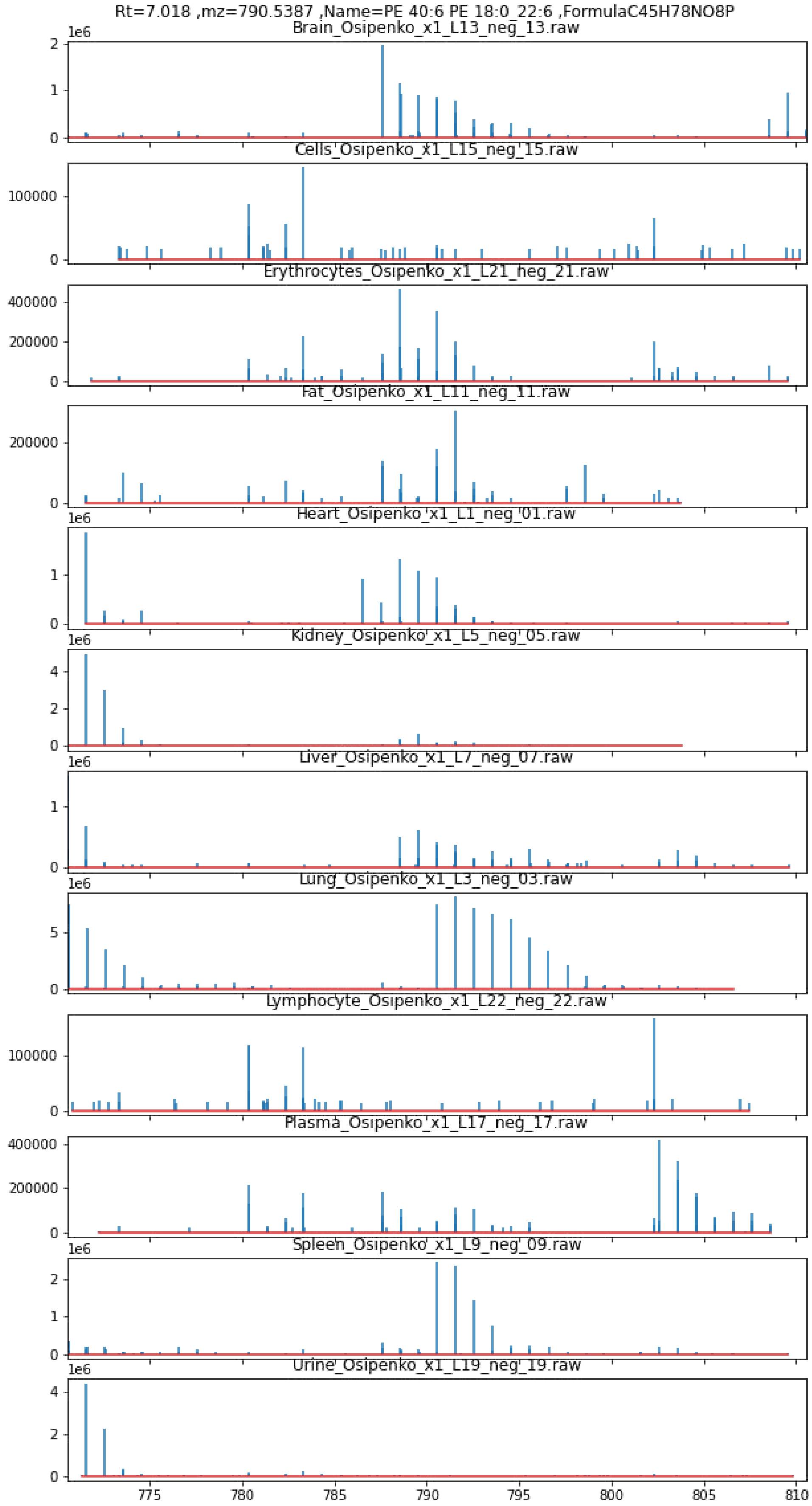
Rt=7.018 ,mz=790.5387 ,Name=PE 40:6 PE 18:0_22:6 ,FormulaC45H78NO8P
Brain_Osipenko_x1_L13_neg_13.raw
Cells_Osipenko_x1_L15_neg_15.raw
Erythrocytes_Osipenko_x1_L21_neg_21.raw
Fat_Osipenko_x1_L11_neg_11.raw
Heart_Osipenko_x1_L1_neg_01.raw
Kidney_Osipenko_x1_L5_neg_05.raw
Liver_Osipenko_x1_L7_neg_07.raw
Lung_Osipenko_x1_L3_neg_03.raw
Lymphocyte_Osipenko_x1_L22_neg_22.raw
Plasma_Osipenko_x1_L17_neg_17.raw
Spleen_Osipenko_x1_L9_neg_09.raw
Urine_Osipenko_x1_L19_neg_19.raw
1e6
775
780
785
790
795
800
805
810

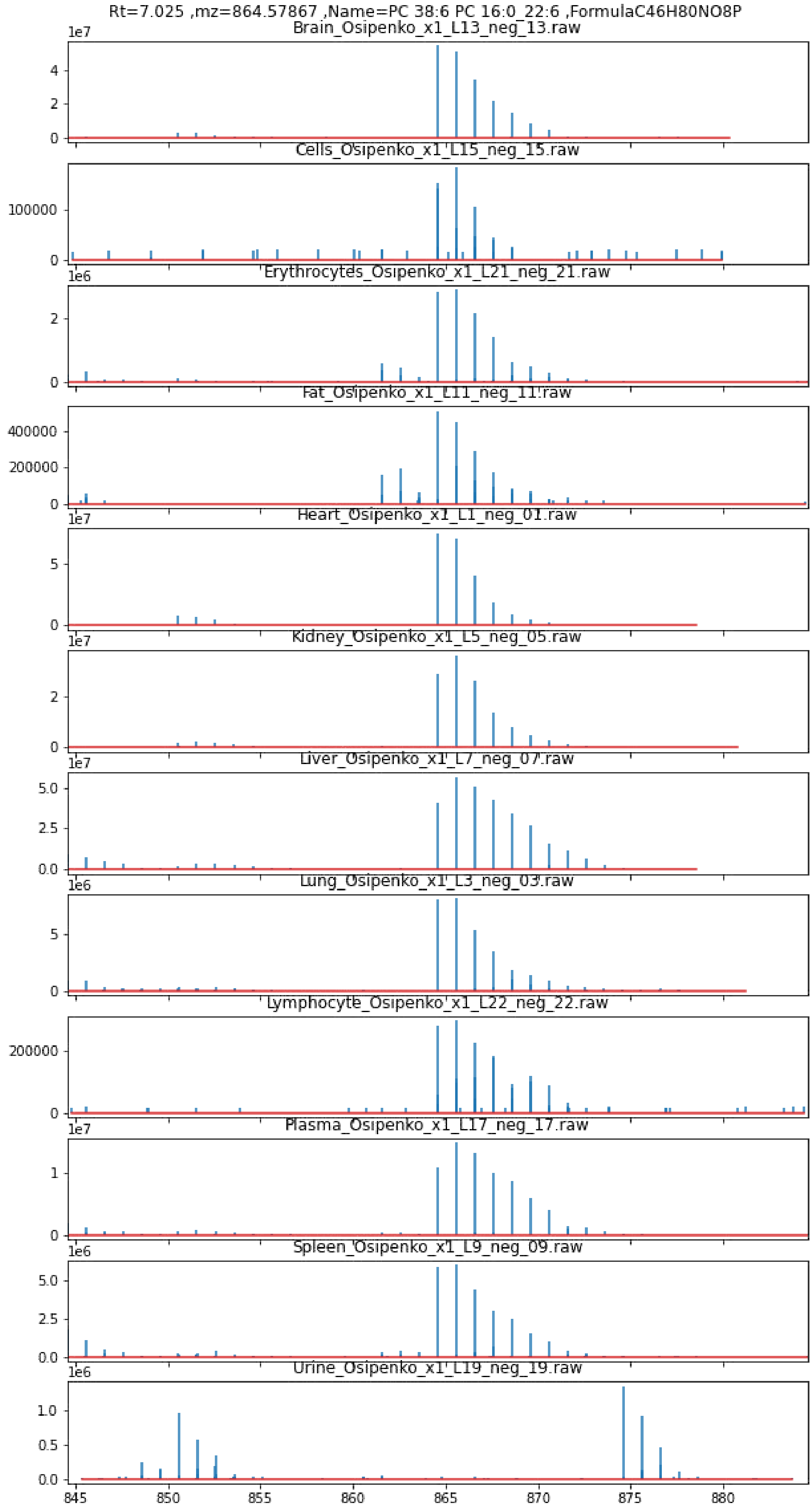

Rt=7.025 ,mz=864.57867 ,Name=PC 38:6 PC 16:0_22:6 ,FormulaC46H80NO8P
Brain_Osipenko_x1_L13_neg_13.raw
Cells_Osipenko_x1_L15_neg_15.raw
Erythrocytes_Osipenko_x1_L21_neg_21.raw
Fat_Osipenko_x1_L11_neg_11.raw
Heart_Osipenko_x1_L1_neg_01.raw
Kidney_Osipenko_x1_L5_neg_05.raw
Liver_Osipenko_x1_L7_neg_07.raw
Lung_Osipenko_x1_L3_neg_03.raw
Lymphocyte_Osipenko_x1_L22_neg_22.raw
Plasma_Osipenko_x1_L17_neg_17.raw
Spleen_Osipenko_x1_L9_neg_09.raw
Urine_Osipenko_x1_L19_neg_19.raw

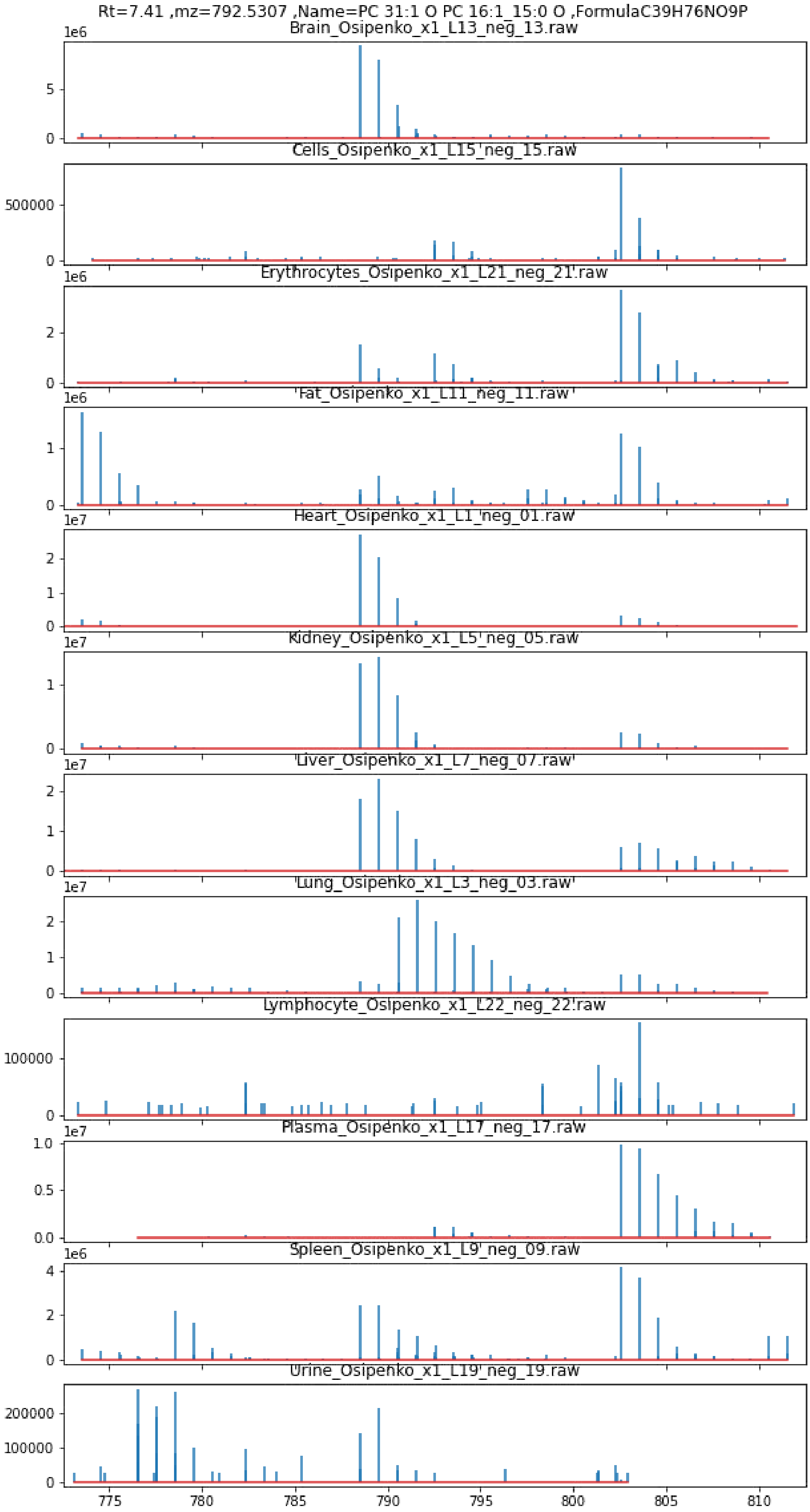

Rt=7.41 ,mz=792.5307 ,Name=PC 31:1 O PC 16:1_15:0 O ,FormulaC39H76NO9P
Brain_Osipenko_x1_L13_neg_13.raw
Cells_Osipenko_x1_L15_neg_15.raw
Erythrocytes_Osipenko_x1_L21_neg_21.raw
'Fat_Osipenko_x1_L11_neg_11.raw'
Heart_Osipenko_x1_L1_neg_01.raw
Kidney_Osipenko_x1_L5_neg_05.raw
'Liver_Osipenko_x1_L7_neg_07.raw'
'Lung_Osipenko_x1_L3_neg_03.raw'
Lymphocyte_Osipenko_x1_L22_neg_22.raw
Plasma_Osipenko_x1_L17_neg_17.raw
Spleen_Osipenko_x1_L9_neg_09.raw
Urine_Osipenko_x1_L19_neg_19.raw
775
780
785
790
795
800
805
810

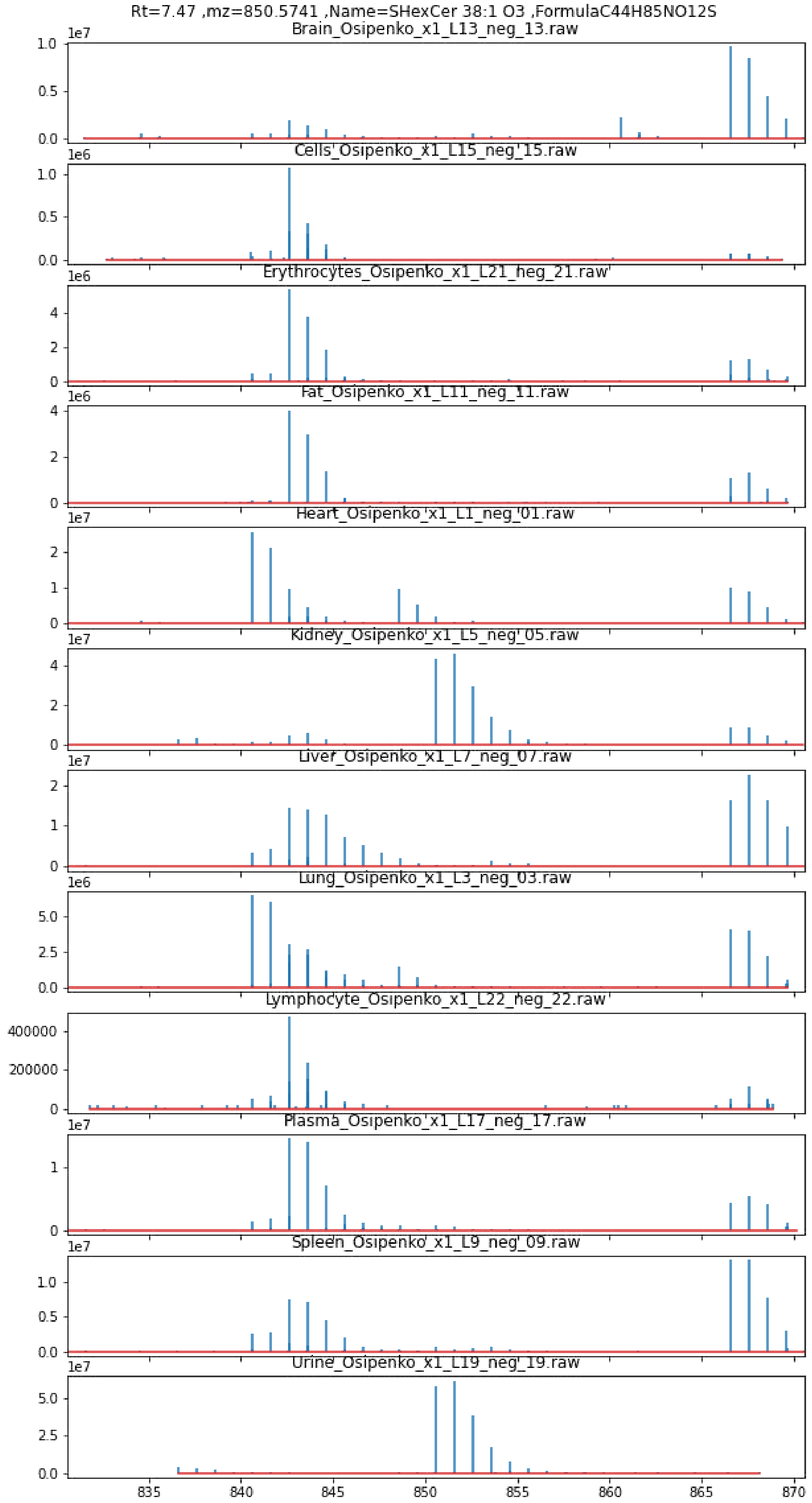

Rt=7.47 ,mz=850.5741 ,Name=SHexCer 38:1 O3 ,FormulaC44H85NO12S
Brain_Osipenko_x1_L13_neg_13.raw
Cells_Osipenko_x1_L15_neg_15.raw
Erythrocytes_Osipenko_x1_L21_neg_21.raw
Fat_Osipenko_x1_L11_neg_11.raw
Heart_Osipenko_x1_L1_neg_01.raw
Kidney_Osipenko_x1_L5_neg_05.raw
Liver_Osipenko_x1_L7_neg_07.raw
Lung_Osipenko_x1_L3_neg_03.raw
Lymphocyte_Osipenko_x1_L22_neg_22.raw
Plasma_Osipenko_x1_L17_neg_17.raw
Spleen_Osipenko_x1_L9_neg_09.raw
Urine_Osipenko_x1_L19_neg_19.raw
835
840
845
850
855
860
865
870

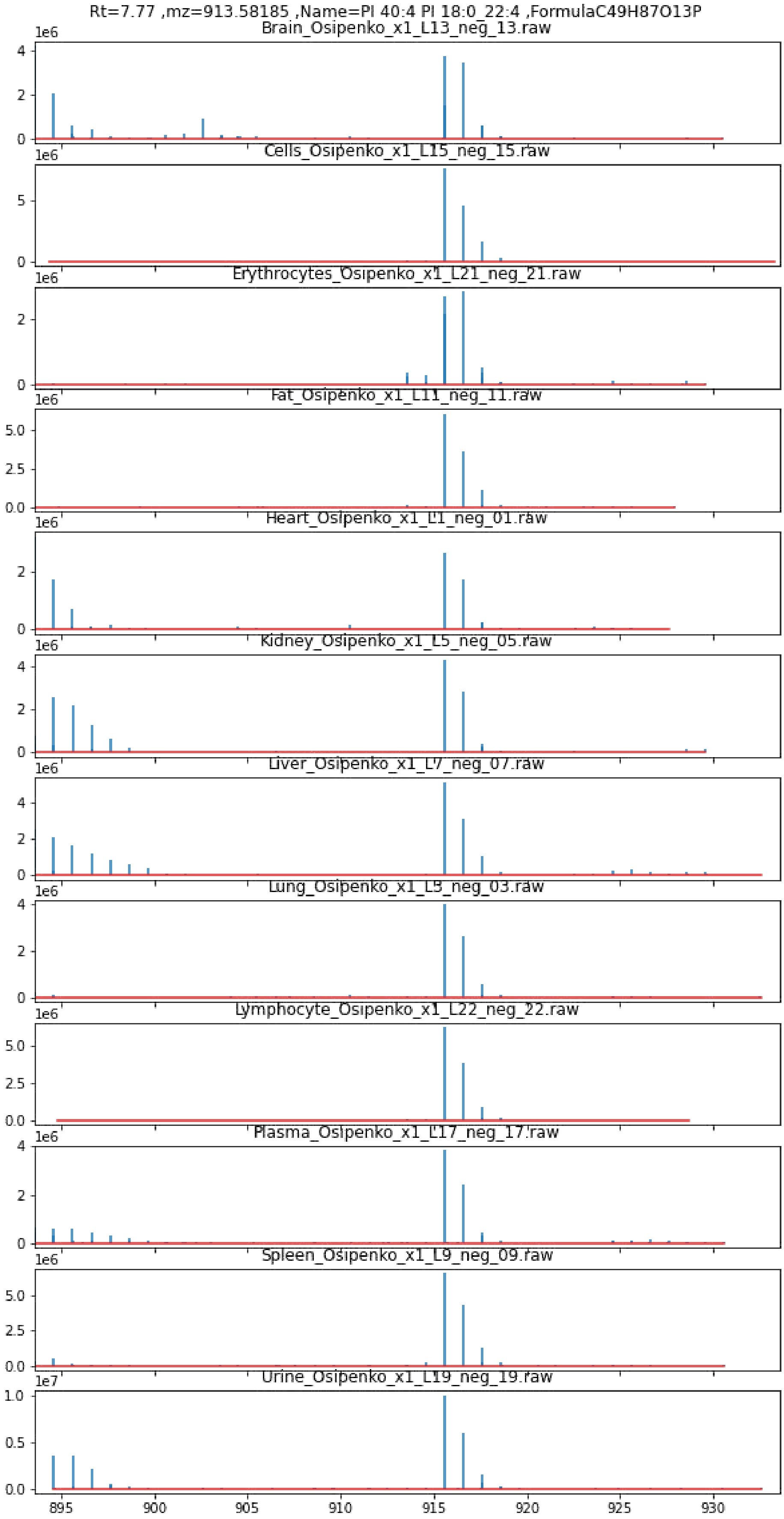
Rt=7.77 ,mz=913.58185 ,Name=PI 40:4 PI 18:0_22:4 ,FormulaC49H87O13P
Brain_Osipenko_x1_L13_neg_13.raw
Cells_Osipenko_x1_L15_neg_15.raw
Erythrocytes_Osipenko_x1_L21_neg_21.raw
Fat_Osipenko_x1_L11_neg_11.raw
Heart_Osipenko_x1_L1_neg_01.raw
Kidney_Osipenko_x1_L5_neg_05.raw
Liver_Osipenko_x1_L7_neg_07.raw
Lung_Osipenko_x1_L3_neg_03.raw
Lymphocyte_Osipenko_x1_L22_neg_22.raw
Plasma_Osipenko_x1_L17_neg_17.raw
Spleen_Osipenko_x1_L9_neg_09.raw
Urine_Osipenko_x1_L19_neg_19.raw
1e6
1e7
895
900
905
910
915
920
925
930

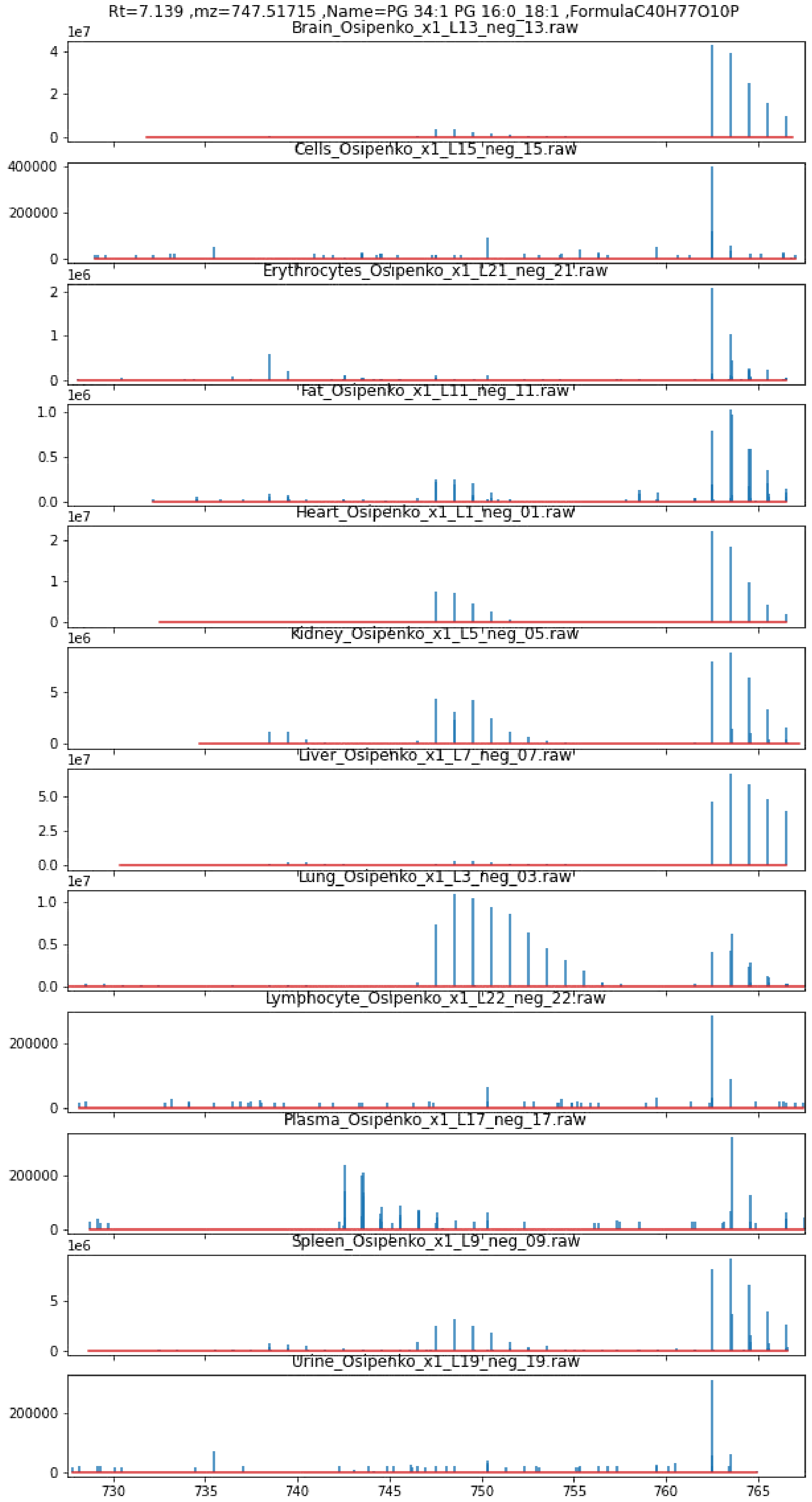

Rt=7.139 ,mz=747.51715 ,Name=PG 34:1 PG 16:0_18:1 ,FormulaC40H77O10P
Brain_Osipenko_x1_L13_neg_13.raw
Cells_Osipenko_x1_L15_neg_15.raw
Erythrocytes_Osipenko_x1_L21_neg_21.raw
Fat_Osipenko_x1_L11_neg_11.raw
Heart_Osipenko_x1_L1_neg_01.raw
Kidney_Osipenko_x1_L5_neg_05.raw
Liver_Osipenko_x1_L7_neg_07.raw
Lung_Osipenko_x1_L3_neg_03.raw
Lymphocyte_Osipenko_x1_L22_neg_22.raw
Plasma_Osipenko_x1_L17_neg_17.raw
Spleen_Osipenko_x1_L9_neg_09.raw
Urine_Osipenko_x1_L19_neg_19.raw
730
735
740
745
750
755
760
765

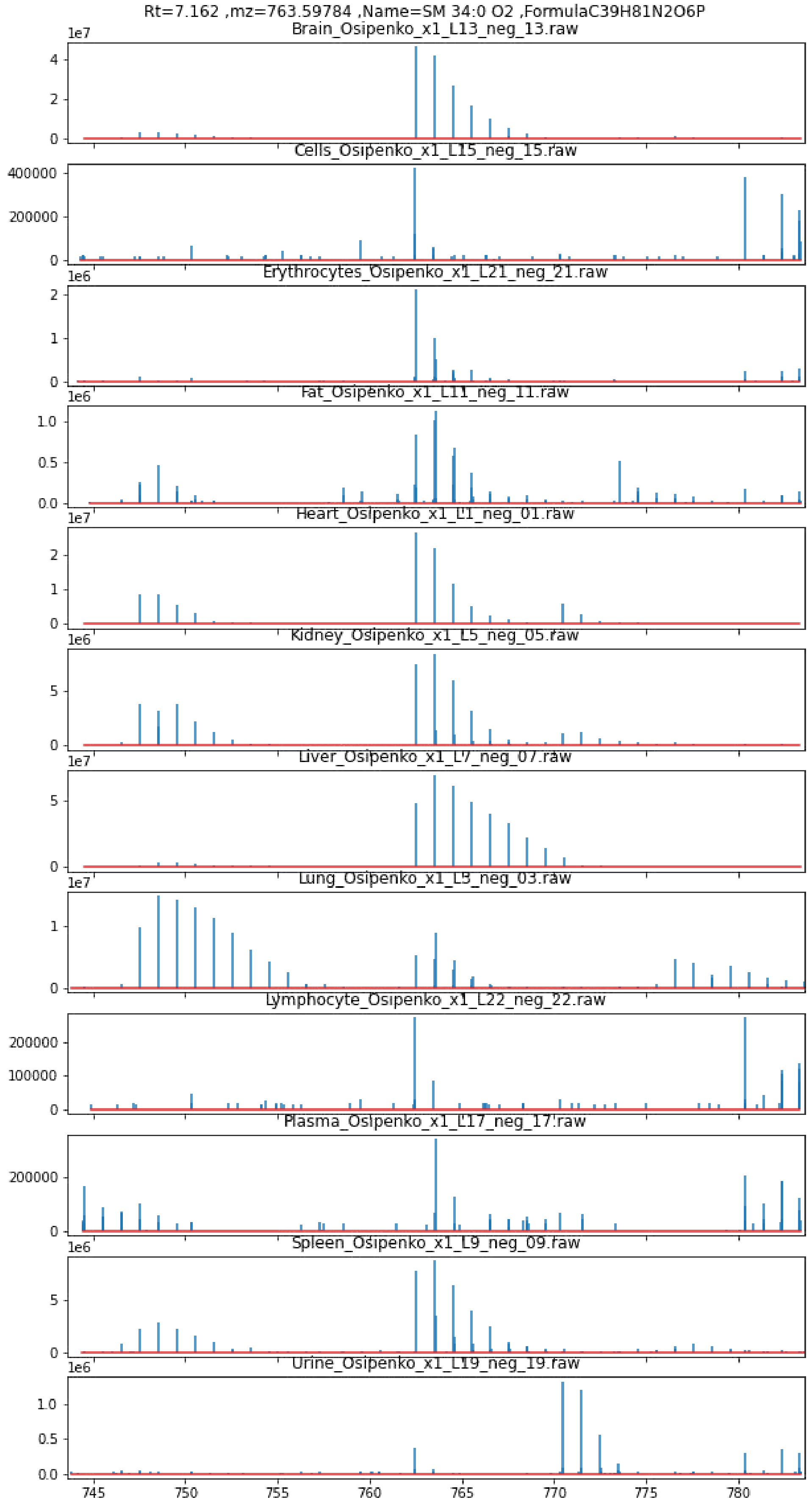

Rt=7.162 ,mz=763.59784 ,Name=SM 34:0 O2 ,FormulaC39H81N2O6P
Brain_Osipenko_x1_L13_neg_13.raw
Cells_Osipenko_x1_L15_neg_15.raw
Erythrocytes_Osipenko_x1_L21_neg_21.raw
Fat_Osipenko_x1_L11_neg_11.raw
Heart_Osipenko_x1_L1_neg_01.raw
Kidney_Osipenko_x1_L5_neg_05.raw
Liver_Osipenko_x1_L7_neg_07.raw
Lung_Osipenko_x1_L3_neg_03.raw
Lymphocyte_Osipenko_x1_L22_neg_22.raw
Plasma_Osipenko_x1_L17_neg_17.raw
Spleen_Osipenko_x1_L9_neg_09.raw
Urine_Osipenko_x1_L19_neg_19.raw

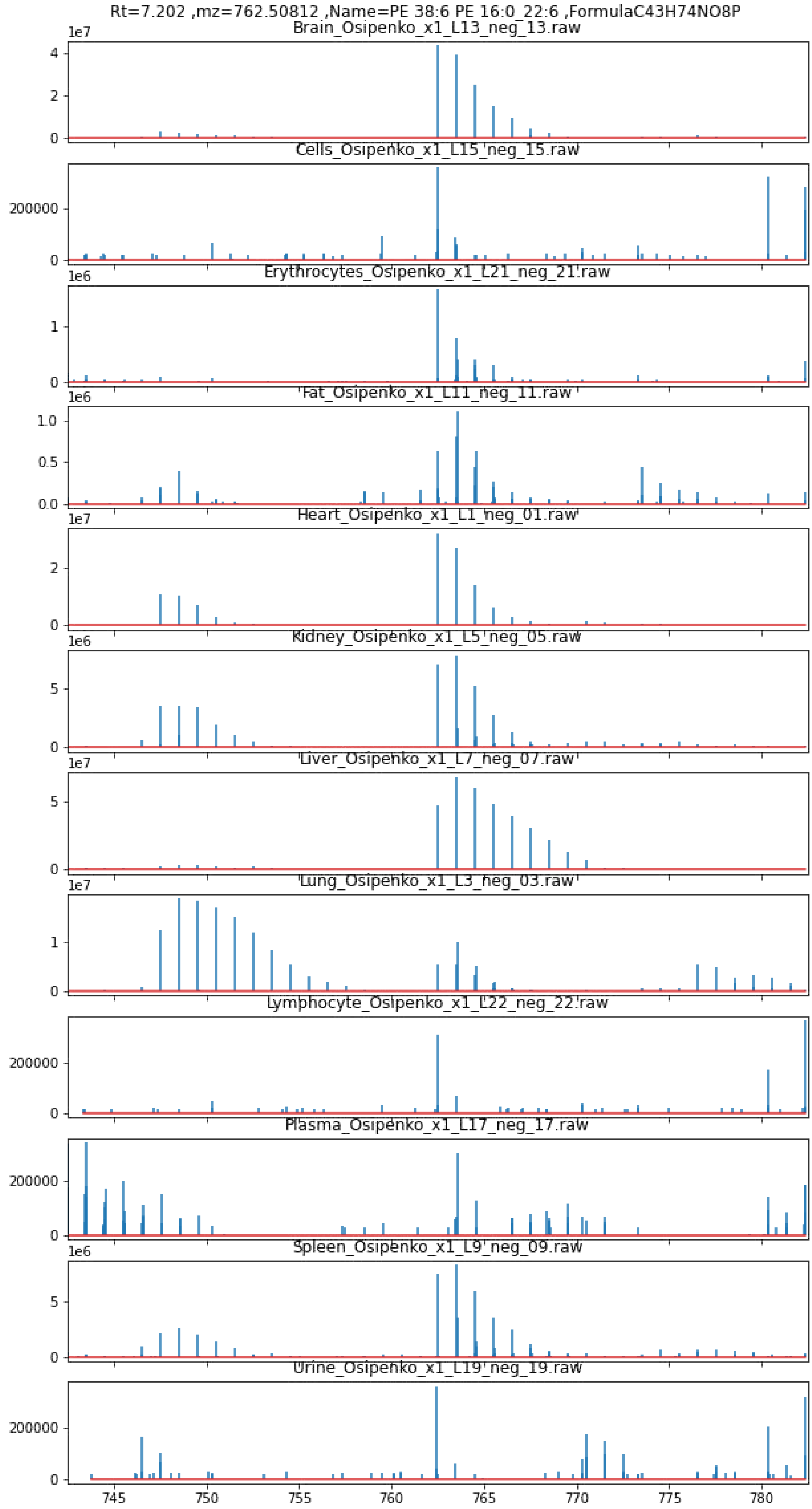

Rt=7.202 ,mz=762.50812 ,Name=PE 38:6 PE 16:0_22:6 ,FormulaC43H74NO8P
Brain_Osipenko_x1_L13_neg_13.raw
Cells_Osipenko_x1_L15_neg_15.raw
Erythrocytes_Osipenko_x1_L21_neg_21.raw
Fat_Osipenko_x1_L11_neg_11.raw
Heart_Osipenko_x1_L1_neg_01.raw
Kidney_Osipenko_x1_L5_neg_05.raw
Liver_Osipenko_x1_L7_neg_07.raw
Lung_Osipenko_x1_L3_neg_03.raw
Lymphocyte_Osipenko_x1_L22_neg_22.raw
Plasma_Osipenko_x1_L17_neg_17.raw
Spleen_Osipenko_x1_L9_neg_09.raw
Urine_Osipenko_x1_L19_neg_19.raw
745
750
755
760
765
770
775
780

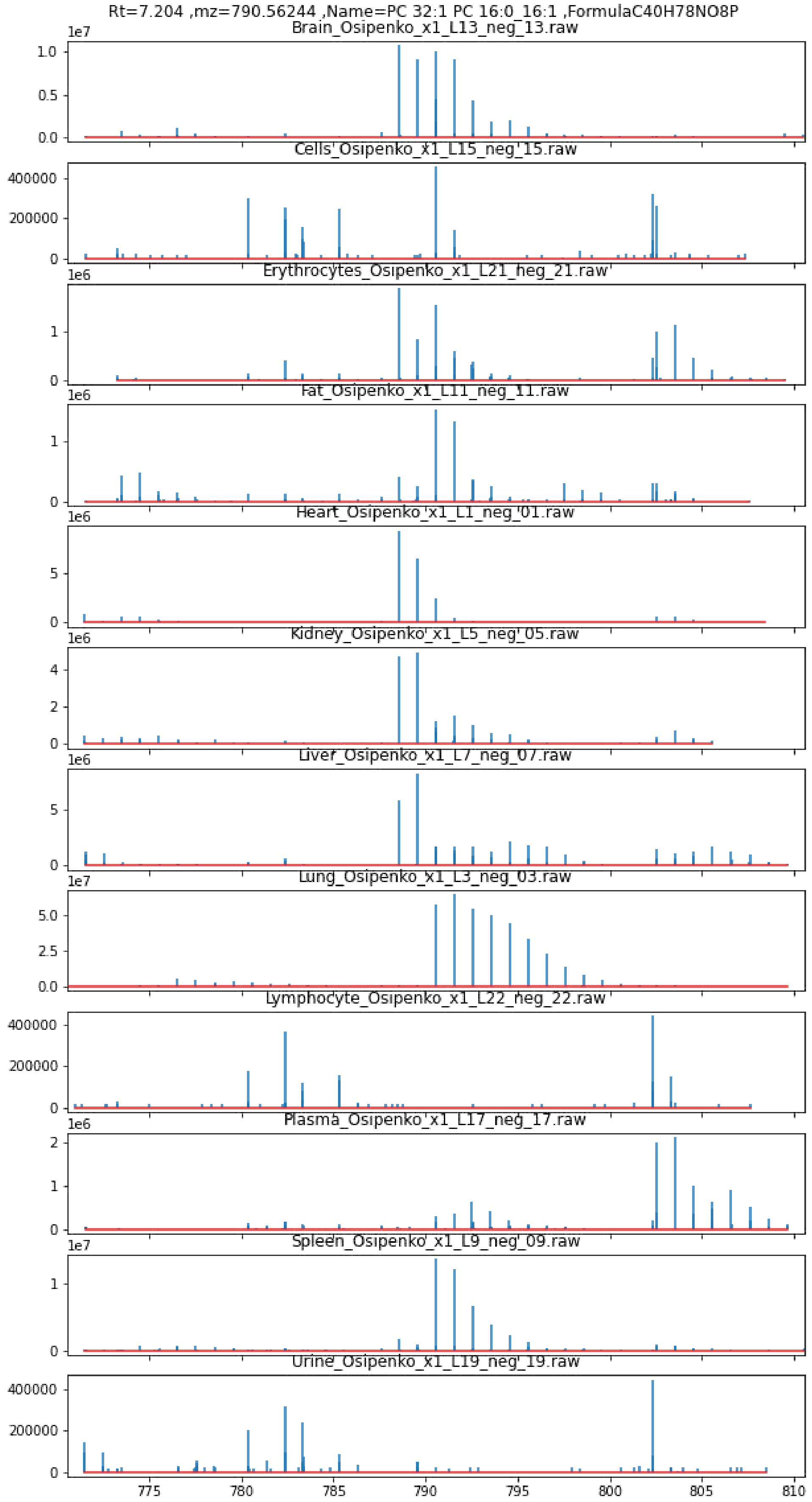
Rt=7.204 ,mz=790.56244 ,Name=PC 32:1 PC 16:0_16:1 ,FormulaC40H78NO8P
Brain_Osipenko_x1_L13_neg_13.raw
Cells_Osipenko_x1_L15_neg_15.raw
Erythrocytes_Osipenko_x1_L21_neg_21.raw
Fat_Osipenko_x1_L11_neg_11.raw
Heart_Osipenko_x1_L1_neg_01.raw
Kidney_Osipenko_x1_L5_neg_05.raw
Liver_Osipenko_x1_L7_neg_07.raw
Lung_Osipenko_x1_L3_neg_03.raw
Lymphocyte_Osipenko_x1_L22_neg_22.raw
Plasma_Osipenko_x1_L17_neg_17.raw
Spleen_Osipenko_x1_L9_neg_09.raw
Urine_Osipenko_x1_L19_neg_19.raw

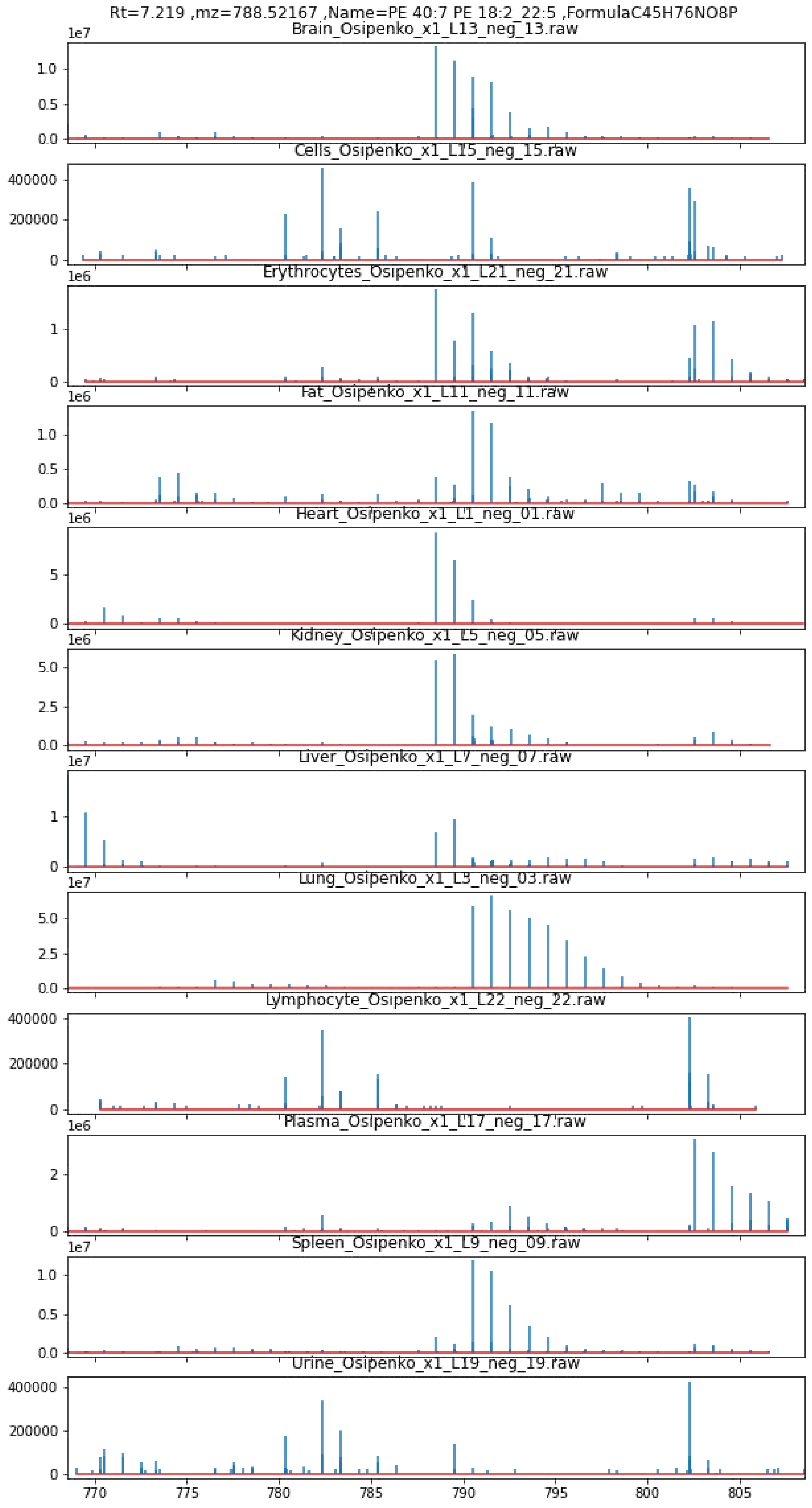
Rt=7.219 ,mz=788.52167 ,Name=PE 40:7 PE 18:2_22:5 ,FormulaC45H76NO8P
Brain_Osipenko_x1_L13_neg_13.raw
Cells_Osipenko_x1_L15_neg_15.raw
Erythrocytes_Osipenko_x1_L21_neg_21.raw
Fat_Osipenko_x1_L11_neg_11.raw
Heart_Osipenko_x1_L1_neg_01.raw
Kidney_Osipenko_x1_L5_neg_05.raw
Liver_Osipenko_x1_L7_neg_07.raw
Lung_Osipenko_x1_L3_neg_03.raw
Lymphocyte_Osipenko_x1_L22_neg_22.raw
Plasma_Osipenko_x1_L17_neg_17.raw
Spleen_Osipenko_x1_L9_neg_09.raw
Urine_Osipenko_x1_L19_neg_19.raw
770
775
780
785
790
795
800
805

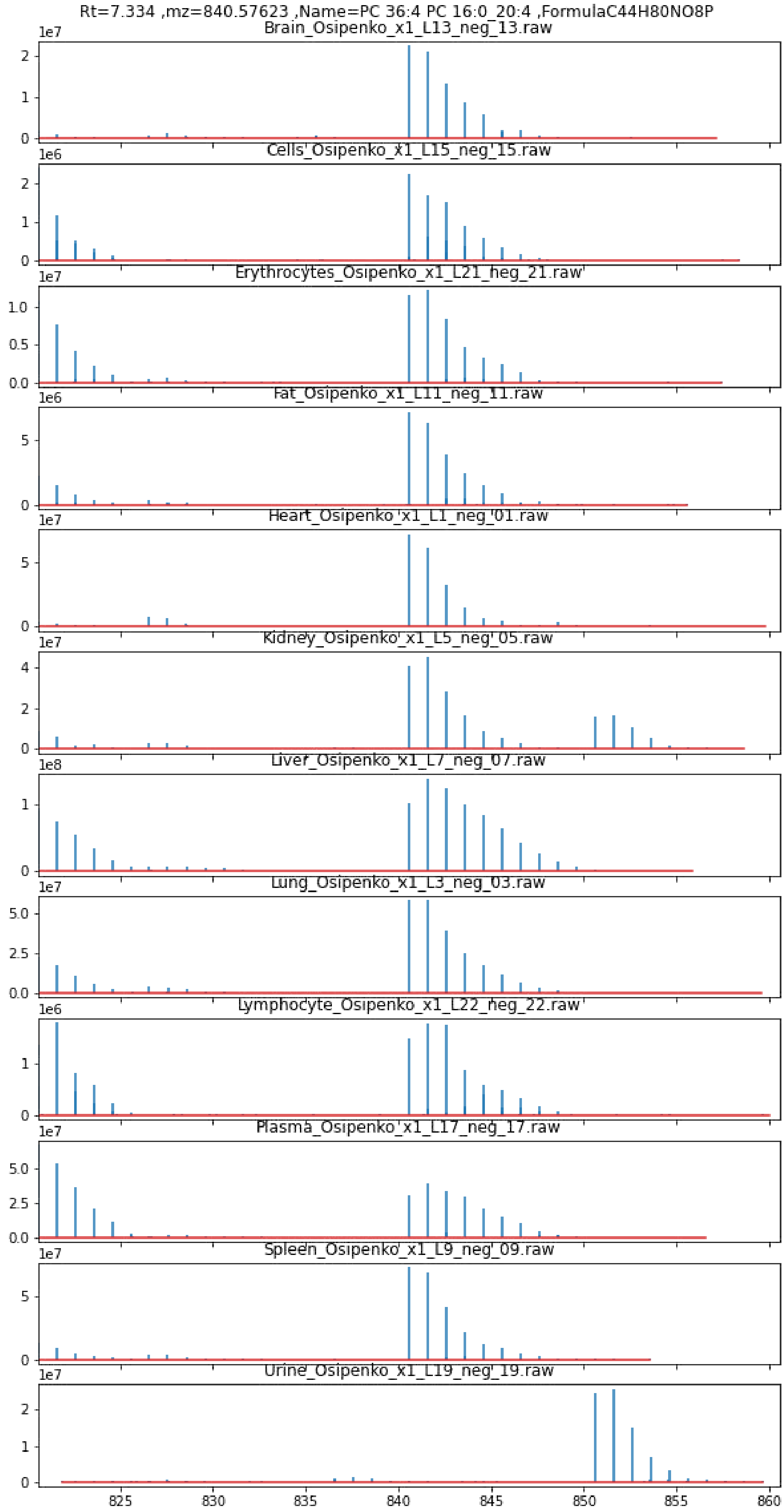

Rt=7.334 ,mz=840.57623 ,Name=PC 36:4 PC 16:0_20:4 ,FormulaC44H80NO8P
Brain_Osipenko_x1_L13_neg_13.raw
Cells_Osipenko_x1_L15_neg_15.raw
Erythrocytes_Osipenko_x1_L21_neg_21.raw
Fat_Osipenko_x1_L11_neg_11.raw
Heart_Osipenko_x1_L1_neg_01.raw
Kidney_Osipenko_x1_L5_neg_05.raw
Liver_Osipenko_x1_L7_neg_07.raw
Lung_Osipenko_x1_L3_neg_03.raw
Lymphocyte_Osipenko_x1_L22_neg_22.raw
Plasma_Osipenko_x1_L17_neg_17.raw
Spleen_Osipenko_x1_L9_neg_09.raw
Urine_Osipenko_x1_L19_neg_19.raw
825
830
835
840
845
850
855
860

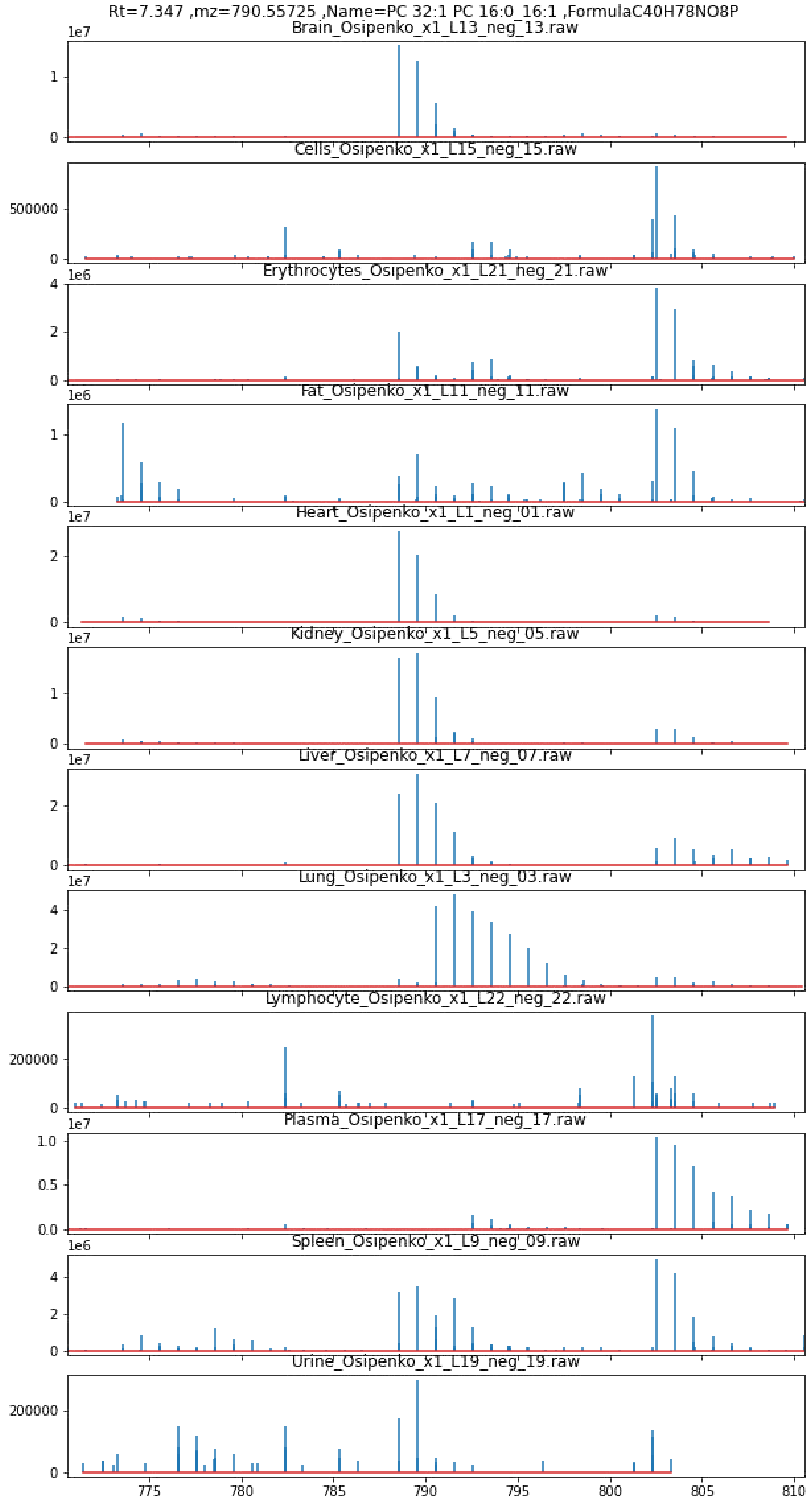

Rt=7.347 ,mz=790.55725 ,Name=PC 32:1 PC 16:0_16:1 ,FormulaC40H78NO8P
Brain_Osipenko_x1_L13_neg_13.raw
Cells_Osipenko_x1_L15_neg_15.raw
Erythrocytes_Osipenko_x1_L21_neg_21.raw
Fat_Osipenko_x1_L11_neg_11.raw
Heart_Osipenko_x1_L1_neg_01.raw
Kidney_Osipenko_x1_L5_neg_05.raw
Liver_Osipenko_x1_L7_neg_07.raw
Lung_Osipenko_x1_L3_neg_03.raw
Lymphocyte_Osipenko_x1_L22_neg_22.raw
Plasma_Osipenko_x1_L17_neg_17.raw
Spleen_Osipenko_x1_L9_neg_09.raw
Urine_Osipenko_x1_L19_neg_19.raw
775
780
785
790
795
800
805
810

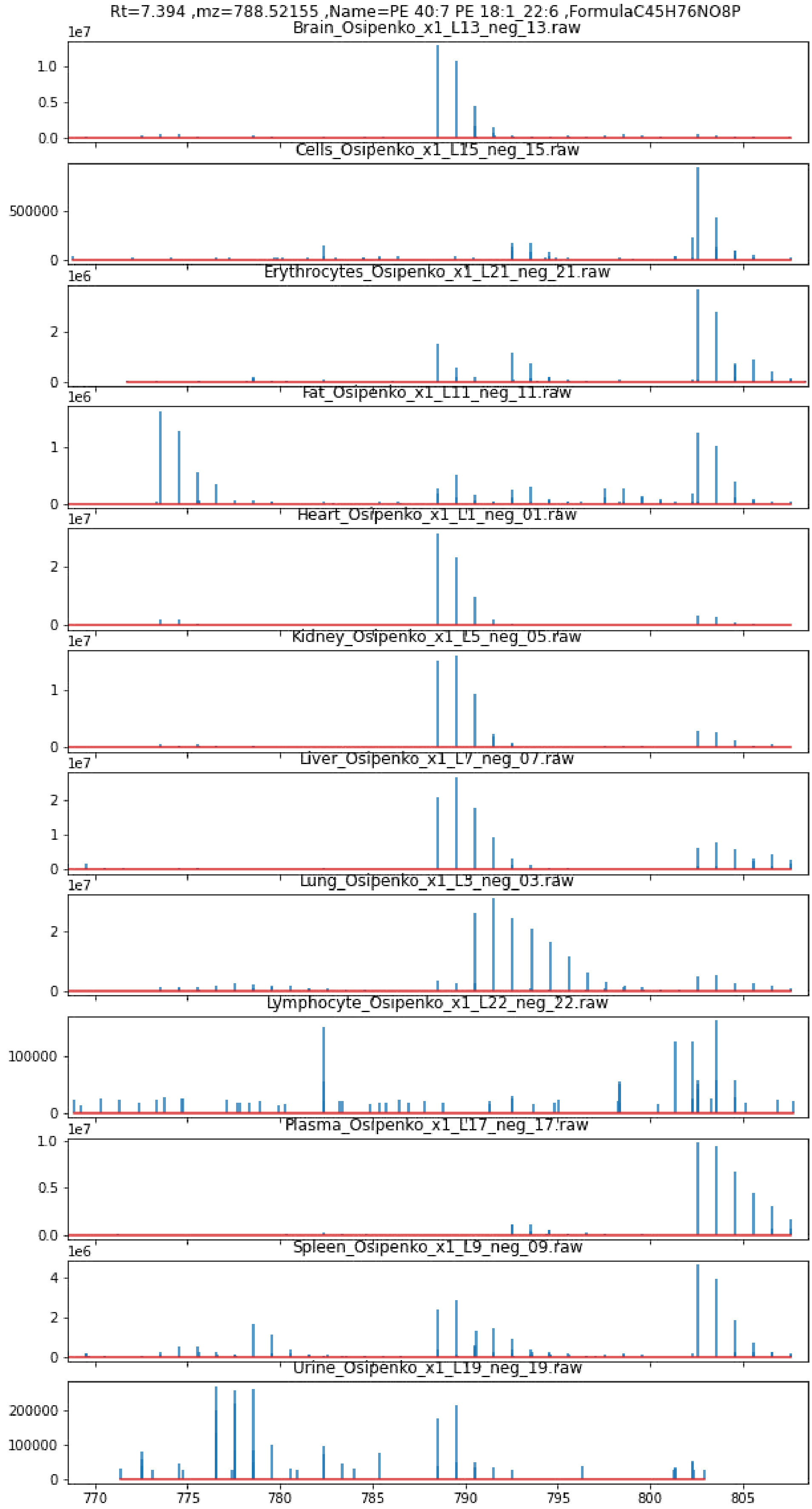

Rt=7.394 ,mz=788.52155 ,Name=PE 40:7 PE 18:1_22:6 ,FormulaC45H76NO8P
Brain_Osipenko_x1_L13_neg_13.raw
Cells_Osipenko_x1_L15_neg_15.raw
Erythrocytes_Osipenko_x1_L21_neg_21.raw
Fat_Osipenko_x1_L11_neg_11.raw
Heart_Osipenko_x1_L1_neg_01.raw
Kidney_Osipenko_x1_L5_neg_05.raw
Liver_Osipenko_x1_L7_neg_07.raw
Lung_Osipenko_x1_L3_neg_03.raw
Lymphocyte_Osipenko_x1_L22_neg_22.raw
Plasma_Osipenko_x1_L17_neg_17.raw
Spleen_Osipenko_x1_L9_neg_09.raw
Urine_Osipenko_x1_L19_neg_19.raw
1e7
1e6
770
775
780
785
790
795
800
805

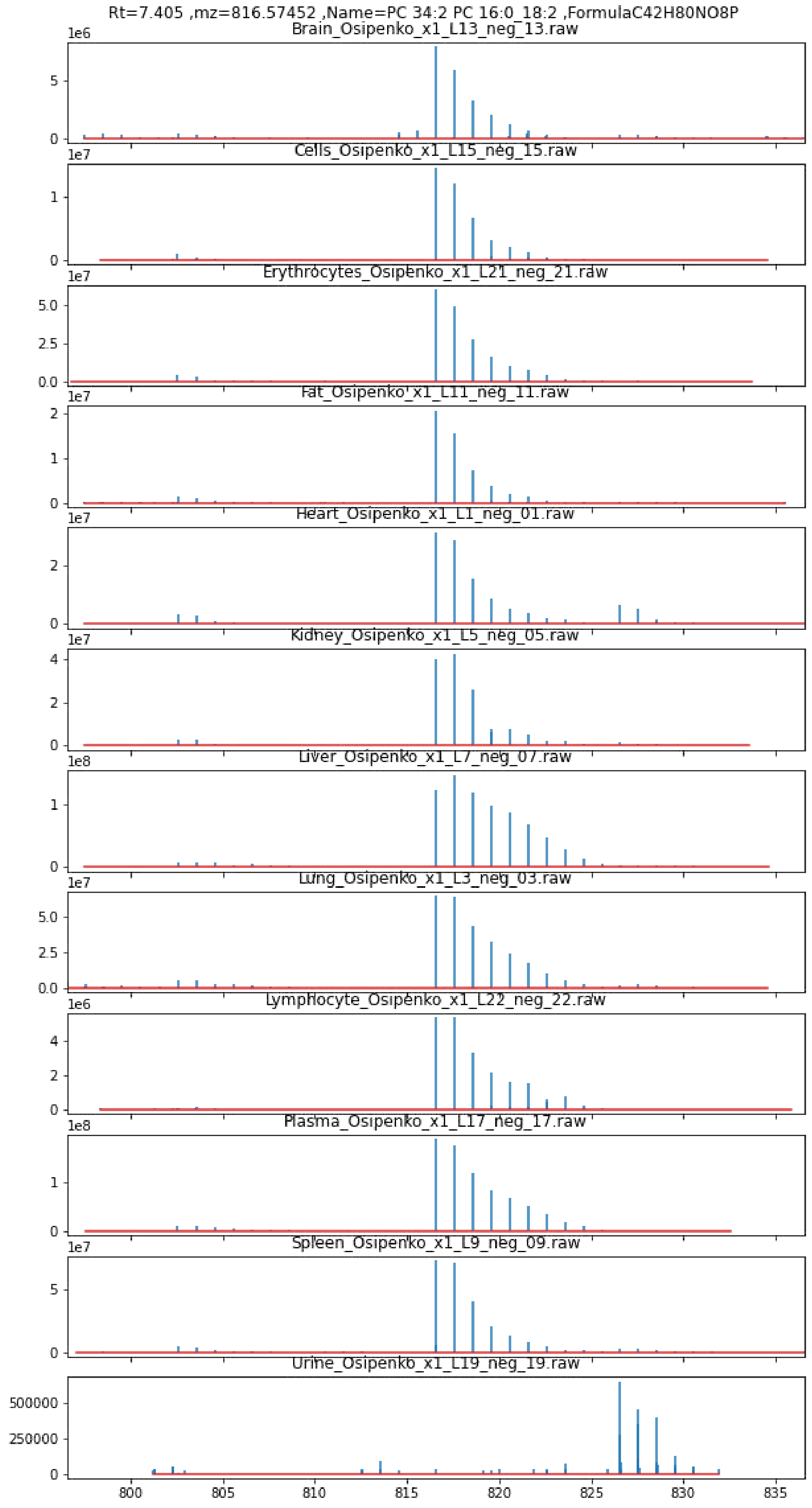

Rt=7.405 ,mz=816.57452 ,Name=PC 34:2 PC 16:0_18:2 ,FormulaC42H80NO8P
Brain_Osipenko_x1_L13_neg_13.raw
Cells_Osipenko_x1_L15_neg_15.raw
Erythrocytes_Osipenko_x1_L21_neg_21.raw
Fat_Osipenko_x1_L11_neg_11.raw
Heart_Osipenko_x1_L1_neg_01.raw
Kidney_Osipenko_x1_L5_neg_05.raw
Liver_Osipenko_x1_L7_neg_07.raw
Lung_Osipenko_x1_L3_neg_03.raw
Lymphocyte_Osipenko_x1_L22_neg_22.raw
Plasma_Osipenko_x1_L17_neg_17.raw
Spleen_Osipenko_x1_L9_neg_09.raw
Urine_Osipenko_x1_L19_neg_19.raw
800
805
810
815
820
825
830
835

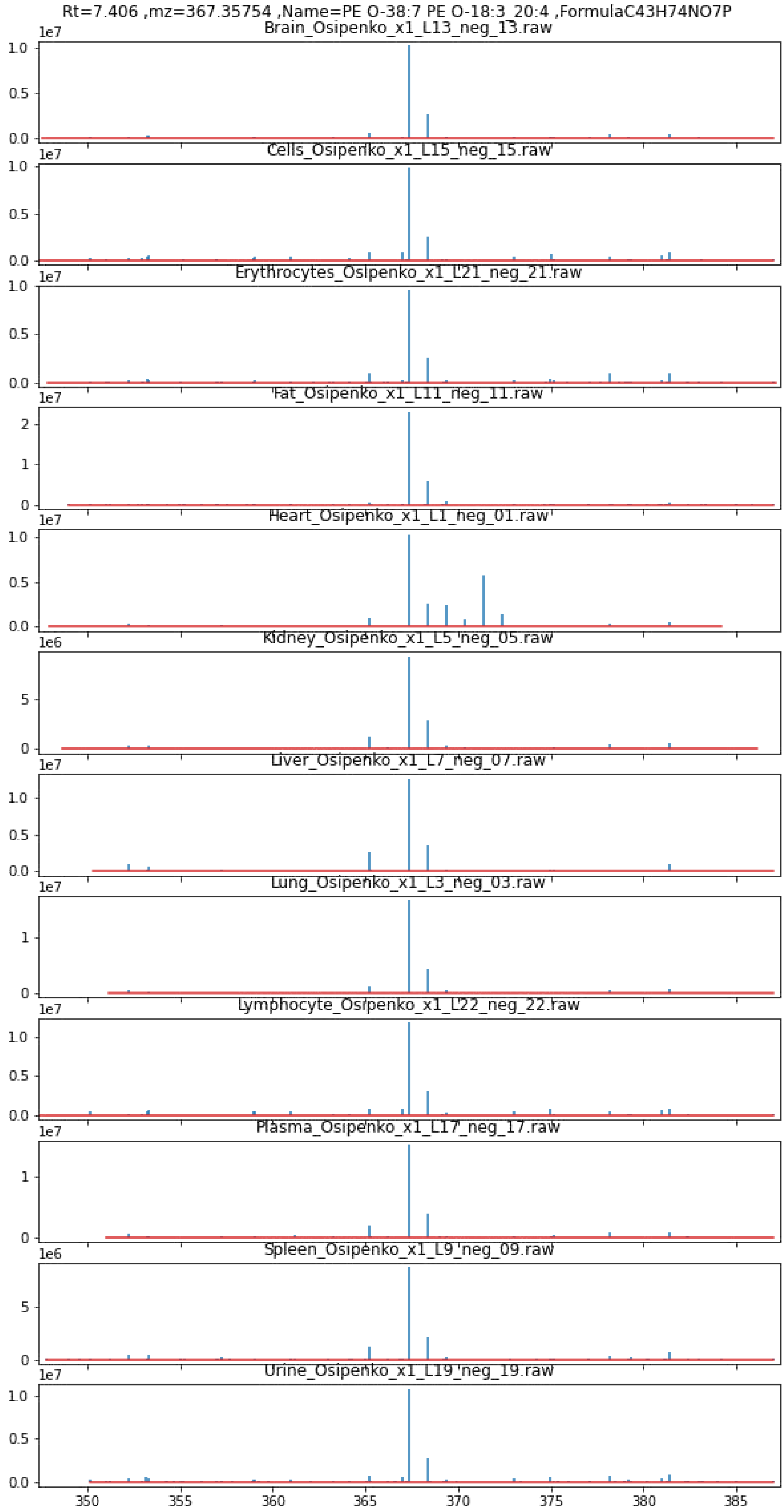
Rt=7.406 ,mz=367.35754 ,Name=PE O-38:7 PE O-18:3_20:4 ,FormulaC43H74NO7P
Brain_Osipenko_x1_L13_neg_13.raw
Cells_Osipenko_x1_L15_neg_15.raw
Erythrocytes_Osipenko_x1_L21_neg_21.raw
Fat_Osipenko_x1_L11_neg_11.raw
Heart_Osipenko_x1_L1_neg_01.raw
Kidney_Osipenko_x1_L5_neg_05.raw
Liver_Osipenko_x1_L7_neg_07.raw
Lung_Osipenko_x1_L3_neg_03.raw
Lymphocyte_Osipenko_x1_L22_neg_22.raw
Plasma_Osipenko_x1_L17_neg_17.raw
Spleen_Osipenko_x1_L9_neg_09.raw
Urine_Osipenko_x1_L19_neg_19.raw
350
355
360
365
370
375
380
385

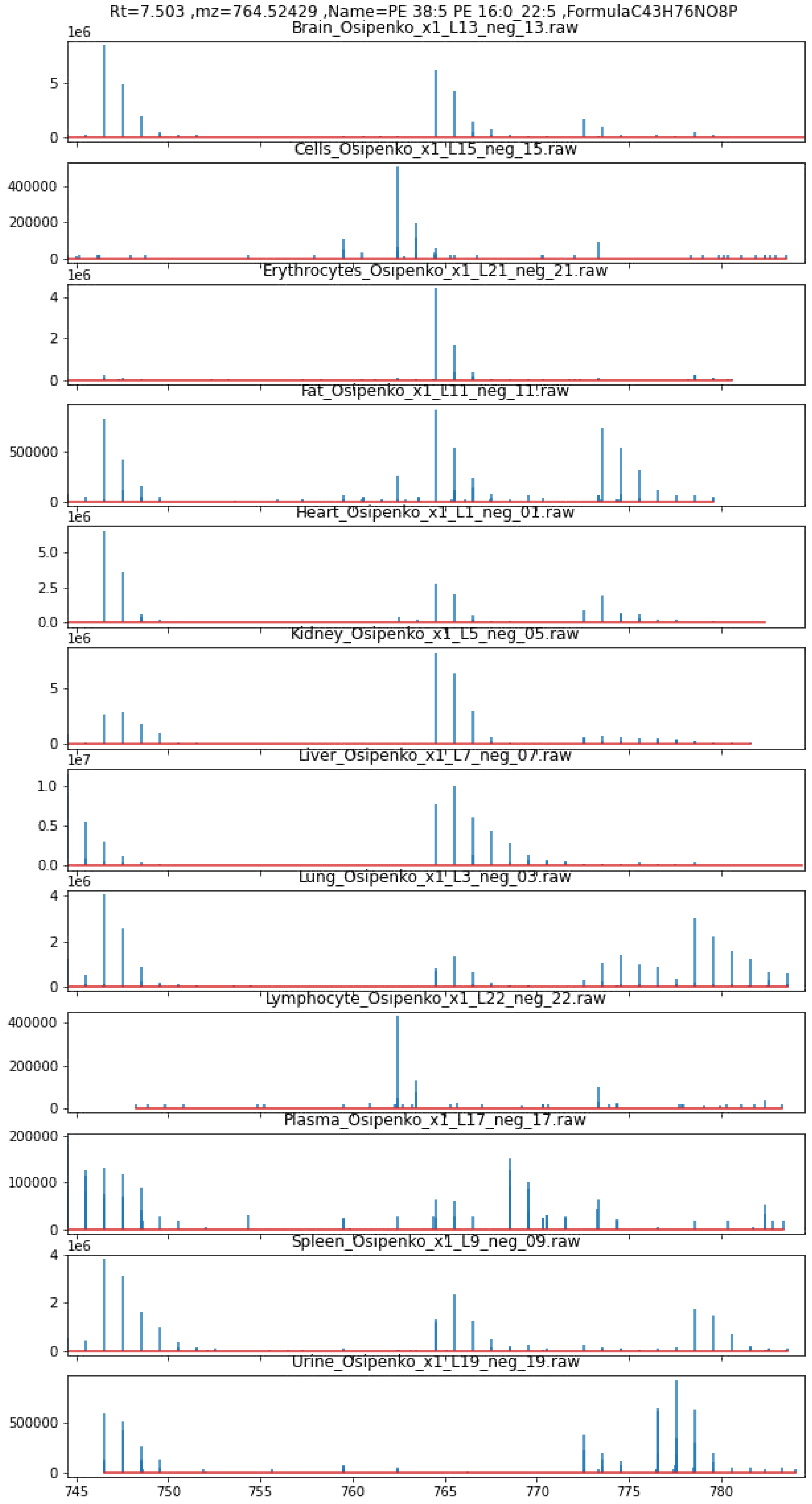
Rt=7.503 ,mz=764.52429 ,Name=PE 38:5 PE 16:0_22:5 ,FormulaC43H76NO8P
Brain_Osipenko_x1_L13_neg_13.raw
Cells_Osipenko_x1_L15_neg_15.raw
Erythrocytes_Osipenko_x1_L21_neg_21.raw
Fat_Osipenko_x1_L11_neg_11.raw
Heart_Osipenko_x1_L1_neg_01.raw
Kidney_Osipenko_x1_L5_neg_05.raw
Liver_Osipenko_x1_L7_neg_07.raw
Lung_Osipenko_x1_L3_neg_03.raw
Lymphocyte_Osipenko_x1_L22_neg_22.raw
Plasma_Osipenko_x1_L17_neg_17.raw
Spleen_Osipenko_x1_L9_neg_09.raw
Urine_Osipenko_x1_L19_neg_19.raw

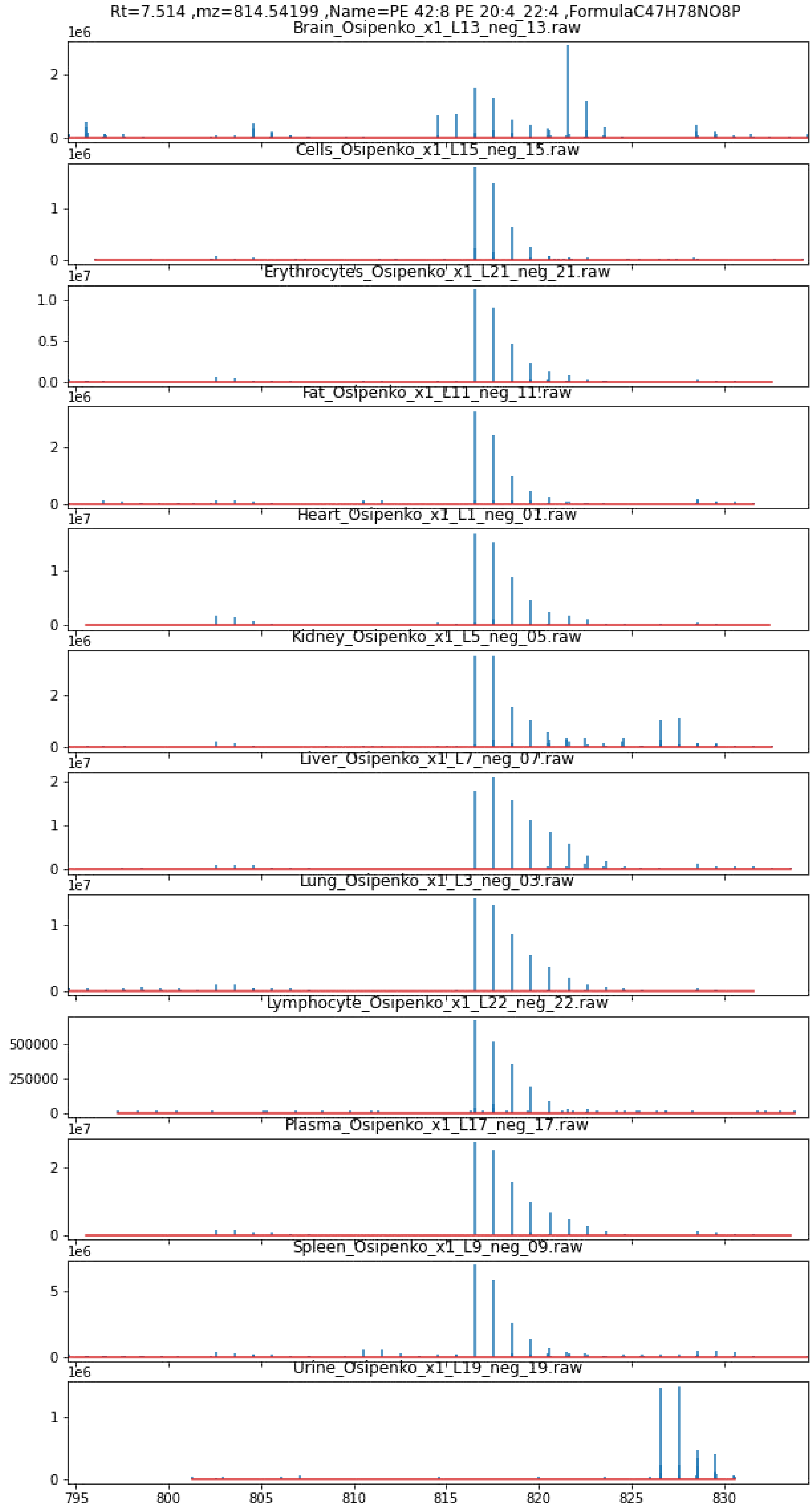
Rt=7.514 ,mz=814.54199 ,Name=PE 42:8 PE 20:4_22:4 ,FormulaC47H78NO8P
Brain_Osipenko_x1_L13_neg_13.raw
Cells_Osipenko_x1_L15_neg_15.raw
Erythrocytes_Osipenko_x1_L21_neg_21.raw
Fat_Osipenko_x1_L11_neg_11.raw
Heart_Osipenko_x1_L1_neg_01.raw
Kidney_Osipenko_x1_L5_neg_05.raw
Liver_Osipenko_x1_L7_neg_07.raw
Lung_Osipenko_x1_L3_neg_03.raw
Lymphocyte_Osipenko_x1_L22_neg_22.raw
Plasma_Osipenko_x1_L17_neg_17.raw
Spleen_Osipenko_x1_L9_neg_09.raw
Urine_Osipenko_x1_L19_neg_19.raw
1e6
1e7
500000
250000
795
800
805
810
815
820
825
830

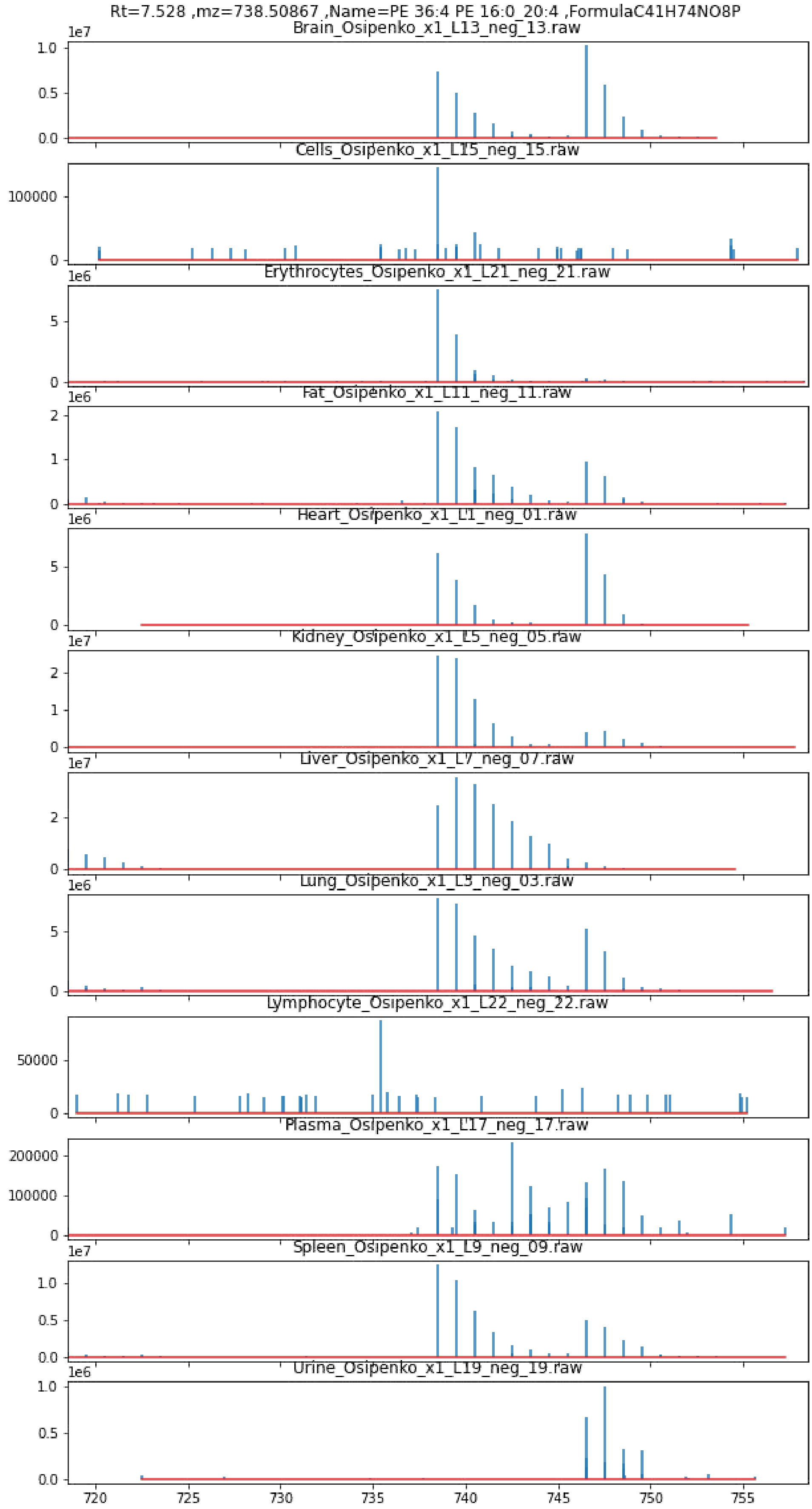

Rt=7.528 ,mz=738.50867 ,Name=PE 36:4 PE 16:0_20:4 ,FormulaC41H74NO8P
Brain_Osipenko_x1_L13_neg_13.raw
Cells_Osipenko_x1_L15_neg_15.raw
Erythrocytes_Osipenko_x1_L21_neg_21.raw
Fat_Osipenko_x1_L11_neg_11.raw
Heart_Osipenko_x1_L1_neg_01.raw
Kidney_Osipenko_x1_L5_neg_05.raw
Liver_Osipenko_x1_L7_neg_07.raw
Lung_Osipenko_x1_L3_neg_03.raw
Lymphocyte_Osipenko_x1_L22_neg_22.raw
Plasma_Osipenko_x1_L17_neg_17.raw
Spleen_Osipenko_x1_L9_neg_09.raw
Urine_Osipenko_x1_L19_neg_19.raw
720
725
730
735
740
745
750
755

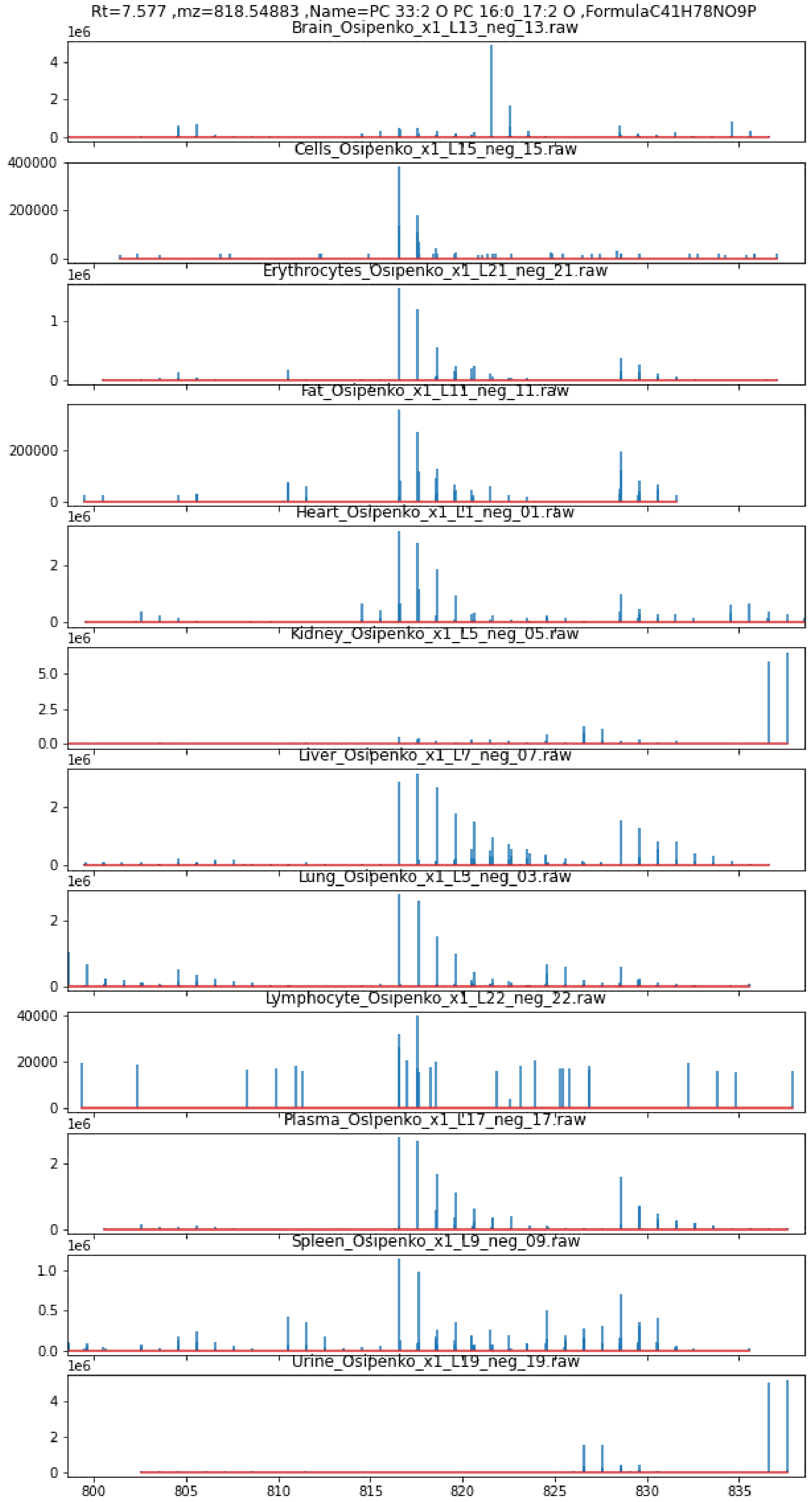
Rt=7.577 ,mz=818.54883 ,Name=PC 33:2 O PC 16:0_17:2 O ,FormulaC41H78NO9P
Brain_Osipenko_x1_L13_neg_13.raw
Cells_Osipenko_x1_L15_neg_15.raw
Erythrocytes_Osipenko_x1_L21_neg_21.raw
Fat_Osipenko_x1_L11_neg_11.raw
Heart_Osipenko_x1_L1_neg_01.raw
Kidney_Osipenko_x1_L5_neg_05.raw
Liver_Osipenko_x1_L7_neg_07.raw
Lung_Osipenko_x1_L3_neg_03.raw
Lymphocyte_Osipenko_x1_L22_neg_22.raw
Plasma_Osipenko_x1_L17_neg_17.raw
Spleen_Osipenko_x1_L9_neg_09.raw
Urine_Osipenko_x1_L19_neg_19.raw
1e6
800
805
810
815
820
825
830
835

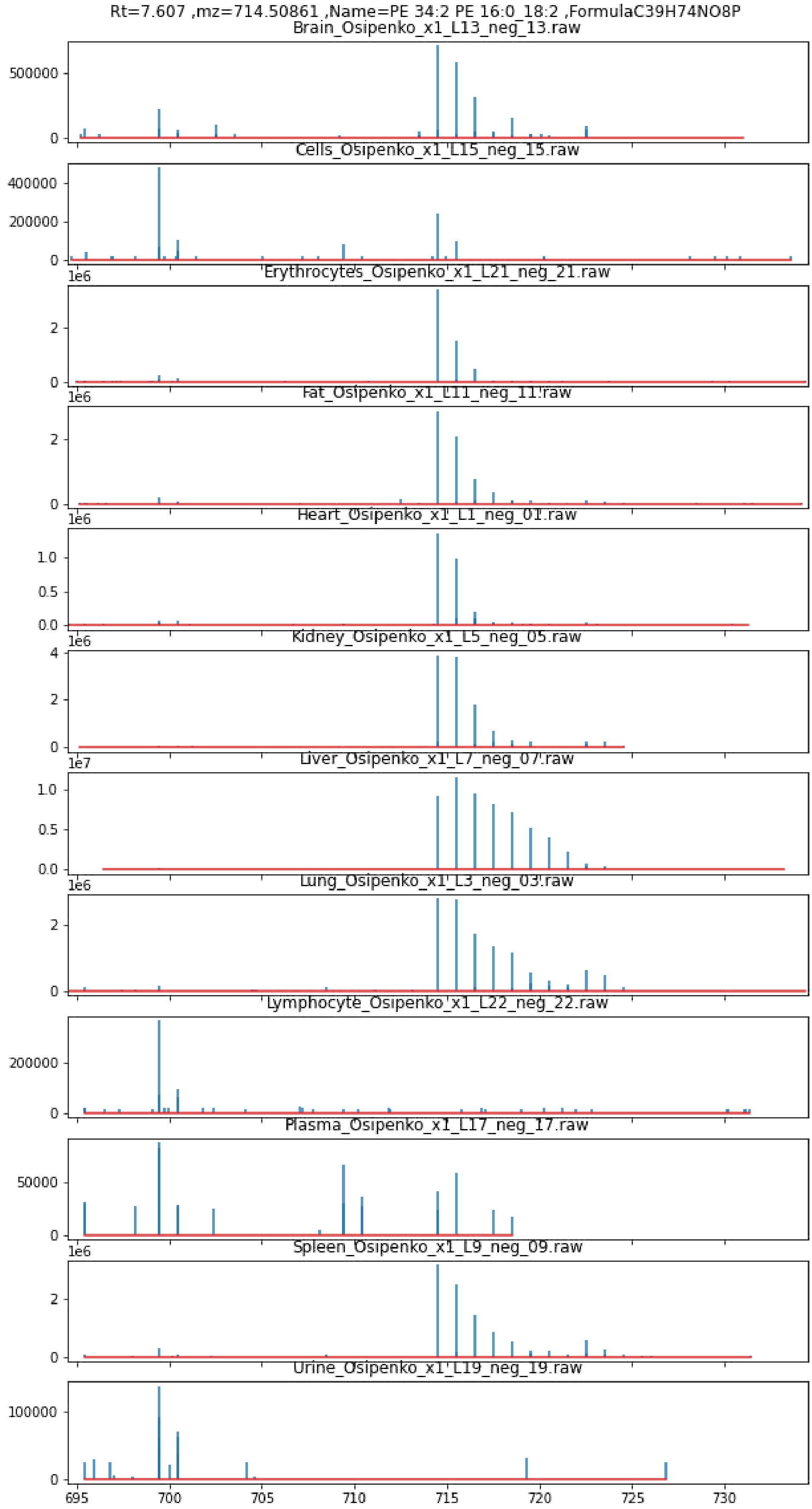

Rt=7.607 ,mz=714.50861 ,Name=PE 34:2 PE 16:0_18:2 ,FormulaC39H74NO8P
Brain_Osipenko_x1_L13_neg_13.raw
Cells_Osipenko_x1_L15_neg_15.raw
Erythrocytes_Osipenko_x1_L21_neg_21.raw
Fat_Osipenko_x1_L11_neg_11.raw
Heart_Osipenko_x1_L1_neg_01.raw
Kidney_Osipenko_x1_L5_neg_05.raw
Liver_Osipenko_x1_L7_neg_07.raw
Lung_Osipenko_x1_L3_neg_03.raw
Lymphocyte_Osipenko_x1_L22_neg_22.raw
Plasma_Osipenko_x1_L17_neg_17.raw
Spleen_Osipenko_x1_L9_neg_09.raw
Urine_Osipenko_x1_L19_neg_19.raw
695
700
705
710
715
720
725
730

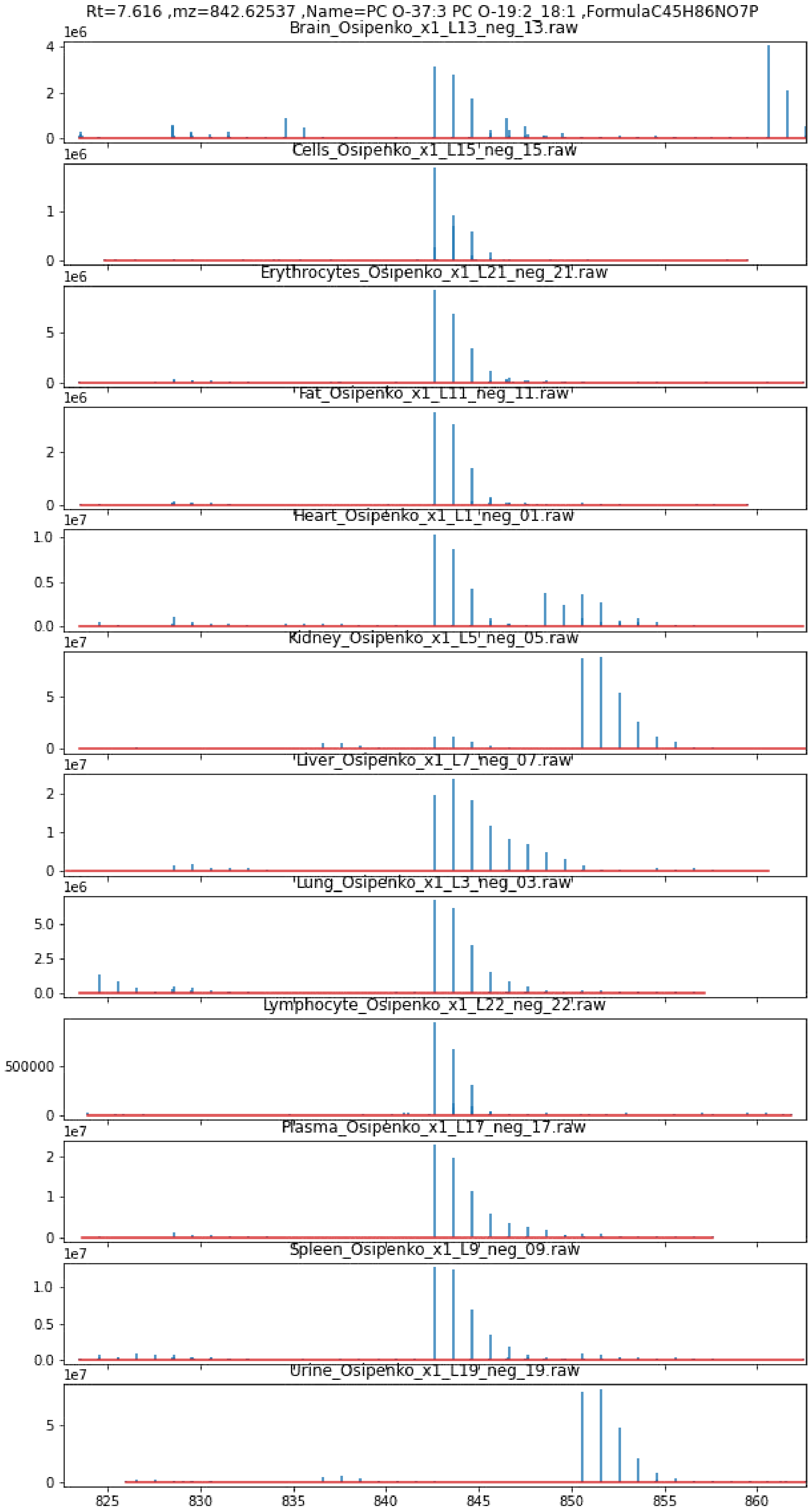

Rt=7.616 ,mz=842.62537 ,Name=PC O-37:3 PC O-19:2_18:1 ,FormulaC45H86NO7P
Brain_Osipenko_x1_L13_neg_13.raw
Cells_Osipenko_x1_L15_neg_15.raw
Erythrocytes_Osipenko_x1_L21_neg_21.raw
Fat_Osipenko_x1_L11_neg_11.raw
Heart_Osipenko_x1_L1_neg_01.raw
Kidney_Osipenko_x1_L5_neg_05.raw
Liver_Osipenko_x1_L7_neg_07.raw
Lung_Osipenko_x1_L3_neg_03.raw
Lymphocyte_Osipenko_x1_L22_neg_22.raw
Plasma_Osipenko_x1_L17_neg_17.raw
Spleen_Osipenko_x1_L9_neg_09.raw
Urine_Osipenko_x1_L19_neg_19.raw
825
830
835
840
845
850
855
860

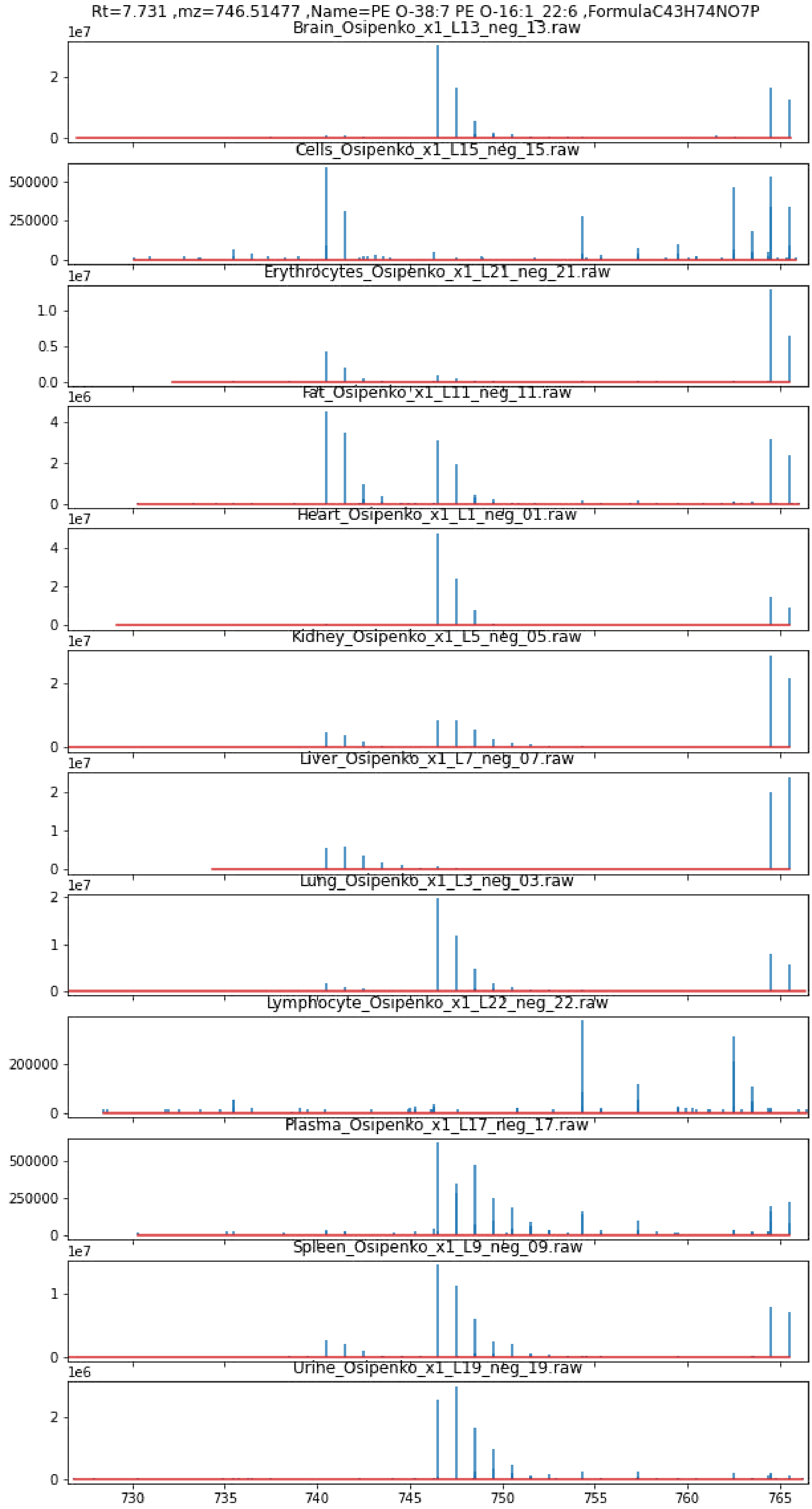
Rt=7.731 ,mz=746.51477 ,Name=PE O-38:7 PE O-16:1_22:6 ,FormulaC43H74NO7P
Brain_Osipenko_x1_L13_neg_13.raw
Cells_Osipenko_x1_L15_neg_15.raw
Erythrocytes_Osipenko_x1_L21_neg_21.raw
Fat_Osipenko_x1_L11_neg_11.raw
Heart_Osipenko_x1_L1_neg_01.raw
Kidney_Osipenko_x1_L5_neg_05.raw
Liver_Osipenko_x1_L7_neg_07.raw
Lung_Osipenko_x1_L3_neg_03.raw
Lymphocyte_Osipenko_x1_L22_neg_22.raw
Plasma_Osipenko_x1_L17_neg_17.raw
Spleen_Osipenko_x1_L9_neg_09.raw
Urine_Osipenko_x1_L19_neg_19.raw
1e7
1e6
0
2
4
0.0
0.5
1.0
1
250000
500000
200000
730
735
740
745
750
755
760
765

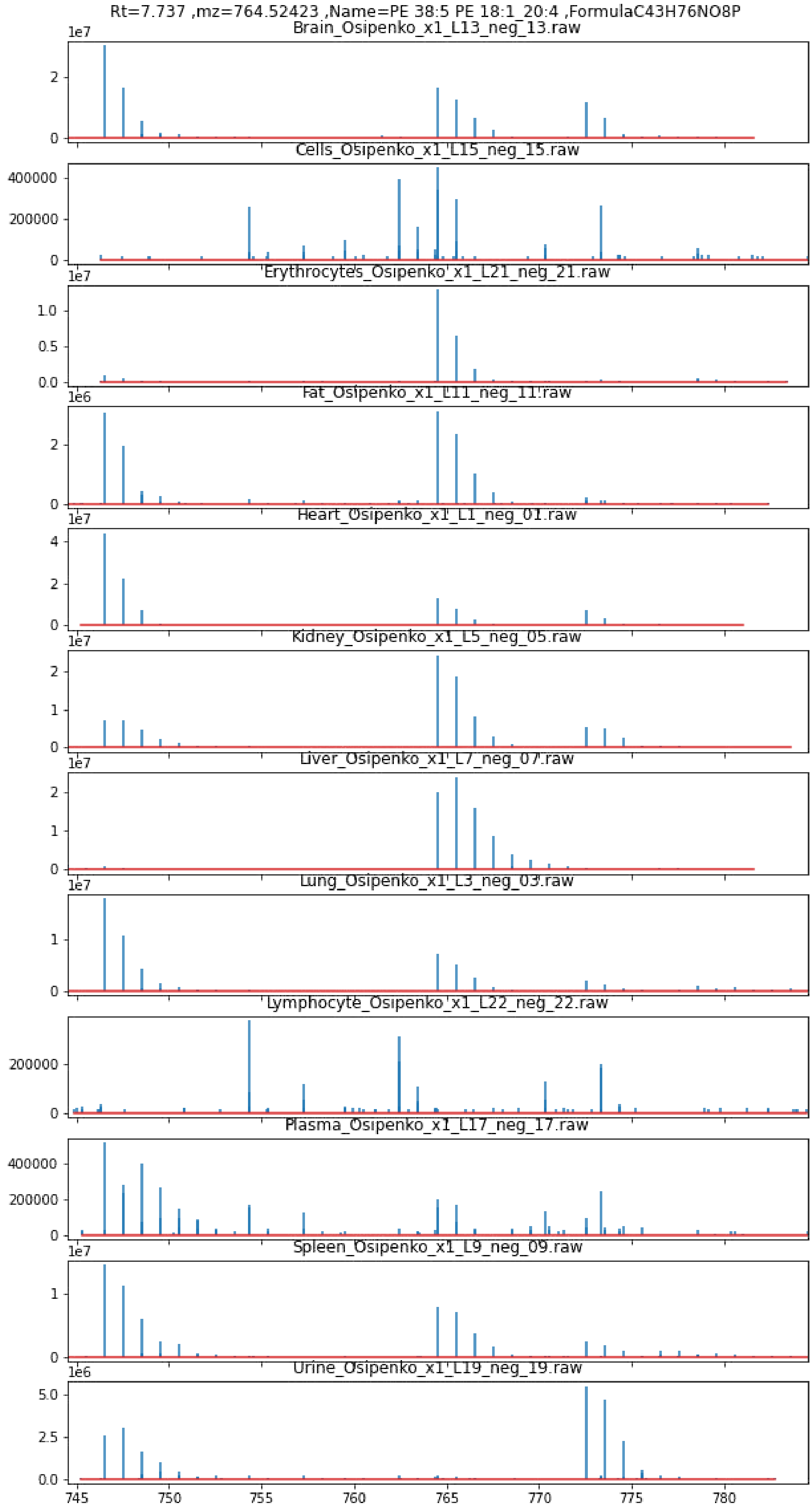

Rt=7.737 ,mz=764.52423 ,Name=PE 38:5 PE 18:1_20:4 ,FormulaC43H76NO8P
Brain_Osipenko_x1_L13_neg_13.raw
Cells_Osipenko_x1_L15_neg_15.raw
Erythrocytes_Osipenko_x1_L21_neg_21.raw
Fat_Osipenko_x1_L11_neg_11.raw
Heart_Osipenko_x1_L1_neg_01.raw
Kidney_Osipenko_x1_L5_neg_05.raw
Liver_Osipenko_x1_L7_neg_07.raw
Lung_Osipenko_x1_L3_neg_03.raw
Lymphocyte_Osipenko_x1_L22_neg_22.raw
Plasma_Osipenko_x1_L17_neg_17.raw
Spleen_Osipenko_x1_L9_neg_09.raw
Urine_Osipenko_x1_L19_neg_19.raw
745 750 755 760 765 770 775 780

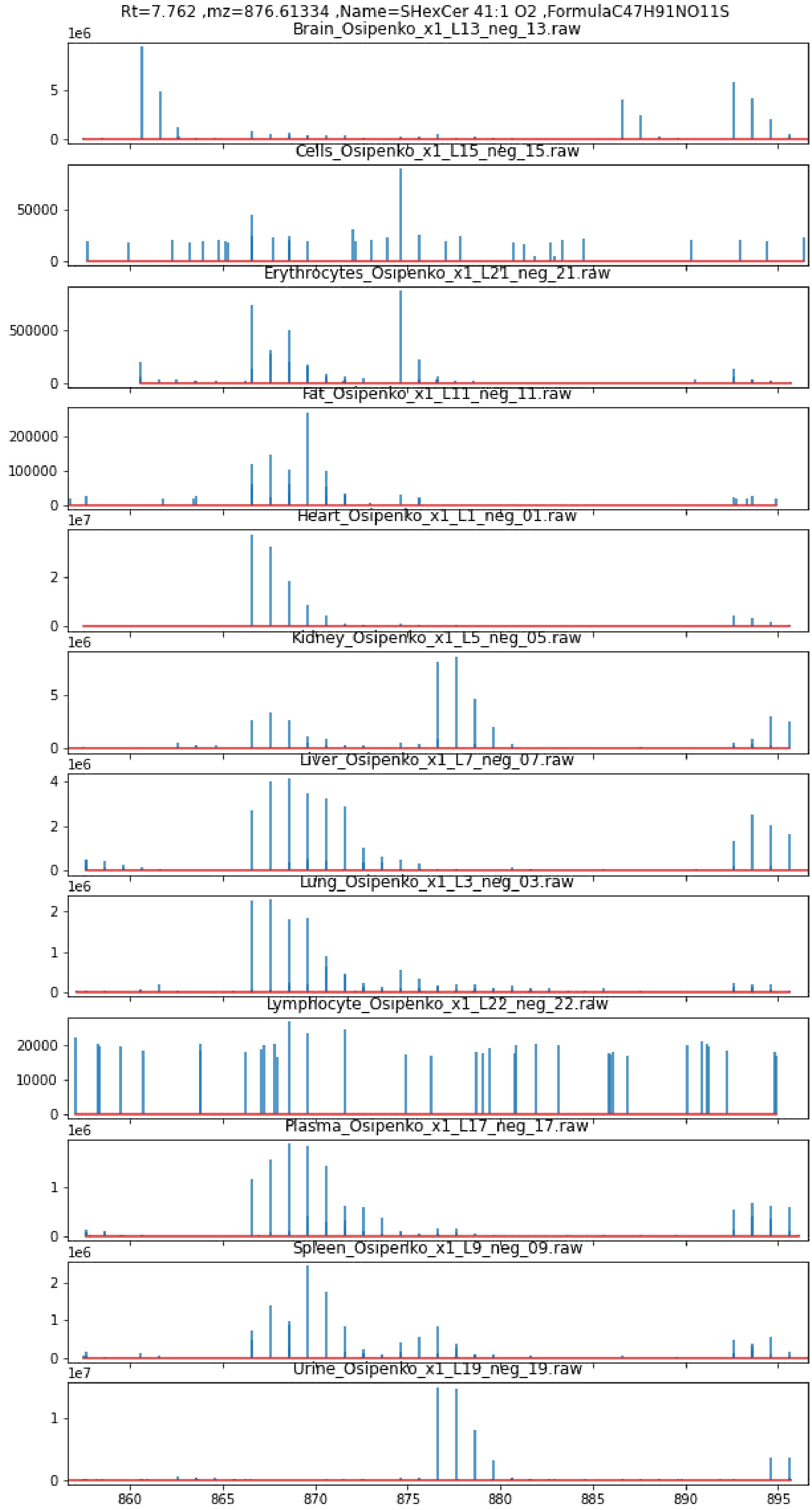
Rt=7.762 ,mz=876.61334 ,Name=SHexCer 41:1 O2 ,FormulaC47H91NO11S
Brain_Osipenko_x1_L13_neg_13.raw
Cells_Osipenko_x1_L15_neg_15.raw
Erythrocytes_Osipenko_x1_L21_neg_21.raw
Fat_Osipenko_x1_L11_neg_11.raw
Heart_Osipenko_x1_L1_neg_01.raw
Kidney_Osipenko_x1_L5_neg_05.raw
Liver_Osipenko_x1_L7_neg_07.raw
Lung_Osipenko_x1_L3_neg_03.raw
Lymphocyte_Osipenko_x1_L22_neg_22.raw
Plasma_Osipenko_x1_L17_neg_17.raw
Spleen_Osipenko_x1_L9_neg_09.raw
Urine_Osipenko_x1_L19_neg_19.raw

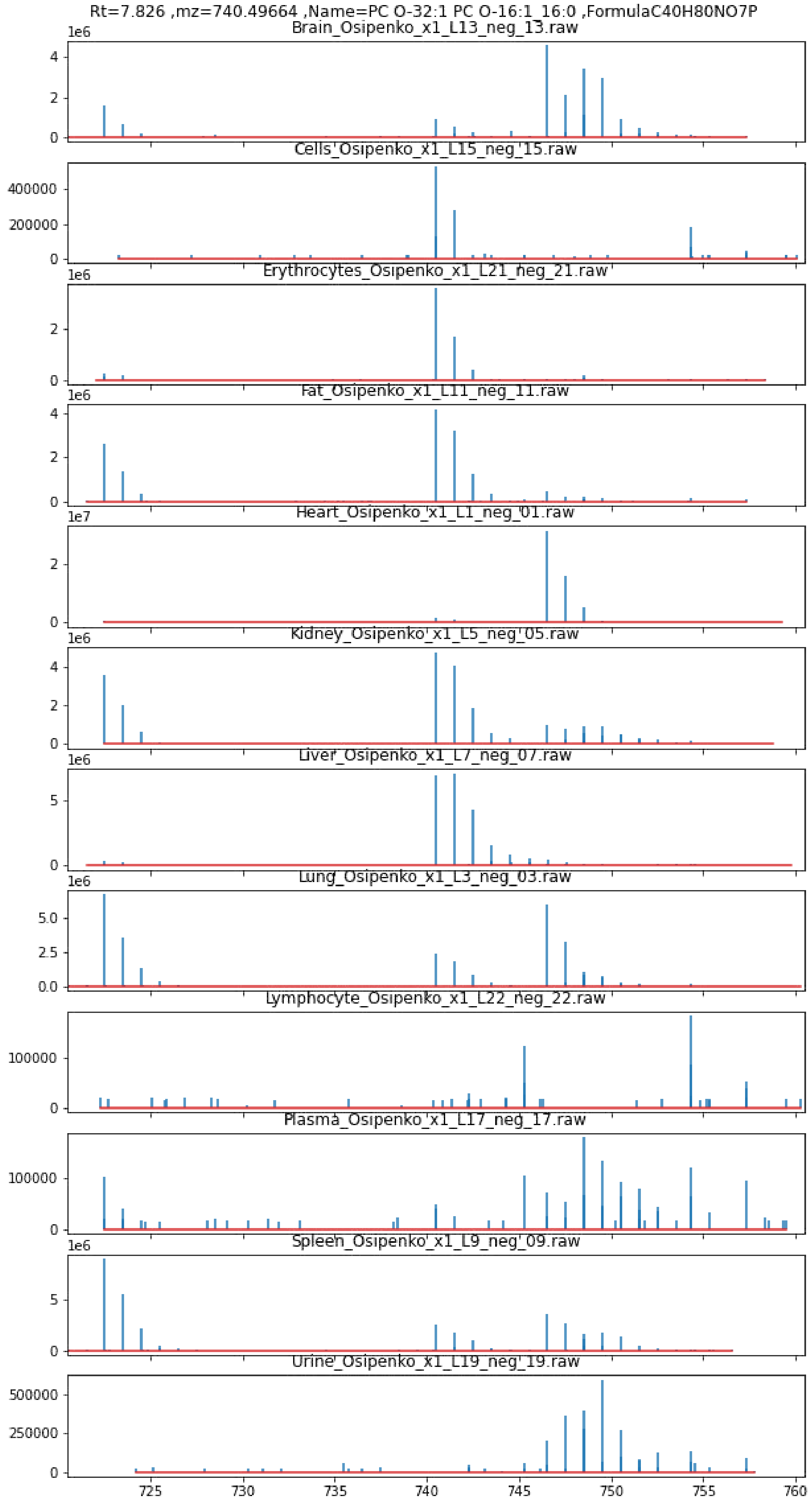

Rt=7.826 ,mz=740.49664 ,Name=PC O-32:1 PC O-16:1_16:0 ,FormulaC40H80NO7P
Brain_Osipenko_x1_L13_neg_13.raw
Cells_Osipenko_x1_L15_neg_15.raw
Erythrocytes_Osipenko_x1_L21_neg_21.raw
Fat_Osipenko_x1_L11_neg_11.raw
Heart_Osipenko_x1_L1_neg_01.raw
Kidney_Osipenko_x1_L5_neg_05.raw
Liver_Osipenko_x1_L7_neg_07.raw
Lung_Osipenko_x1_L3_neg_03.raw
Lymphocyte_Osipenko_x1_L22_neg_22.raw
Plasma_Osipenko_x1_L17_neg_17.raw
Spleen_Osipenko_x1_L9_neg_09.raw
Urine_Osipenko_x1_L19_neg_19.raw
725
730
735
740
745
750
755
760

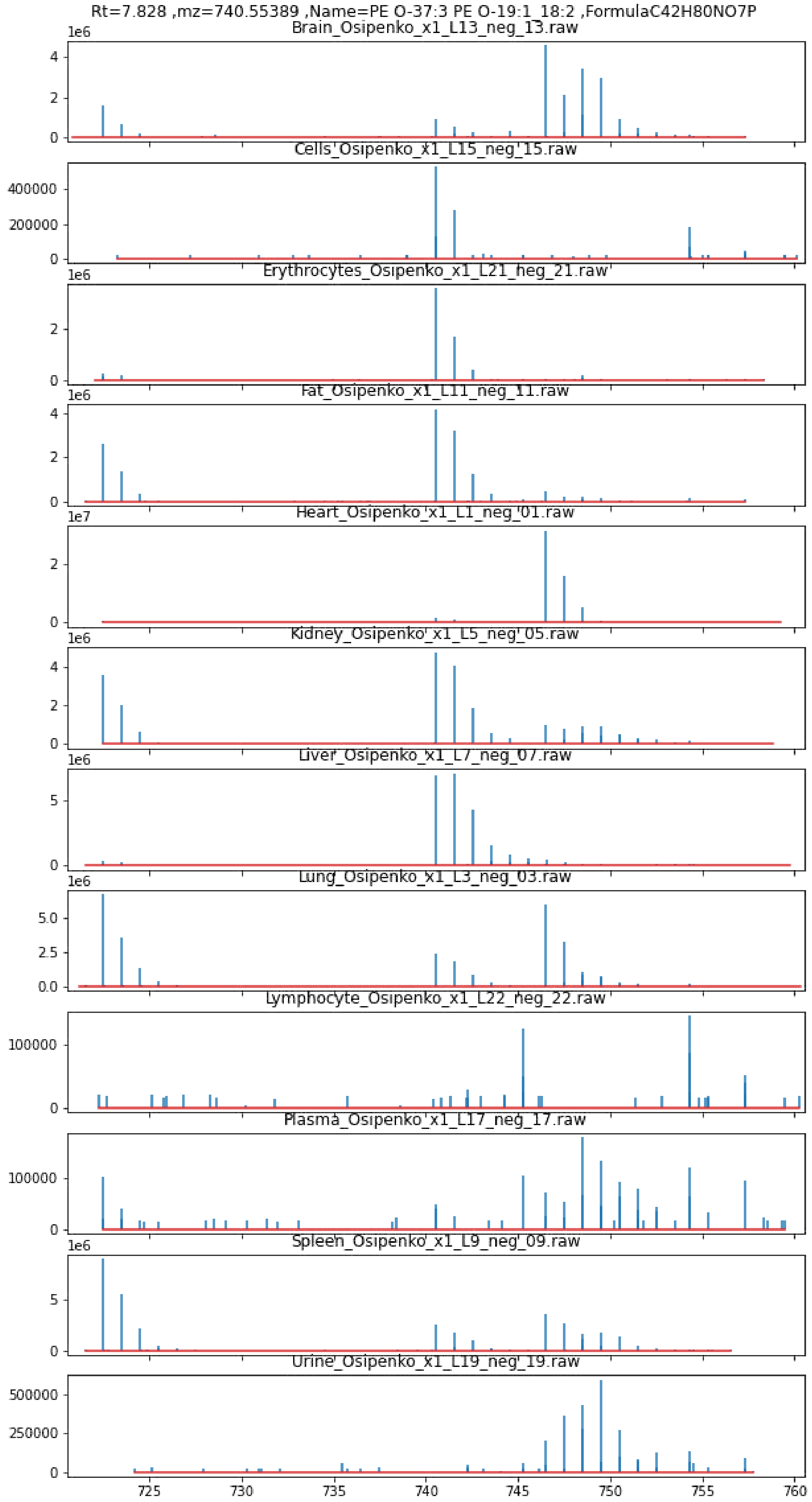
Rt=7.828 ,mz=740.55389 ,Name=PE O-37:3 PE O-19:1_18:2 ,FormulaC42H80NO7P
Brain_Osipenko_x1_L13_neg_13.raw
Cells_Osipenko_x1_L15_neg_15.raw
Erythrocytes_Osipenko_x1_L21_neg_21.raw
Fat_Osipenko_x1_L11_neg_11.raw
Heart_Osipenko_x1_L1_neg_01.raw
Kidney_Osipenko_x1_L5_neg_05.raw
Liver_Osipenko_x1_L7_neg_07.raw
Lung_Osipenko_x1_L3_neg_03.raw
Lymphocyte_Osipenko_x1_L22_neg_22.raw
Plasma_Osipenko_x1_L17_neg_17.raw
Spleen_Osipenko_x1_L9_neg_09.raw
Urine_Osipenko_x1_L19_neg_19.raw

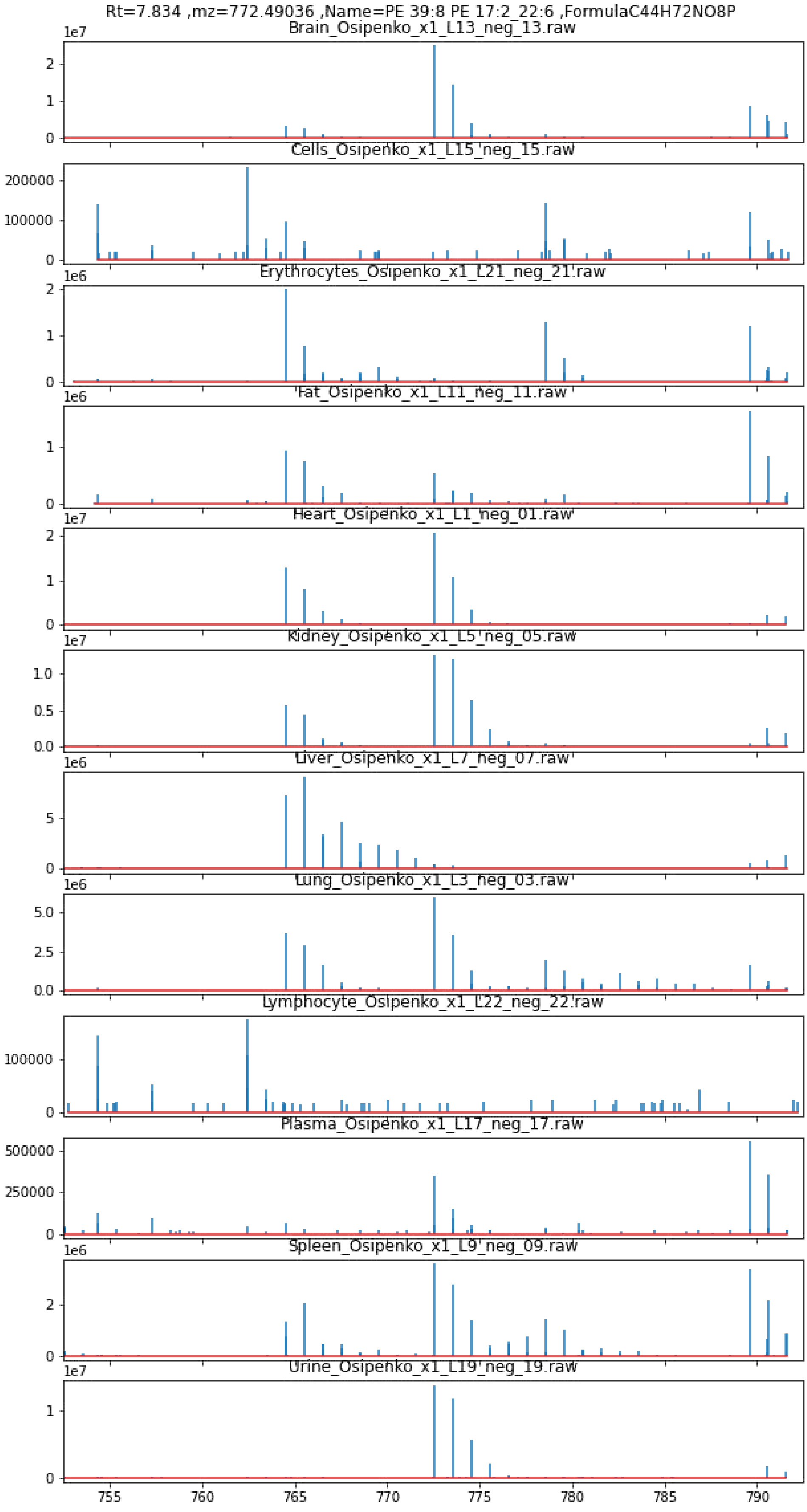
Rt=7.834 ,mz=772.49036 ,Name=PE 39:8 PE 17:2_22:6 ,FormulaC44H72NO8P
Brain_Osipenko_x1_L13_neg_13.raw
Cells_Osipenko_x1_L15_neg_15.raw
Erythrocytes_Osipenko_x1_L21_neg_21.raw
Fat_Osipenko_x1_L11_neg_11.raw
Heart_Osipenko_x1_L1_neg_01.raw
Kidney_Osipenko_x1_L5_neg_05.raw
Liver_Osipenko_x1_L7_neg_07.raw
Lung_Osipenko_x1_L3_neg_03.raw
Lymphocyte_Osipenko_x1_L22_neg_22.raw
Plasma_Osipenko_x1_L17_neg_17.raw
Spleen_Osipenko_x1_L9_neg_09.raw
Urine_Osipenko_x1_L19_neg_19.raw
755
760
765
770
775
780
785
790

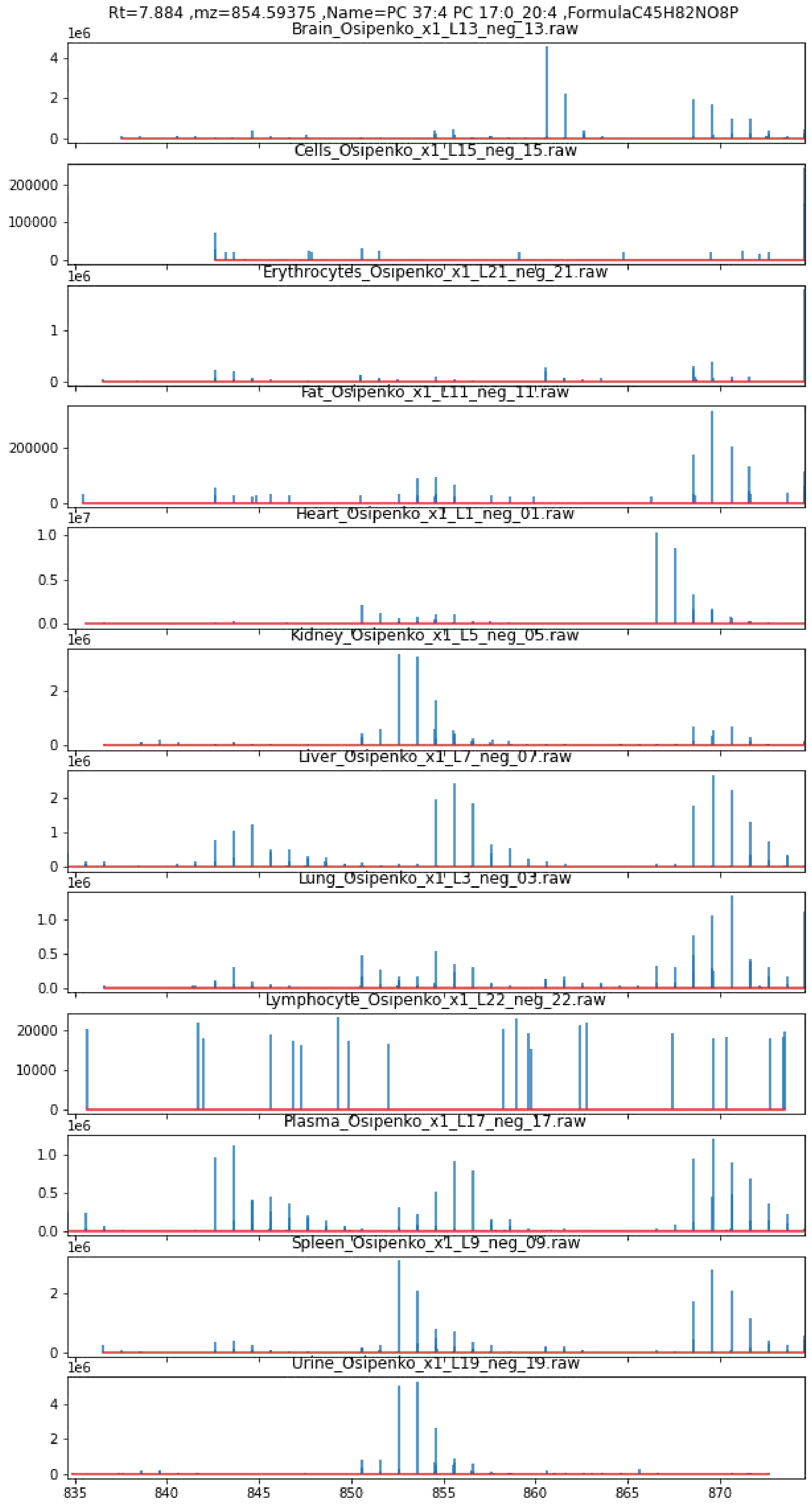

Rt=7.884 ,mz=854.59375 ,Name=PC 37:4 PC 17:0_20:4 ,FormulaC45H82NO8P
Brain_Osipenko_x1_L13_neg_13.raw
Cells_Osipenko_x1_L15_neg_15.raw
Erythrocytes_Osipenko_x1_L21_neg_21.raw
Fat_Osipenko_x1_L11_neg_11.raw
Heart_Osipenko_x1_L1_neg_01.raw
Kidney_Osipenko_x1_L5_neg_05.raw
Liver_Osipenko_x1_L7_neg_07.raw
Lung_Osipenko_x1_L3_neg_03.raw
Lymphocyte_Osipenko_x1_L22_neg_22.raw
Plasma_Osipenko_x1_L17_neg_17.raw
Spleen_Osipenko_x1_L9_neg_09.raw
Urine_Osipenko_x1_L19_neg_19.raw
835
840
845
850
855
860
865
870

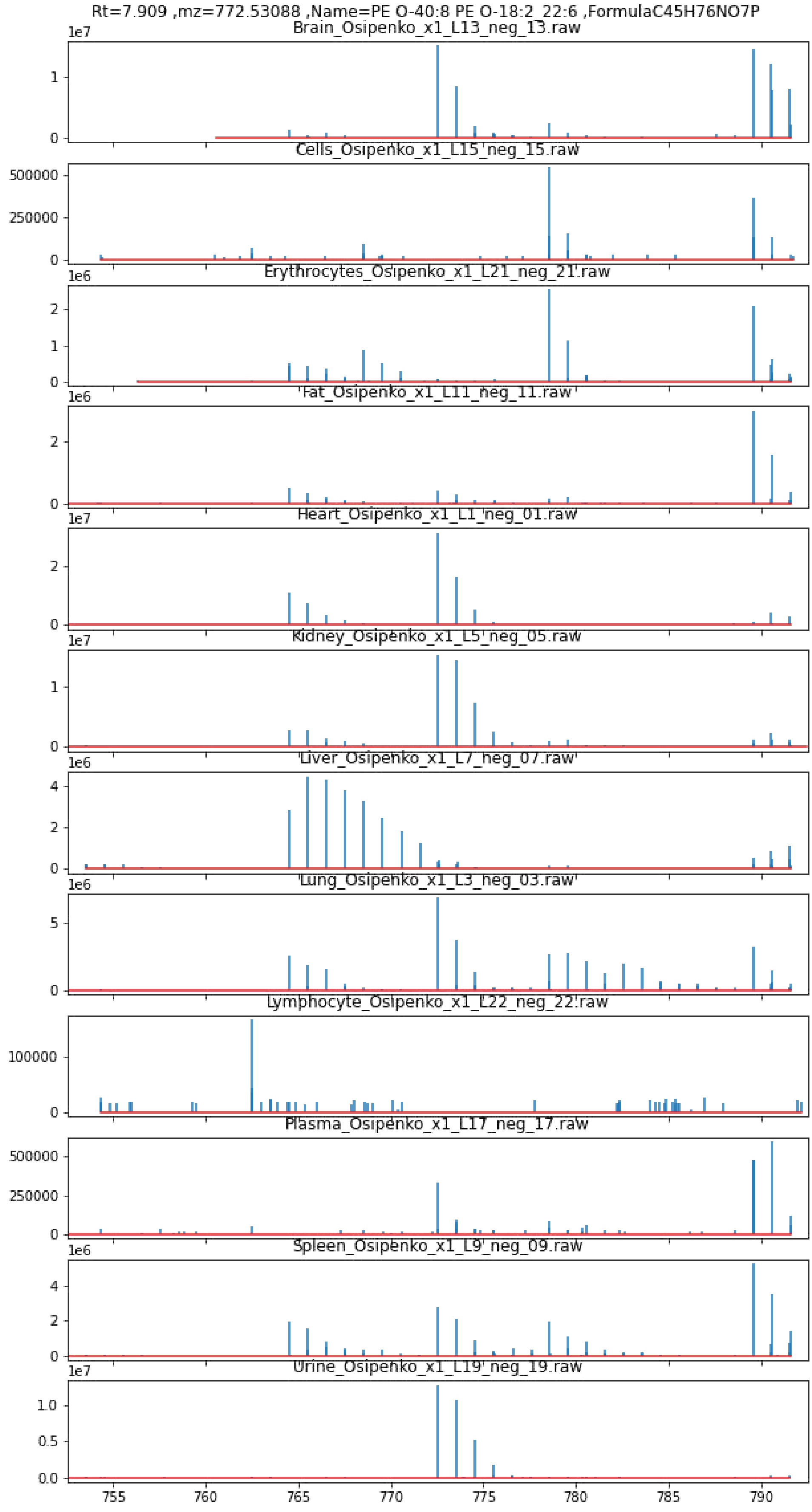

Rt=7.909 ,mz=772.53088 ,Name=PE O-40:8 PE O-18:2_22:6 ,FormulaC45H76NO7P
Brain_Osipenko_x1_L13_neg_13.raw
Cells_Osipenko_x1_L15_neg_15.raw
Erythrocytes_Osipenko_x1_L21_neg_21.raw
Fat_Osipenko_x1_L11_neg_11.raw
Heart_Osipenko_x1_L1_neg_01.raw
Kidney_Osipenko_x1_L5_neg_05.raw
Liver_Osipenko_x1_L7_neg_07.raw
Lung_Osipenko_x1_L3_neg_03.raw
Lymphocyte_Osipenko_x1_L22_neg_22.raw
Plasma_Osipenko_x1_L17_neg_17.raw
Spleen_Osipenko_x1_L9_neg_09.raw
Urine_Osipenko_x1_L19_neg_19.raw
755
760
765
770
775
780
785
790

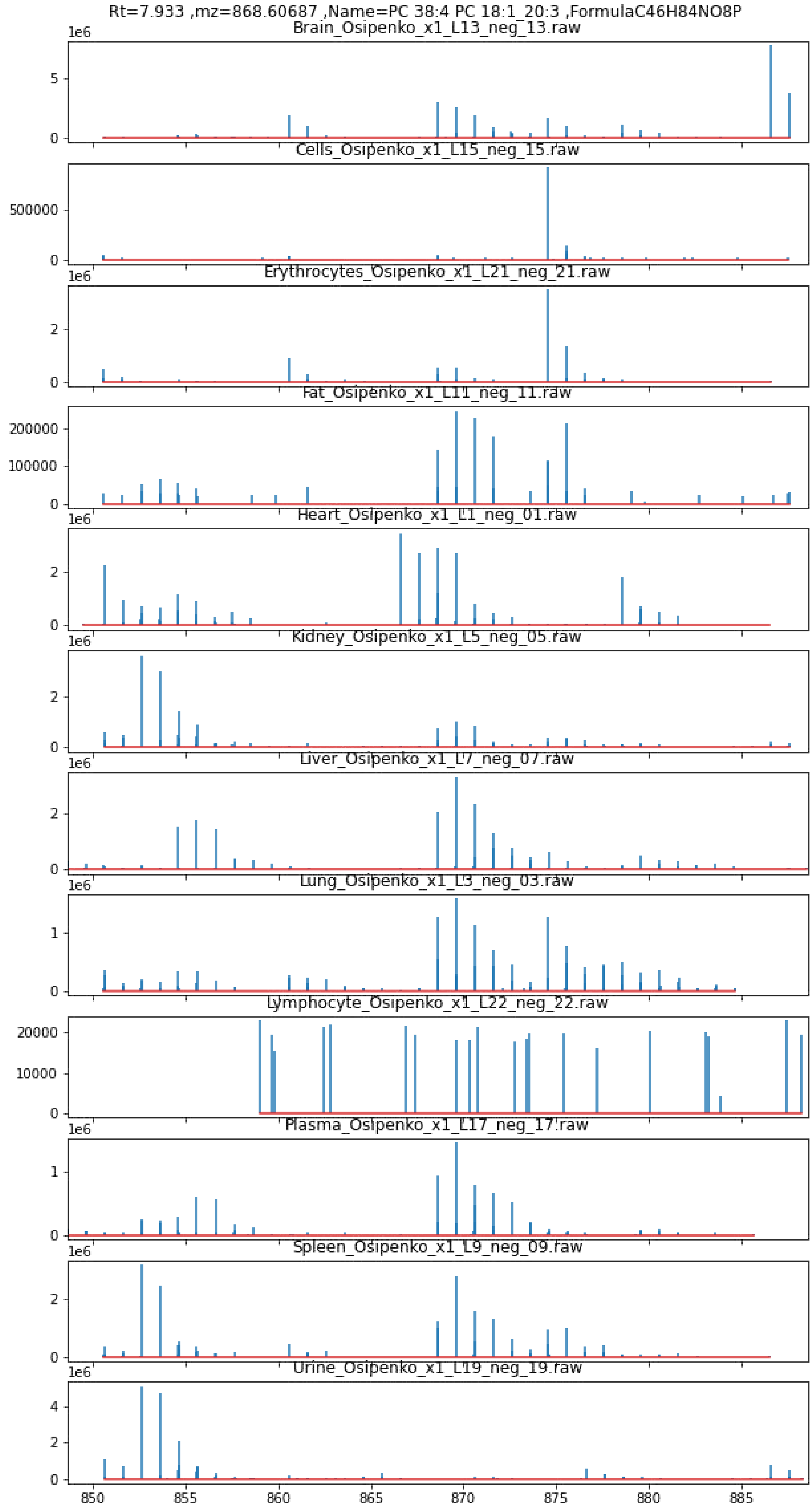

Rt=7.933 ,mz=868.60687 ,Name=PC 38:4 PC 18:1_20:3 ,FormulaC46H84NO8P
Brain_Osipenko_x1_L13_neg_13.raw
Cells_Osipenko_x1_L15_neg_15.raw
Erythrocytes_Osipenko_x1_L21_neg_21.raw
Fat_Osipenko_x1_L11_neg_11.raw
Heart_Osipenko_x1_L1_neg_01.raw
Kidney_Osipenko_x1_L5_neg_05.raw
Liver_Osipenko_x1_L7_neg_07.raw
Lung_Osipenko_x1_L3_neg_03.raw
Lymphocyte_Osipenko_x1_L22_neg_22.raw
Plasma_Osipenko_x1_L17_neg_17.raw
Spleen_Osipenko_x1_L9_neg_09.raw
Urine_Osipenko_x1_L19_neg_19.raw
1e6
850
855
860
865
870
875
880
885

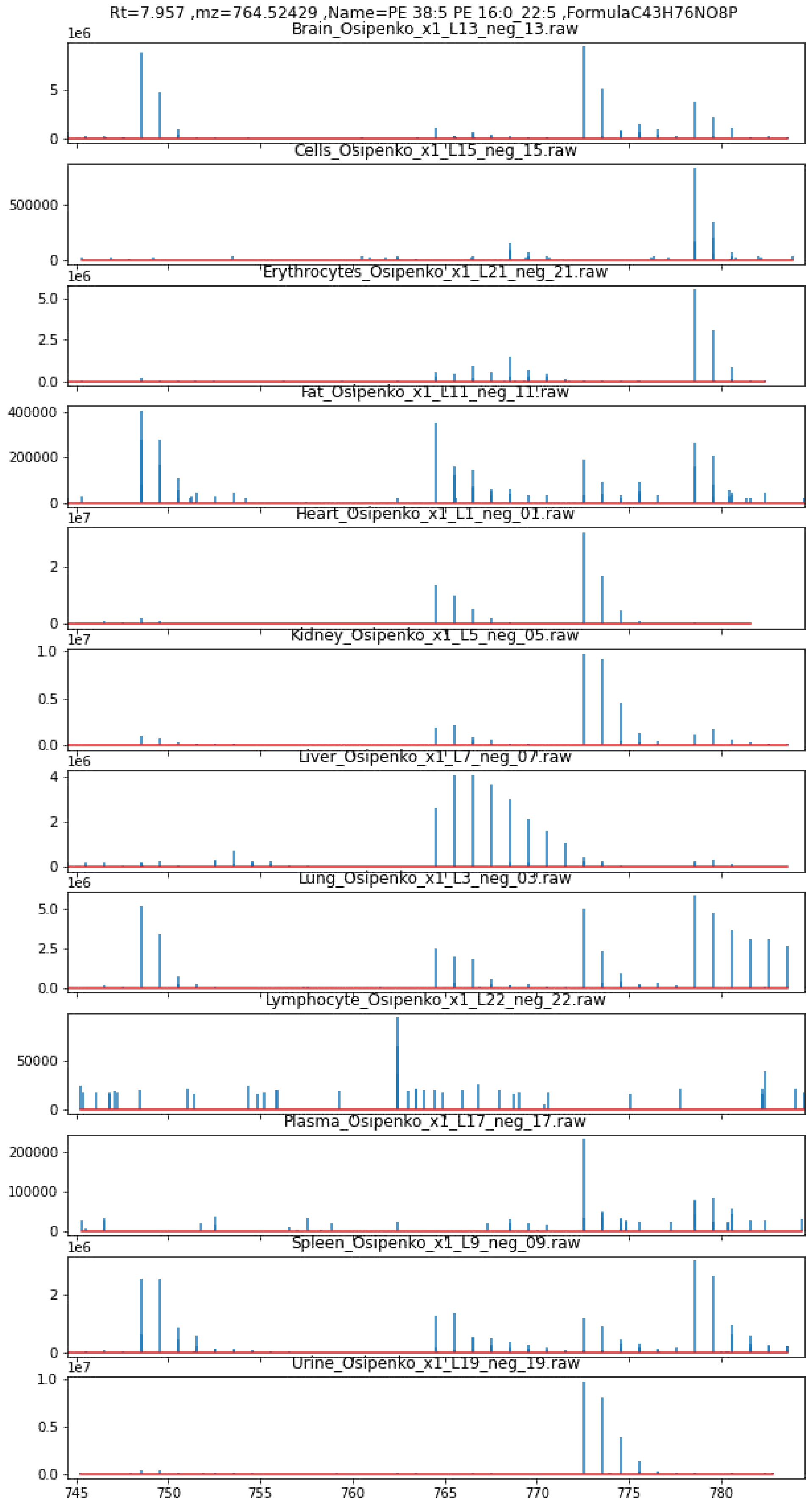
Rt=7.957 ,mz=764.52429 ,Name=PE 38:5 PE 16:0_22:5 ,FormulaC43H76NO8P
Brain_Osipenko_x1_L13_neg_13.raw
Cells_Osipenko_x1_L15_neg_15.raw
Erythrocytes_Osipenko_x1_L21_neg_21.raw
Fat_Osipenko_x1_L11_neg_11.raw
Heart_Osipenko_x1_L1_neg_01.raw
Kidney_Osipenko_x1_L5_neg_05.raw
Liver_Osipenko_x1_L7_neg_07.raw
Lung_Osipenko_x1_L3_neg_03.raw
Lymphocyte_Osipenko_x1_L22_neg_22.raw
Plasma_Osipenko_x1_L17_neg_17.raw
Spleen_Osipenko_x1_L9_neg_09.raw
Urine_Osipenko_x1_L19_neg_19.raw
745
750
755
760
765
770
775
780

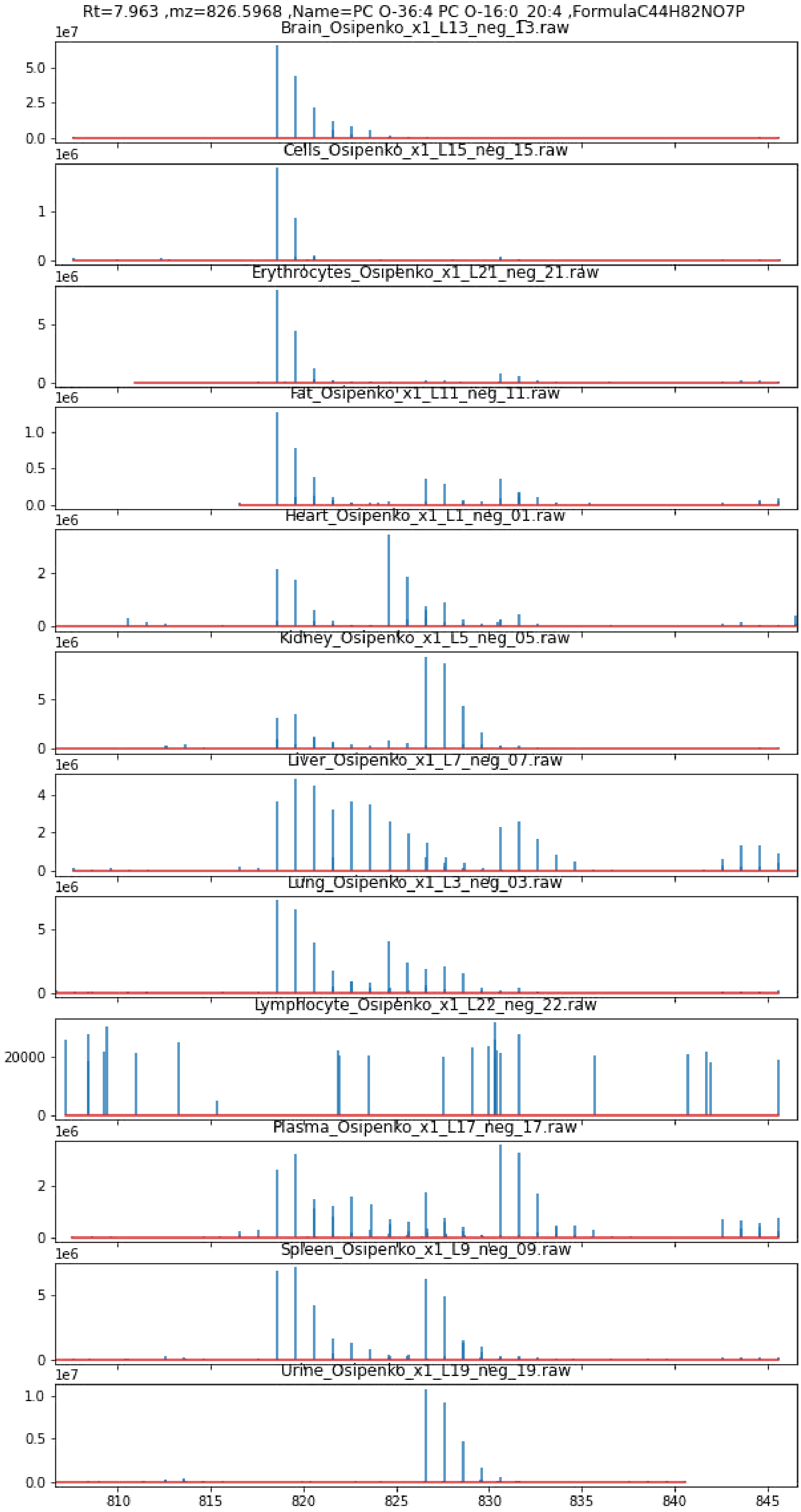

Rt=7.963 ,mz=826.5968 ,Name=PC O-36:4 PC O-16:0_20:4 ,FormulaC44H82NO7P
Brain_Osipenko_x1_L13_neg_13.raw
Cells_Osipenko_x1_L15_neg_15.raw
Erythrocytes_Osipenko_x1_L21_neg_21.raw
Fat_Osipenko_x1_L11_neg_11.raw
Heart_Osipenko_x1_L1_neg_01.raw
Kidney_Osipenko_x1_L5_neg_05.raw
Liver_Osipenko_x1_L7_neg_07.raw
Lung_Osipenko_x1_L3_neg_03.raw
Lymphocyte_Osipenko_x1_L22_neg_22.raw
Plasma_Osipenko_x1_L17_neg_17.raw
Spleen_Osipenko_x1_L9_neg_09.raw
Urine_Osipenko_x1_L19_neg_19.raw
810
815
820
825
830
835
840
845

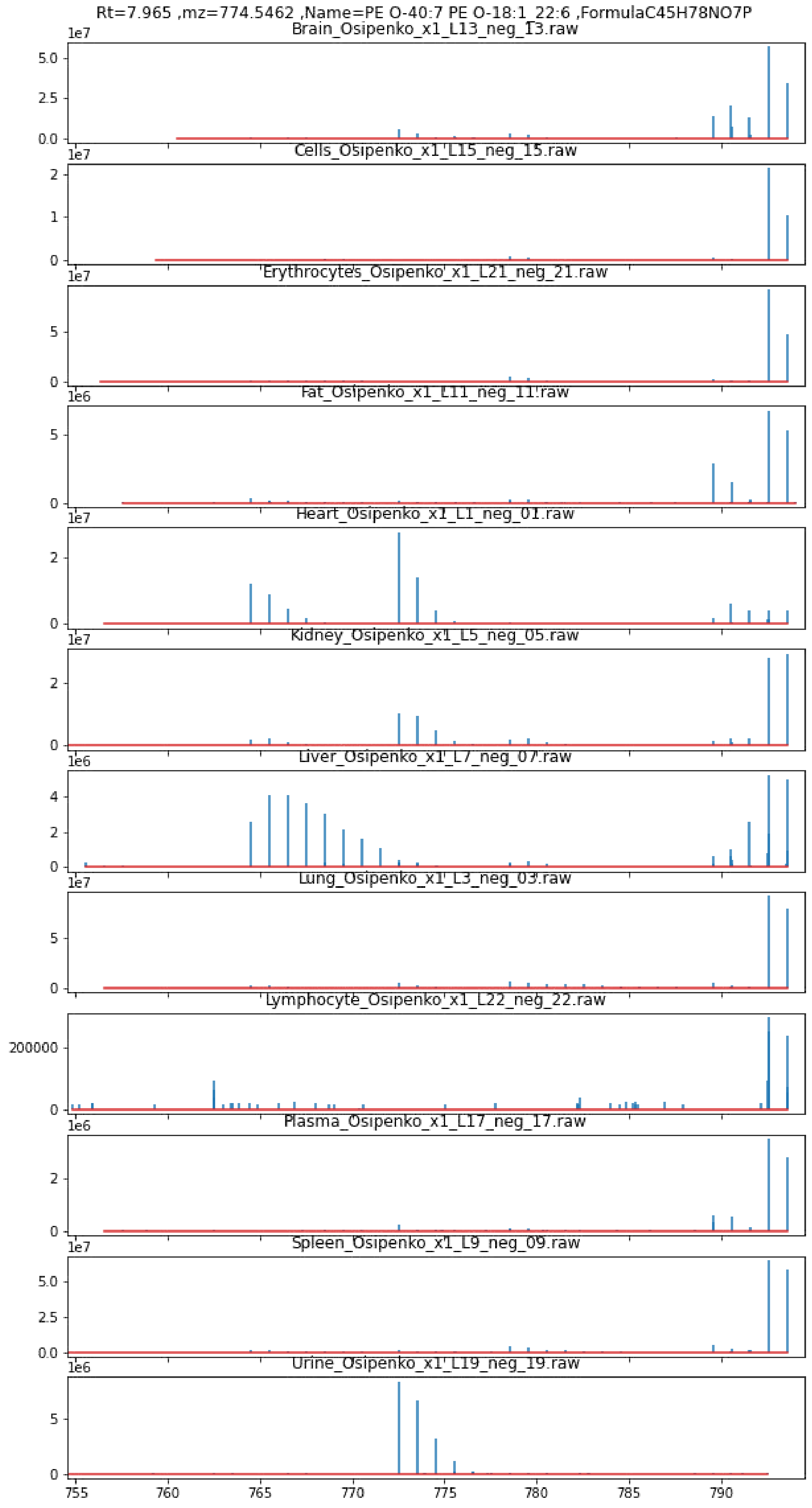

Rt=7.965 ,mz=774.5462 ,Name=PE O-40:7 PE O-18:1_22:6 ,FormulaC45H78NO7P
Brain_Osipenko_x1_L13_neg_13.raw
Cells_Osipenko_x1_L15_neg_15.raw
Erythrocytes_Osipenko_x1_L21_neg_21.raw
Fat_Osipenko_x1_L11_neg_11.raw
Heart_Osipenko_x1_L1_neg_01.raw
Kidney_Osipenko_x1_L5_neg_05.raw
Liver_Osipenko_x1_L7_neg_07.raw
Lung_Osipenko_x1_L3_neg_03.raw
Lymphocyte_Osipenko_x1_L22_neg_22.raw
Plasma_Osipenko_x1_L17_neg_17.raw
Spleen_Osipenko_x1_L9_neg_09.raw
Urine_Osipenko_x1_L19_neg_19.raw
1e7
1e6
200000
755
760
765
770
775
780
785
790

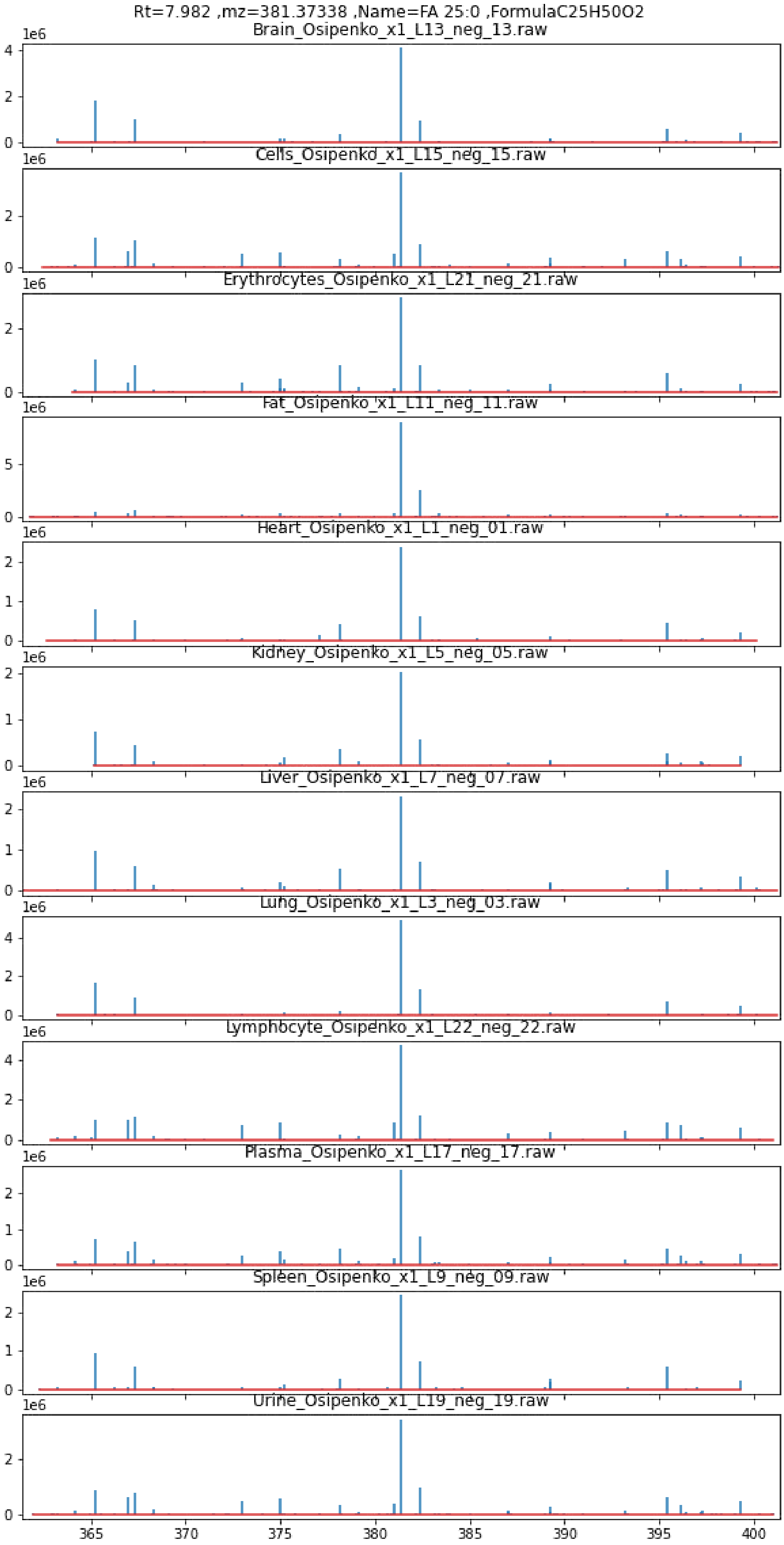

Rt=7.982 ,mz=381.37338 ,Name=FA 25:0 ,FormulaC25H50O2
Brain_Osipenko_x1_L13_neg_13.raw
Cells_Osipenko_x1_L15_neg_15.raw
Erythrocytes_Osipenko_x1_L21_neg_21.raw
Fat_Osipenko_x1_L11_neg_11.raw
Heart_Osipenko_x1_L1_neg_01.raw
Kidney_Osipenko_x1_L5_neg_05.raw
Liver_Osipenko_x1_L7_neg_07.raw
Lung_Osipenko_x1_L3_neg_03.raw
Lymphocyte_Osipenko_x1_L22_neg_22.raw
Plasma_Osipenko_x1_L17_neg_17.raw
Spleen_Osipenko_x1_L9_neg_09.raw
Urine_Osipenko_x1_L19_neg_19.raw
1e6
365
370
375
380
385
390
395
400

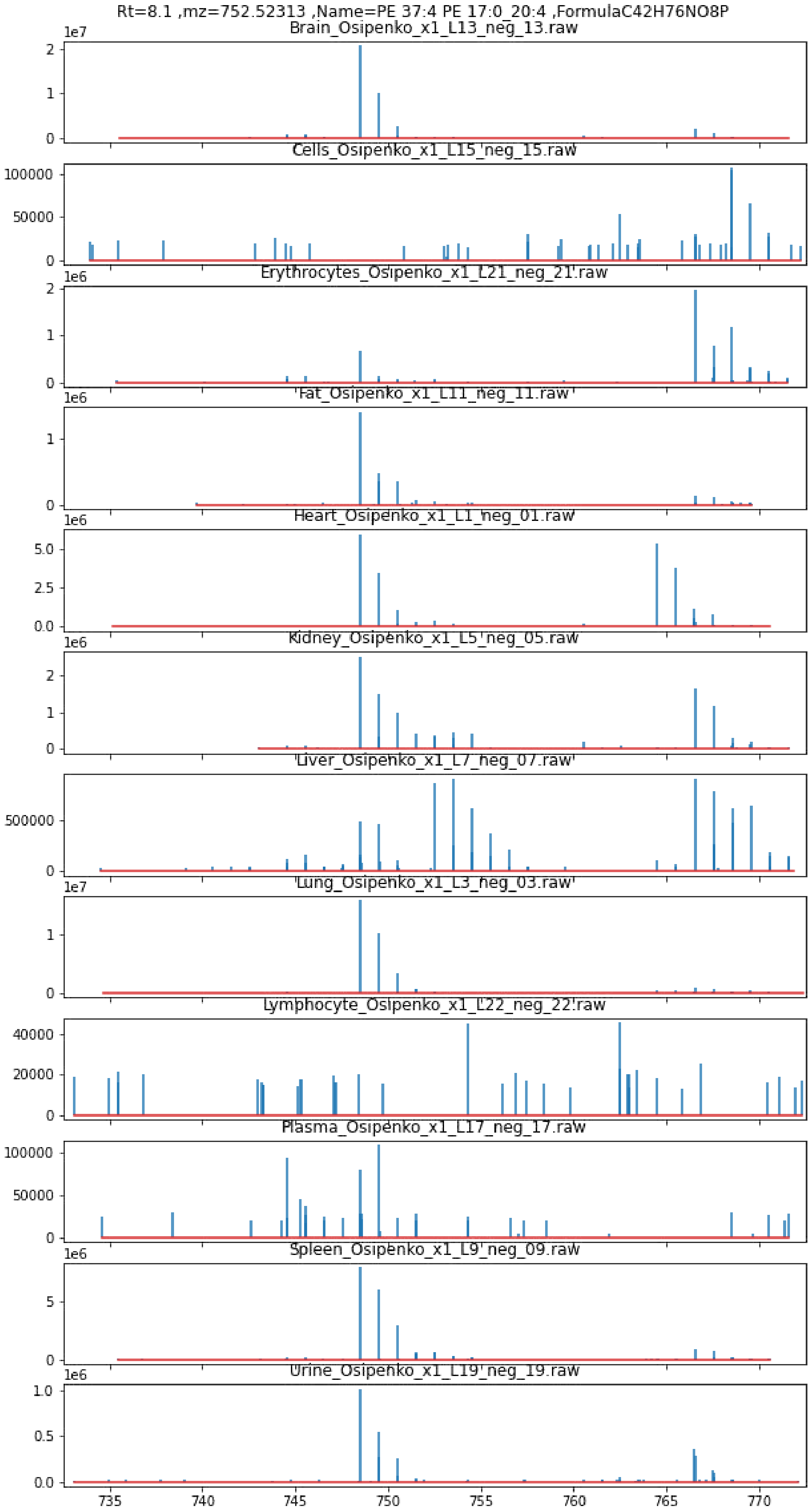

Rt=8.1 ,mz=752.52313 ,Name=PE 37:4 PE 17:0_20:4 ,FormulaC42H76NO8P
Brain_Osipenko_x1_L13_neg_13.raw
Cells_Osipenko_x1_L15_neg_15.raw
Erythrocytes_Osipenko_x1_L21_neg_21.raw
Fat_Osipenko_x1_L11_neg_11.raw
Heart_Osipenko_x1_L1_neg_01.raw
Kidney_Osipenko_x1_L5_neg_05.raw
Liver_Osipenko_x1_L7_neg_07.raw
Lung_Osipenko_x1_L3_neg_03.raw
Lymphocyte_Osipenko_x1_L22_neg_22.raw
Plasma_Osipenko_x1_L17_neg_17.raw
Spleen_Osipenko_x1_L9_neg_09.raw
Urine_Osipenko_x1_L19_neg_19.raw

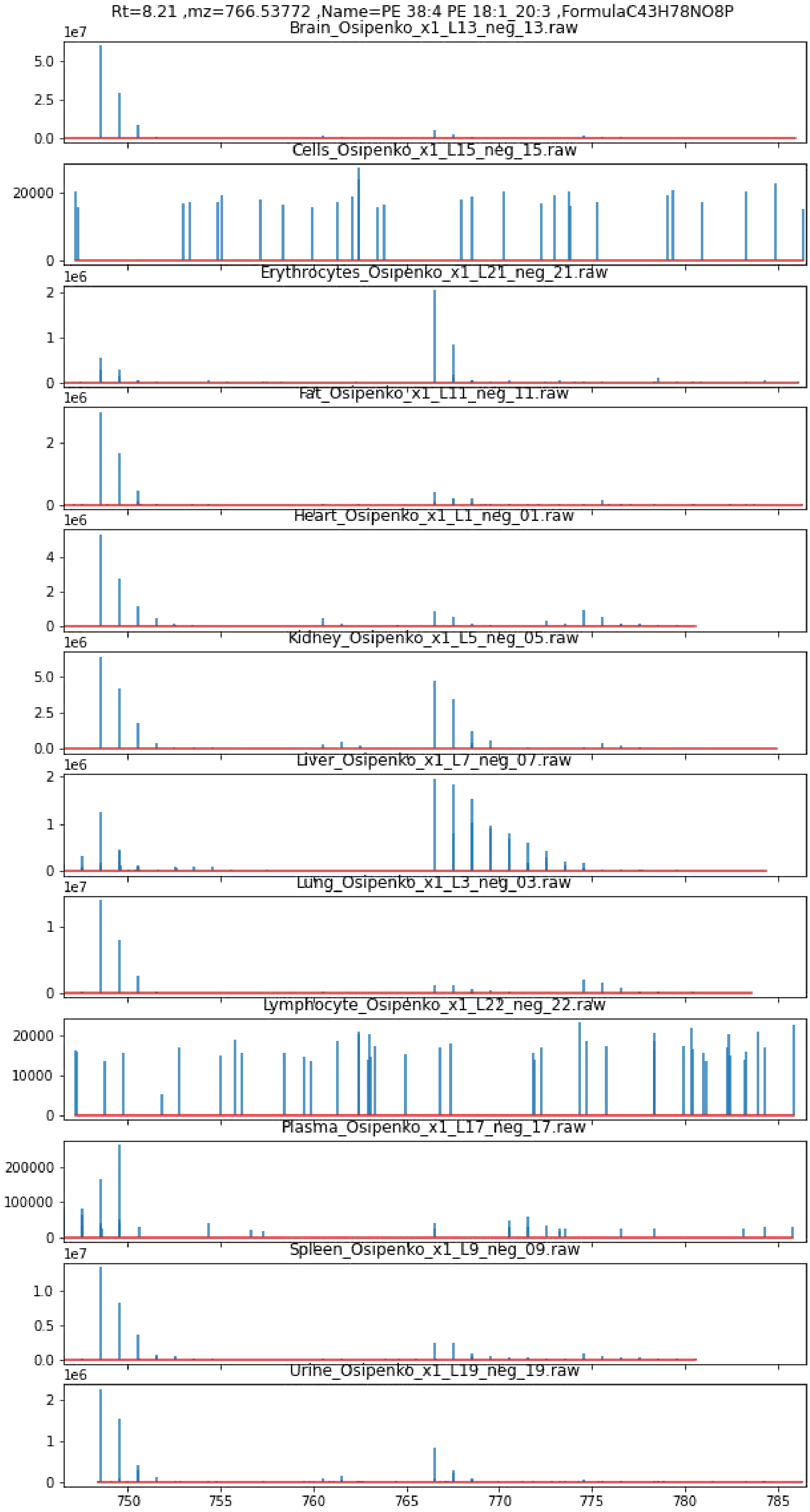

Rt=8.21 ,mz=766.53772 ,Name=PE 38:4 PE 18:1_20:3 ,FormulaC43H78NO8P
Brain_Osipenko_x1_L13_neg_13.raw
Cells_Osipenko_x1_L15_neg_15.raw
Erythrocytes_Osipenko_x1_L21_neg_21.raw
Fat_Osipenko_x1_L11_neg_11.raw
Heart_Osipenko_x1_L1_neg_01.raw
Kidney_Osipenko_x1_L5_neg_05.raw
Liver_Osipenko_x1_L7_neg_07.raw
Lung_Osipenko_x1_L3_neg_03.raw
Lymphocyte_Osipenko_x1_L22_neg_22.raw
Plasma_Osipenko_x1_L17_neg_17.raw
Spleen_Osipenko_x1_L9_neg_09.raw
Urine_Osipenko_x1_L19_neg_19.raw

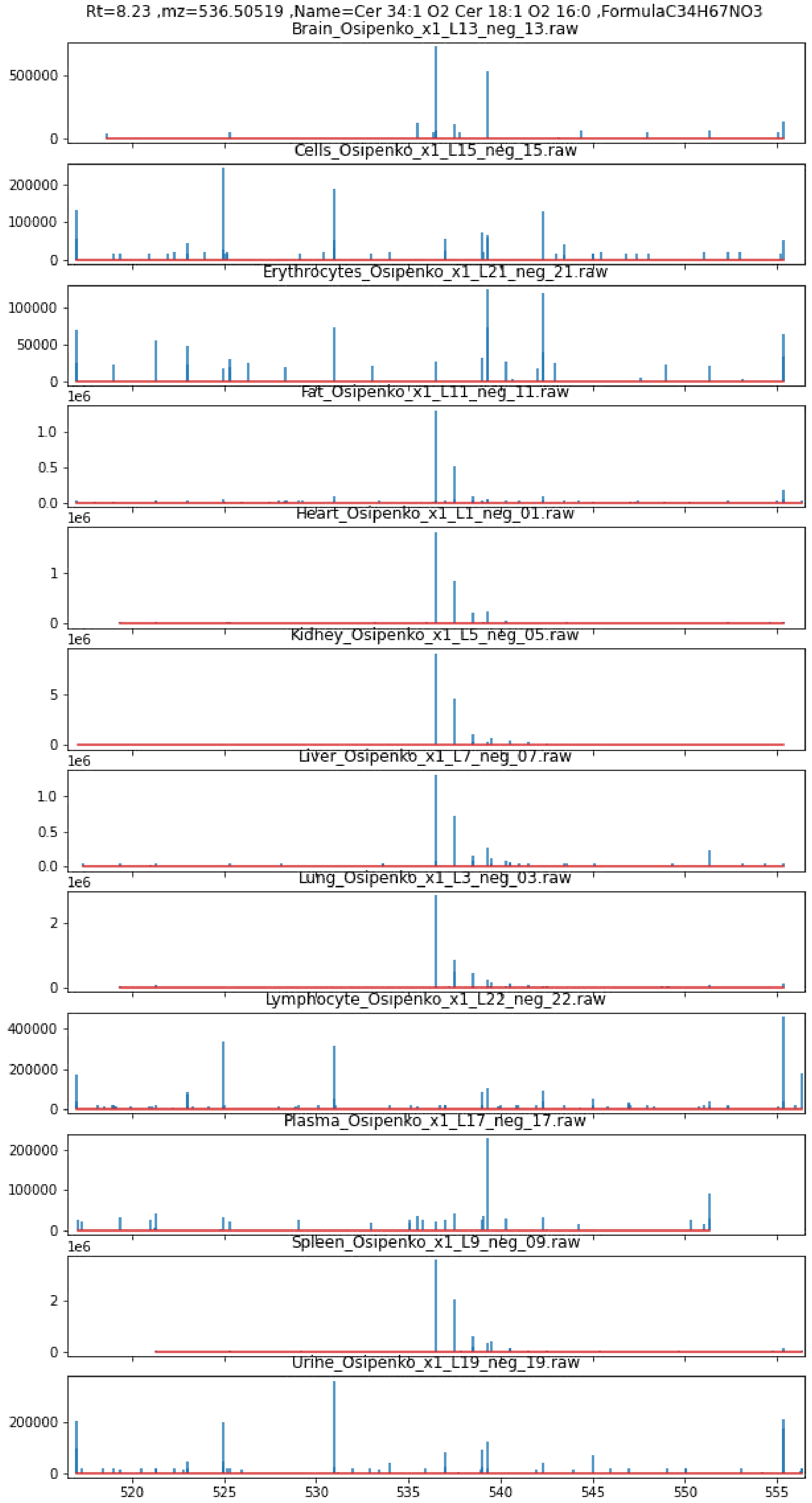
Rt=8.23 ,mz=536.50519 ,Name=Cer 34:1 O2 Cer 18:1 O2 16:0 ,FormulaC34H67NO3
Brain_Osipenko_x1_L13_neg_13.raw
Cells_Osipenko_x1_L15_neg_15.raw
Erythrocytes_Osipenko_x1_L21_neg_21.raw
Fat_Osipenko_x1_L11_neg_11.raw
Heart_Osipenko_x1_L1_neg_01.raw
Kidney_Osipenko_x1_L5_neg_05.raw
Liver_Osipenko_x1_L7_neg_07.raw
Lung_Osipenko_x1_L3_neg_03.raw
Lymphocyte_Osipenko_x1_L22_neg_22.raw
Plasma_Osipenko_x1_L17_neg_17.raw
Spleen_Osipenko_x1_L9_neg_09.raw
Urine_Osipenko_x1_L19_neg_19.raw
1e6
520
525
530
535
540
545
550
555

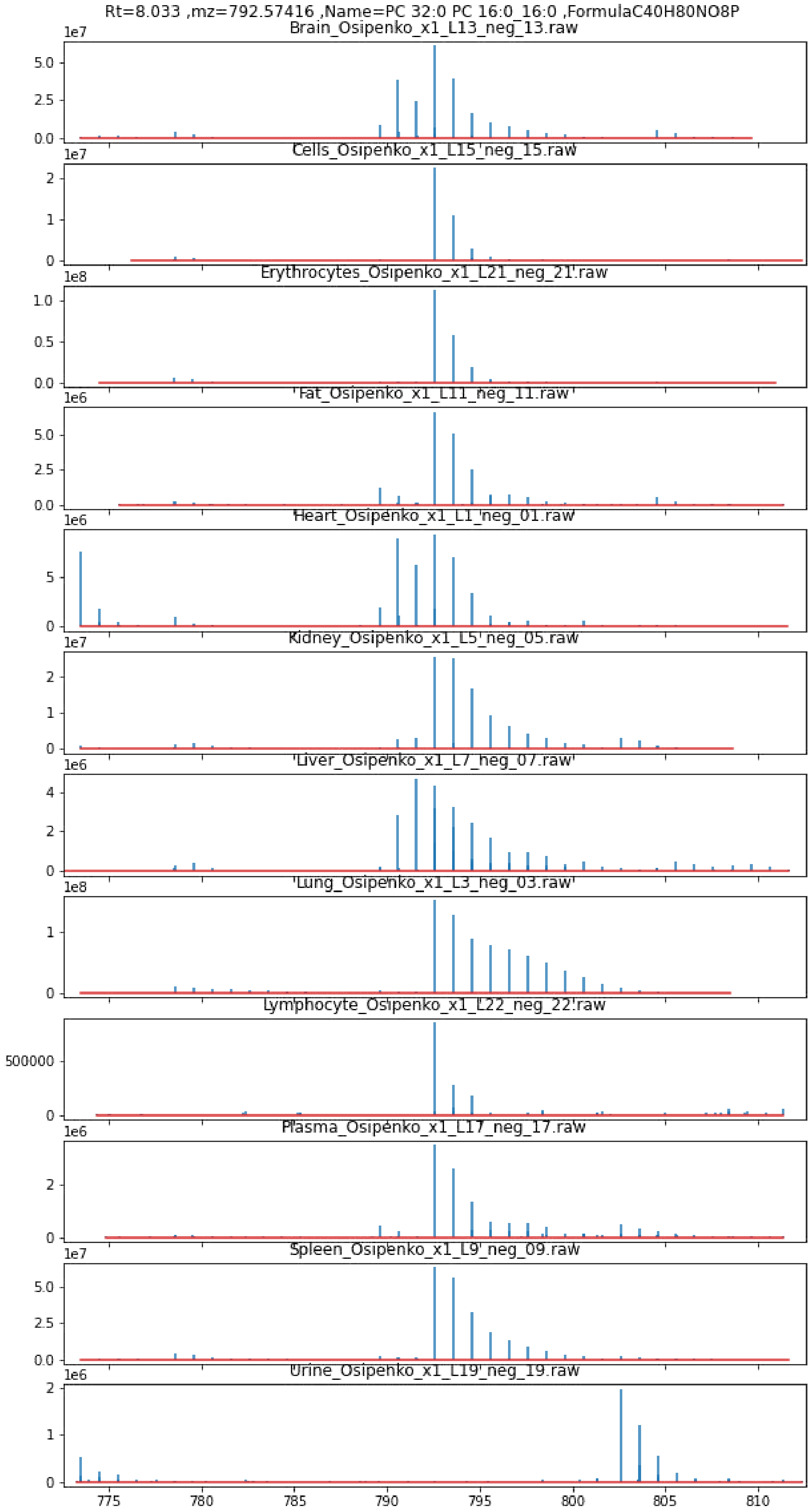
Rt=8.033 ,mz=792.57416 ,Name=PC 32:0 PC 16:0_16:0 ,FormulaC40H80NO8P
Brain_Osipenko_x1_L13_neg_13.raw
Cells_Osipenko_x1_L15_neg_15.raw
Erythrocytes_Osipenko_x1_L21_neg_21.raw
Fat_Osipenko_x1_L11_neg_11.raw
Heart_Osipenko_x1_L1_neg_01.raw
Kidney_Osipenko_x1_L5_neg_05.raw
Liver_Osipenko_x1_L7_neg_07.raw
Lung_Osipenko_x1_L3_neg_03.raw
Lymphocyte_Osipenko_x1_L22_neg_22.raw
Plasma_Osipenko_x1_L17_neg_17.raw
Spleen_Osipenko_x1_L9_neg_09.raw
Urine_Osipenko_x1_L19_neg_19.raw
775 780 785 790 795 800 805 810

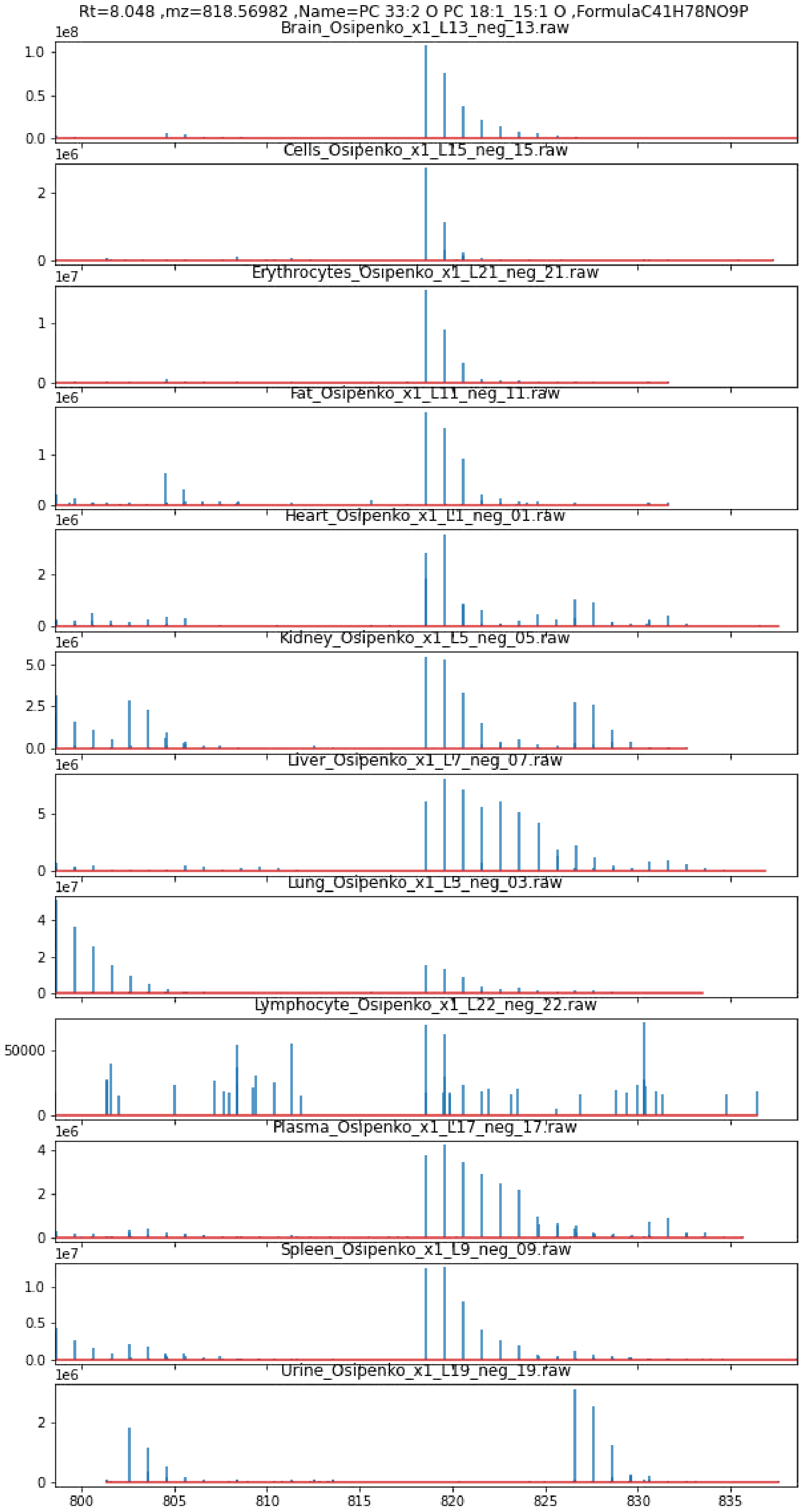
Rt=8.048 ,mz=818.56982 ,Name=PC 33:2 O PC 18:1_15:1 O ,FormulaC41H78NO9P
Brain_Osipenko_x1_L13_neg_13.raw
Cells_Osipenko_x1_L15_neg_15.raw
Erythrocytes_Osipenko_x1_L21_neg_21.raw
Fat_Osipenko_x1_L11_neg_11.raw
Heart_Osipenko_x1_L1_neg_01.raw
Kidney_Osipenko_x1_L5_neg_05.raw
Liver_Osipenko_x1_L7_neg_07.raw
Lung_Osipenko_x1_L3_neg_03.raw
Lymphocyte_Osipenko_x1_L22_neg_22.raw
Plasma_Osipenko_x1_L17_neg_17.raw
Spleen_Osipenko_x1_L9_neg_09.raw
Urine_Osipenko_x1_L19_neg_19.raw
800
805
810
815
820
825
830
835

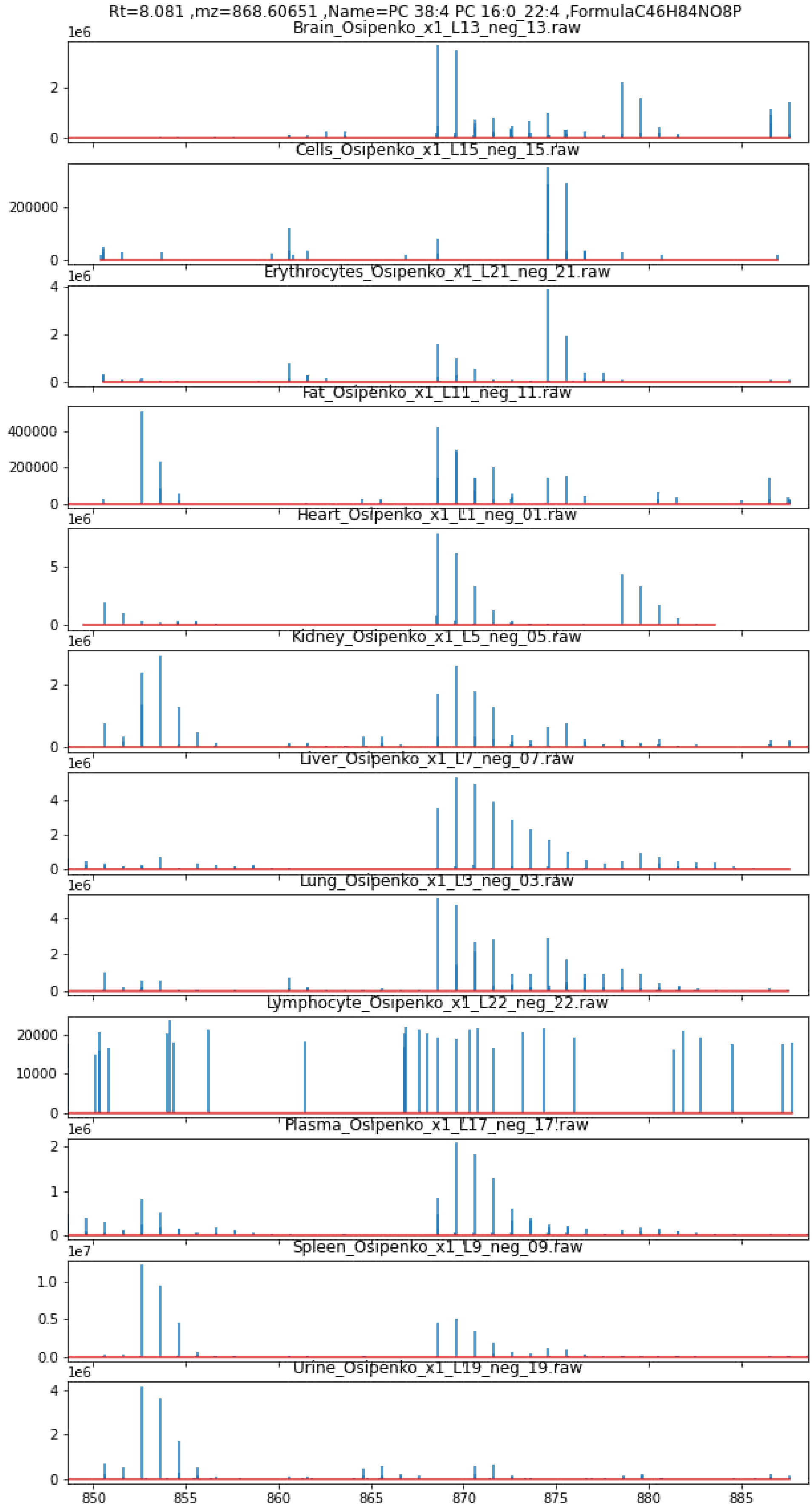
Rt=8.081 ,mz=868.60651 ,Name=PC 38:4 PC 16:0_22:4 ,FormulaC46H84NO8P
Brain_Osipenko_x1_L13_neg_13.raw
Cells_Osipenko_x1_L15_neg_15.raw
Erythrocytes_Osipenko_x1_L21_neg_21.raw
Fat_Osipenko_x1_L11_neg_11.raw
Heart_Osipenko_x1_L1_neg_01.raw
Kidney_Osipenko_x1_L5_neg_05.raw
Liver_Osipenko_x1_L7_neg_07.raw
Lung_Osipenko_x1_L3_neg_03.raw
Lymphocyte_Osipenko_x1_L22_neg_22.raw
Plasma_Osipenko_x1_L17_neg_17.raw
Spleen_Osipenko_x1_L9_neg_09.raw
Urine_Osipenko_x1_L19_neg_19.raw
850 855 860 865 870 875 880 885

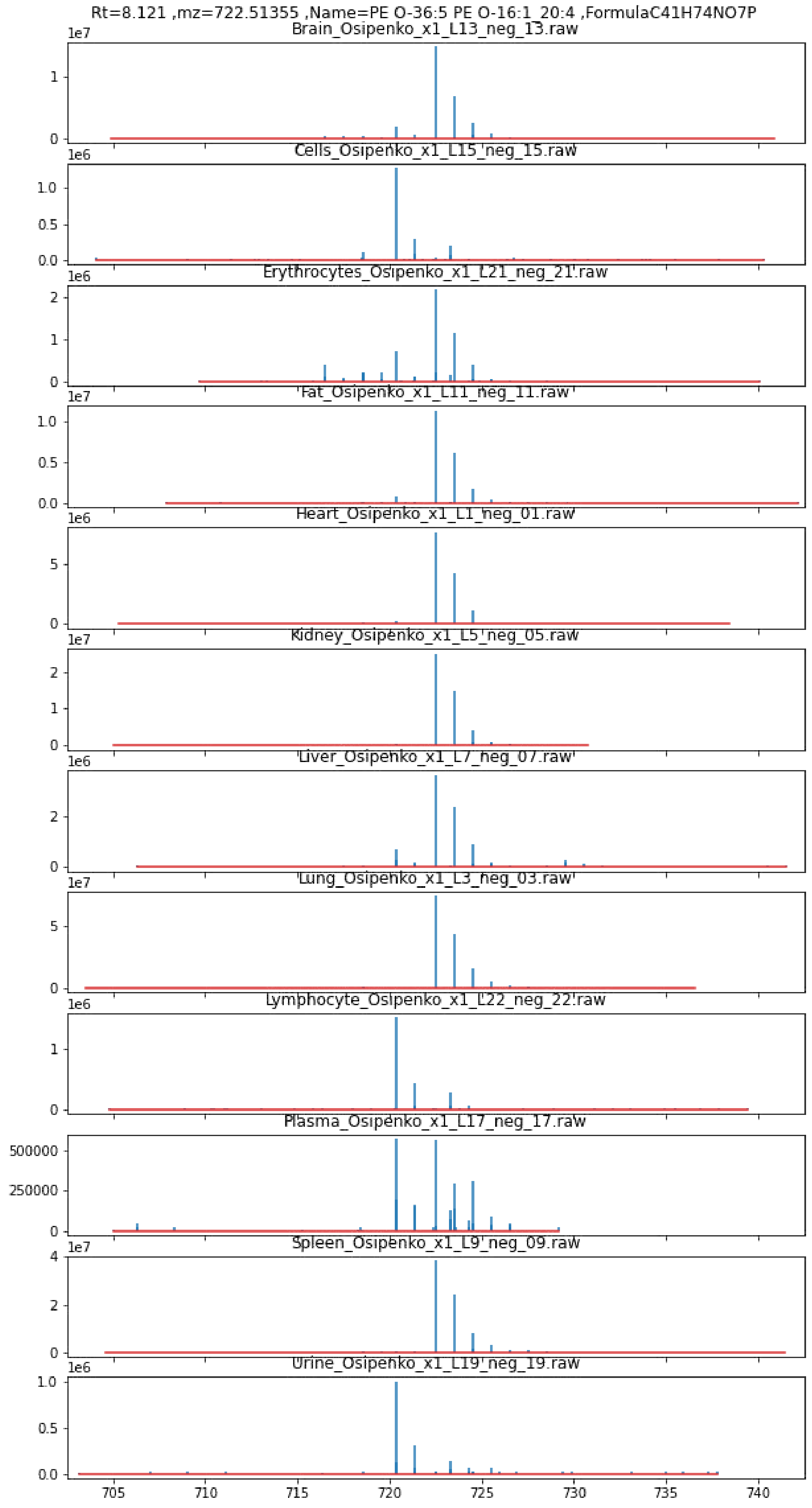
Rt=8.121 ,mz=722.51355 ,Name=PE O-36:5 PE O-16:1_20:4 ,FormulaC41H74NO7P
Brain_Osipenko_x1_L13_neg_13.raw
1e7
Cells_Osipenko_x1_L15_neg_15.raw
1e6
Erythrocytes_Osipenko_x1_L21_neg_21.raw
1e6
Fat_Osipenko_x1_L11_neg_11.raw
1e7
Heart_Osipenko_x1_L1_neg_01.raw
1e6
Kidney_Osipenko_x1_L5_neg_05.raw
1e7
Liver_Osipenko_x1_L7_neg_07.raw
1e6
Lung_Osipenko_x1_L3_neg_03.raw
1e7
Lymphocyte_Osipenko_x1_L22_neg_22.raw
1e6
Plasma_Osipenko_x1_L17_neg_17.raw
500000
250000
Spleen_Osipenko_x1_L9_neg_09.raw
1e7
Urine_Osipenko_x1_L19_neg_19.raw
1e6
705
710
715
720
725
730
735
740

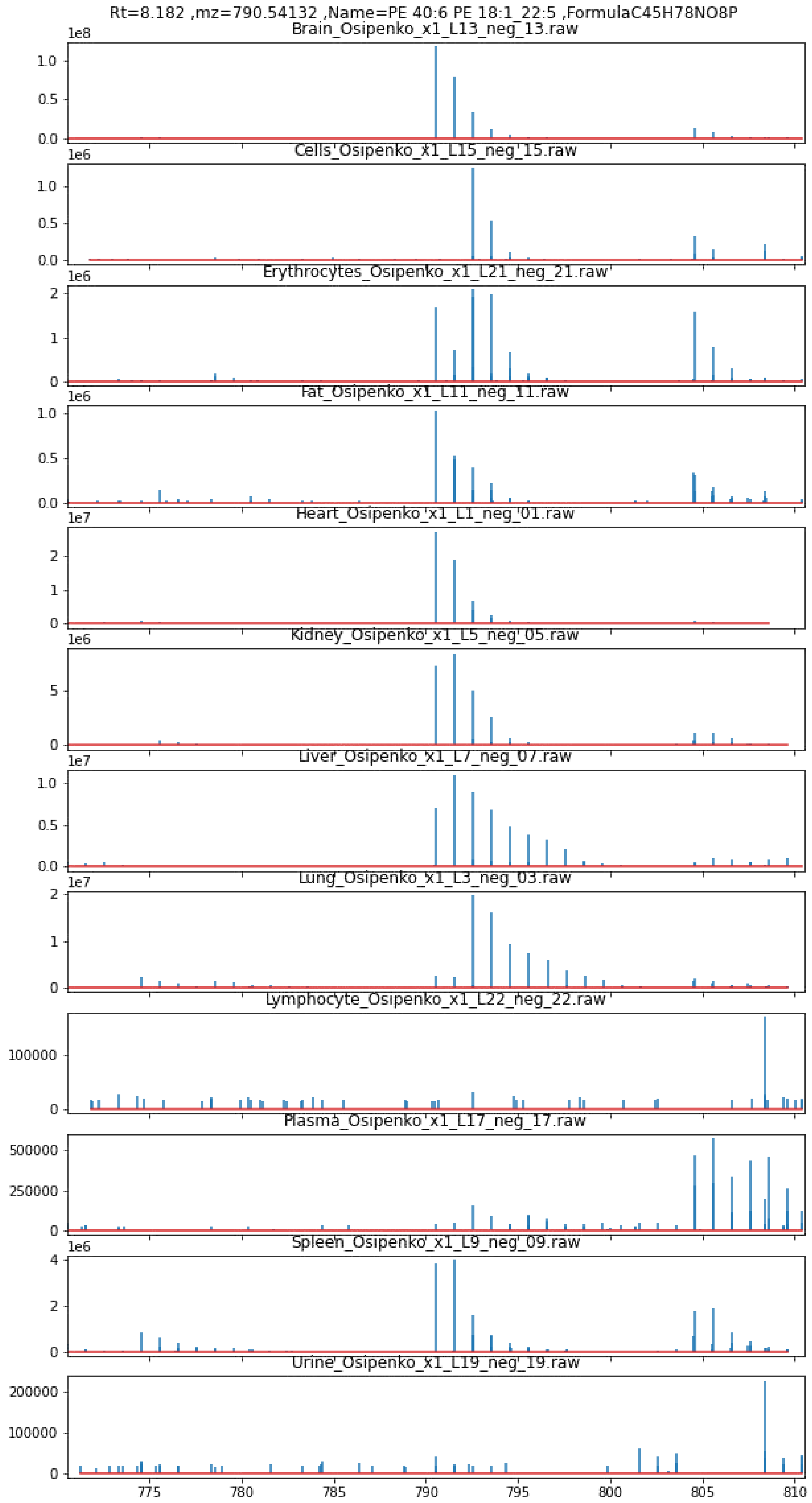
Rt=8.182 ,mz=790.54132 ,Name=PE 40:6 PE 18:1_22:5 ,FormulaC45H78NO8P
Brain_Osipenko_x1_L13_neg_13.raw
Cells_Osipenko_x1_L15_neg_15.raw
Erythrocytes_Osipenko_x1_L21_neg_21.raw
Fat_Osipenko_x1_L11_neg_11.raw
Heart_Osipenko_x1_L1_neg_01.raw
Kidney_Osipenko_x1_L5_neg_05.raw
Liver_Osipenko_x1_L7_neg_07.raw
Lung_Osipenko_x1_L3_neg_03.raw
Lymphocyte_Osipenko_x1_L22_neg_22.raw
Plasma_Osipenko_x1_L17_neg_17.raw
Spleen_Osipenko_x1_L9_neg_09.raw
Urine_Osipenko_x1_L19_neg_19.raw
775
780
785
790
795
800
805
810

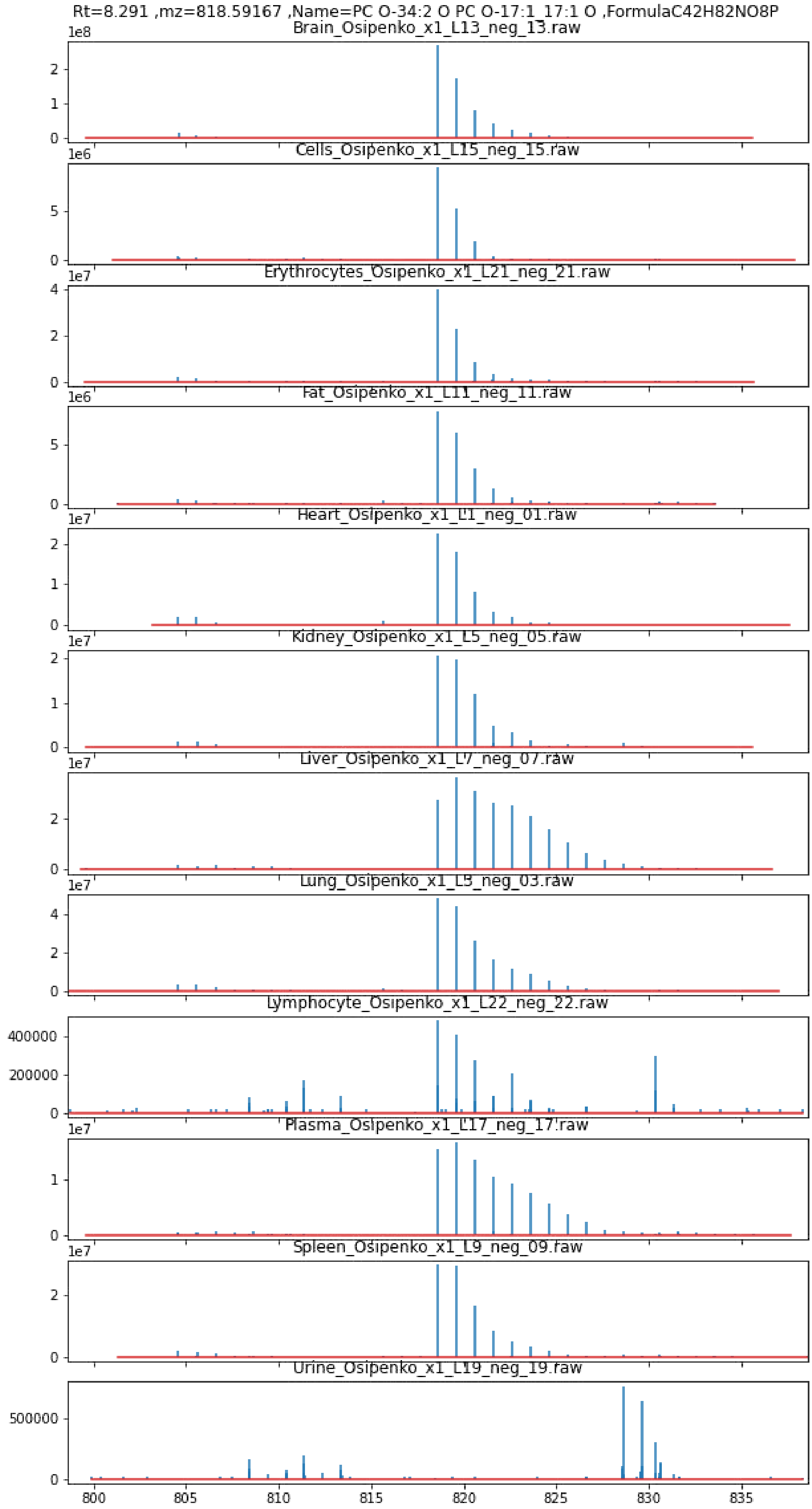

Rt=8.291 ,mz=818.59167 ,Name=PC O-34:2 O PC O-17:1_17:1 O ,FormulaC42H82NO8P
Brain_Osipenko_x1_L13_neg_13.raw
Cells_Osipenko_x1_L15_neg_15.raw
Erythrocytes_Osipenko_x1_L21_neg_21.raw
Fat_Osipenko_x1_L11_neg_11.raw
Heart_Osipenko_x1_L1_neg_01.raw
Kidney_Osipenko_x1_L5_neg_05.raw
Liver_Osipenko_x1_L7_neg_07.raw
Lung_Osipenko_x1_L3_neg_03.raw
Lymphocyte_Osipenko_x1_L22_neg_22.raw
Plasma_Osipenko_x1_L17_neg_17.raw
Spleen_Osipenko_x1_L9_neg_09.raw
Urine_Osipenko_x1_L19_neg_19.raw

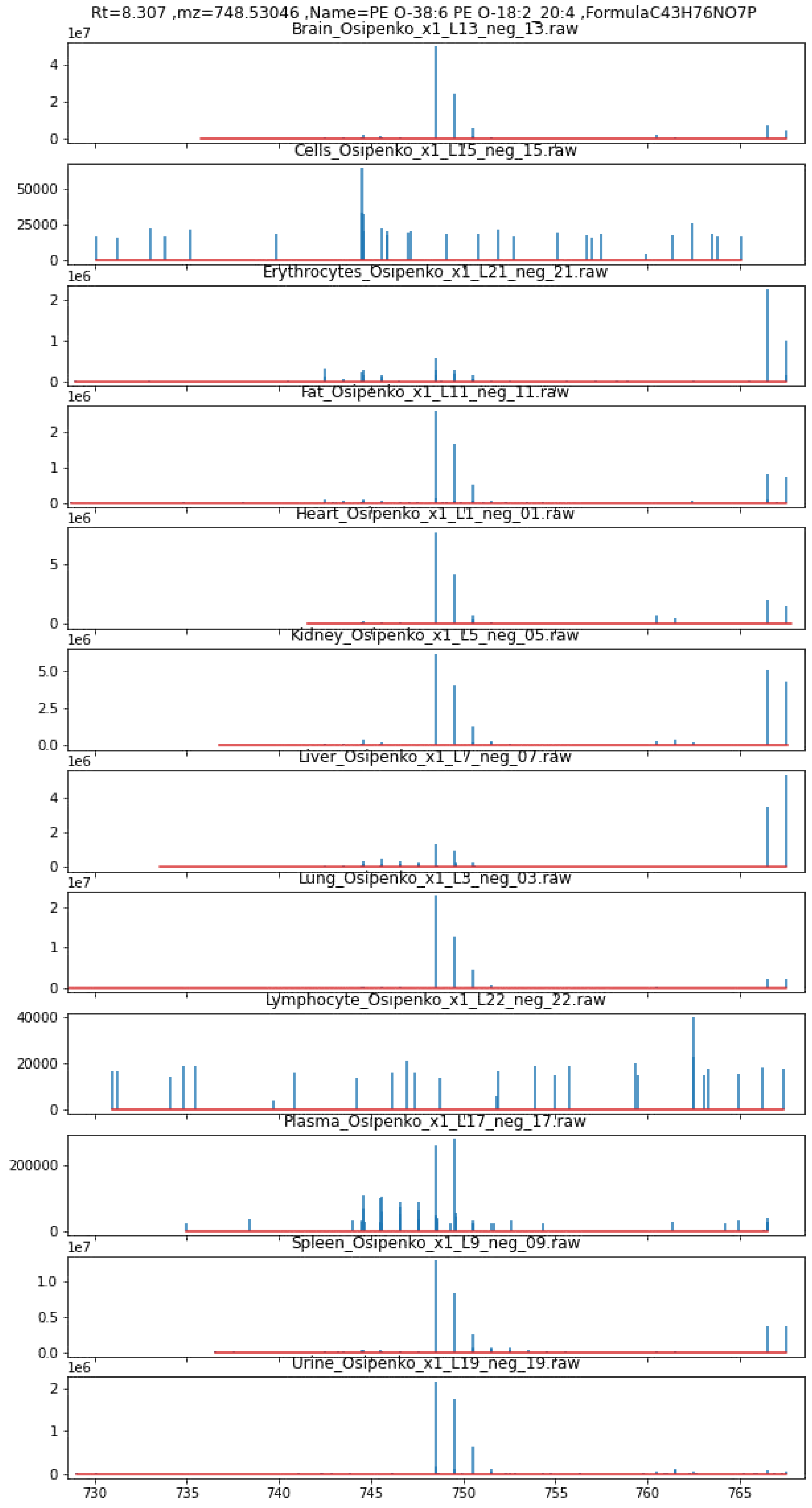
Rt=8.307 ,mz=748.53046 ,Name=PE O-38:6 PE O-18:2_20:4 ,FormulaC43H76NO7P
Brain_Osipenko_x1_L13_neg_13.raw
Cells_Osipenko_x1_L15_neg_15.raw
Erythrocytes_Osipenko_x1_L21_neg_21.raw
Fat_Osipenko_x1_L11_neg_11.raw
Heart_Osipenko_x1_L1_neg_01.raw
Kidney_Osipenko_x1_L5_neg_05.raw
Liver_Osipenko_x1_L7_neg_07.raw
Lung_Osipenko_x1_L3_neg_03.raw
Lymphocyte_Osipenko_x1_L22_neg_22.raw
Plasma_Osipenko_x1_L17_neg_17.raw
Spleen_Osipenko_x1_L9_neg_09.raw
Urine_Osipenko_x1_L19_neg_19.raw
730
735
740
745
750
755
760
765

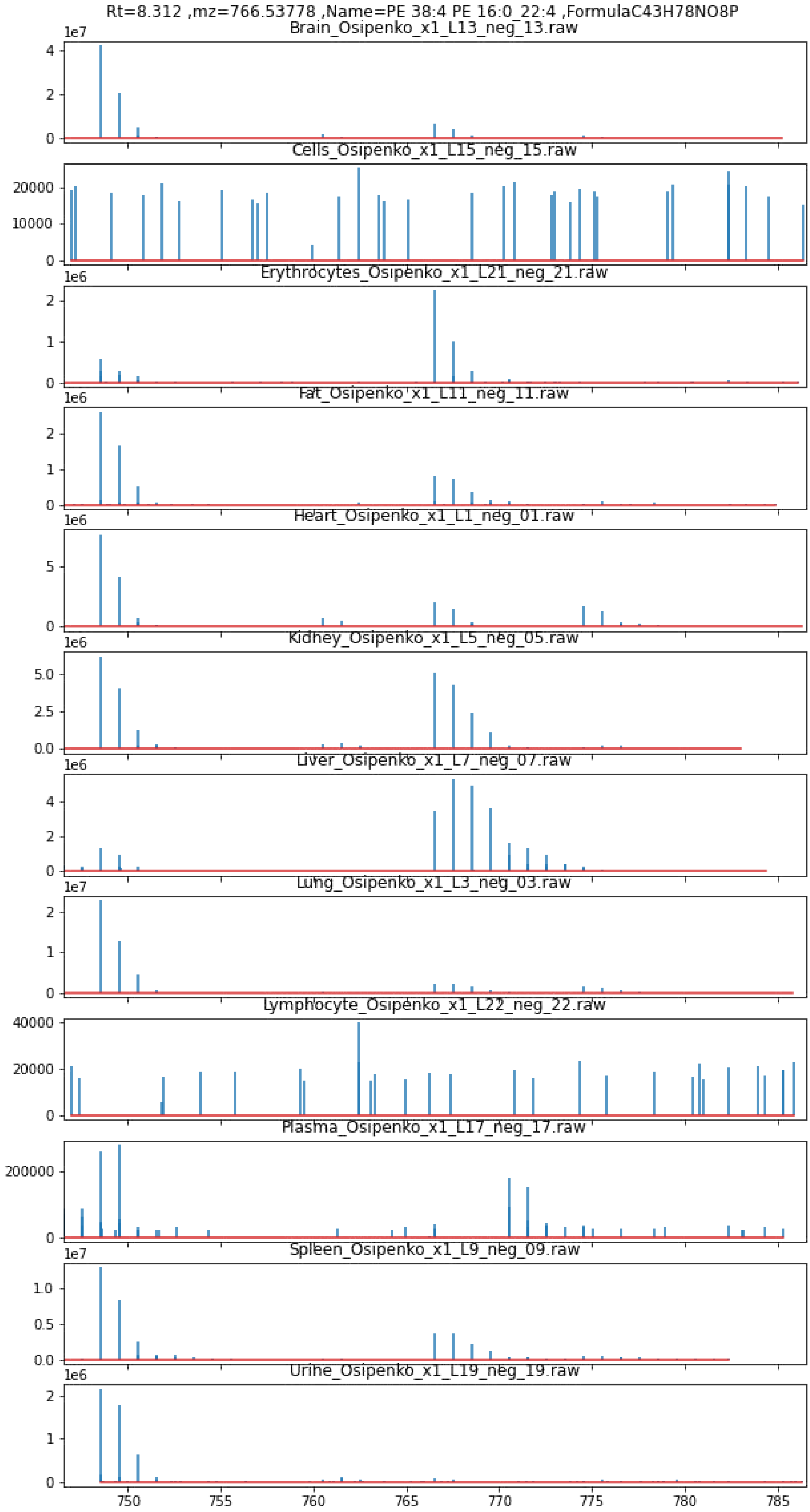

Rt=8.312 ,mz=766.53778 ,Name=PE 38:4 PE 16:0_22:4 ,FormulaC43H78NO8P
Brain_Osipenko_x1_L13_neg_13.raw
Cells_Osipenko_x1_L15_neg_15.raw
Erythrocytes_Osipenko_x1_L21_neg_21.raw
Fat_Osipenko_x1_L11_neg_11.raw
Heart_Osipenko_x1_L1_neg_01.raw
Kidney_Osipenko_x1_L5_neg_05.raw
Liver_Osipenko_x1_L7_neg_07.raw
Lung_Osipenko_x1_L3_neg_03.raw
Lymphocyte_Osipenko_x1_L22_neg_22.raw
Plasma_Osipenko_x1_L17_neg_17.raw
Spleen_Osipenko_x1_L9_neg_09.raw
Urine_Osipenko_x1_L19_neg_19.raw
750
755
760
765
770
775
780
785

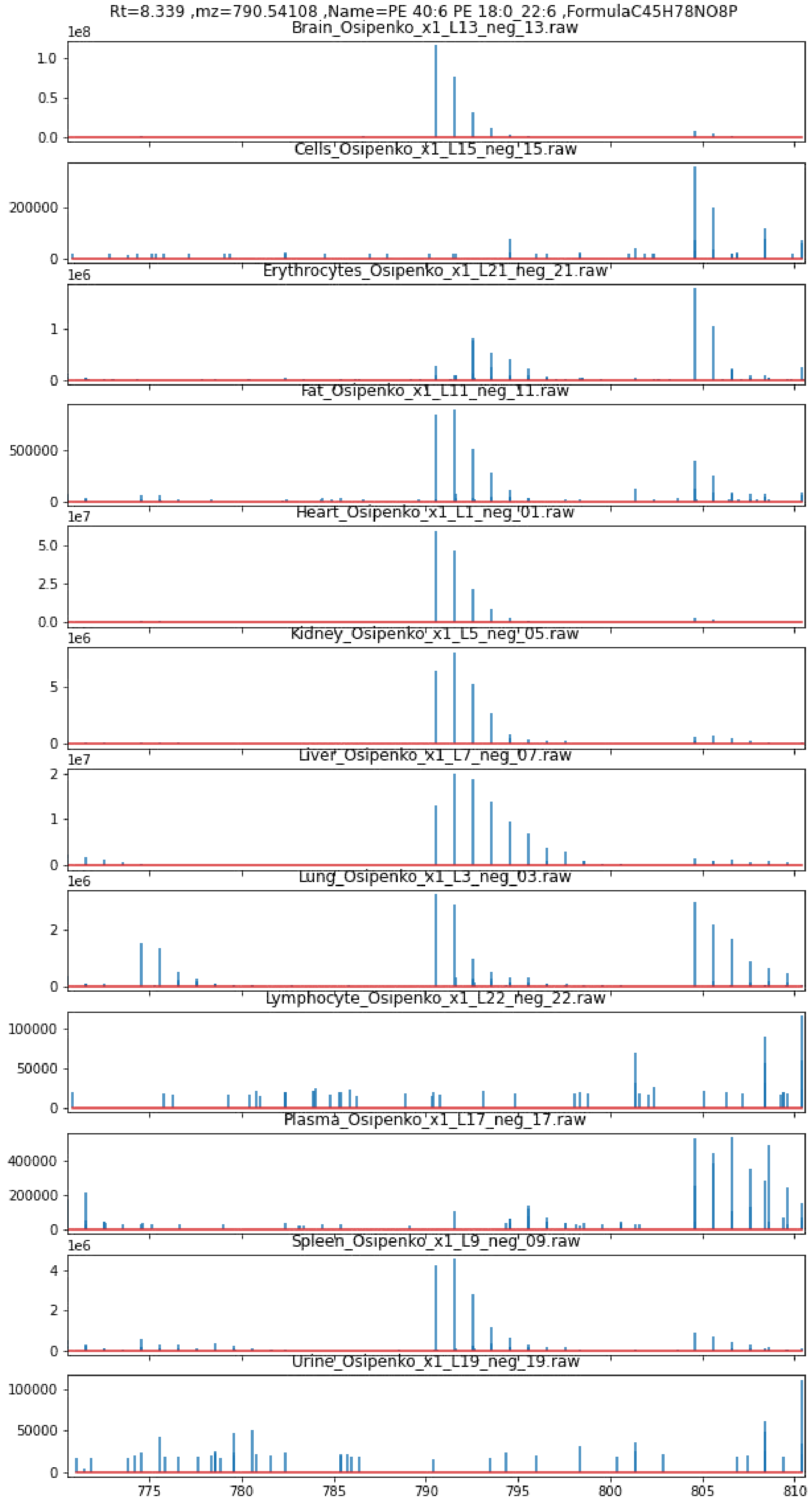

Rt=8.339 ,mz=790.54108 ,Name=PE 40:6 PE 18:0_22:6 ,FormulaC45H78NO8P
Brain_Osipenko_x1_L13_neg_13.raw
Cells_Osipenko_x1_L15_neg_15.raw
Erythrocytes_Osipenko_x1_L21_neg_21.raw
Fat_Osipenko_x1_L11_neg_11.raw
Heart_Osipenko_x1_L1_neg_01.raw
Kidney_Osipenko_x1_L5_neg_05.raw
Liver_Osipenko_x1_L7_neg_07.raw
Lung_Osipenko_x1_L3_neg_03.raw
Lymphocyte_Osipenko_x1_L22_neg_22.raw
Plasma_Osipenko_x1_L17_neg_17.raw
Spleen_Osipenko_x1_L9_neg_09.raw
Urine_Osipenko_x1_L19_neg_19.raw

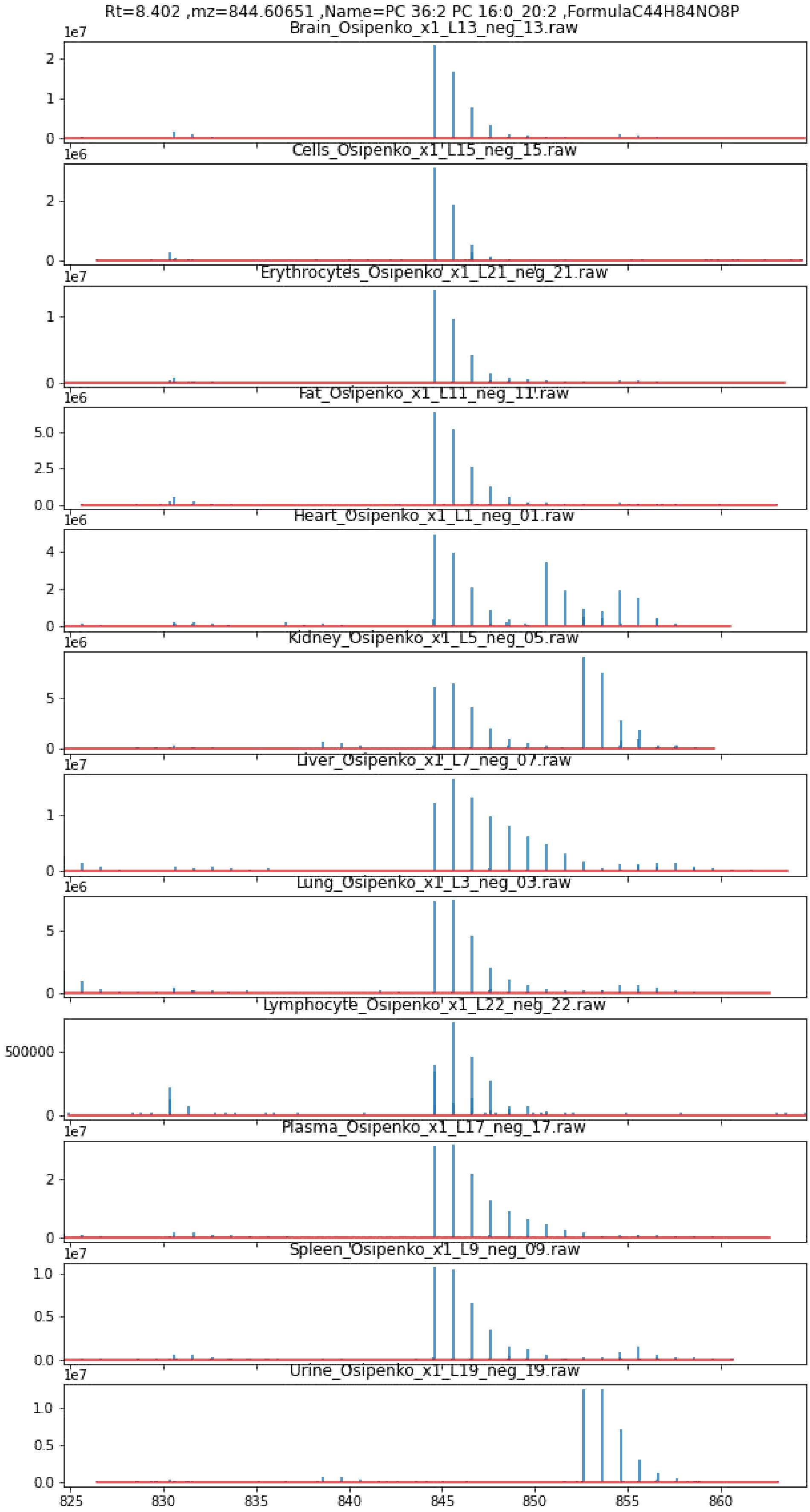

Rt=8.402 ,mz=844.60651 ,Name=PC 36:2 PC 16:0_20:2 ,FormulaC44H84NO8P
Brain_Osipenko_x1_L13_neg_13.raw
Cells_Osipenko_x1_L15_neg_15.raw
Erythrocytes_Osipenko_x1_L21_neg_21.raw
Fat_Osipenko_x1_L11_neg_11.raw
Heart_Osipenko_x1_L1_neg_01.raw
Kidney_Osipenko_x1_L5_neg_05.raw
Liver_Osipenko_x1_L7_neg_07.raw
Lung_Osipenko_x1_L3_neg_03.raw
Lymphocyte_Osipenko_x1_L22_neg_22.raw
Plasma_Osipenko_x1_L17_neg_17.raw
Spleen_Osipenko_x1_L9_neg_09.raw
Urine_Osipenko_x1_L19_neg_19.raw
825
830
835
840
845
850
855
860

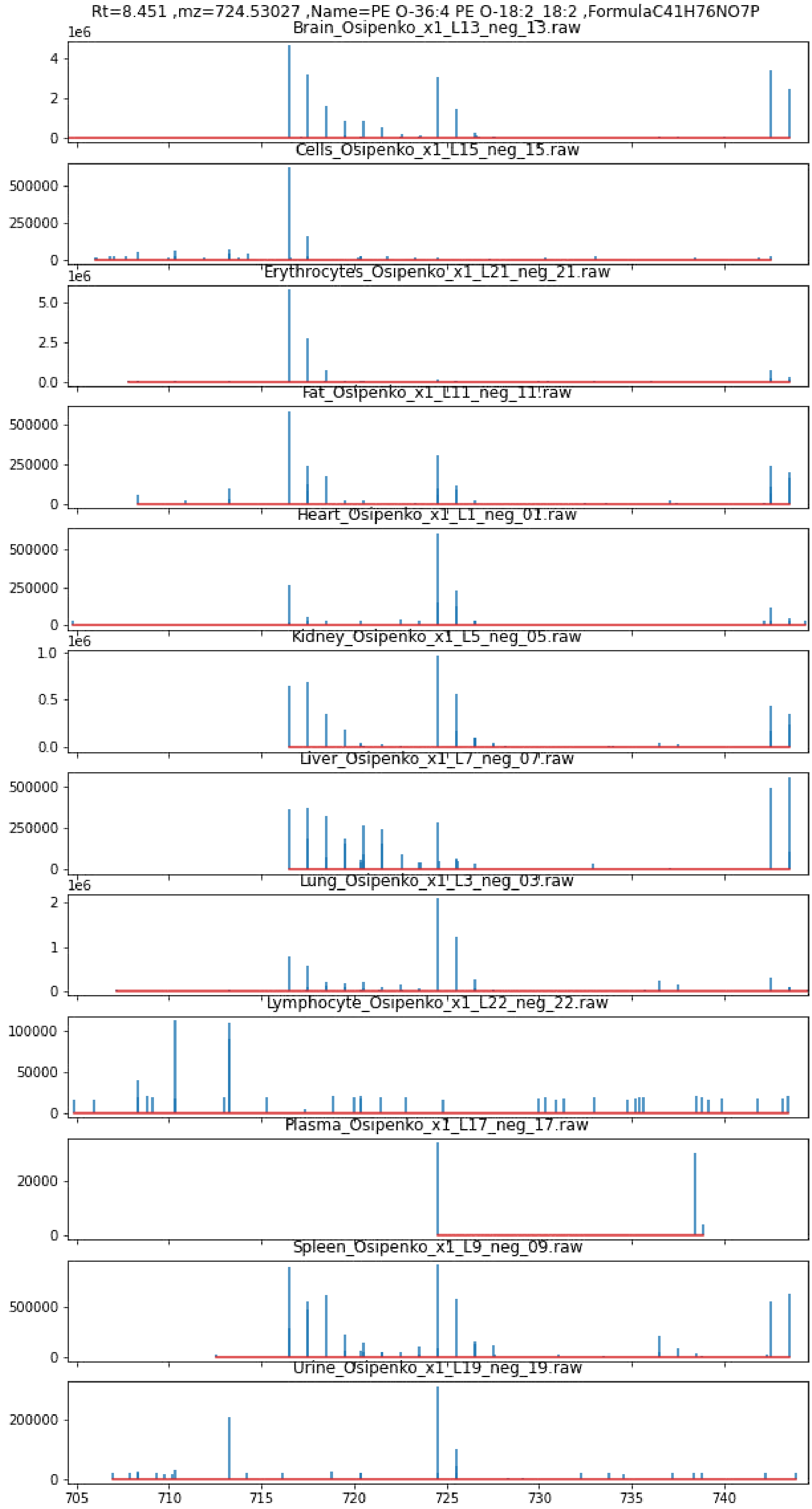

Rt=8.451 ,mz=724.53027 ,Name=PE O-36:4 PE O-18:2_18:2 ,FormulaC41H76NO7P
Brain_Osipenko_x1_L13_neg_13.raw
Cells_Osipenko_x1_L15_neg_15.raw
Erythrocytes_Osipenko_x1_L21_neg_21.raw
Fat_Osipenko_x1_L11_neg_11.raw
Heart_Osipenko_x1_L1_neg_01.raw
Kidney_Osipenko_x1_L5_neg_05.raw
Liver_Osipenko_x1_L7_neg_07.raw
Lung_Osipenko_x1_L3_neg_03.raw
Lymphocyte_Osipenko_x1_L22_neg_22.raw
Plasma_Osipenko_x1_L17_neg_17.raw
Spleen_Osipenko_x1_L9_neg_09.raw
Urine_Osipenko_x1_L19_neg_19.raw
1e6
705
710
715
720
725
730
735
740

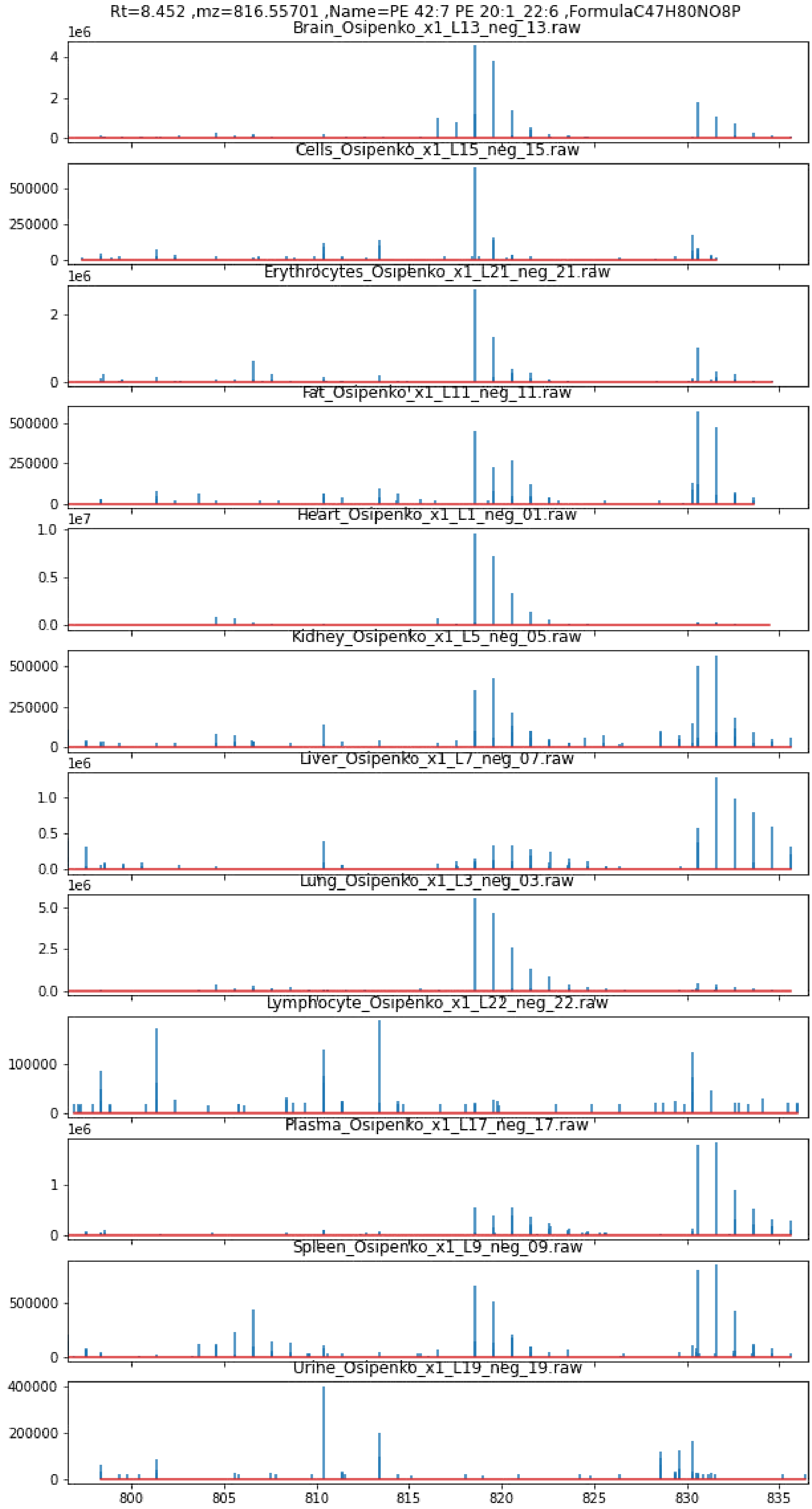
Rt=8.452 ,mz=816.55701 ,Name=PE 42:7 PE 20:1_22:6 ,FormulaC47H80NO8P
Brain_Osipenko_x1_L13_neg_13.raw
Cells_Osipenko_x1_L15_neg_15.raw
Erythrocytes_Osipenko_x1_L21_neg_21.raw
Fat_Osipenko_x1_L11_neg_11.raw
Heart_Osipenko_x1_L1_neg_01.raw
Kidney_Osipenko_x1_L5_neg_05.raw
Liver_Osipenko_x1_L7_neg_07.raw
Lung_Osipenko_x1_L3_neg_03.raw
Lymphocyte_Osipenko_x1_L22_neg_22.raw
Plasma_Osipenko_x1_L17_neg_17.raw
Spleen_Osipenko_x1_L9_neg_09.raw
Urine_Osipenko_x1_L19_neg_19.raw
800
805
810
815
820
825
830
835

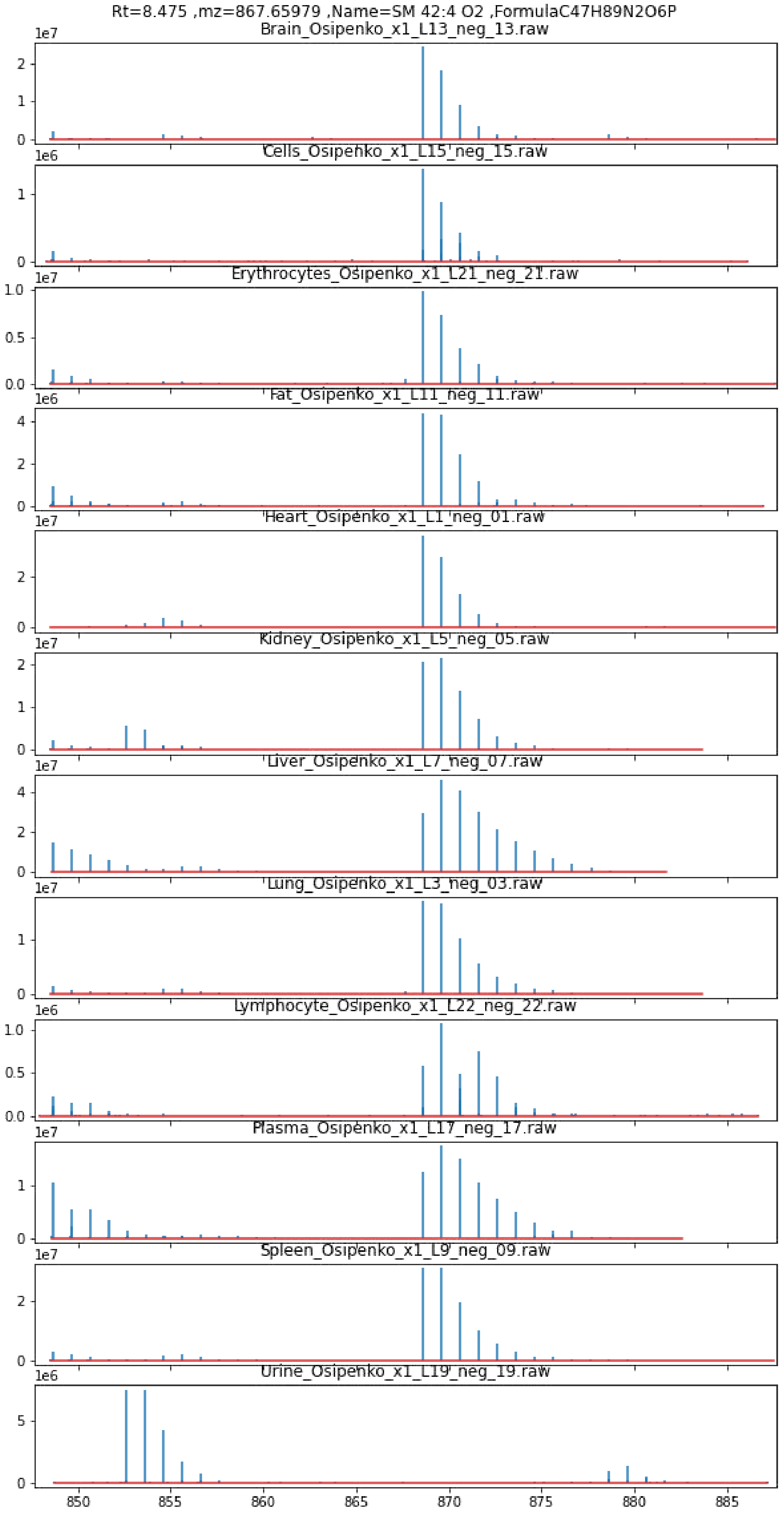
Rt=8.475 ,mz=867.65979 ,Name=SM 42:4 O2 ,FormulaC47H89N2O6P
Brain_Osipenko_x1_L13_neg_13.raw
Cells_Osipenko_x1_L15_neg_15.raw
Erythrocytes_Osipenko_x1_L21_neg_21.raw
Fat_Osipenko_x1_L11_neg_11.raw
Heart_Osipenko_x1_L1_neg_01.raw
Kidney_Osipenko_x1_L5_neg_05.raw
Liver_Osipenko_x1_L7_neg_07.raw
Lung_Osipenko_x1_L3_neg_03.raw
Lymphocyte_Osipenko_x1_L22_neg_22.raw
Plasma_Osipenko_x1_L17_neg_17.raw
Spleen_Osipenko_x1_L9_neg_09.raw
Urine_Osipenko_x1_L19_neg_19.raw
1e7
1e6
850
855
860
865
870
875
880
885

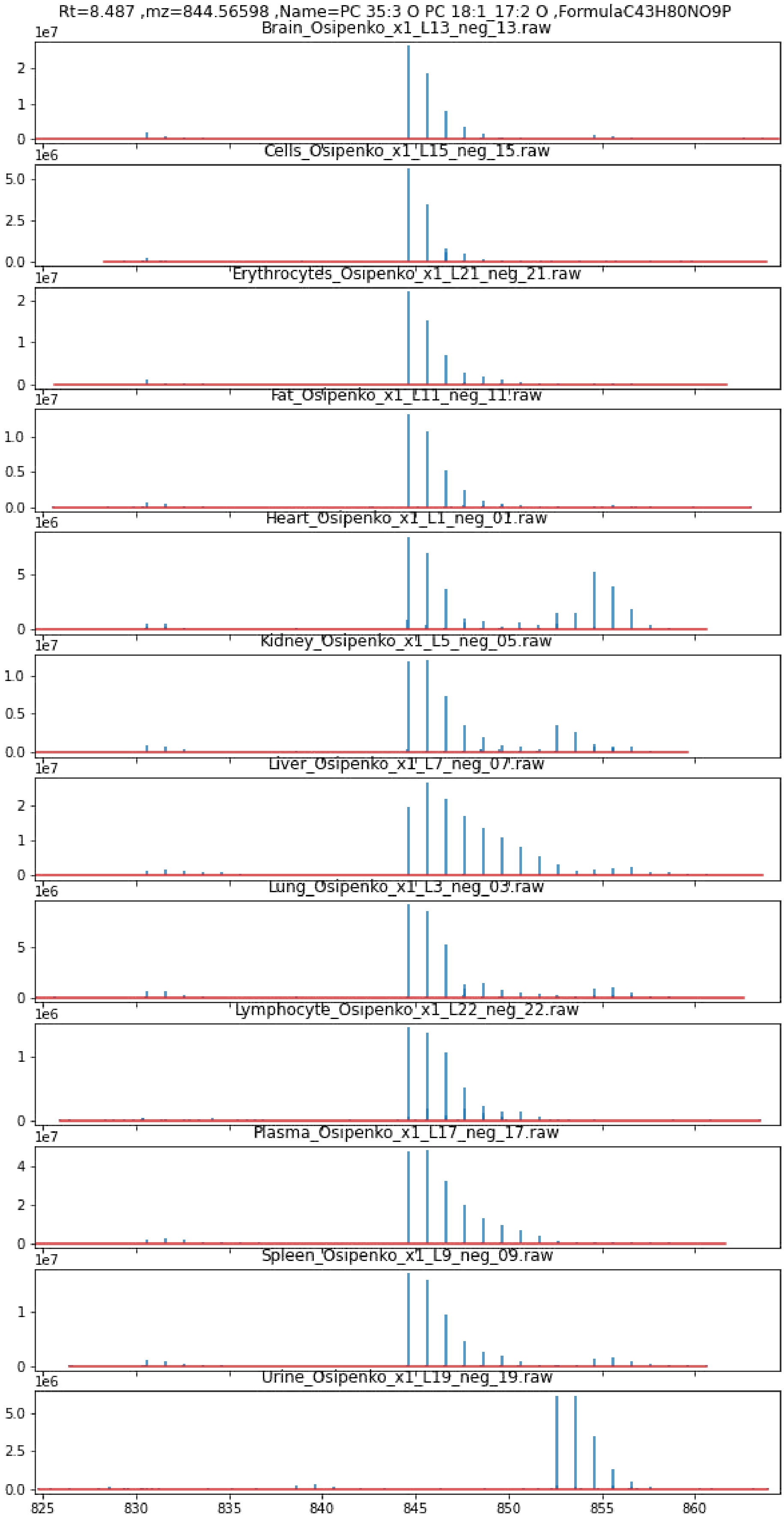

Rt=8.487 ,mz=844.56598 ,Name=PC 35:3 O PC 18:1_17:2 O ,FormulaC43H80NO9P
Brain_Osipenko_x1_L13_neg_13.raw
Cells_Osipenko_x1_L15_neg_15.raw
Erythrocytes_Osipenko_x1_L21_neg_21.raw
Fat_Osipenko_x1_L11_neg_11.raw
Heart_Osipenko_x1_L1_neg_01.raw
Kidney_Osipenko_x1_L5_neg_05.raw
Liver_Osipenko_x1_L7_neg_07.raw
Lung_Osipenko_x1_L3_neg_03.raw
Lymphocyte_Osipenko_x1_L22_neg_22.raw
Plasma_Osipenko_x1_L17_neg_17.raw
Spleen_Osipenko_x1_L9_neg_09.raw
Urine_Osipenko_x1_L19_neg_19.raw
825
830
835
840
845
850
855
860

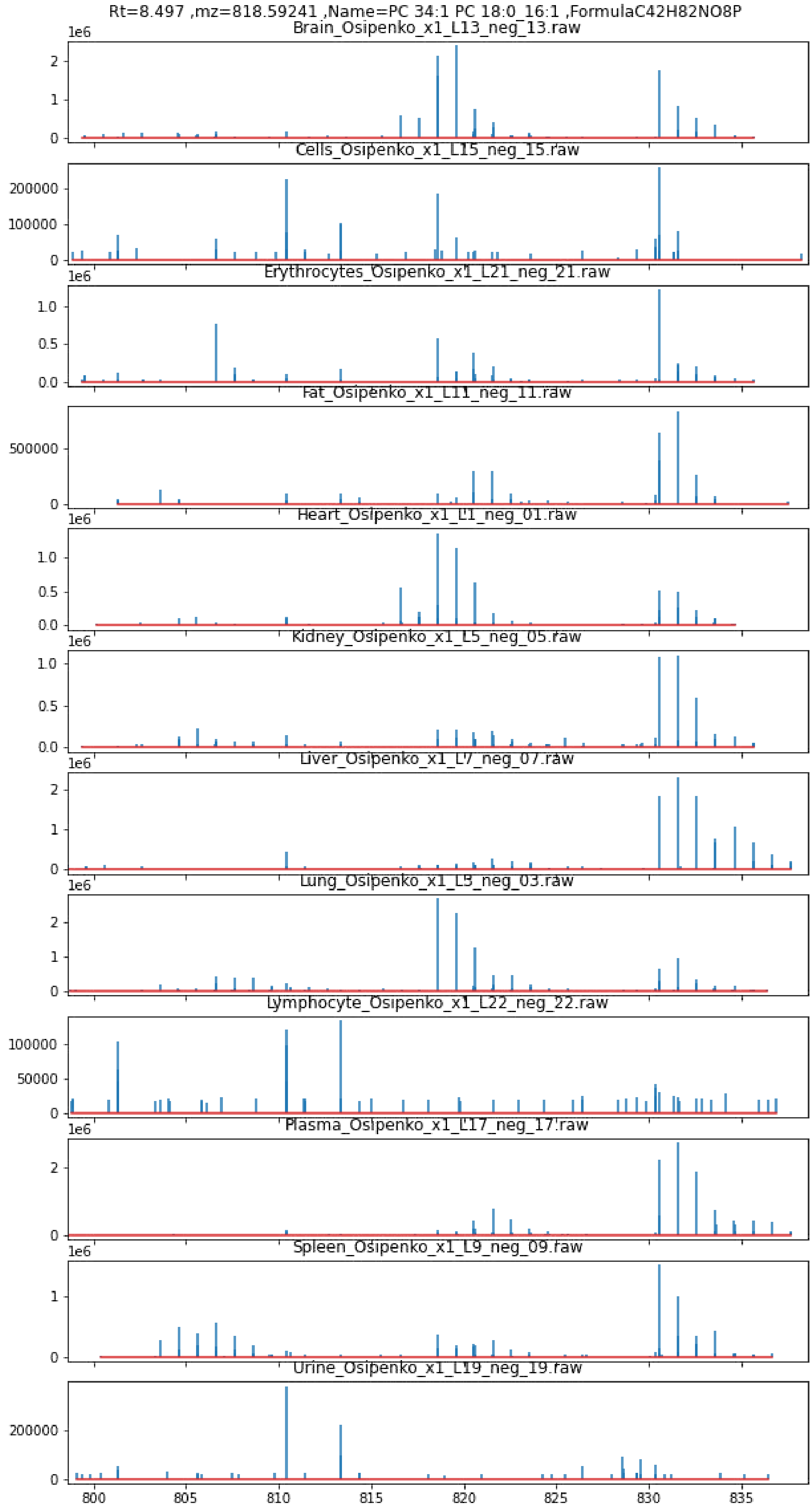
Rt=8.497 ,mz=818.59241 ,Name=PC 34:1 PC 18:0_16:1 ,FormulaC42H82NO8P
Brain_Osipenko_x1_L13_neg_13.raw
Cells_Osipenko_x1_L15_neg_15.raw
Erythrocytes_Osipenko_x1_L21_neg_21.raw
Fat_Osipenko_x1_L11_neg_11.raw
Heart_Osipenko_x1_L1_neg_01.raw
Kidney_Osipenko_x1_L5_neg_05.raw
Liver_Osipenko_x1_L7_neg_07.raw
Lung_Osipenko_x1_L3_neg_03.raw
Lymphocyte_Osipenko_x1_L22_neg_22.raw
Plasma_Osipenko_x1_L17_neg_17.raw
Spleen_Osipenko_x1_L9_neg_09.raw
Urine_Osipenko_x1_L19_neg_19.raw
1e6
800
805
810
815
820
825
830
835

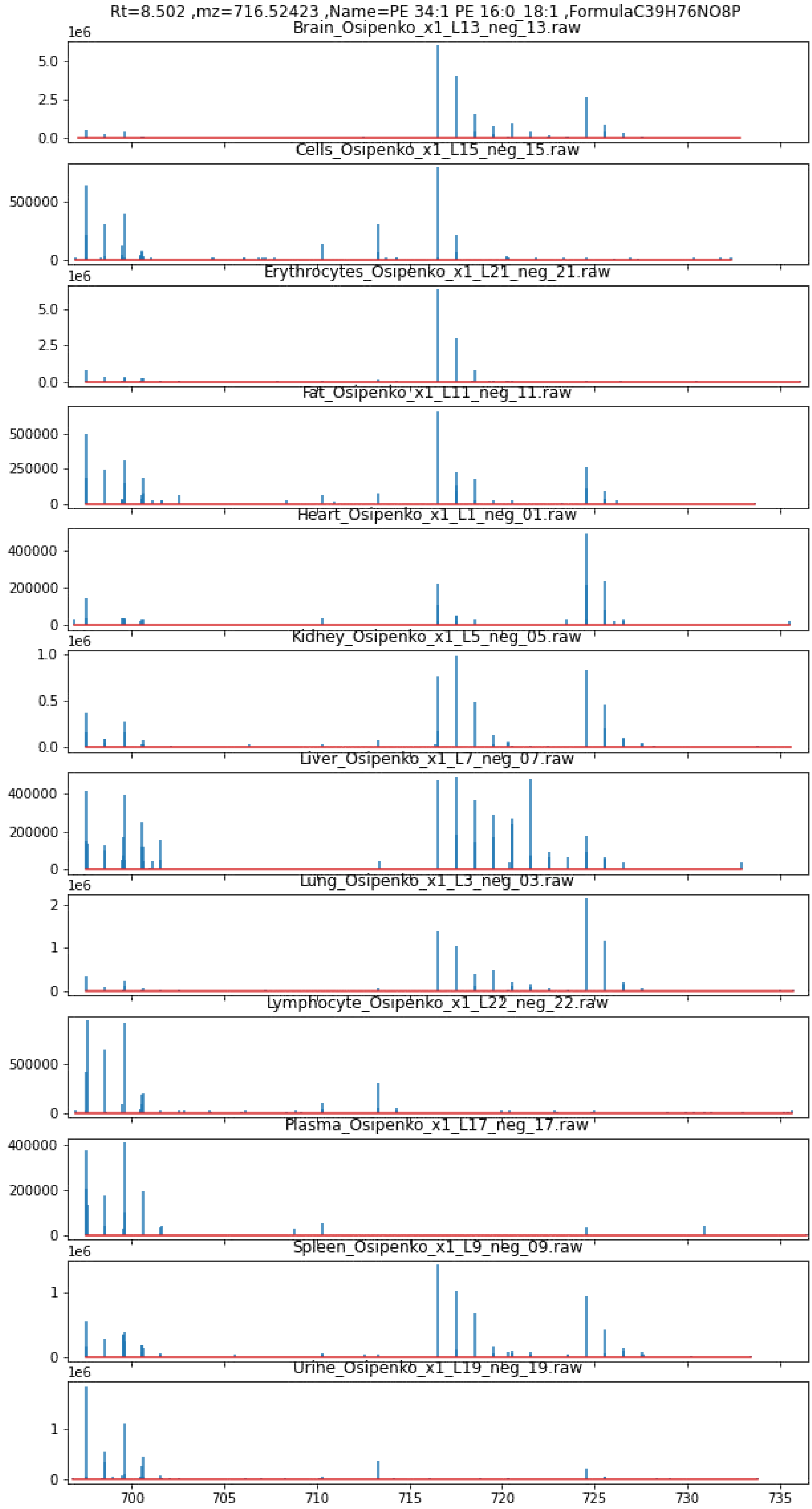

Rt=8.502 ,mz=716.52423 ,Name=PE 34:1 PE 16:0_18:1 ,FormulaC39H76NO8P
Brain_Osipenko_x1_L13_neg_13.raw
Cells_Osipenko_x1_L15_neg_15.raw
Erythrocytes_Osipenko_x1_L21_neg_21.raw
Fat_Osipenko_x1_L11_neg_11.raw
Heart_Osipenko_x1_L1_neg_01.raw
Kidney_Osipenko_x1_L5_neg_05.raw
Liver_Osipenko_x1_L7_neg_07.raw
Lung_Osipenko_x1_L3_neg_03.raw
Lymphocyte_Osipenko_x1_L22_neg_22.raw
Plasma_Osipenko_x1_L17_neg_17.raw
Spleen_Osipenko_x1_L9_neg_09.raw
Urine_Osipenko_x1_L19_neg_19.raw
700
705
710
715
720
725
730
735

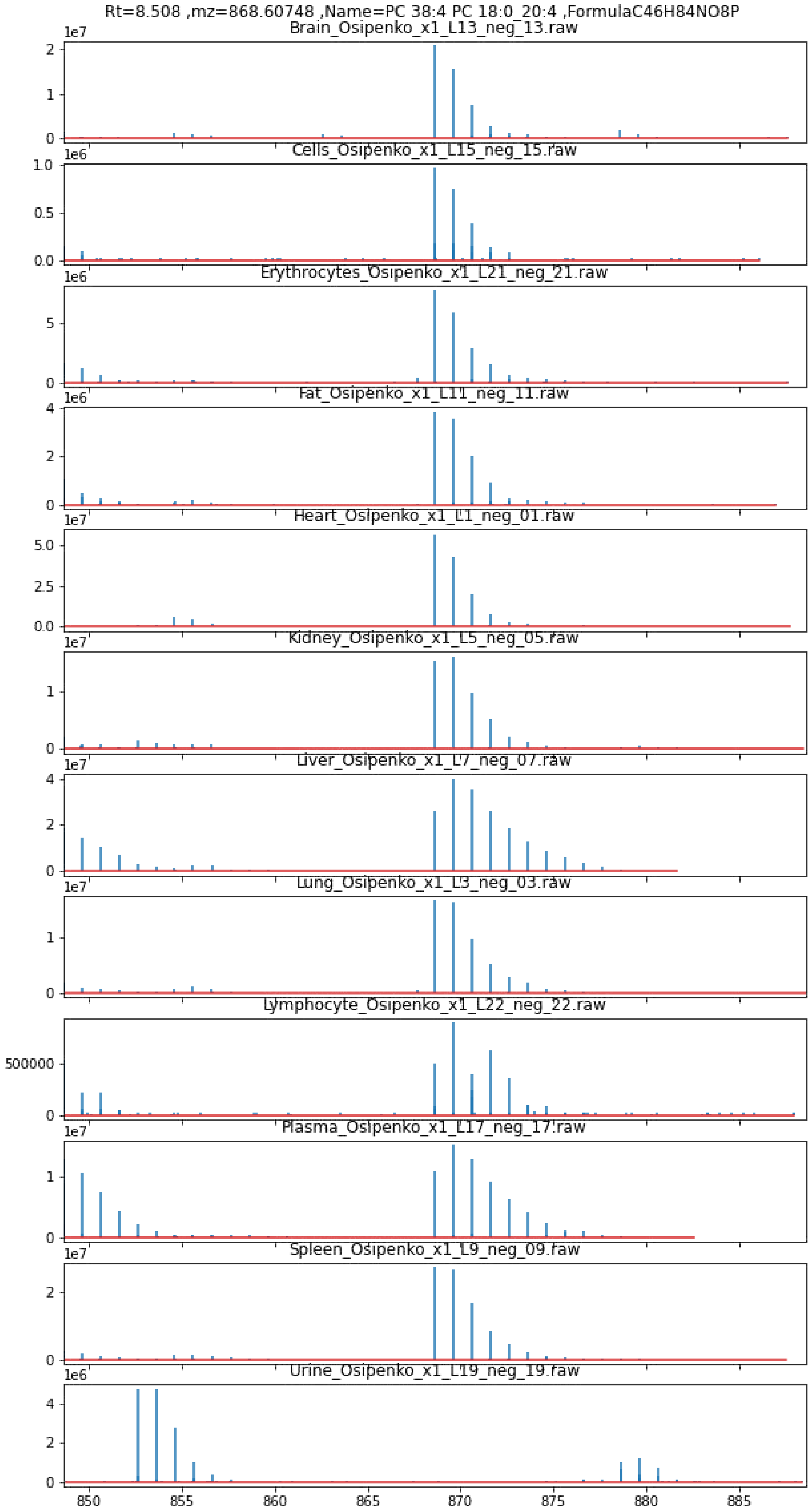
Rt=8.508 ,mz=868.60748 ,Name=PC 38:4 PC 18:0_20:4 ,FormulaC46H84NO8P
Brain_Osipenko_x1_L13_neg_13.raw
Cells_Osipenko_x1_L15_neg_15.raw
Erythrocytes_Osipenko_x1_L21_neg_21.raw
Fat_Osipenko_x1_L11_neg_11.raw
Heart_Osipenko_x1_L1_neg_01.raw
Kidney_Osipenko_x1_L5_neg_05.raw
Liver_Osipenko_x1_L7_neg_07.raw
Lung_Osipenko_x1_L3_neg_03.raw
Lymphocyte_Osipenko_x1_L22_neg_22.raw
Plasma_Osipenko_x1_L17_neg_17.raw
Spleen_Osipenko_x1_L9_neg_09.raw
Urine_Osipenko_x1_L19_neg_19.raw
850
855
860
865
870
875
880
885

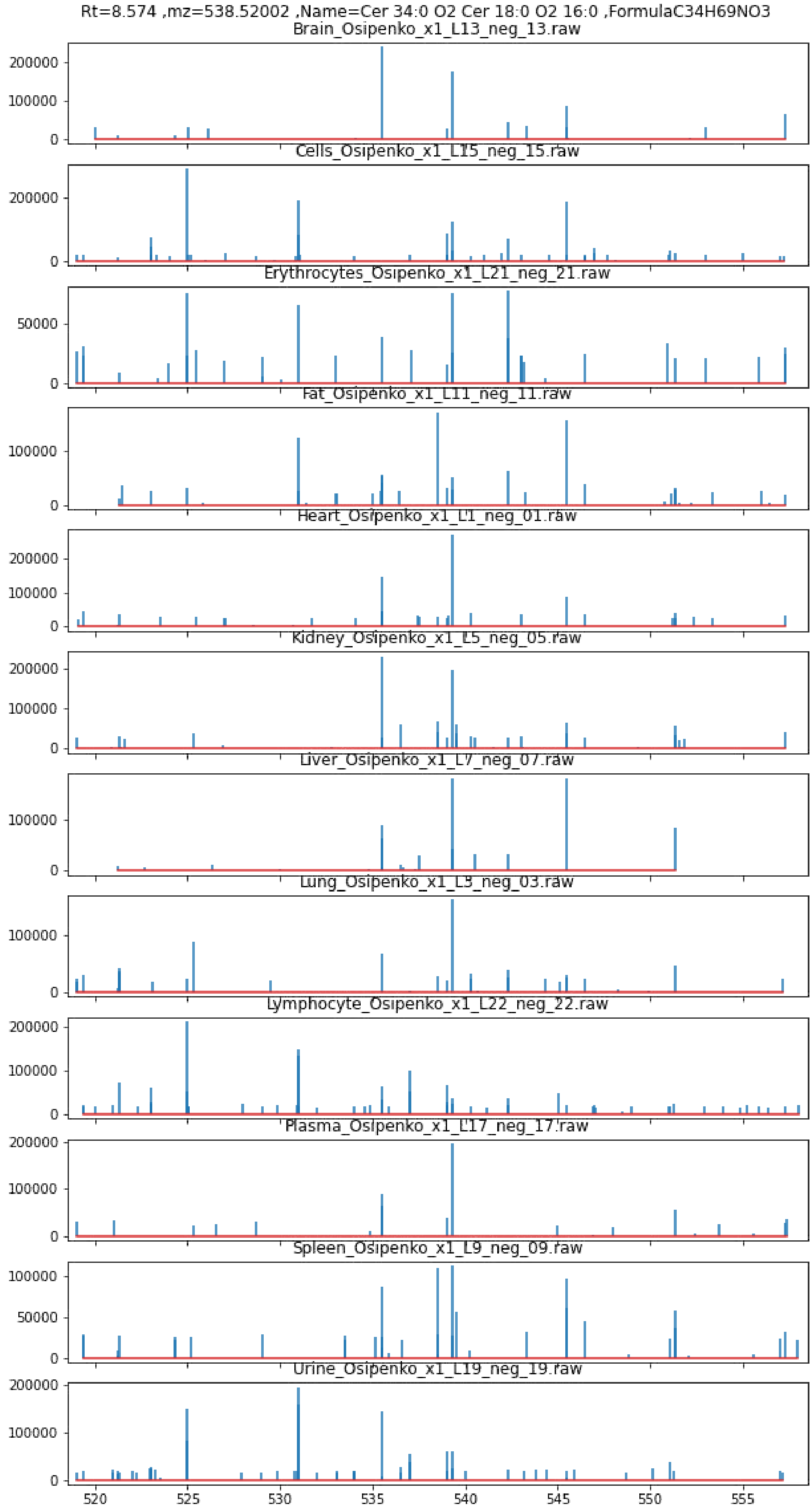

Rt=8.574 ,mz=538.52002 ,Name=Cer 34:0 O2 Cer 18:0 O2 16:0 ,FormulaC34H69NO3
Brain_Osipenko_x1_L13_neg_13.raw
Cells_Osipenko_x1_L15_neg_15.raw
Erythrocytes_Osipenko_x1_L21_neg_21.raw
Fat_Osipenko_x1_L11_neg_11.raw
Heart_Osipenko_x1_L1_neg_01.raw
Kidney_Osipenko_x1_L5_neg_05.raw
Liver_Osipenko_x1_L7_neg_07.raw
Lung_Osipenko_x1_L3_neg_03.raw
Lymphocyte_Osipenko_x1_L22_neg_22.raw
Plasma_Osipenko_x1_L17_neg_17.raw
Spleen_Osipenko_x1_L9_neg_09.raw
Urine_Osipenko_x1_L19_neg_19.raw
0
50000
100000
200000
520
525
530
535
540
545
550
555

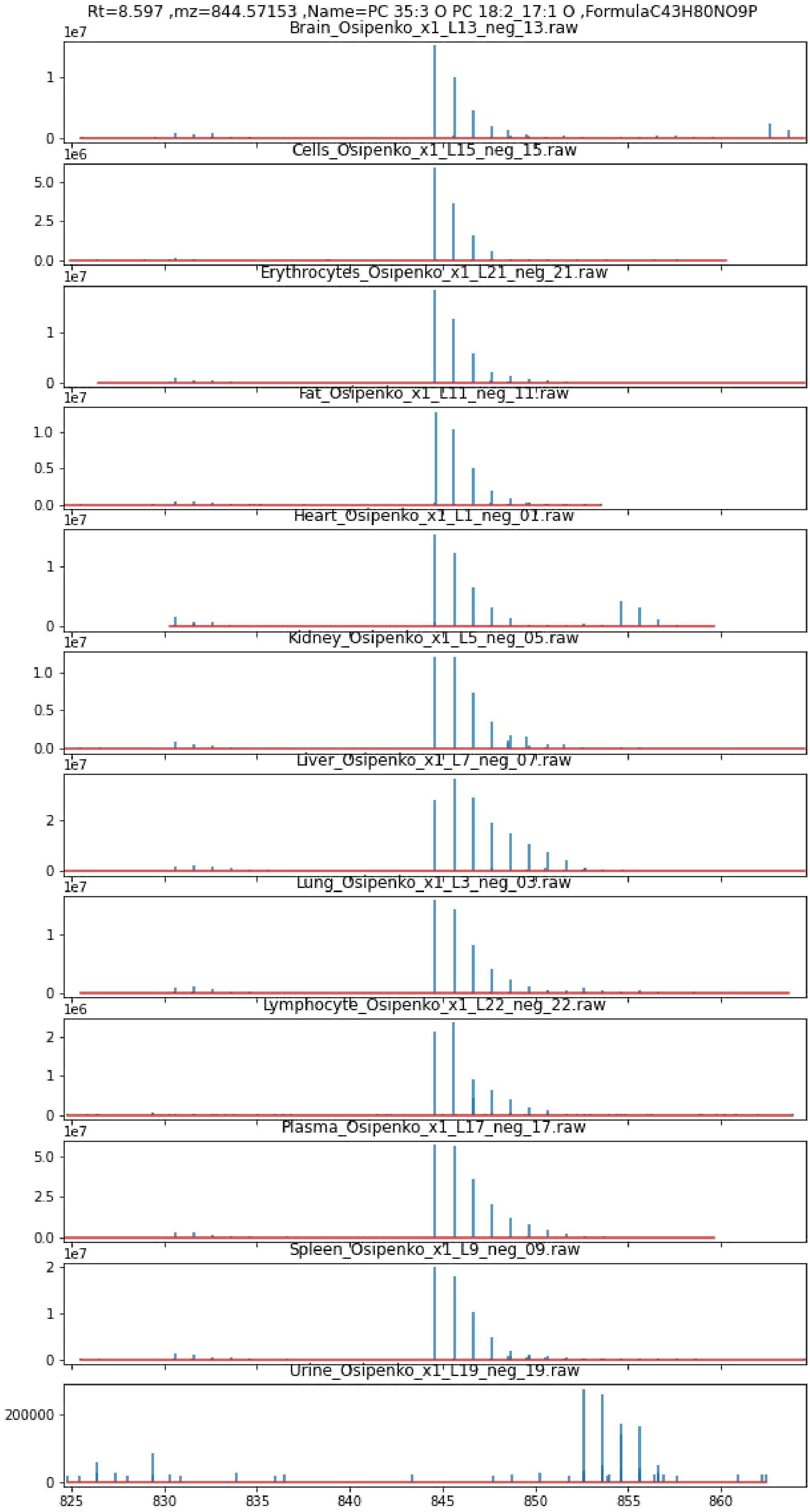

Rt=8.597 ,mz=844.57153 ,Name=PC 35:3 O PC 18:2_17:1 O ,FormulaC43H80NO9P
Brain_Osipenko_x1_L13_neg_13.raw
Cells_Osipenko_x1_L15_neg_15.raw
Erythrocytes_Osipenko_x1_L21_neg_21.raw
Fat_Osipenko_x1_L11_neg_11.raw
Heart_Osipenko_x1_L1_neg_01.raw
Kidney_Osipenko_x1_L5_neg_05.raw
Liver_Osipenko_x1_L7_neg_07.raw
Lung_Osipenko_x1_L3_neg_03.raw
Lymphocyte_Osipenko_x1_L22_neg_22.raw
Plasma_Osipenko_x1_L17_neg_17.raw
Spleen_Osipenko_x1_L9_neg_09.raw
Urine_Osipenko_x1_L19_neg_19.raw
825
830
835
840
845
850
855
860

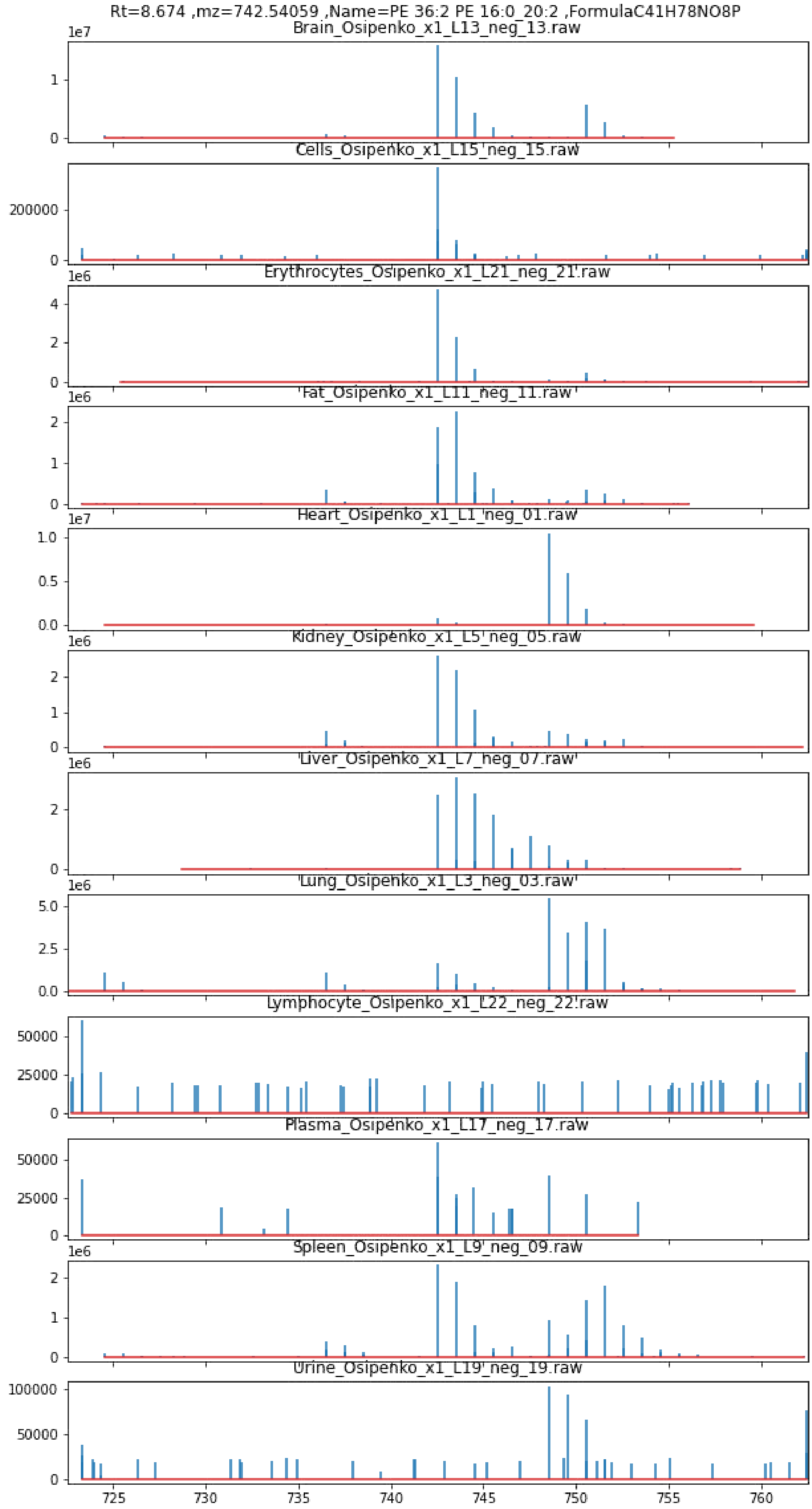

Rt=8.674 ,mz=742.54059 ,Name=PE 36:2 PE 16:0_20:2 ,FormulaC41H78NO8P
Brain_Osipenko_x1_L13_neg_13.raw
Cells_Osipenko_x1_L15_neg_15.raw
Erythrocytes_Osipenko_x1_L21_neg_21.raw
'Fat_Osipenko_x1_L11_neg_11.raw'
Heart_Osipenko_x1_L1_neg_01.raw
Kidney_Osipenko_x1_L5_neg_05.raw
'Liver_Osipenko_x1_L7_neg_07.raw'
'Lung_Osipenko_x1_L3_neg_03.raw'
Lymphocyte_Osipenko_x1_L22_neg_22.raw
Plasma_Osipenko_x1_L17_neg_17.raw
Spleen_Osipenko_x1_L9_neg_09.raw
Urine_Osipenko_x1_L19_neg_19.raw
725
730
735
740
745
750
755
760

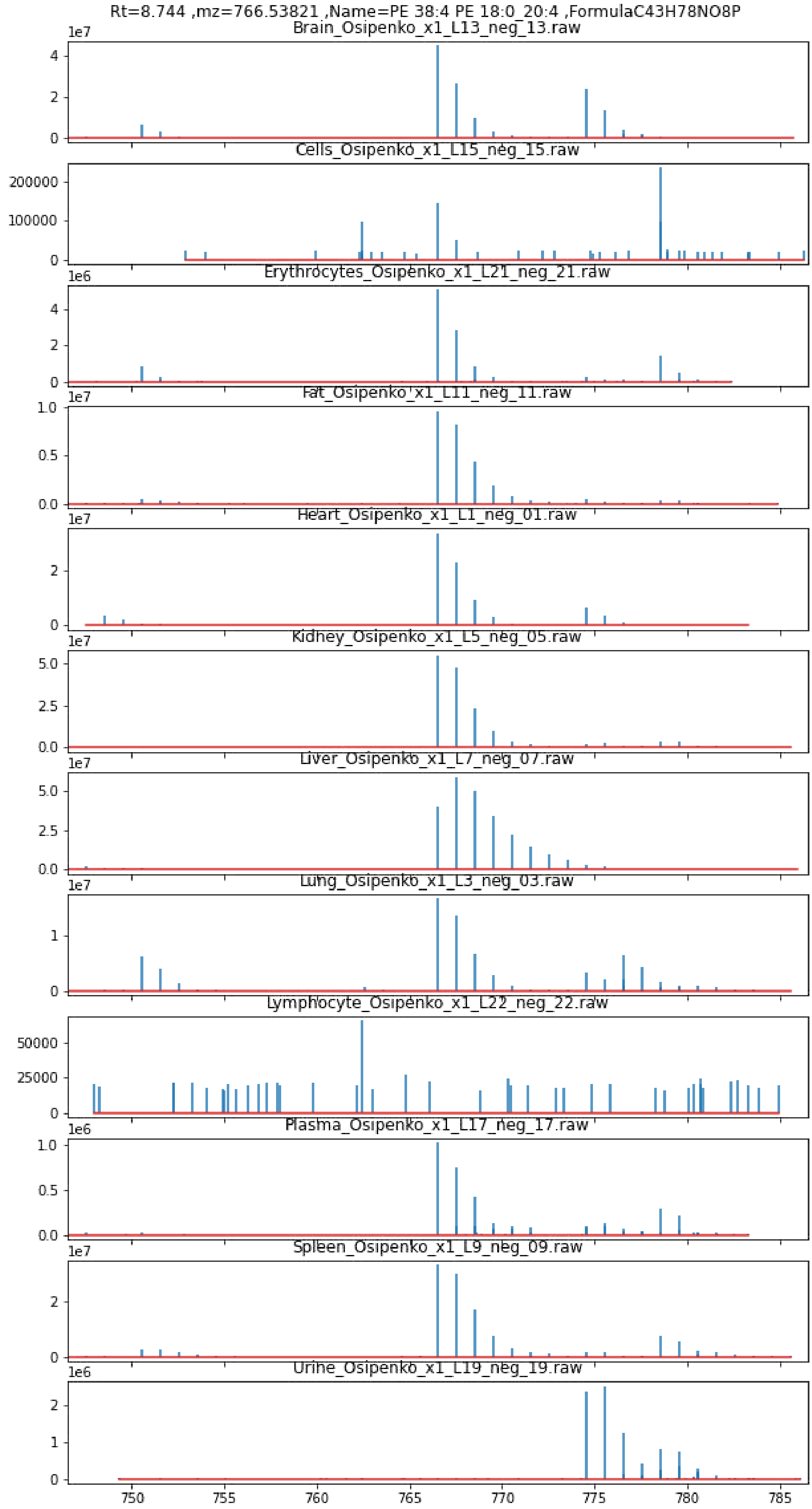
Rt=8.744 ,mz=766.53821 ,Name=PE 38:4 PE 18:0_20:4 ,FormulaC43H78NO8P
Brain_Osipenko_x1_L13_neg_13.raw
Cells_Osipenko_x1_L15_neg_15.raw
Erythrocytes_Osipenko_x1_L21_neg_21.raw
Fat_Osipenko_x1_L11_neg_11.raw
Heart_Osipenko_x1_L1_neg_01.raw
Kidney_Osipenko_x1_L5_neg_05.raw
Liver_Osipenko_x1_L7_neg_07.raw
Lung_Osipenko_x1_L3_neg_03.raw
Lymphocyte_Osipenko_x1_L22_neg_22.raw
Plasma_Osipenko_x1_L17_neg_17.raw
Spleen_Osipenko_x1_L9_neg_09.raw
Urine_Osipenko_x1_L19_neg_19.raw
1e7
1e6
750
755
760
765
770
775
780
785

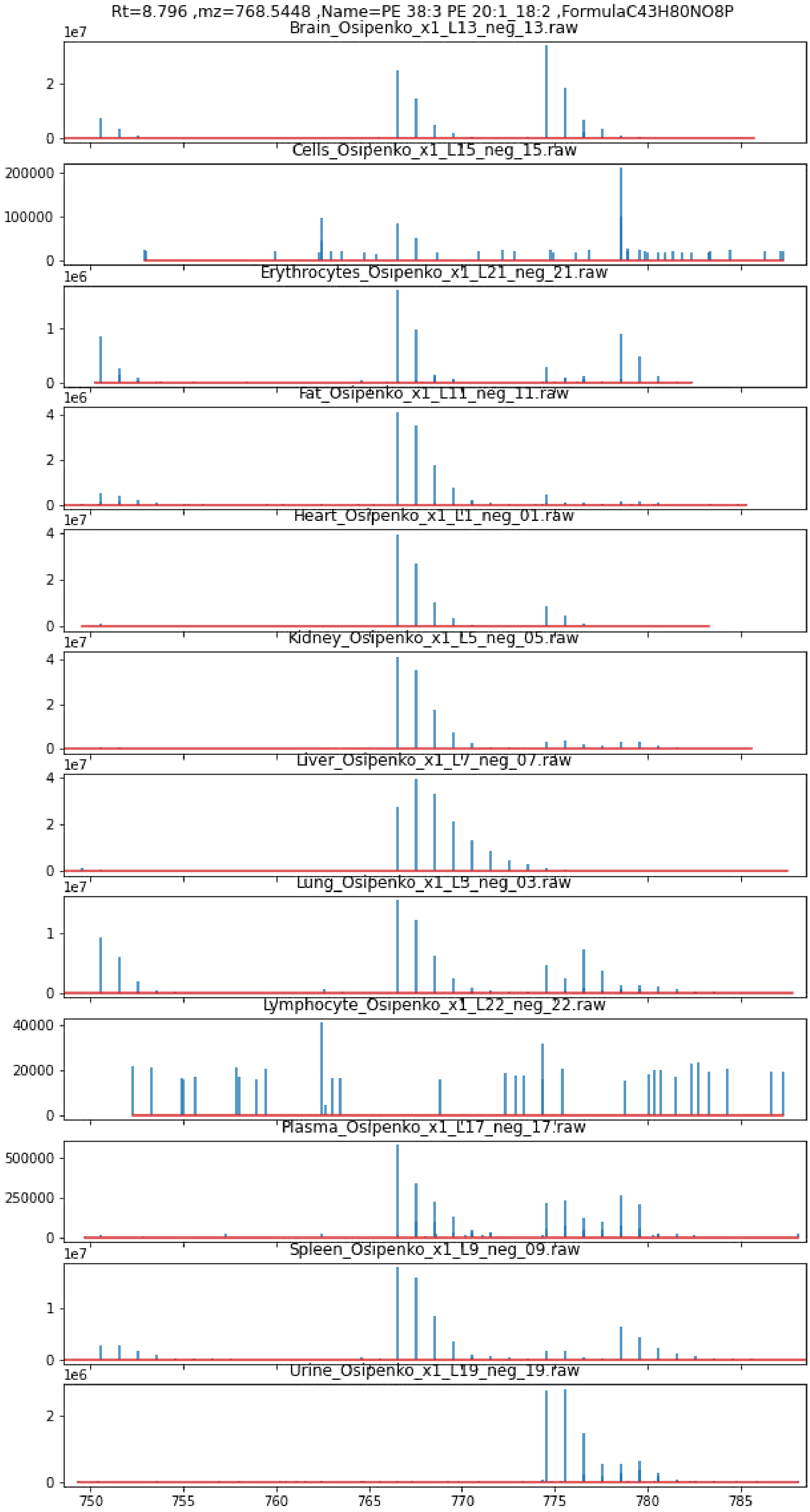

Rt=8.796 ,mz=768.5448 ,Name=PE 38:3 PE 20:1_18:2 ,FormulaC43H80NO8P
Brain_Osipenko_x1_L13_neg_13.raw
Cells_Osipenko_x1_L15_neg_15.raw
Erythrocytes_Osipenko_x1_L21_neg_21.raw
Fat_Osipenko_x1_L11_neg_11.raw
Heart_Osipenko_x1_L1_neg_01.raw
Kidney_Osipenko_x1_L5_neg_05.raw
Liver_Osipenko_x1_L7_neg_07.raw
Lung_Osipenko_x1_L3_neg_03.raw
Lymphocyte_Osipenko_x1_L22_neg_22.raw
Plasma_Osipenko_x1_L17_neg_17.raw
Spleen_Osipenko_x1_L9_neg_09.raw
Urine_Osipenko_x1_L19_neg_19.raw
750
755
760
765
770
775
780
785

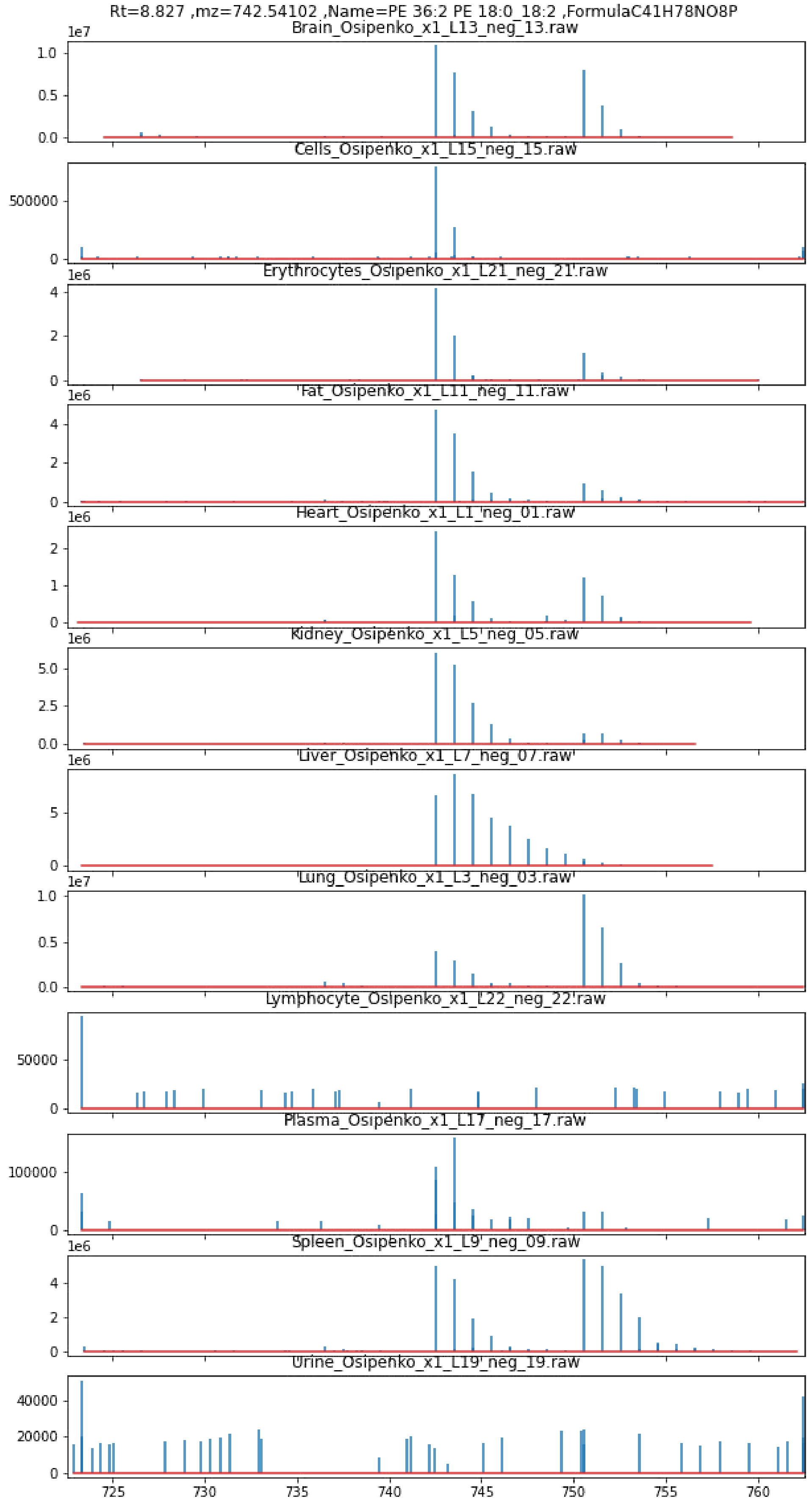

Rt=8.827 ,mz=742.54102 ,Name=PE 36:2 PE 18:0_18:2 ,FormulaC41H78NO8P
Brain_Osipenko_x1_L13_neg_13.raw
Cells_Osipenko_x1_L15_neg_15.raw
Erythrocytes_Osipenko_x1_L21_neg_21.raw
Fat_Osipenko_x1_L11_neg_11.raw
Heart_Osipenko_x1_L1_neg_01.raw
Kidney_Osipenko_x1_L5_neg_05.raw
Liver_Osipenko_x1_L7_neg_07.raw
Lung_Osipenko_x1_L3_neg_03.raw
Lymphocyte_Osipenko_x1_L22_neg_22.raw
Plasma_Osipenko_x1_L17_neg_17.raw
Spleen_Osipenko_x1_L9_neg_09.raw
Urine_Osipenko_x1_L19_neg_19.raw
725
730
735
740
745
750
755
760

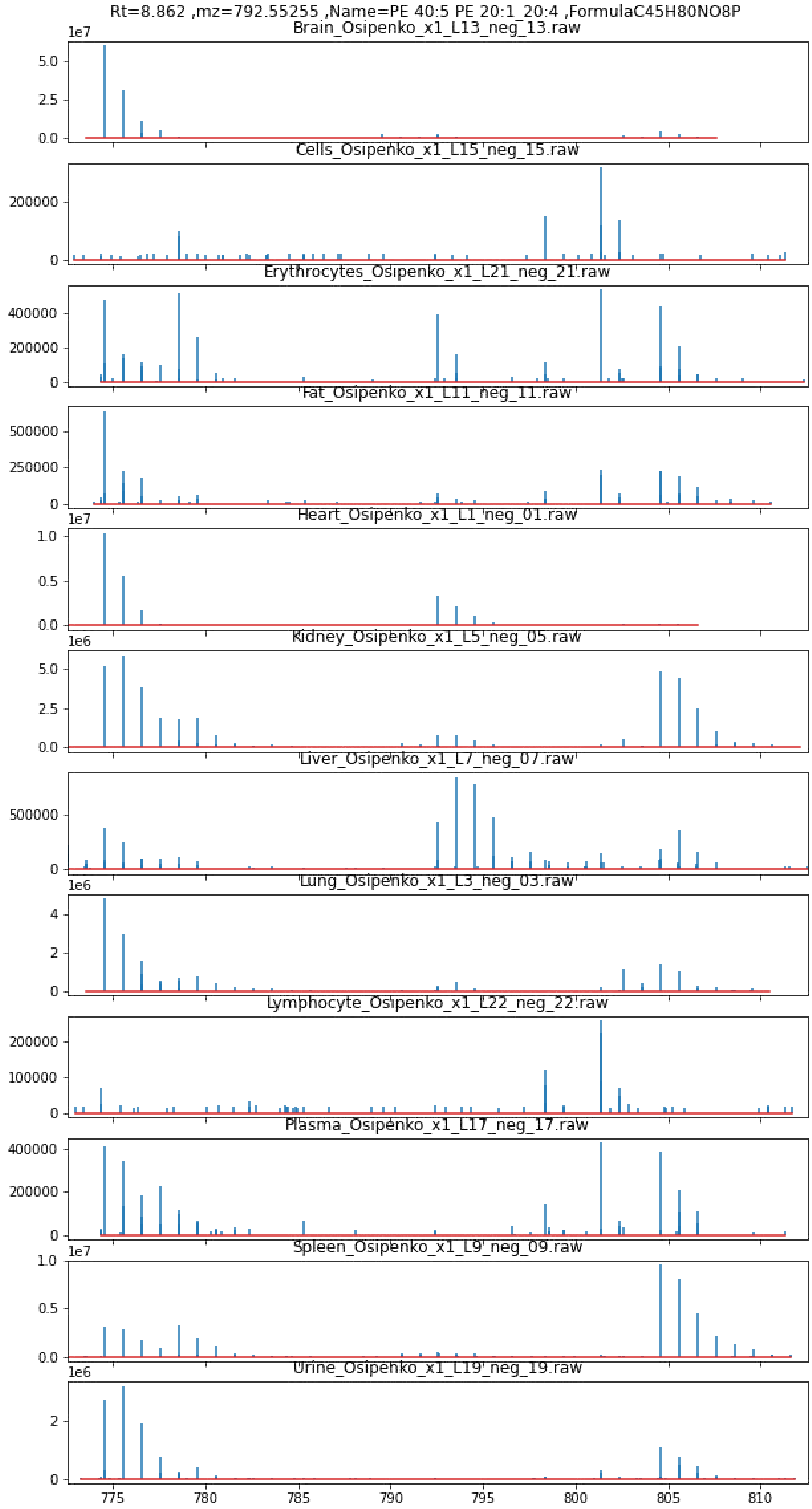

Rt=8.862 ,mz=792.55255 ,Name=PE 40:5 PE 20:1_20:4 ,FormulaC45H80NO8P
Brain_Osipenko_x1_L13_neg_13.raw
Cells_Osipenko_x1_L15_neg_15.raw
Erythrocytes_Osipenko_x1_L21_neg_21.raw
'Fat_Osipenko_x1_L11_neg_11.raw'
Heart_Osipenko_x1_L1_neg_01.raw
Kidney_Osipenko_x1_L5_neg_05.raw
'Liver_Osipenko_x1_L7_neg_07.raw'
'Lung_Osipenko_x1_L3_neg_03.raw'
Lymphocyte_Osipenko_x1_L22_neg_22.raw
Plasma_Osipenko_x1_L17_neg_17.raw
Spleen_Osipenko_x1_L9_neg_09.raw
Urine_Osipenko_x1_L19_neg_19.raw
775
780
785
790
795
800
805
810

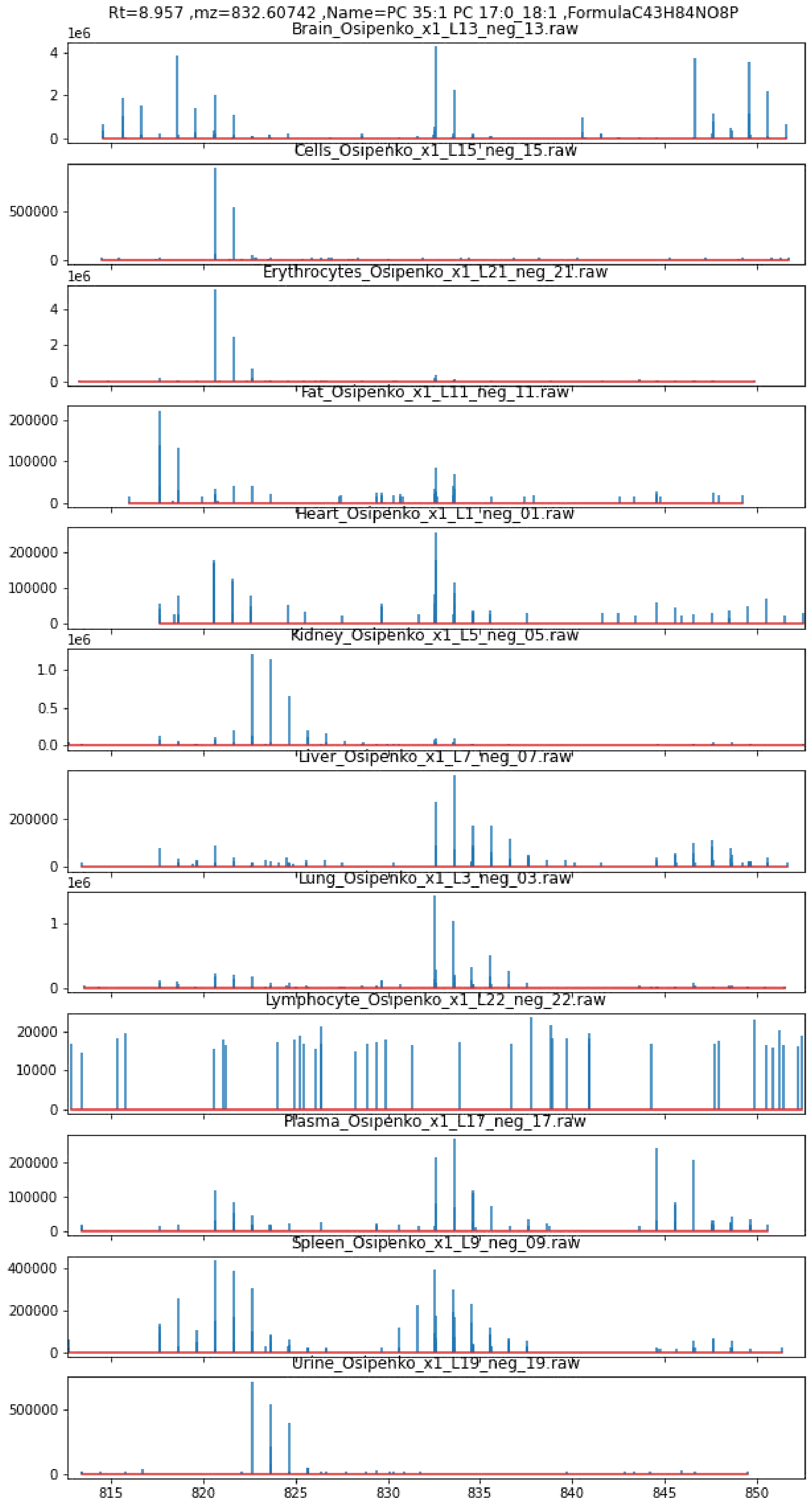

Rt=8.957 ,mz=832.60742 ,Name=PC 35:1 PC 17:0_18:1 ,FormulaC43H84NO8P
Brain_Osipenko_x1_L13_neg_13.raw
Cells_Osipenko_x1_L15_neg_15.raw
Erythrocytes_Osipenko_x1_L21_neg_21.raw
Fat_Osipenko_x1_L11_neg_11.raw
Heart_Osipenko_x1_L1_neg_01.raw
Kidney_Osipenko_x1_L5_neg_05.raw
Liver_Osipenko_x1_L7_neg_07.raw
Lung_Osipenko_x1_L3_neg_03.raw
Lymphocyte_Osipenko_x1_L22_neg_22.raw
Plasma_Osipenko_x1_L17_neg_17.raw
Spleen_Osipenko_x1_L9_neg_09.raw
Urine_Osipenko_x1_L19_neg_19.raw

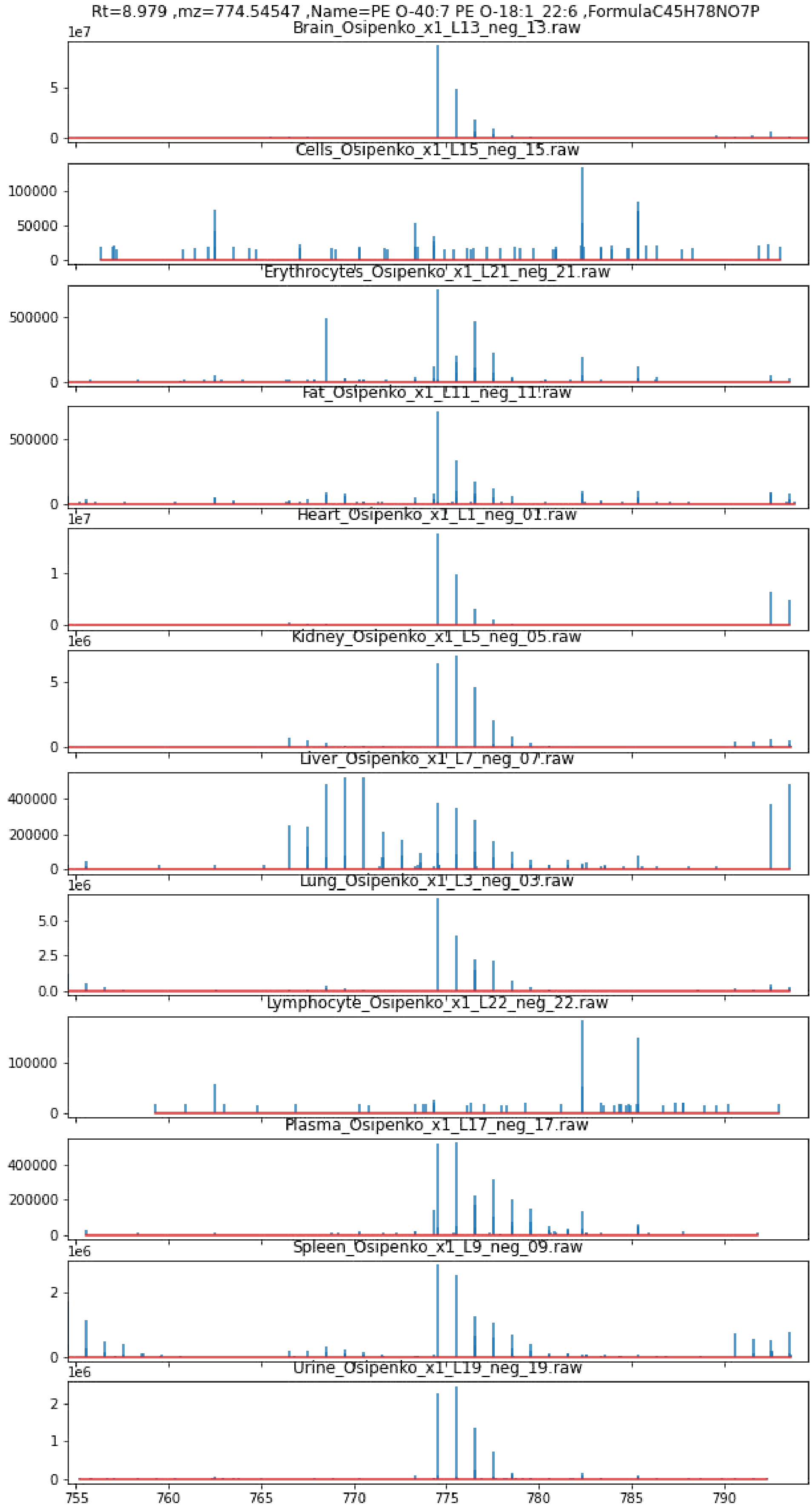

Rt=8.979 ,mz=774.54547 ,Name=PE O-40:7 PE O-18:1_22:6 ,FormulaC45H78NO7P
Brain_Osipenko_x1_L13_neg_13.raw
Cells_Osipenko_x1_L15_neg_15.raw
Erythrocytes_Osipenko_x1_L21_neg_21.raw
Fat_Osipenko_x1_L11_neg_11.raw
Heart_Osipenko_x1_L1_neg_01.raw
Kidney_Osipenko_x1_L5_neg_05.raw
Liver_Osipenko_x1_L7_neg_07.raw
Lung_Osipenko_x1_L3_neg_03.raw
Lymphocyte_Osipenko_x1_L22_neg_22.raw
Plasma_Osipenko_x1_L17_neg_17.raw
Spleen_Osipenko_x1_L9_neg_09.raw
Urine_Osipenko_x1_L19_neg_19.raw
755
760
765
770
775
780
785
790

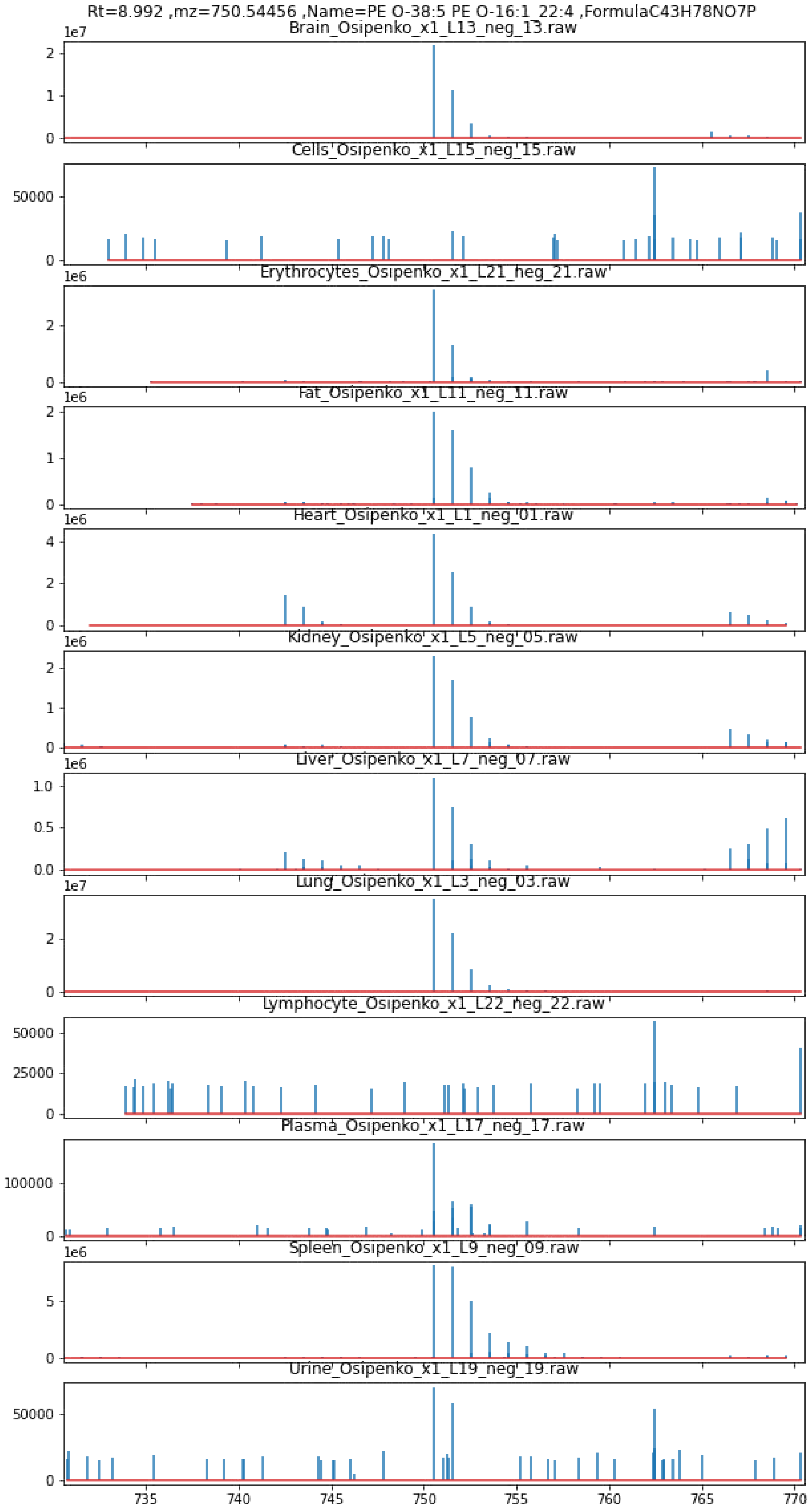
Rt=8.992 ,mz=750.54456 ,Name=PE O-38:5 PE O-16:1_22:4 ,FormulaC43H78NO7P
Brain_Osipenko_x1_L13_neg_13.raw
Cells_Osipenko_x1_L15_neg_15.raw
Erythrocytes_Osipenko_x1_L21_neg_21.raw
Fat_Osipenko_x1_L11_neg_11.raw
Heart_Osipenko_x1_L1_neg_01.raw
Kidney_Osipenko_x1_L5_neg_05.raw
Liver_Osipenko_x1_L7_neg_07.raw
Lung_Osipenko_x1_L3_neg_03.raw
Lymphocyte_Osipenko_x1_L22_neg_22.raw
Plasma_Osipenko_x1_L17_neg_17.raw
Spleen_Osipenko_x1_L9_neg_09.raw
Urine_Osipenko_x1_L19_neg_19.raw
735
740
745
750
755
760
765
770

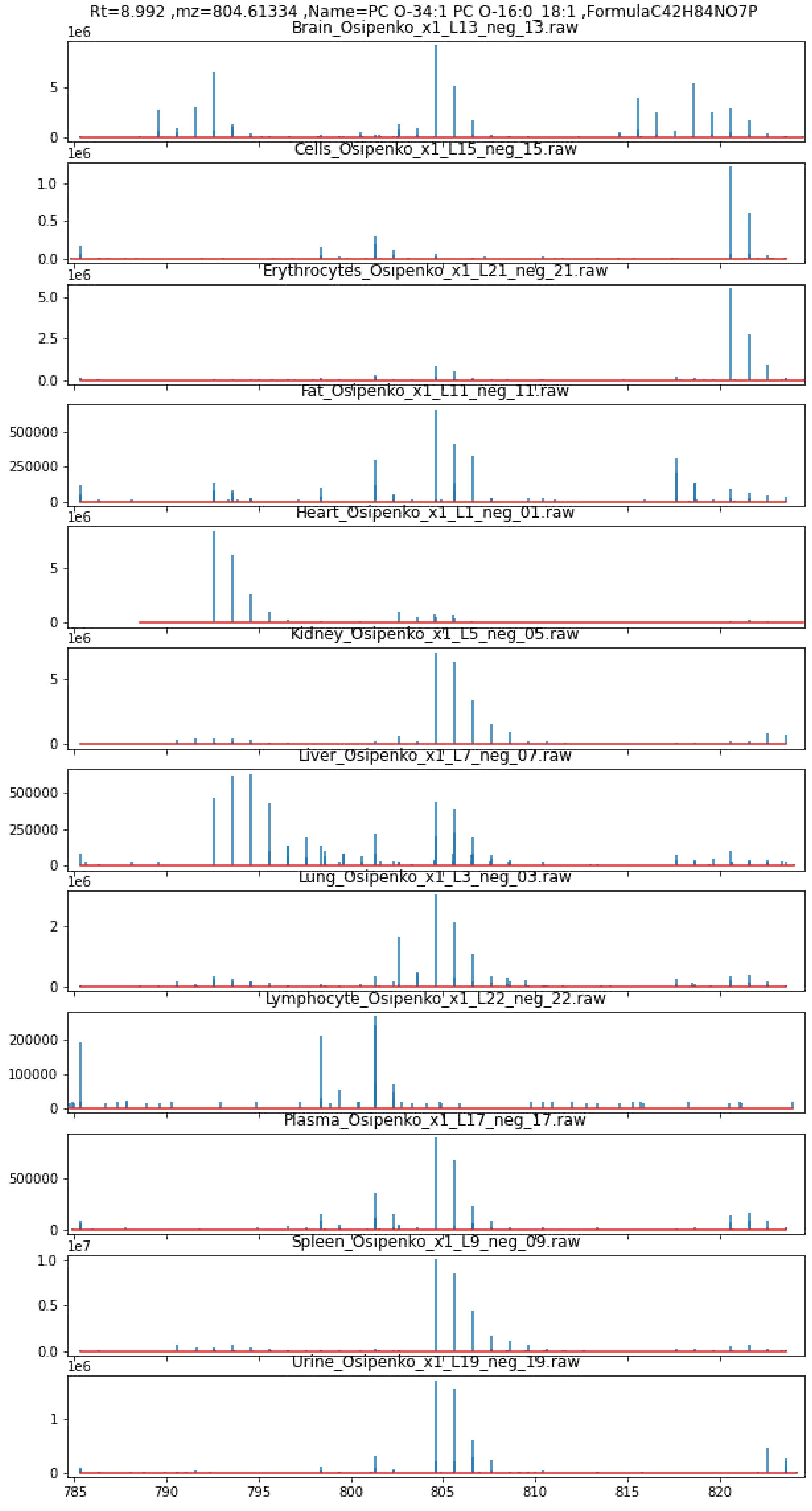

Rt=8.992 ,mz=804.61334 ,Name=PC O-34:1 PC O-16:0_18:1 ,FormulaC42H84NO7P
Brain_Osipenko_x1_L13_neg_13.raw
Cells_Osipenko_x1_L15_neg_15.raw
Erythrocytes_Osipenko_x1_L21_neg_21.raw
Fat_Osipenko_x1_L11_neg_11.raw
Heart_Osipenko_x1_L1_neg_01.raw
Kidney_Osipenko_x1_L5_neg_05.raw
Liver_Osipenko_x1_L7_neg_07.raw
Lung_Osipenko_x1_L3_neg_03.raw
Lymphocyte_Osipenko_x1_L22_neg_22.raw
Plasma_Osipenko_x1_L17_neg_17.raw
Spleen_Osipenko_x1_L9_neg_09.raw
Urine_Osipenko_x1_L19_neg_19.raw

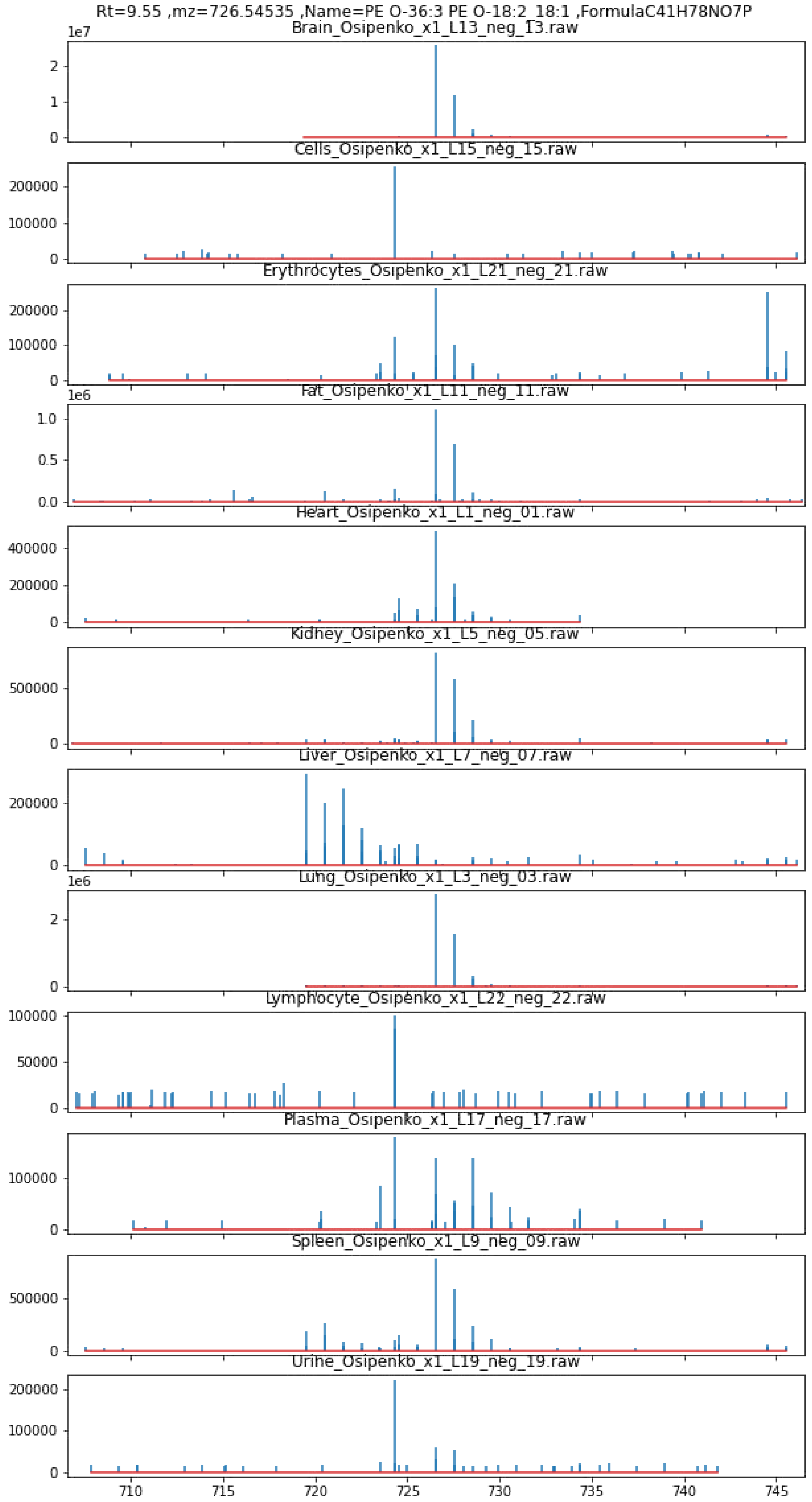
Rt=9.55 ,mz=726.54535 ,Name=PE O-36:3 PE O-18:2_18:1 ,FormulaC41H78NO7P
Brain_Osipenko_x1_L13_neg_13.raw
Cells_Osipenko_x1_L15_neg_15.raw
Erythrocytes_Osipenko_x1_L21_neg_21.raw
Fat_Osipenko_x1_L11_neg_11.raw
Heart_Osipenko_x1_L1_neg_01.raw
Kidney_Osipenko_x1_L5_neg_05.raw
Liver_Osipenko_x1_L7_neg_07.raw
Lung_Osipenko_x1_L3_neg_03.raw
Lymphocyte_Osipenko_x1_L22_neg_22.raw
Plasma_Osipenko_x1_L17_neg_17.raw
Spleen_Osipenko_x1_L9_neg_09.raw
Urine_Osipenko_x1_L19_neg_19.raw
1e7
1e6
1e6
710
715
720
725
730
735
740
745

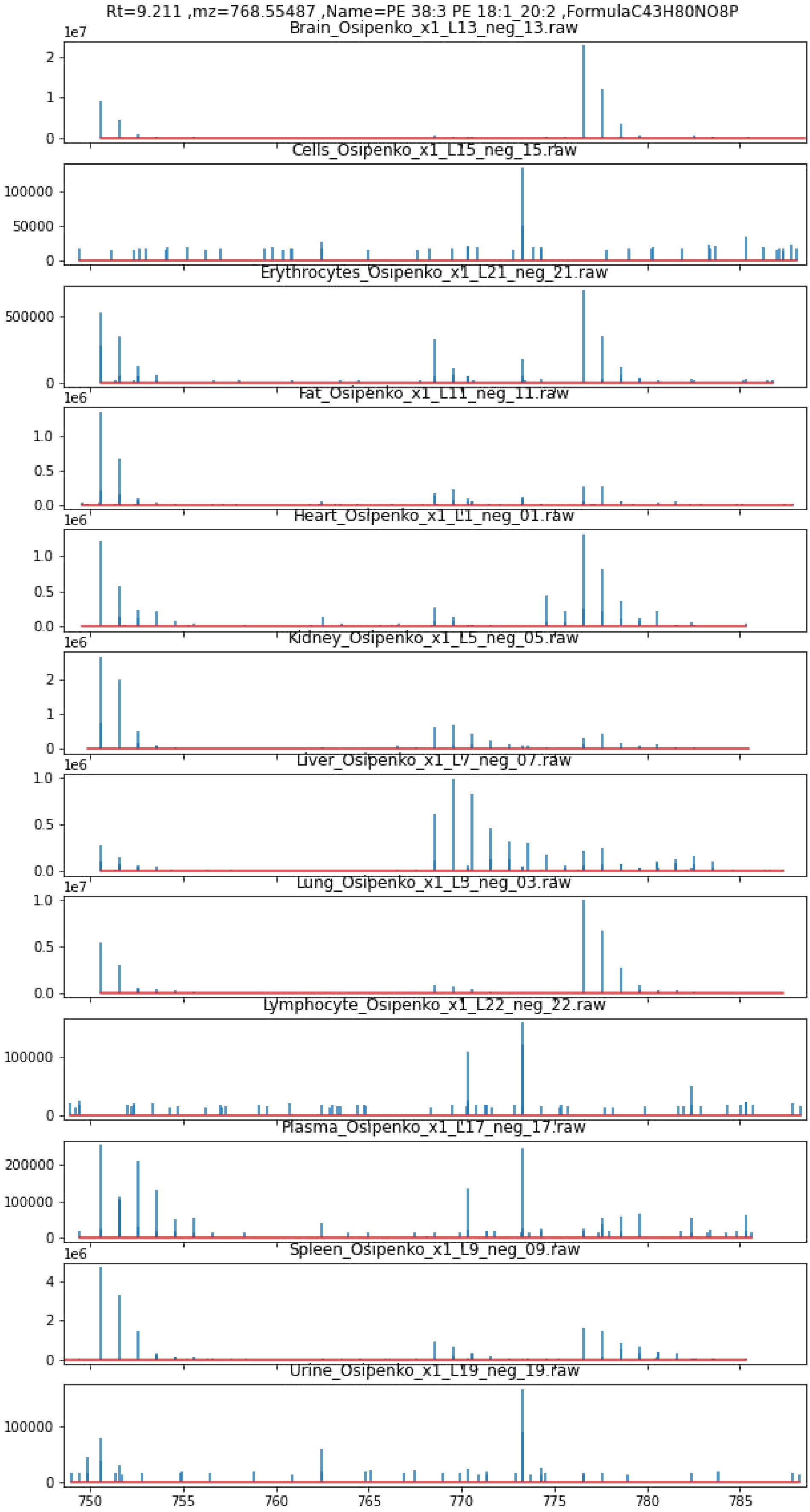

Rt=9.211 ,mz=768.55487 ,Name=PE 38:3 PE 18:1_20:2 ,FormulaC43H80NO8P
Brain_Osipenko_x1_L13_neg_13.raw
Cells_Osipenko_x1_L15_neg_15.raw
Erythrocytes_Osipenko_x1_L21_neg_21.raw
Fat_Osipenko_x1_L11_neg_11.raw
Heart_Osipenko_x1_L1_neg_01.raw
Kidney_Osipenko_x1_L5_neg_05.raw
Liver_Osipenko_x1_L7_neg_07.raw
Lung_Osipenko_x1_L3_neg_03.raw
Lymphocyte_Osipenko_x1_L22_neg_22.raw
Plasma_Osipenko_x1_L17_neg_17.raw
Spleen_Osipenko_x1_L9_neg_09.raw
Urine_Osipenko_x1_L19_neg_19.raw
750
755
760
765
770
775
780
785

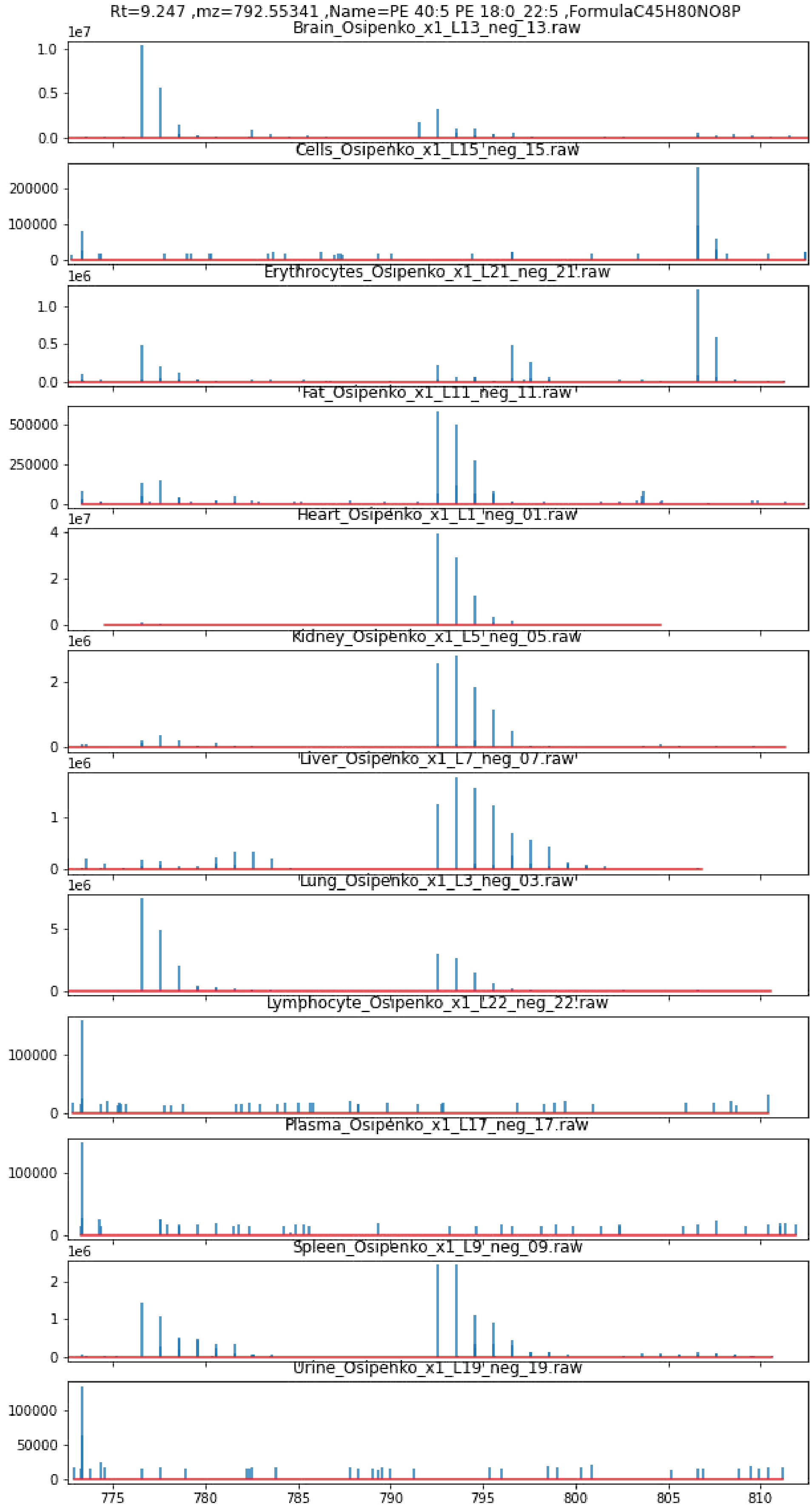
Rt=9.247 ,mz=792.55341 ,Name=PE 40:5 PE 18:0_22:5 ,FormulaC45H80NO8P
Brain_Osipenko_x1_L13_neg_13.raw
1e7
Cells_Osipenko_x1_L15_neg_15.raw
Erythrocytes_Osipenko_x1_L21_neg_21.raw
1e6
Fat_Osipenko_x1_L11_neg_11.raw
Heart_Osipenko_x1_L1_neg_01.raw
1e7
Kidney_Osipenko_x1_L5_neg_05.raw
1e6
Liver_Osipenko_x1_L7_neg_07.raw
1e6
Lung_Osipenko_x1_L3_neg_03.raw
1e6
Lymphocyte_Osipenko_x1_L22_neg_22.raw
Plasma_Osipenko_x1_L17_neg_17.raw
Spleen_Osipenko_x1_L9_neg_09.raw
1e6
Urine_Osipenko_x1_L19_neg_19.raw
775
780
785
790
795
800
805
810

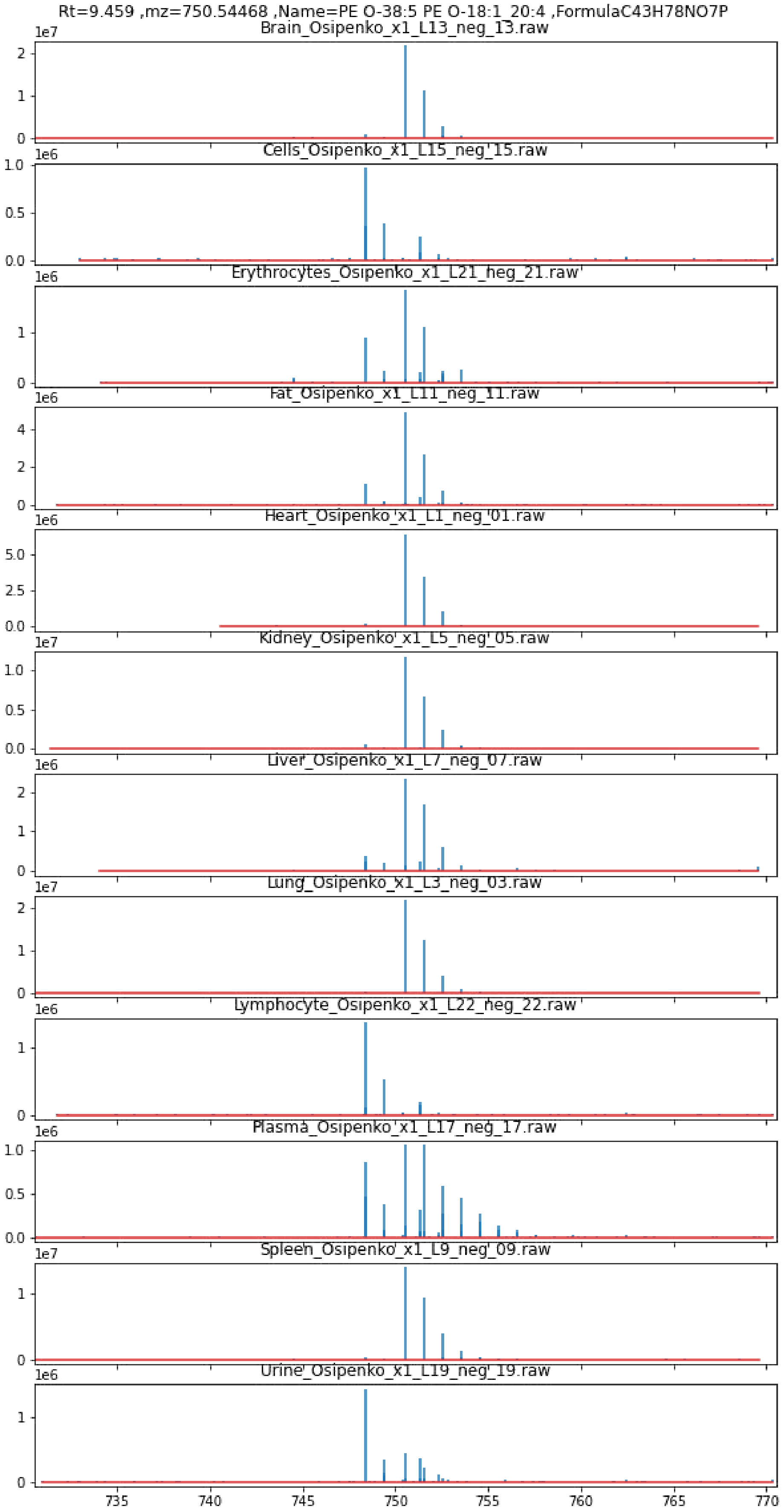
Rt=9.459 ,mz=750.54468 ,Name=PE O-38:5 PE O-18:1_20:4 ,FormulaC43H78NO7P
Brain_Osipenko_x1_L13_neg_13.raw
Cells_Osipenko_x1_L15_neg_15.raw
Erythrocytes_Osipenko_x1_L21_neg_21.raw
Fat_Osipenko_x1_L11_neg_11.raw
Heart_Osipenko_x1_L1_neg_01.raw
Kidney_Osipenko_x1_L5_neg_05.raw
Liver_Osipenko_x1_L7_neg_07.raw
Lung_Osipenko_x1_L3_neg_03.raw
Lymphocyte_Osipenko_x1_L22_neg_22.raw
Plasma_Osipenko_x1_L17_neg_17.raw
Spleen_Osipenko_x1_L9_neg_09.raw
Urine_Osipenko_x1_L19_neg_19.raw
735
740
745
750
755
760
765
770

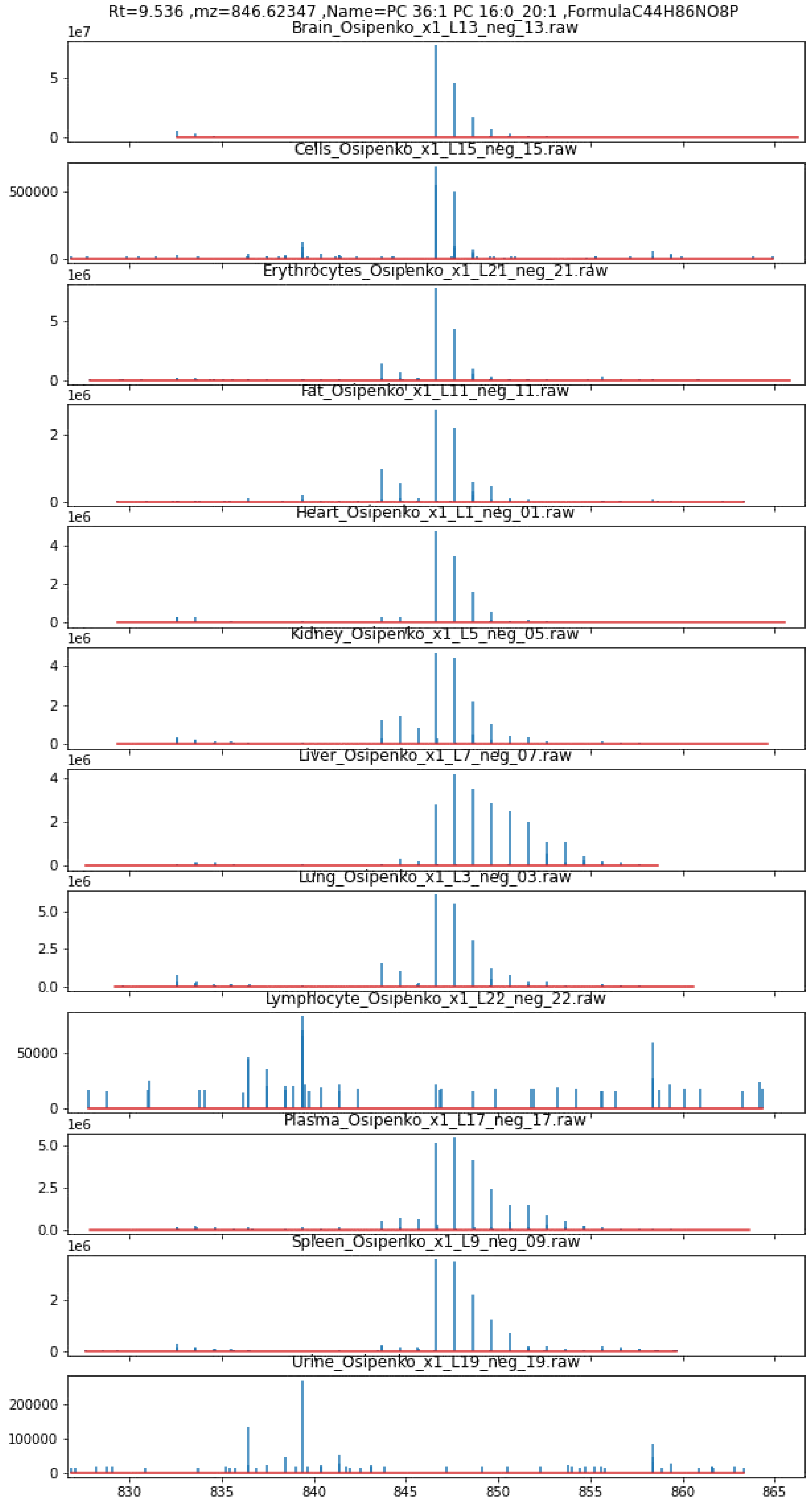

Rt=9.536 ,mz=846.62347 ,Name=PC 36:1 PC 16:0_20:1 ,FormulaC44H86NO8P
Brain_Osipenko_x1_L13_neg_13.raw
Cells_Osipenko_x1_L15_neg_15.raw
Erythrocytes_Osipenko_x1_L21_neg_21.raw
Fat_Osipenko_x1_L11_neg_11.raw
Heart_Osipenko_x1_L1_neg_01.raw
Kidney_Osipenko_x1_L5_neg_05.raw
Liver_Osipenko_x1_L7_neg_07.raw
Lung_Osipenko_x1_L3_neg_03.raw
Lymphocyte_Osipenko_x1_L22_neg_22.raw
Plasma_Osipenko_x1_L17_neg_17.raw
Spleen_Osipenko_x1_L9_neg_09.raw
Urine_Osipenko_x1_L19_neg_19.raw
830
835
840
845
850
855
860
865

Rt=9.583 ,mz=869.67627 ,Name=SM 42:3 O2 SM 18:1 O2 24:2 ,FormulaC47H91N2O6P

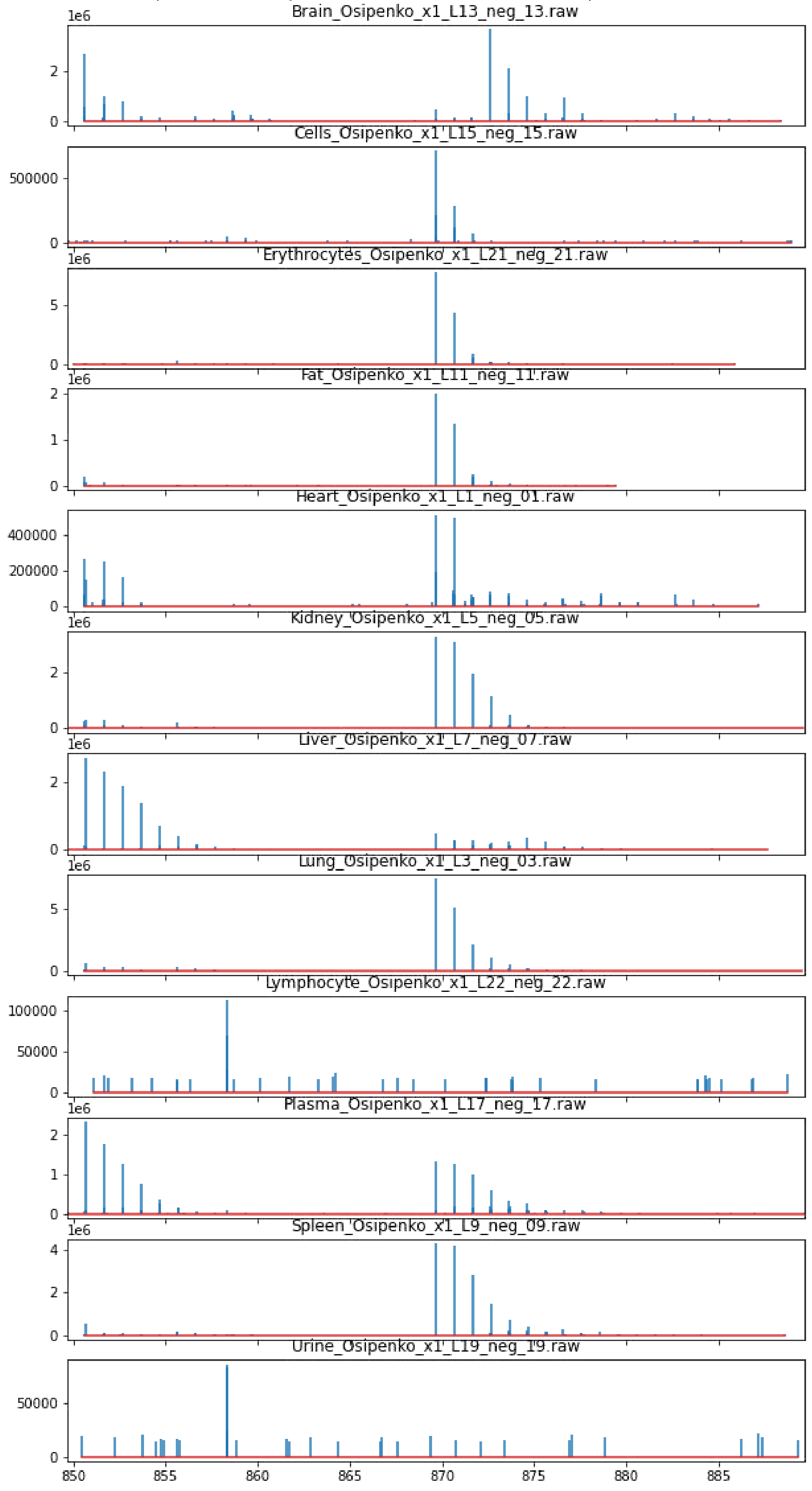

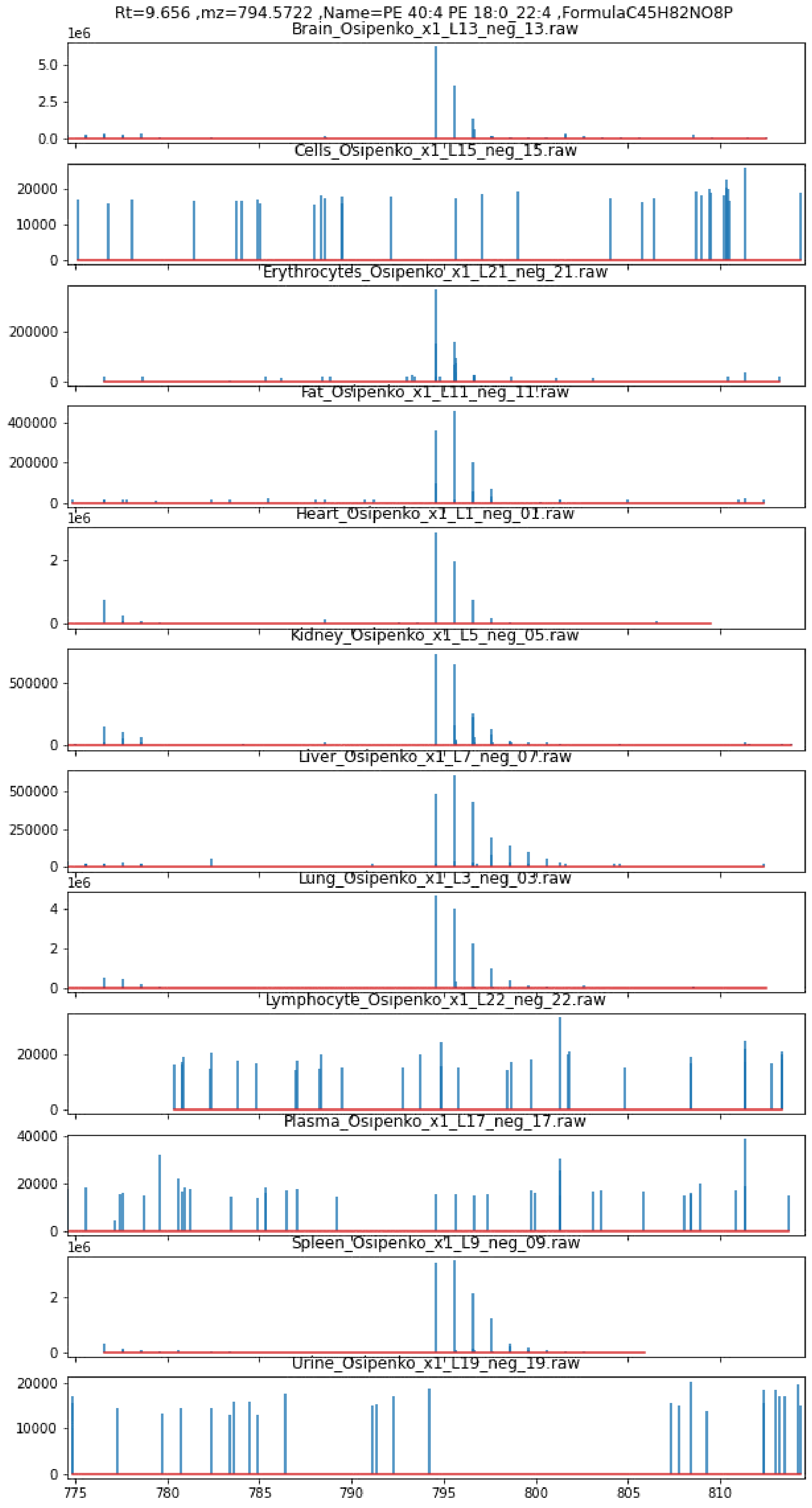

Rt=9.656 ,mz=794.5722 ,Name=PE 40:4 PE 18:0_22:4 ,FormulaC45H82NO8P
Brain_Osipenko_x1_L13_neg_13.raw
Cells_Osipenko_x1_L15_neg_15.raw
Erythrocytes_Osipenko_x1_L21_neg_21.raw
Fat_Osipenko_x1_L11_neg_11.raw
Heart_Osipenko_x1_L1_neg_01.raw
Kidney_Osipenko_x1_L5_neg_05.raw
Liver_Osipenko_x1_L7_neg_07.raw
Lung_Osipenko_x1_L3_neg_03.raw
Lymphocyte_Osipenko_x1_L22_neg_22.raw
Plasma_Osipenko_x1_L17_neg_17.raw
Spleen_Osipenko_x1_L9_neg_09.raw
Urine_Osipenko_x1_L19_neg_19.raw

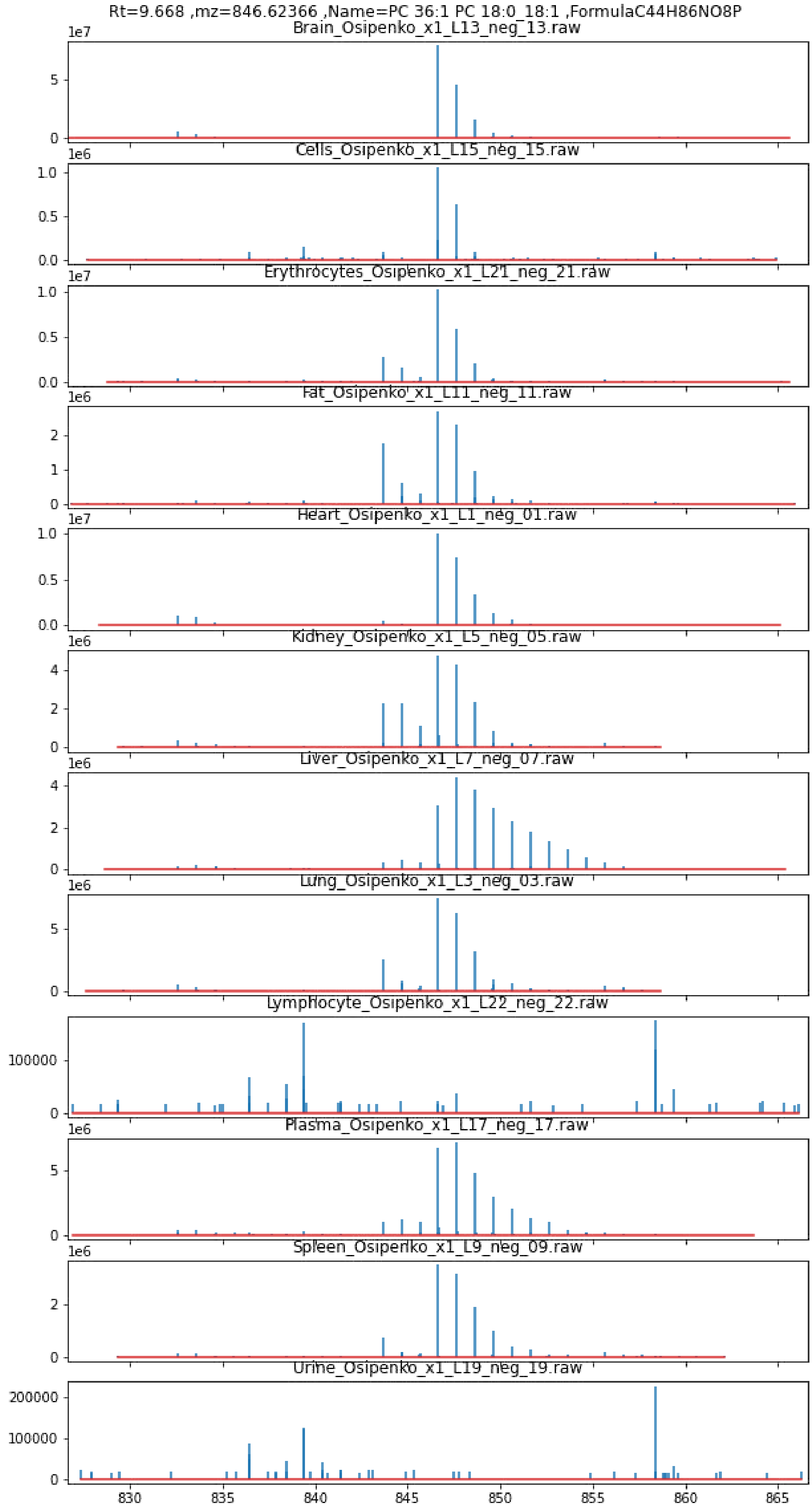

Rt=9.668 ,mz=846.62366 ,Name=PC 36:1 PC 18:0_18:1 ,FormulaC44H86NO8P
Brain_Osipenko_x1_L13_neg_13.raw
Cells_Osipenko_x1_L15_neg_15.raw
Erythrocytes_Osipenko_x1_L21_neg_21.raw
Fat_Osipenko_x1_L11_neg_11.raw
Heart_Osipenko_x1_L1_neg_01.raw
Kidney_Osipenko_x1_L5_neg_05.raw
Liver_Osipenko_x1_L7_neg_07.raw
Lung_Osipenko_x1_L3_neg_03.raw
Lymphocyte_Osipenko_x1_L22_neg_22.raw
Plasma_Osipenko_x1_L17_neg_17.raw
Spleen_Osipenko_x1_L9_neg_09.raw
Urine_Osipenko_x1_L19_neg_19.raw
830
835
840
845
850
855
860
865

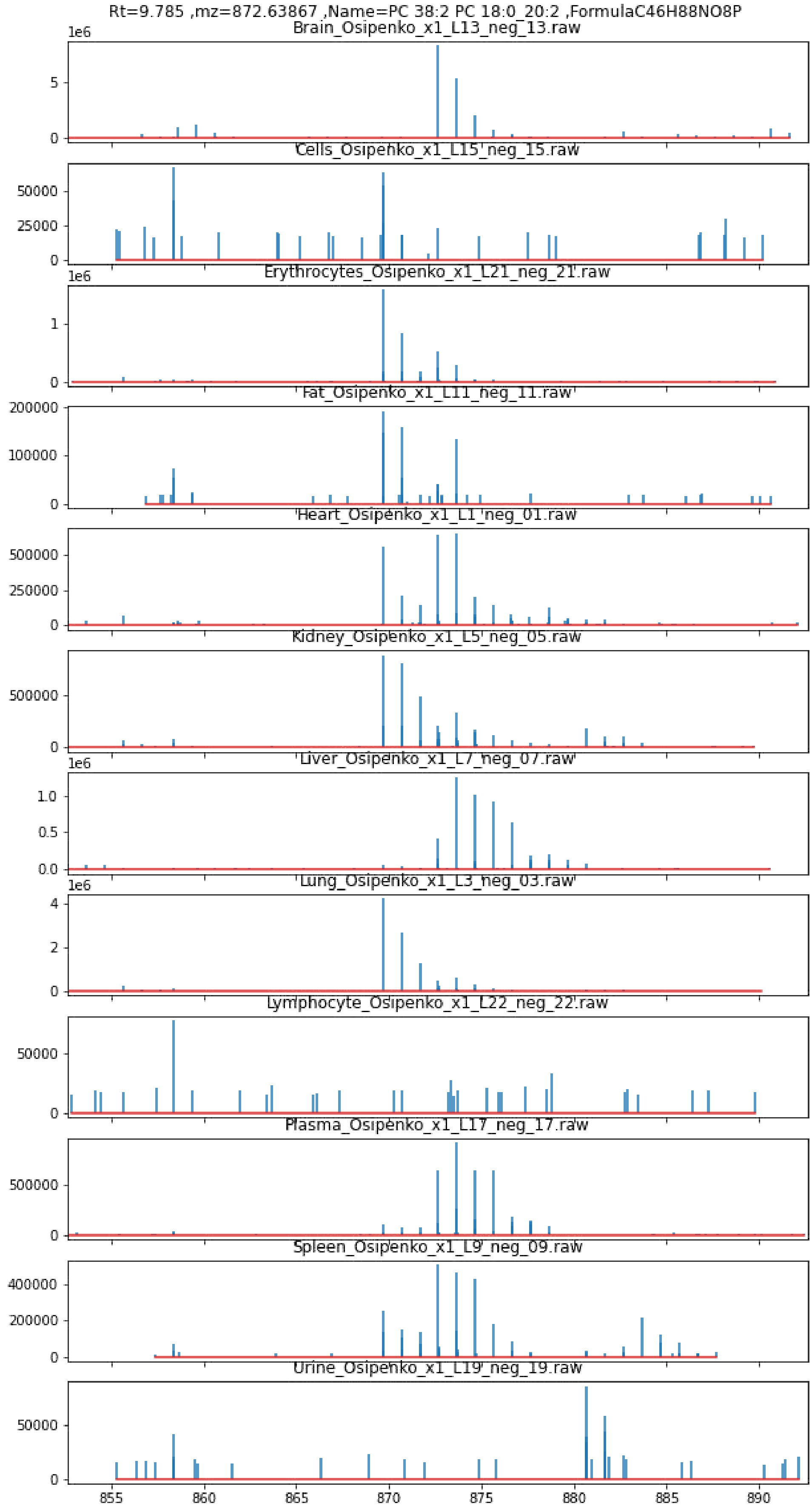

Rt=9.785 ,mz=872.63867 ,Name=PC 38:2 PC 18:0_20:2 ,FormulaC46H88NO8P
Brain_Osipenko_x1_L13_neg_13.raw
Cells_Osipenko_x1_L15_neg_15.raw
Erythrocytes_Osipenko_x1_L21_neg_21.raw
Fat_Osipenko_x1_L11_neg_11.raw
Heart_Osipenko_x1_L1_neg_01.raw
Kidney_Osipenko_x1_L5_neg_05.raw
Liver_Osipenko_x1_L7_neg_07.raw
Lung_Osipenko_x1_L3_neg_03.raw
Lymphocyte_Osipenko_x1_L22_neg_22.raw
Plasma_Osipenko_x1_L17_neg_17.raw
Spleen_Osipenko_x1_L9_neg_09.raw
Urine_Osipenko_x1_L19_neg_19.raw

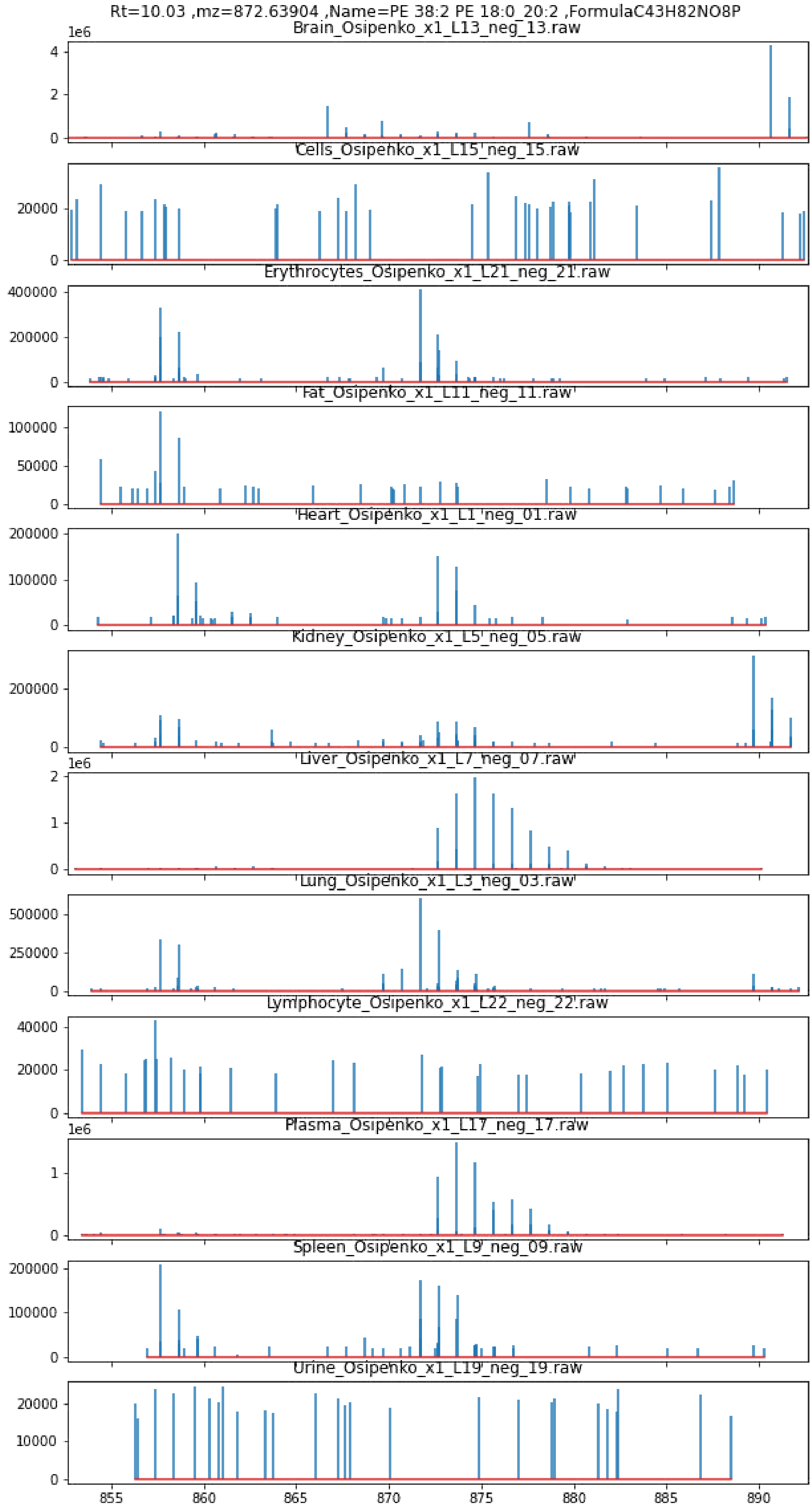

Rt=10.03 ,mz=872.63904 ,Name=PE 38:2 PE 18:0_20:2 ,FormulaC43H82NO8P
Brain_Osipenko_x1_L13_neg_13.raw
1e6
Cells_Osipenko_x1_L15_neg_15.raw
Erythrocytes_Osipenko_x1_L21_neg_21.raw
Fat_Osipenko_x1_L11_neg_11.raw
Heart_Osipenko_x1_L1_neg_01.raw
Kidney_Osipenko_x1_L5_neg_05.raw
Liver_Osipenko_x1_L7_neg_07.raw
1e6
Lung_Osipenko_x1_L3_neg_03.raw
Lymphocyte_Osipenko_x1_L22_neg_22.raw
Plasma_Osipenko_x1_L17_neg_17.raw
1e6
Spleen_Osipenko_x1_L9_neg_09.raw
Urine_Osipenko_x1_L19_neg_19.raw
855
860
865
870
875
880
885
890

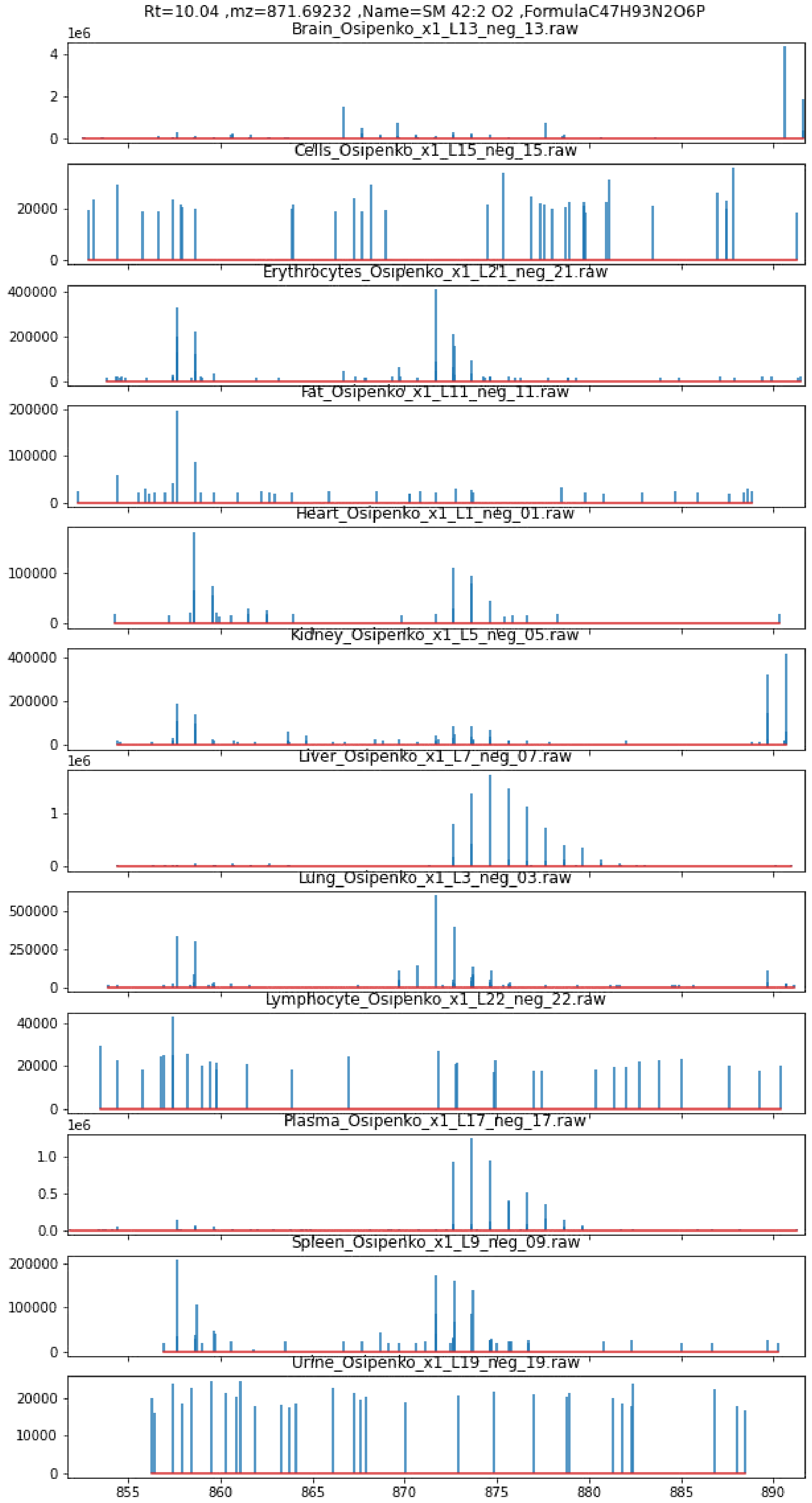

Rt=10.04 ,mz=871.69232 ,Name=SM 42:2 O2 ,FormulaC47H93N2O6P
Brain_Osipenko_x1_L13_neg_13.raw
Cells_Osipenko_x1_L15_neg_15.raw
Erythrocytes_Osipenko_x1_L21_neg_21.raw
Fat_Osipenko_x1_L11_neg_11.raw
Heart_Osipenko_x1_L1_neg_01.raw
Kidney_Osipenko_x1_L5_neg_05.raw
Liver_Osipenko_x1_L7_neg_07.raw
Lung_Osipenko_x1_L3_neg_03.raw
Lymphocyte_Osipenko_x1_L22_neg_22.raw
Plasma_Osipenko_x1_L17_neg_17.raw
Spleen_Osipenko_x1_L9_neg_09.raw
Urine_Osipenko_x1_L19_neg_19.raw
1e6
855
860
865
870
875
880
885
890

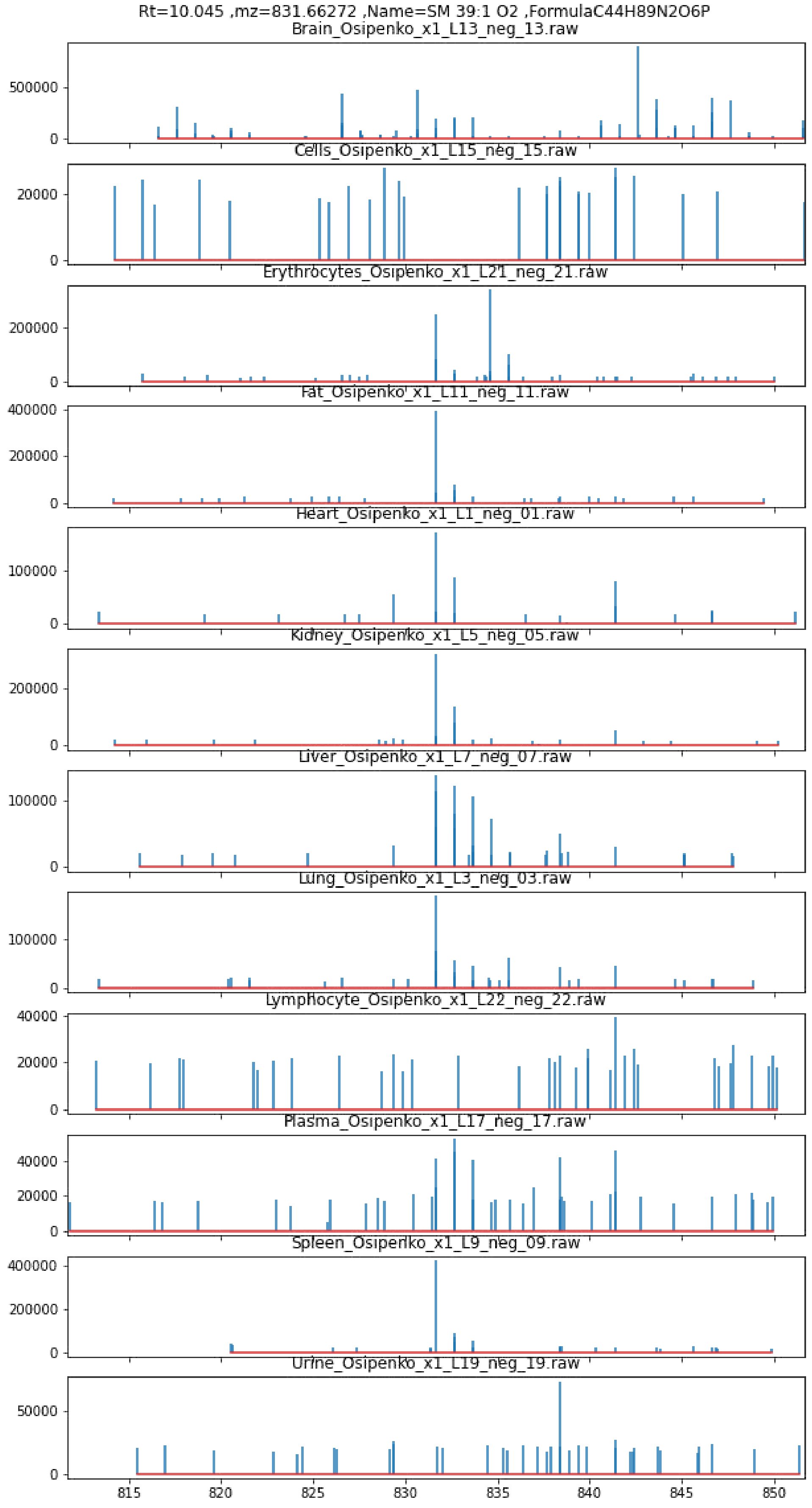
Rt=10.045 ,mz=831.66272 ,Name=SM 39:1 O2 ,FormulaC44H89N2O6P
Brain_Osipenko_x1_L13_neg_13.raw
Cells_Osipenko_x1_L15_neg_15.raw
Erythrocytes_Osipenko_x1_L21_neg_21.raw
Fat_Osipenko_x1_L11_neg_11.raw
Heart_Osipenko_x1_L1_neg_01.raw
Kidney_Osipenko_x1_L5_neg_05.raw
Liver_Osipenko_x1_L7_neg_07.raw
Lung_Osipenko_x1_L3_neg_03.raw
Lymphocyte_Osipenko_x1_L22_neg_22.raw
Plasma_Osipenko_x1_L17_neg_17.raw
Spleen_Osipenko_x1_L9_neg_09.raw
Urine_Osipenko_x1_L19_neg_19.raw
815
820
825
830
835
840
845
850

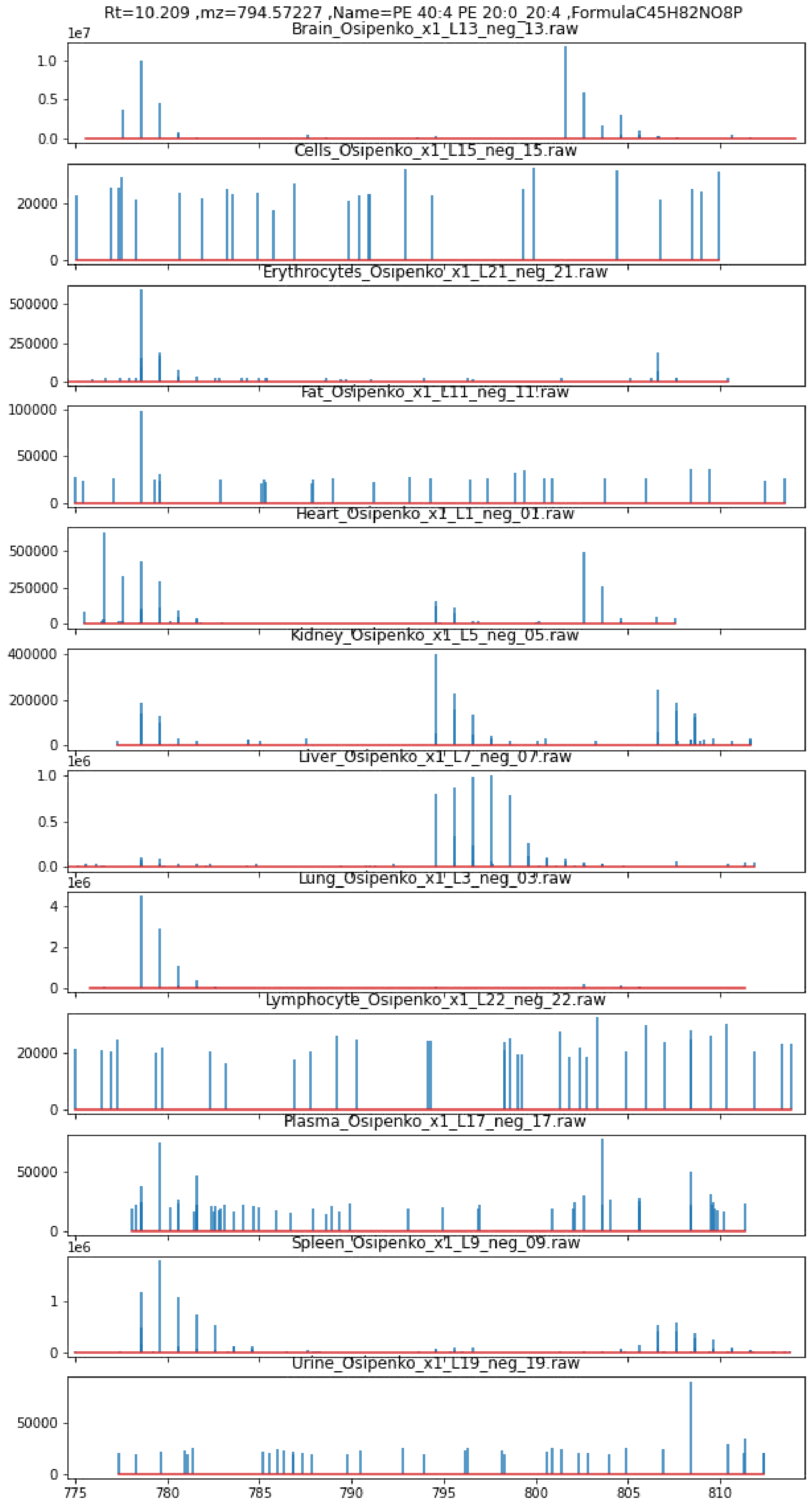

Rt=10.209 ,mz=794.57227 ,Name=PE 40:4 PE 20:0_20:4 ,FormulaC45H82NO8P
Brain_Osipenko_x1_L13_neg_13.raw
Cells_Osipenko_x1_L15_neg_15.raw
Erythrocytes_Osipenko_x1_L21_neg_21.raw
Fat_Osipenko_x1_L11_neg_11.raw
Heart_Osipenko_x1_L1_neg_01.raw
Kidney_Osipenko_x1_L5_neg_05.raw
Liver_Osipenko_x1_L7_neg_07.raw
Lung_Osipenko_x1_L3_neg_03.raw
Lymphocyte_Osipenko_x1_L22_neg_22.raw
Plasma_Osipenko_x1_L17_neg_17.raw
Spleen_Osipenko_x1_L9_neg_09.raw
Urine_Osipenko_x1_L19_neg_19.raw

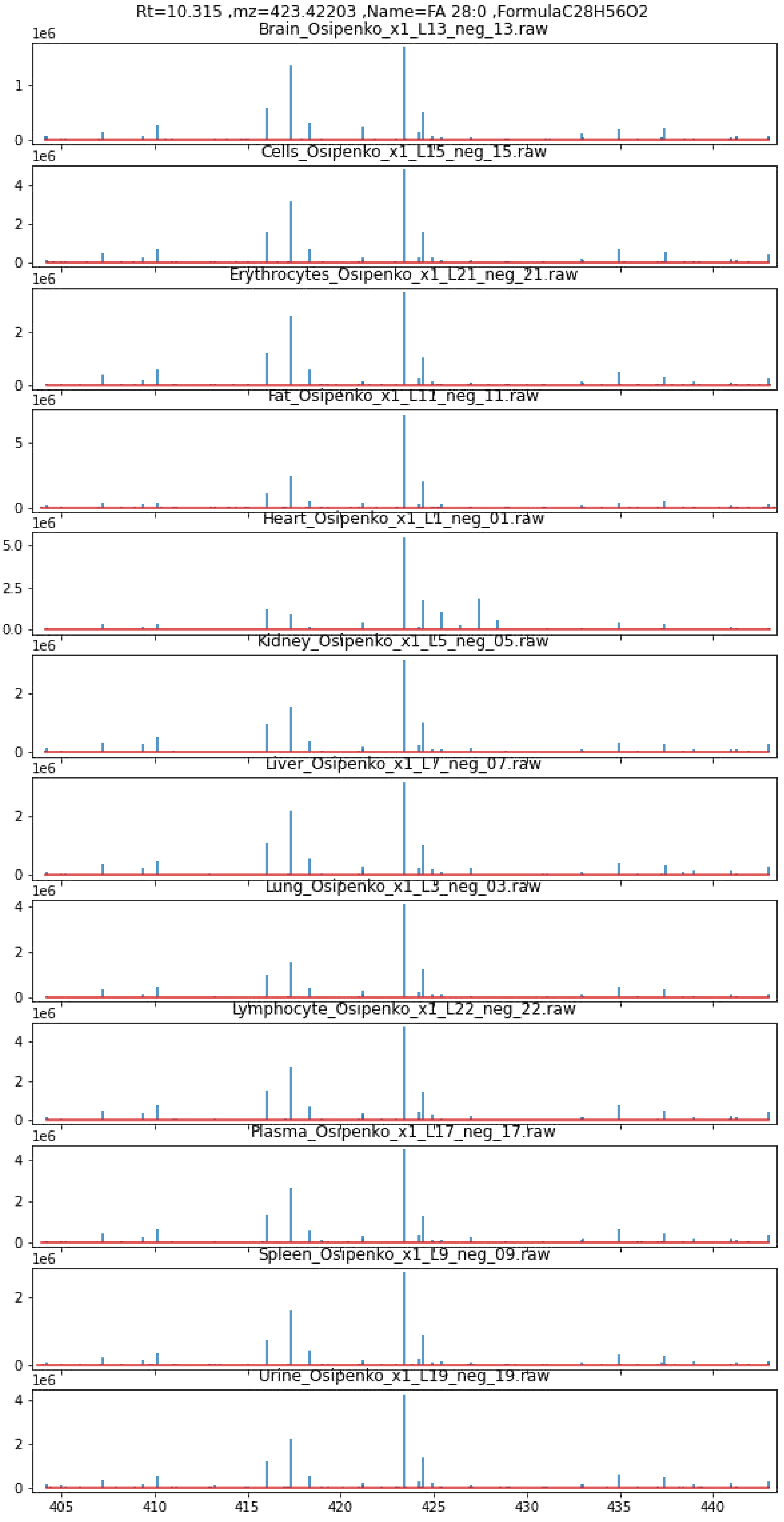
Rt=10.315 ,mz=423.42203 ,Name=FA 28:0 ,FormulaC28H56O2
Brain_Osipenko_x1_L13_neg_13.raw
Cells_Osipenko_x1_L15_neg_15.raw
Erythrocytes_Osipenko_x1_L21_neg_21.raw
Fat_Osipenko_x1_L11_neg_11.raw
Heart_Osipenko_x1_L1_neg_01.raw
Kidney_Osipenko_x1_L5_neg_05.raw
Liver_Osipenko_x1_L7_neg_07.raw
Lung_Osipenko_x1_L3_neg_03.raw
Lymphocyte_Osipenko_x1_L22_neg_22.raw
Plasma_Osipenko_x1_L17_neg_17.raw
Spleen_Osipenko_x1_L9_neg_09.raw
Urine_Osipenko_x1_L19_neg_19.raw
1e6
405
410
415
420
425
430
435
440

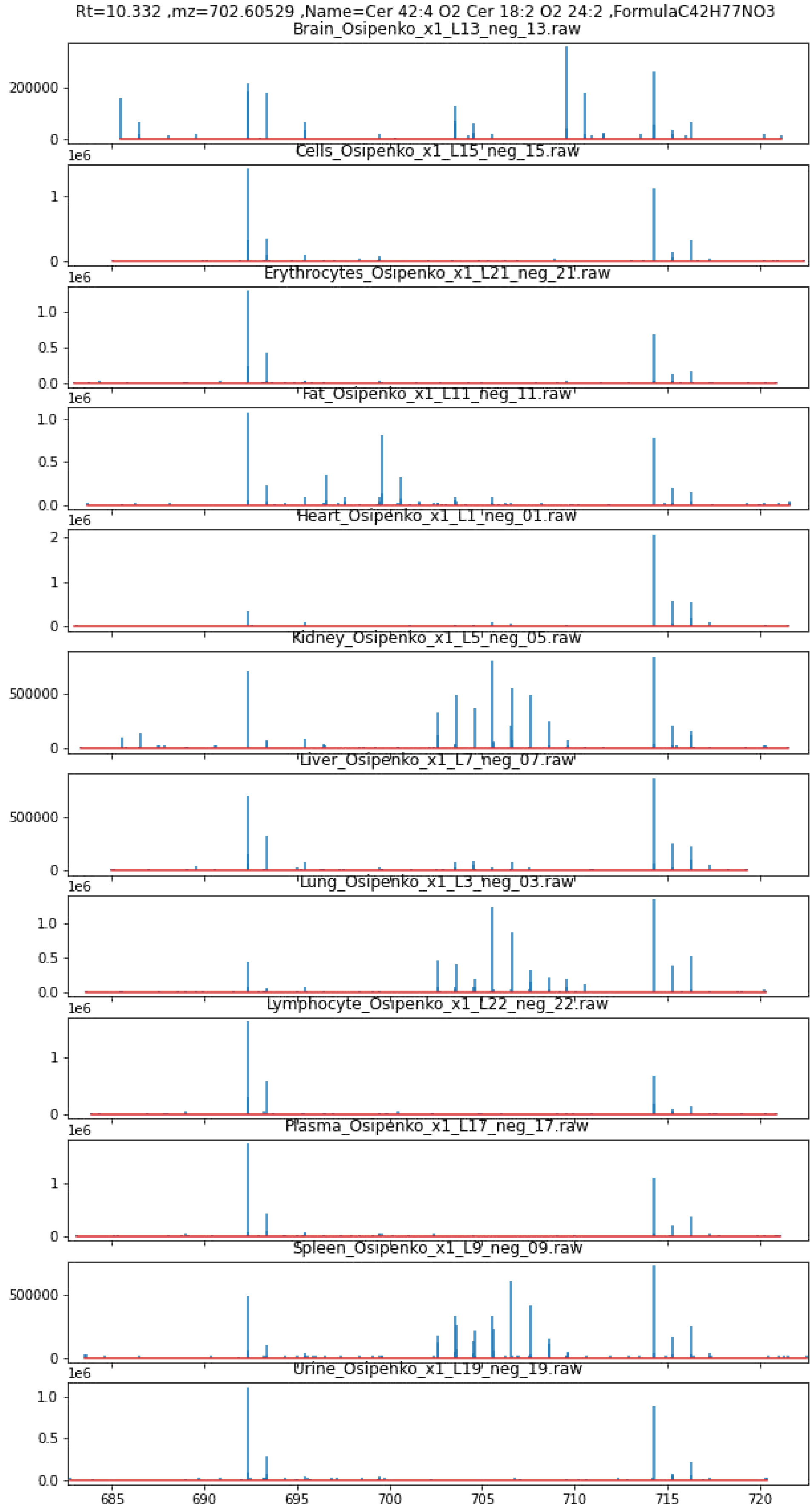
Rt=10.332 ,mz=702.60529 ,Name=Cer 42:4 O2 Cer 18:2 O2 24:2 ,FormulaC42H77NO3
Brain_Osipenko_x1_L13_neg_13.raw
Cells_Osipenko_x1_L15_neg_15.raw
Erythrocytes_Osipenko_x1_L21_neg_21.raw
Fat_Osipenko_x1_L11_neg_11.raw
Heart_Osipenko_x1_L1_neg_01.raw
Kidney_Osipenko_x1_L5_neg_05.raw
Liver_Osipenko_x1_L7_neg_07.raw
Lung_Osipenko_x1_L3_neg_03.raw
Lymphocyte_Osipenko_x1_L22_neg_22.raw
Plasma_Osipenko_x1_L17_neg_17.raw
Spleen_Osipenko_x1_L9_neg_09.raw
Urine_Osipenko_x1_L19_neg_19.raw
1e6
200000
500000
0
0.5
1.0
1
2
685
690
695
700
705
710
715
720

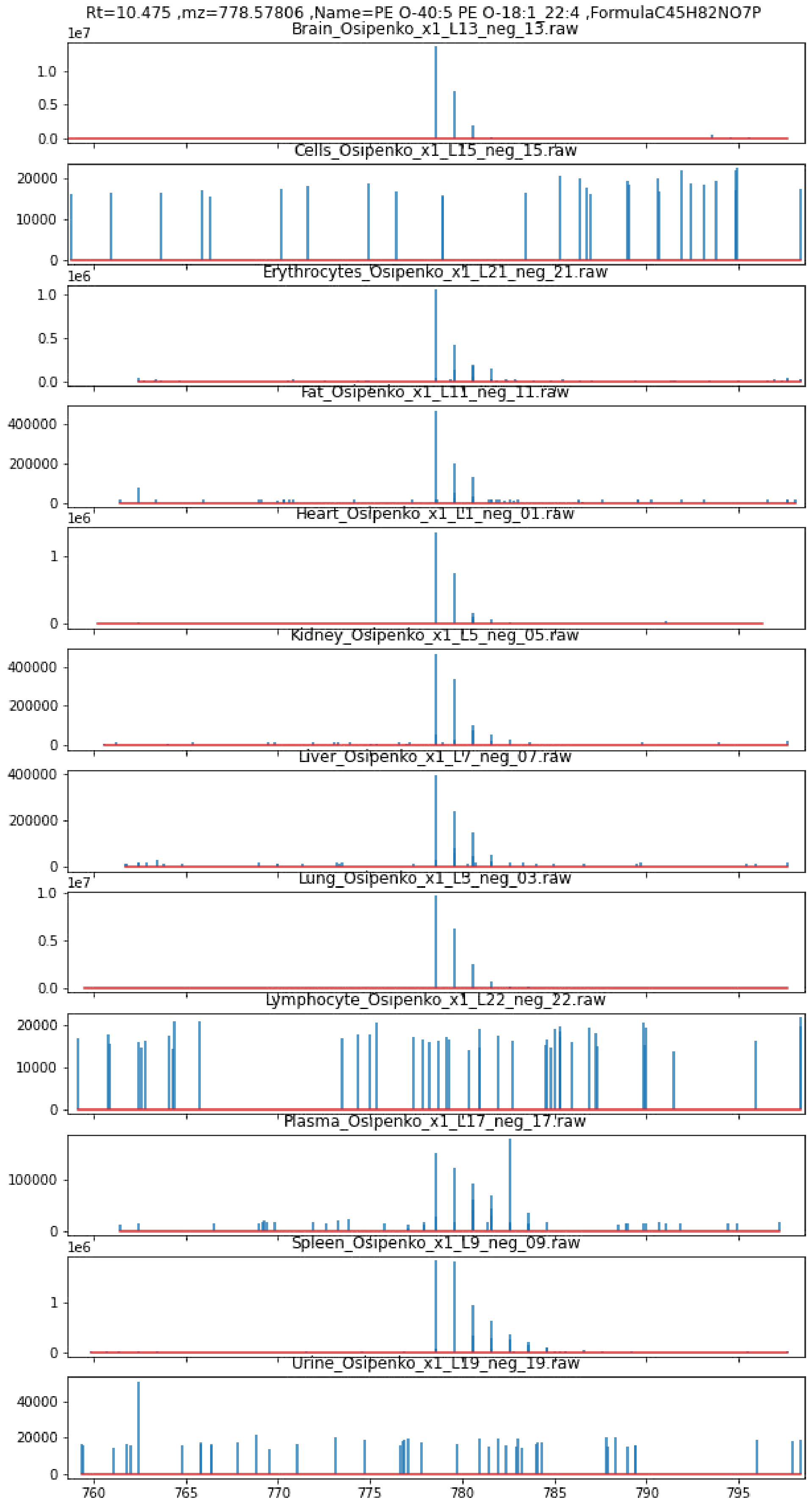
Rt=10.475 ,mz=778.57806 ,Name=PE O-40:5 PE O-18:1_22:4 ,FormulaC45H82NO7P
Brain_Osipenko_x1_L13_neg_13.raw
Cells_Osipenko_x1_L15_neg_15.raw
Erythrocytes_Osipenko_x1_L21_neg_21.raw
Fat_Osipenko_x1_L11_neg_11.raw
Heart_Osipenko_x1_L1_neg_01.raw
Kidney_Osipenko_x1_L5_neg_05.raw
Liver_Osipenko_x1_L7_neg_07.raw
Lung_Osipenko_x1_L3_neg_03.raw
Lymphocyte_Osipenko_x1_L22_neg_22.raw
Plasma_Osipenko_x1_L17_neg_17.raw
Spleen_Osipenko_x1_L9_neg_09.raw
Urine_Osipenko_x1_L19_neg_19.raw

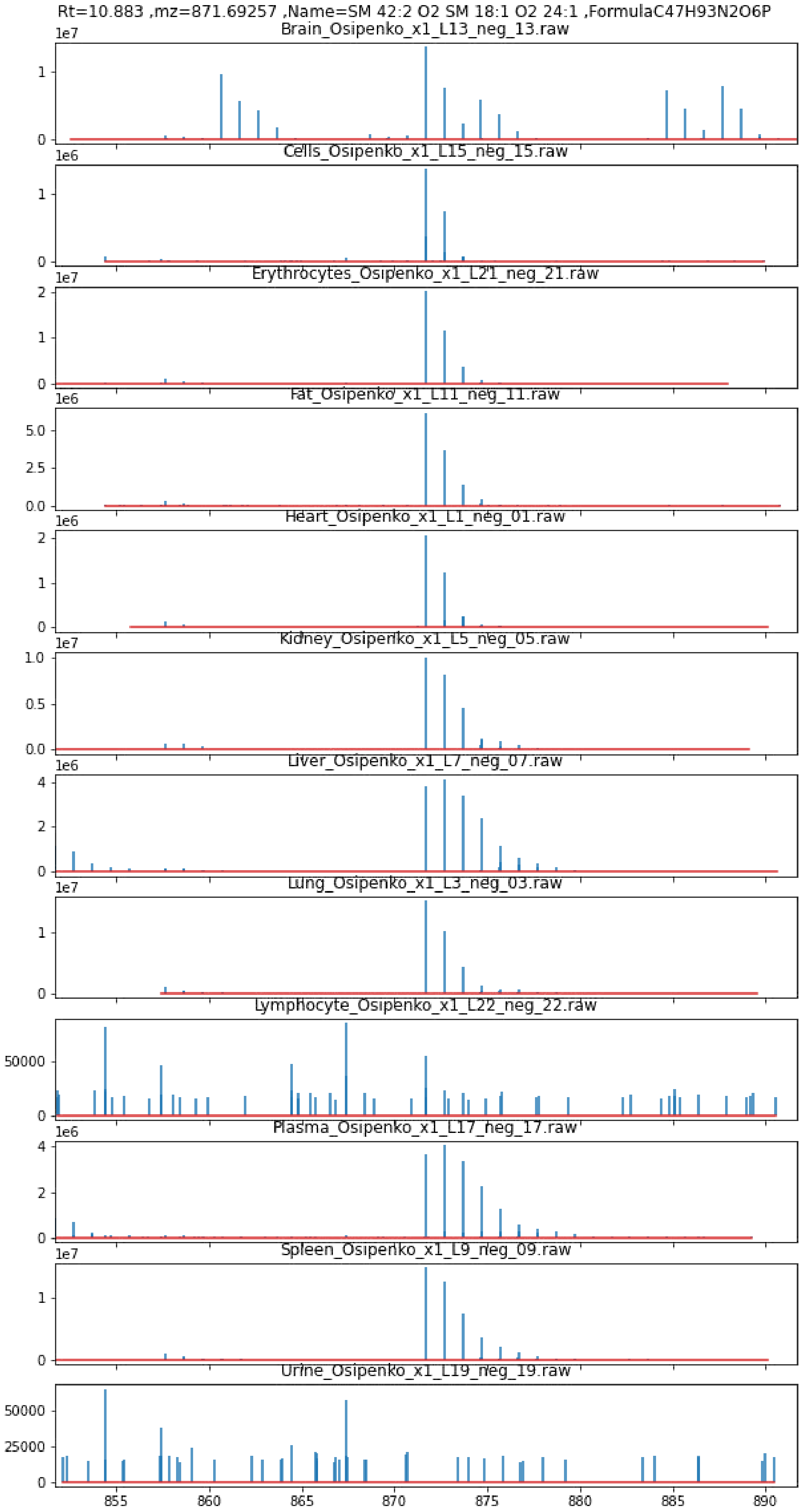

Rt=10.883 ,mz=871.69257 ,Name=SM 42:2 O2 SM 18:1 O2 24:1 ,FormulaC47H93N2O6P
Brain_Osipenko_x1_L13_neg_13.raw
Cells_Osipenko_x1_L15_neg_15.raw
Erythrocytes_Osipenko_x1_L21_neg_21.raw
Fat_Osipenko_x1_L11_neg_11.raw
Heart_Osipenko_x1_L1_neg_01.raw
Kidney_Osipenko_x1_L5_neg_05.raw
Liver_Osipenko_x1_L7_neg_07.raw
Lung_Osipenko_x1_L3_neg_03.raw
Lymphocyte_Osipenko_x1_L22_neg_22.raw
Plasma_Osipenko_x1_L17_neg_17.raw
Spleen_Osipenko_x1_L9_neg_09.raw
Urine_Osipenko_x1_L19_neg_19.raw
1e7
1e6
50000
25000
0
855
860
865
870
875
880
885
890

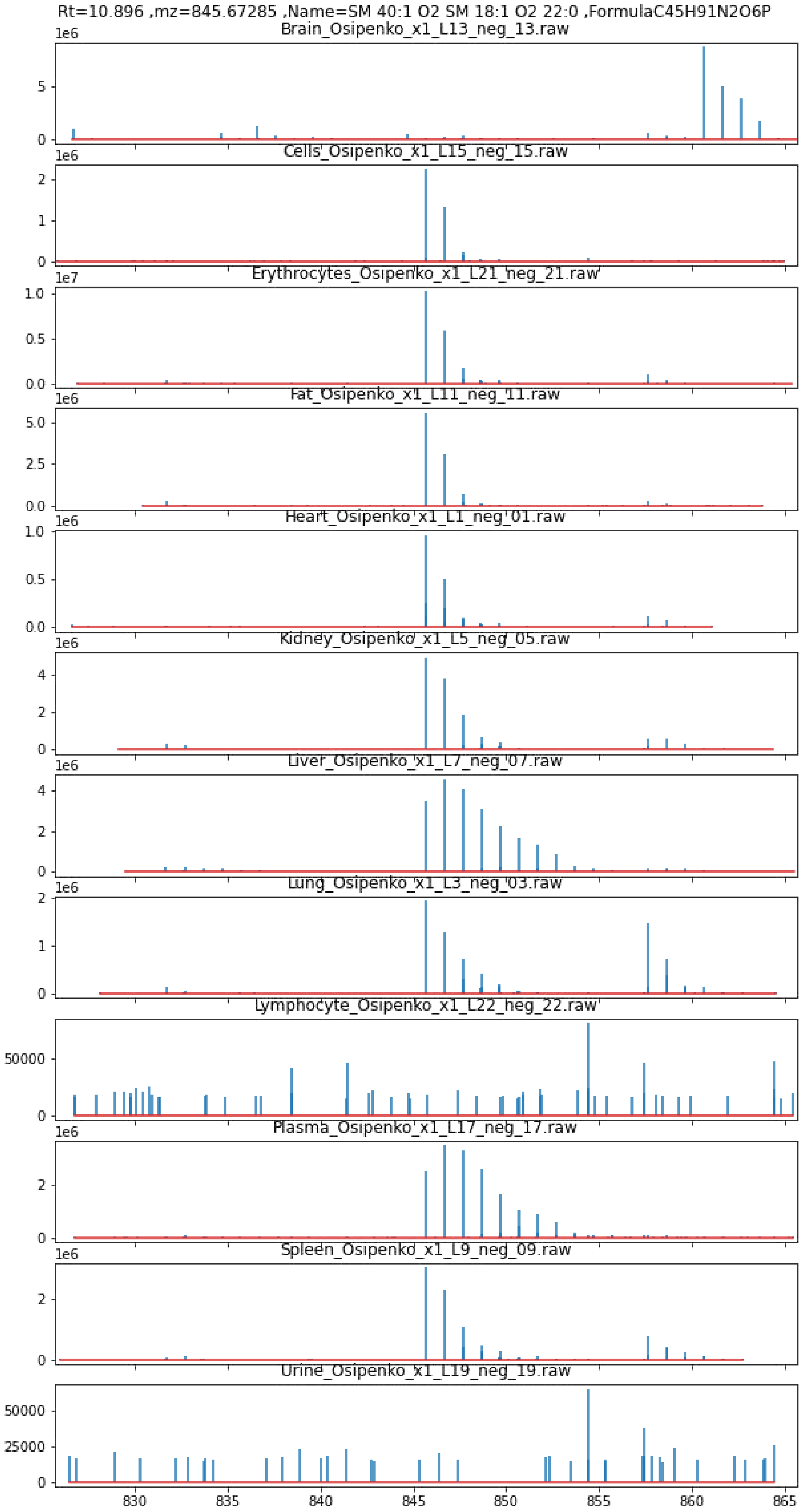

Rt=10.896 ,mz=845.67285 ,Name=SM 40:1 O2 SM 18:1 O2 22:0 ,FormulaC45H91N2O6P
Brain_Osipenko_x1_L13_neg_13.raw
Cells_Osipenko_x1_L15_neg_15.raw
Erythrocytes_Osipenko_x1_L21_neg_21.raw
Fat_Osipenko_x1_L11_neg_11.raw
Heart_Osipenko_x1_L1_neg_01.raw
Kidney_Osipenko_x1_L5_neg_05.raw
Liver_Osipenko_x1_L7_neg_07.raw
Lung_Osipenko_x1_L3_neg_03.raw
Lymphocyte_Osipenko_x1_L22_neg_22.raw
Plasma_Osipenko_x1_L17_neg_17.raw
Spleen_Osipenko_x1_L9_neg_09.raw
Urine_Osipenko_x1_L19_neg_19.raw
830
835
840
845
850
855
860
865

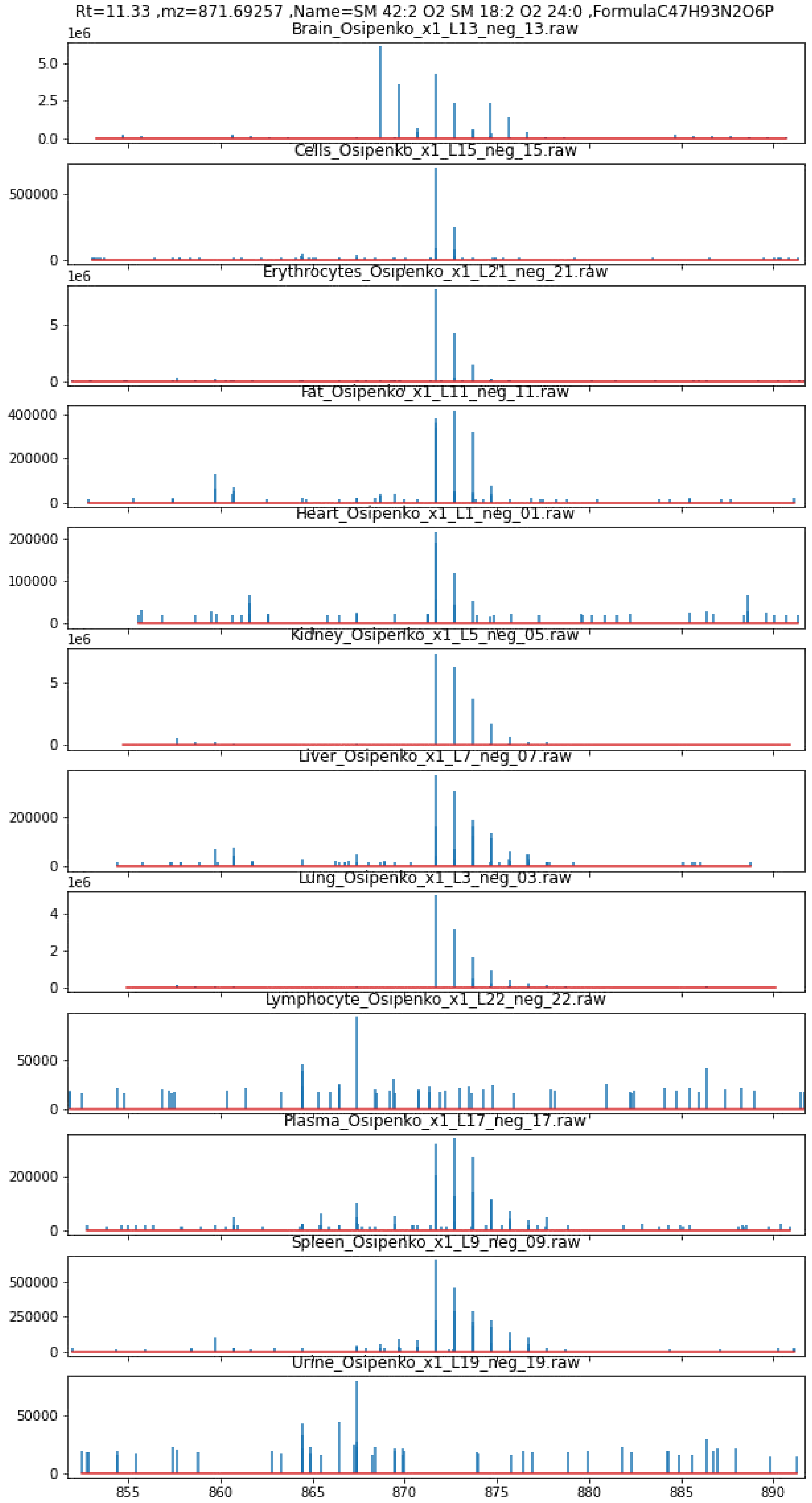

Rt=11.33 ,mz=871.69257 ,Name=SM 42:2 O2 SM 18:2 O2 24:0 ,FormulaC47H93N2O6P
Brain_Osipenko_x1_L13_neg_13.raw
Cells_Osipenko_x1_L15_neg_15.raw
Erythrocytes_Osipenko_x1_L21_neg_21.raw
Fat_Osipenko_x1_L11_neg_11.raw
Heart_Osipenko_x1_L1_neg_01.raw
Kidney_Osipenko_x1_L5_neg_05.raw
Liver_Osipenko_x1_L7_neg_07.raw
Lung_Osipenko_x1_L3_neg_03.raw
Lymphocyte_Osipenko_x1_L22_neg_22.raw
Plasma_Osipenko_x1_L17_neg_17.raw
Spleen_Osipenko_x1_L9_neg_09.raw
Urine_Osipenko_x1_L19_neg_19.raw

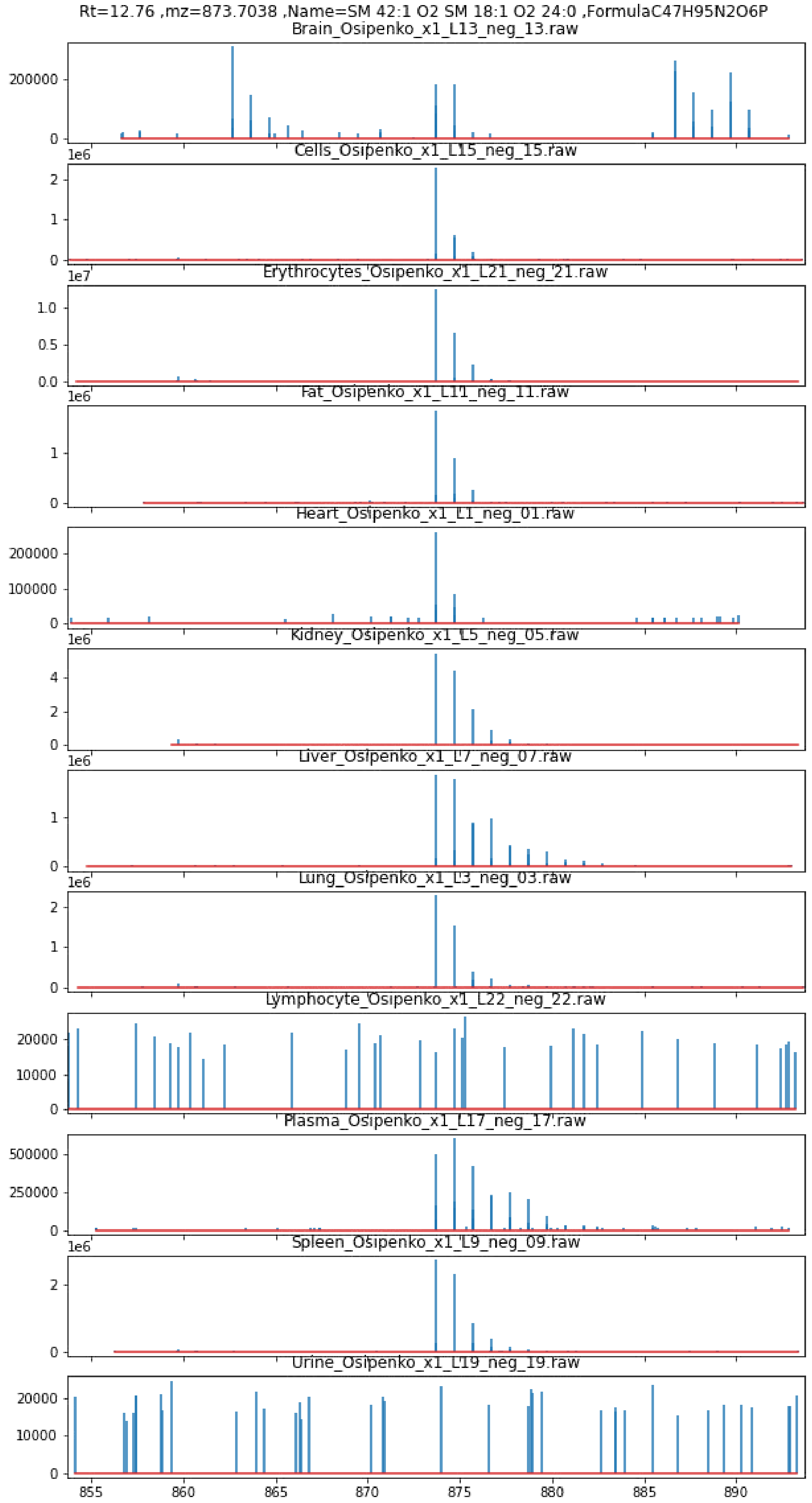

Rt=12.76 ,mz=873.7038 ,Name=SM 42:1 O2 SM 18:1 O2 24:0 ,FormulaC47H95N2O6P
Brain_Osipenko_x1_L13_neg_13.raw
Cells_Osipenko_x1_L15_neg_15.raw
Erythrocytes_Osipenko_x1_L21_neg_21.raw
Fat_Osipenko_x1_L11_neg_11.raw
Heart_Osipenko_x1_L1_neg_01.raw
Kidney_Osipenko_x1_L5_neg_05.raw
Liver_Osipenko_x1_L7_neg_07.raw
Lung_Osipenko_x1_L3_neg_03.raw
Lymphocyte_Osipenko_x1_L22_neg_22.raw
Plasma_Osipenko_x1_L17_neg_17.raw
Spleen_Osipenko_x1_L9_neg_09.raw
Urine_Osipenko_x1_L19_neg_19.raw

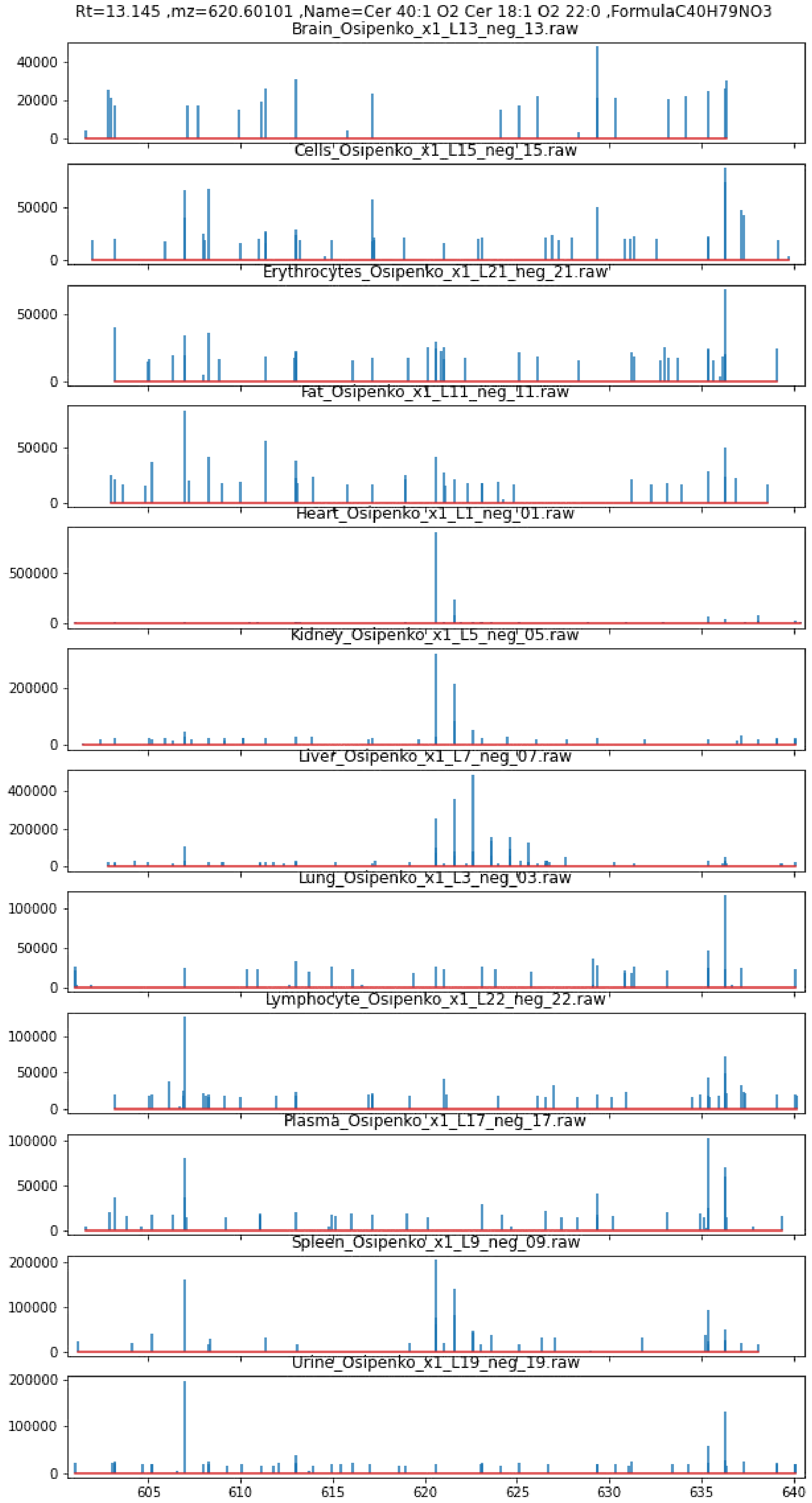
Rt=13.145 ,mz=620.60101 ,Name=Cer 40:1 O2 Cer 18:1 O2 22:0 ,FormulaC40H79NO3
Brain_Osipenko_x1_L13_neg_13.raw
Cells_Osipenko_x1_L15_neg_15.raw
Erythrocytes_Osipenko_x1_L21_neg_21.raw
Fat_Osipenko_x1_L11_neg_11.raw
Heart_Osipenko_x1_L1_neg_01.raw
Kidney_Osipenko_x1_L5_neg_05.raw
Liver_Osipenko_x1_L7_neg_07.raw
Lung_Osipenko_x1_L3_neg_03.raw
Lymphocyte_Osipenko_x1_L22_neg_22.raw
Plasma_Osipenko_x1_L17_neg_17.raw
Spleen_Osipenko_x1_L9_neg_09.raw
Urine_Osipenko_x1_L19_neg_19.raw
605
610
615
620
625
630
635
640
