# Supplementary figures and images for: Untargeted Lipidomics after D2O Administration Reveals the Turnover Rate of Individual Lipids in Various Organs of Living Organisms
Source: Int J Mol Sci. 2023 Jul 21;24(14):11725. doi: 10.3390/ijms241411725 (PMC10380497; doi:10.3390/ijms241411725)

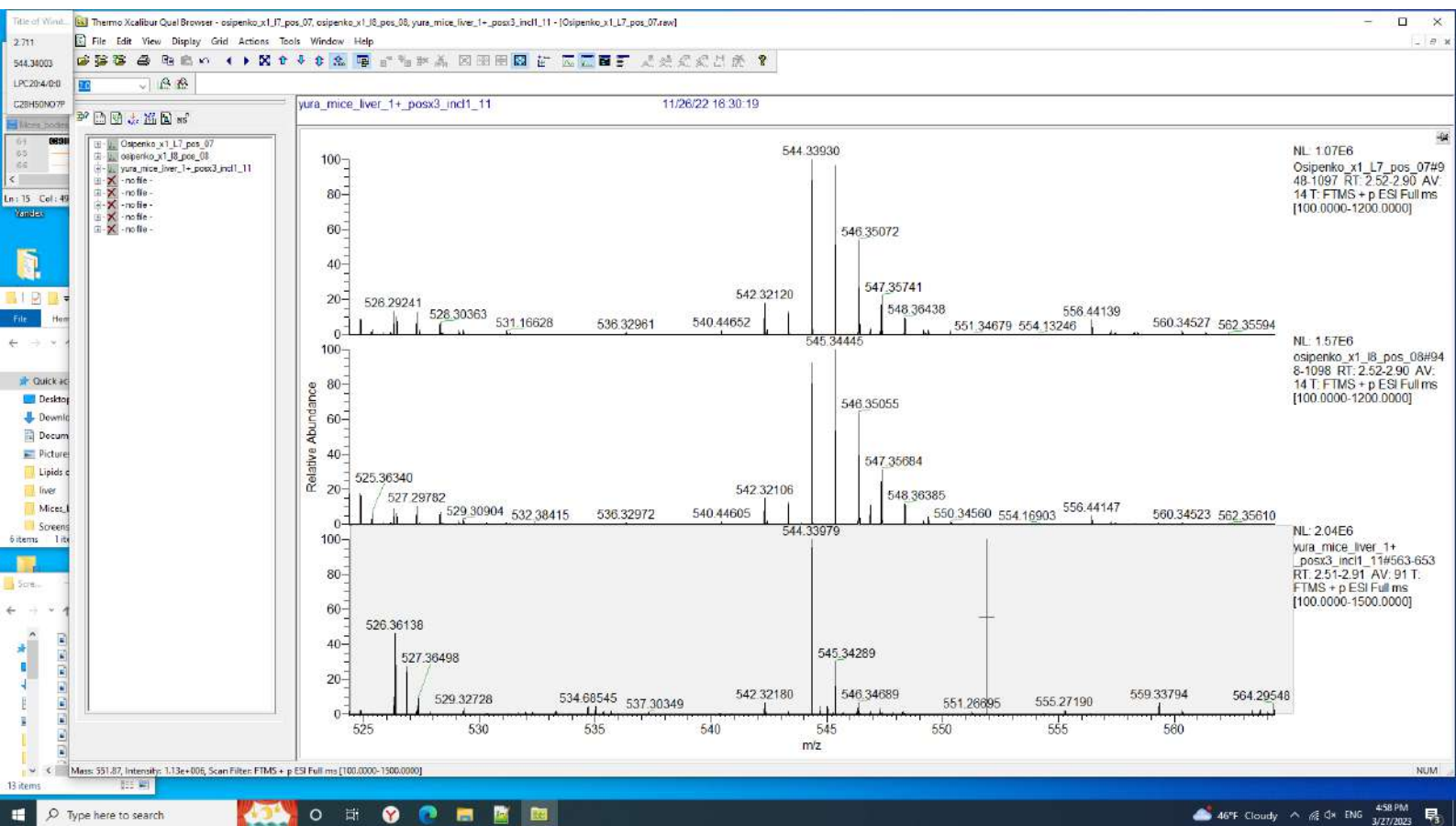

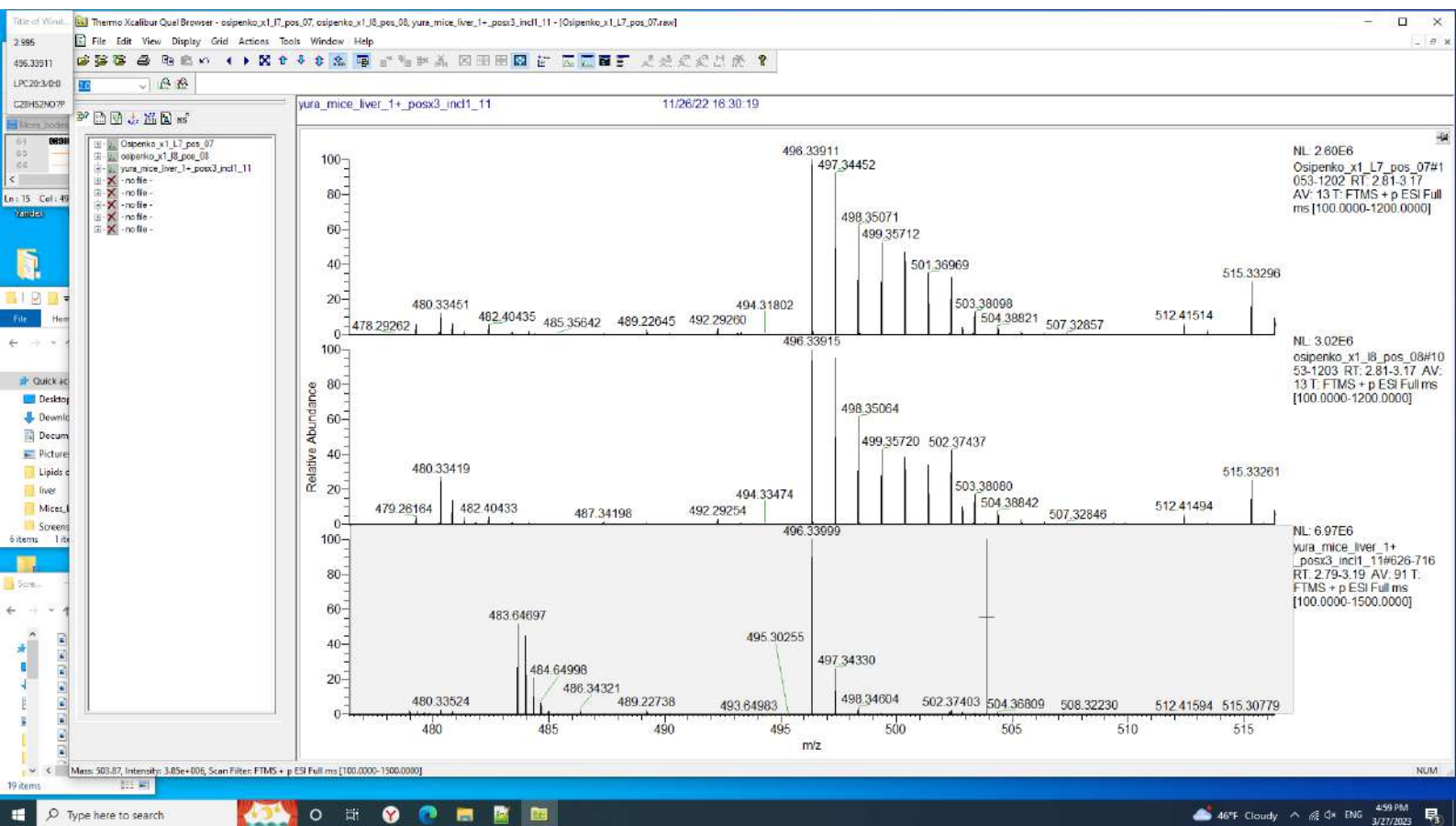

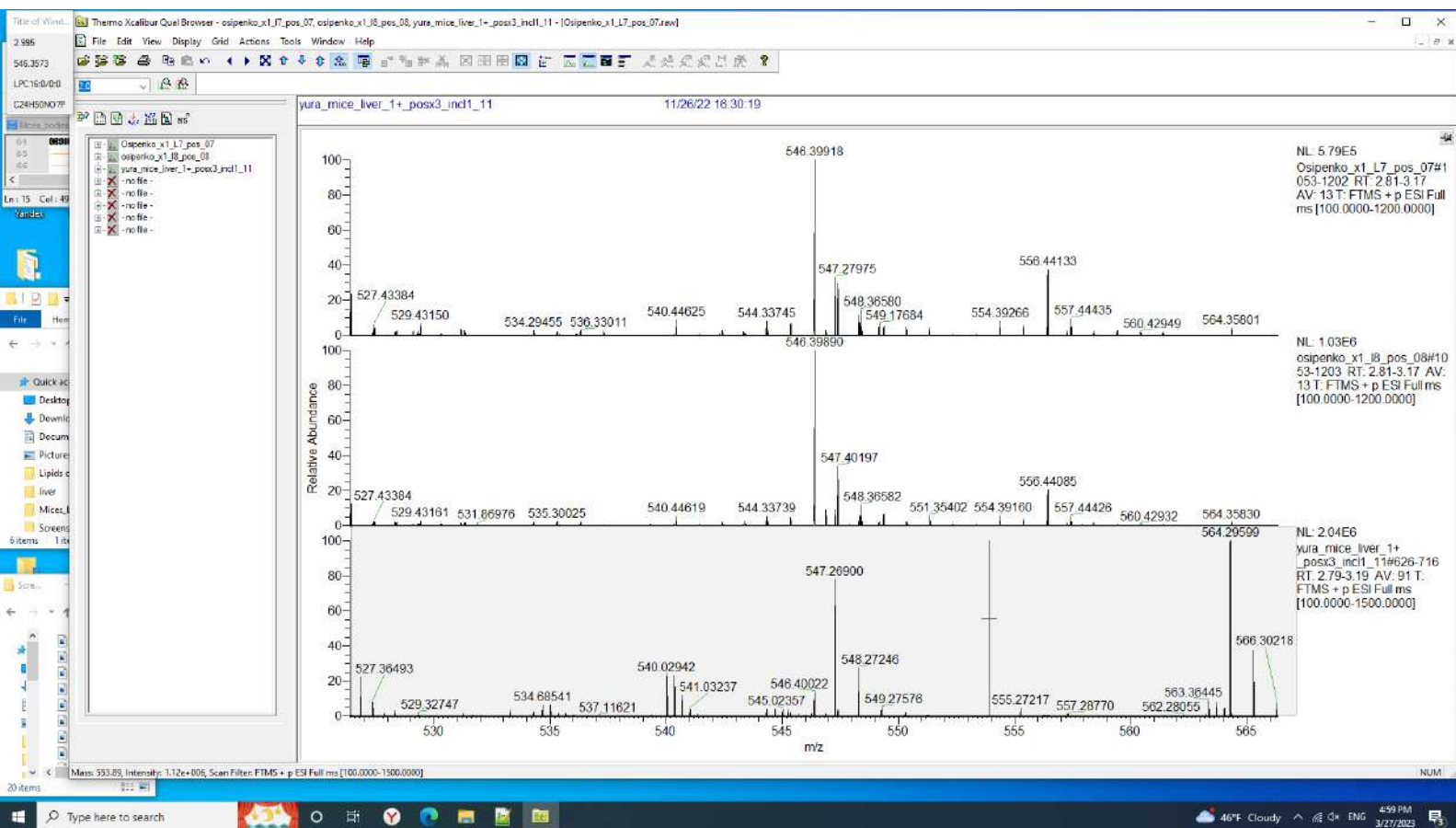

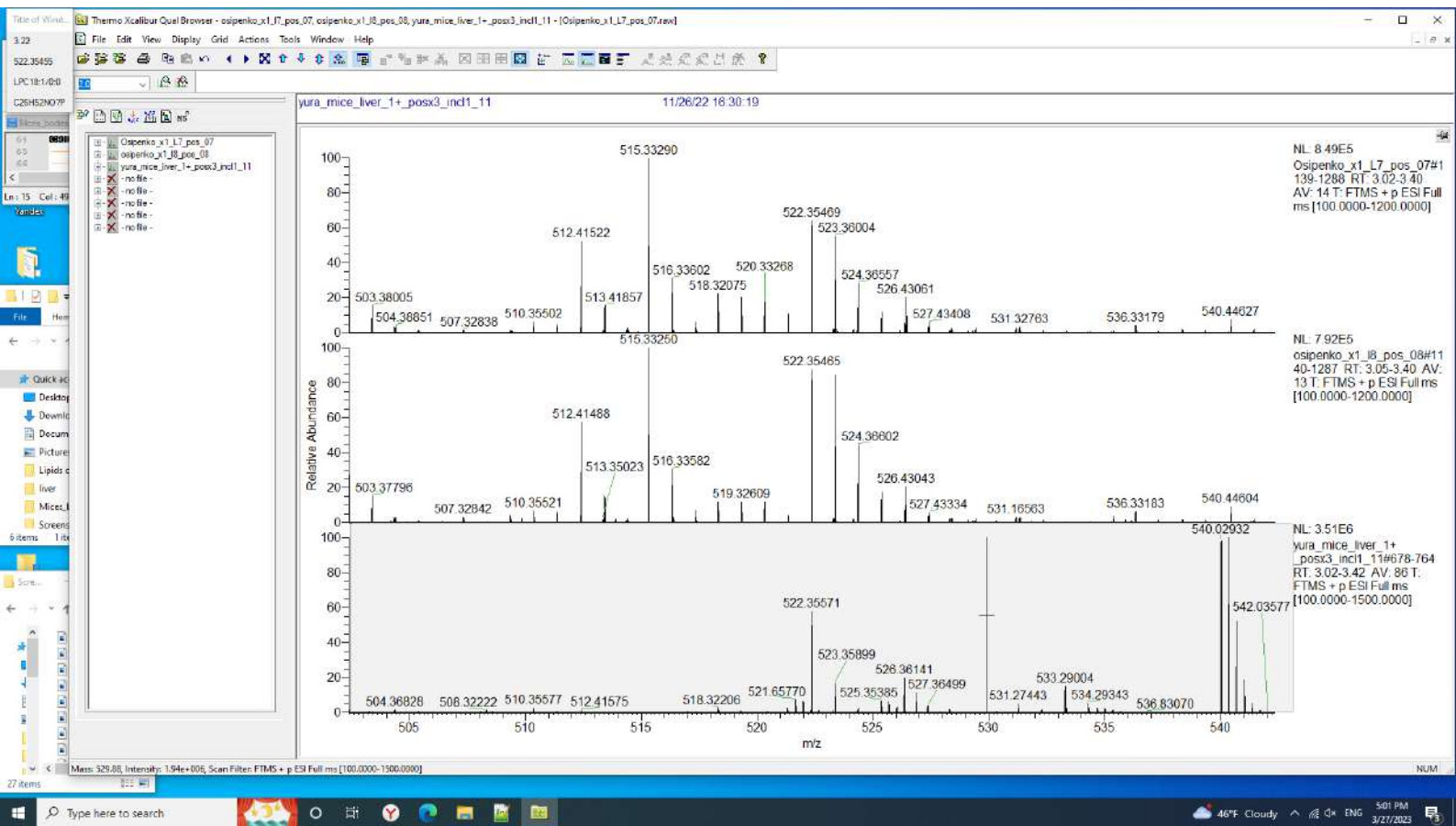

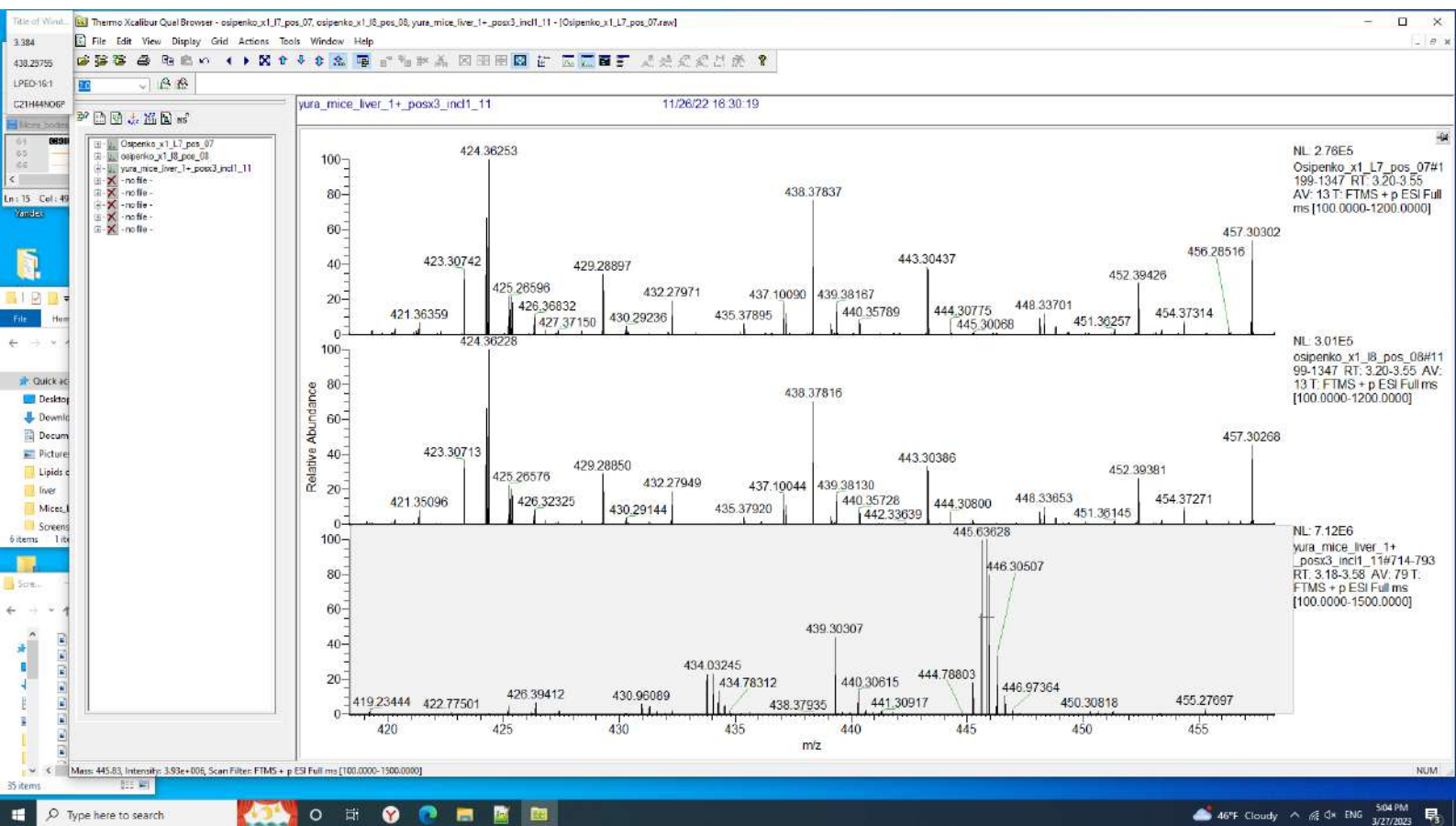

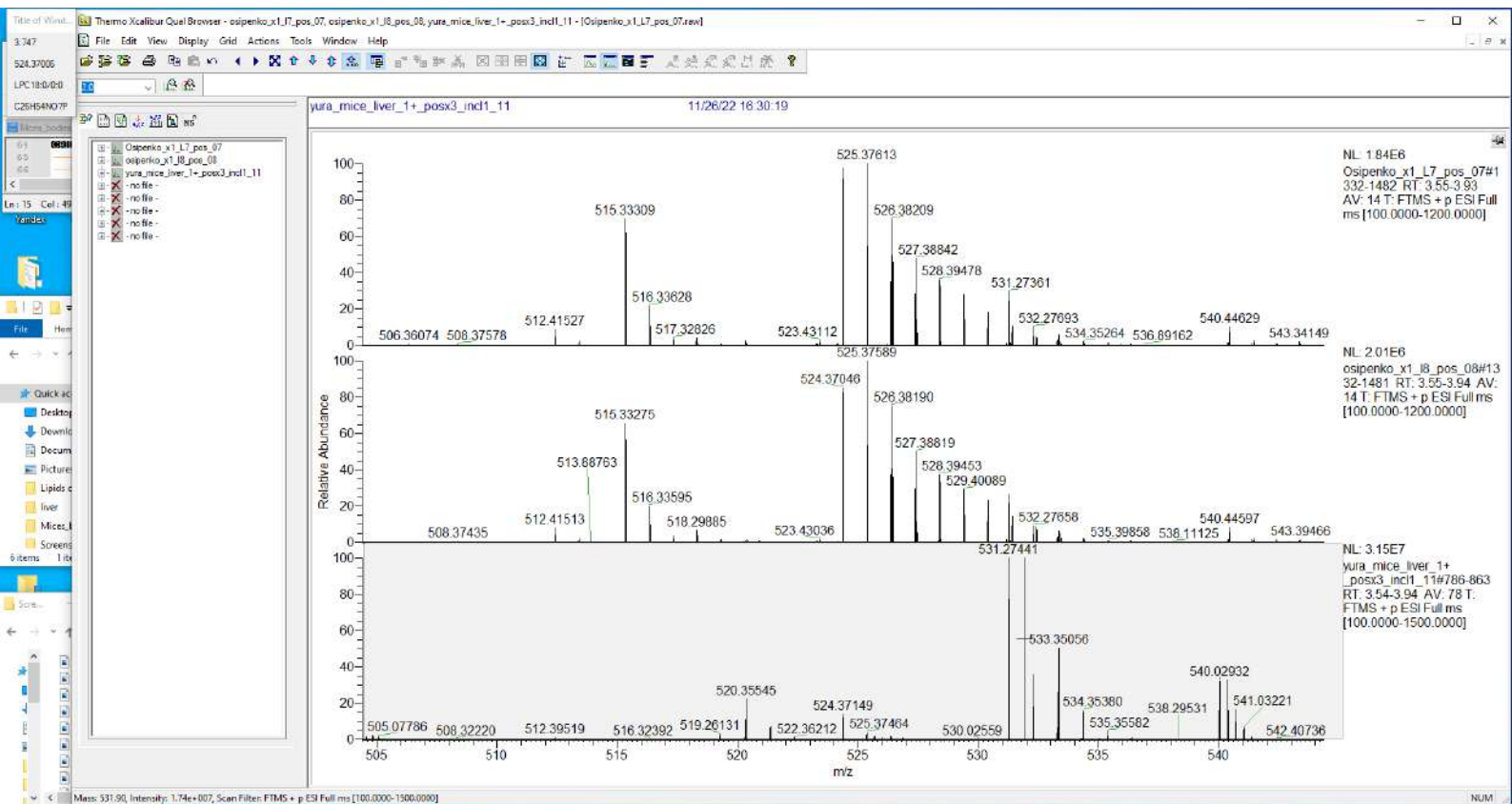

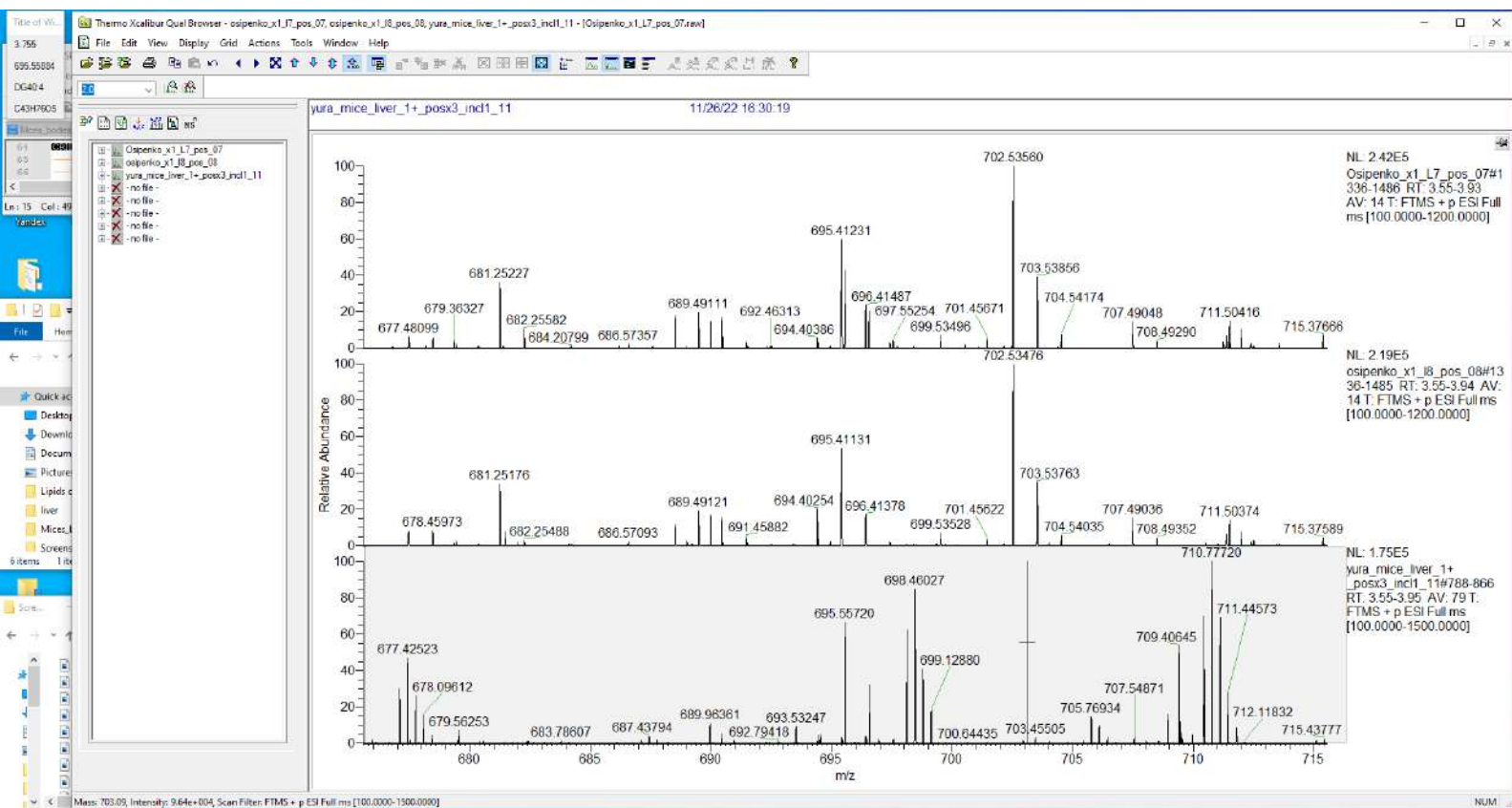

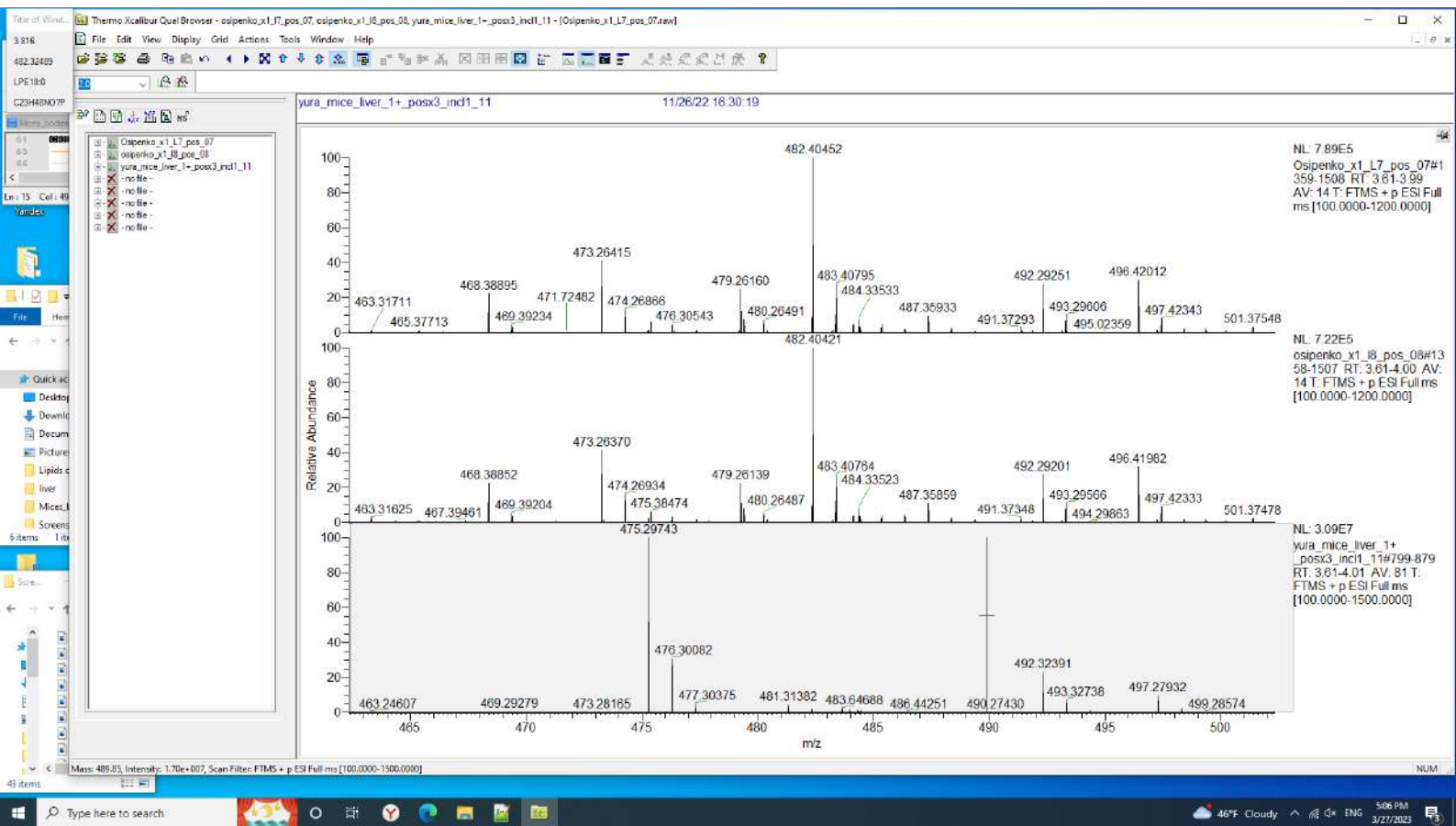

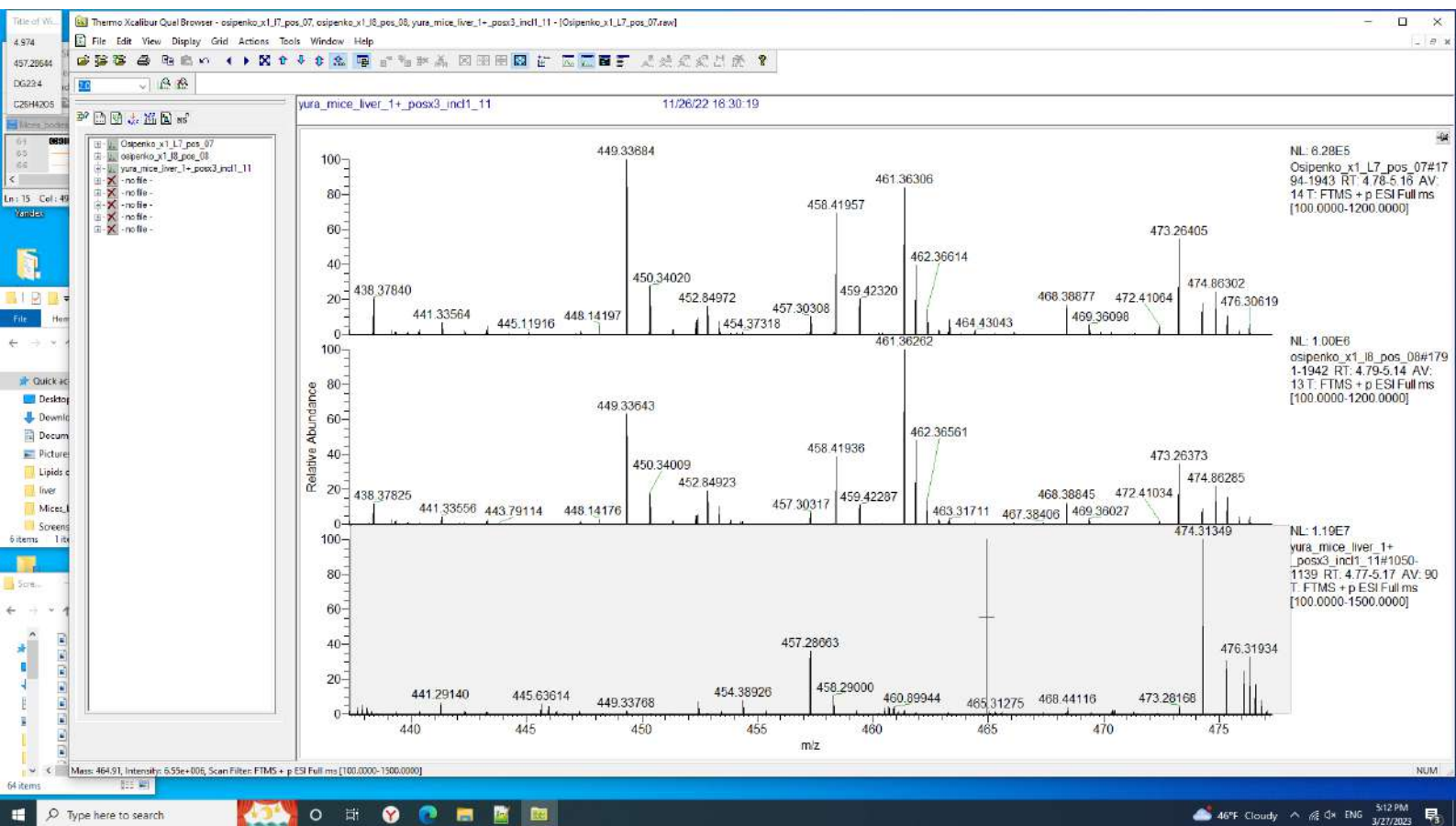

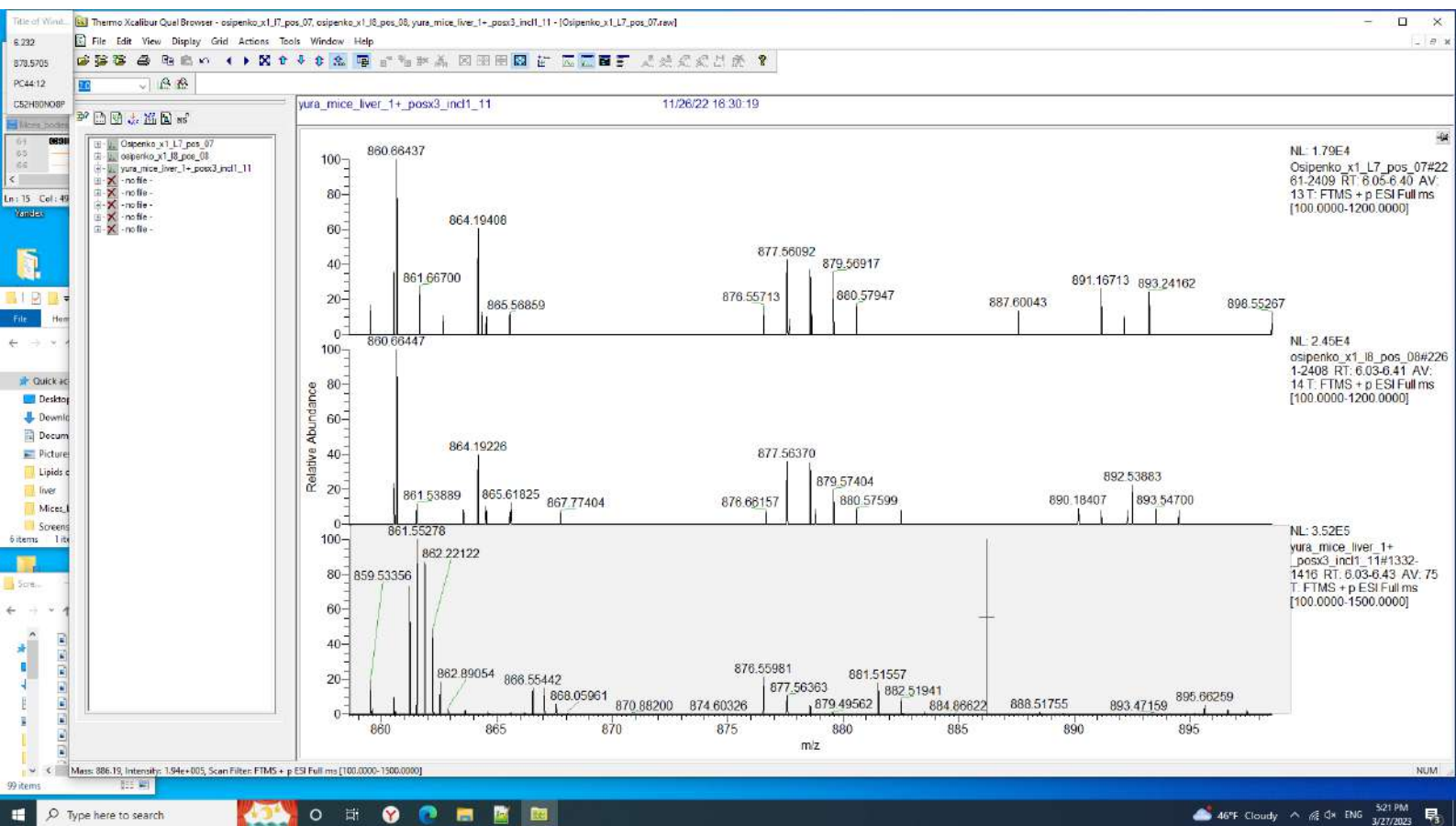

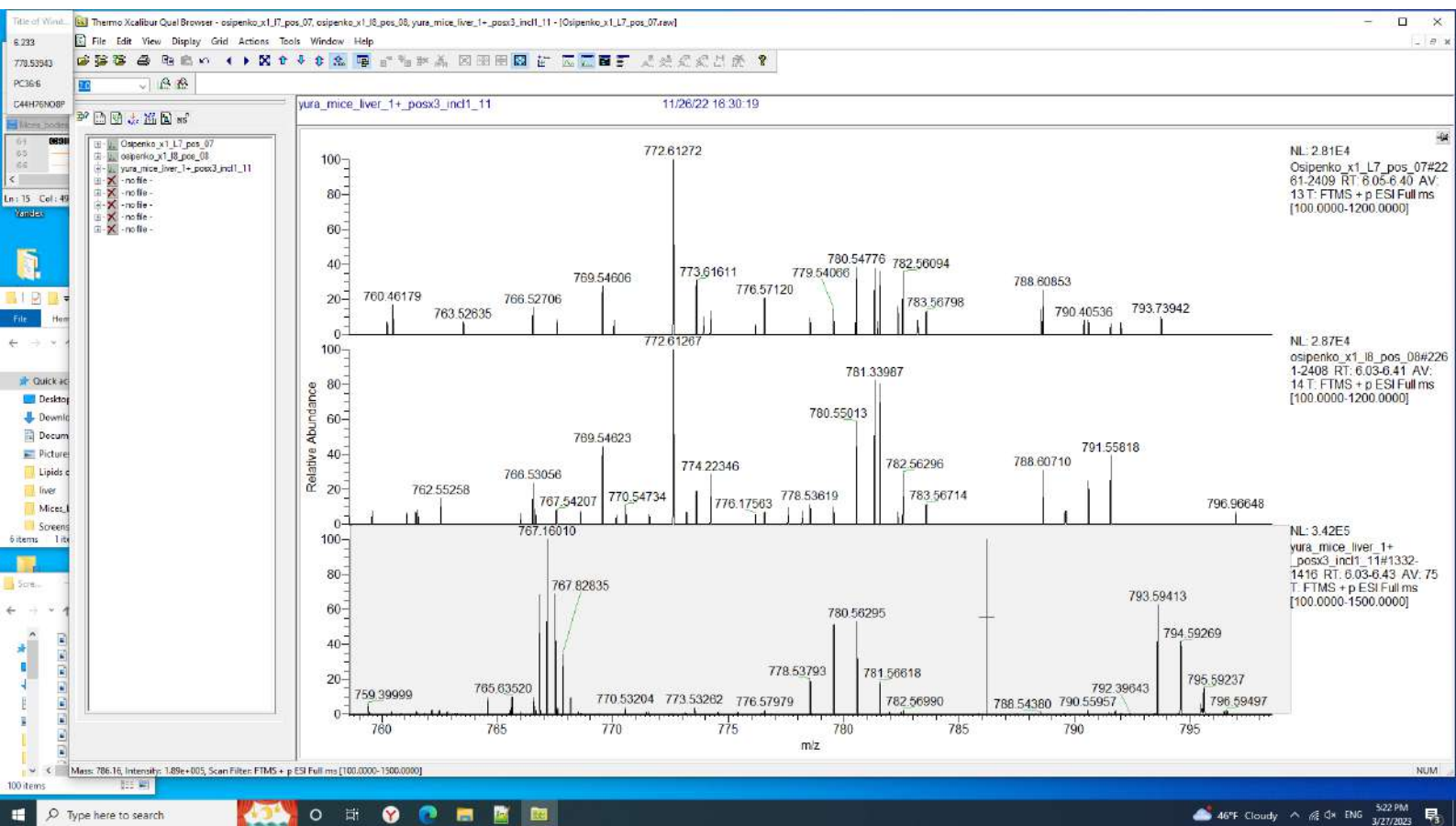

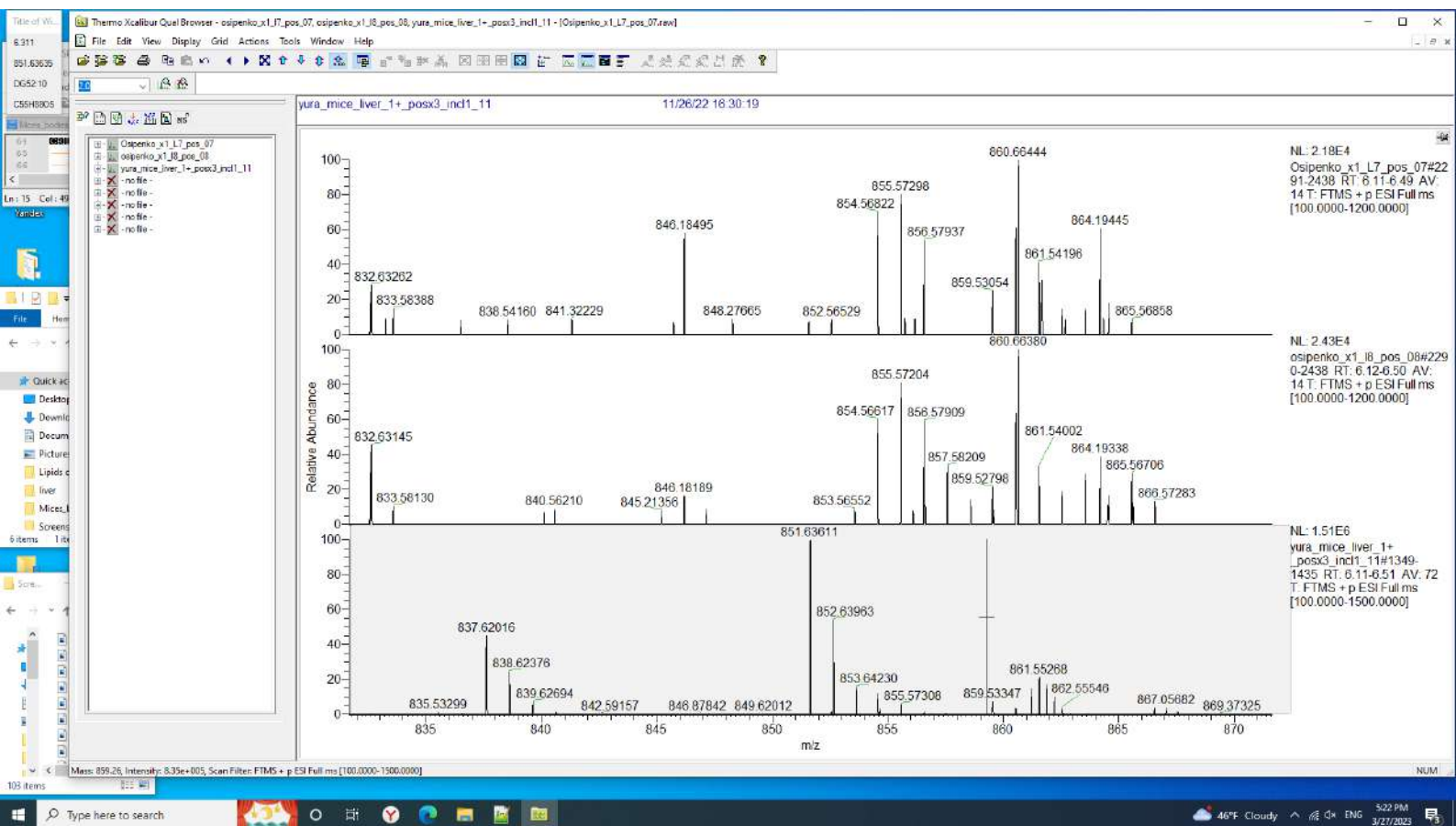

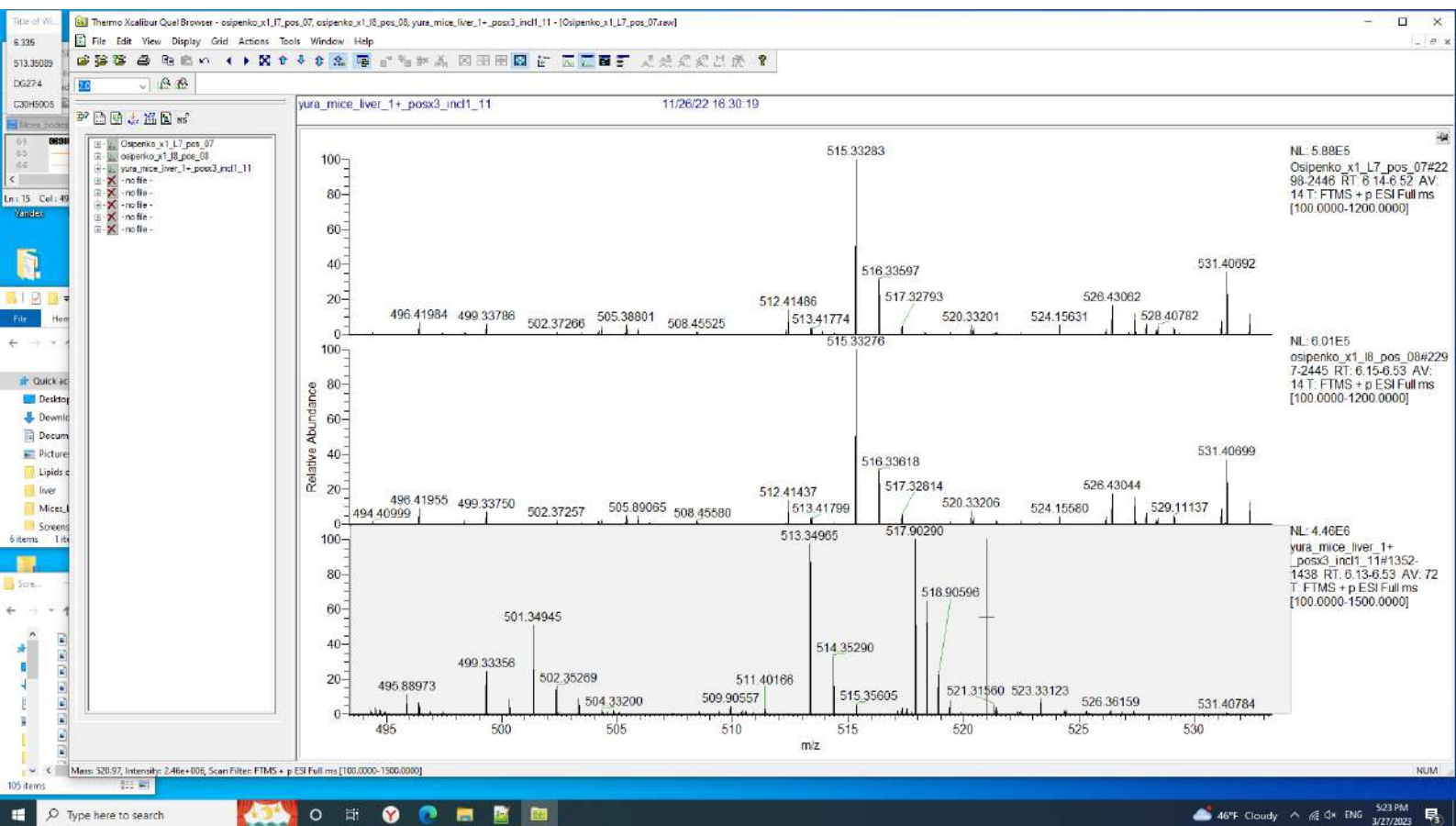

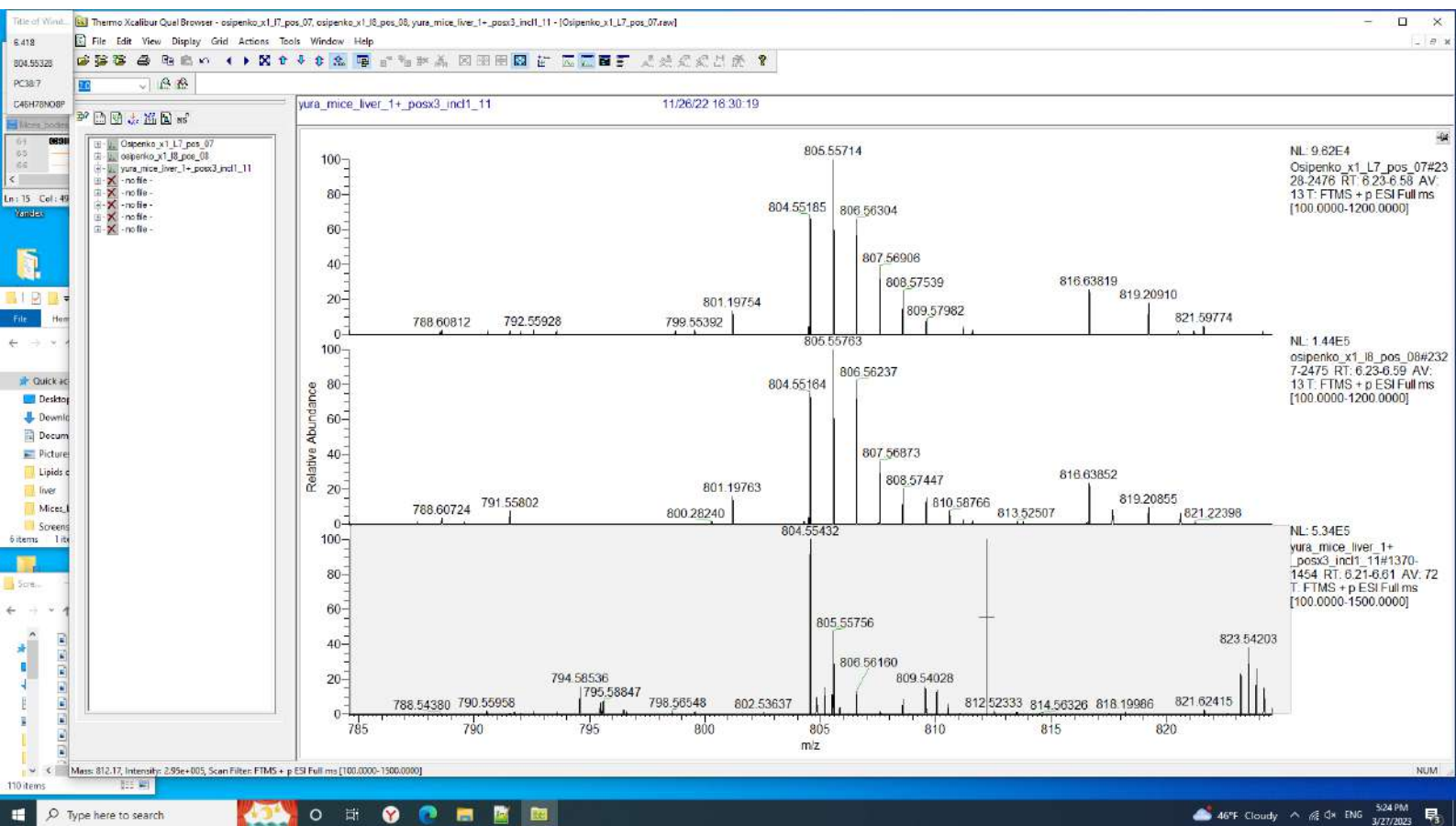

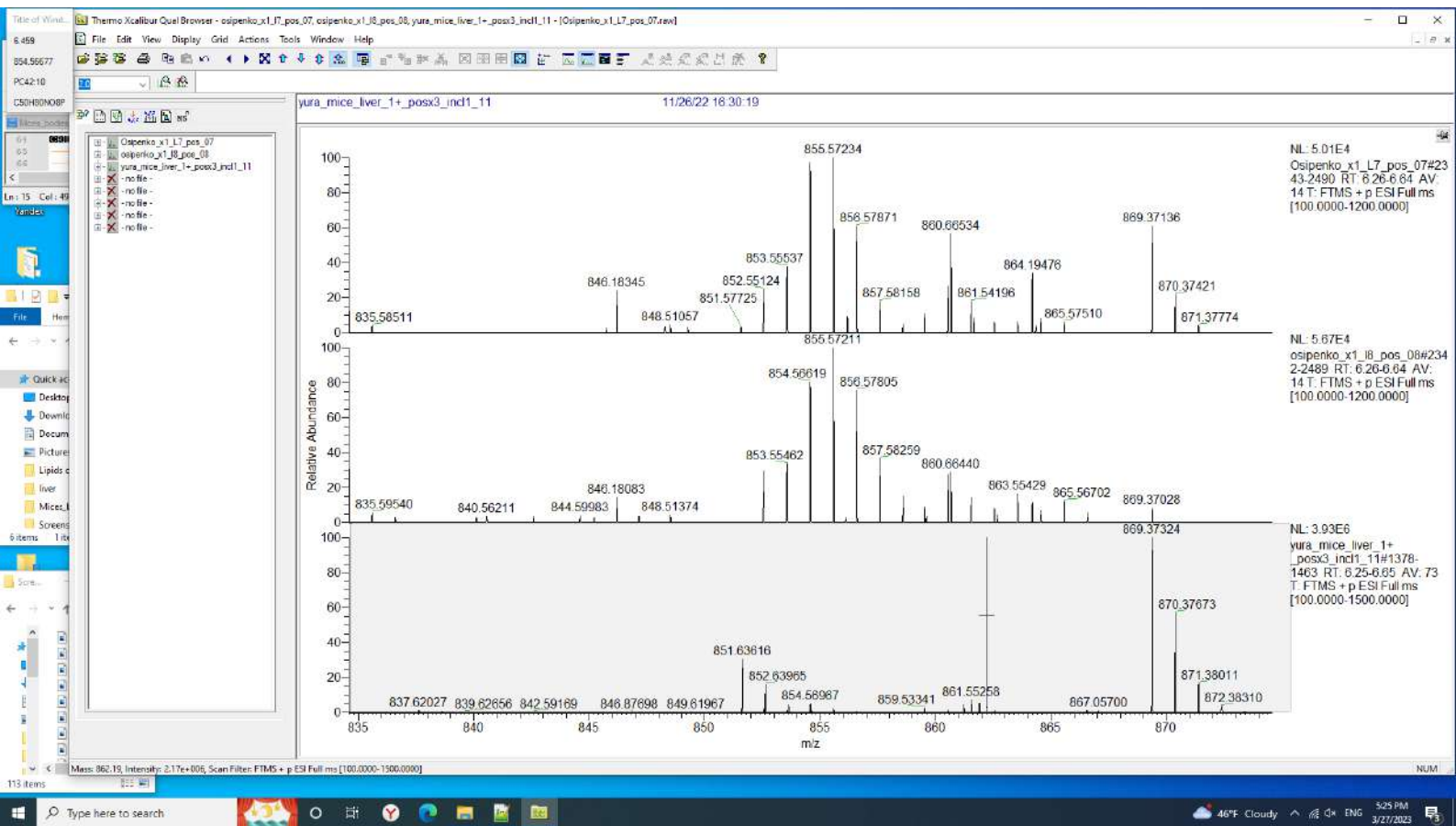

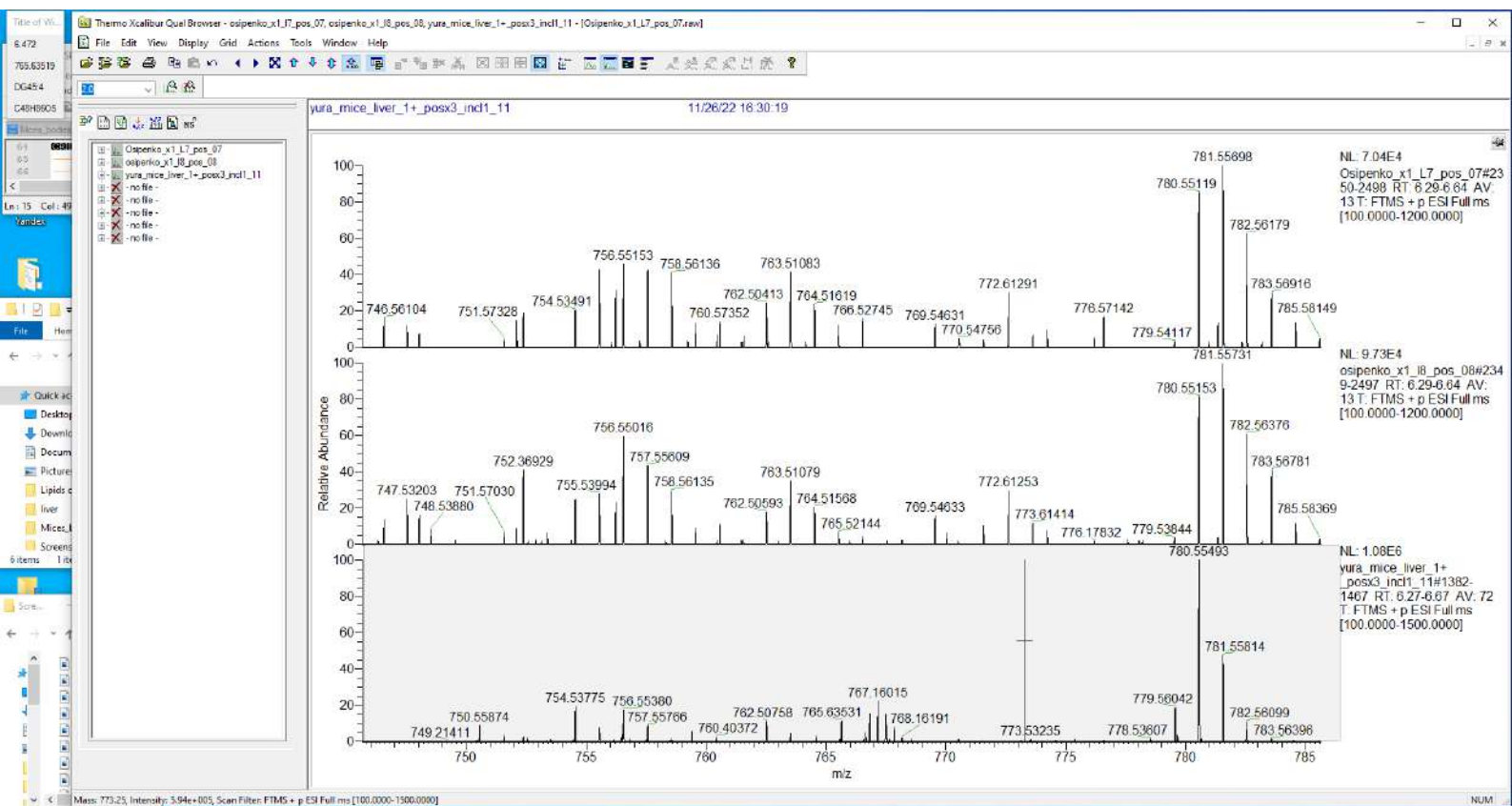

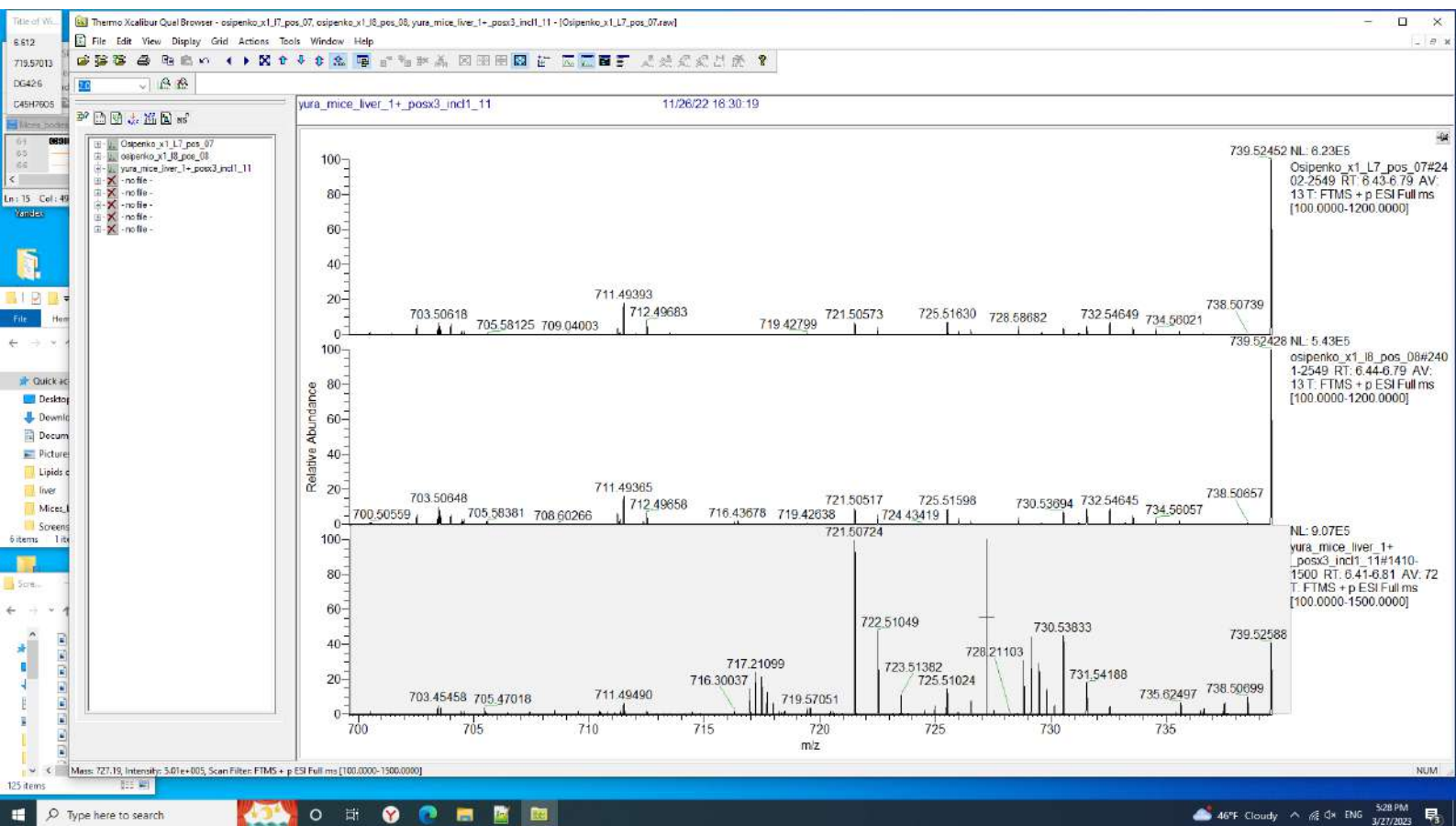

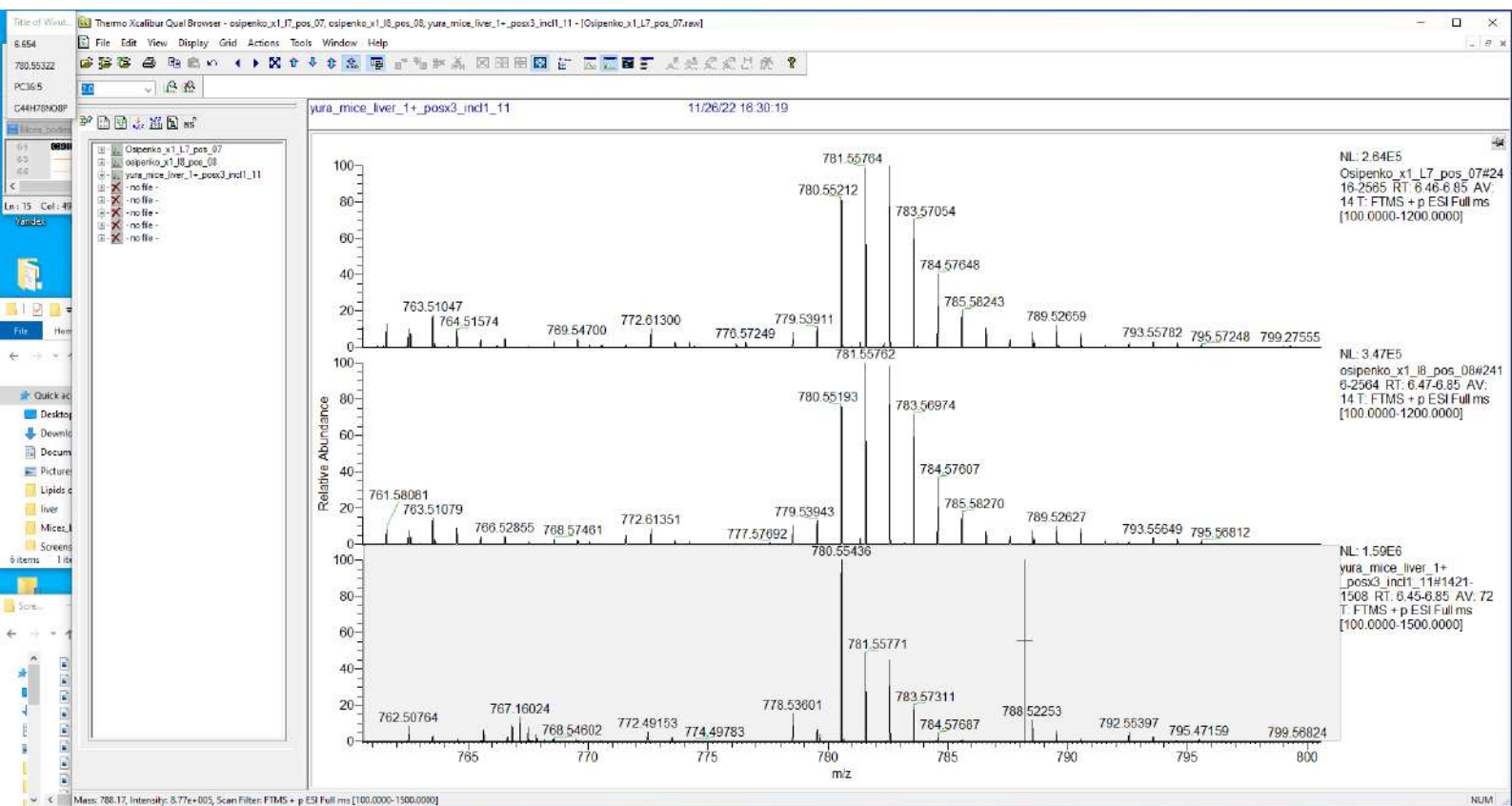

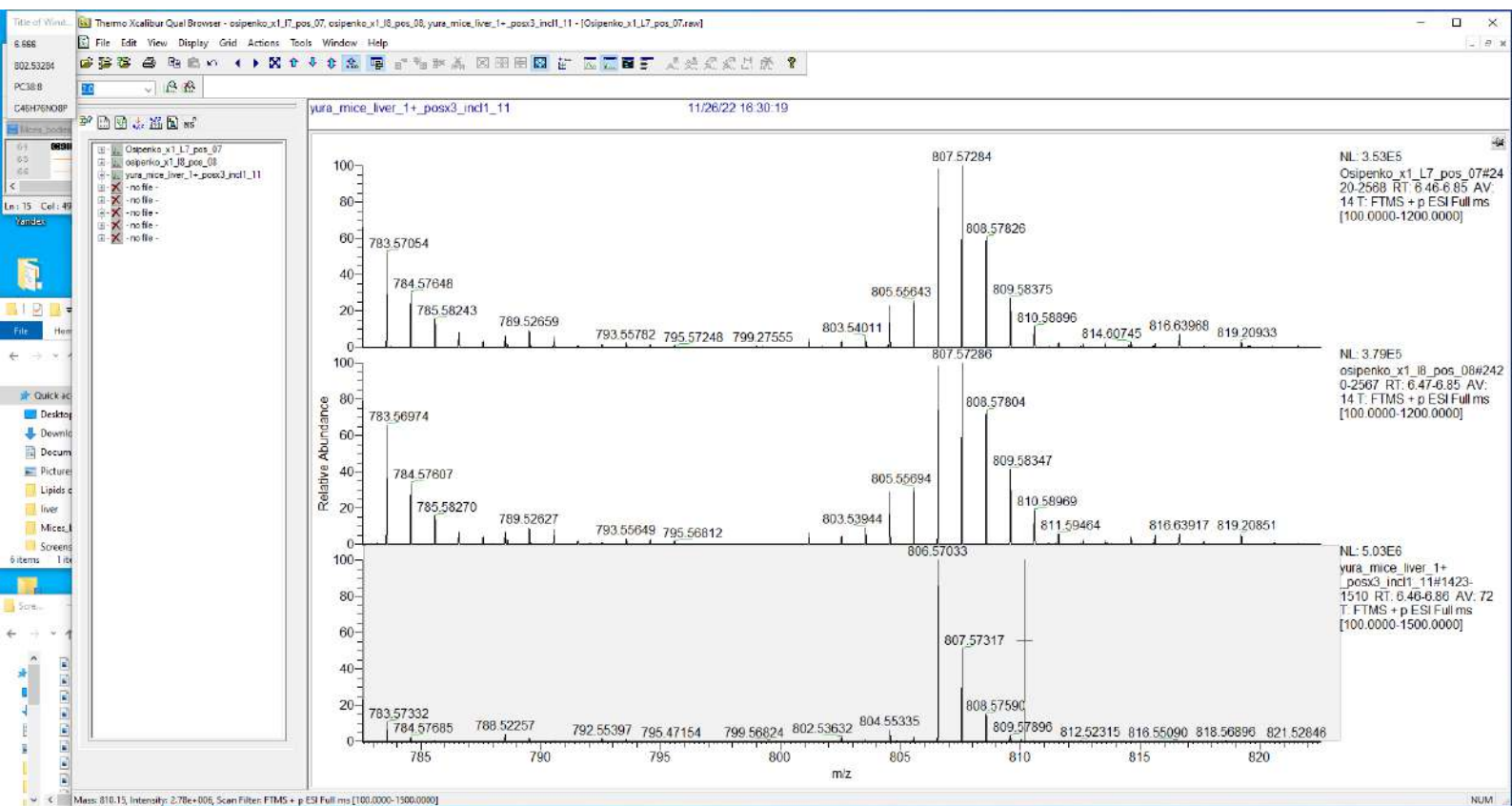

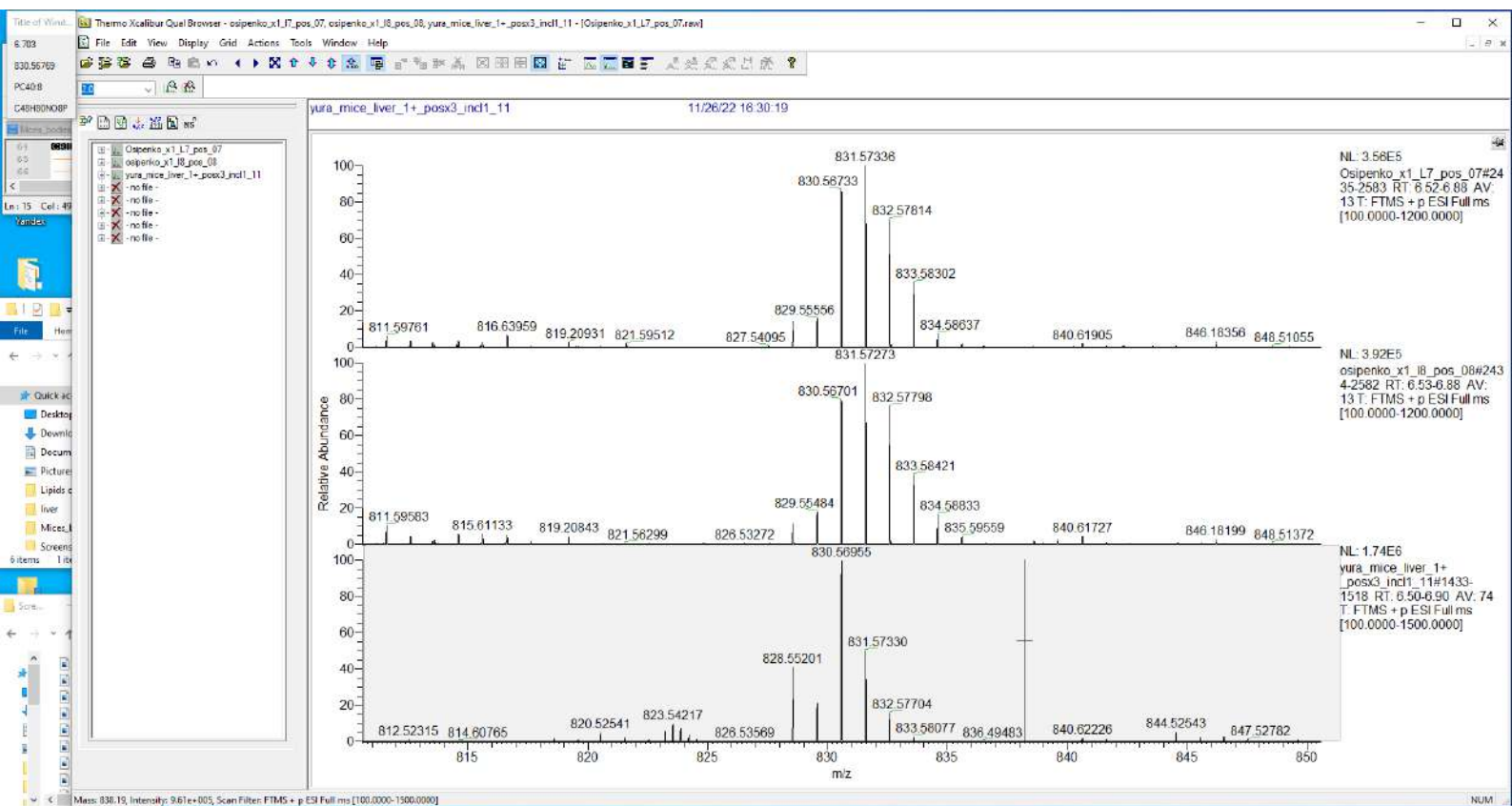

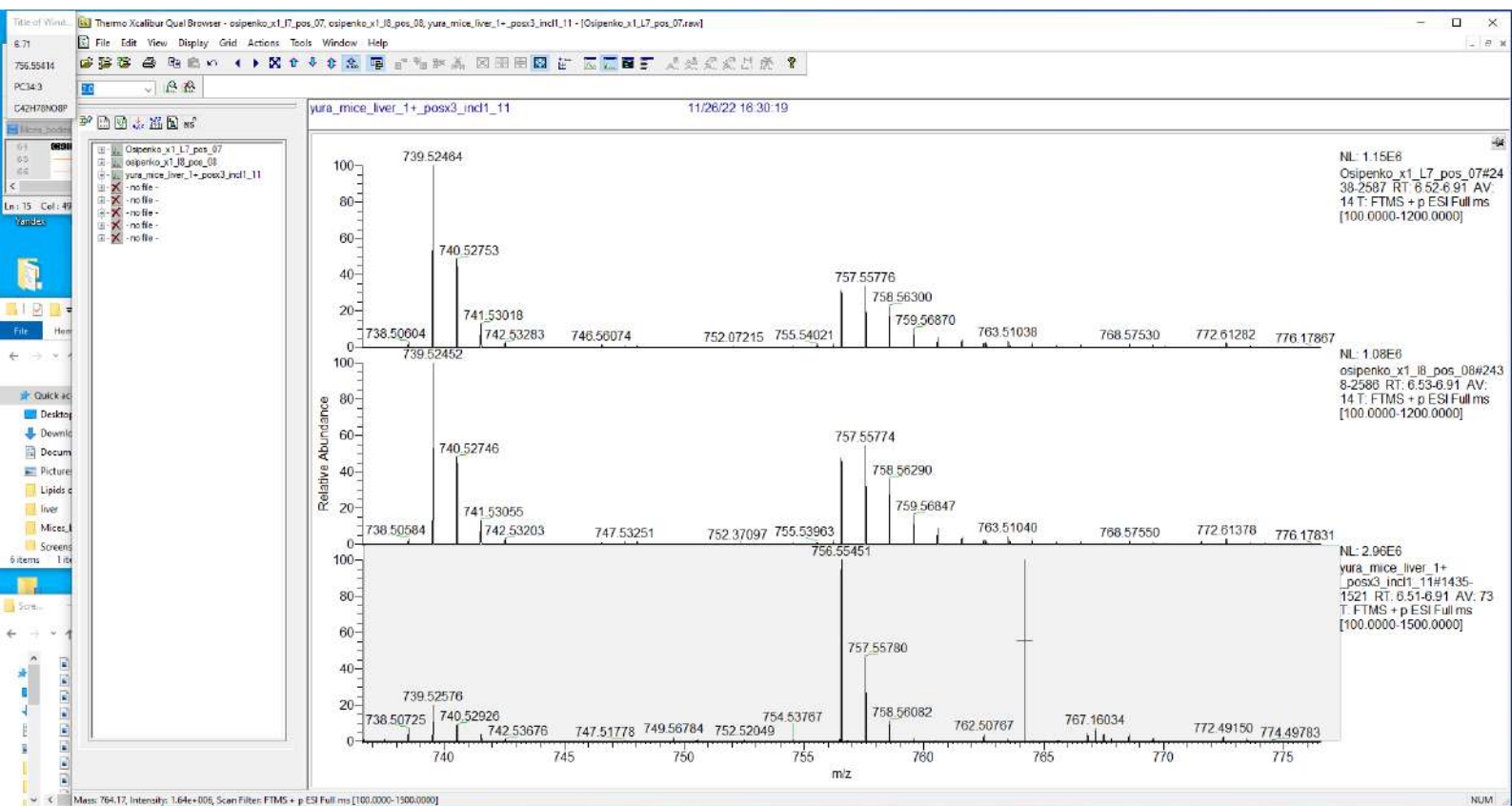

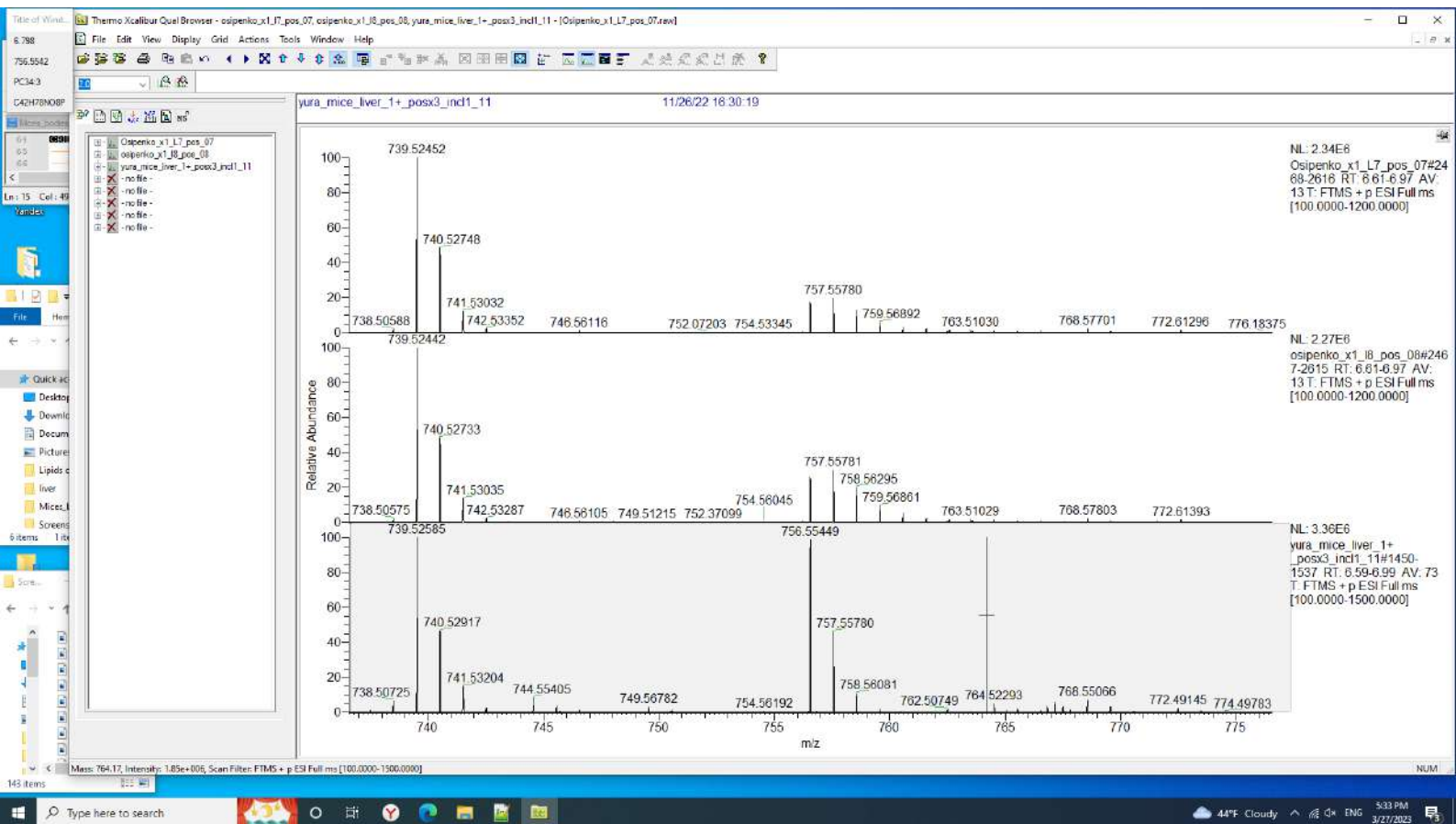

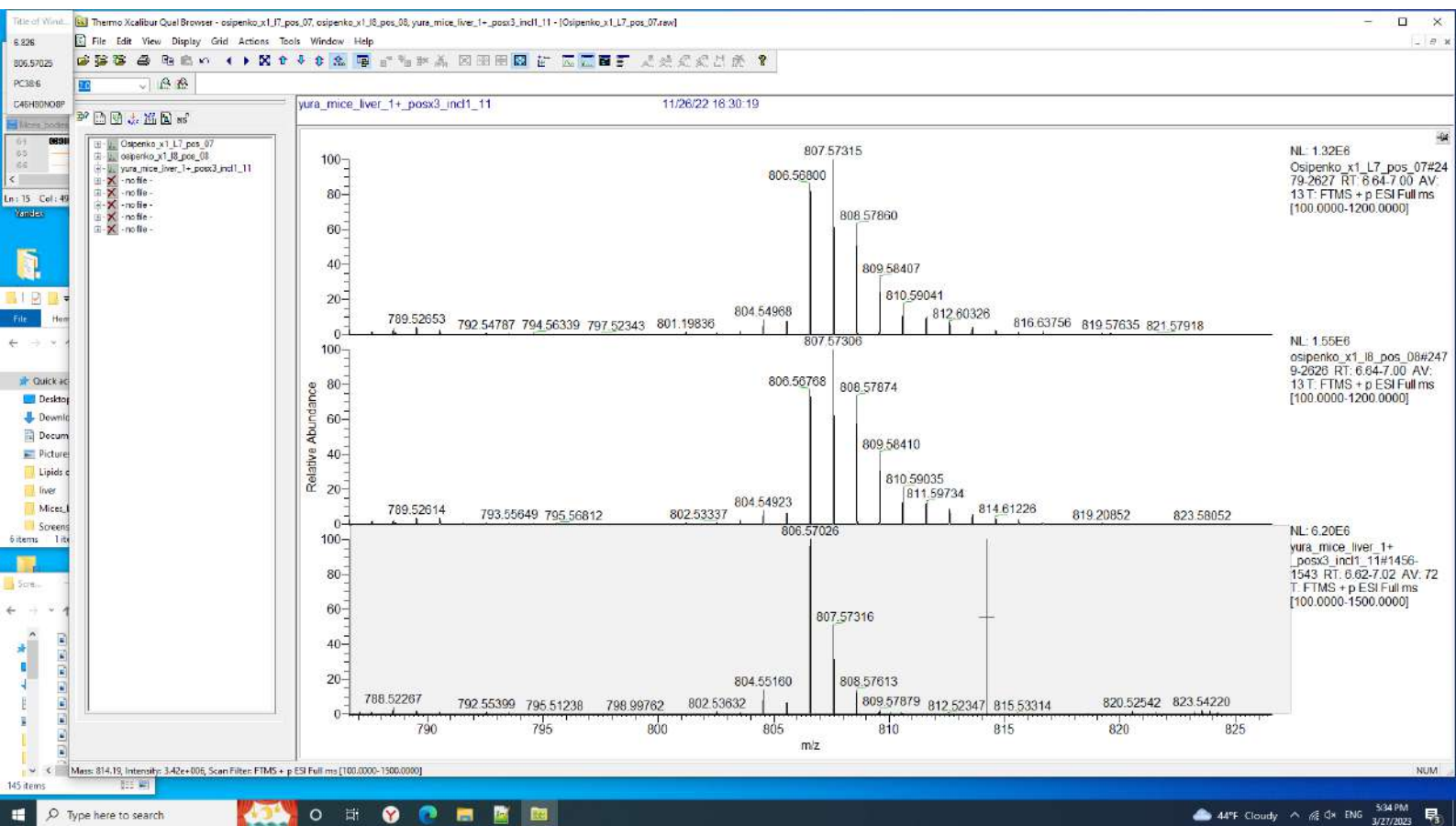

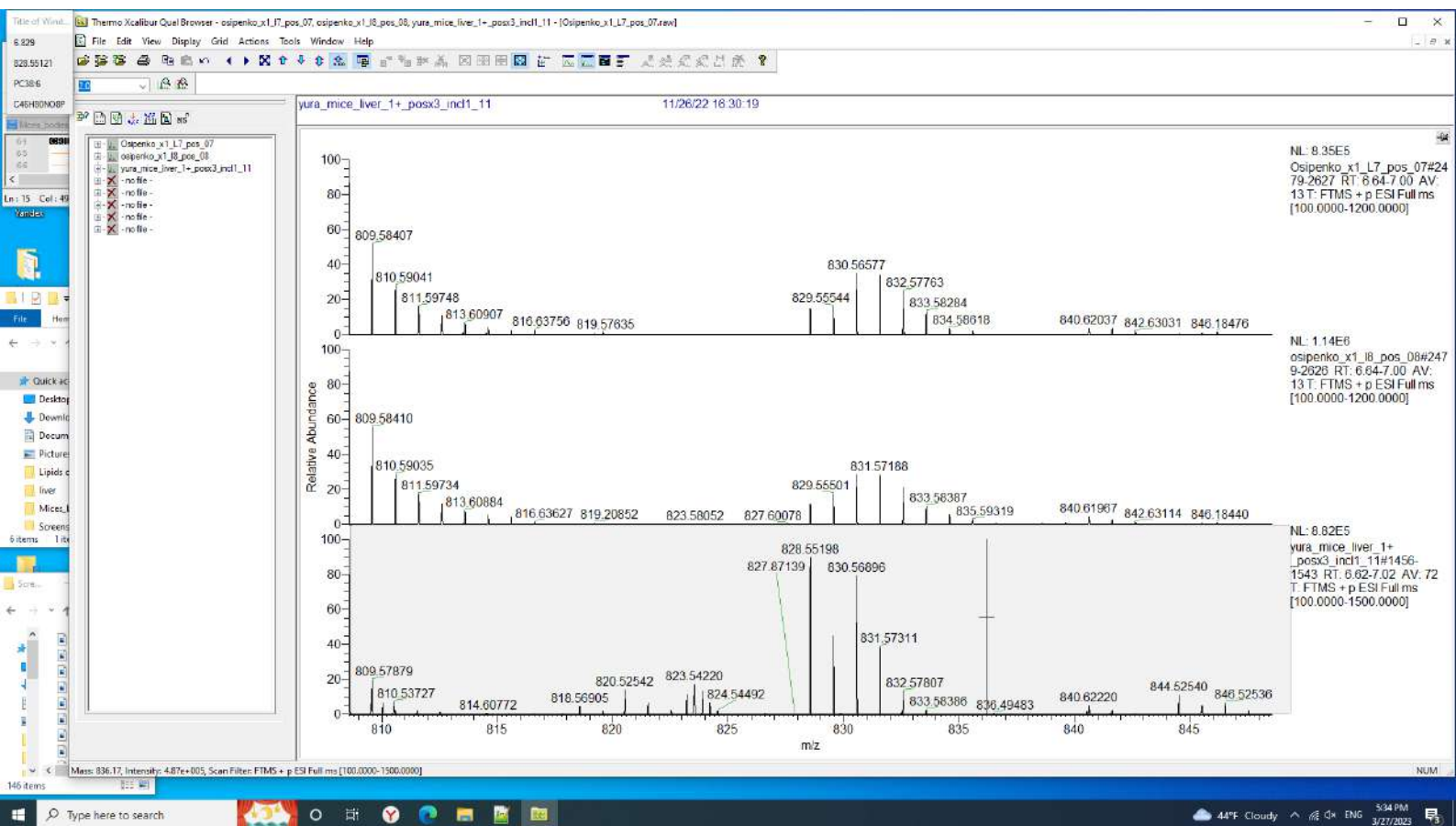

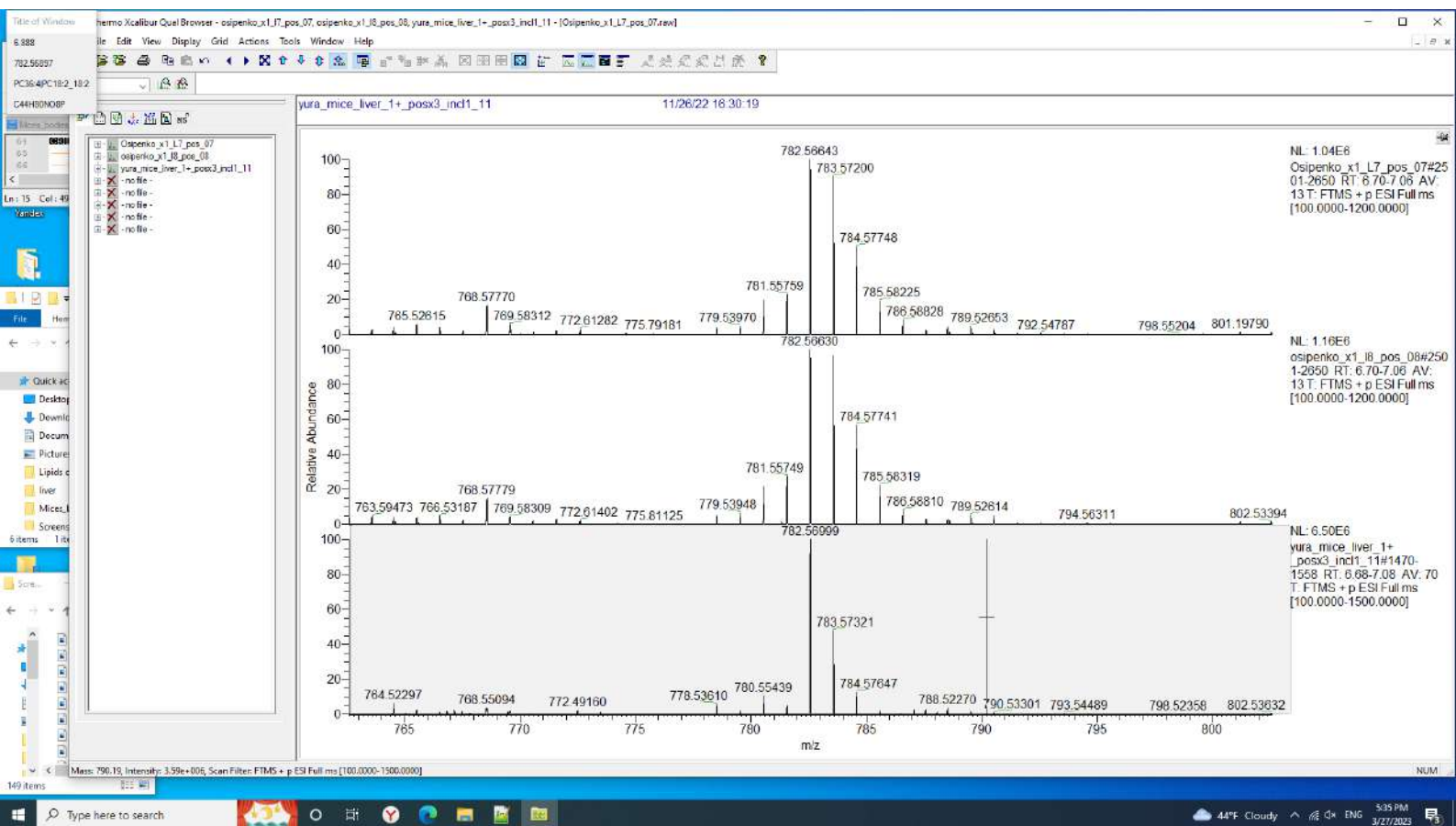

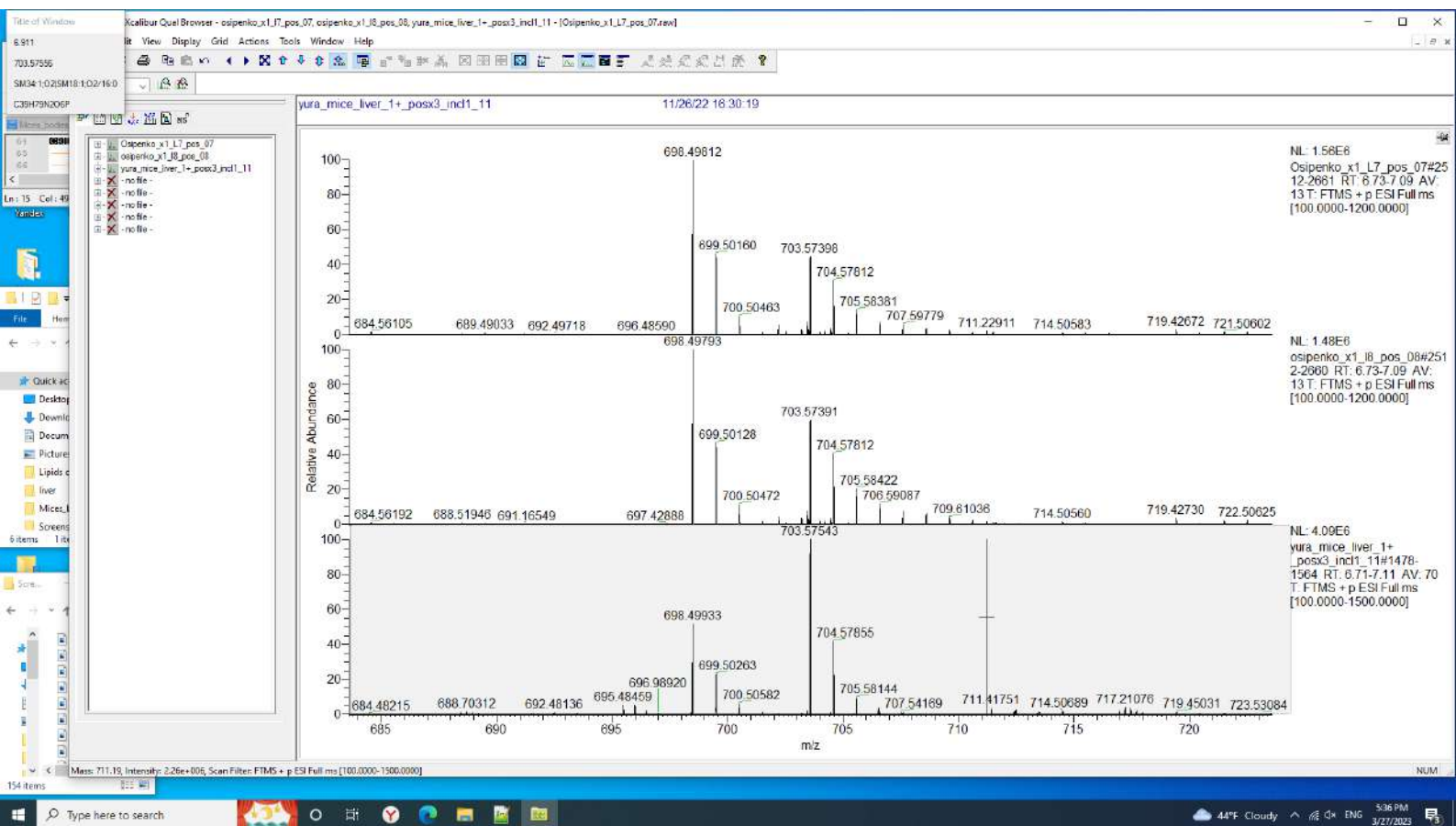

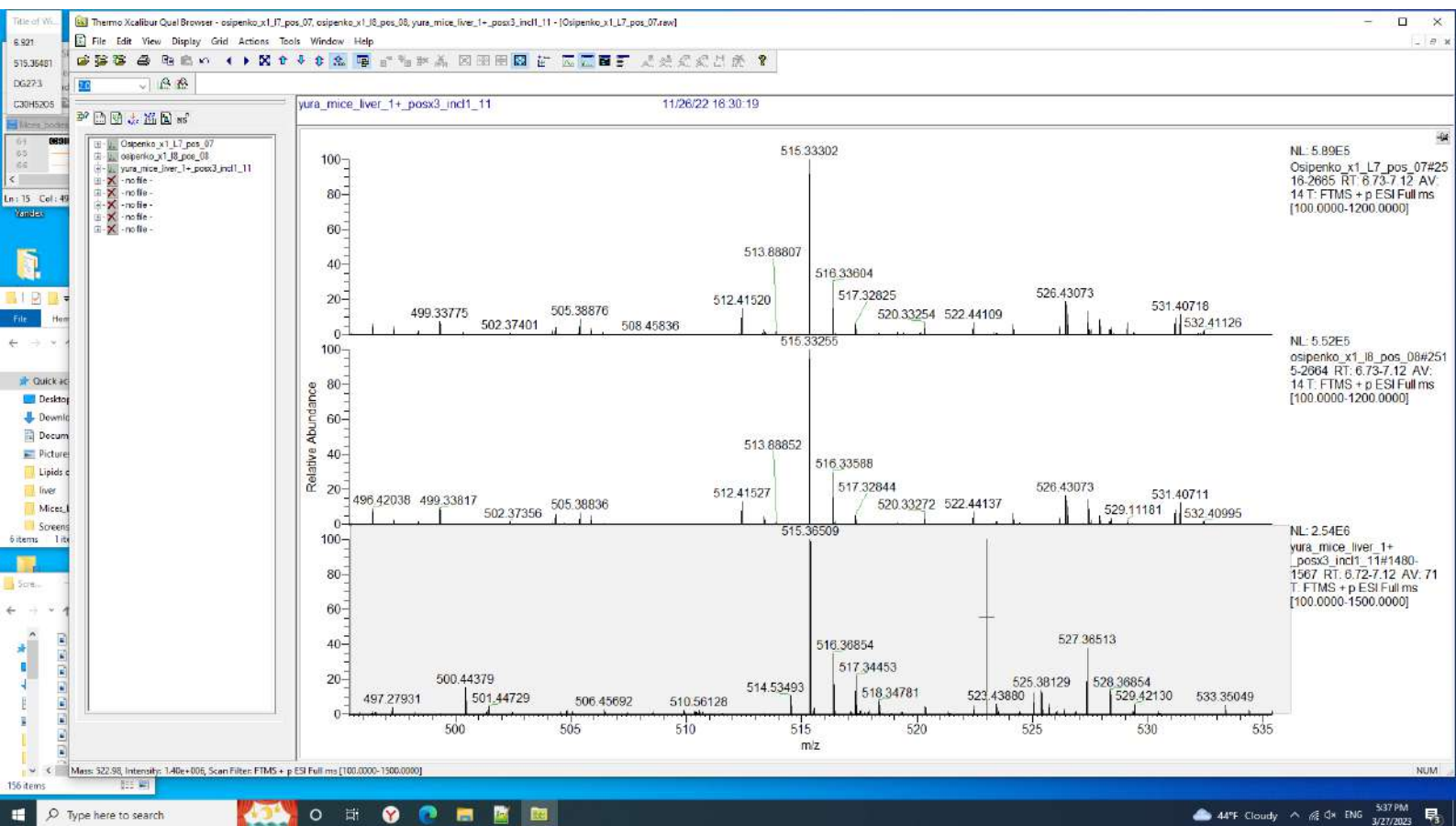

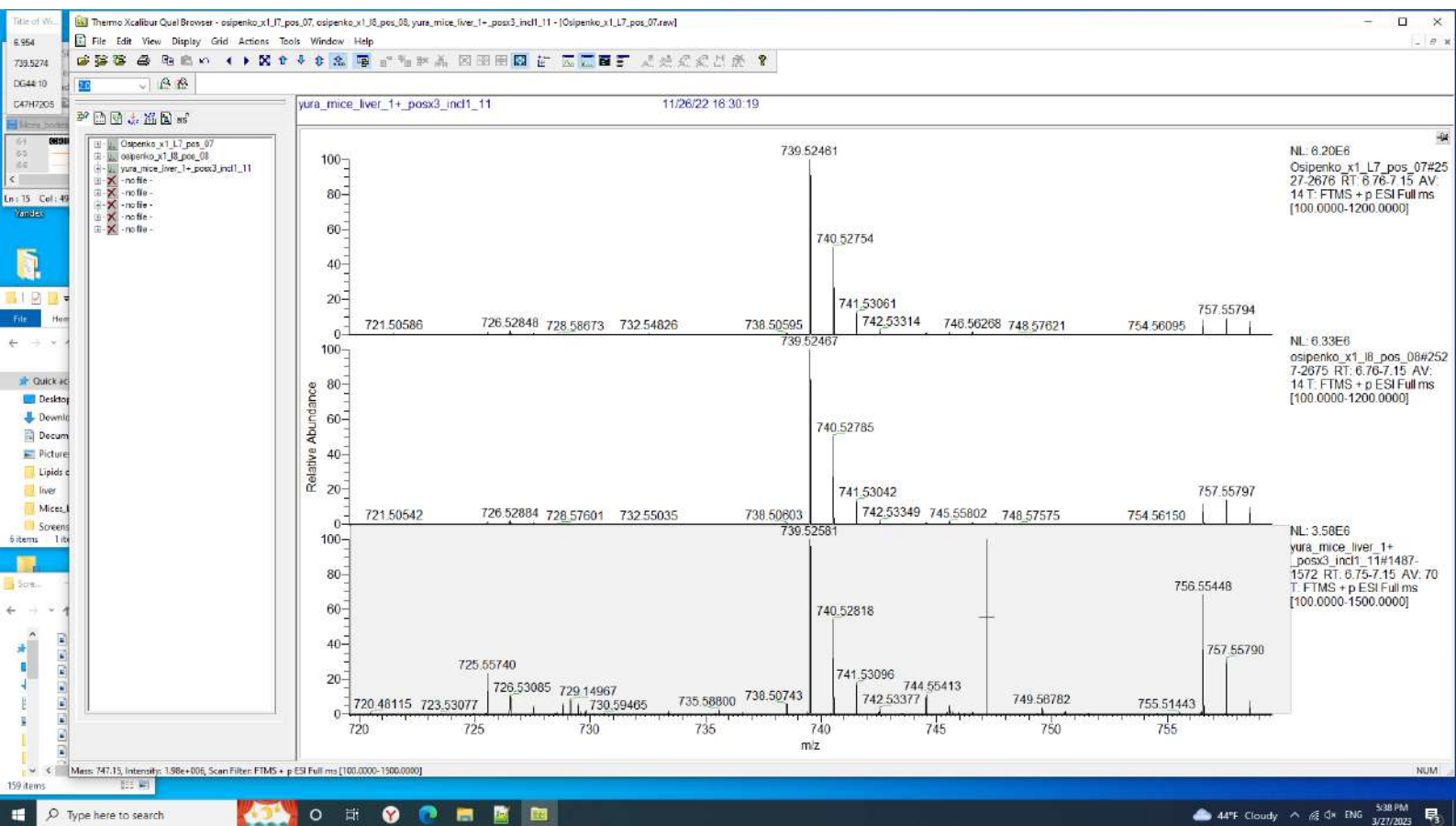

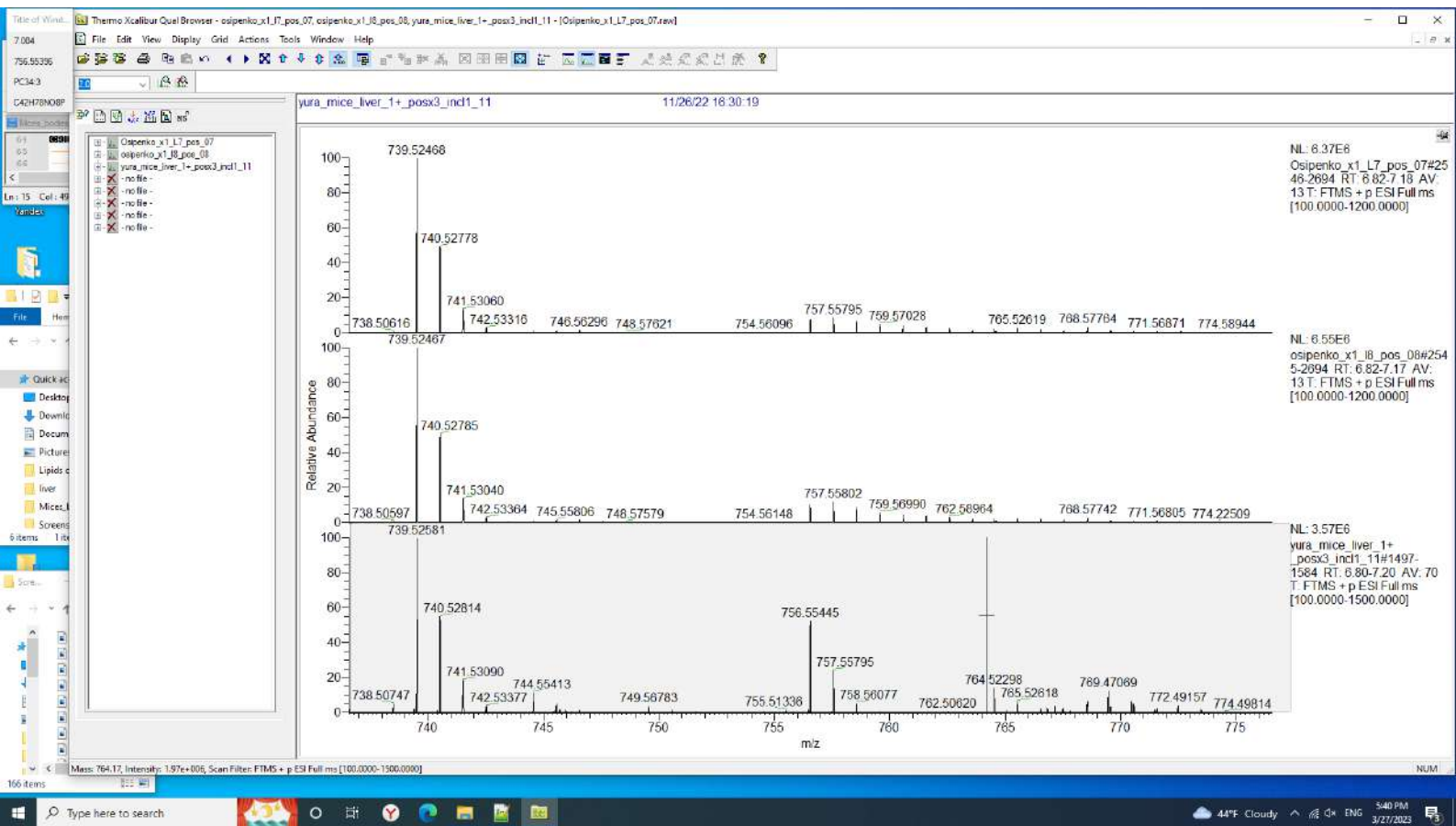

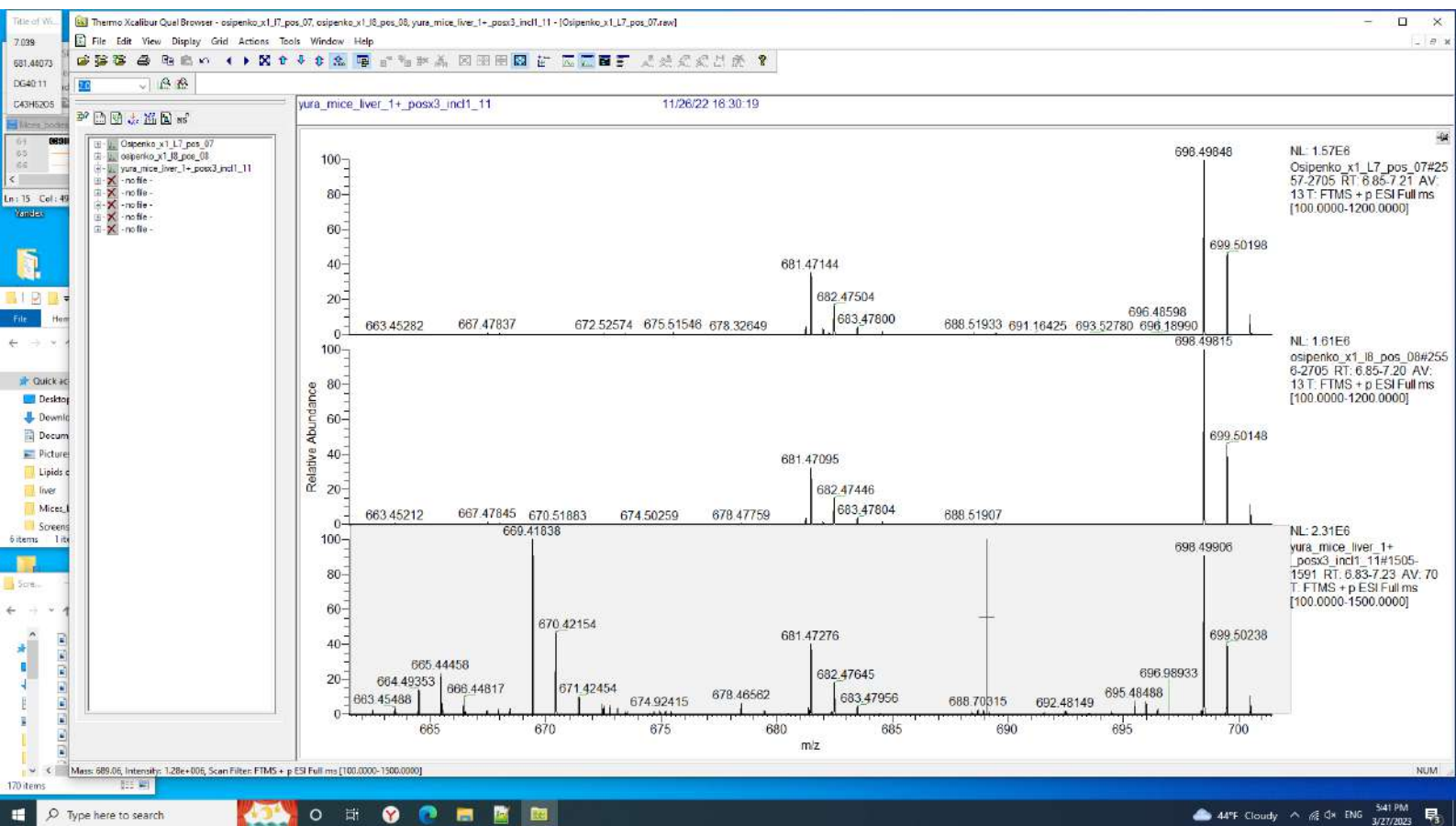

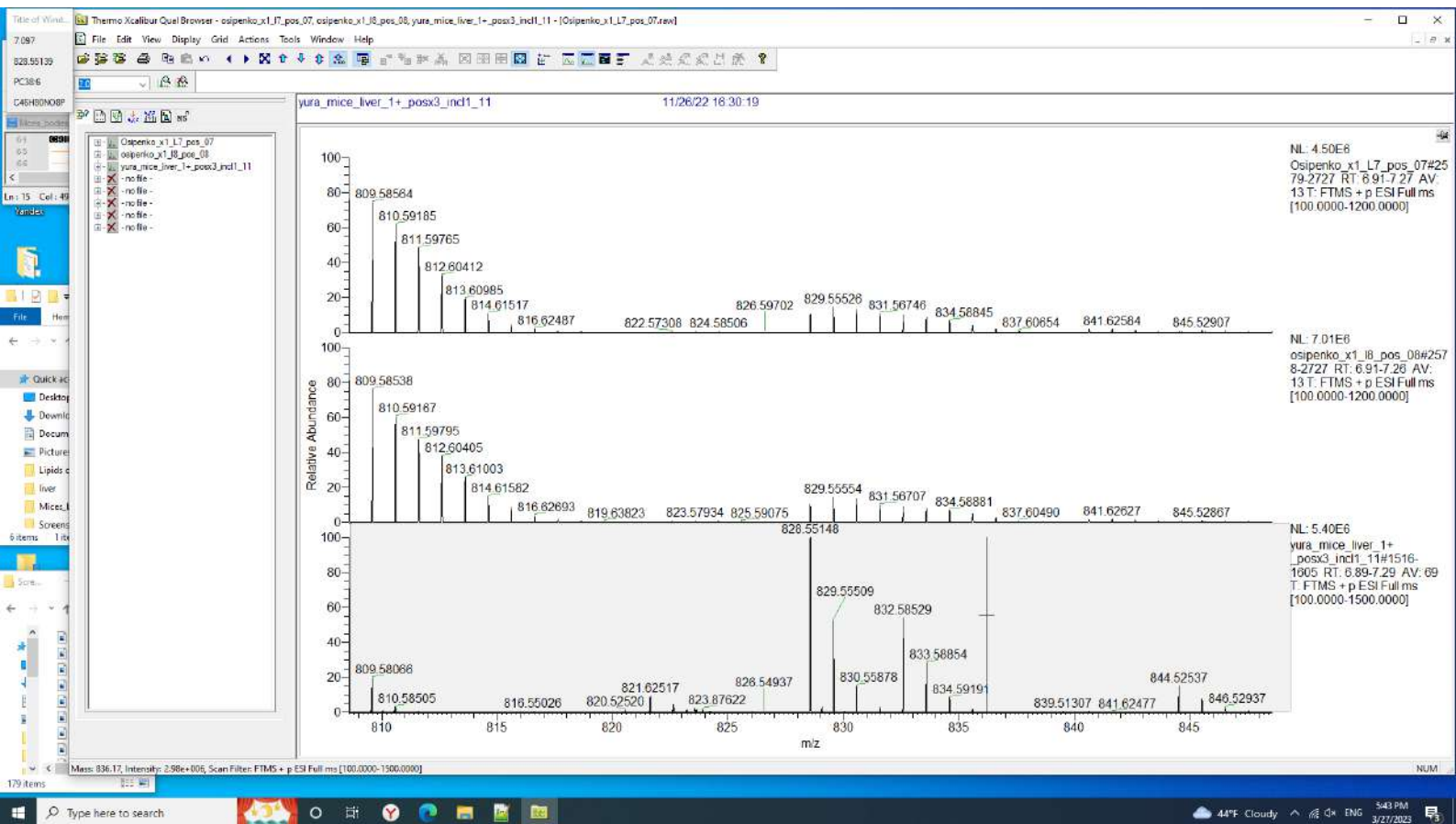

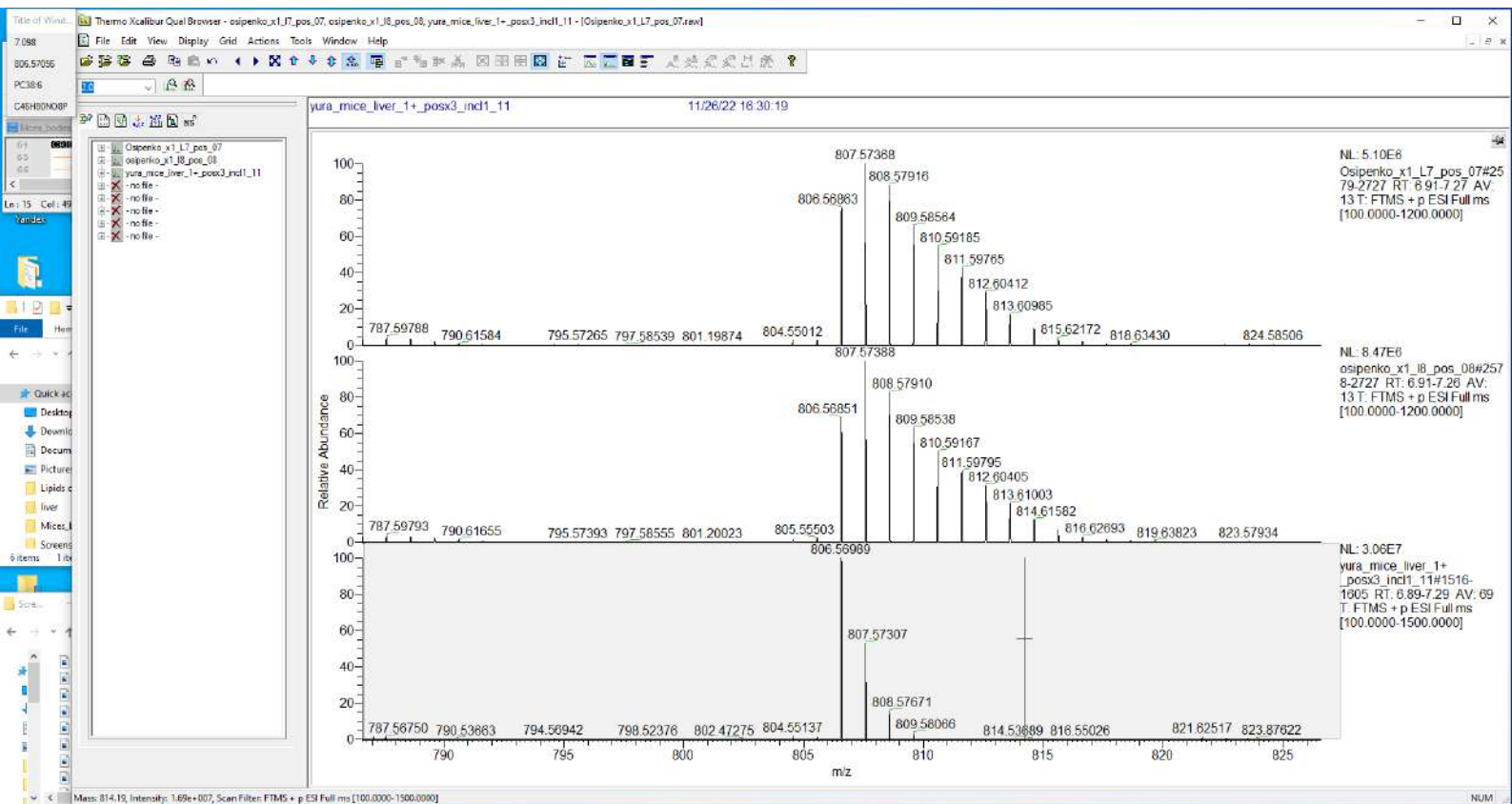

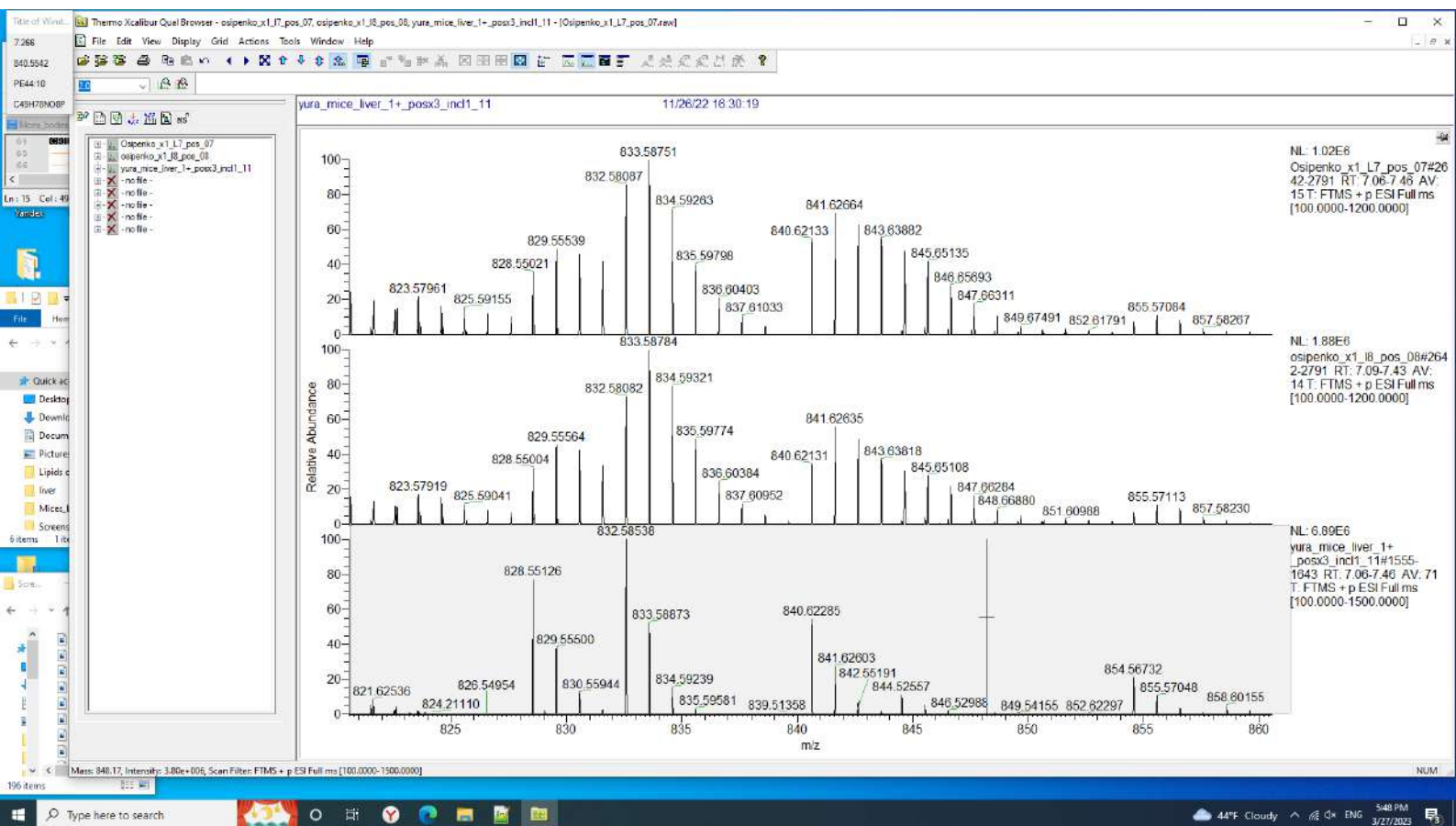

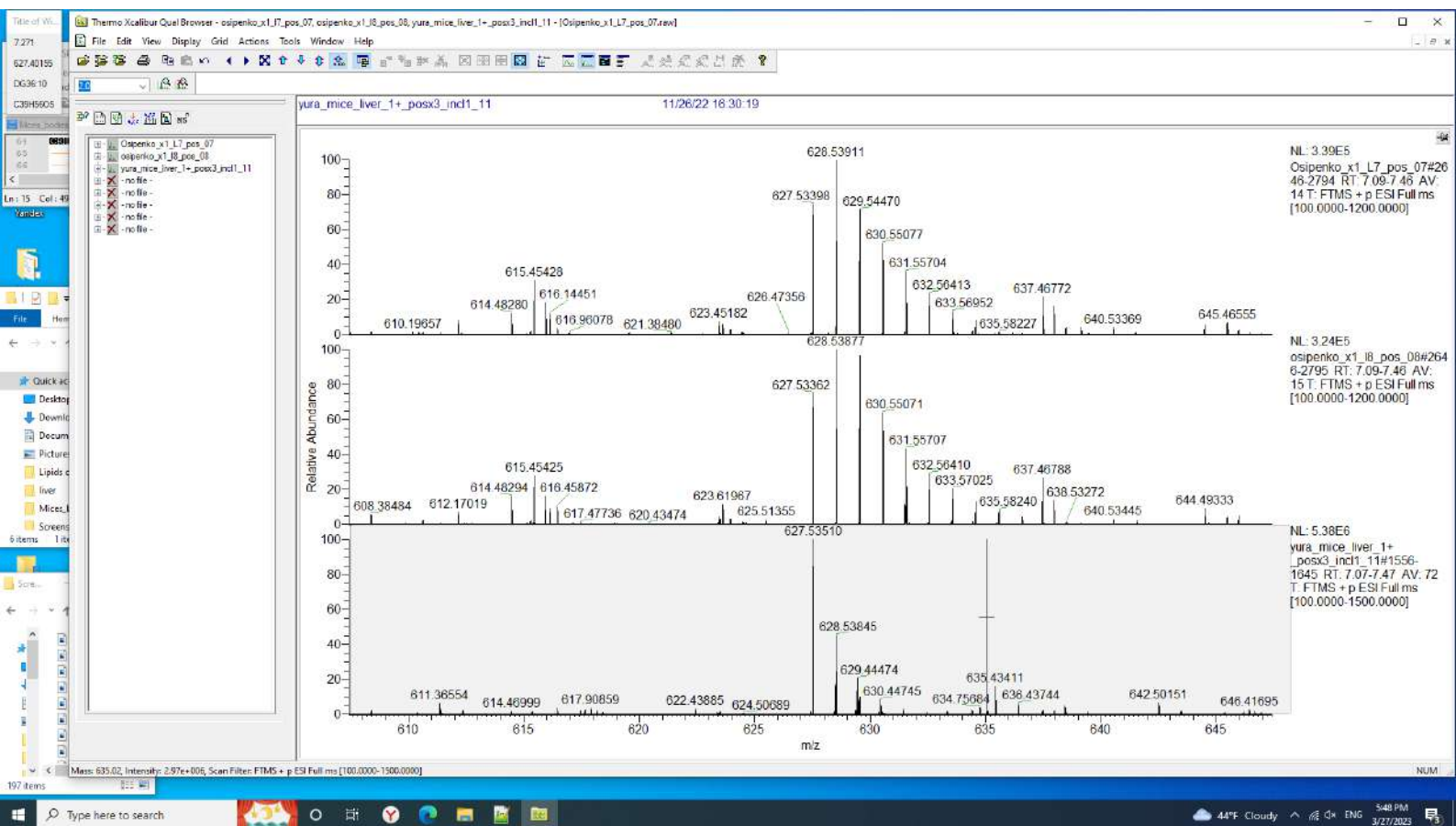

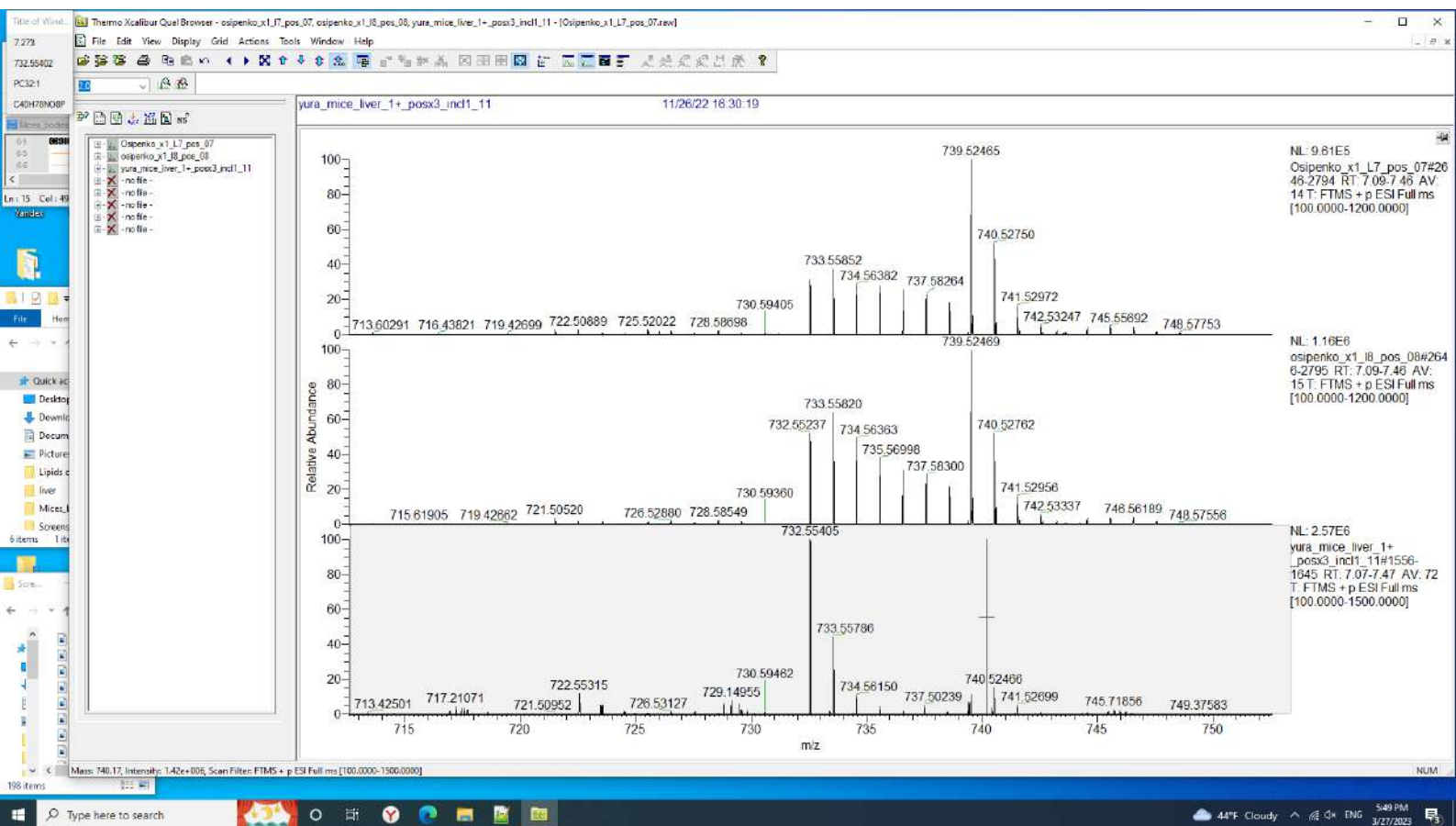

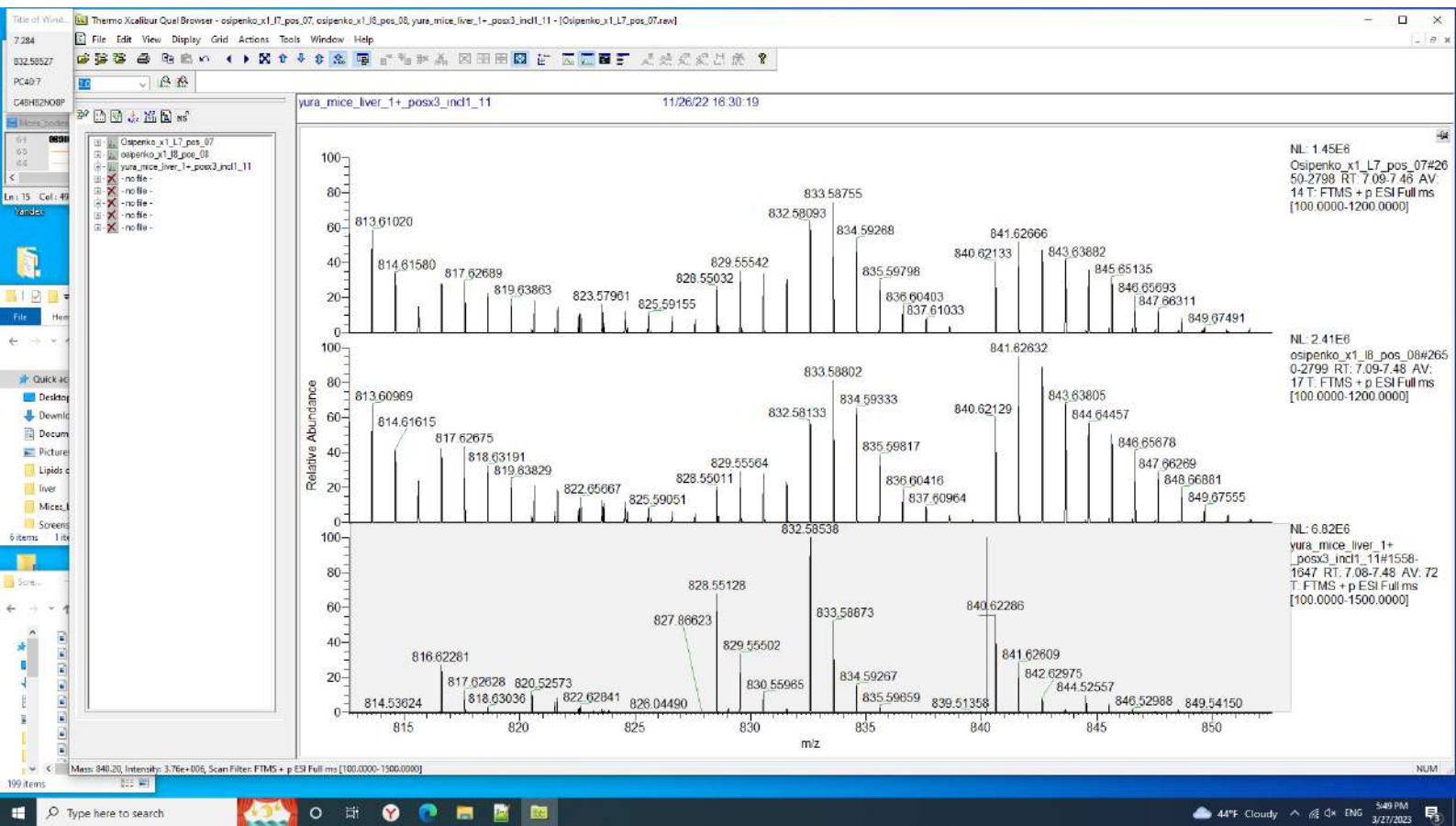

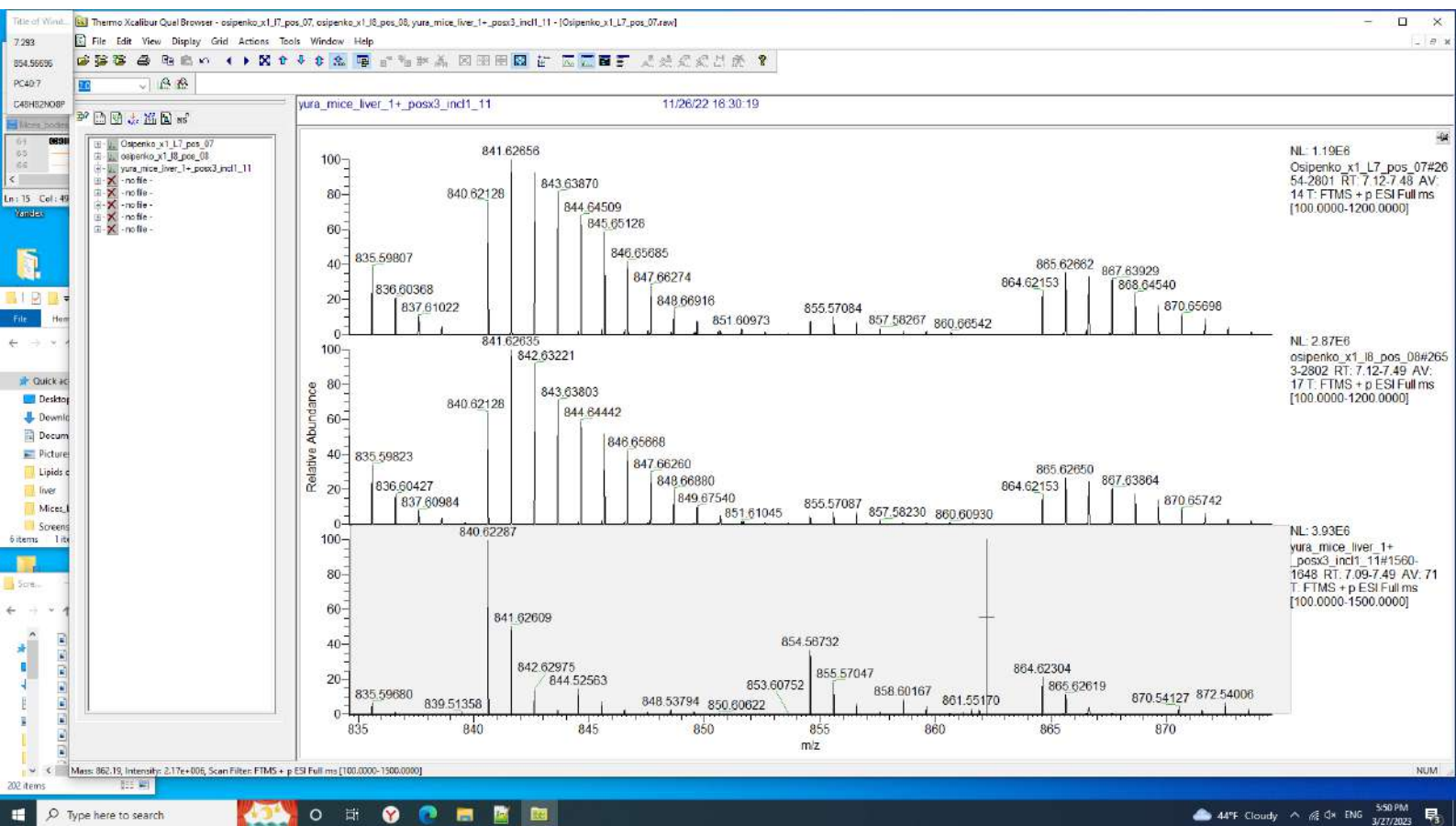

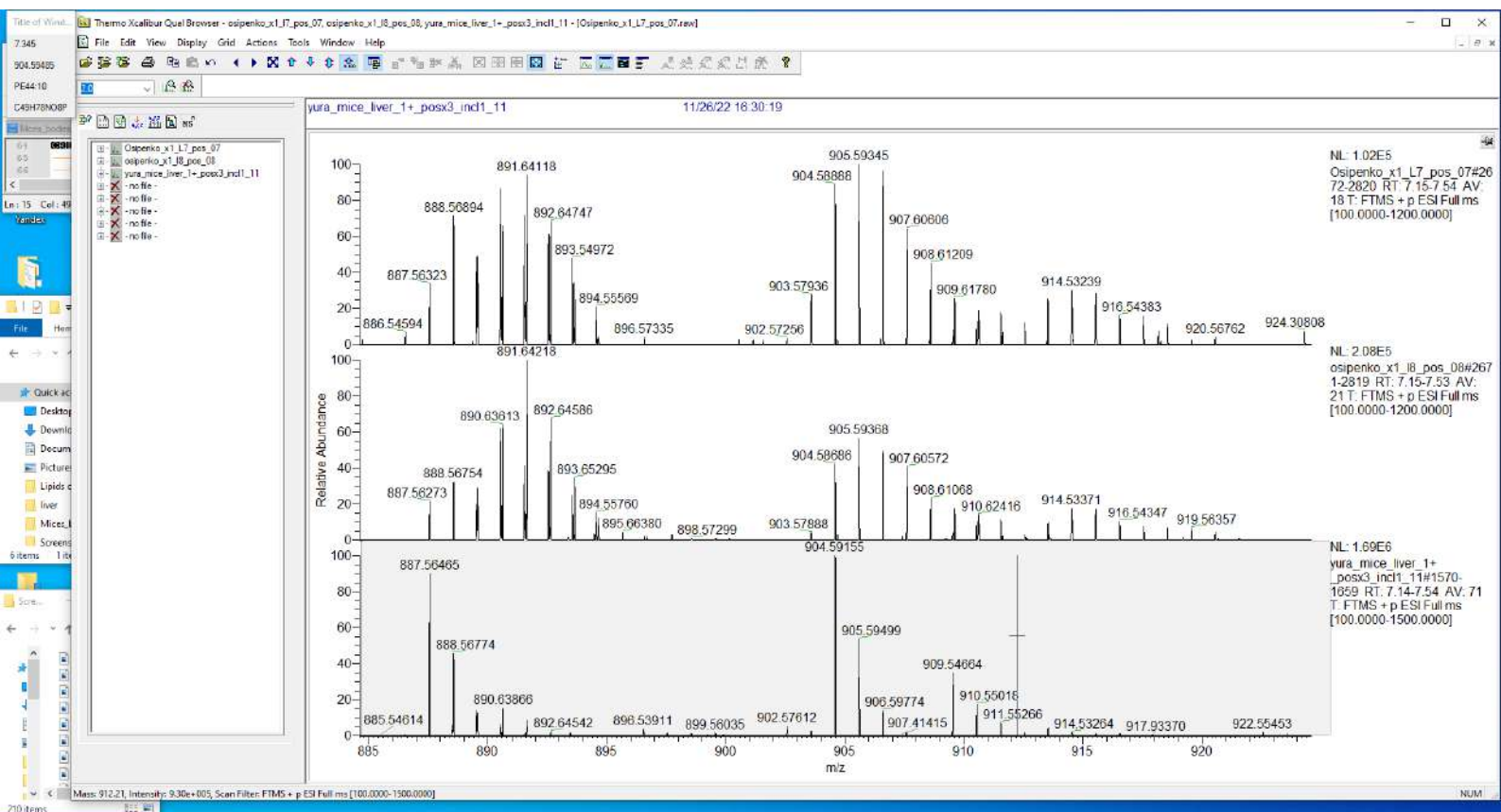

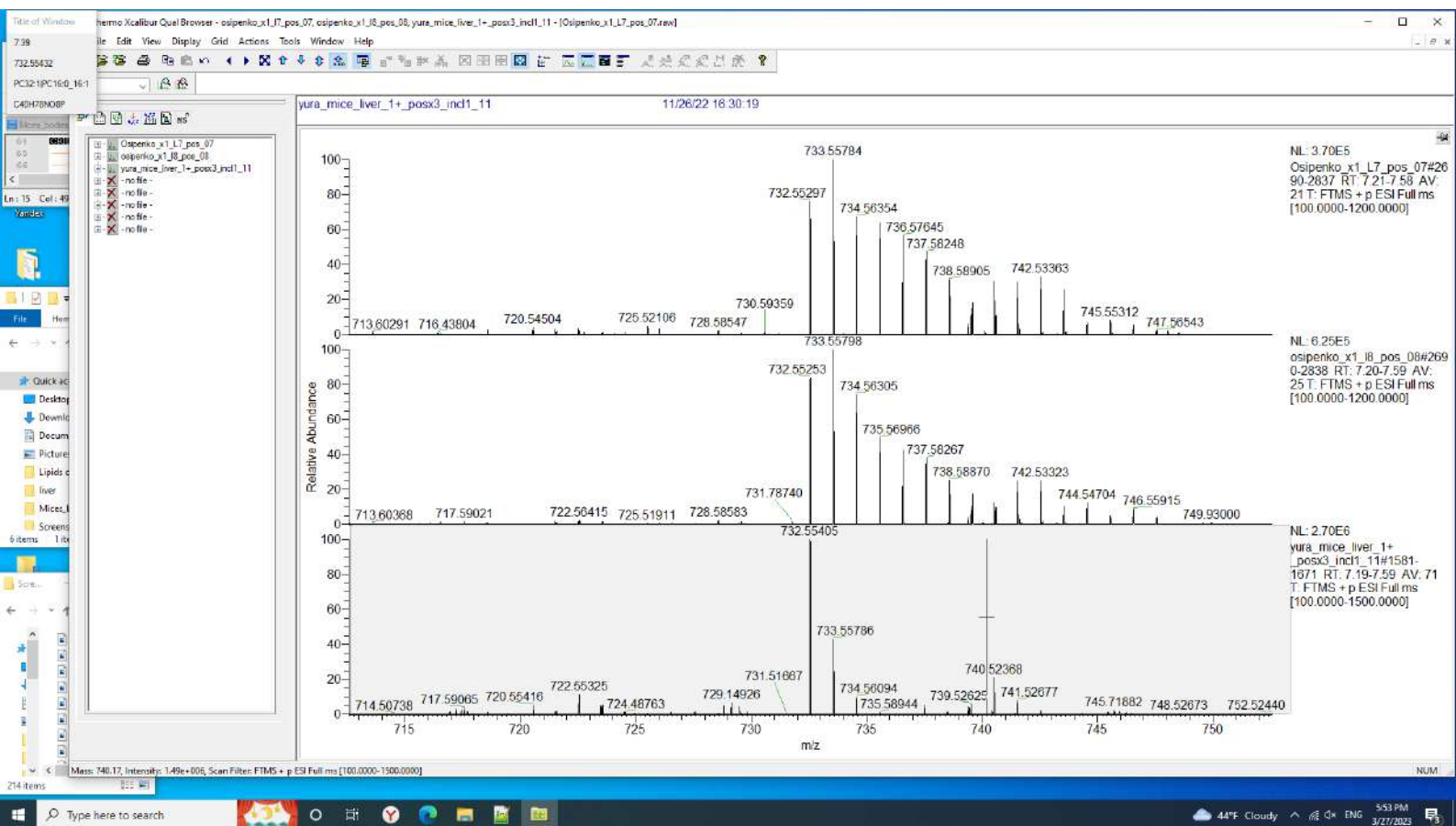

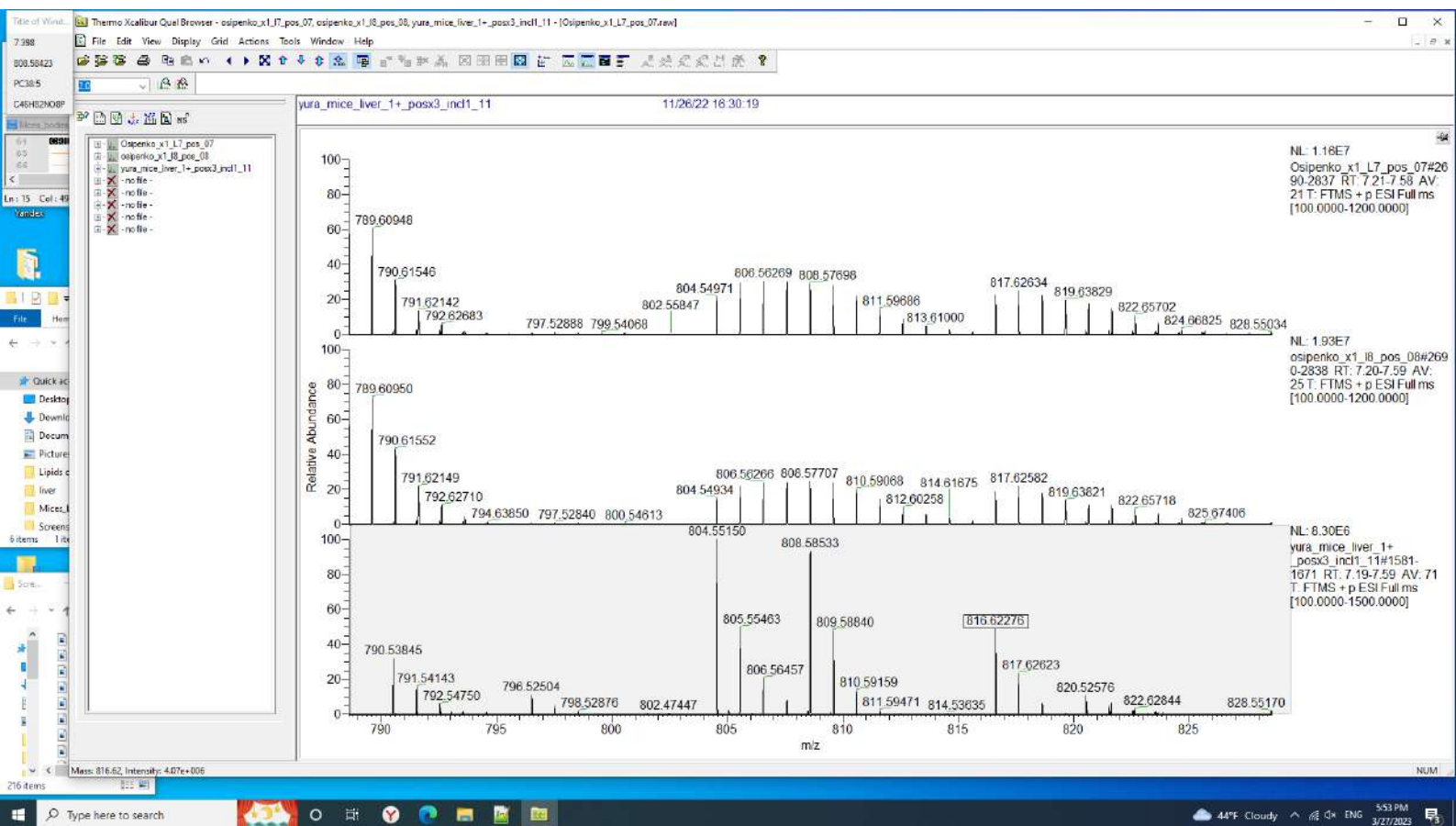

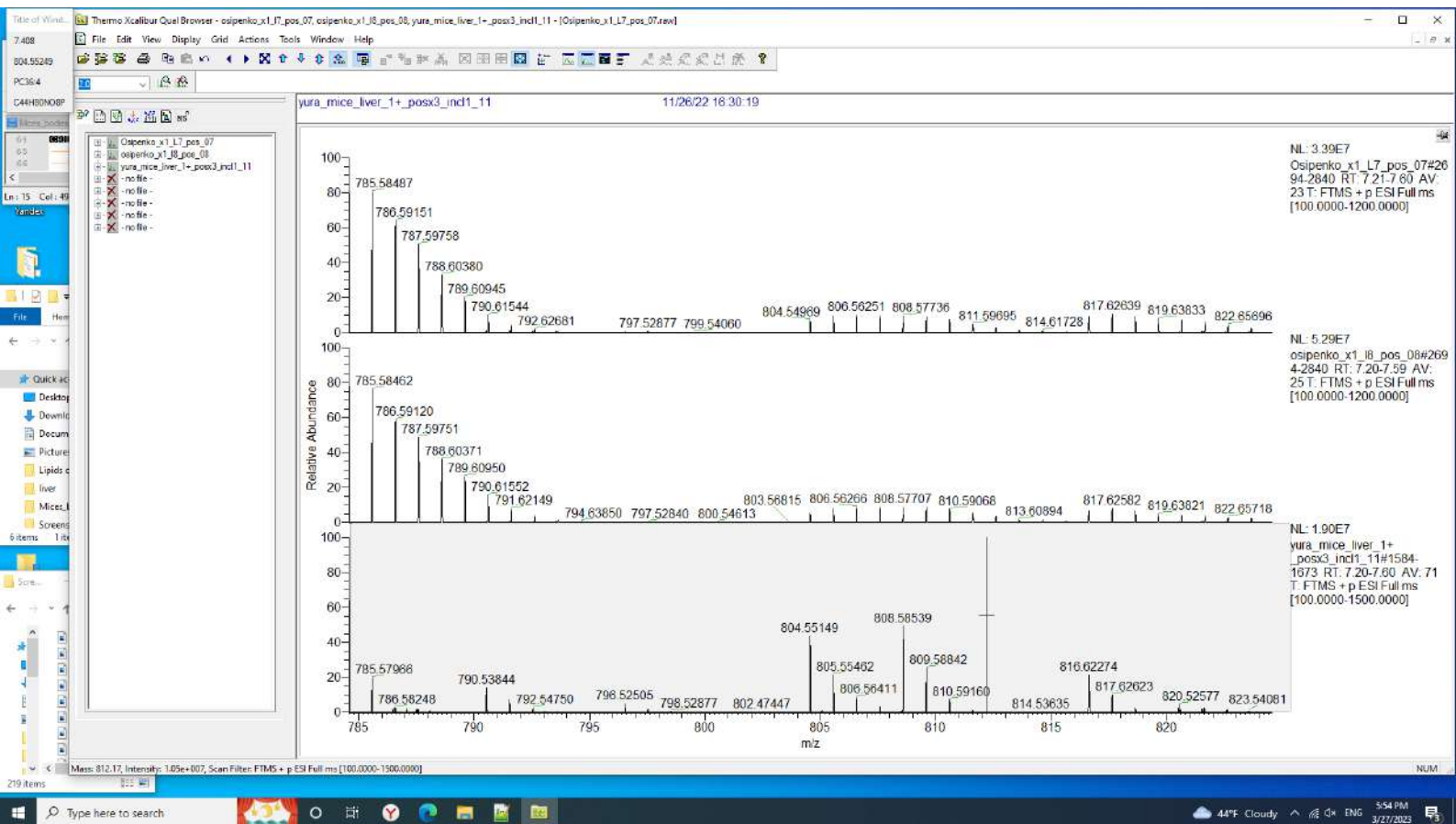

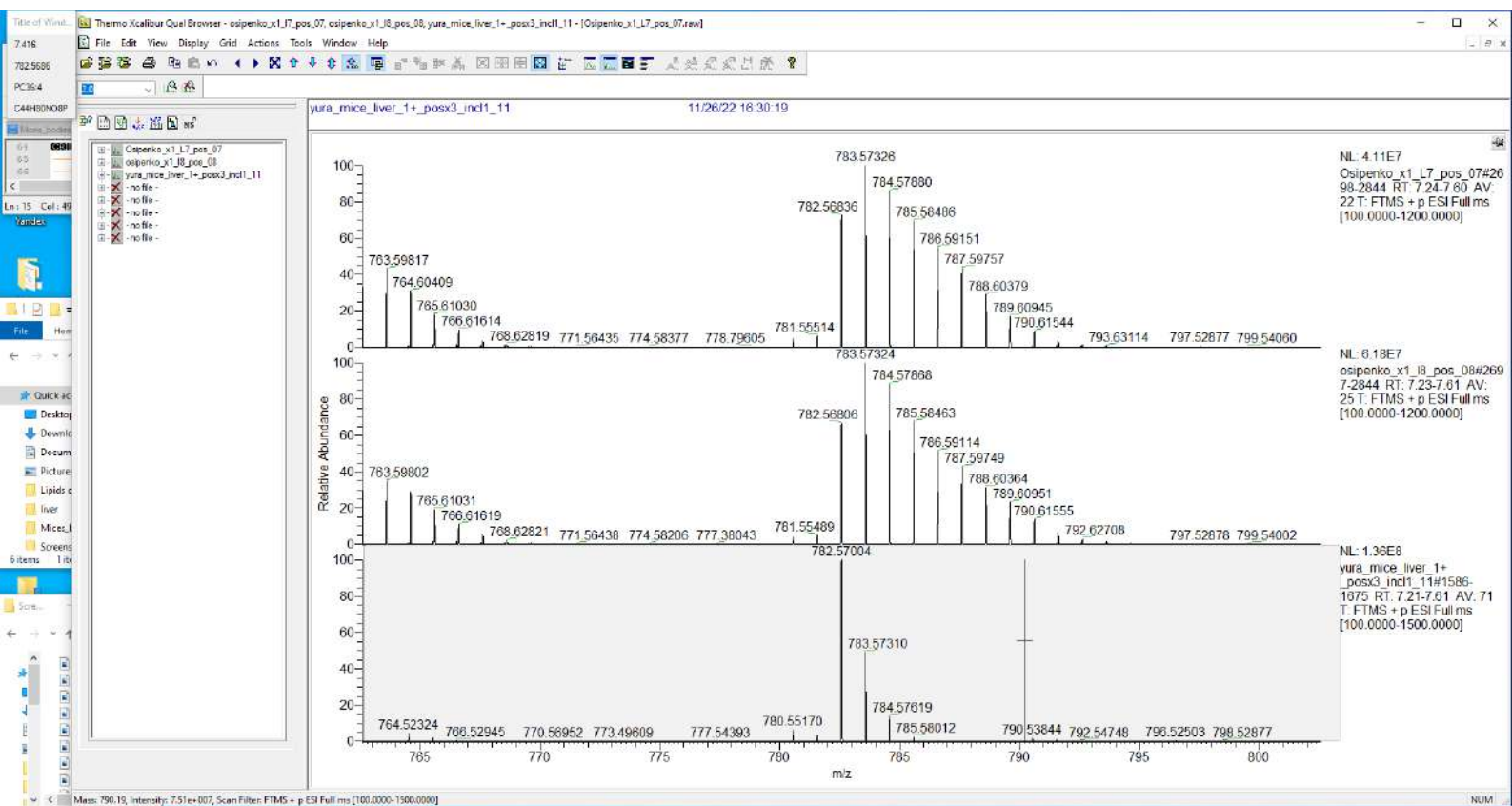

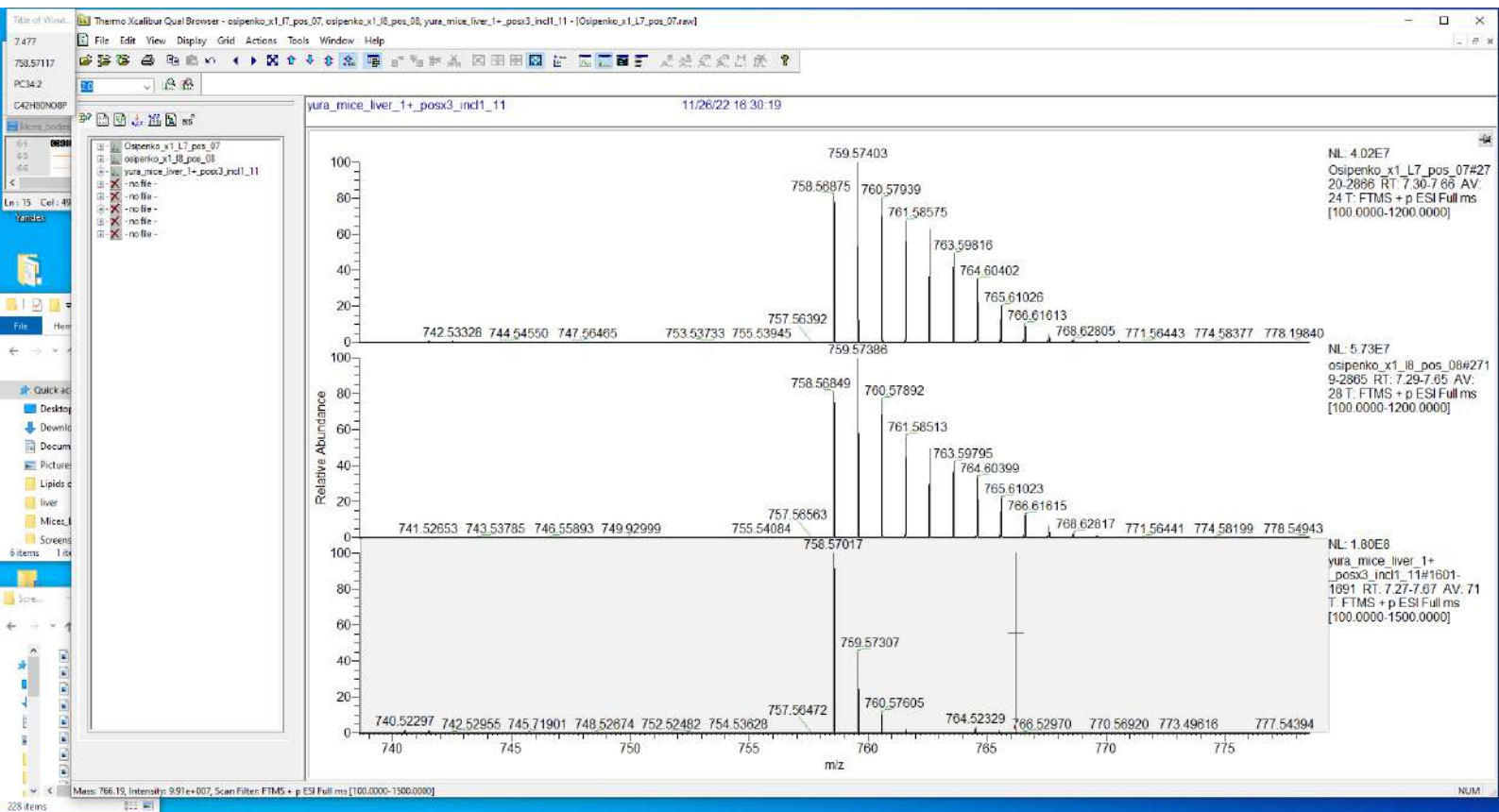

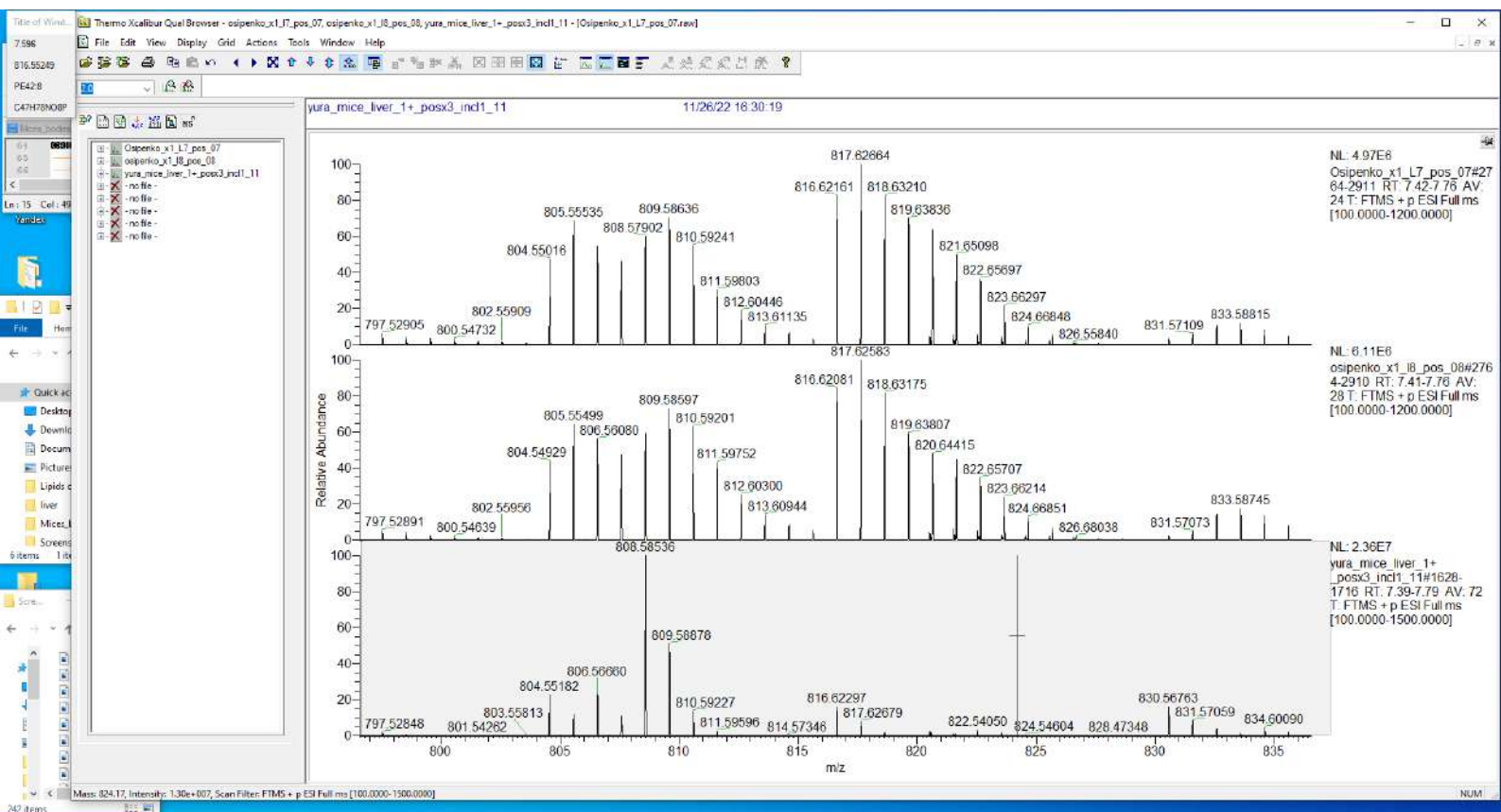

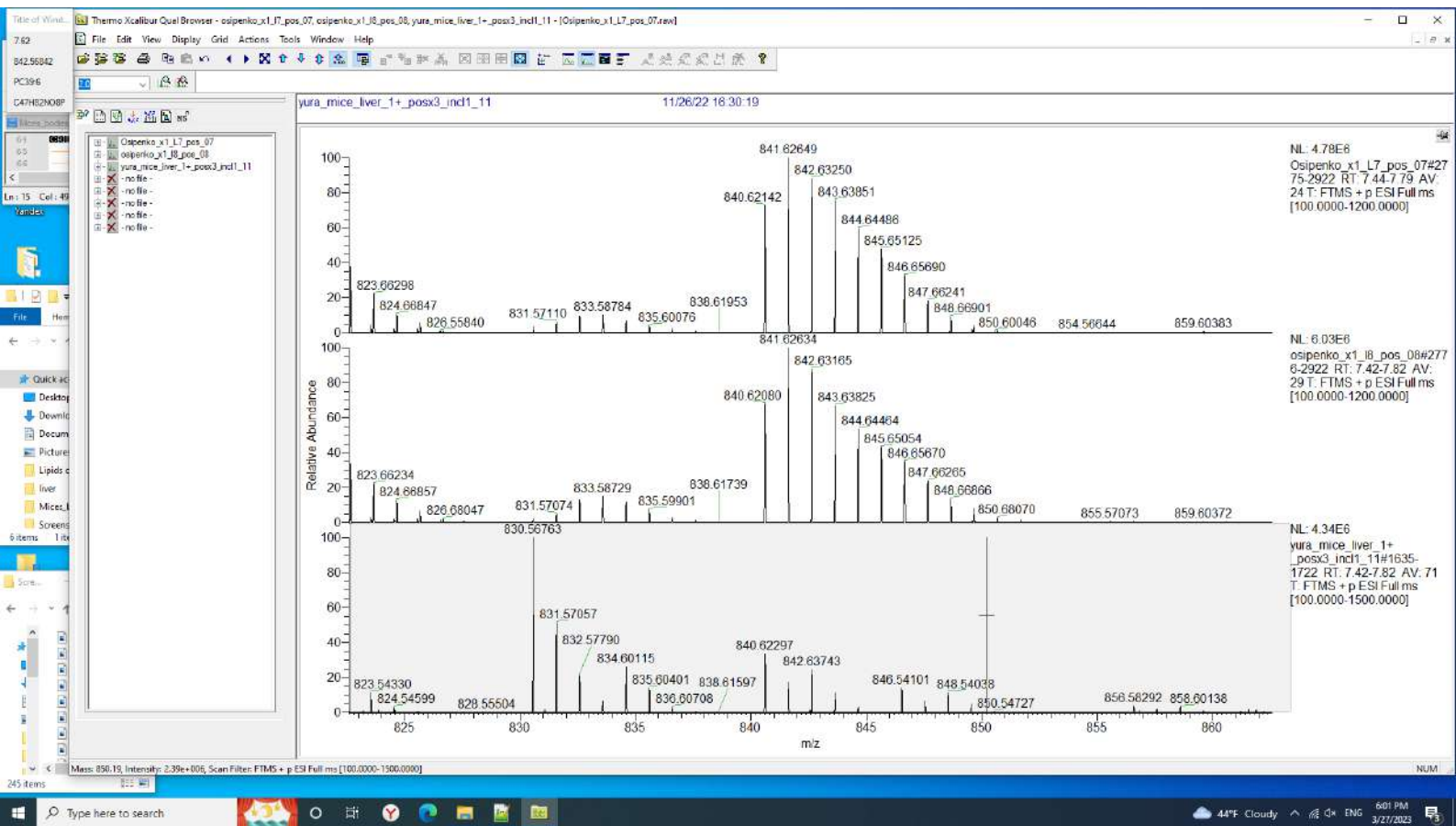

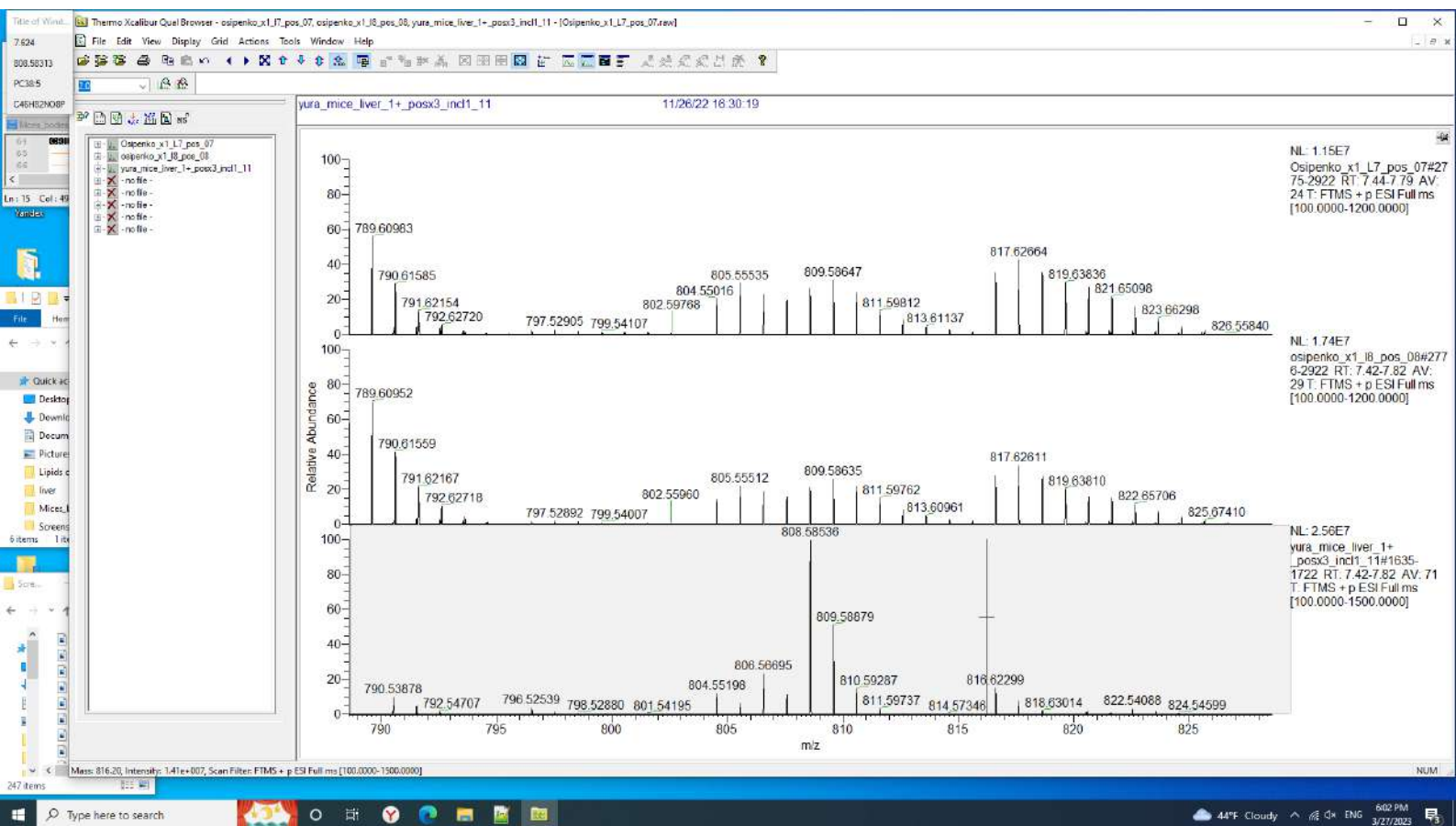

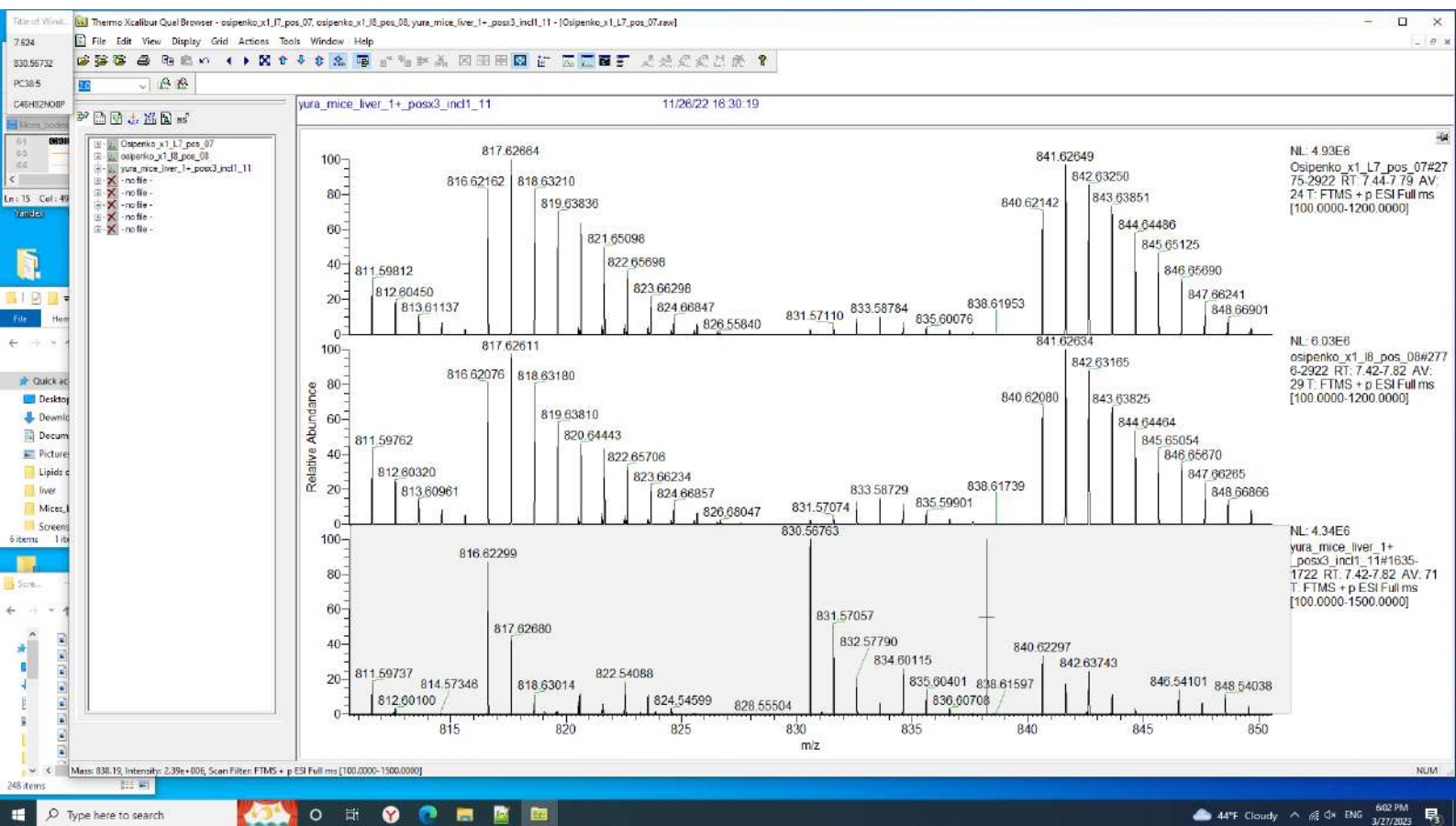

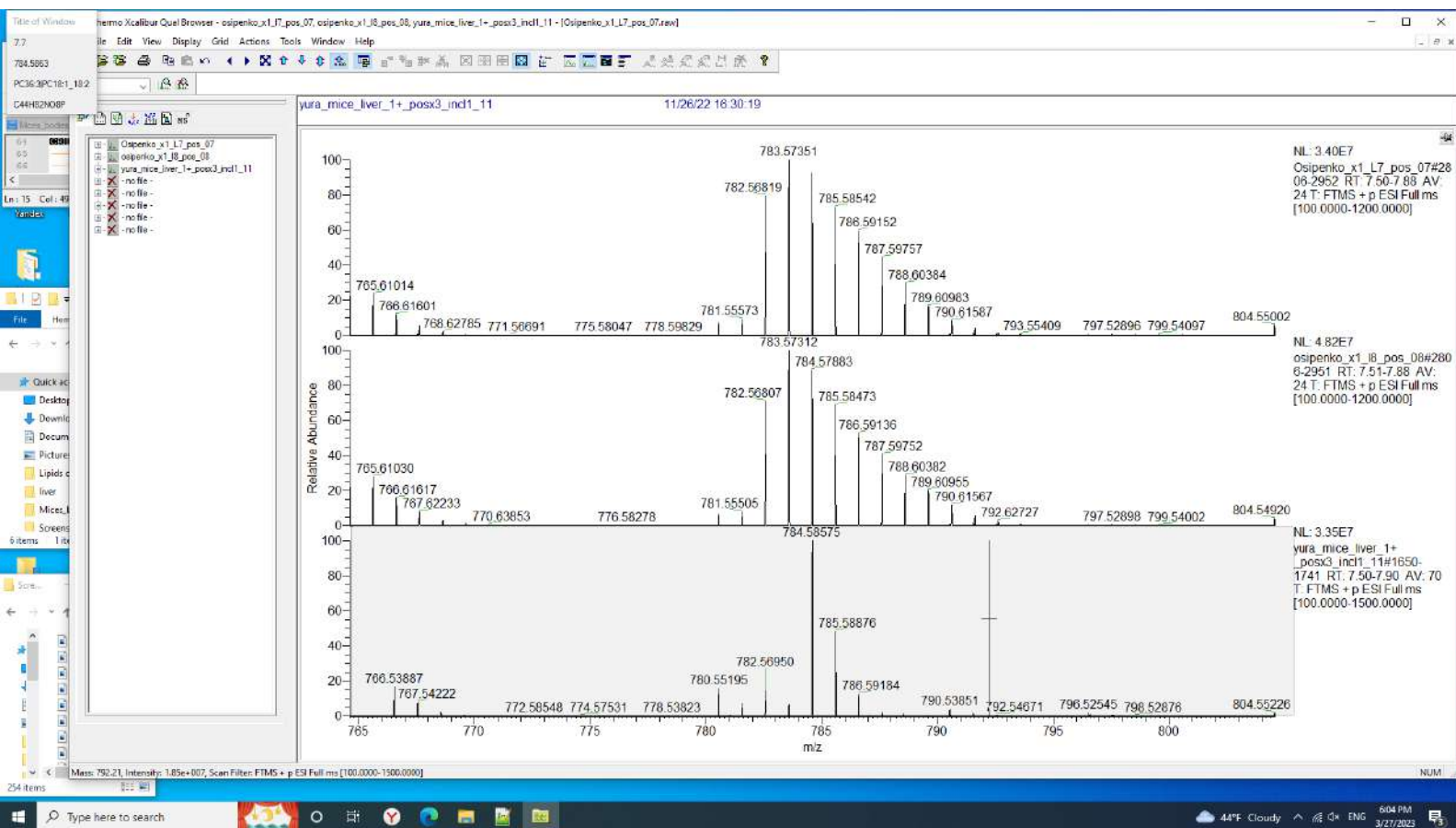

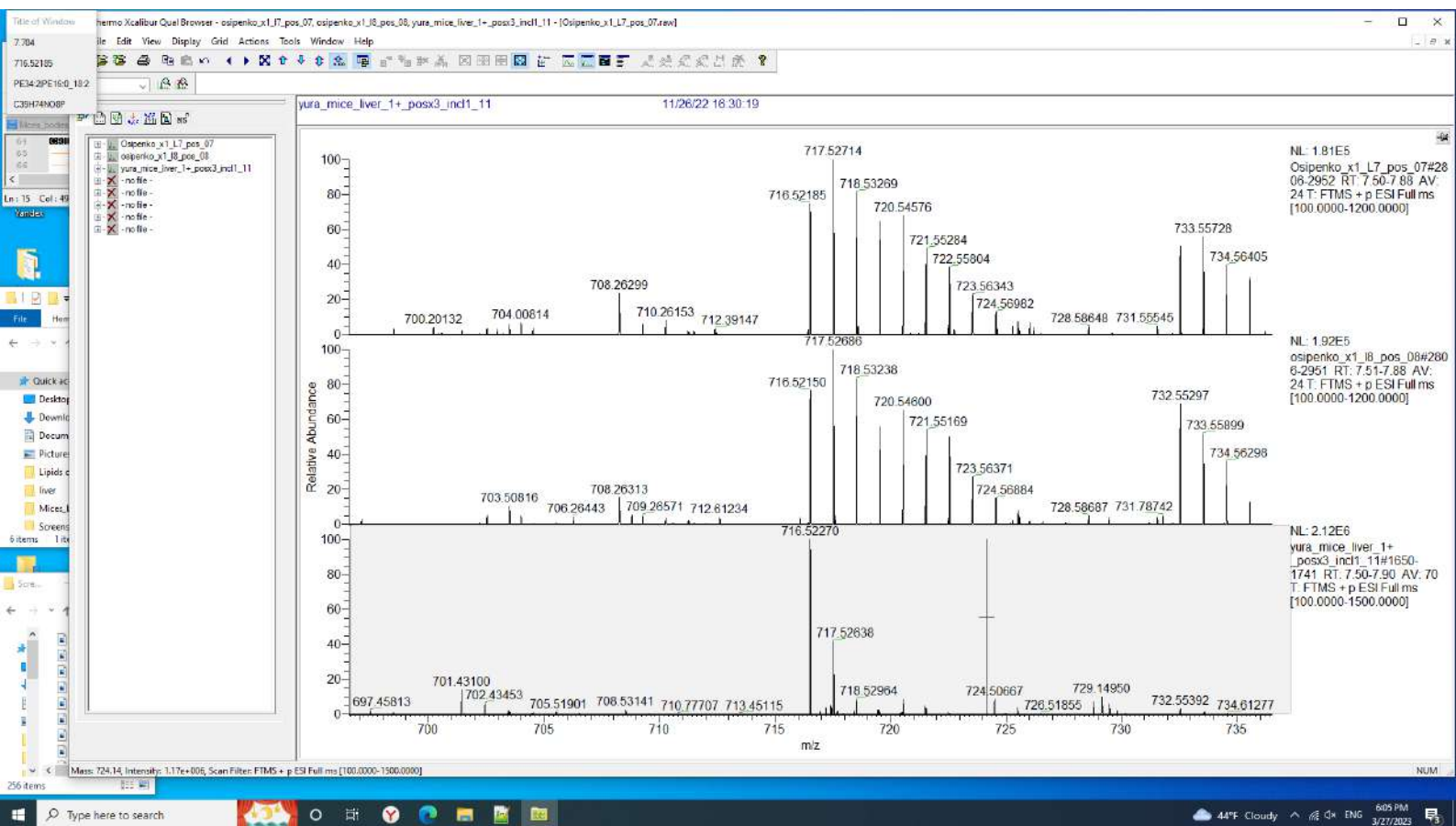

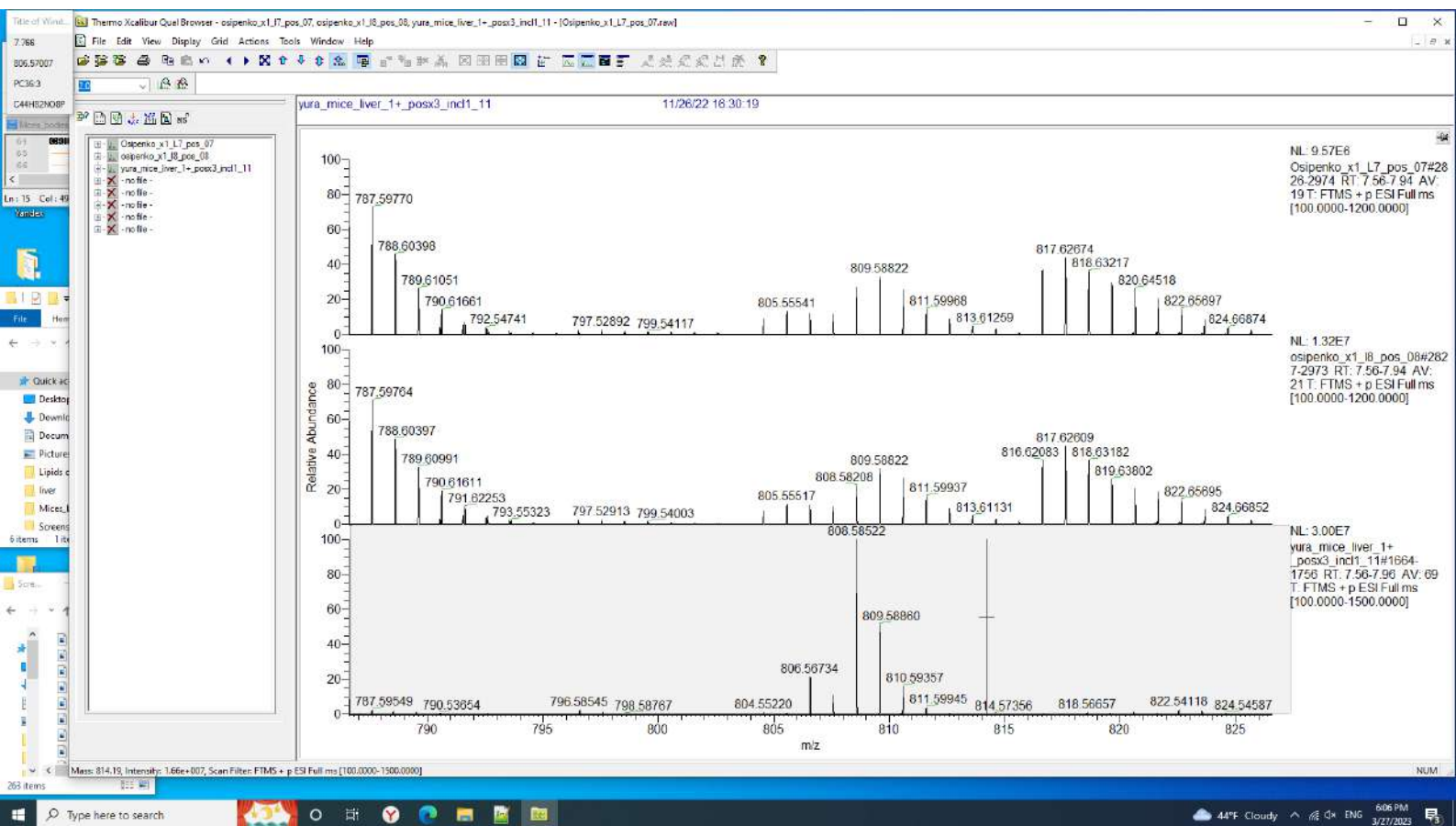

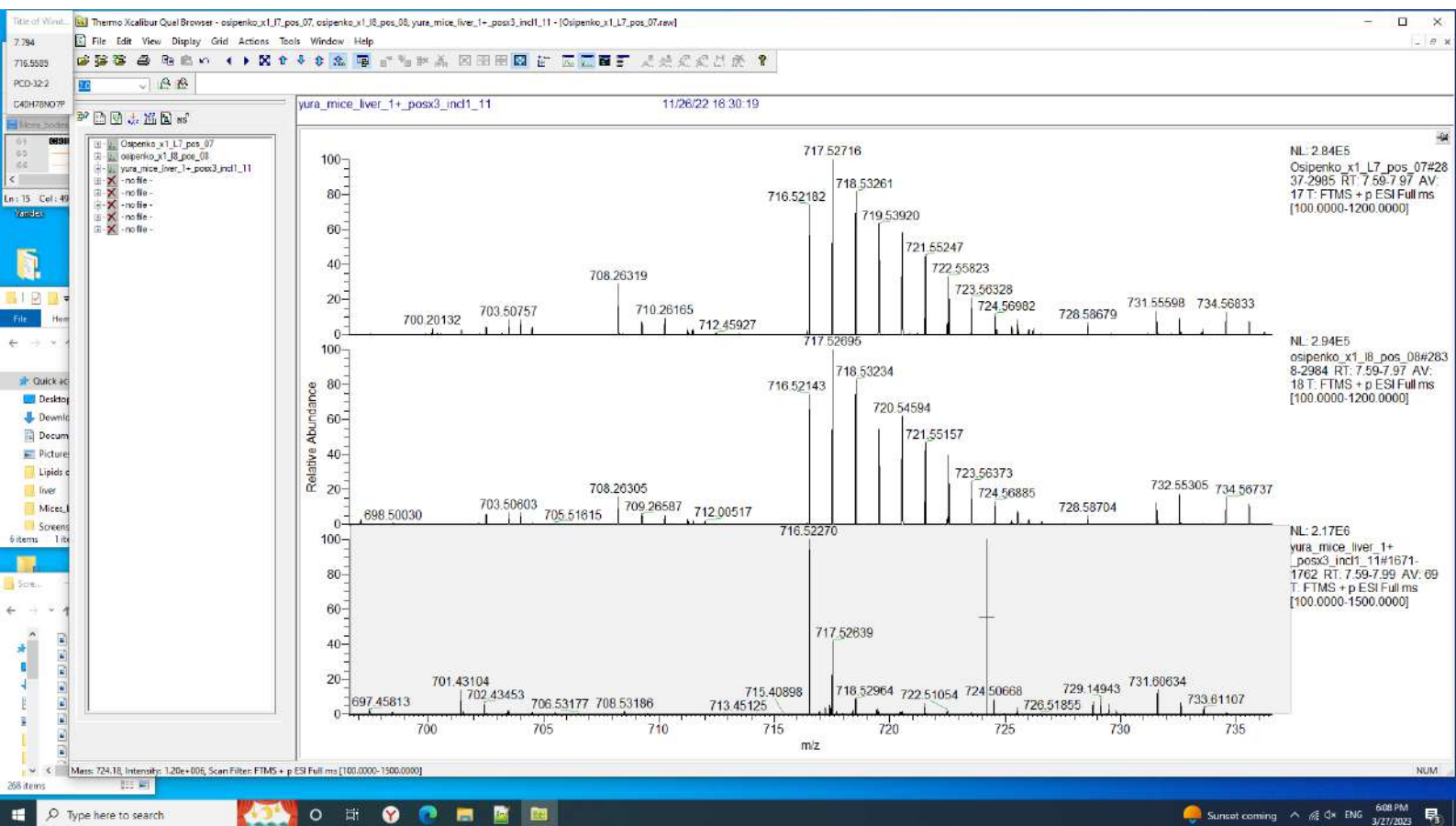

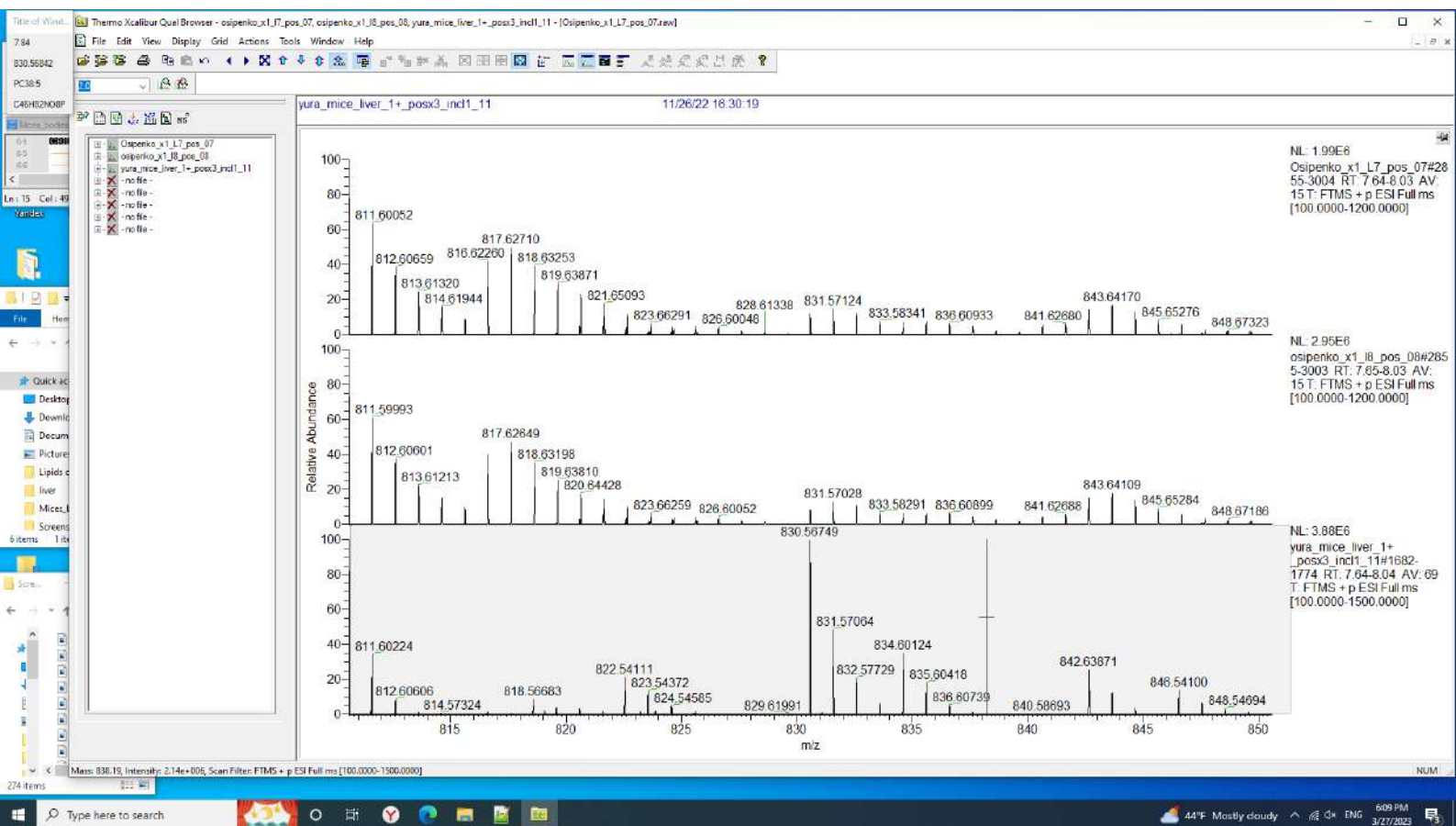

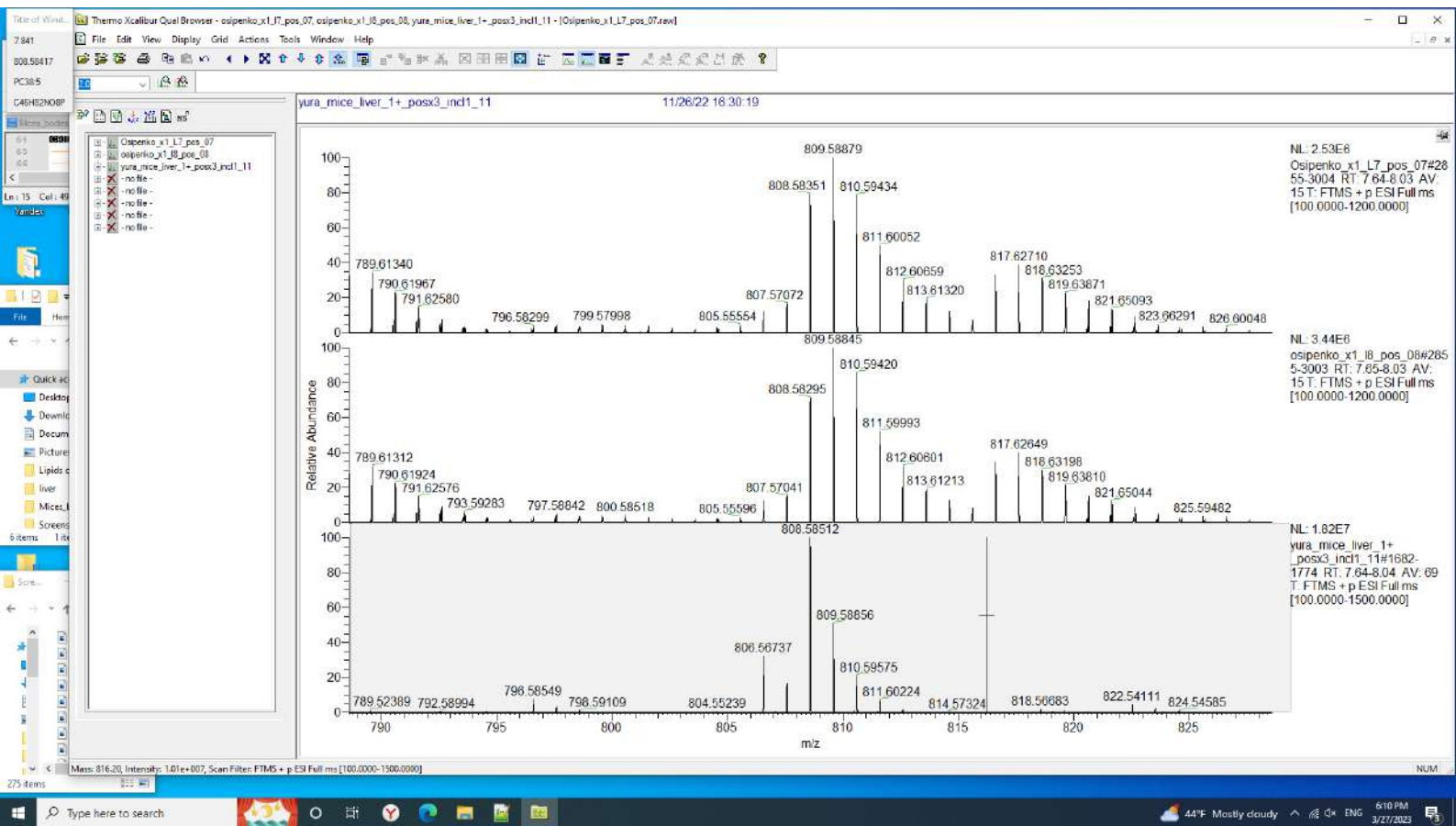

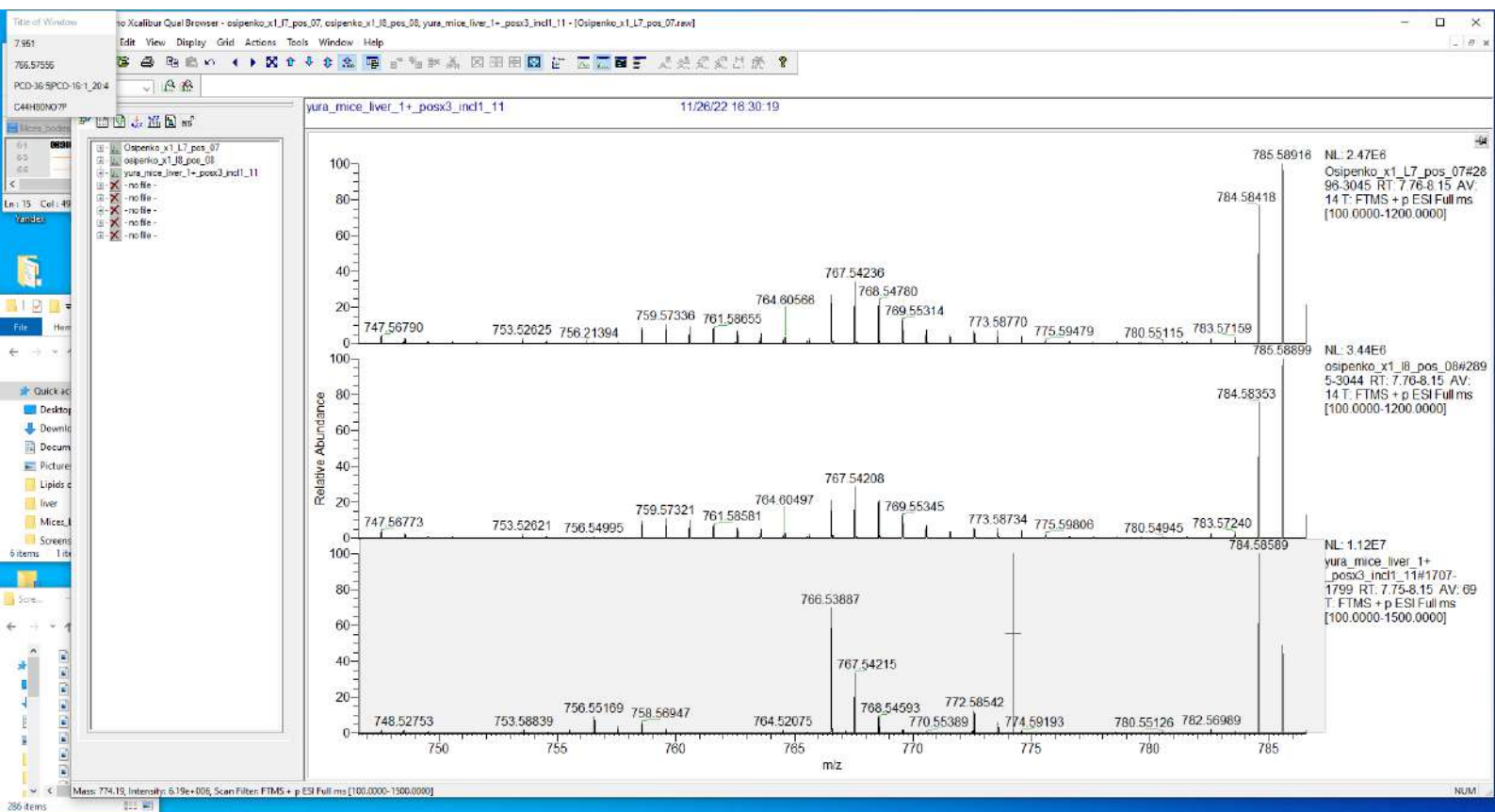

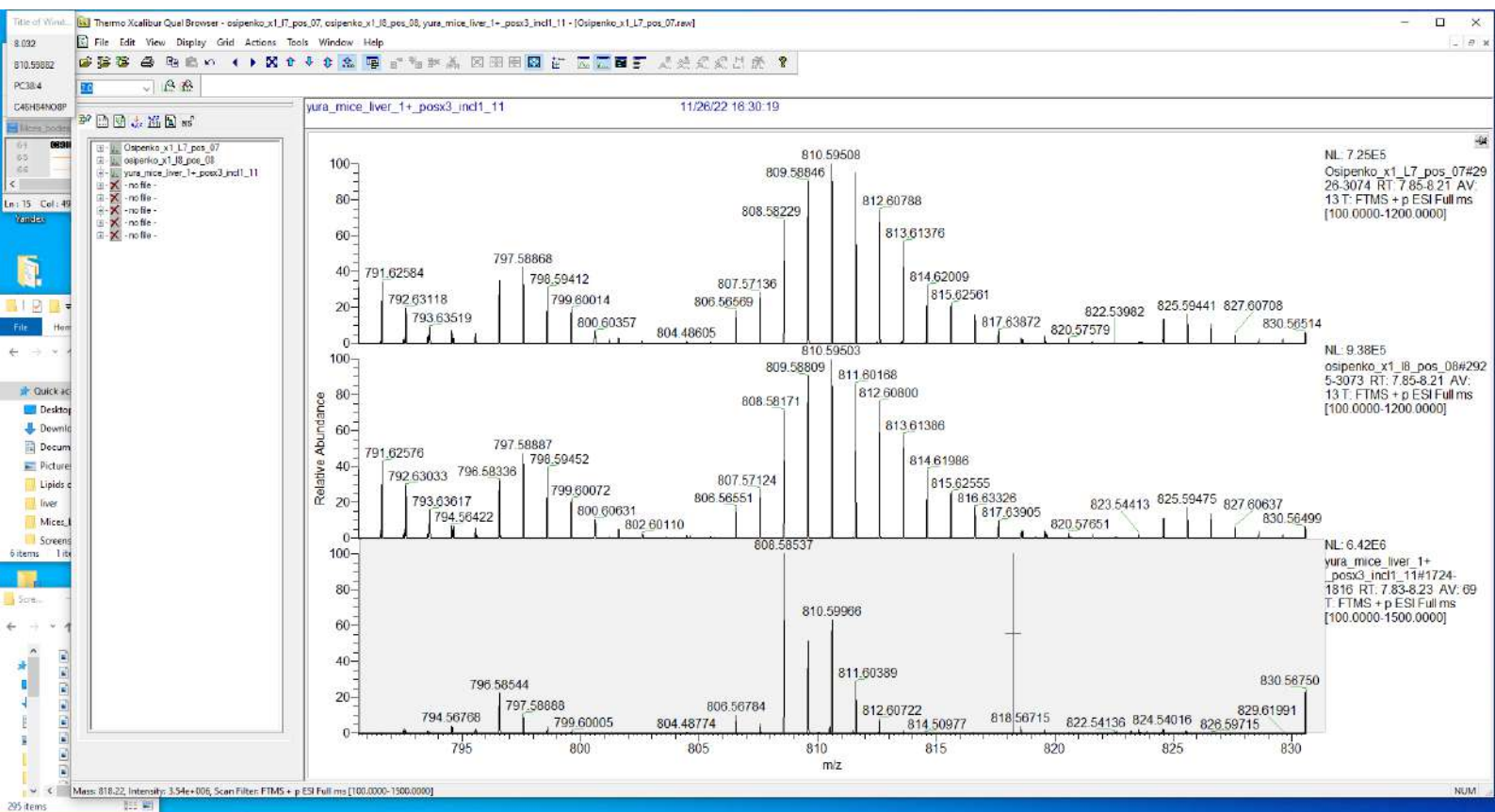

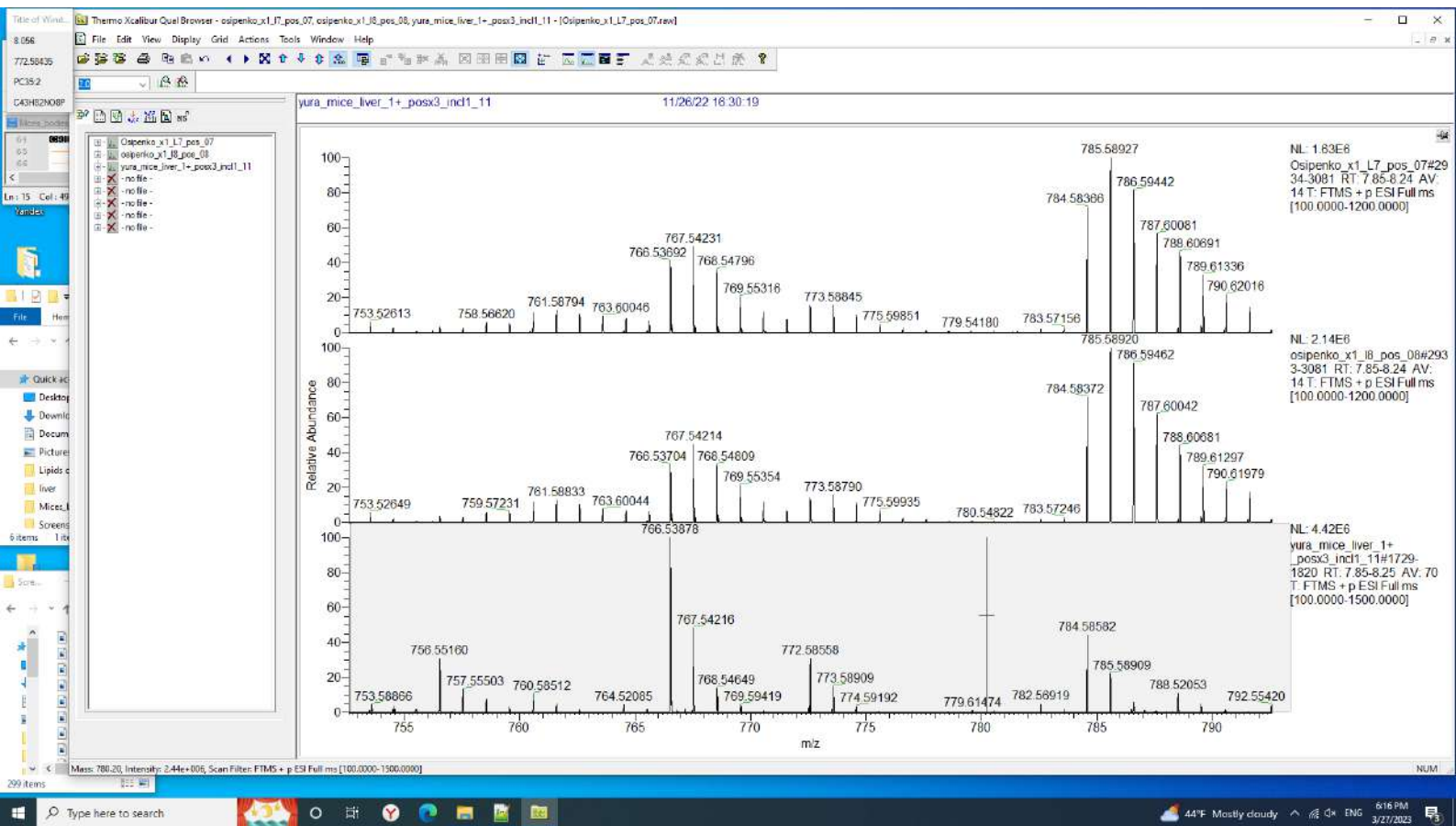

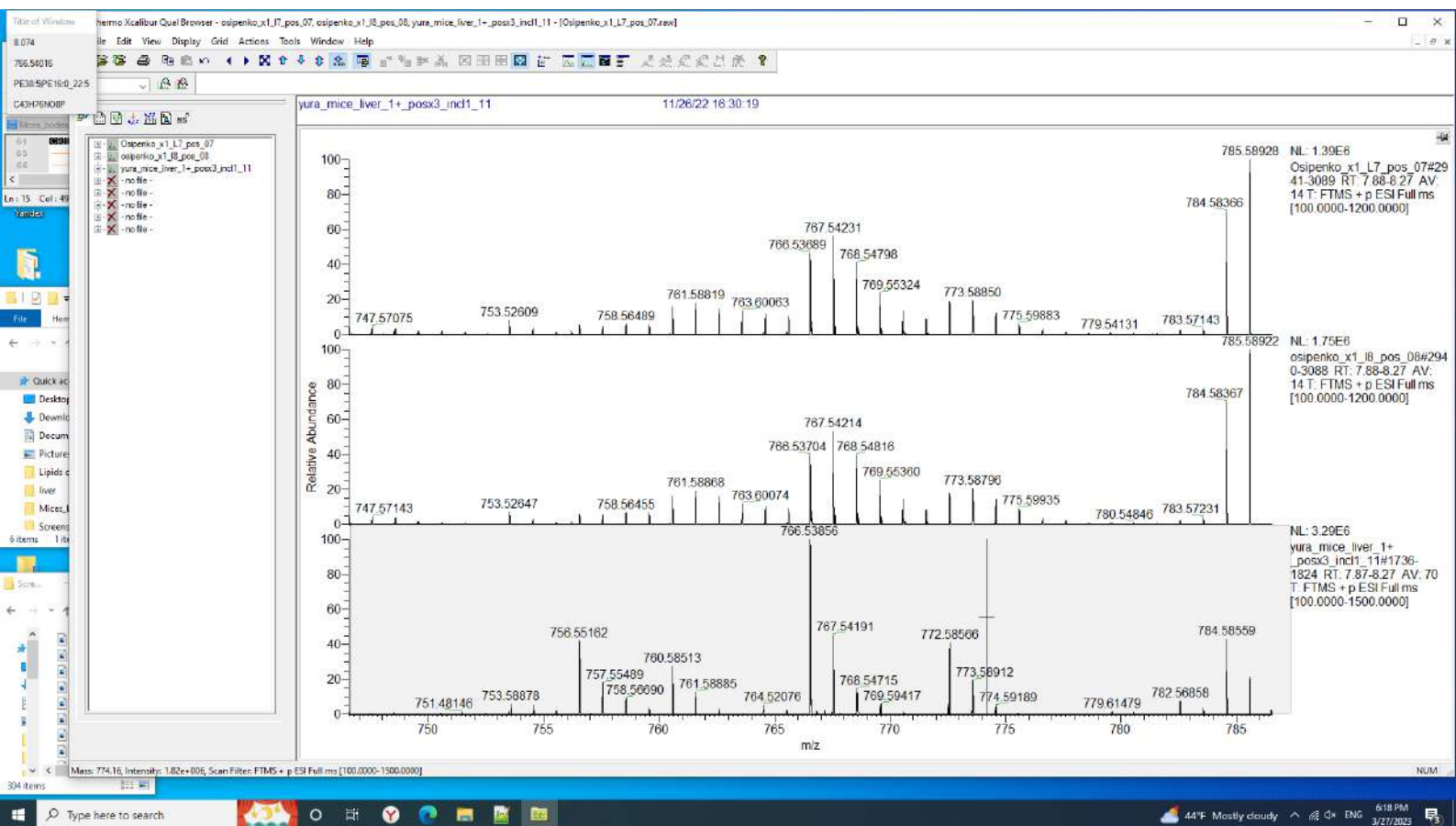

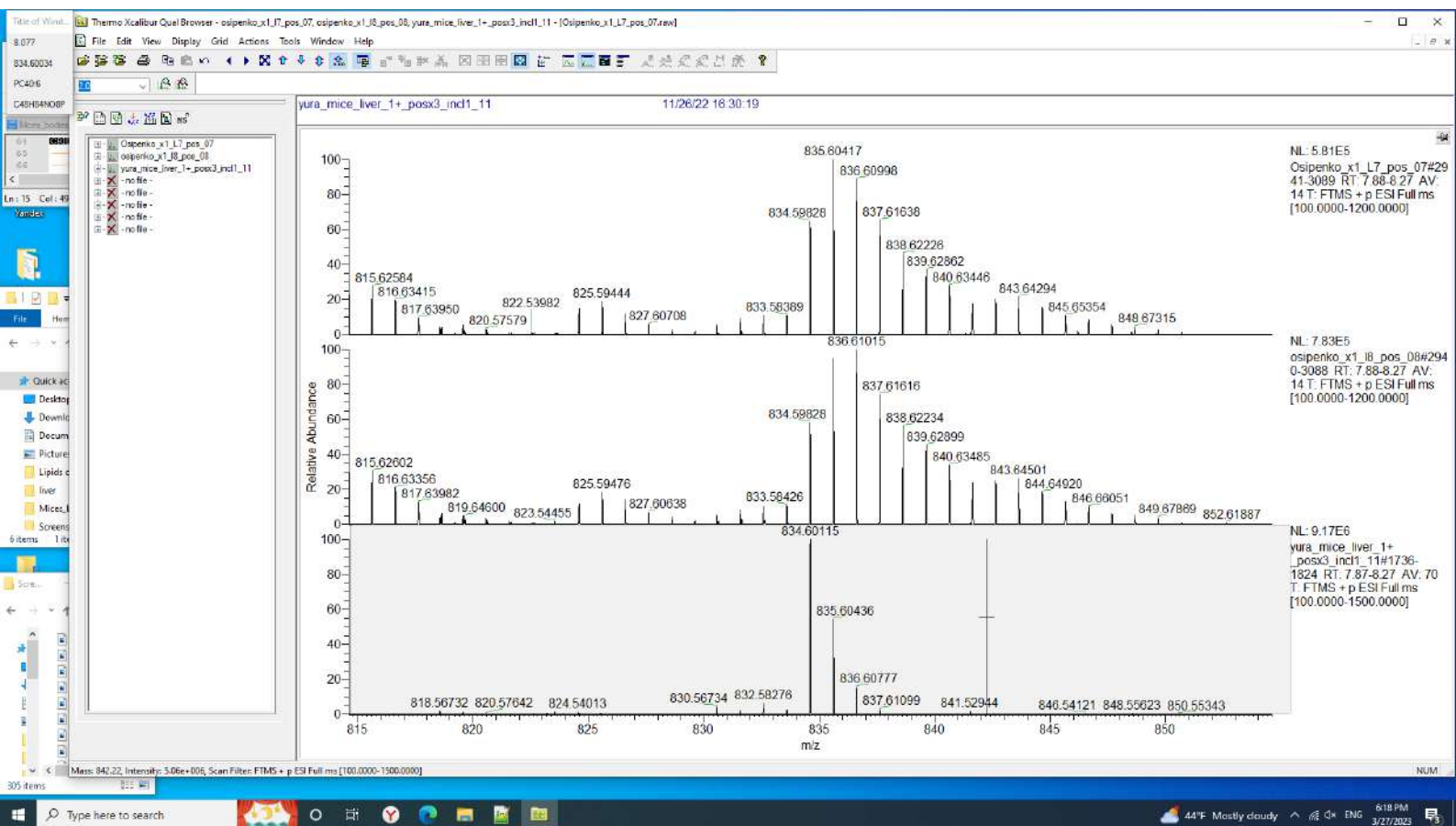

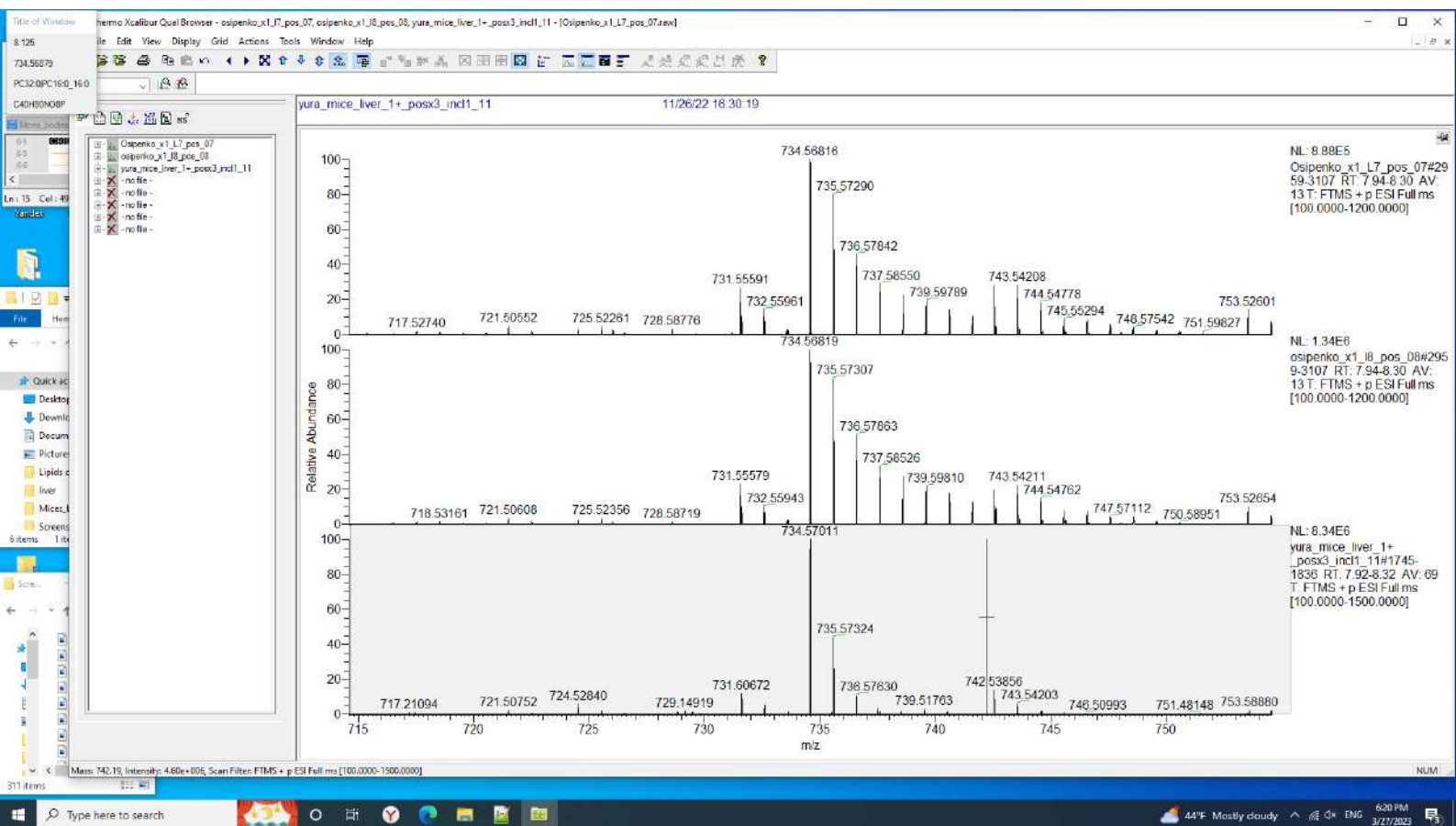

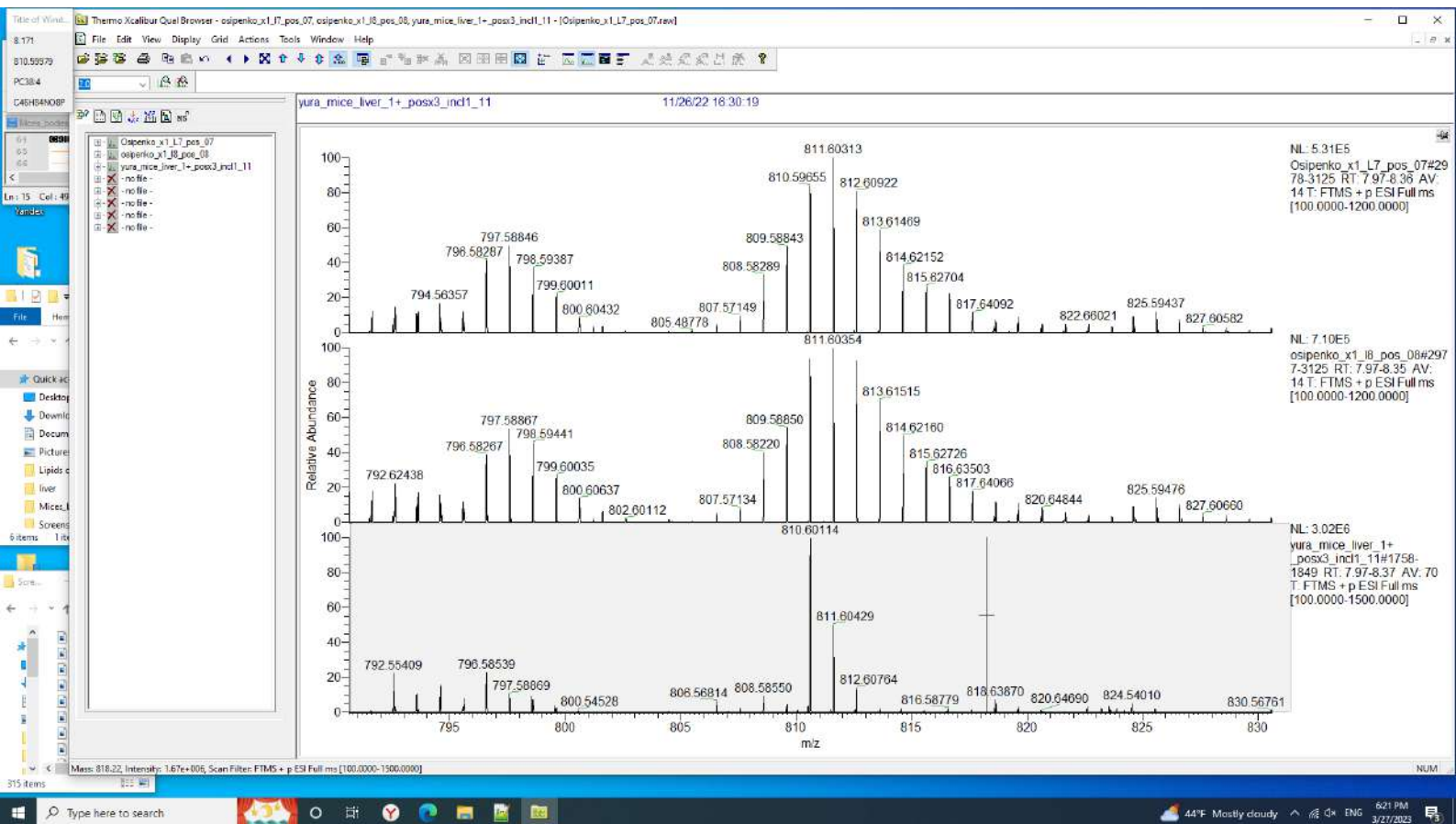

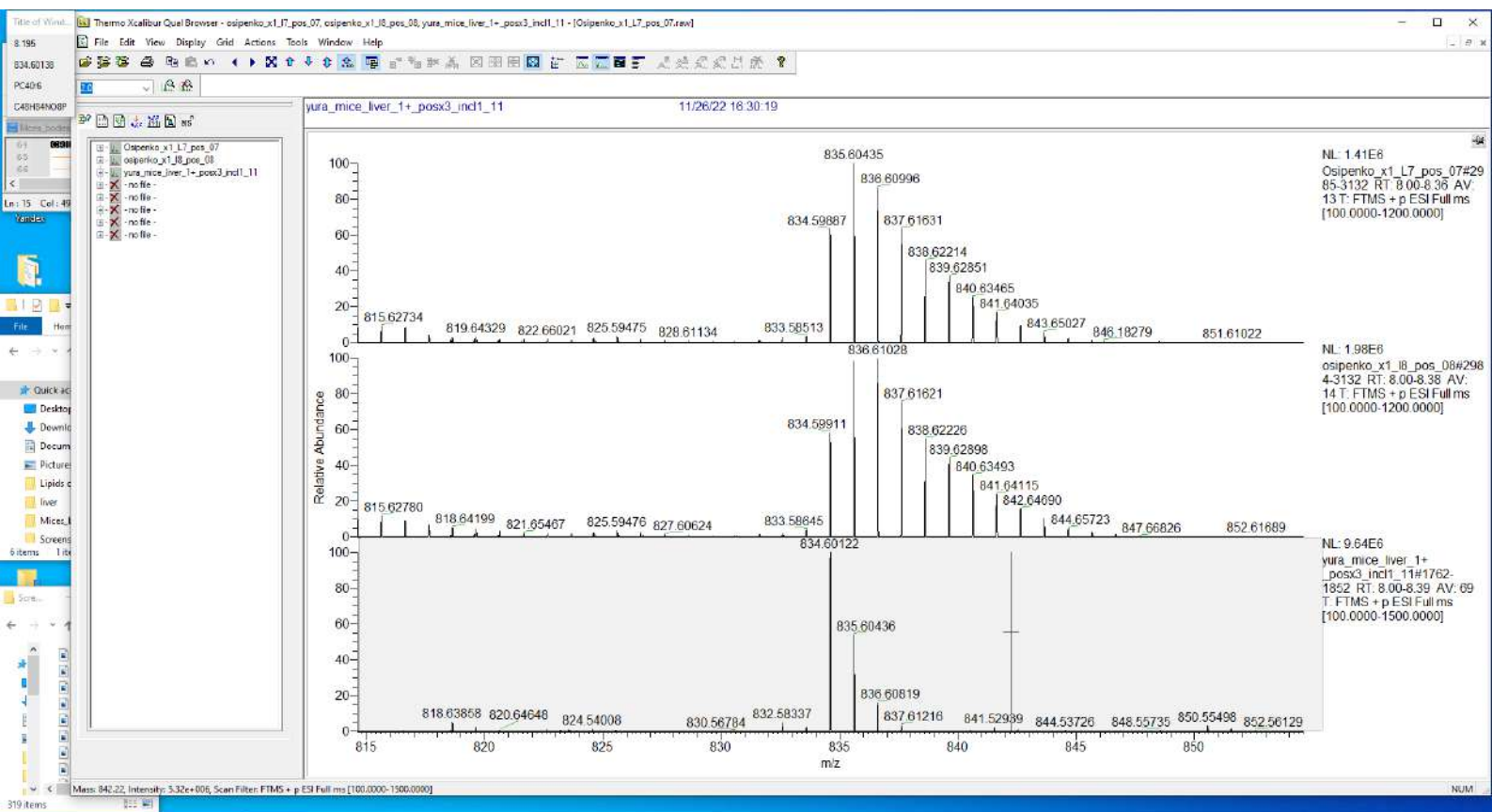

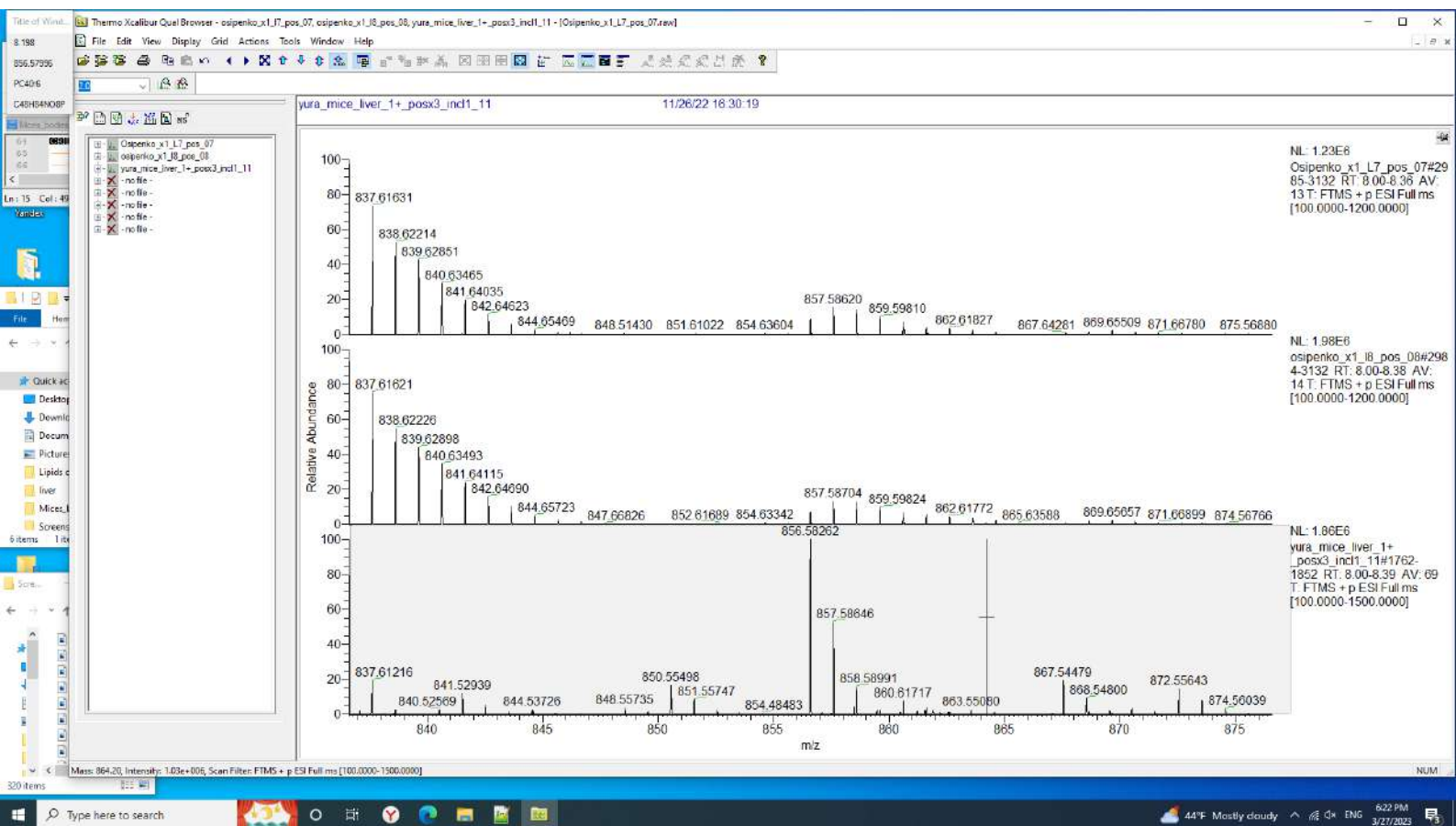

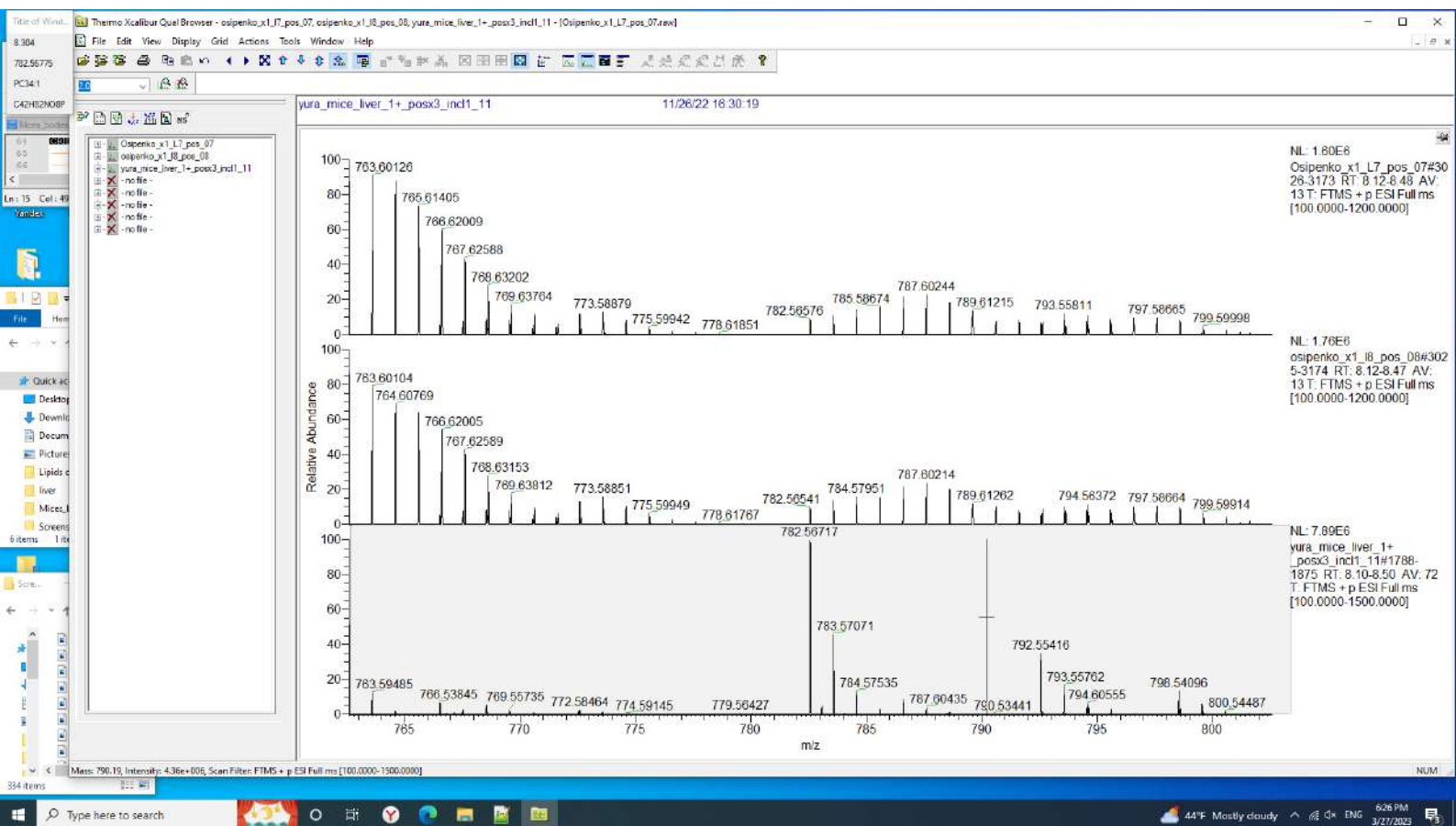

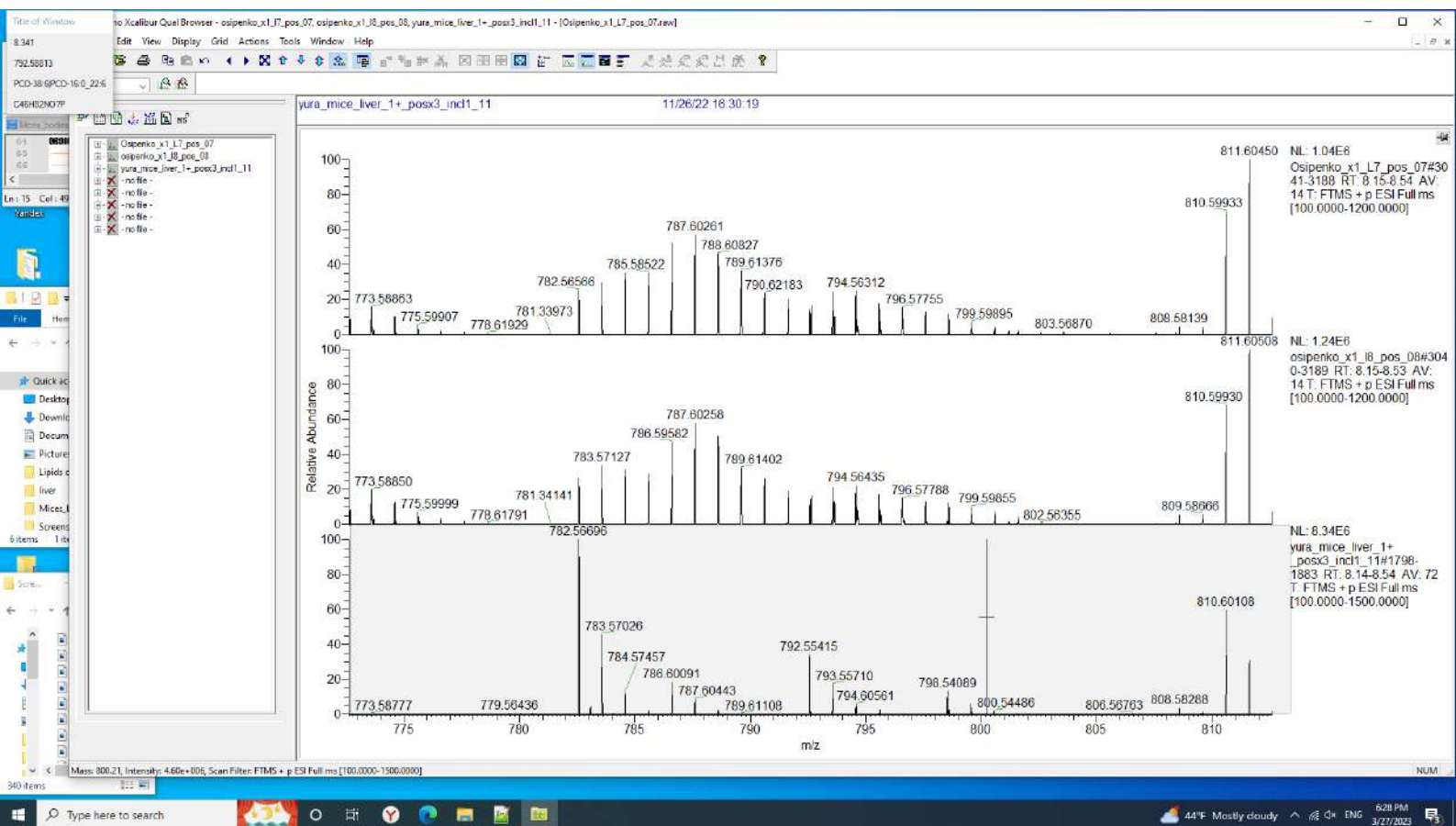

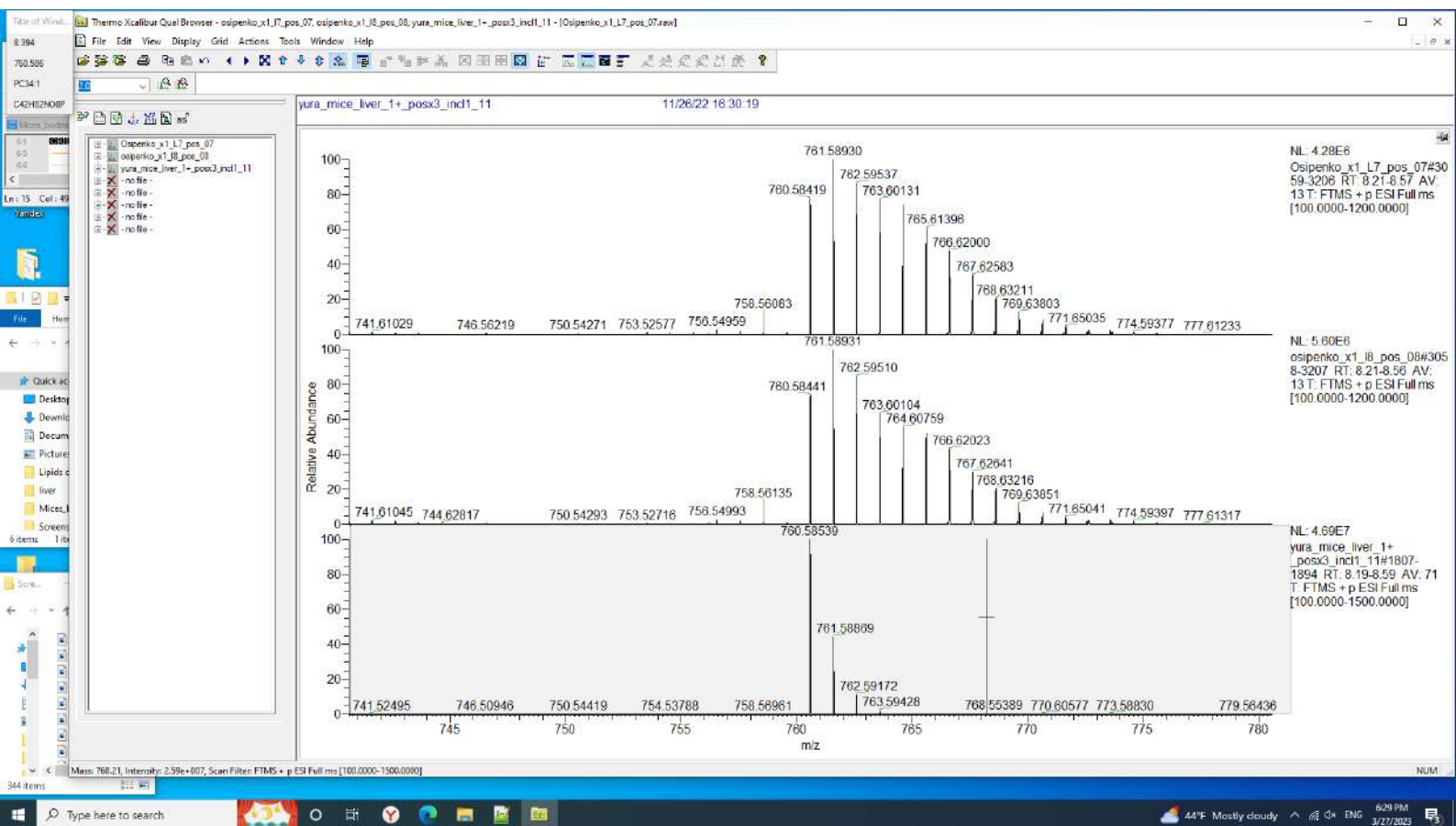

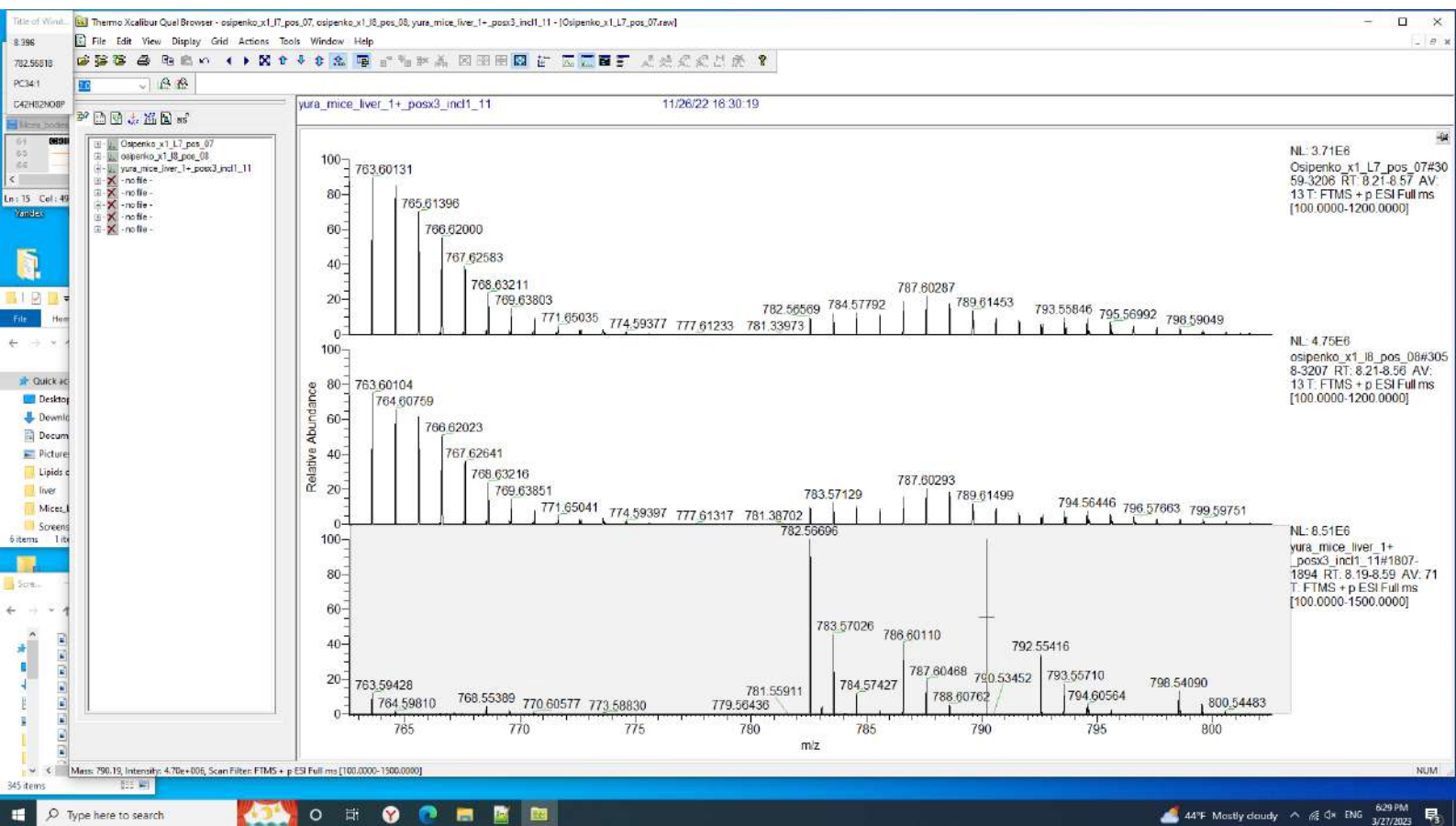

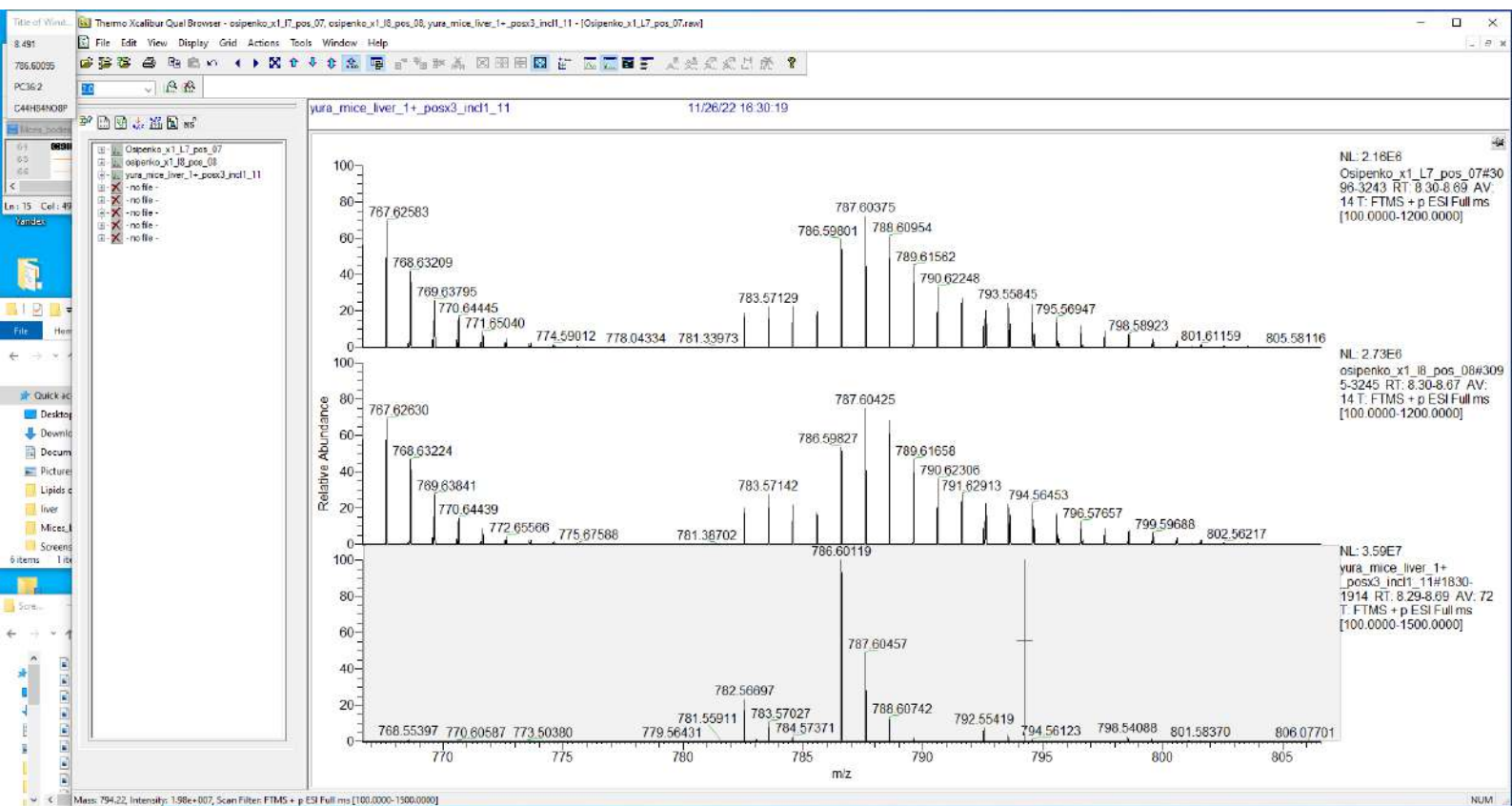

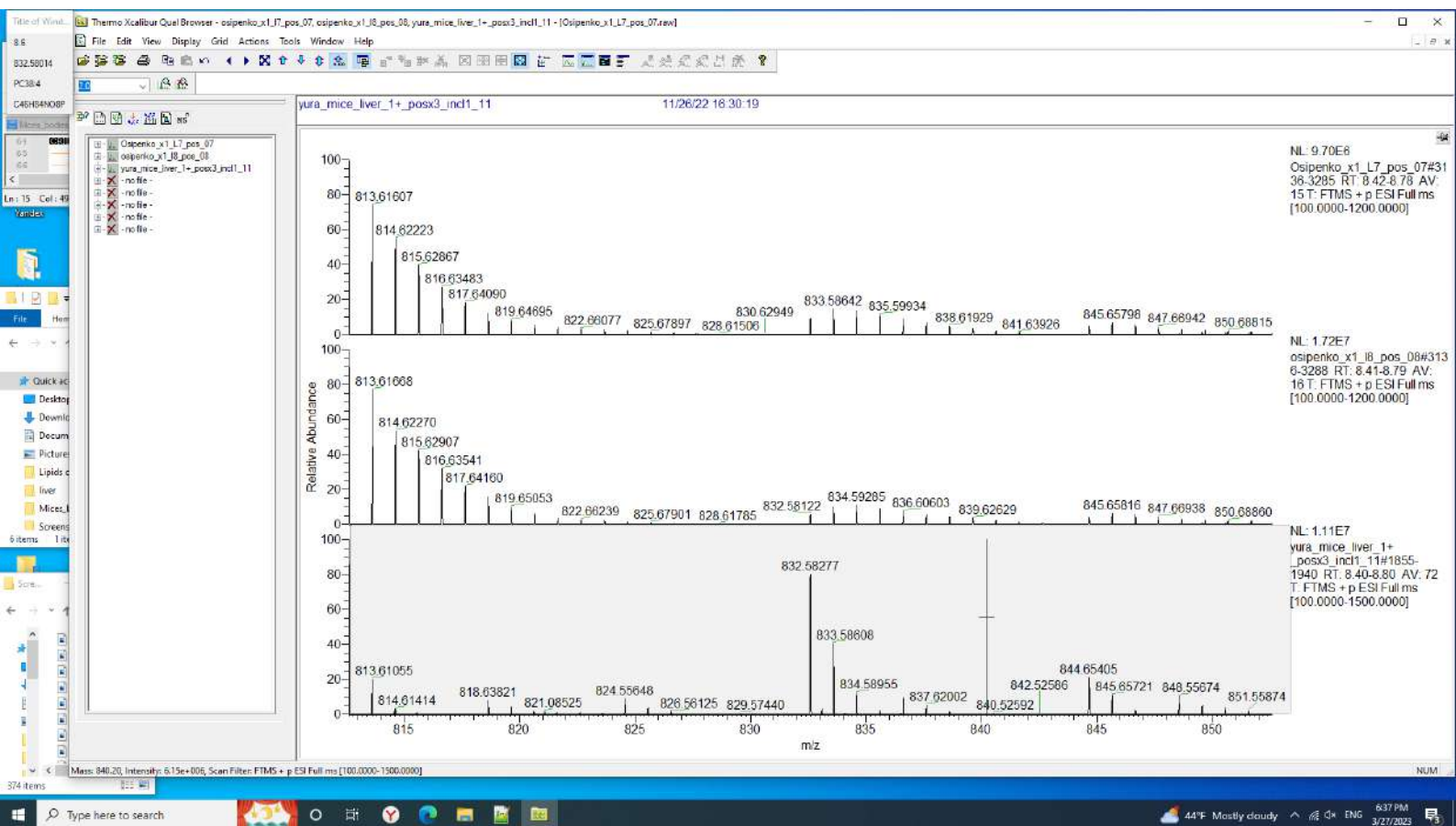

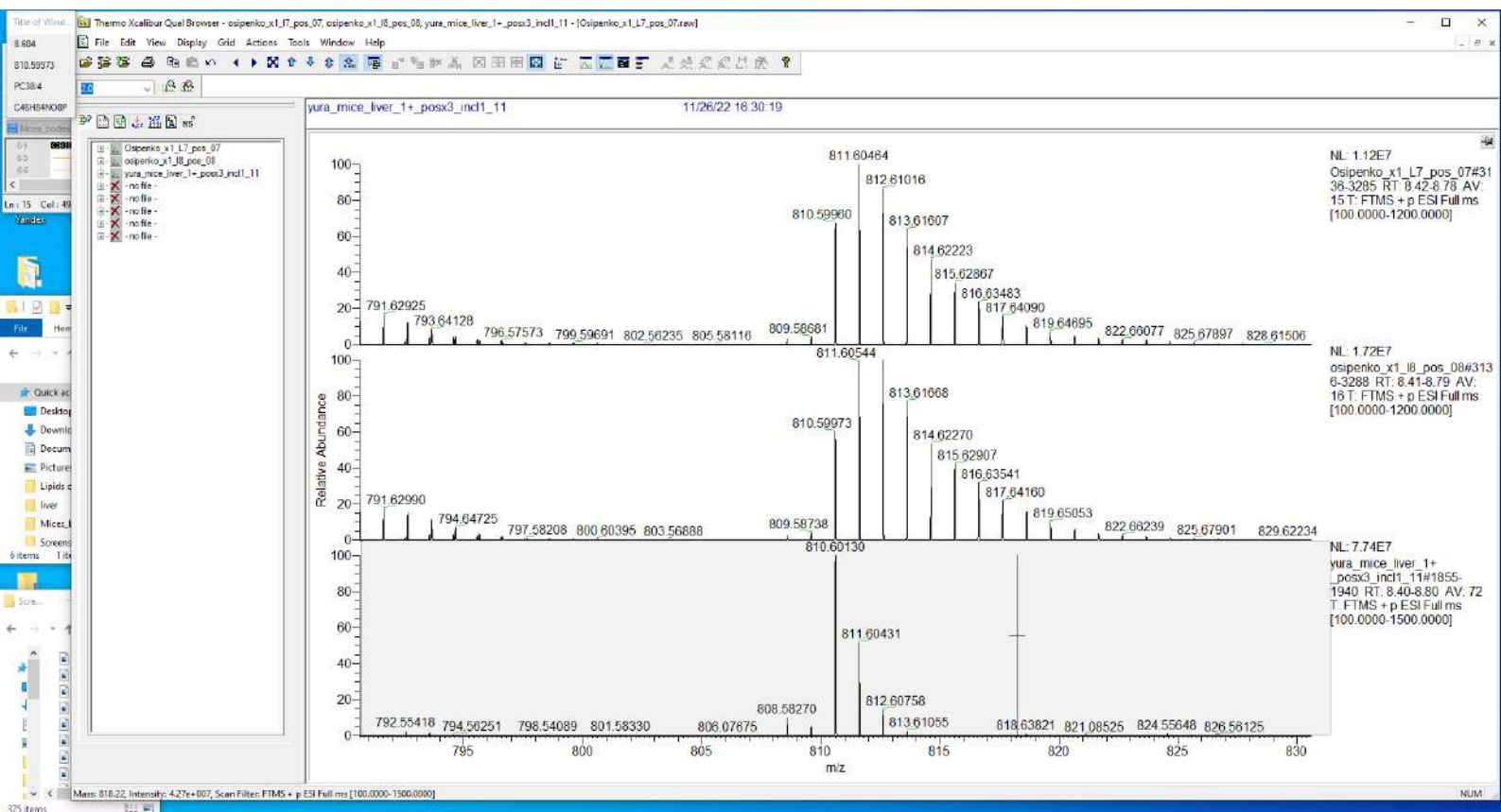

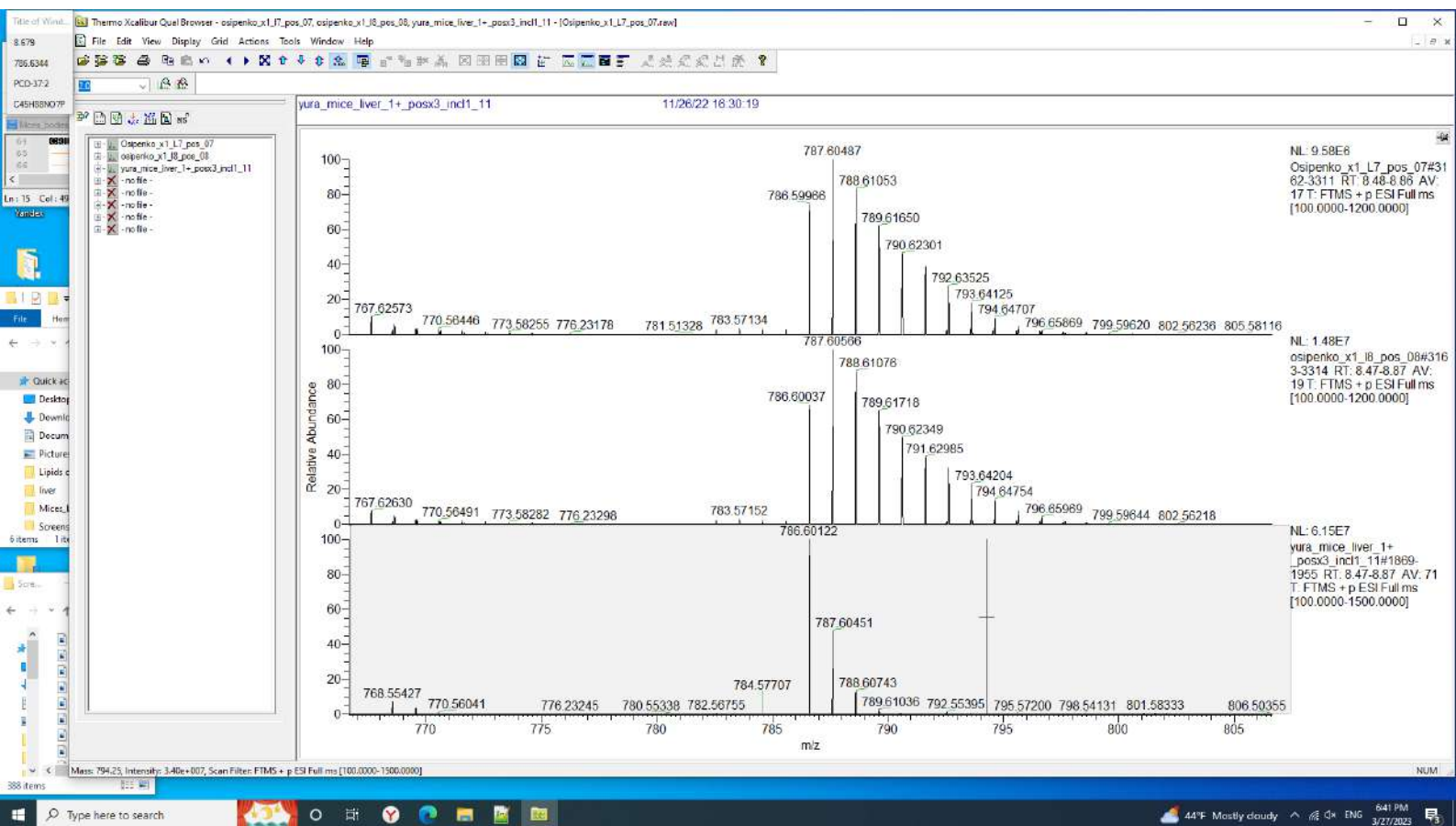

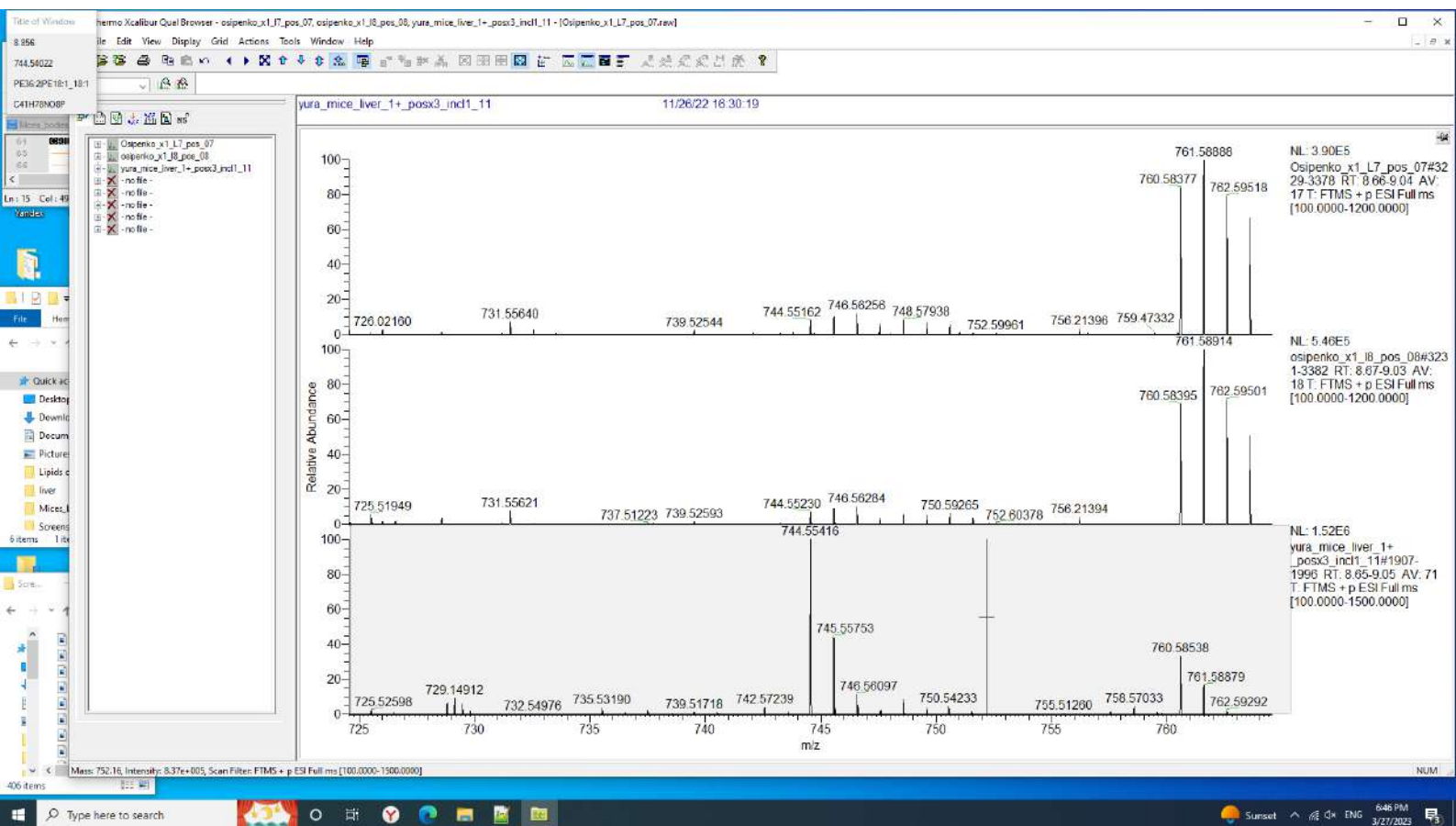

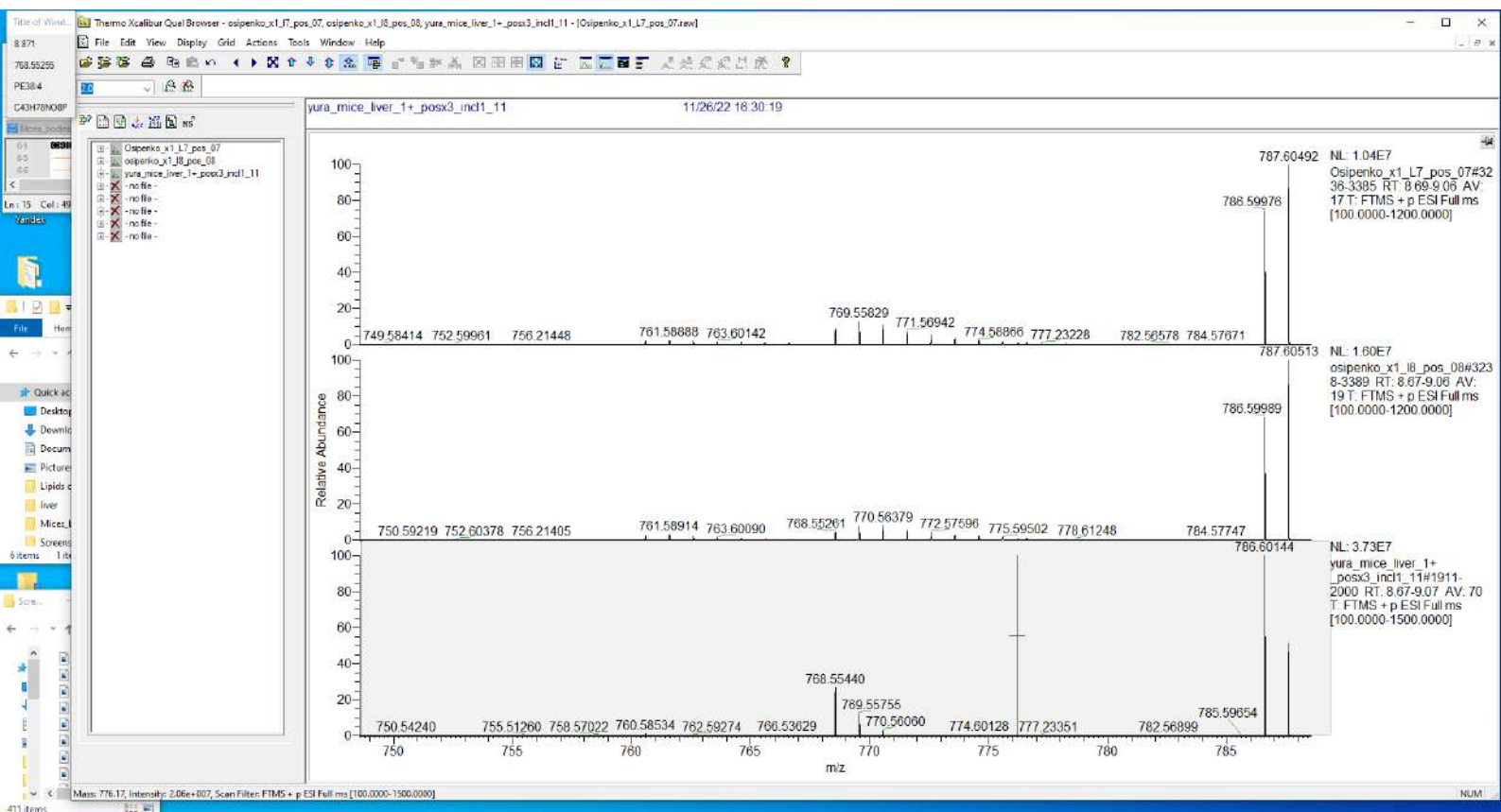

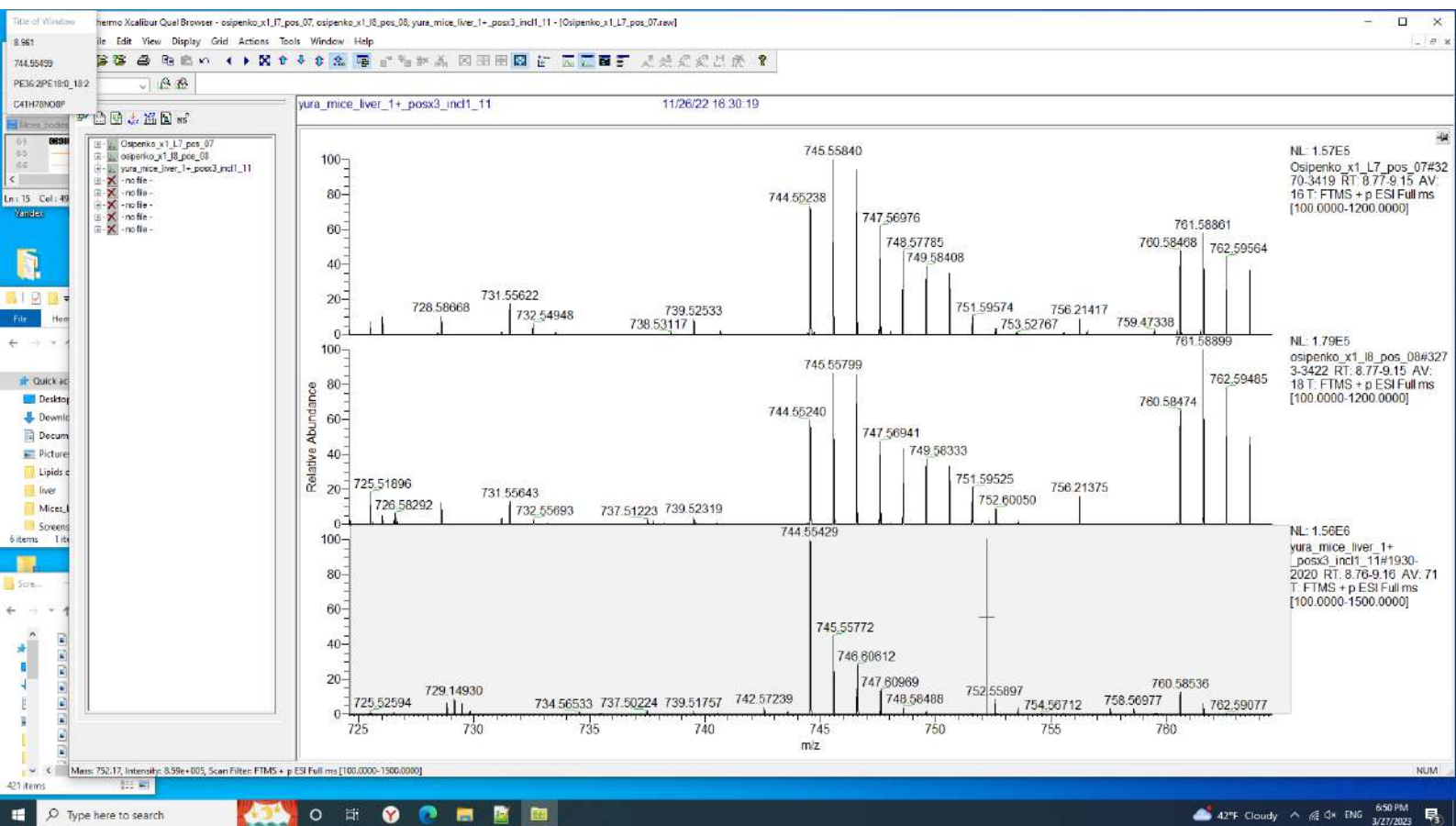

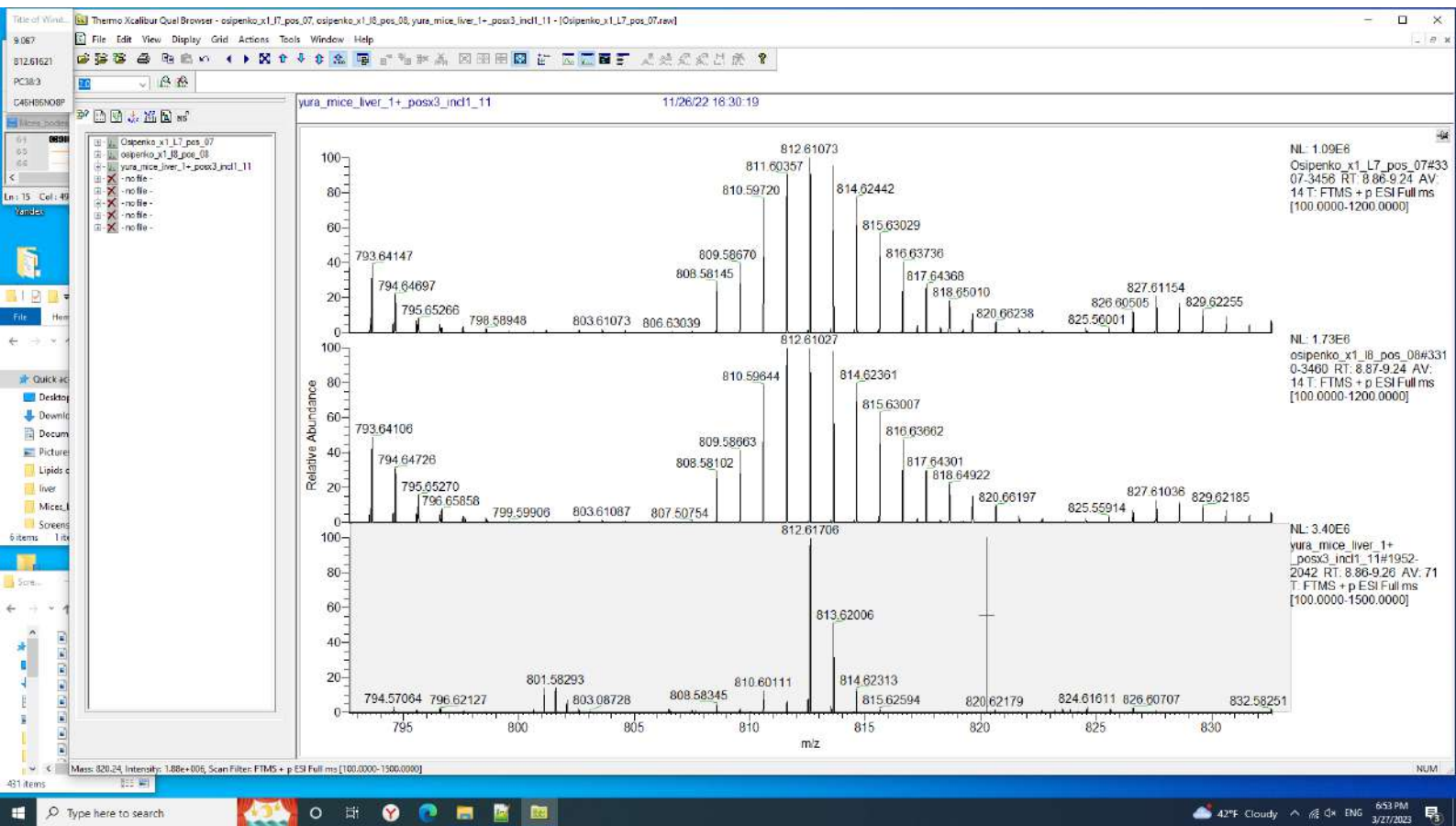

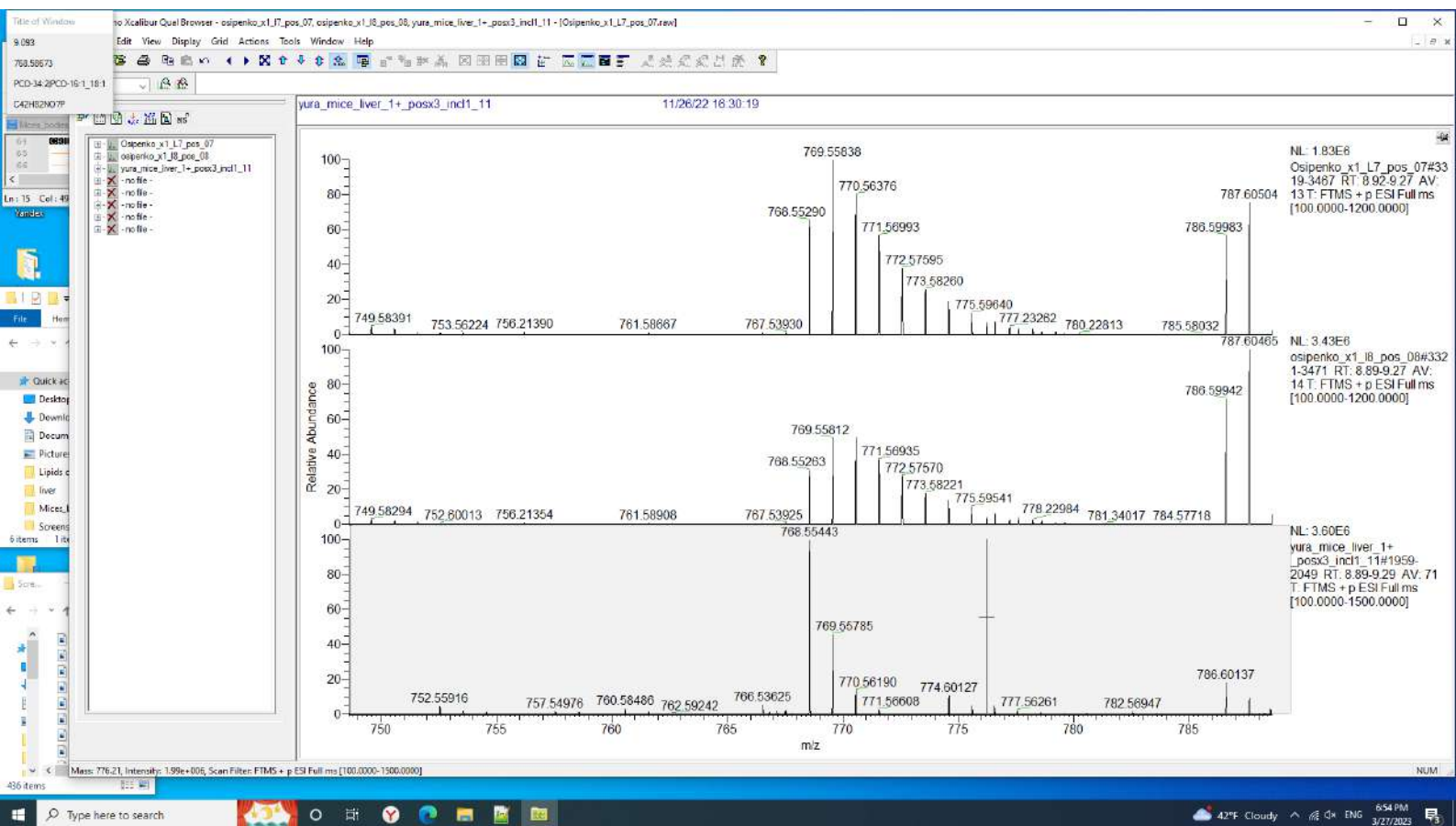

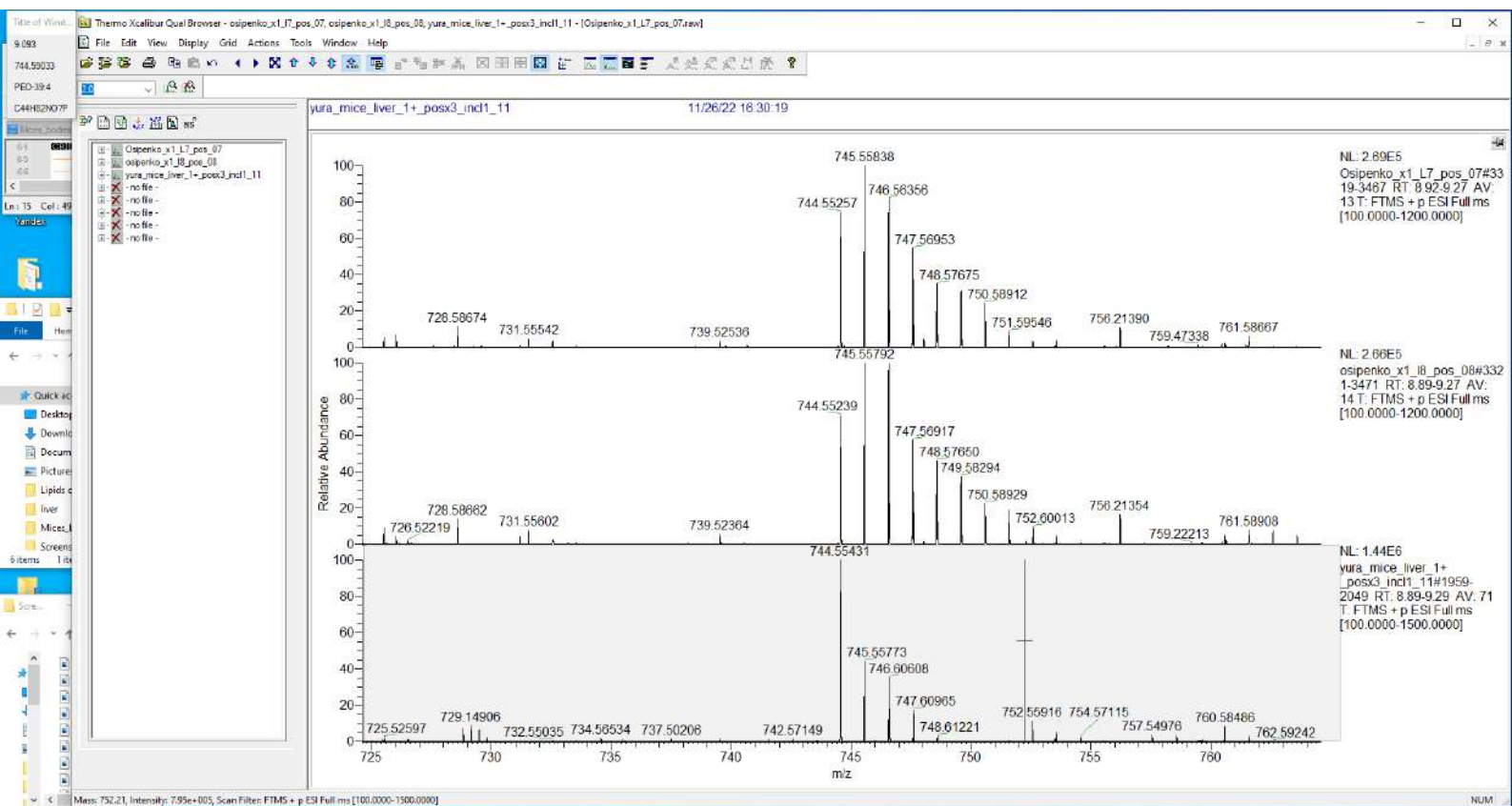

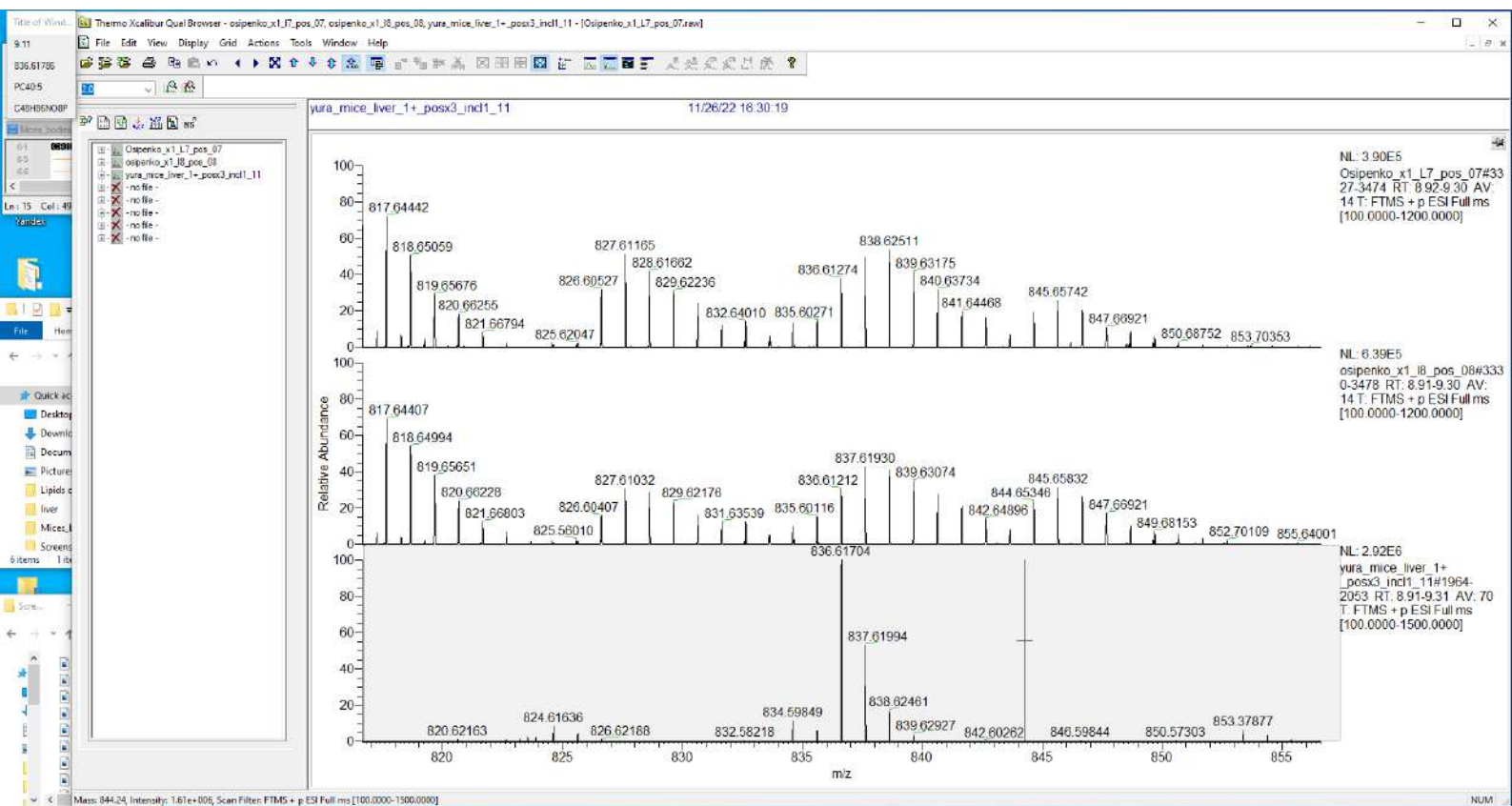

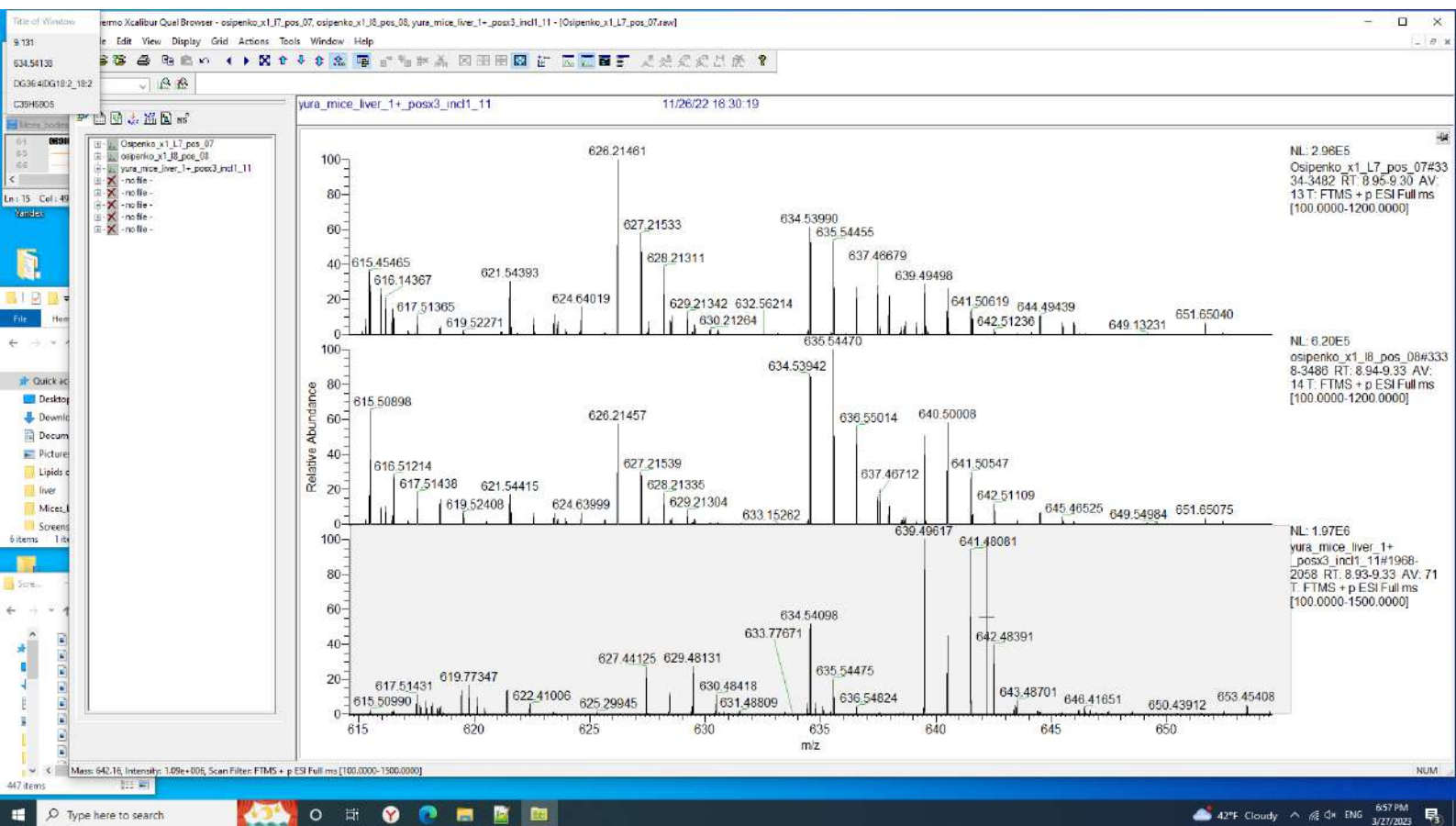

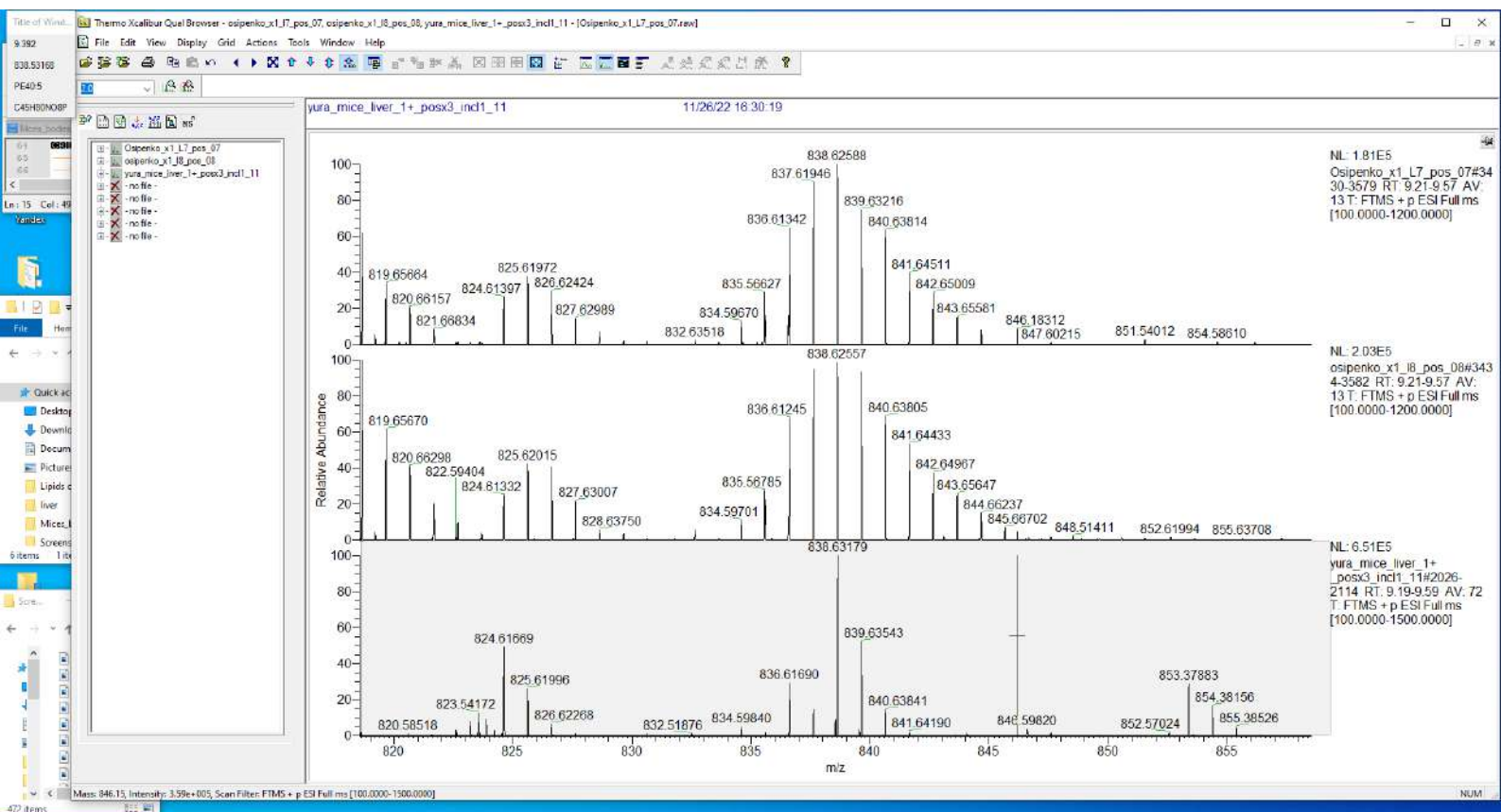

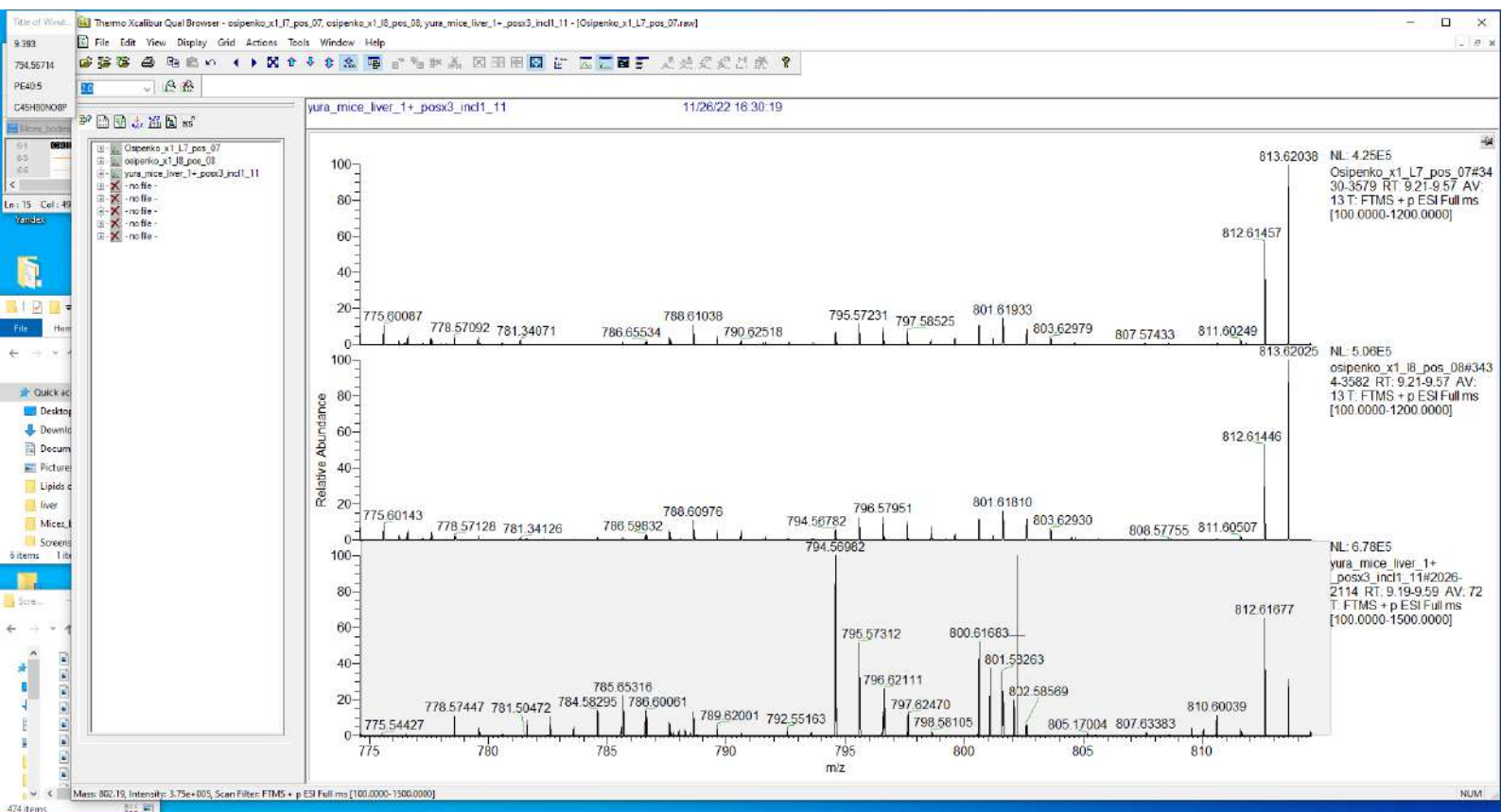

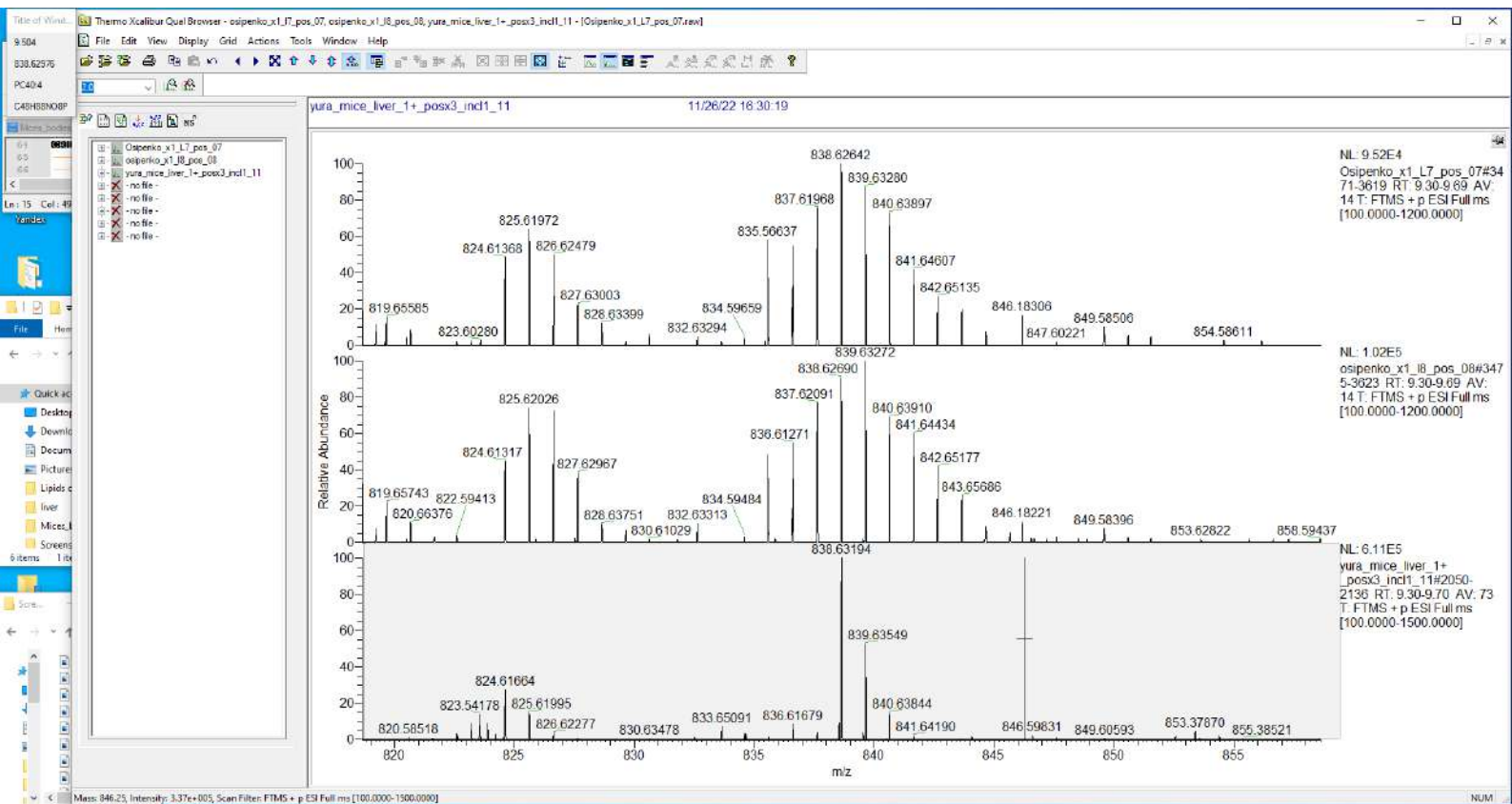

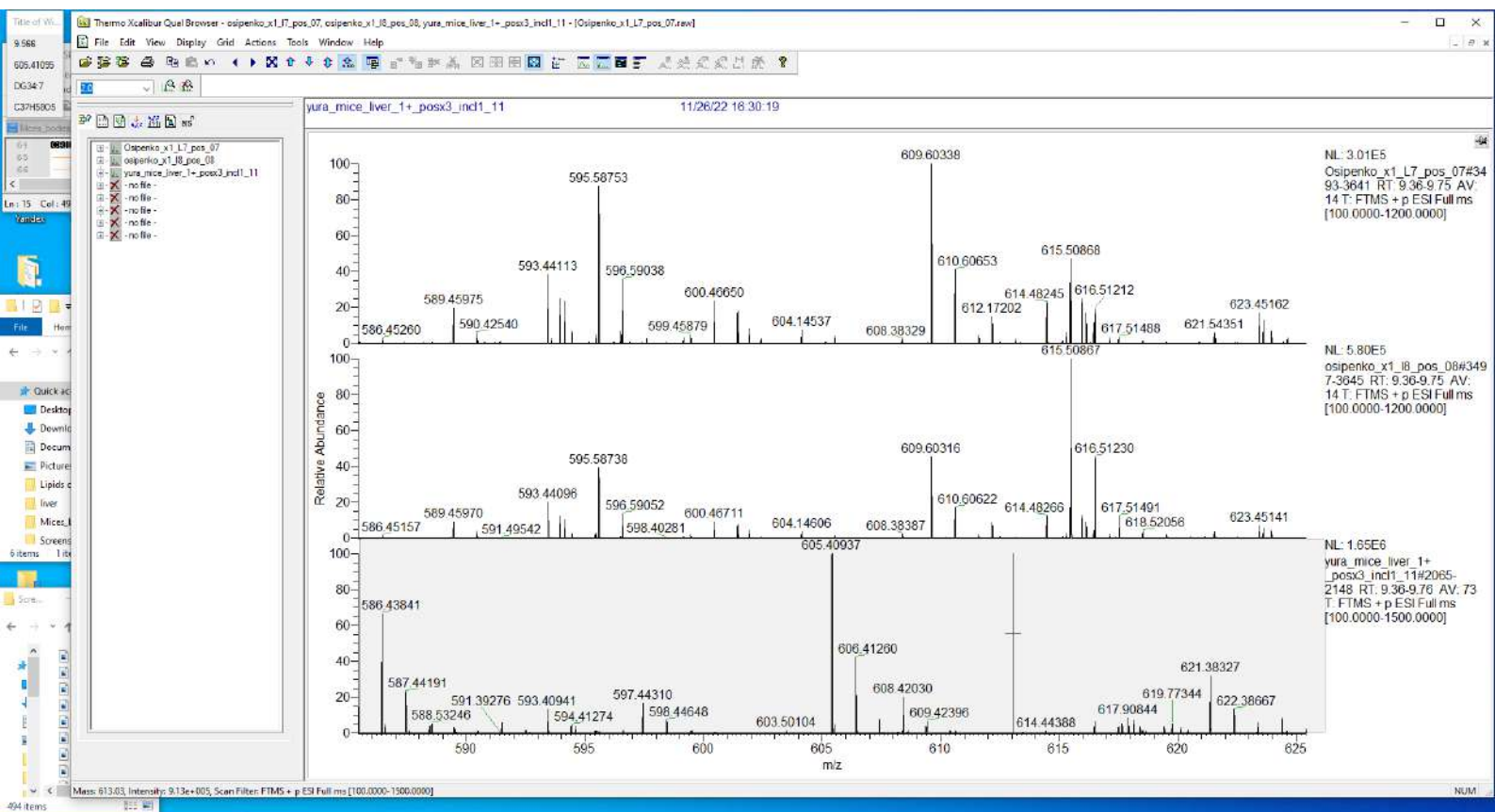

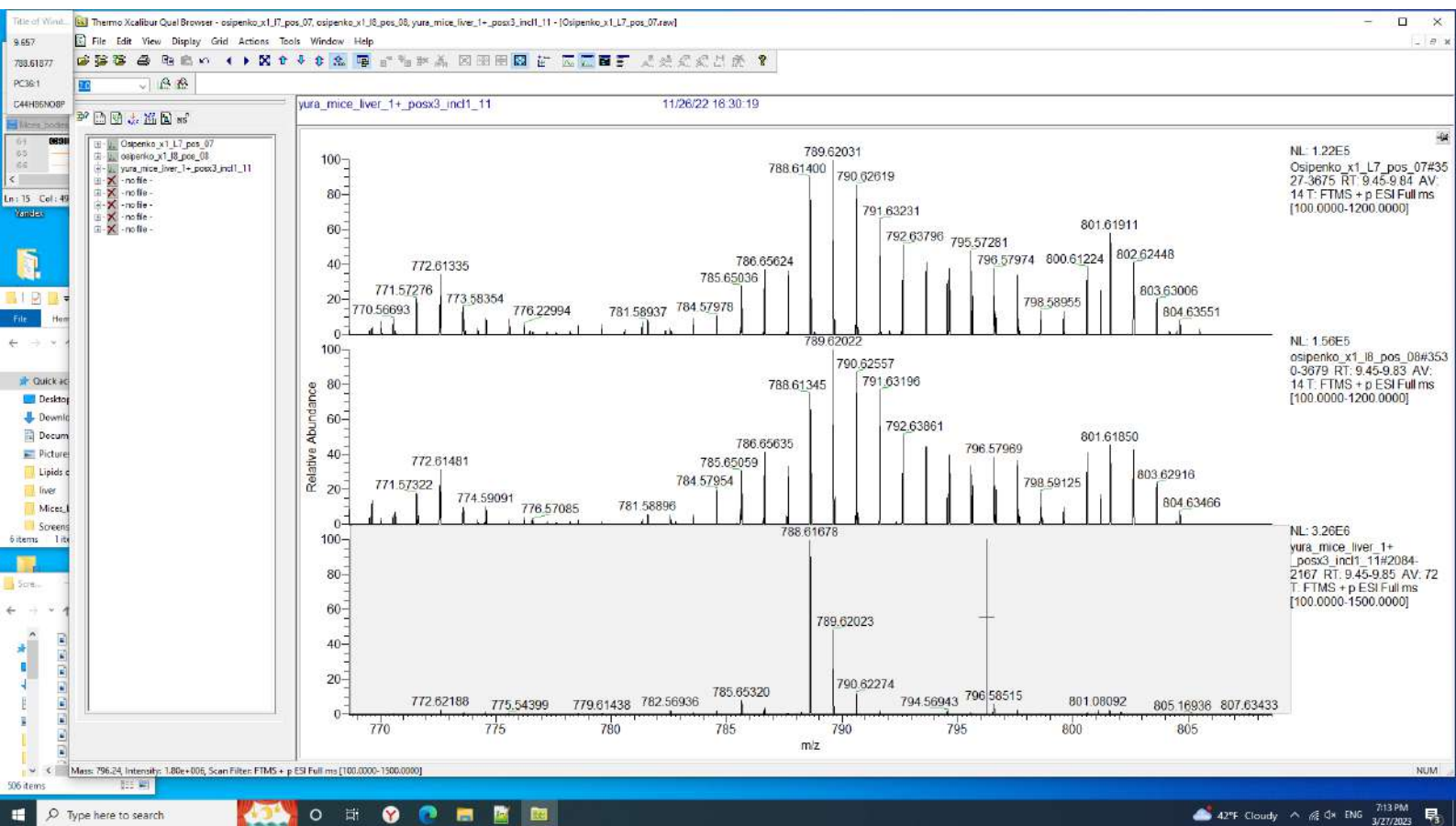

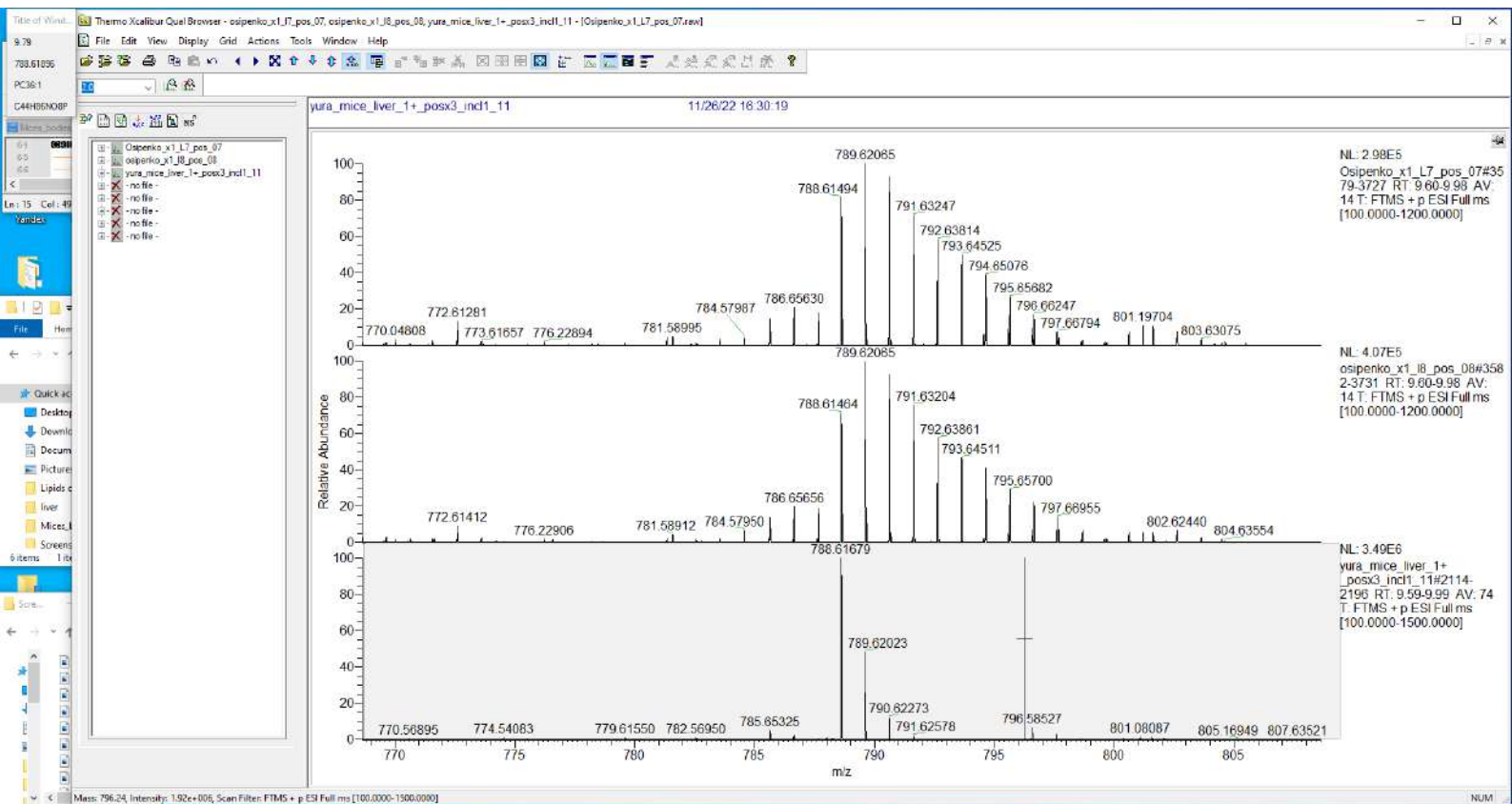

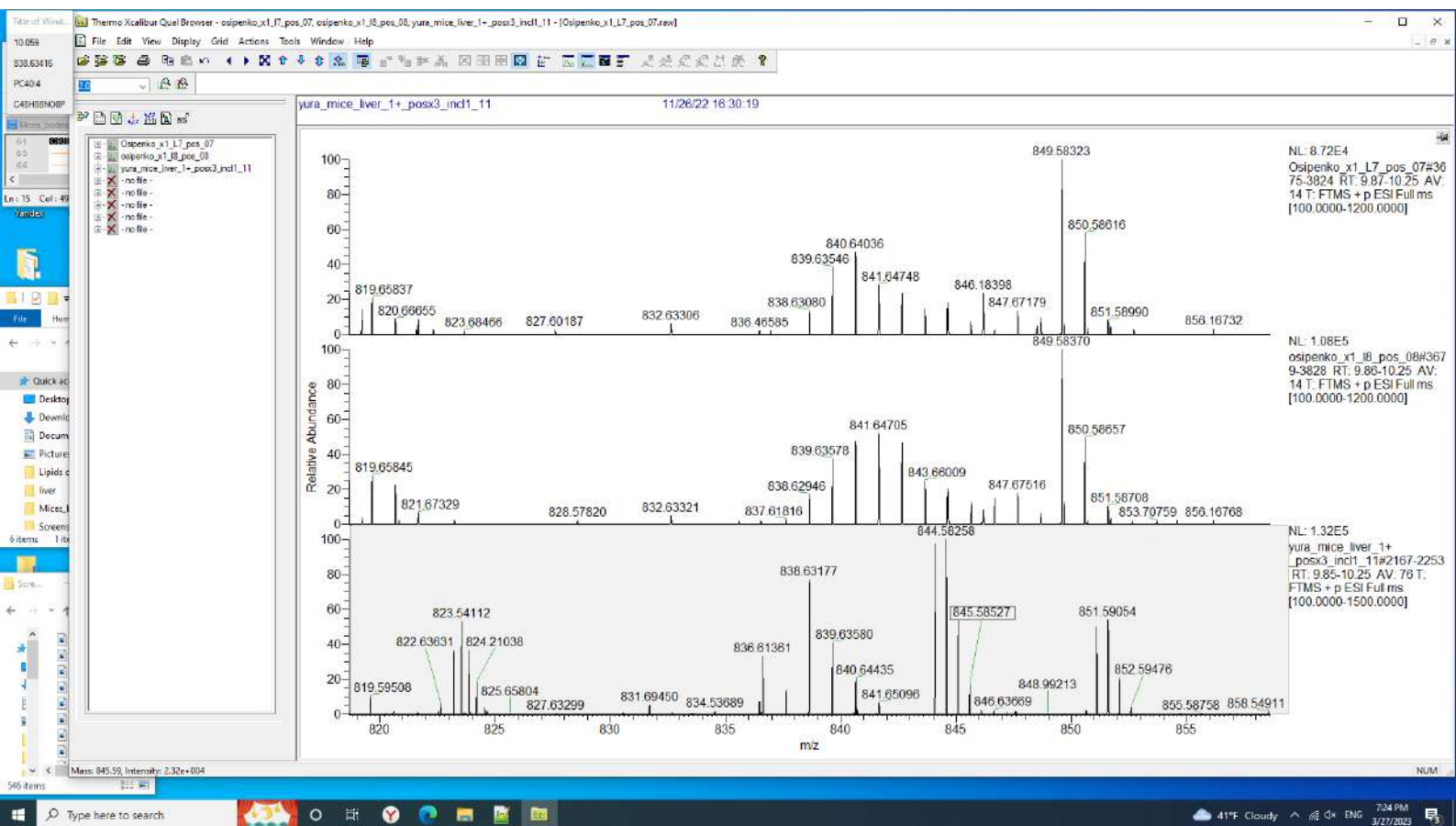

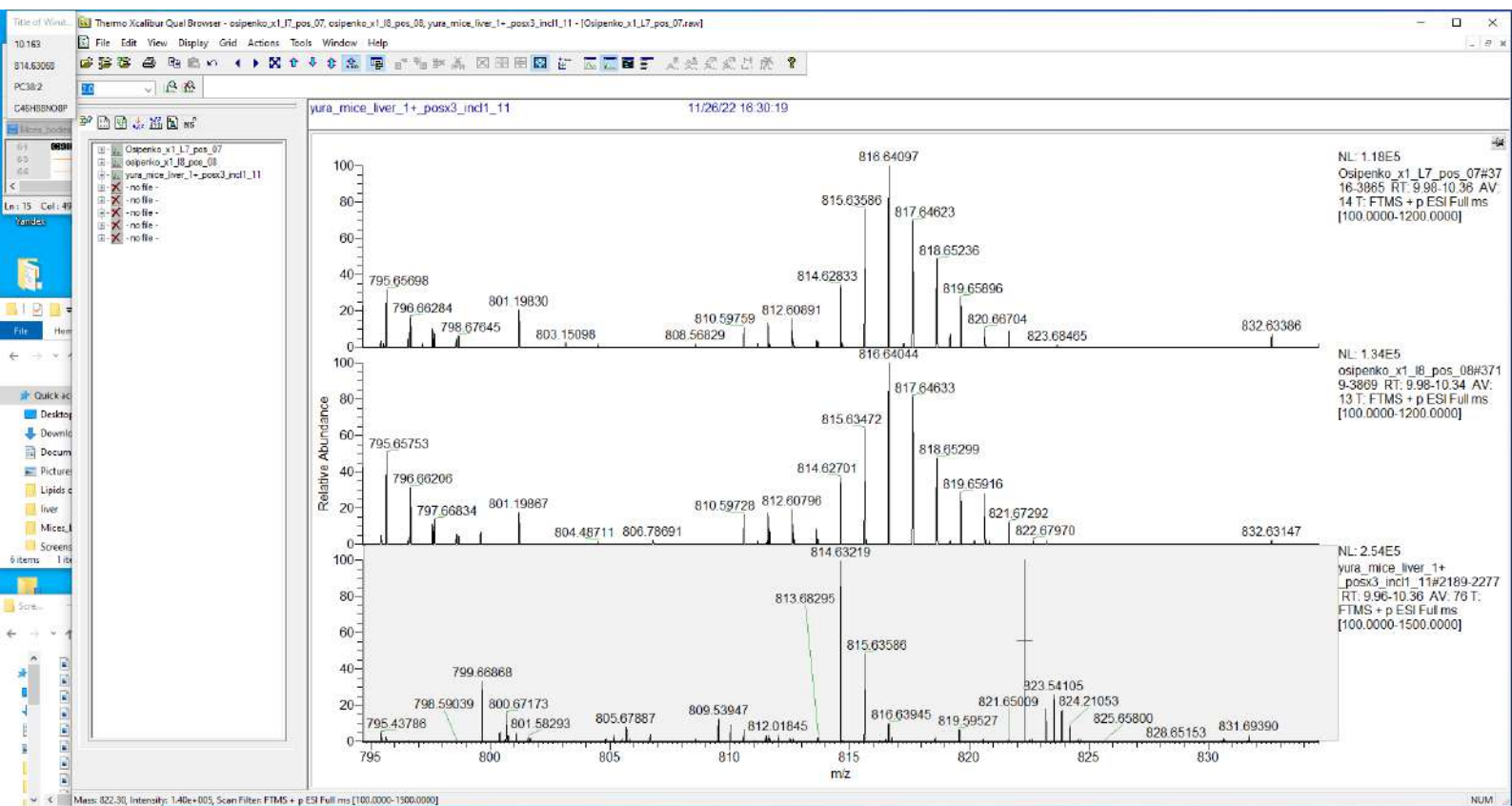

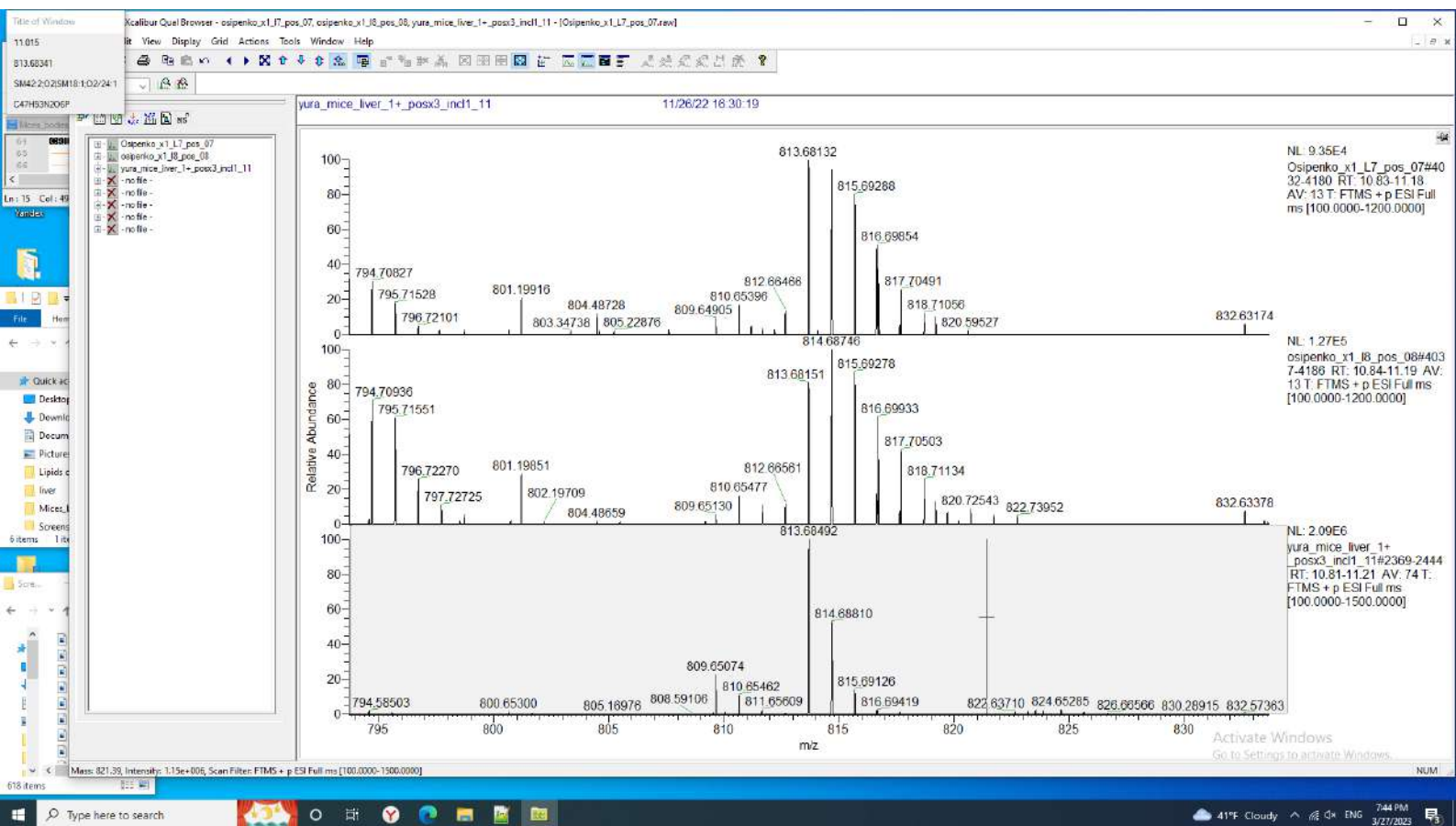

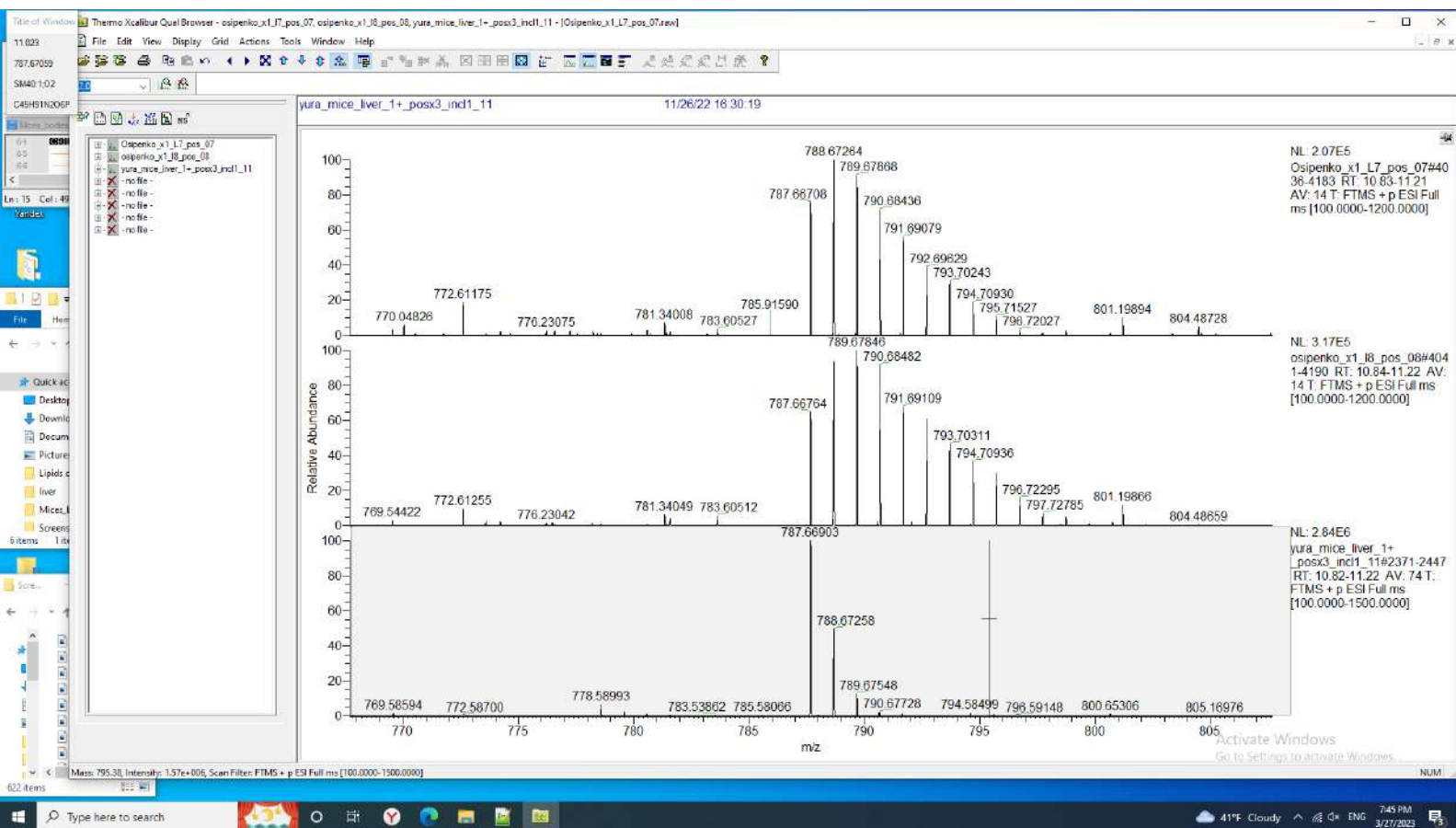

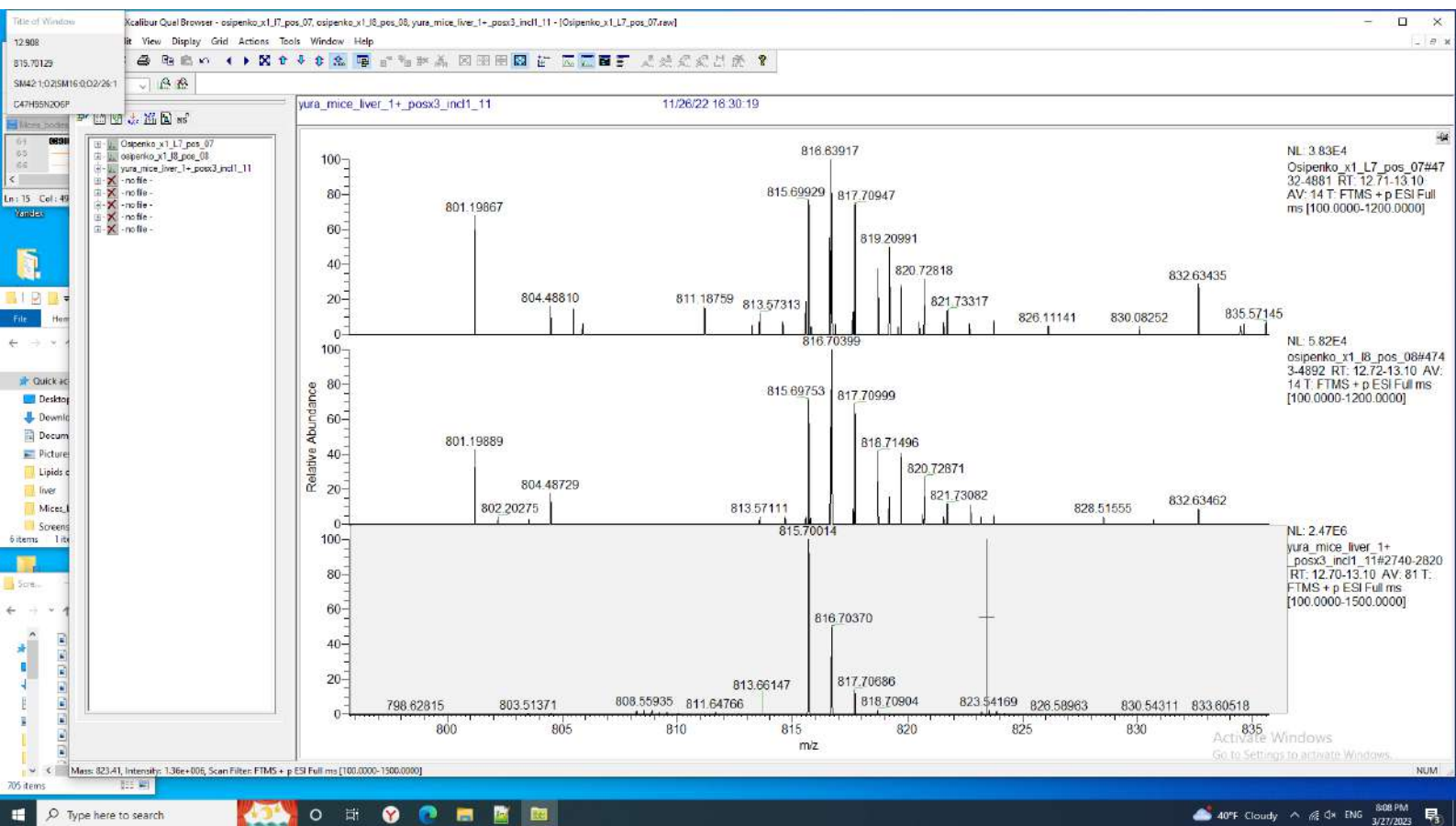

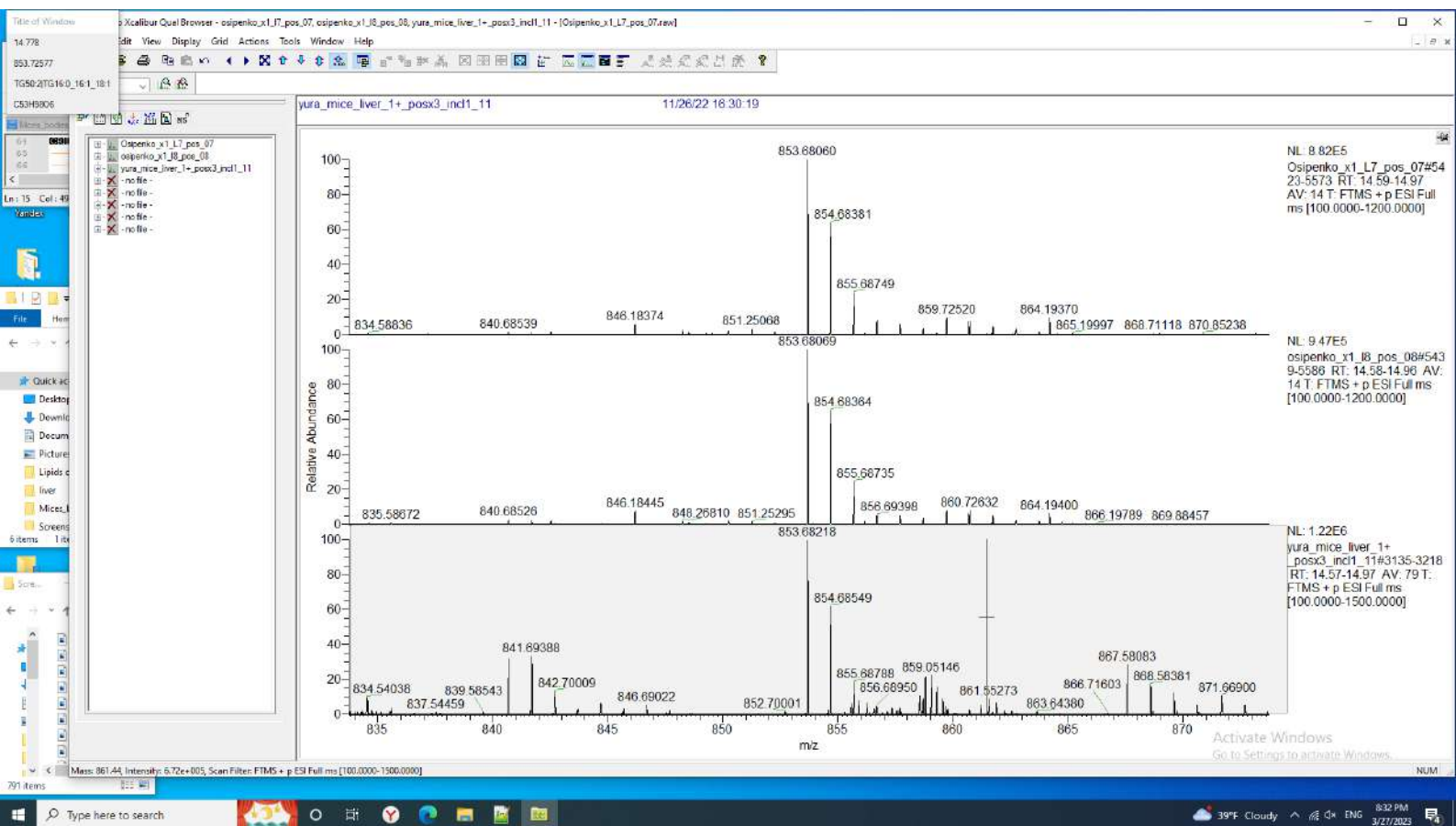

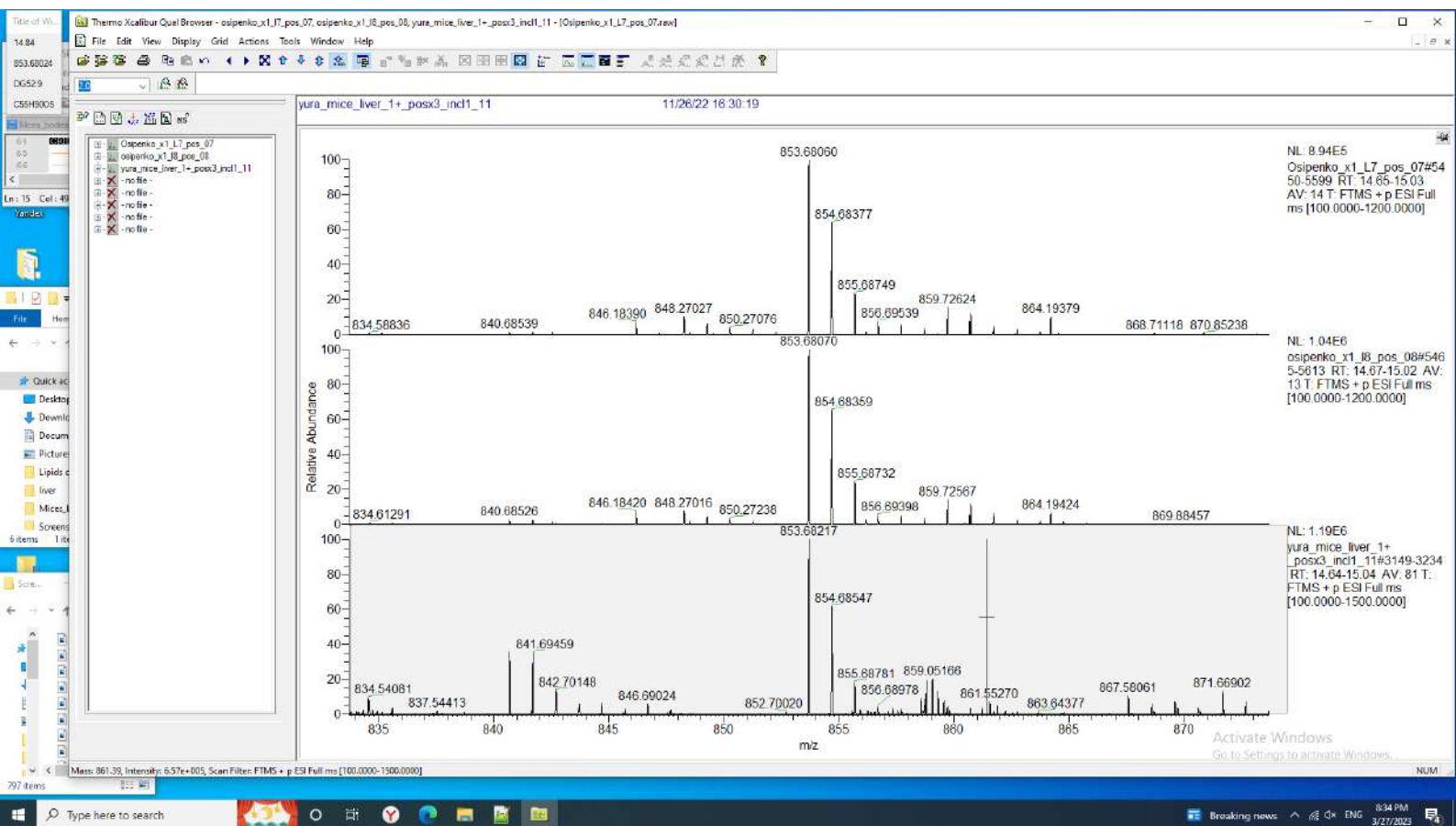

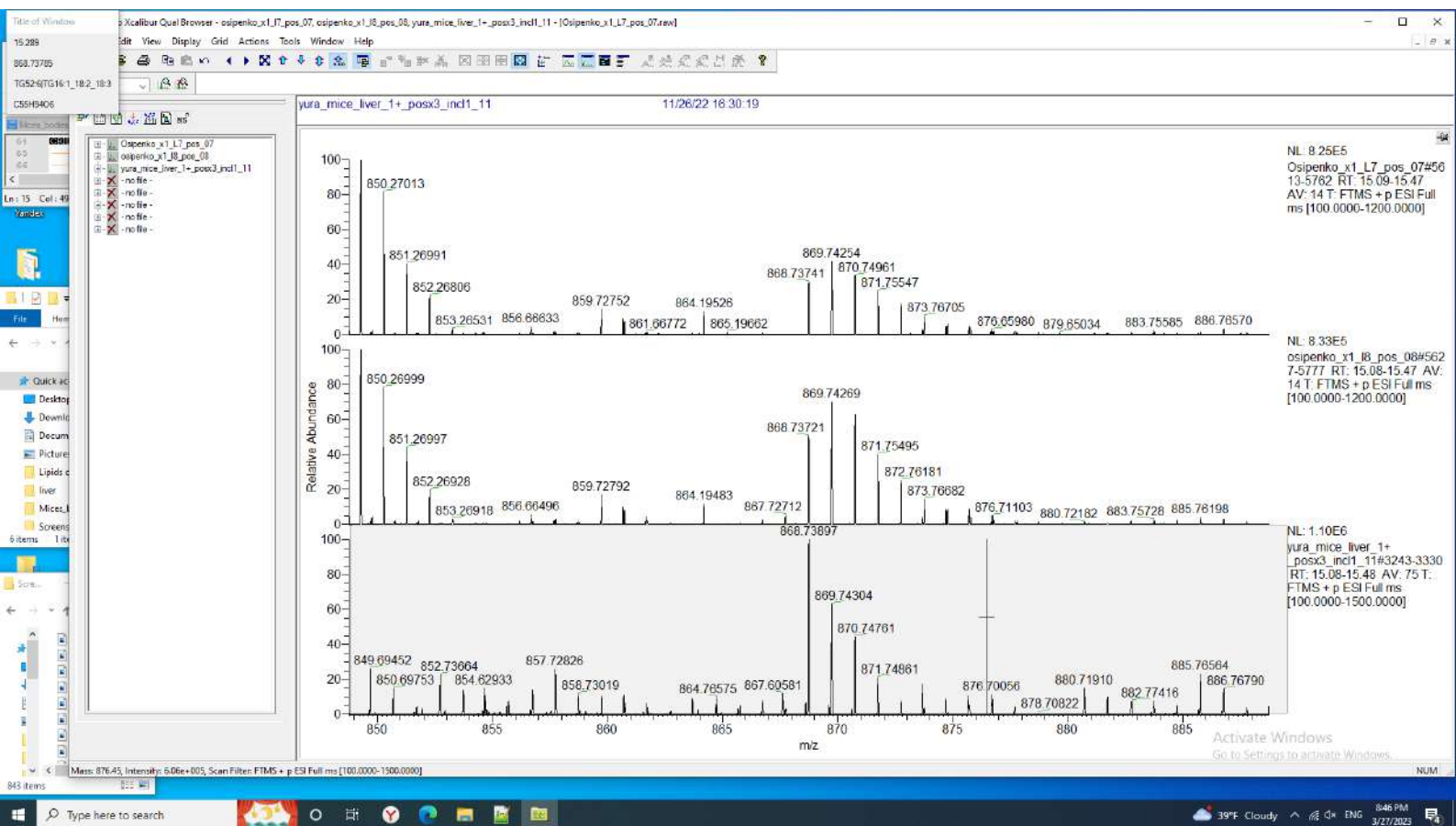

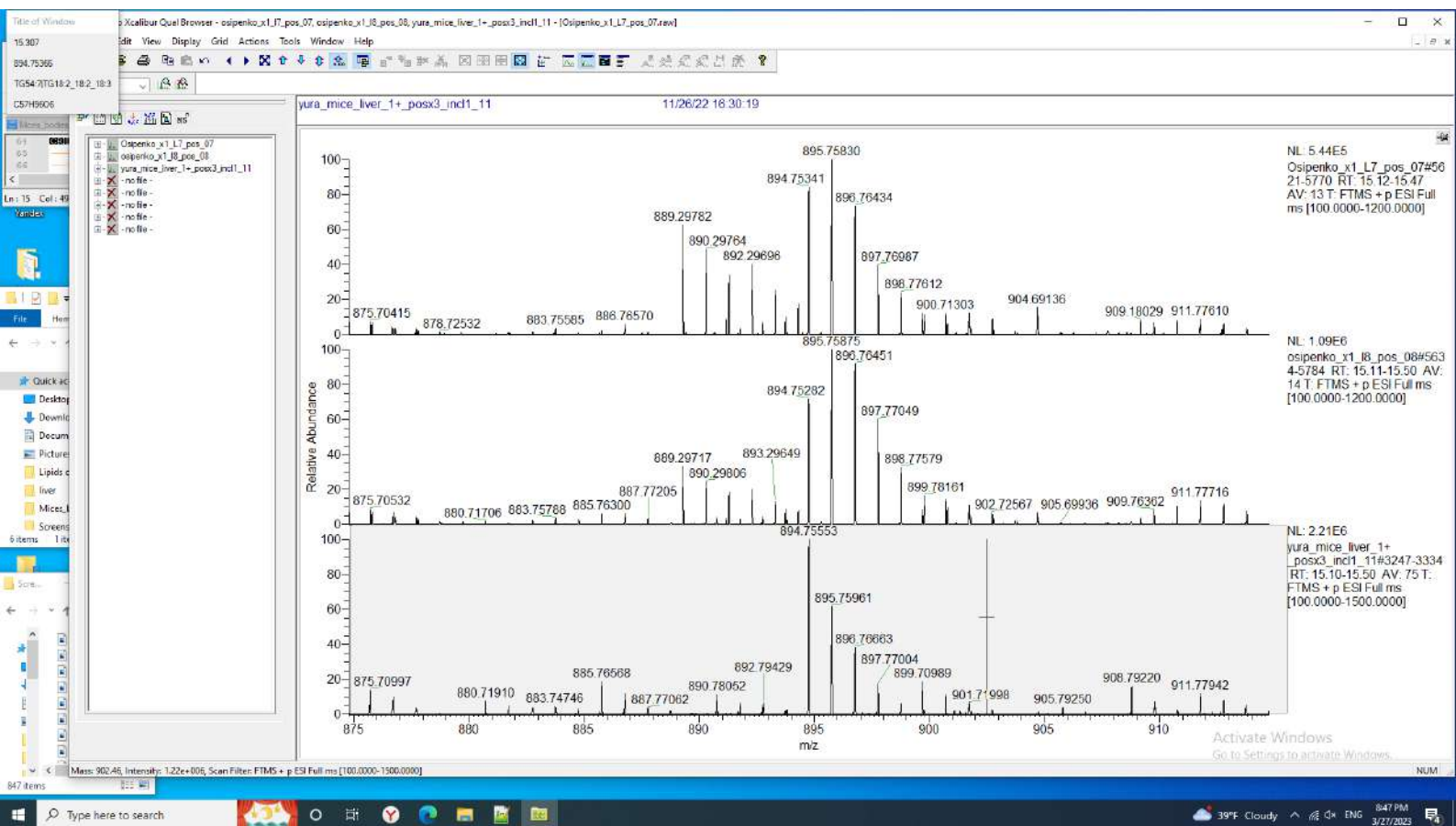

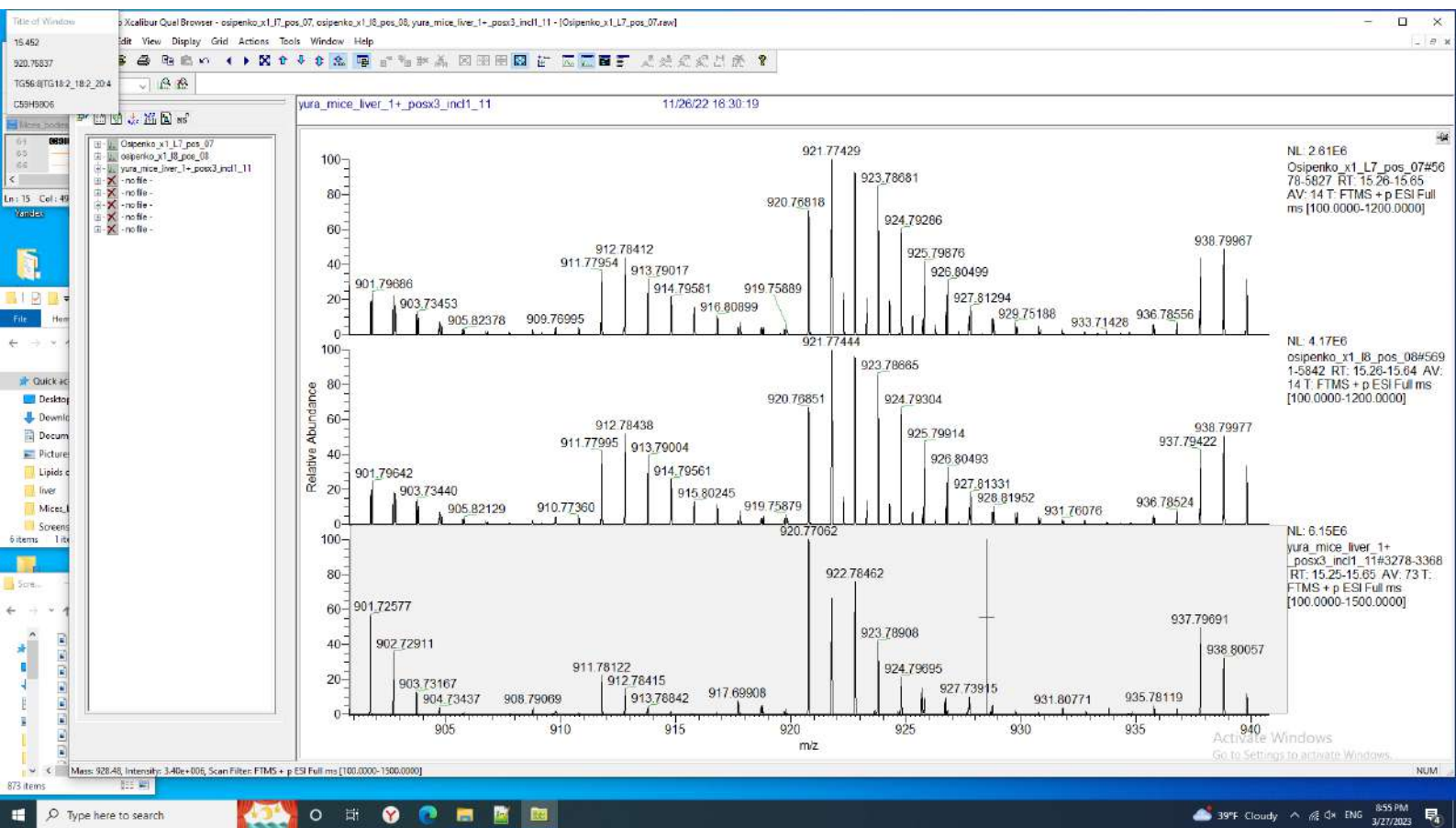

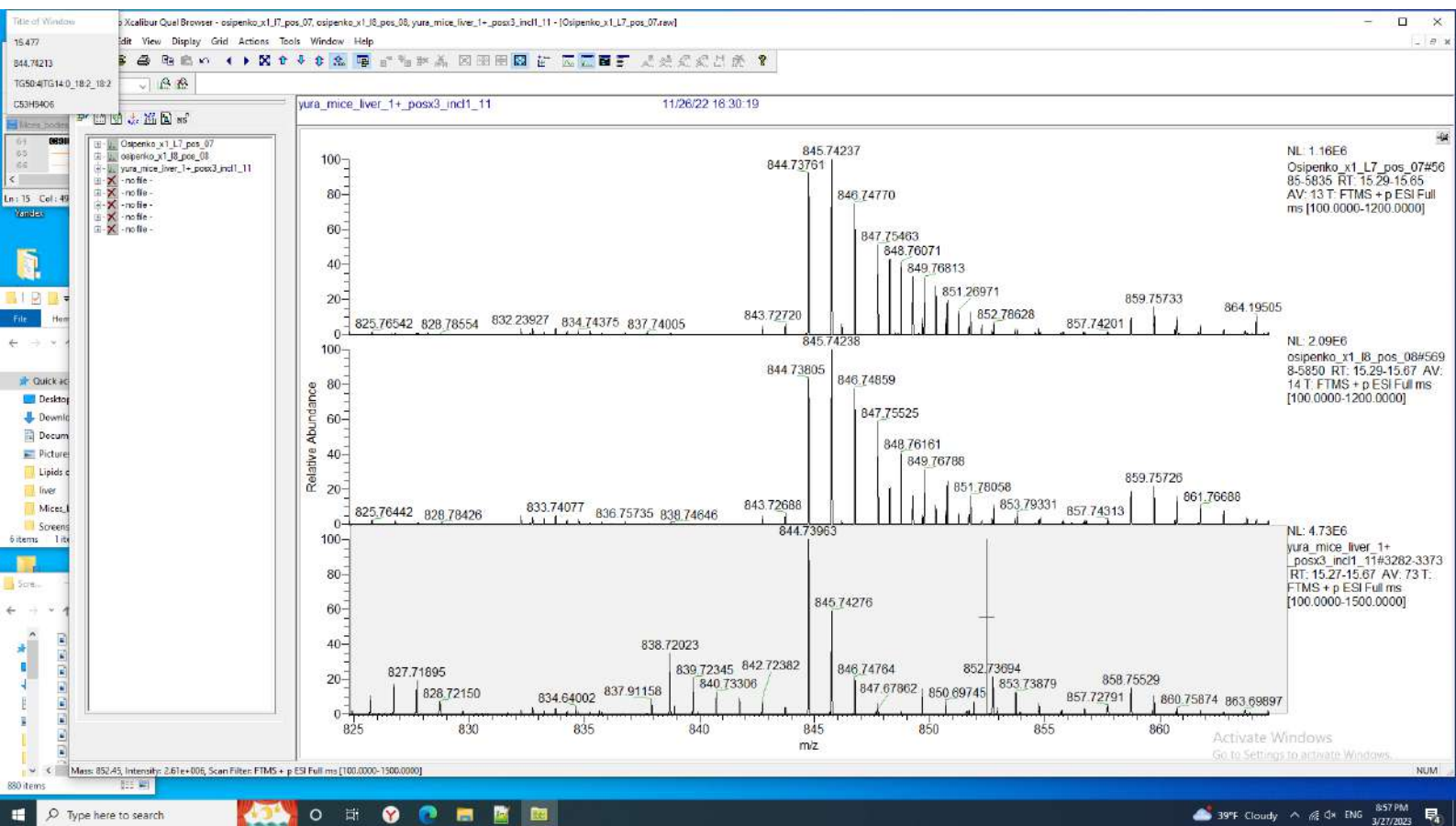

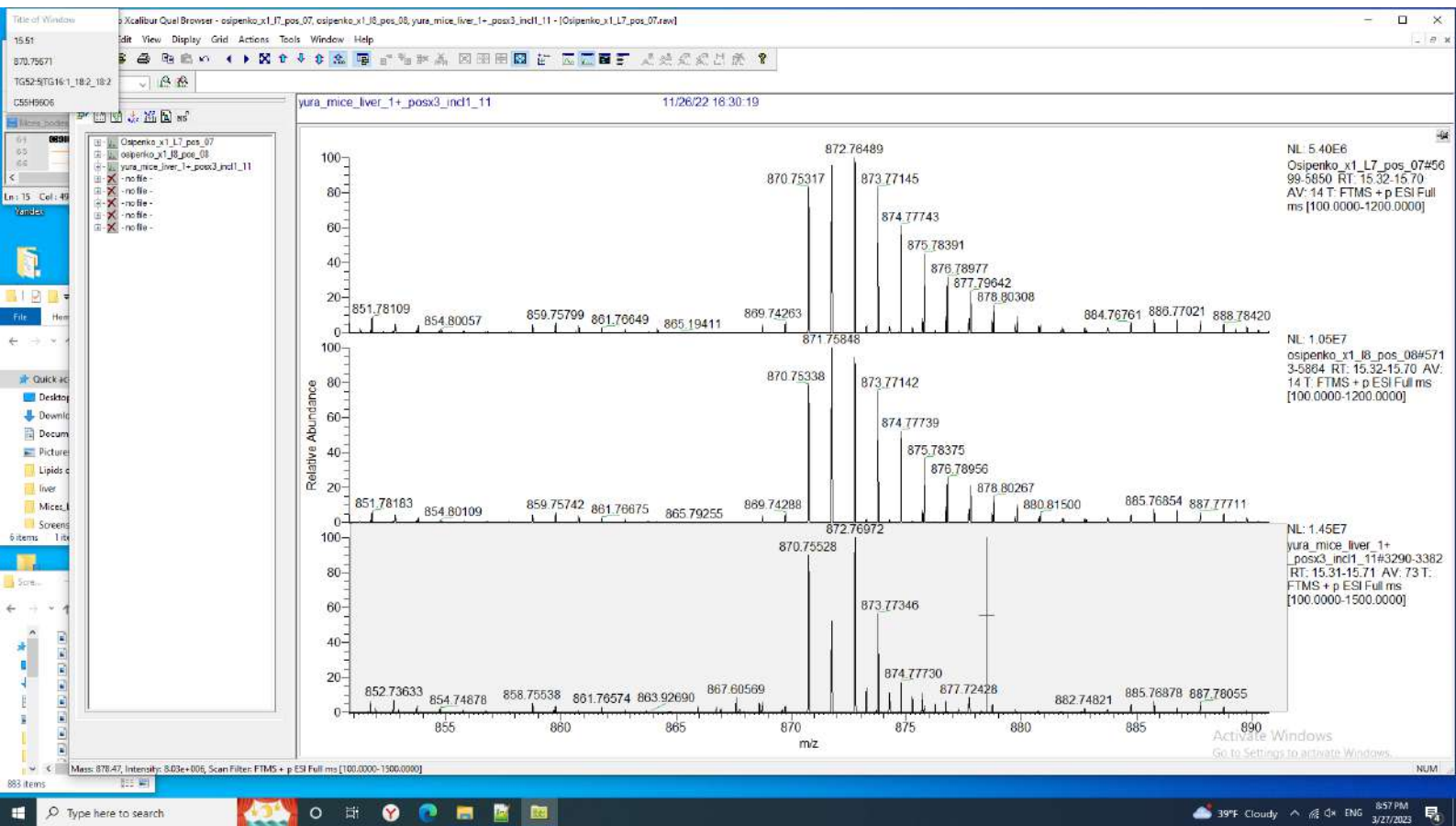

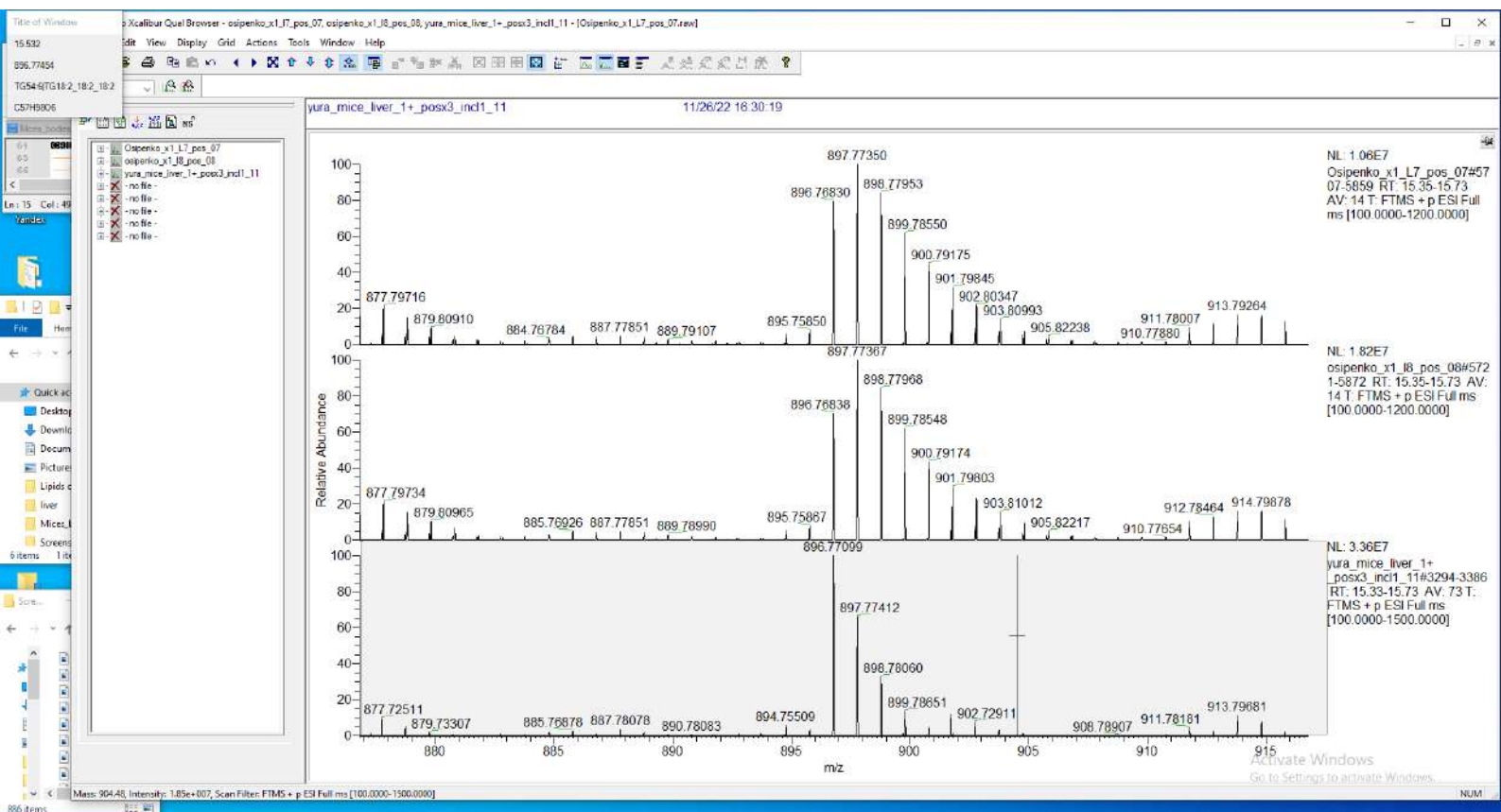

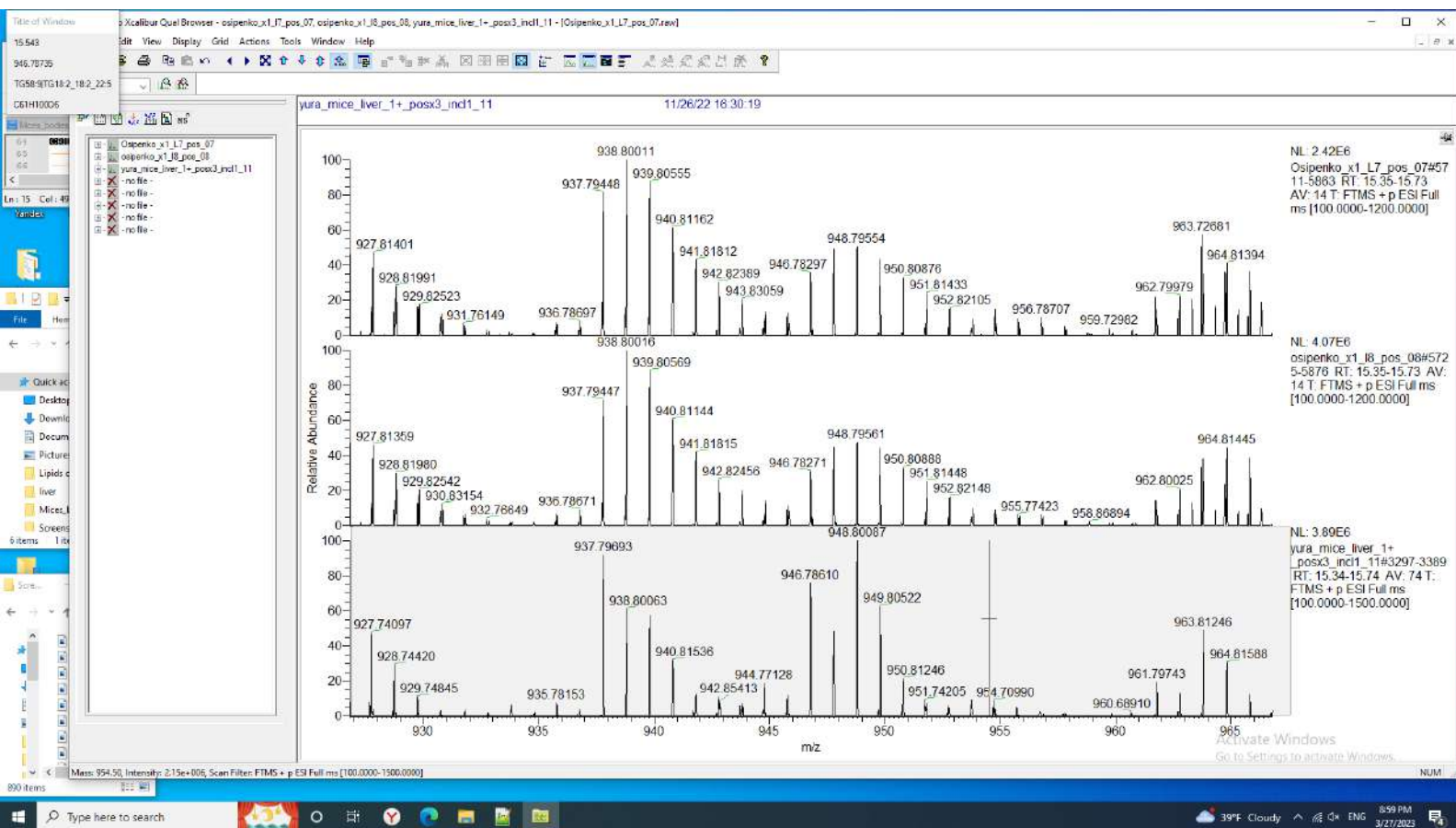

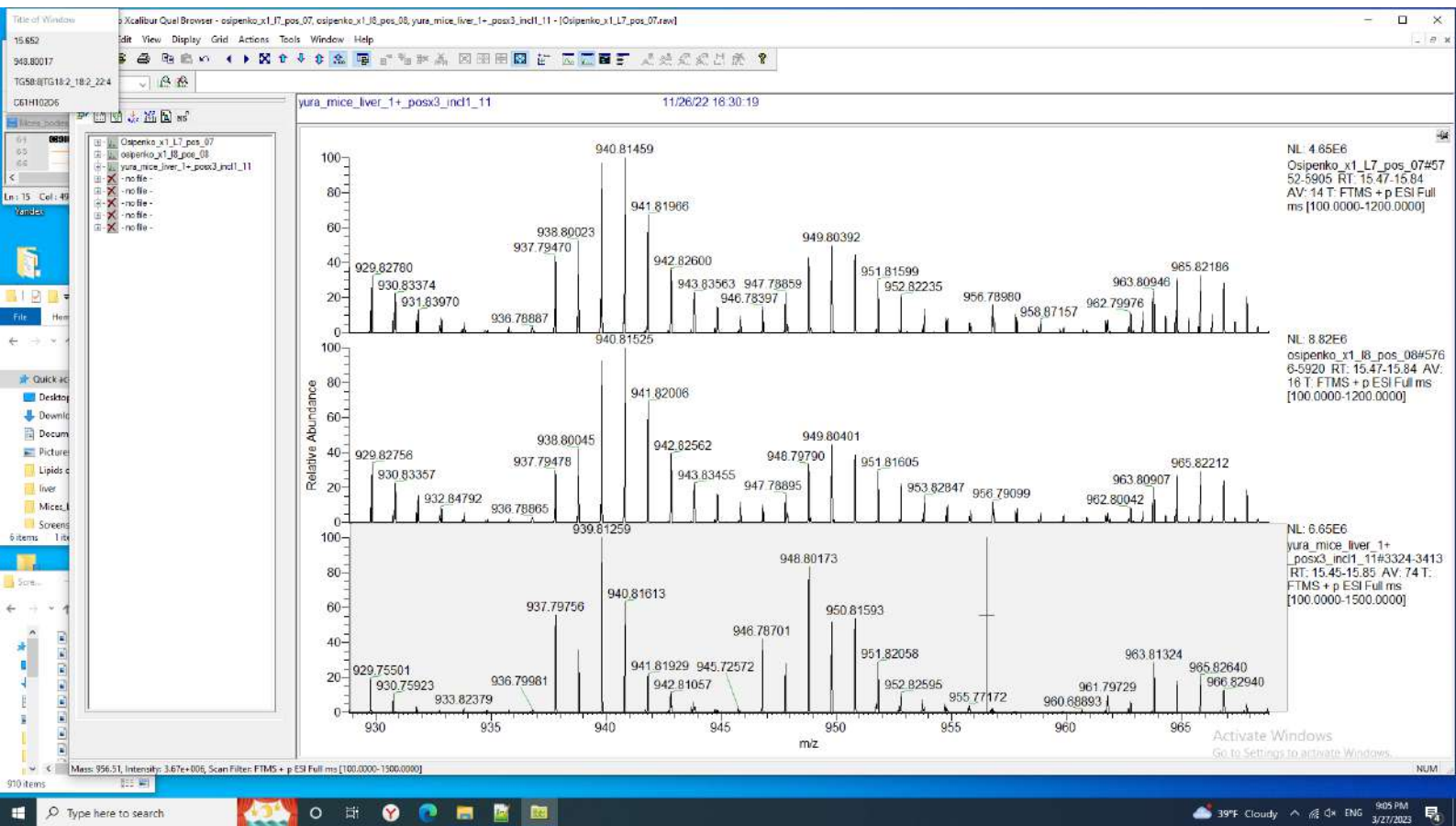

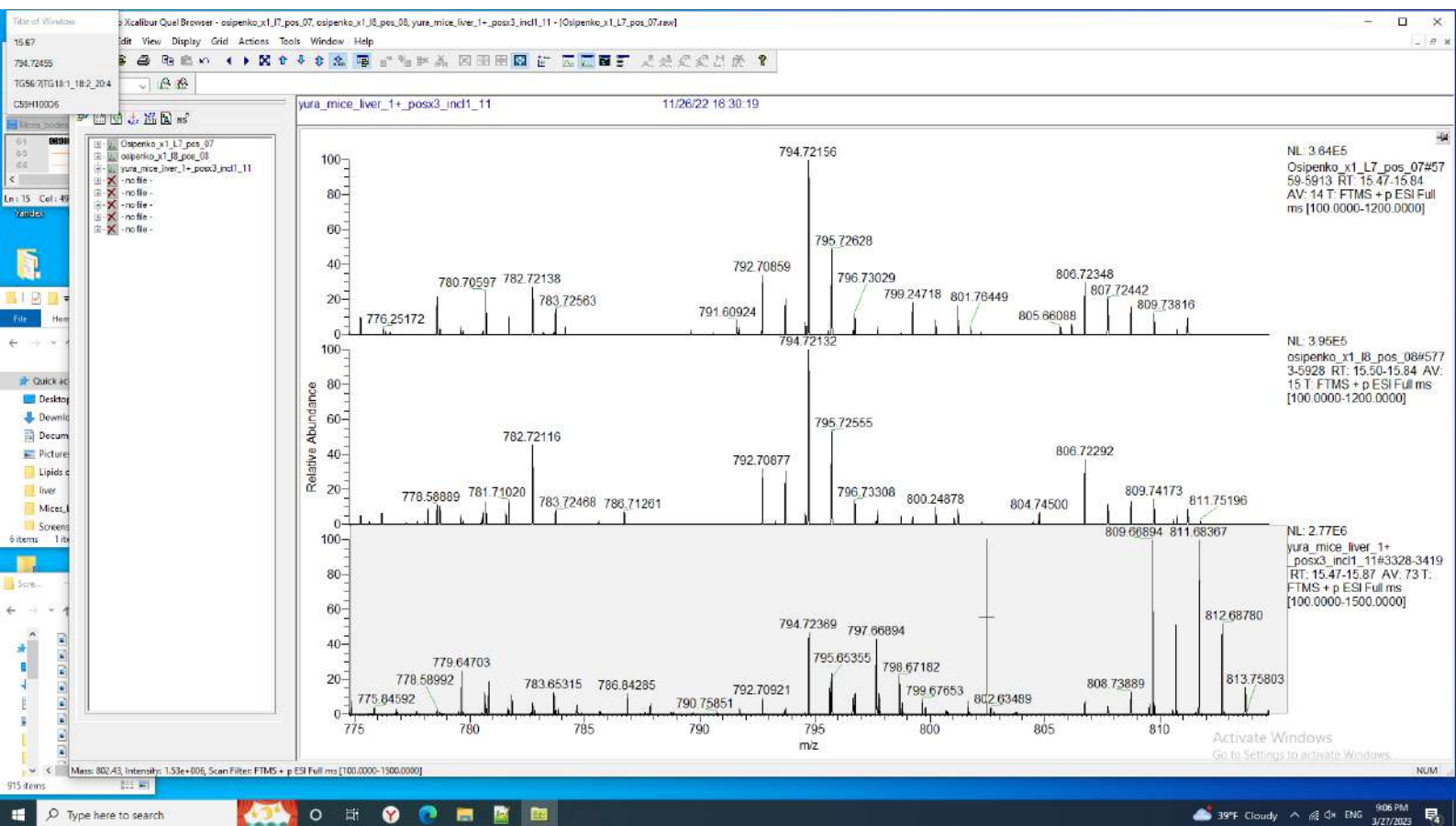

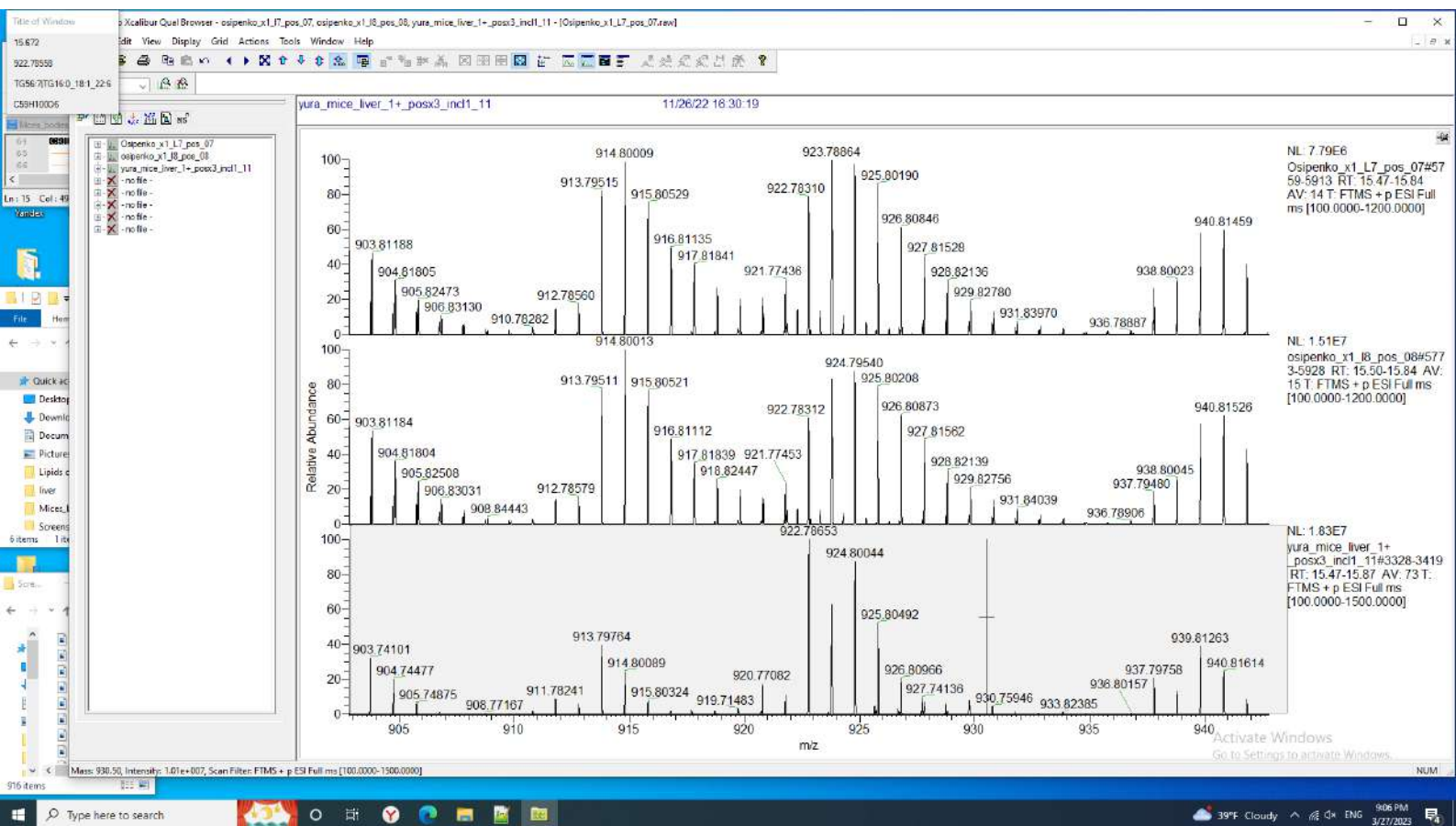

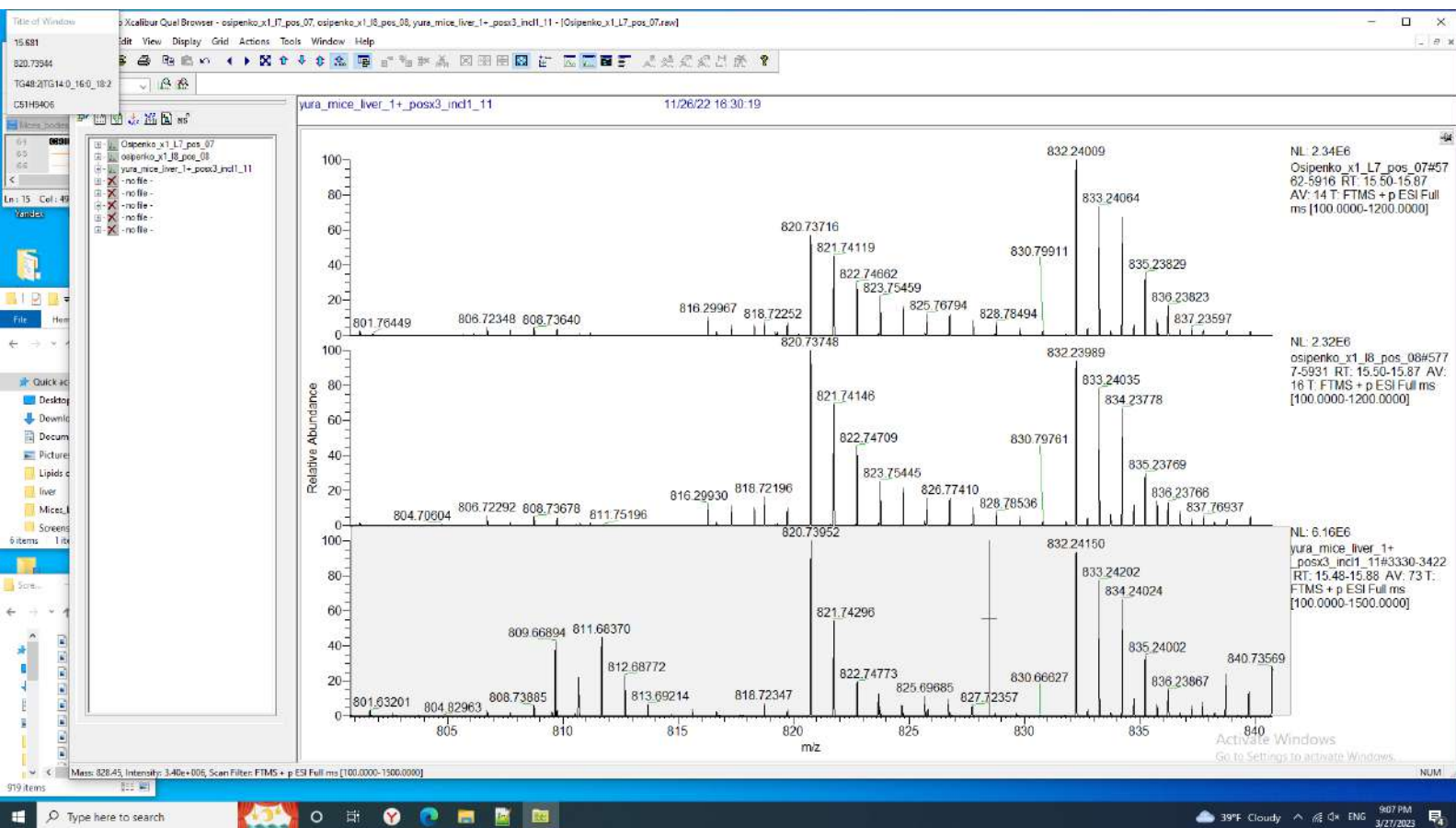

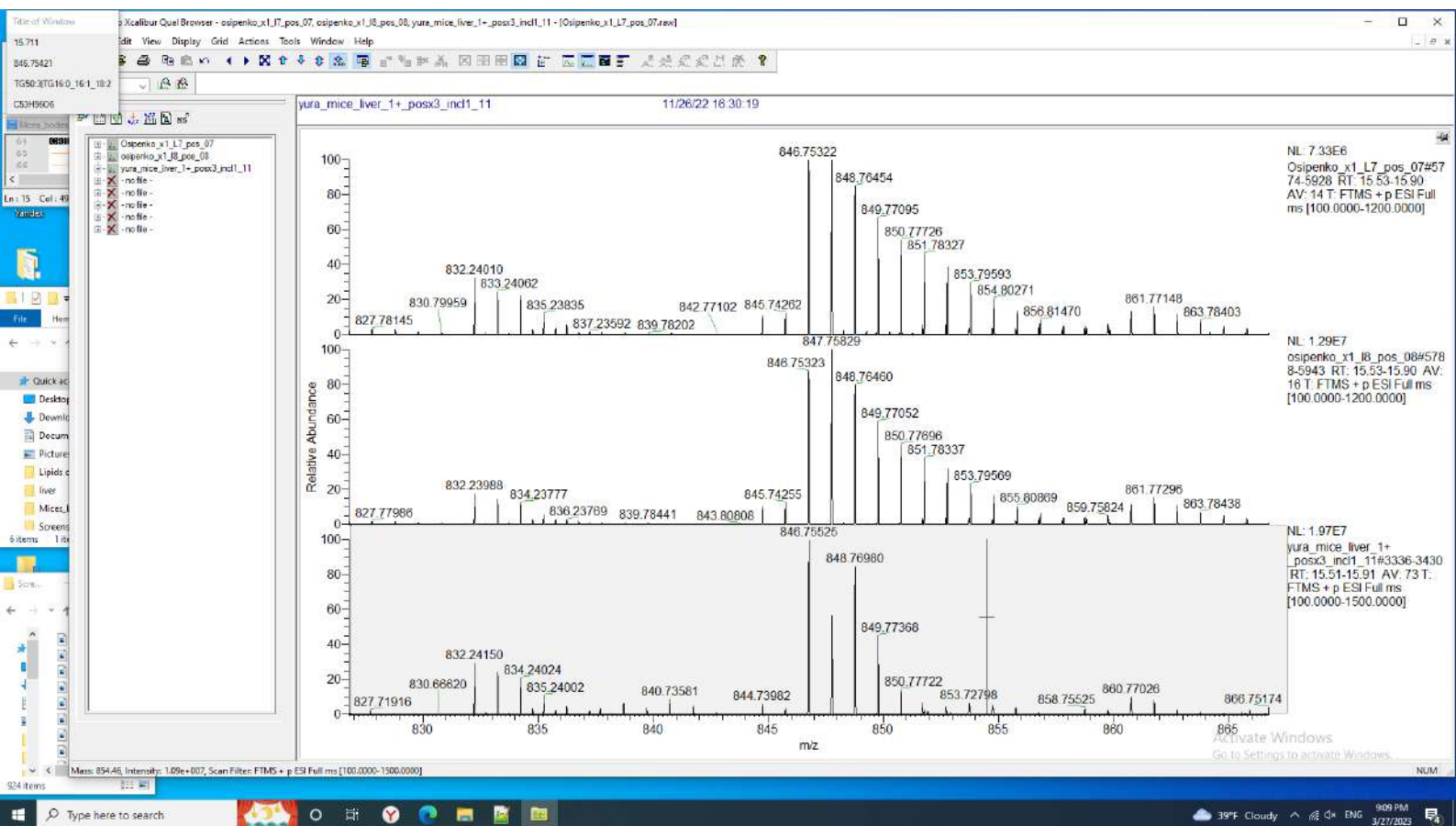

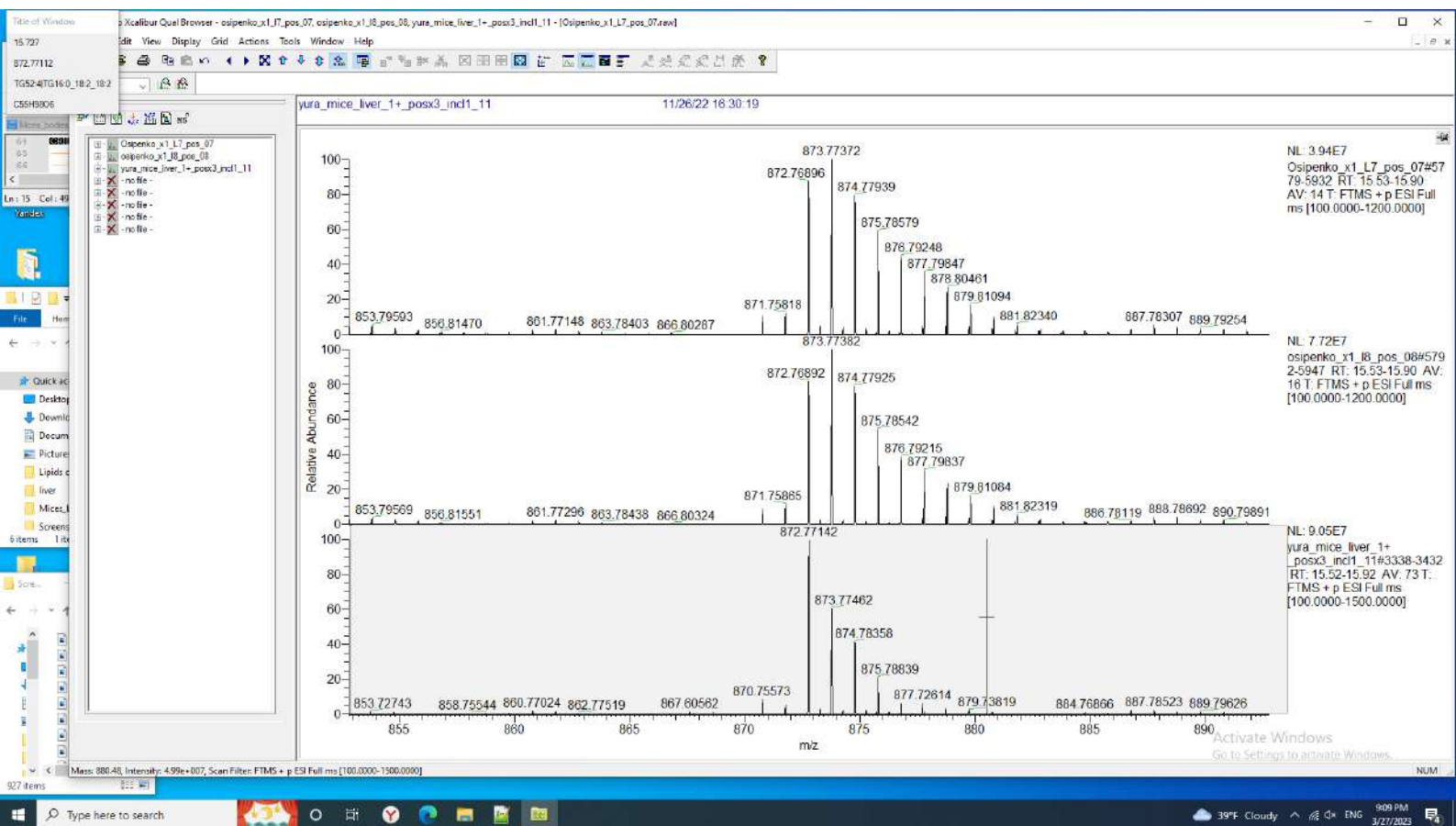

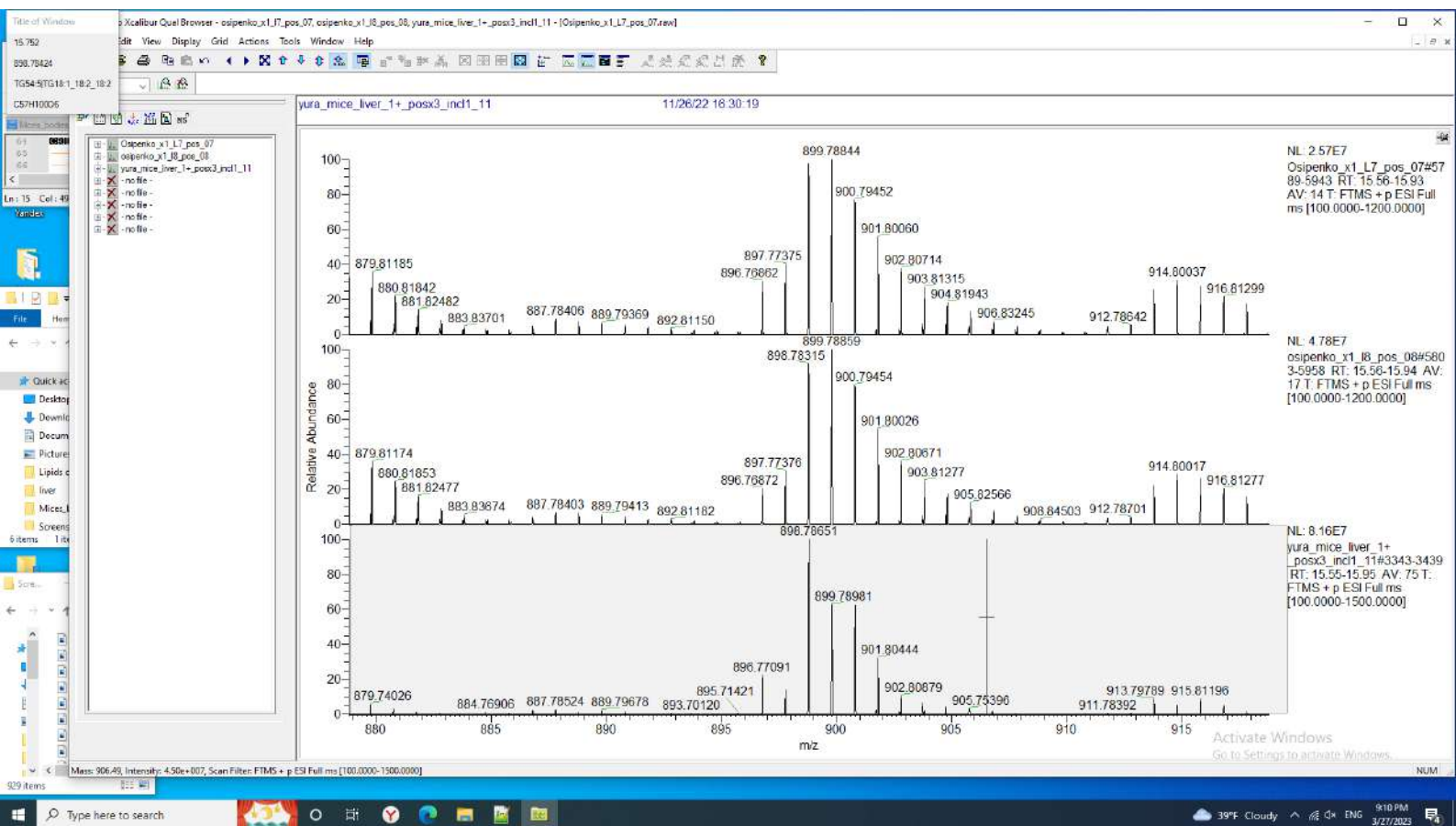

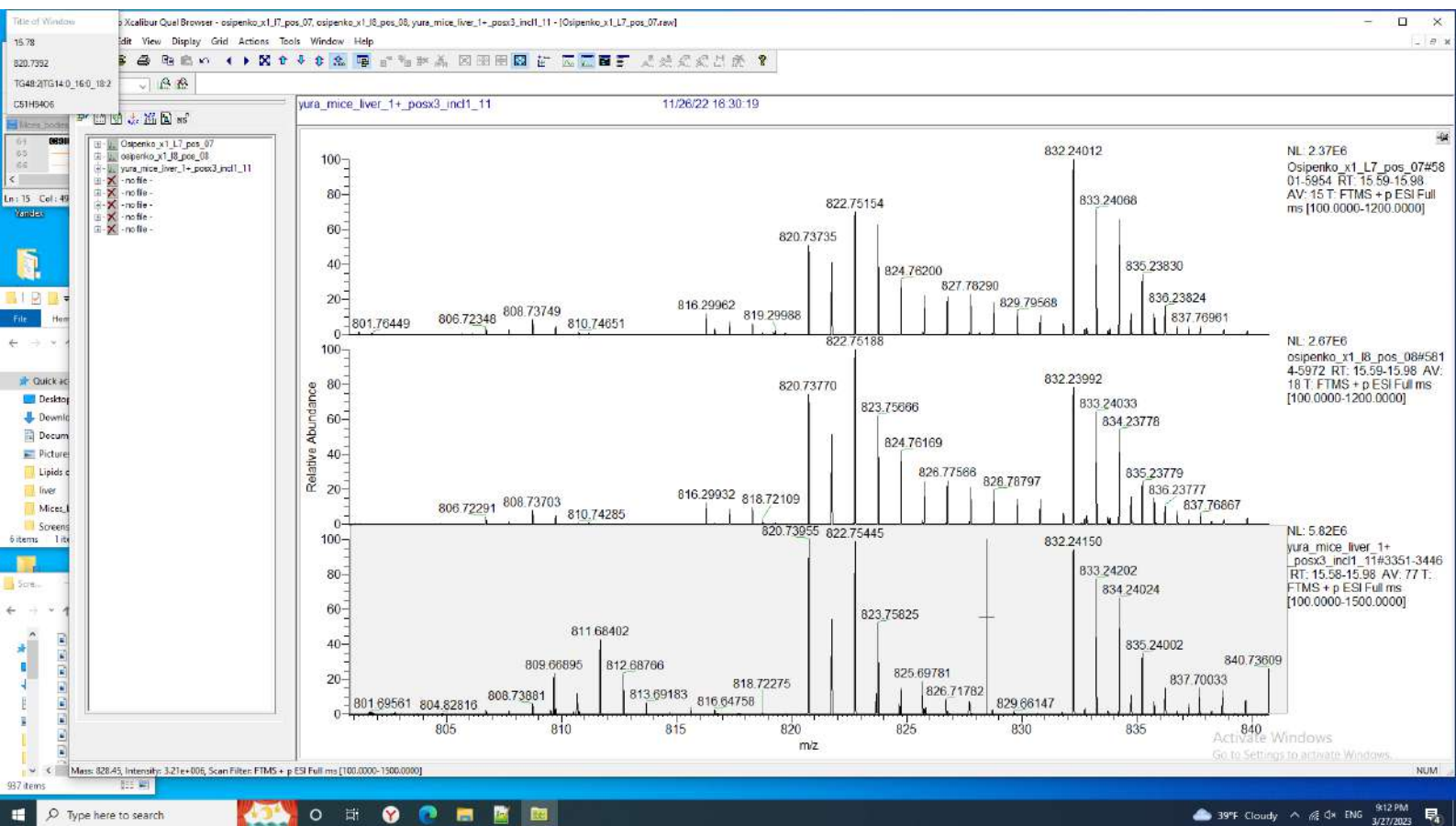

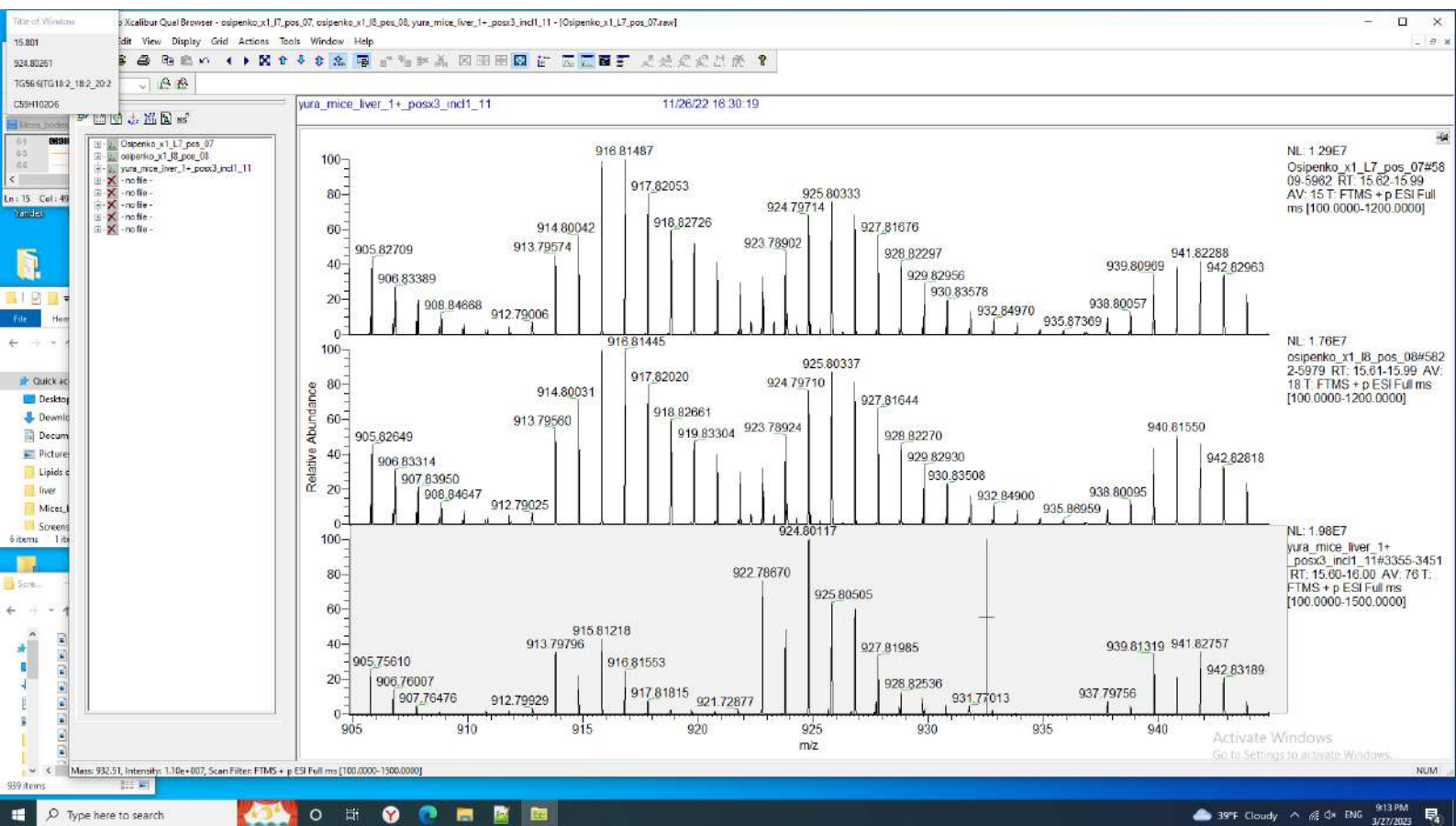

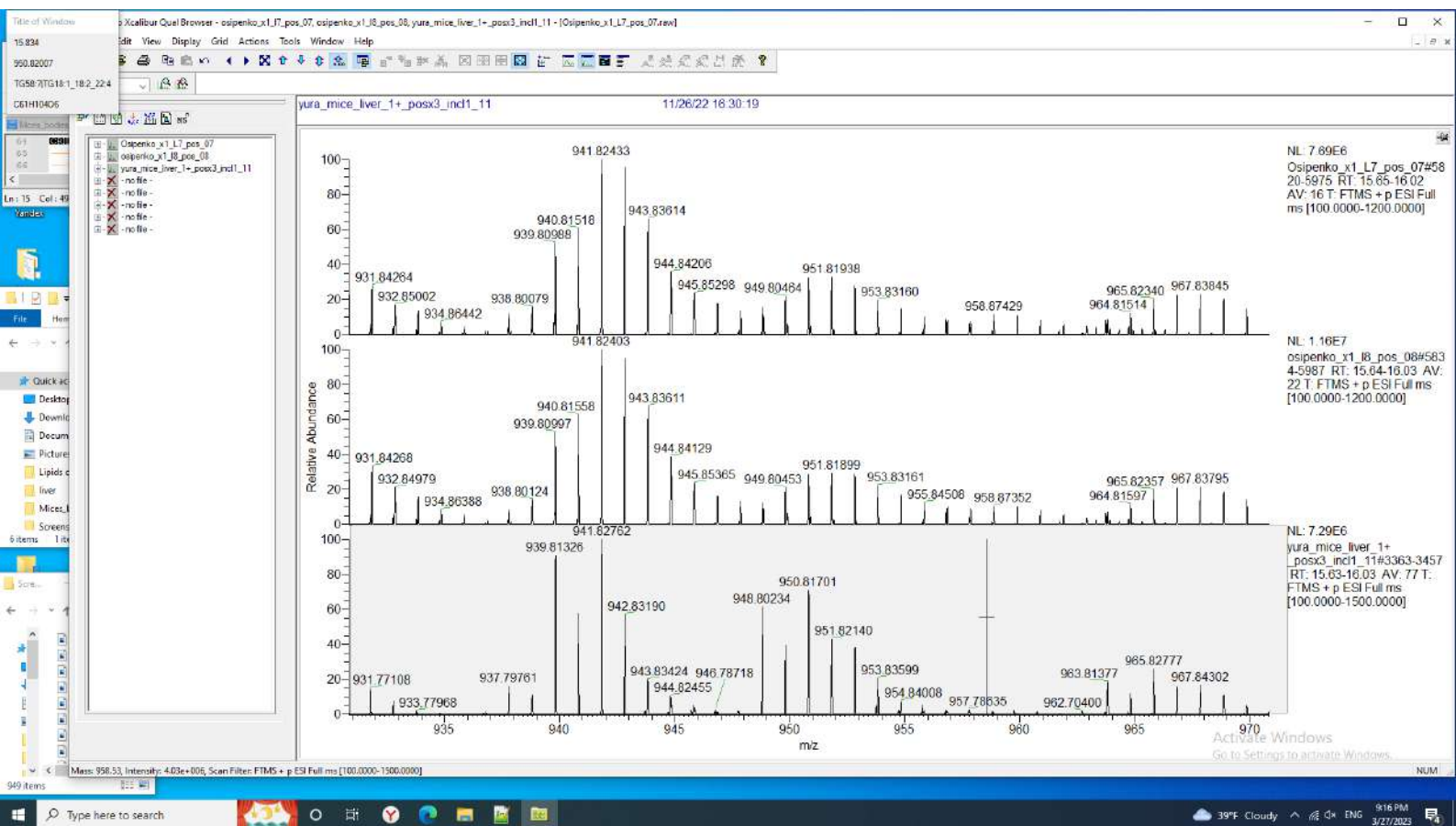

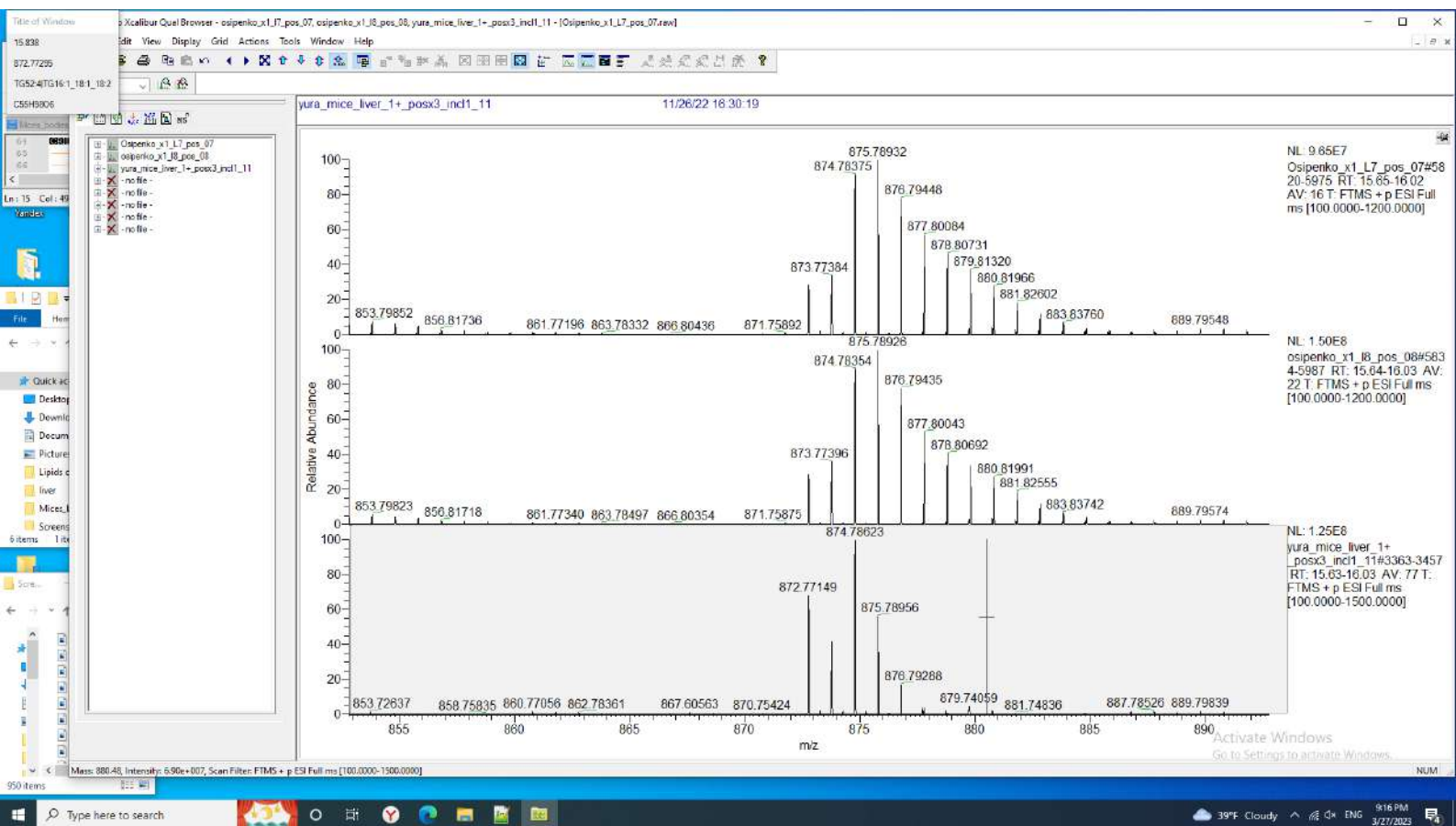

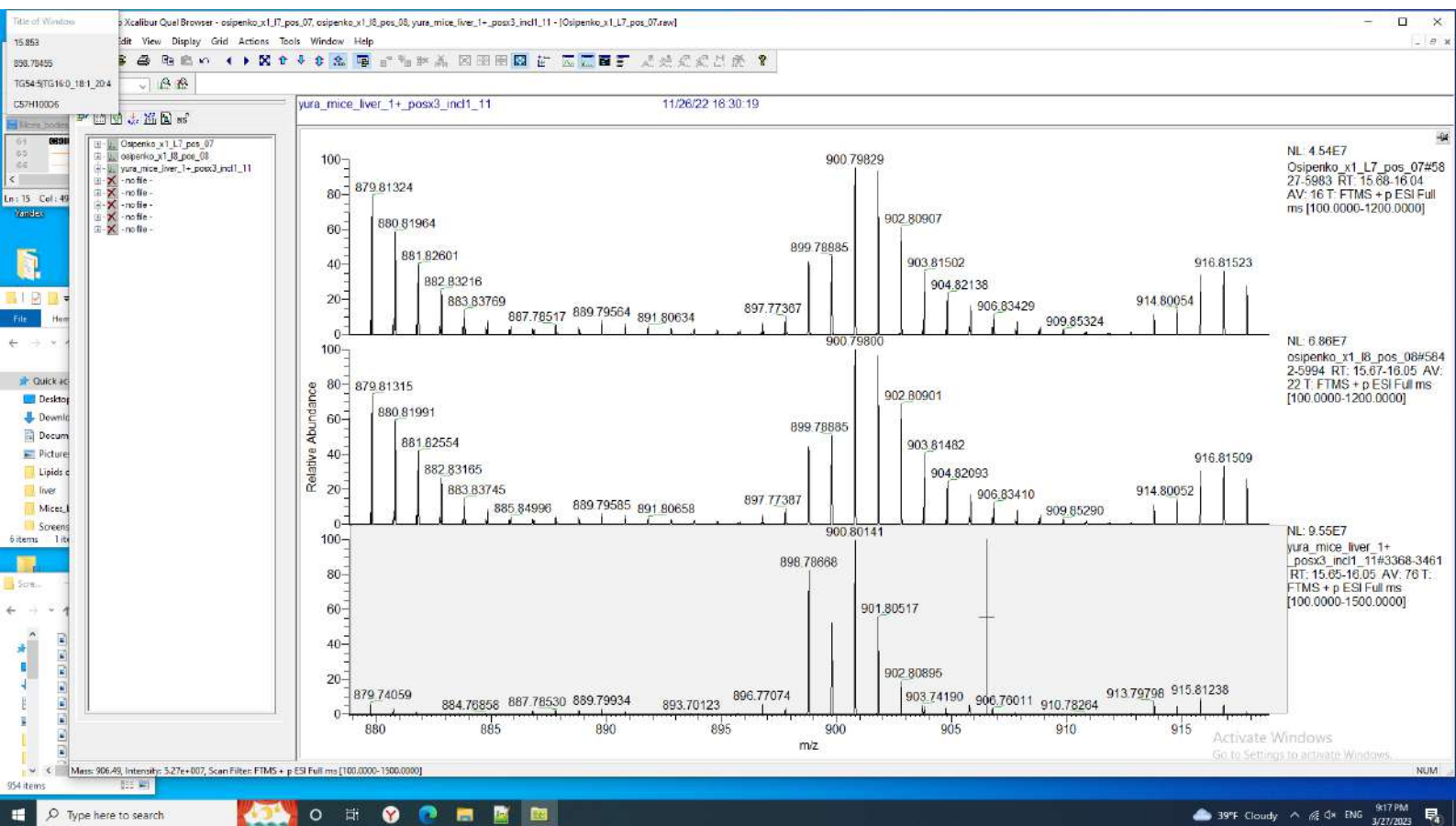

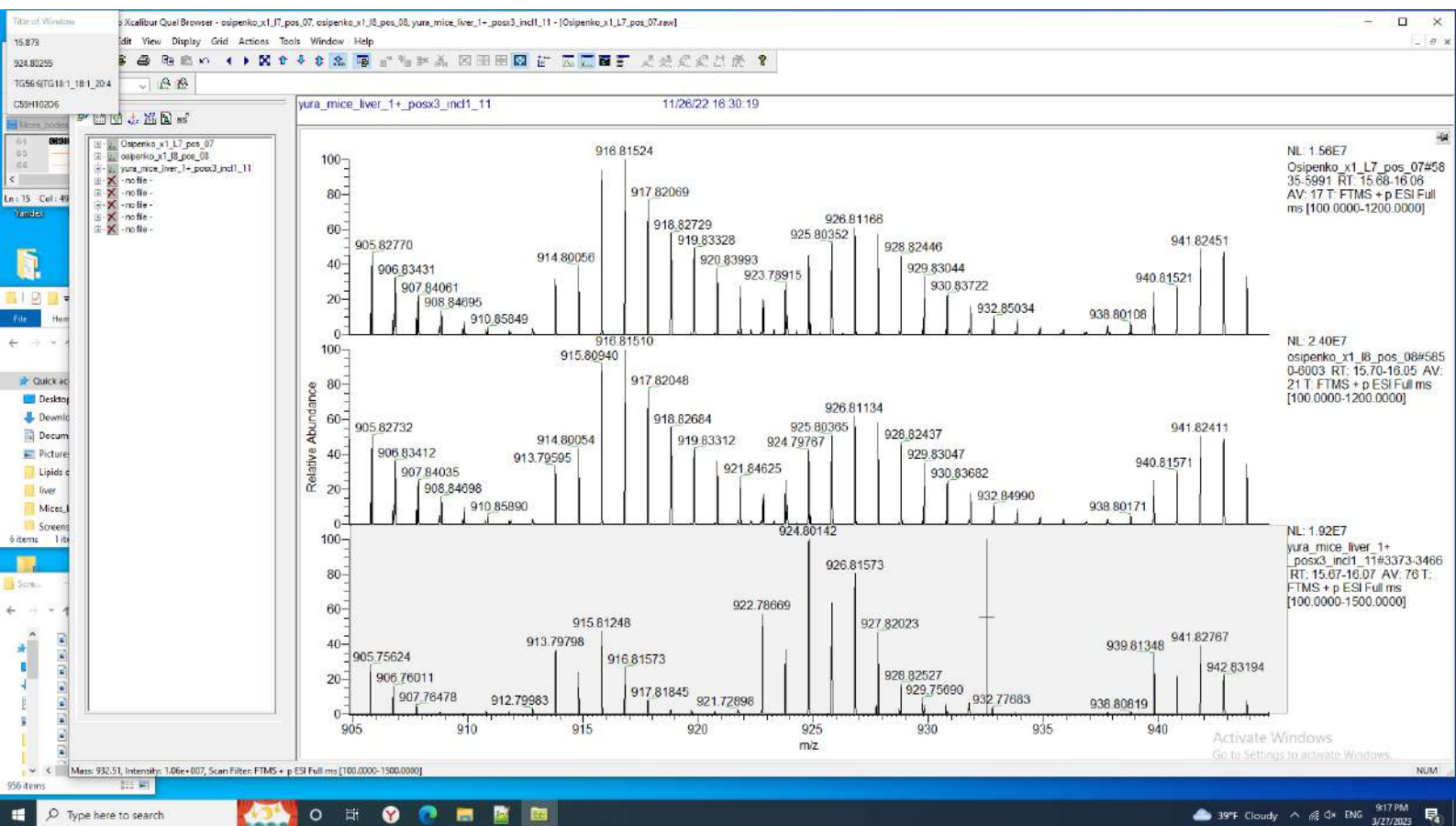

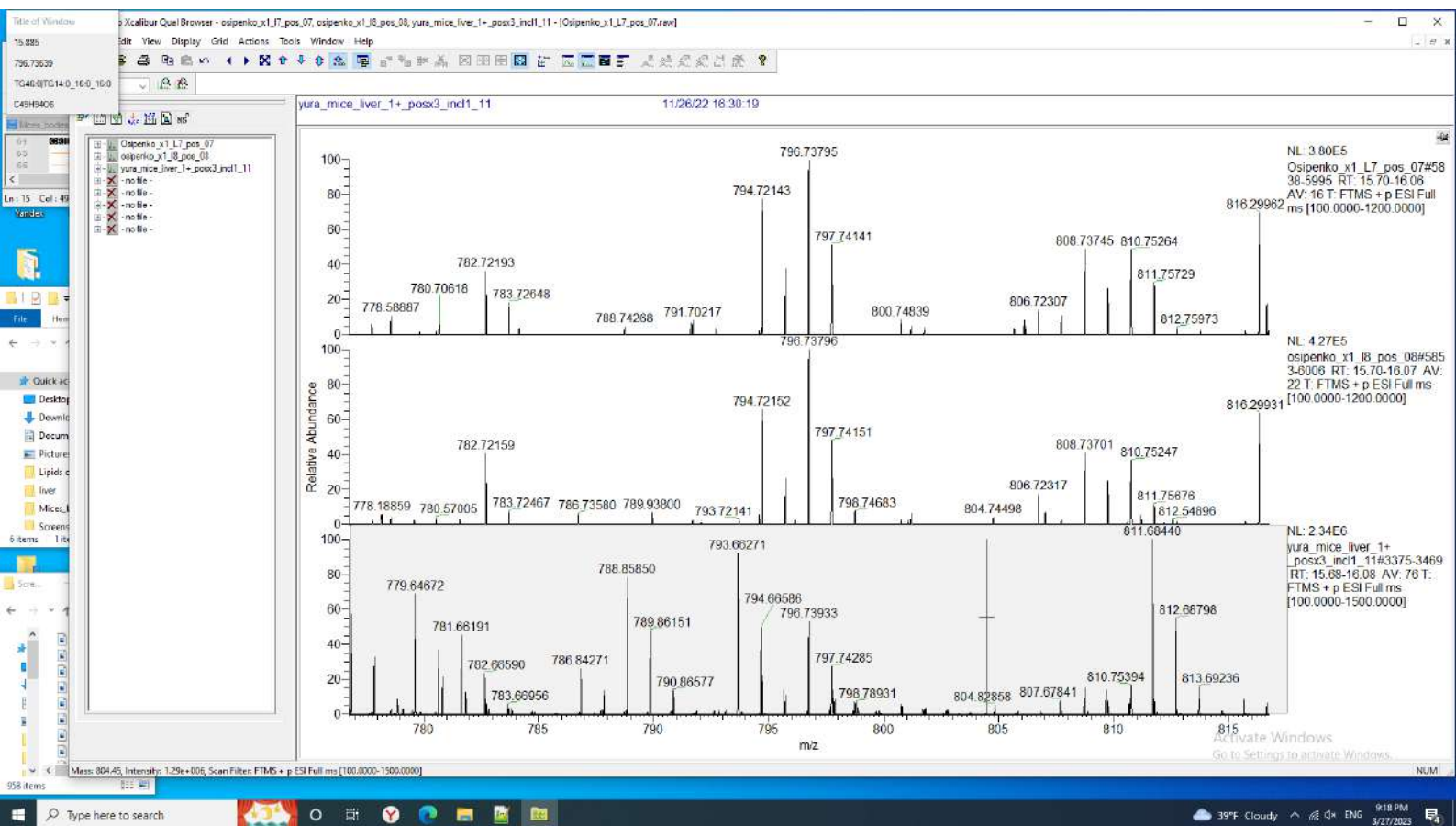

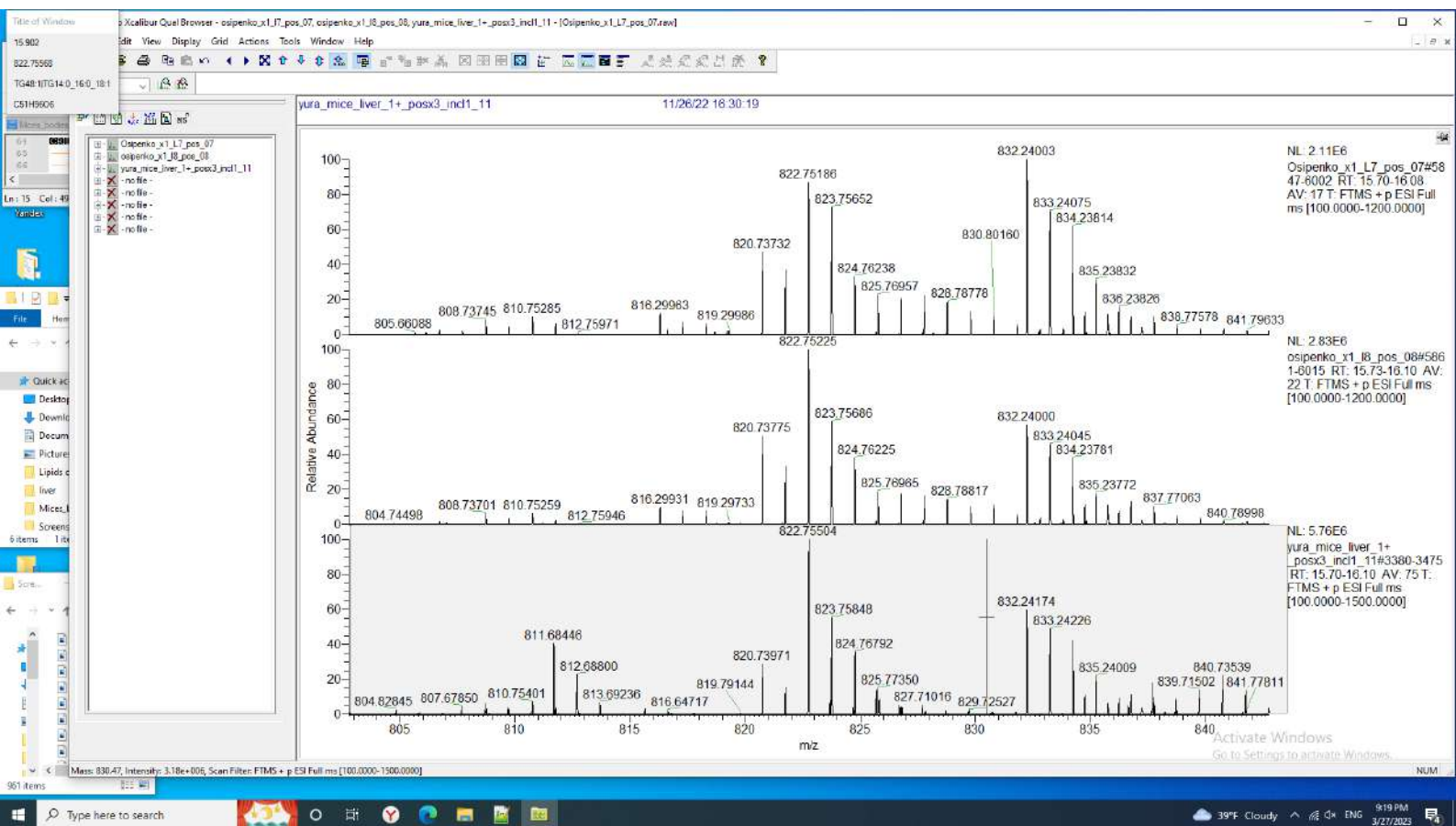

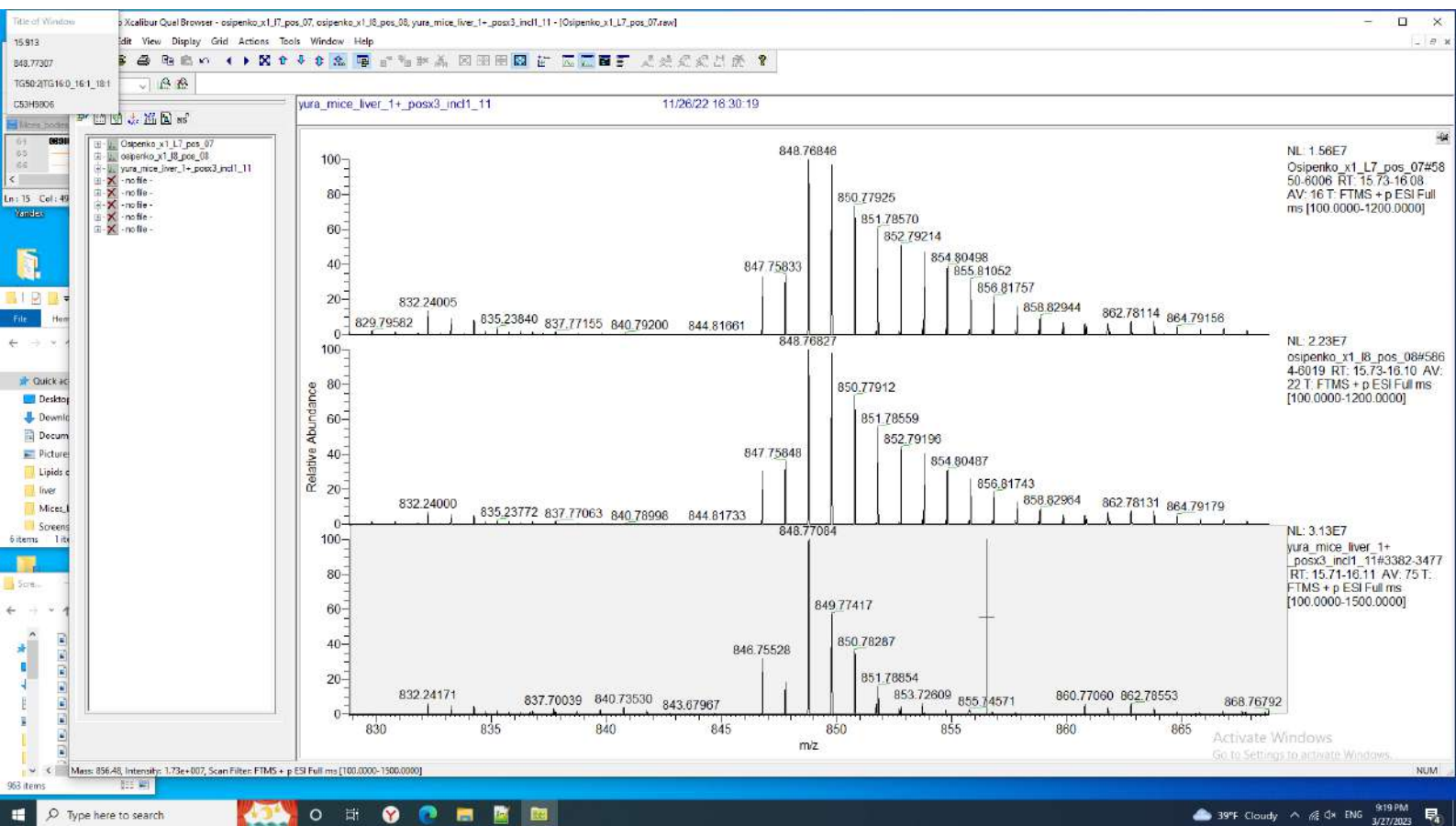

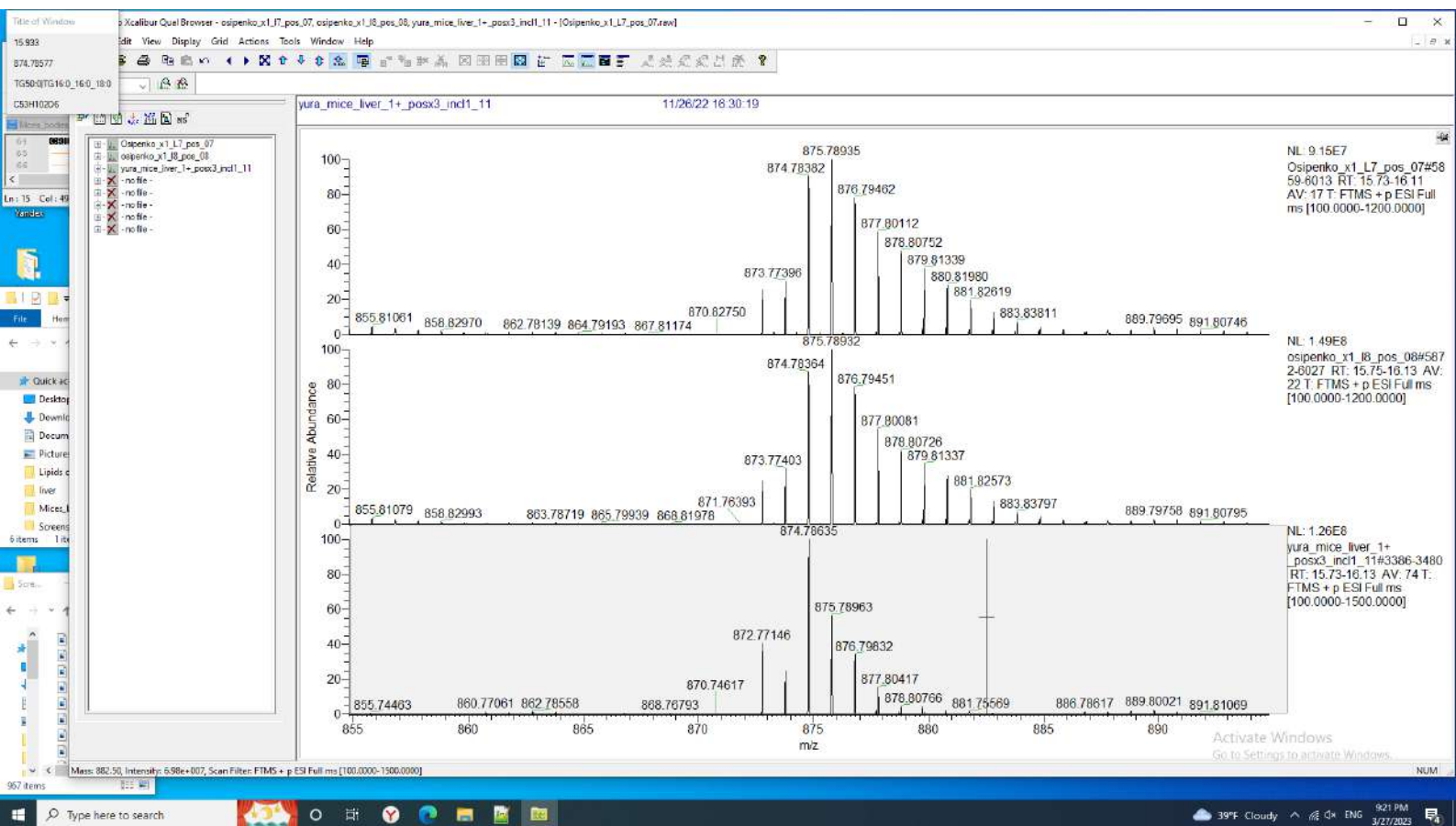

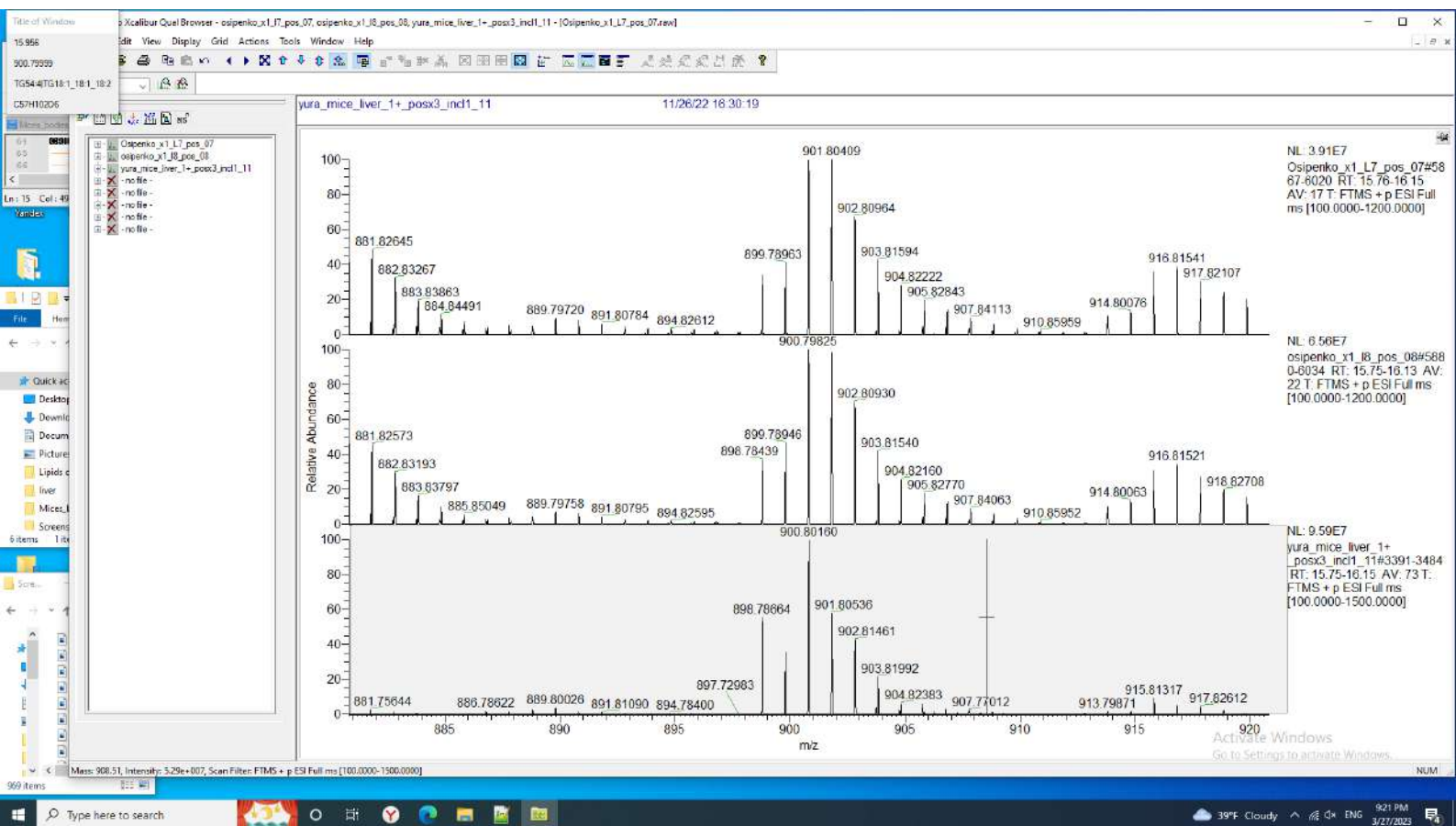

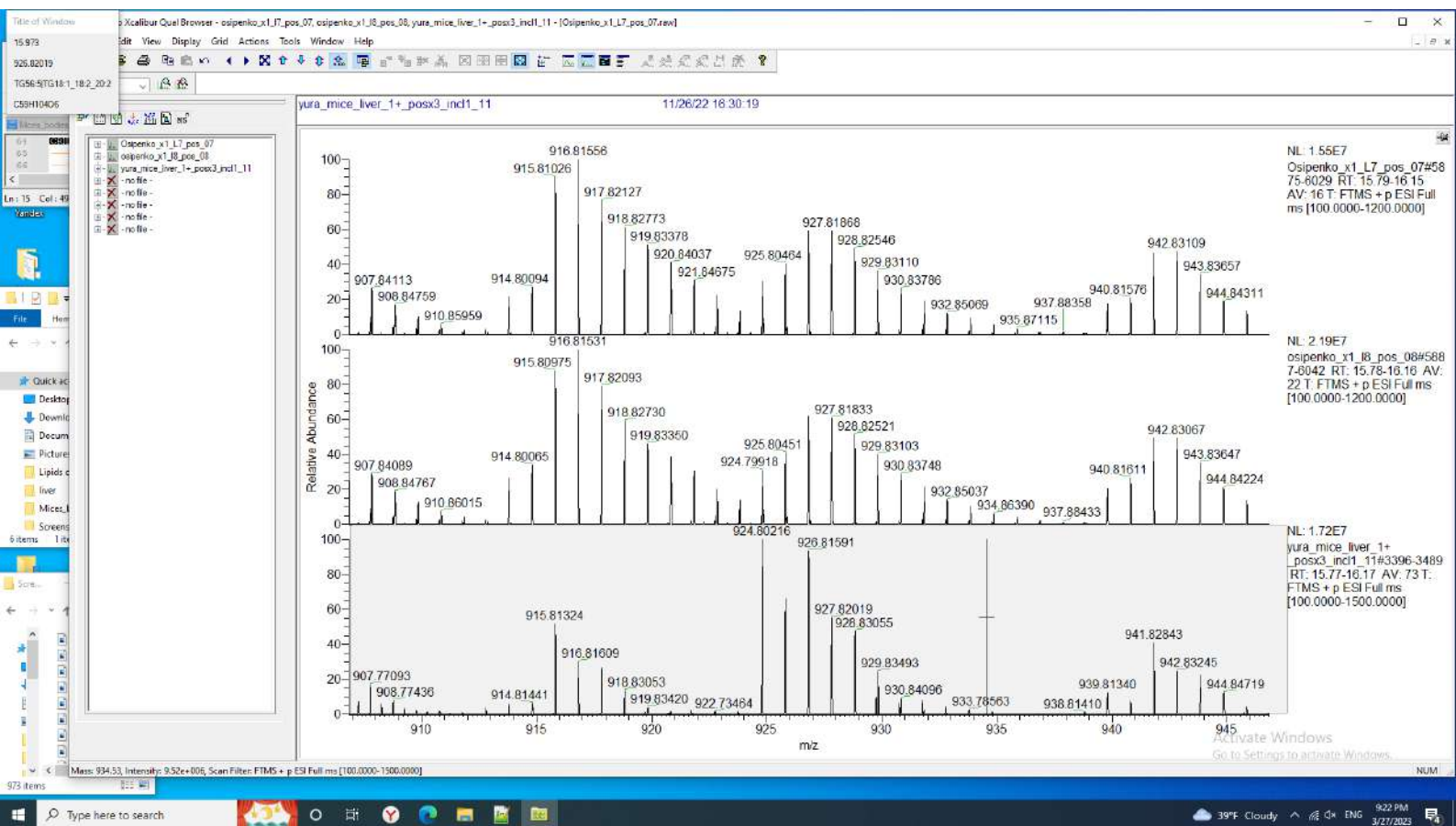

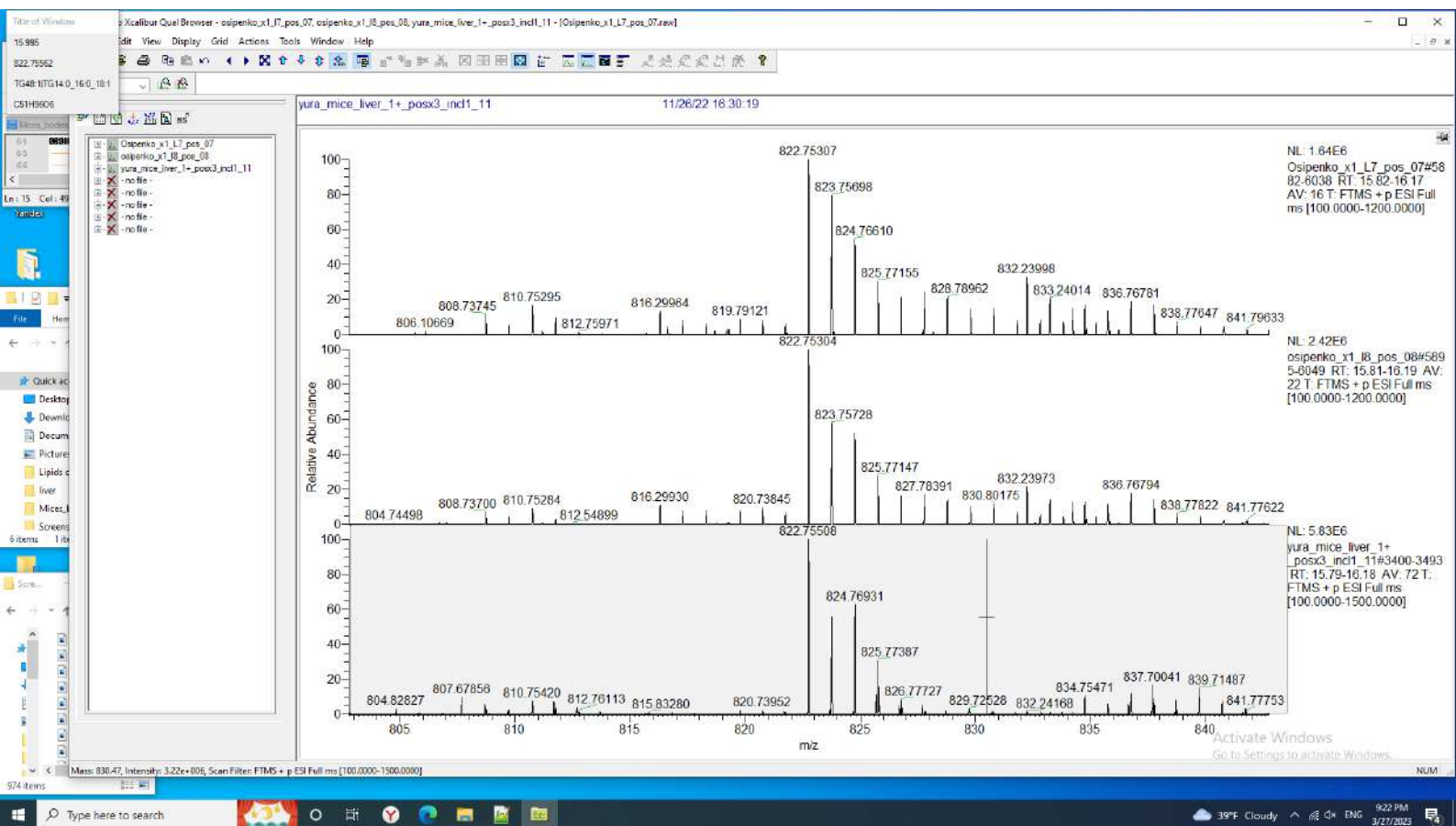

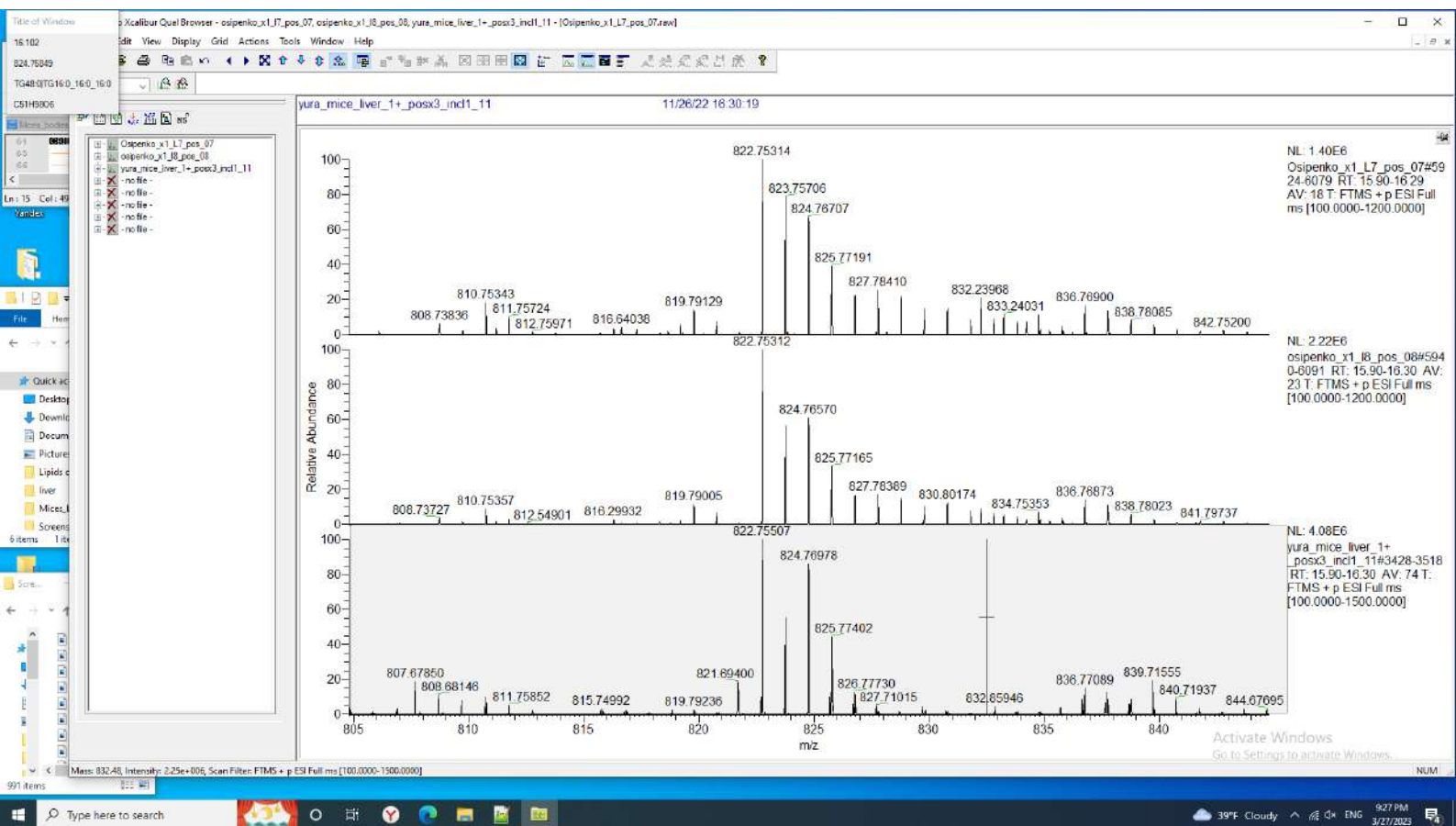

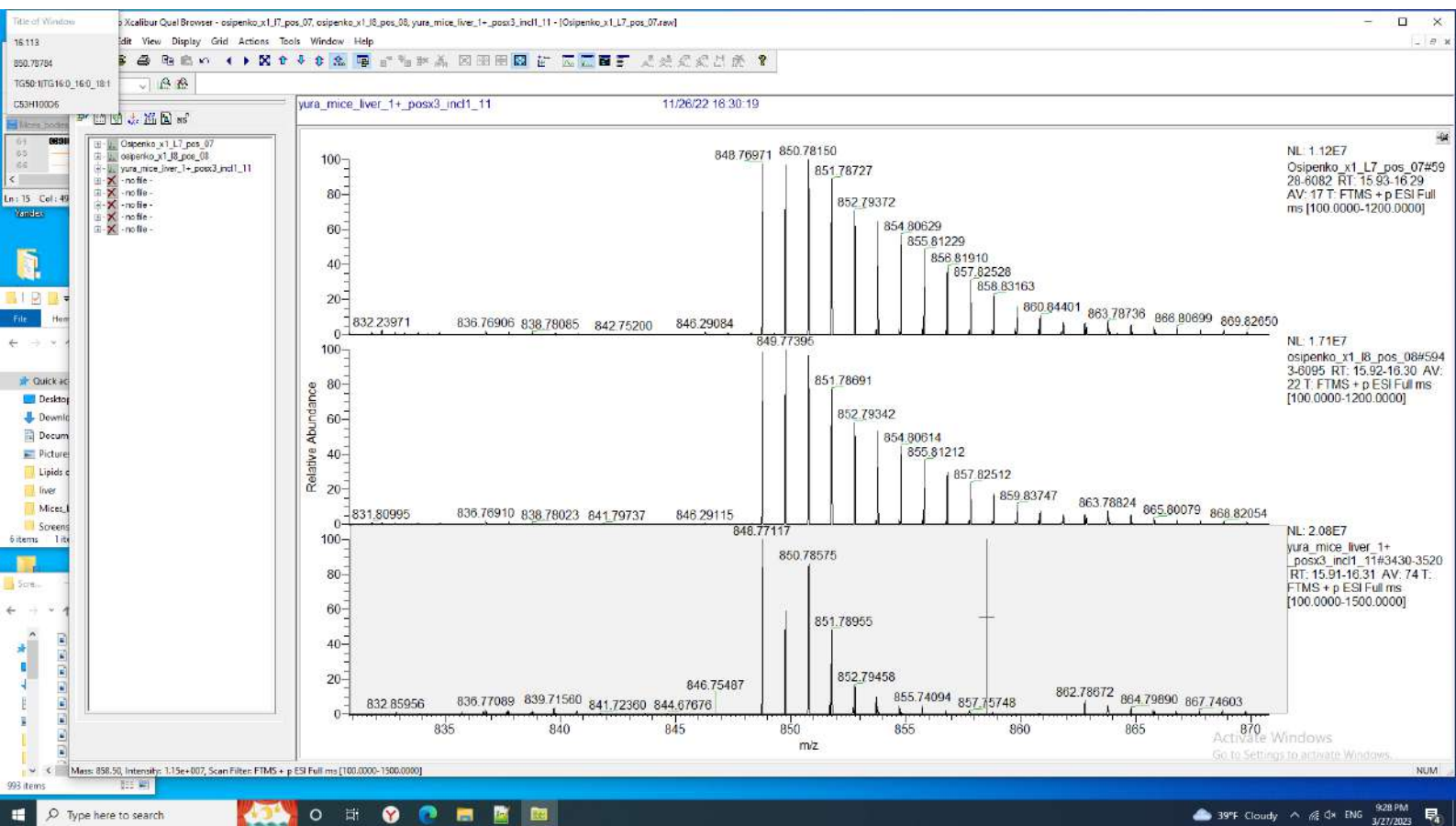

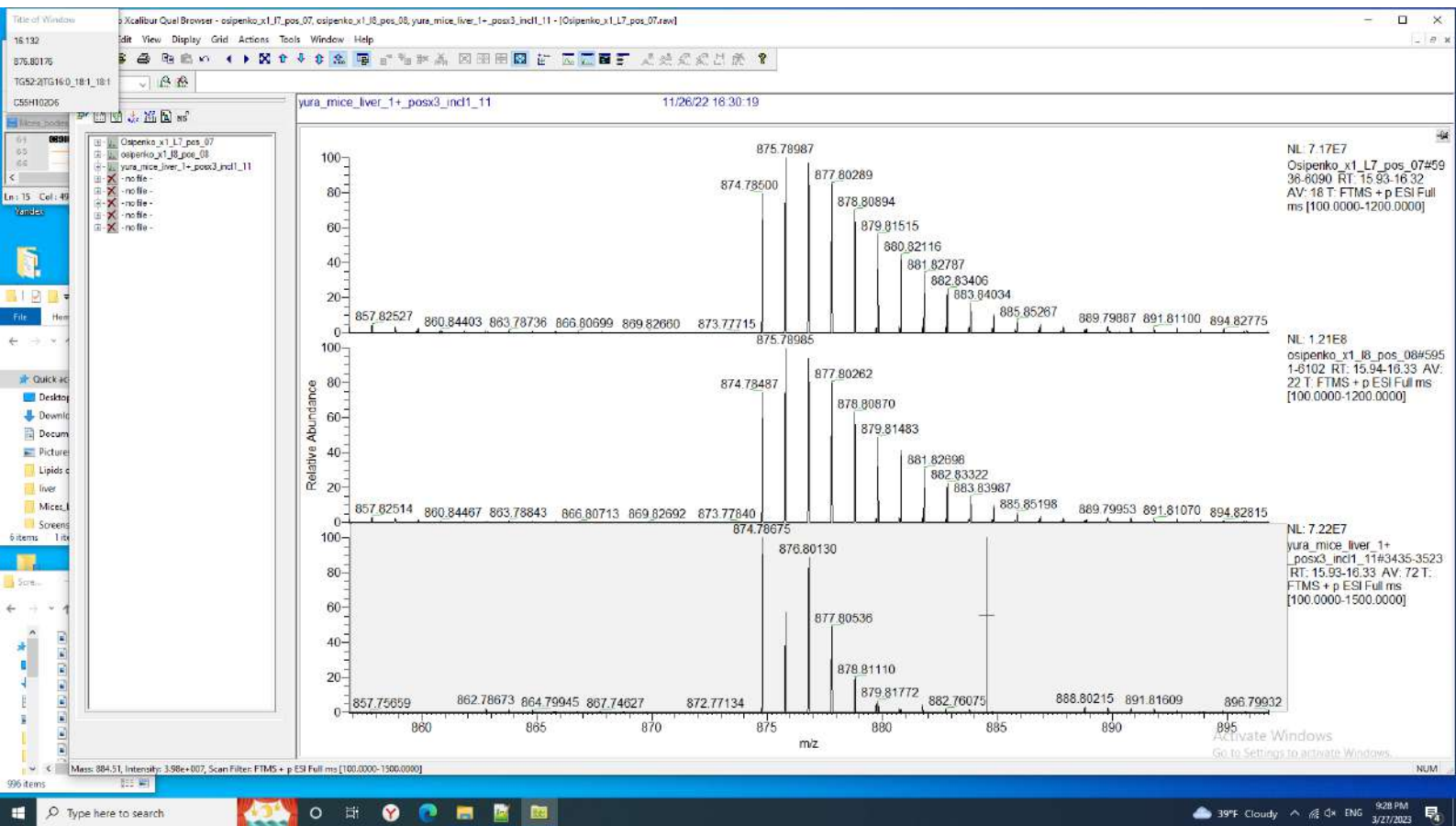

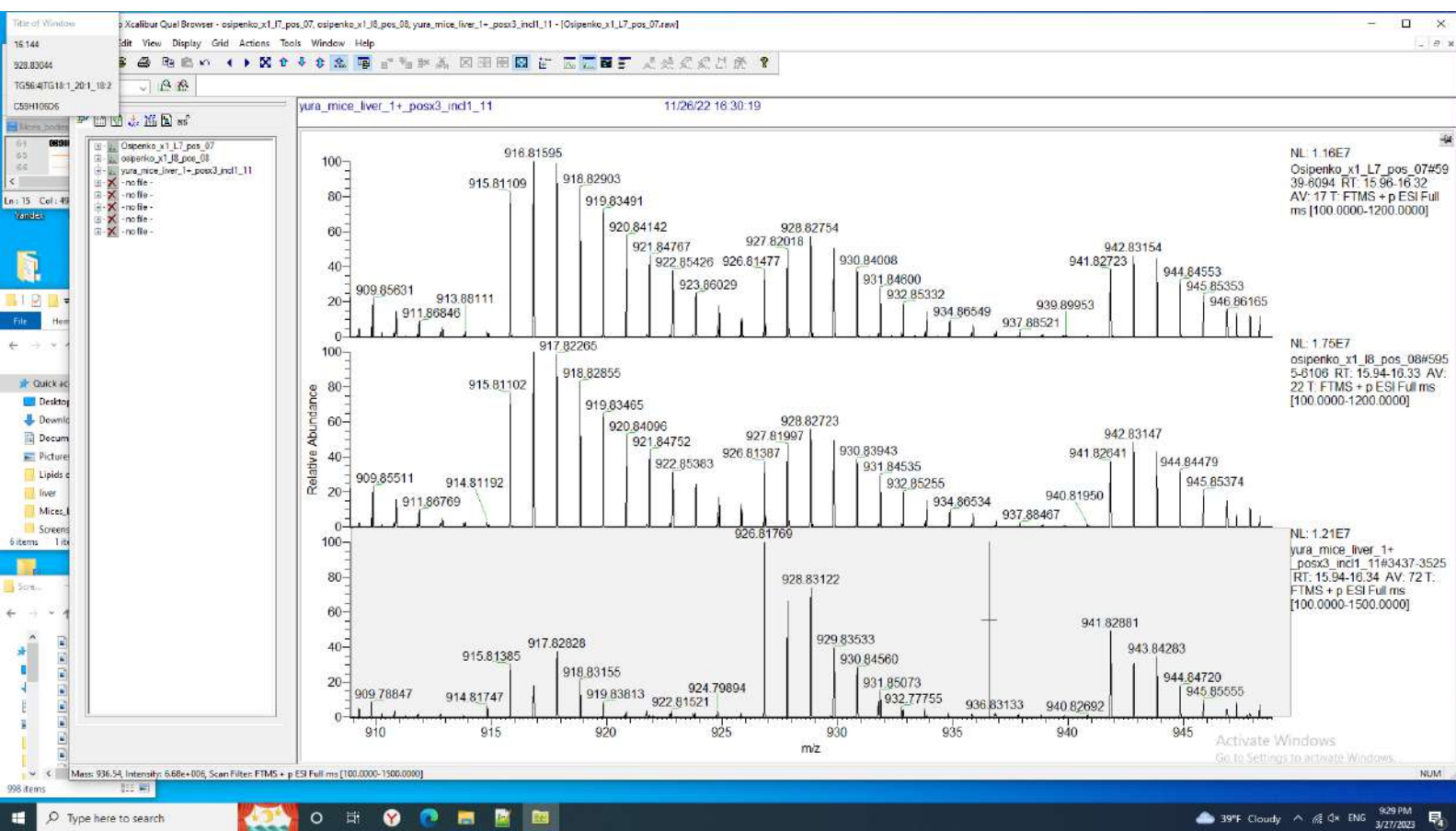

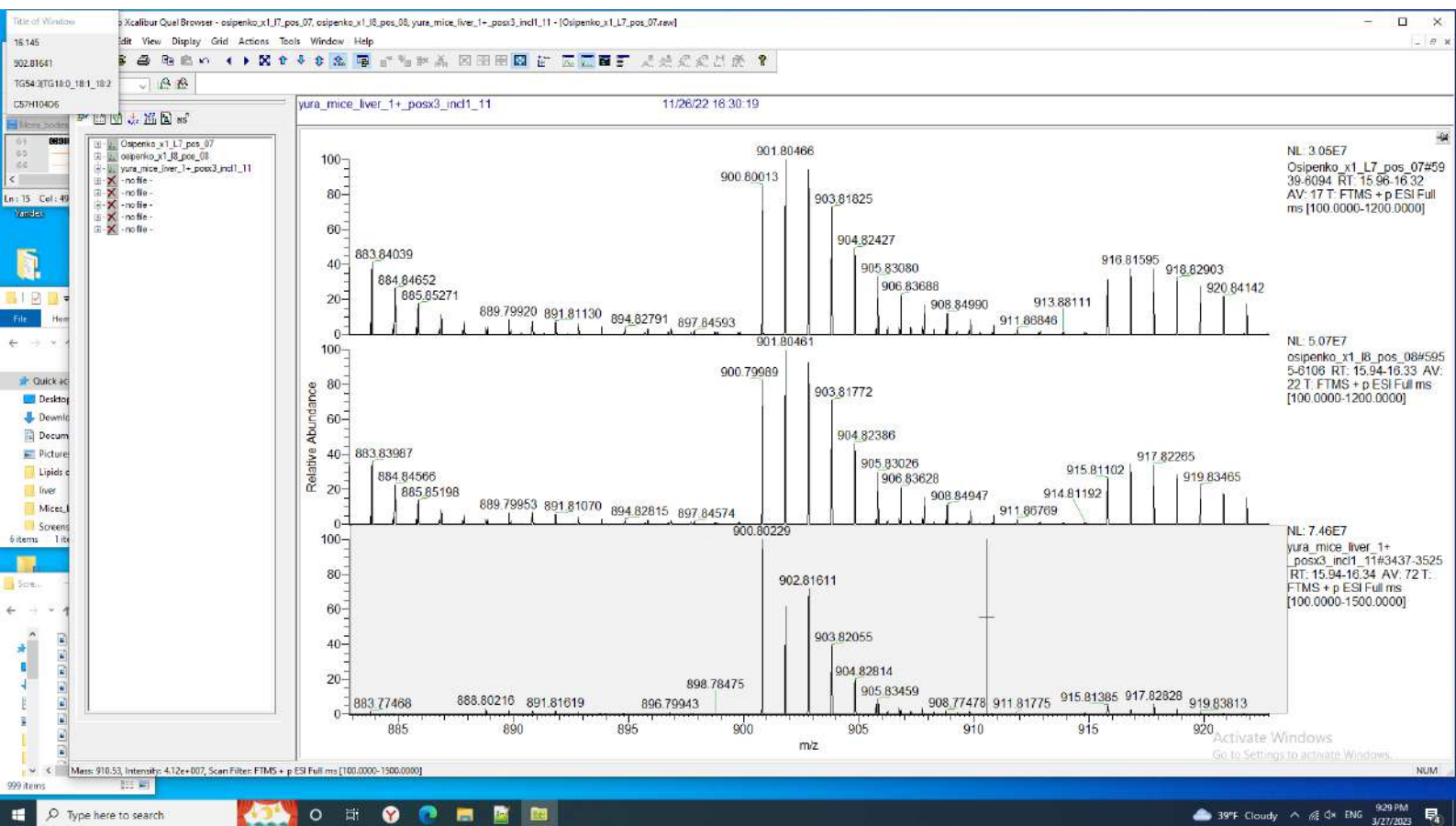

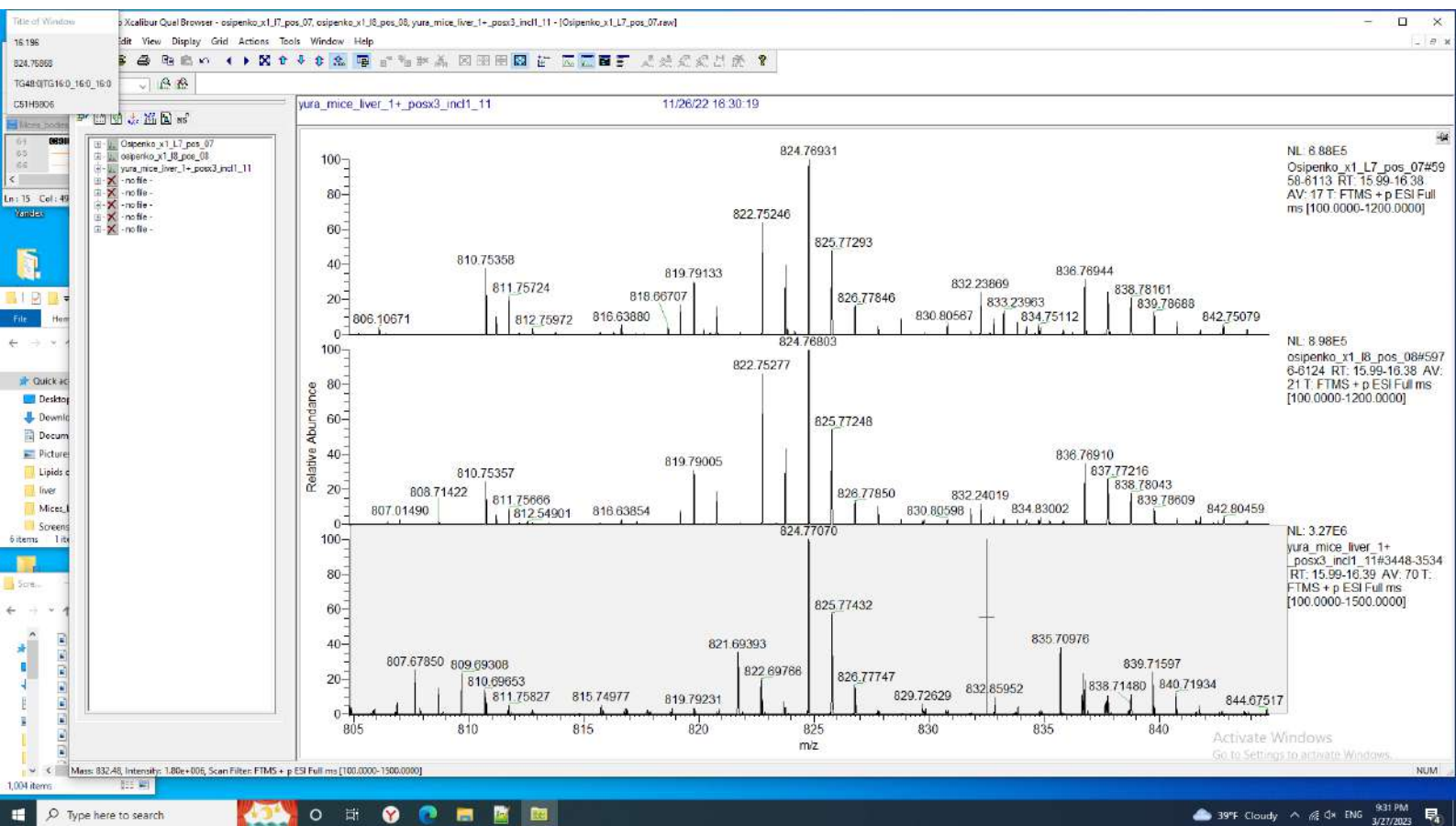

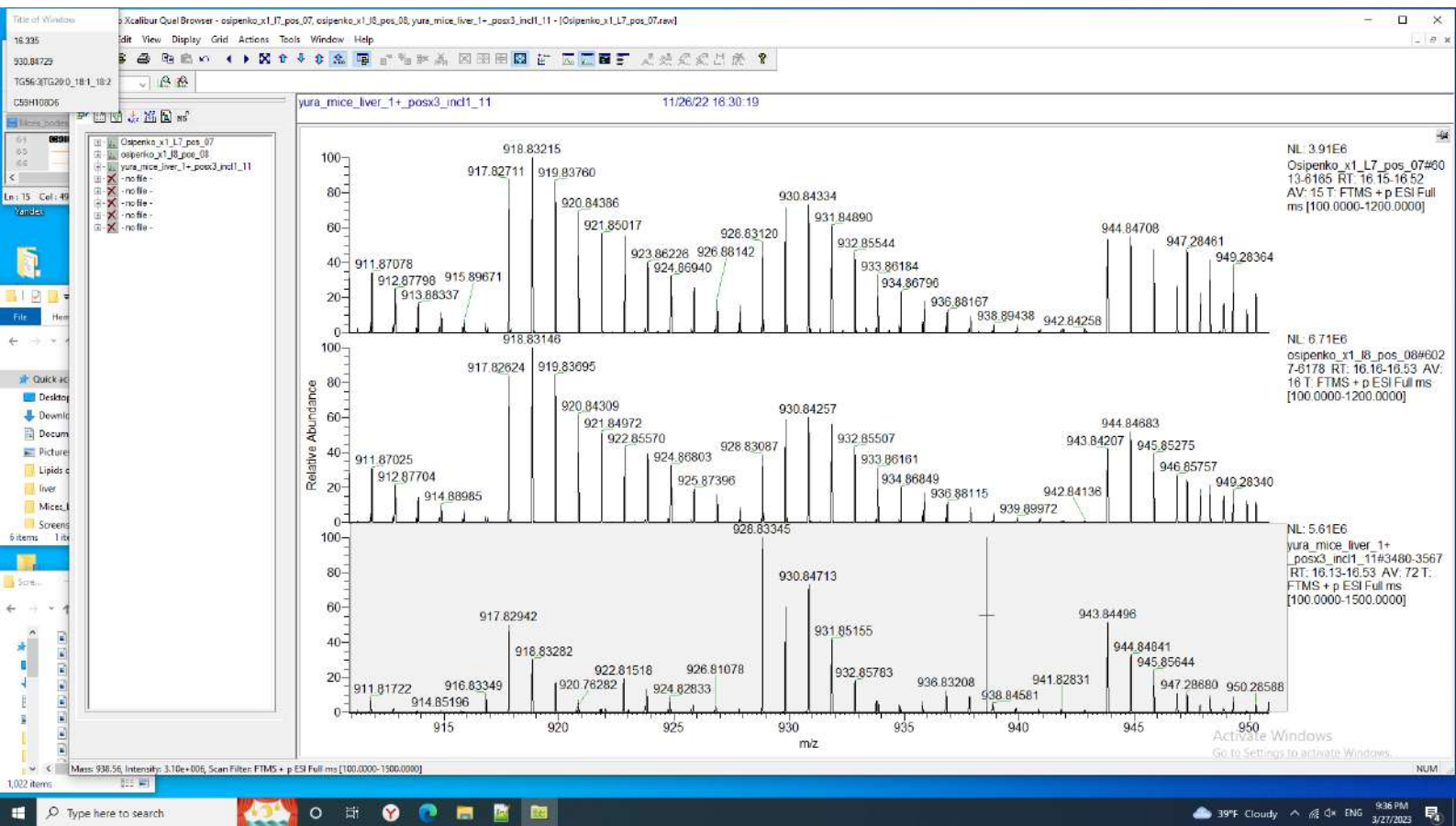

Supplement: Supplementary file 1 [file ijms-24-11725-s001.zip › Supporting Info 1. Deuterium distribution for liver.pdf]
